# Titanium‐Catalyzed Diastereoselective Keto‐ and Iminonitrile Cyclizations

**DOI:** 10.1002/chem.71206

**Published:** 2026-06-06

**Authors:** Christoph Kern, Ritu Singh, Michael A. Unkrig‐Bau, Lukas Jakob, Jan Streuff

**Affiliations:** ^1^ Department of Chemistry for Life Sciences Uppsala University Uppsala Sweden; ^2^ Institut Für Organische Chemie Albert‐Ludwigs‐Universität Freiburg Freiburg im Breisgau Germany

**Keywords:** catalysis, cyclization, diastereoselectivity, single‐electron transfer, titanium

## Abstract

Substituted five‐membered carbocycles with multiple stereocenters are common structural motifs of natural products but difficult to synthesize in a stereoselective fashion. In this study, we report a titanium(III)‐catalyzed diastereoselective cyclization of keto‐ and iminonitriles that forms oxygenated and aminated cyclopentanones in good yield and diastereoselectivity. The derivatization into cyclopentyls with three consecutive stereocenters and a cyclopentenone is demonstrated. With support by DFT calculations, a transition state model is derived that is in agreement with an earlier proposal for related ketomalononitrile cyclizations.

## Graphical abstract

The titanium(III)‐catalyzed diastereoselective cyclization of readily‐available substituted ketonitriles gives cyclopentanones in good yield and stereoselectivity. Subsequent 1,2‐addition allows the preparation of cyclopentyls with three consecutive stereocenters. A stereoselectivity model is derived based on computational studies and earlier studies of titanium‐catalyzed ketone‐nitrile cyclizations.

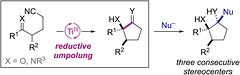

## 1 Introduction

Oxygenated and aminated cyclopentane frameworks that have several consecutive stereocenters are core motifs of numerous natural products, for example barbacenic acid, sieboldin A, rocaglamide, and pactamycin. Although advanced methods for the synthesis and functionalization of substituted cyclopentanone and cyclopentenone building blocks are well‐established, for example by means of cycloadditions as well as ionic and radical cyclizations [1, 2, 3, 4, 5, 6], preparing functionalized cyclopentyls is still challenging.

**SCHEME 1:**
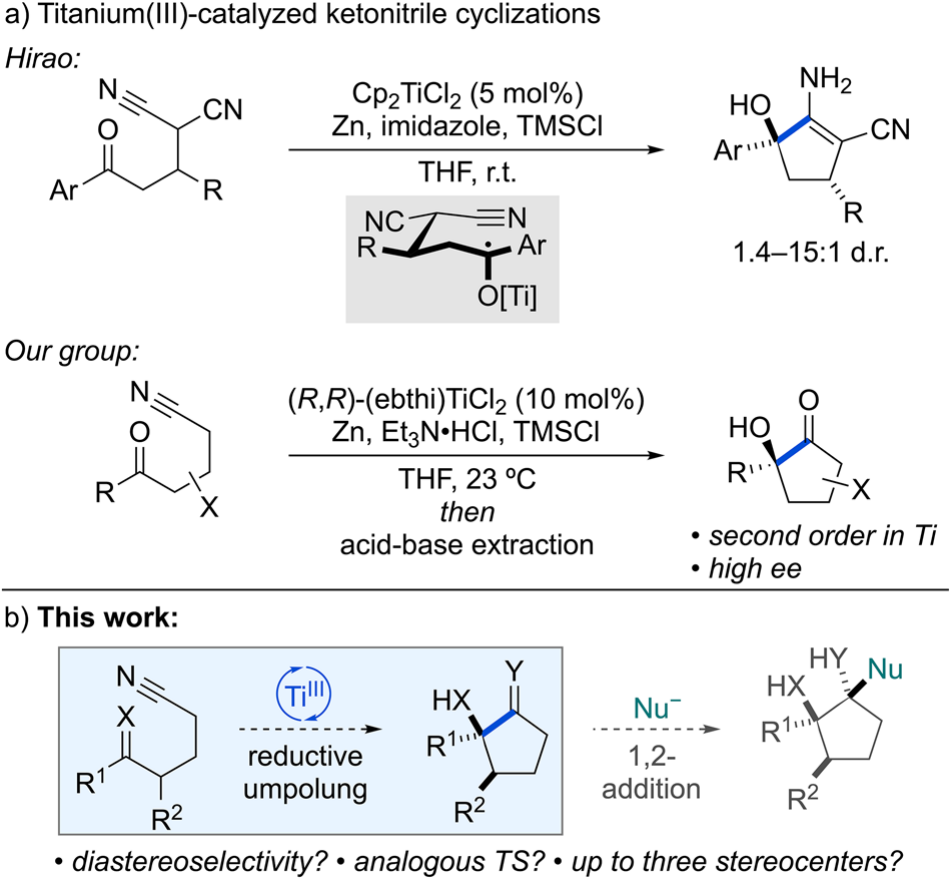
(a) Titanium‐catalyzed cyclizations established by Hirao and us. (b) Diastereoselective strategy to multisubstituted cyclopentyls pursued herein.

Titanium(III)‐catalysis has enabled a large number of radical C–C couplings and cyclizations that are complementary to late transition metal chemistry and proceed under excellent catalyst control [7, 8, 9, 10, 11, 12, 13, 14, 15, 16, 17, 18, 19, 20, 21, 22, 23, 24, 25, 26, 27, 28, 29, 30, 31, 32, 33, 34, 35, 36]. Following earlier SmI_2_‐promoted examples [37, 38, 39, 40], Hirao reported a diastereoselective titanium(III)‐catalyzed cyclization of β‐substituted δ‐ketomalononitriles to 2‐amino‐3‐cyano‐cyclopentenols that proceeded with moderate‐to‐good diastereoselectivity and for which a Beckwith–Houk analogous selectivity model was proposed (Scheme 1a) [41]. Our group developed a series of related titanium(III)‐catalyzed cyclizations that give access to a broad range of functionalized five‐membered ring systems, including a mild asymmetric α‐hydroxyketone synthesis as well as related imine‐nitrile cyclizations to α‐aminoketones, pyrrolidin‐3‐ones, 3‐aminoindoles, and other heterocycles [15, 42, 43, 44]. This ketone‐nitrile coupling was shown to proceed with a higher order in catalyst due to two catalyst species being coordinated to the substrate in the C–C coupling step [26, 39, 45, 46, 47]. We now report the expansion of this strategy towards corresponding diastereoselective keto‐ and iminonitrile cyclizations that give α‐oxygenated and aminated cyclopentanones with two neighboring stereocenters in high diastereoselectivity (Scheme 1b). The combination with a following stereoselective 1,2‐addition then gives access to five‐membered carbocycles with three consecutive stereocenters. In addition, we address the question whether the original selectivity model can be applied to these cyclizations as well.

## 2 Results and Discussion

Our investigations started with readily‐available ketonitrile **1** that was submitted to the cyclization conditions established earlier by us, consisting of Cp_2_TiCl_2_ (10 mol%) as catalyst, zinc as terminal reducing agent, and chlorotrimethylsilane as turnover‐enabling reagent (Table 1) [42]. We observed a good *cis‐*diastereoselectivity for product **2**, but only a moderate yield was achieved and dehydroxylated cyclopentanone **3** was obtained as byproduct (entry 1). Motivated by the earlier observation that additives, in particular amine hydrochlorides, have a strong effect on the catalyst stability and the stereoselectivity of titanium(III)‐catalyzed reactions [17, 42, 45, 48], we investigated the effect of various amine hydrochlorides. The addition of triethylamine hydrochloride had a beneficial influence on the reaction yield but slightly lowered the diastereoselectivity to 6:1 (entry 2). At this point, a brief survey of titanocene catalysts was carried out and Cp_2_TiCl_2_ was found to be superior to Cp*_2_TiCl_2_ and (ebthi)TiCl_2_, for example, both of which gave inferior yields (see the Supporting Information). Without a titanium catalyst, no reaction was observed. Further hydrochloride additives were tested and the diastereomeric ratio could be increased by switching to the bulkier DIPEA•HCl or to the more acidic Coll•HCl and Morph•HCl, the latter reaching 17:1 d.r. (entries 3–5). In addition, we tested propylamine hydrochloride and cinchonidine hydrochloride, the latter of which required *O*‐benzylation for the reaction to take place, as well as ammonium chloride (entries 6–9). We suspected that the formation of deoxygenated product **3** could have occurred through the reaction with water either during the catalysis or during workup. The titanium(III)‐promoted and catalyzed C─O cleavage of tertiary and benzylic alcohols is well‐known and water activated by a titanium(III) center is a surprisingly good hydrogen atom donor [31, 49, 50, 51, 52, 53]. The addition of 0.5 equiv of deoxygenated water to the reaction mixture led to an increased formation of **3**, which supported this hypothesis (entry 10). We further tested alternative reducing agents and Zn proved to be superior to Mn or Mg (see the Supporting Information). Finally, increasing the amount of Morph•HCl and reducing the amount of TMSCl led to conditions that gave **2** in 84%–90% yield and excellent 22:1 *cis*/*trans* ratio (entry 11).

**SCHEME 2:**
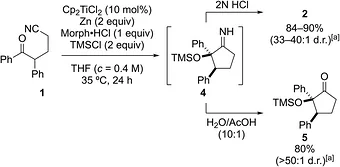
Modified workup conditions lead to *O*‐silylated product **5**. [a] Diastereomeric ratio of purified product.

Interestingly, the desilylation of the primary α‐siloxyimine product **4** during workup was slower than for products lacking the β‐aryl substituent, which readily hydrolyzed with cold 1N HCl within minutes [42]. The workup was therefore modified and carried out by stirring with 2N HCl for 1 h to achieve complete hydrolysis of both, the imine and siloxy groups (Scheme 2). Column chromatography led to further enrichment of the *cis* diastereomer and allowed the separation of the minor diastereomer for characterization purposes. The selective isolation of the TMS‐protected *cis*‐product **5** was possible after treatment of the crude mixture with water and a small amount of glacial acetic acid for 2 h followed by chromatography (Scheme 2). It was again noticed that the additional phenyl stereocenter increased the stability of the trimethylsiloxy group.

**TABLE 1:** Selected Optimization Experiments.

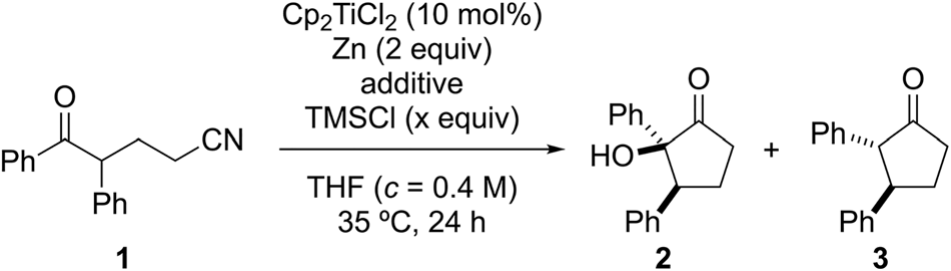					
Entry	Additive (equiv)	x	d.r. (2)^a^	Yield (2) [%]^b^	Conversion to 3 [%]^a^
1	none	3	13:1	51	6
2	Et_3_N•HCl (0.5)	3	6:1	86	3
3	DIPEA•HCl (0.5)	3	13:1	81	5
4	Coll•HCl (0.5)	3	15:1	73	8
5	Morph•HCl (0.5)	3	17:1	83	5
6	*n*‐PrNH_2_•HCl (0.5)	3	8:1	61	4
7	OBnCin•HCl (0.5)	3	15:1	75	9
9	NH_4_Cl (0.5)	3	10:1	58	6
10	Morph•HCl (0.5), H_2_O (0.5)	3	8:1	49	24
**11**	**Morph•HCl (1.0)**	**2**	**22:1** ^c^	**84–90** ^c^	**3‐5** ^c^

^a^ Determined by ^1^H NMR of the crude mixture after workup.

^b^ Yield of isolated product.

^c^ Range of two experiments. DIPEA = *N*,*N*‐diisopropylethylamine, Coll = 2,4,6‐collidine, Morph = morpholine, and OBnCin = *O*‐benzylcinchonidine.

**SCHEME 3:**
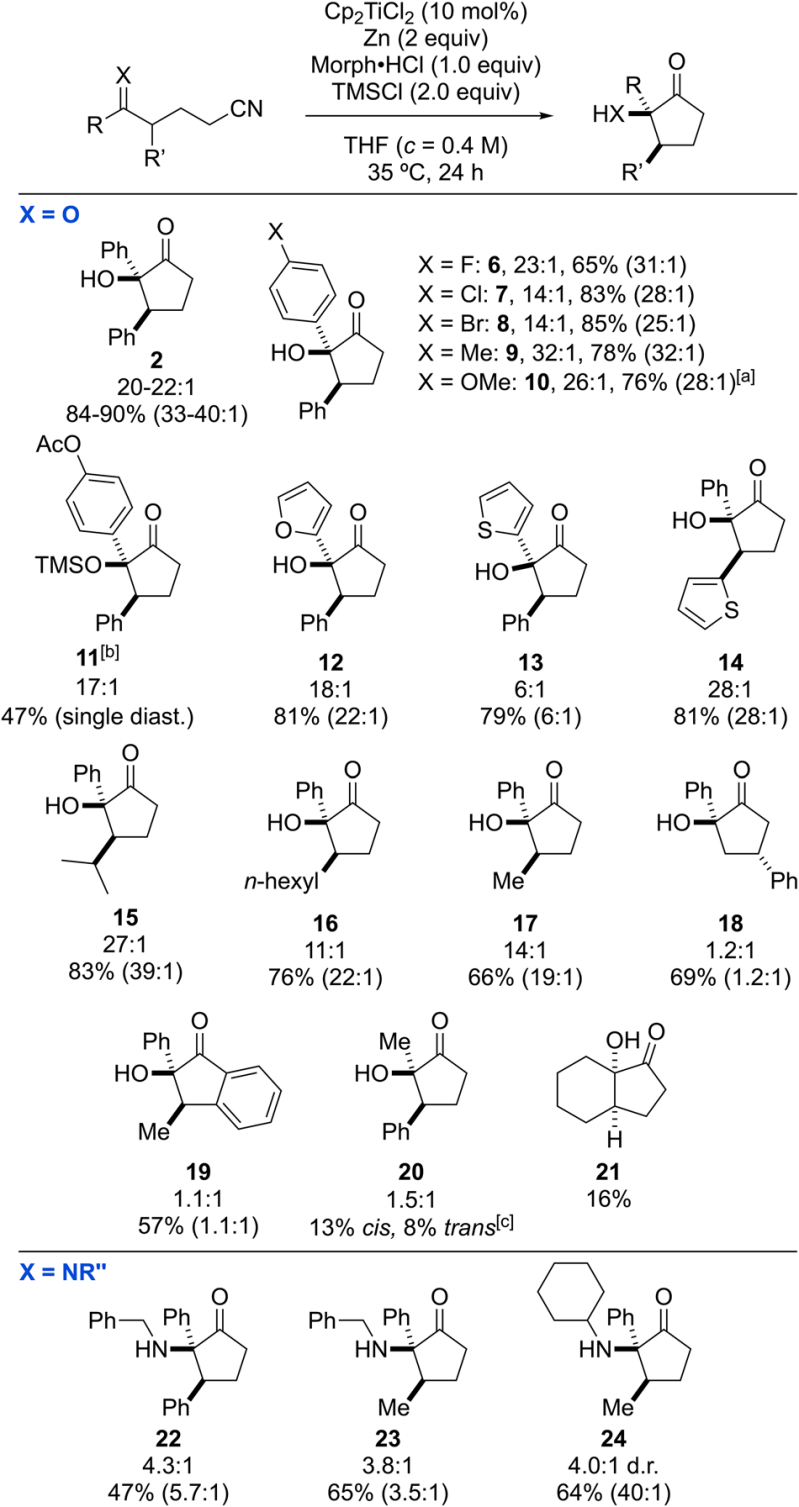
Explored scope of the diastereoselective ketonitrile and iminonitrile cyclizations on a 0.2 mmol scale. The diastereomeric ratio is reported as determined by ^1^H NMR of the crude reaction mixture in C_6_D_6_. Yields of isolated product are given. Brackets show the diastereomeric ratio after chromatography. [a] 45°C reaction temperature. [b] Workup with H_2_O/AcOH to prevent ester hydrolysis. [c] Yields of the two separated diastereomers.

We then submitted a range of substrates to the optimized conditions to explore the influence of various substituents (Scheme 3). In general, α‐substituted aryl ketones cyclized in good yield and diastereomeric ratio, the latter being further improved by chromatography. Electron‐rich substituents increased the diastereomeric ratio to about 30:1 d.r. but no clear electronic influence on the yields was observed (**6–10**). In the case of *para*‐methoxy substituted **10**, an increased reaction temperature of 45°C was found to be beneficial. An acetoxy function was tolerated as well (**11**) and, here, the mild workup with acetic acid prevented the unintended cleavage of the acetyl group and led to isolation of the silylated product. 2‐Furyl and 2‐thienyl products **12** and **13**, respectively, gave good yields but the diastereomeric ratio of the latter was significantly lower (6:1 d.r.). Swapping the phenyl and thienyl groups restored the high *cis*‐selectivity (28:1). Alkyl substituents in the α‐position of the ketone (R’) with varying bulkiness gave good results with isopropyl exceeding *n*‐hexyl and methyl groups (**15–17**). However, products **18** and **19** having a β‐phenyl substituent or a benzannulated ring resulted in no significant diasteroselectivity. For example, product **18** was obtained in a 1.2:1 diastereomeric mixture, which was in agreement with the reaction of a β‐phenyl malononitrile substrate in Hirao's earlier study [41]. Substrates with a methyl ketone or a cyclohexanone gave the cyclization products in inferior yields (**20**, **21**).

In the following, a set of three iminonitriles was prepared by straightforward condensation of the corresponding ketonitriles with either benzylamine or cyclohexylamine [43]. Application of the titanium(III)‐catalyzed cyclization conditions then gave aminoketones **22–24** in 47%–65% yield and moderate diastereoselectivity. Purification gave **24** as a single diastereomer in 64% yield.

**FIGURE 1:**
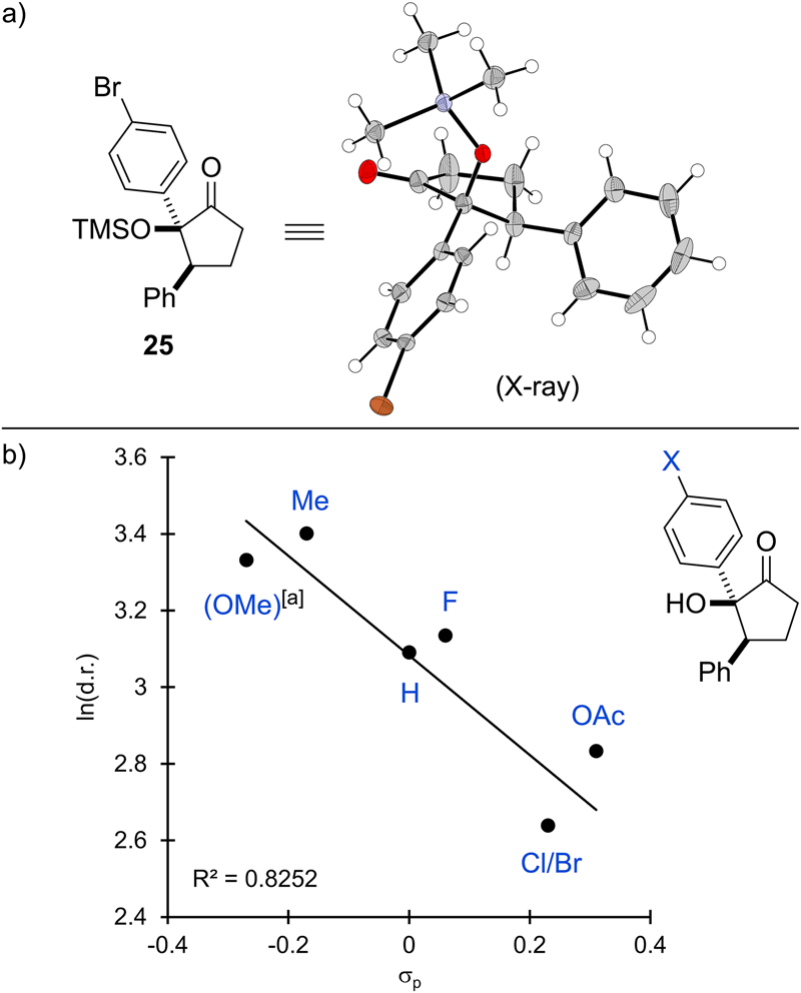
(a) Structure of **25** obtained by single crystal X‐ray diffraction analysis used for stereochemical assignment. Thermal ellipsoids are drawn at 50% probability. (b) Correlation of the observed diastereoselectivity against the Hammett constant σ_p_. [a] Reaction at 45°C instead of 35°C.

The relative configuration of the major product diastereomer was unambiguously established as *cis* (*trans* with respect to the two aryl substituents) for the *O*‐silylated 4‐bromophenyl product **25** by single crystal X‐ray diffraction analysis (Figure 1a) [54]. It was prepared in analogy to **5** and obtained in 46% yield after crystallization from *i*‐PrOH. All related products were assigned in analogy. Product **18** was assigned based on Hirao's report [41].

Besides the bulkiness of the substituents in the α‐ and β‐positions of the ketone, the electronic properties of the aryl ketone had a significant influence on the diastereoselectivity as is illustrated in Figure 1b. The logarithmic plot of the diastereomeric ratio against the Hammett parameter showed a clear linear correlation with electron‐rich aryl substituents resulting in a higher selectivity [55]. A similar positive effect of electron‐donating groups on the diastereoselectivity was observed earlier for pinacol couplings with stoichiometric amounts of Cp_2_TiCl [56].

**SCHEME 4:**
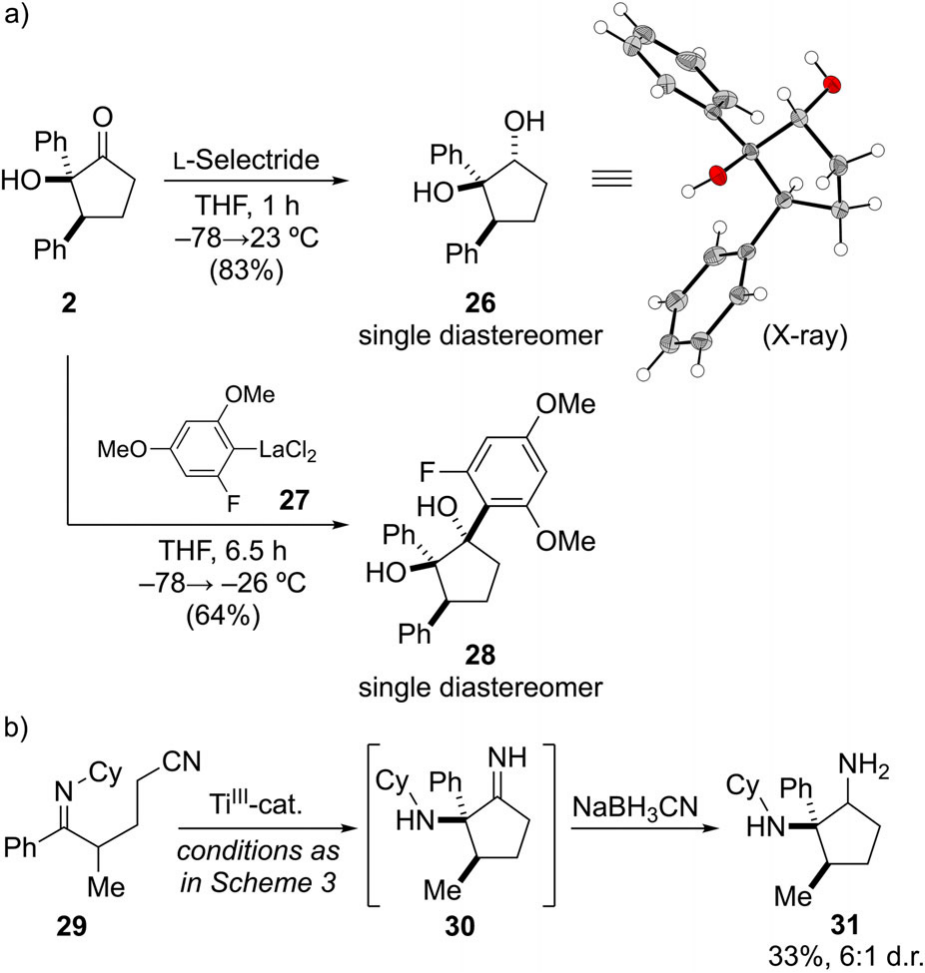
Synthesis of cyclopentyls with three consecutive stereocenters. (a) 1,2‐Additions to **2** to give *trans‐*1,2‐cyclopentanediols. (b) Imine‐nitrile cyclization/reduction sequence to give a 1,2‐diamine.

We then carried out exemplary 1,2‐addition reactions in order to test whether this could be an entry to the synthesis of cyclopentadiols and ‐diamines with three stereocenters. Previously, we could show that l‐Selectride reductions of the α‐hydroxycyclopentanone and siloxyimine products occur with high diastereoselectivity [42]. Gratifyingly, the analogous reduction of product **2** led to a single diol diastereomer **26** (Scheme 4a). The relative configuration was established by X‐ray diffraction analysis as *trans*‐*cis*. The α‐aryl substituent seems to dominate the stereoselectivity and leads to a backside attack on the carbonyl, presumably aided by chelate formation. Inspired by Tius’ rocaglamide synthesis, we further carried out the addition of aryllanthanum reagent **27** to **2** [57, 58]. The transmetalation from lithium to lanthanum was earlier found to improve 1,2‐addition yields to particularly crowded ketones [59]. The triarylated cyclopentanediol **28** was obtained in 64% yield as a single diastereomer with a presumed *trans*‐1,2‐diol configuration assigned in analogy to **27**. However, an attempt to achieve a following S_N_Ar cyclization to the corresponding benzodihydrofuran was not successful. A one‐pot imine‐nitrile cyclization of ketimine **29** followed by reduction of primary product **30** led to the isolation of 1,2‐diamine **31** as a 6:1 mixture of only two diastereomers in 33% yield (Scheme 4b). NOESY experiments of the major one indicated a *syn*‐1,2‐diamine configuration, but attempts to obtain decisive structural proof were unsuccessful. Nevertheless, these examples show the strength of our approach to access such functionalized five‐membered rings with multiple consecutive stereocenters.

**SCHEME 5:**
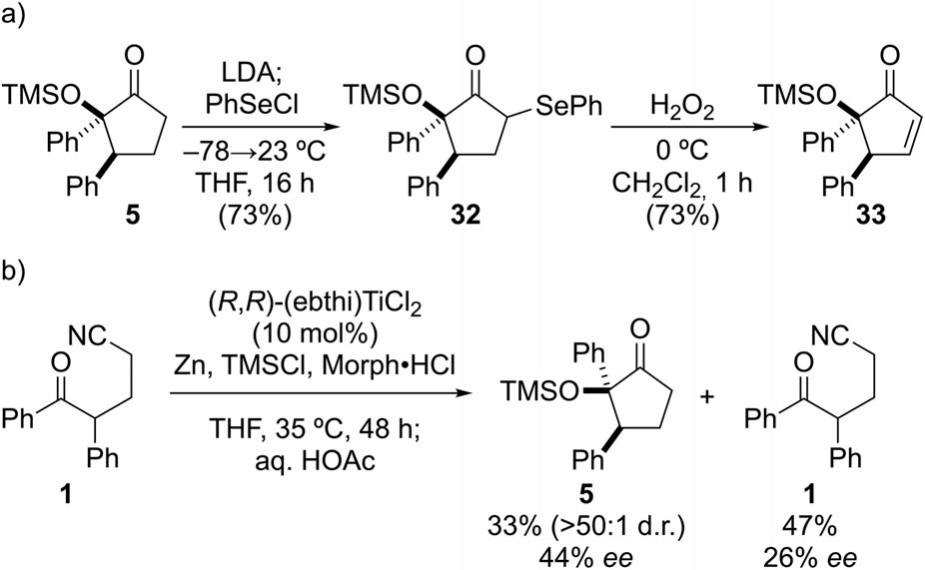
(a) Desaturation of **5** to enone **33**. (b) Tested kinetic resolution of **1** in the presence of an enantiopure titanium catalyst.

Further follow‐up studies included the oxidation of TMS‐protected cyclopentanone **5** to access the corresponding unsaturated ketone **33** in high diastereoselectivity (Scheme 5a). Interestingly, established protocols for Saegusa oxidations via the silyl enol ether were unsuccessful and the direct oxidation with IBX gave only 25% yield (see the Supporting Information) [60, 61]. Instead, conventional selenoxide pyrolysis via selenide **32** gave reliably good yields (Scheme 5a). Here, portion‐wise addition of hydrogen peroxide at 0°C together with a short reaction time of 1 h suppressed undesired Baeyer‐Villiger reaction products. Following our earlier work on enantioselective ketonitrile cyclizations, we briefly investigated whether a kinetic resolution could be achieved using (*R*,*R*)‐(ebthi)TiCl_2_ as catalyst (Scheme 5b). The cyclization of **1** followed by the mildly acidic workup conditions gave **5** in 33% yield as a single diastereomer with 44% *ee*. The remaining **1** was re‐isolated in 47% yield and 26% *ee*. Thus, a kinetic resolution is in principle possible but would require a separate optimization study.

**FIGURE 2:**
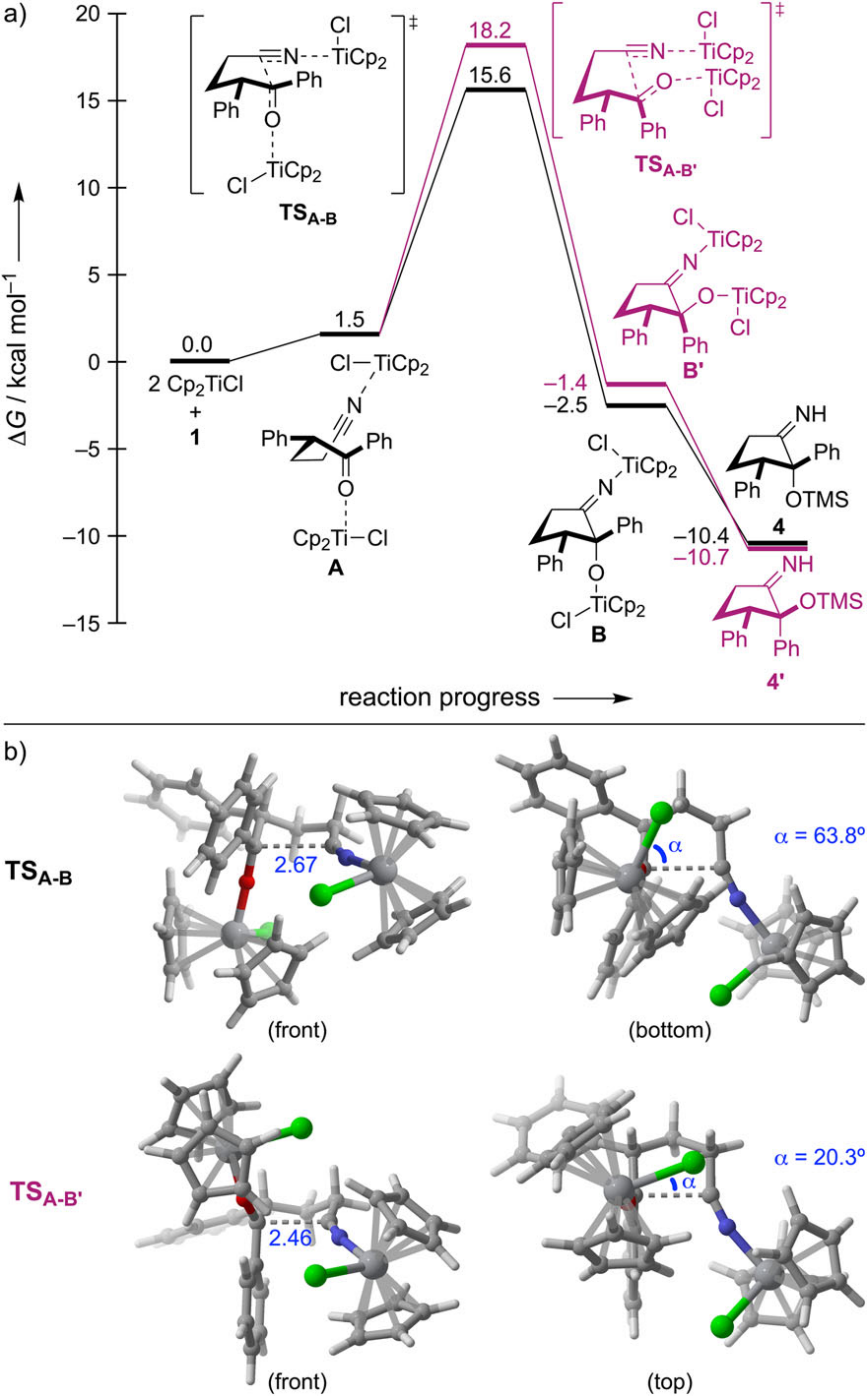
(a) Calculated reaction profile for the formation of diastereomers **4** and **4**'. (b) Visualization of the two transition state structures **TS_A‐B_** and **TS_A‐B'_** with C─C bond distances given in Å.

Finally, it was the goal to probe with the help of DFT calculations whether the originally proposed Beckwith–Houk like selectivity model was applicable considering that two titanium(III) centers participated in the C─C bond forming step [45, 46]. We therefore calculated the reaction path for the cyclization of **1** to **4** using the Orca 5.0.4 program package on the D4‐PW6B95‐RIJCOSX‐CPCM(THF)/def2‐QZVP//r^2^SCAN‐3c‐CPCM(THF) level (Figure 2a) [62, 63, 64, 65, 66, 67, 68, 69, 70, 71, 72]. The transition states of the radical‐radical coupling were calculated using the ‘broken symmetry’ formalism [73]. In addition to a CREST‐ENSO conformer search, a manual conformer analysis was carried out to localize the lowest‐energy stationary points and transition states. From substrate‐catalyst complex **A**, the two lowest‐lying transition states leading to the *cis* (**TS_A‐B_**) and the *trans* (**TS_A‐B'_**) product were located (Figure 2b). Both matched the expected substrate conformation, having the α‐phenyl groups in pseudo‐equatorial positions. Transition state **TS_A‐B_** had the Ti^III^‐ketone unit facing downwards in an orientation orthogonal to the cyclization plane and was preferred over transition state **TS_A‐B'_** by ΔΔ*G* ^‡^ = 2.6 kcal mol^−1^, which was in agreement with the observed high diastereoselectivity. Interestingly, the bond distance between the carbonyl and nitrile carbons was significantly larger in **TS_A‐B_** (2.67 Å) in comparison to **TS_A‐B'_** (2.46 Å). The reason lies in the different orientation of the oxygen‐bound titanocene unit of the former, having the chloride not directly pointing towards the nitrile carbon as in **TS_A‐B'_**. This leads to an earlier transition state with a larger Cl‐Ti‐C‐C dihedral angle α between the Ti─Cl bond and the newly formed C─C bond as indicated in Figure 2b. This conformation is favored by 2.4 kcal mol^−1^ over a transition state with a smaller dihedral angle as in **TS_A‐B'_** (see the Supporting Information). Protonation and silylation of the resulting intermediates **B** and **B'** to give product diastereomers **4** and **4'** was exergonic in both cases, leading to overall reaction energies of Δ*G* = −10.4 and −10.7 kcal mol^−1^, respectively. The lower stereoselectivity of the imine‐nitrile cyclization likely originates from the weaker Ti─N bond and the larger size of the imine, leading to a smaller energy difference of the two transition states [43]. Overall, these results were in agreement with the earlier proposal of the preferred transition state for the cyclization of β‐substituted ketomalononitriles [41]. A similar Beckwith–Houk like transition state model can thus be derived for the titanium(III)‐catalyzed cyclization of α‐substituted ketonitriles.

## 3 Conclusion

The diastereoselective titanium(III)‐catalyzed cyclization of ketonitriles and iminonitriles provides α‐hydroxyketones and α‐aminoketones in good yield and stereoselectivity. Morpholine hydrochloride was identified as the ideal additive to provide high diastereomeric ratios. The selectivity model that was established earlier by Hirao for related ketomanolonitrile cyclizations was expanded with the support of DFT calculations. The reaction serves as an entrance point for the synthesis of five‐membered carbocycles with multiple stereocenters and functional groups as was demonstrated by application in the synthesis of five‐membered diol, diamine, and enone building blocks. Since enantiomerically enriched acyclic ketonitrile precursors are accessible via established auxiliary‐based enolate alkylations [74, 75], the reaction described herein could further prove useful in asymmetric synthesis.

## Conflicts of Interest

The authors declare no conflicts of interest.

## Supporting information



The authors have cited additional references within the Supporting Information [76, 77, 78, 79, 80, 81, 82, 83, 84, 85, 86, 87, 88, 89, 90, 91, 92, 93, 94, 95, 96, 97, 98, 99, 100, 101, 102, 103, 104, 105, 106, 107, 108, 109, 110, 111, 112, 113, 114, 115, 116, 117, 118, 119, 120, 121, 122, 123, 124, 125, 126, 127]. **Supporting file**: chem71206‐sup‐0001‐SuppMat.pdf.

## Data Availability

The data that support the findings of this study are available free of charge in the Supporting Information and in the Zenodo repository at http://www.zenodo.org, reference number 19613239.

## References

[1] T. Hudlicky and J. D. Price , “Anionic Approaches to the Construction of Cyclopentanoids,” Chemical Reviews 89 (1989): 1467–1486.

[2] B. Gerard , S. Sangji , D. J. O'Leary , and J. A. Porco , “Enantioselective Photocycloaddition Mediated by Chiral Brønsted Acids: Asymmetric Synthesis of the Rocaglamides,” Journal of the American Chemical Society 128 (2006): 7754–7755. 10.1021/ja062621j 16771486

[3] B. Heasley , “Stereocontrolled Preparation of Fully Substituted Cyclopentanes: Relevance to Total Synthesis,” European Journal of Organic Chemistry 2009 (2009): 1477–1489.

[4] T. Vaidya , R. Eisenberg , and A. J. Frontier , “Catalytic Nazarov Cyclization: The State of the Art,” ChemCatChem 3 (2011): 1531–1548.

[5] D. R. Wenz and J. Read De Alaniz , “The Nazarov Cyclization: A Valuable Method to Synthesize Fully Substituted Carbon Stereocenters,” European Journal of Organic Chemistry 2015 (2015): 23–37.

[6] S. P. Simeonov , J. P. M. Nunes , K. Guerra , V. B. Kurteva , and C. A. M. Afonso , “Synthesis of Chiral Cyclopentenones,” Chemical Reviews 116 (2016): 5744–5893. 10.1021/cr500504w 27101336

[7] T. McCallum , X. Wu , and S. Lin , “Recent Advances in Titanium Radical Redox Catalysis,” The Journal of Organic Chemistry 84 (2019): 14369–14380. 10.1021/acs.joc.9b02465 31647872

[8] X. Wu , Y. Chang , and S. Lin , “Titanium Radical Redox Catalysis: Recent Innovations in Catalysts, Reactions, and Modes of Activation,” Chem 8 (2022): 1805–1821. 10.1016/j.chempr.2022.06.005 PMC9543366 36213842

[9] E. P. Beaumier , A. J. Pearce , X. Y. See , and I. A. Tonks , “Modern Applications of Low‐Valent Early Transition Metals in Synthesis and Catalysis,” Nature Reviews Chemistry 3 (2019): 15–34. 10.1038/s41570-018-0059-x PMC6462221 30989127

[10] A. R. Martínez , L. P. Morales , E. D. Ojeda , M. C. Rodríguez , and I. Rodríguez‐García , “The Proven Versatility of Cp_2_TiCl,” The Journal of Organic Chemistry 86 (2021): 1311–1329. 10.1021/acs.joc.0c01233 33147037

[11] S. P. Morcillo , D. Miguel , A. G. Campaña , L. Álvarez de Cienfuegos , J. Justicia , and J. M. Cuerva , “Recent Applications of Cp_2_TiCl in Natural Product Synthesis,” Organic Chemistry Frontiers 1 (2014): 15–33.

[12] A. Gansäuer , J. Justicia , C.‐A. Fan , D. Worgull , and F. Piestert , in Metal Catalyzed Reductive C–C Bond Formation (Ed.: M.J. Krische ), Springer Berlin Heidelberg, (2007): 25–52.

[13] T. Hirao in Metal Catalyzed Reductive C–C Bond Formation (Ed.: M.J. Krische ), Springer Berlin Heidelberg, (2007): 53–75.

[14] J. Streuff , “The Electron‐Way: Metal‐Catalyzed Reductive Umpolung Reactions of Saturated and α,β‐Unsaturated Carbonyl Derivatives,” Synthesis 45 (2013): 281–307.

[15] J. Streuff , “Reductive Umpolung and Defunctionalization Reactions through Higher‐Order Titanium(III) Catalysis,” Synlett 34 (2023): 314–326.

[16] J. Streuff and A. Gansäuer , “Metal‐Catalyzed β‐Functionalization of Michael Acceptors Through Reductive Radical Addition Reactions,” Angewandte Chemie International Edition 54 (2015): 14232–14242. 10.1002/anie.201505231 26471460

[17] A. Gansäuer , M. Behlendorf , D. von Laufenberg , et al., “Catalytic, Atom‐Economical Radical Arylation of Epoxides,” Angewandte Chemie International Edition 51 (2012): 4739–4742. 10.1002/anie.201200431 22461392

[18] J. B. Gianino and B. L. Ashfeld , “Titanocene‐Catalyzed Multicomponent Coupling Approach to Diarylethynyl Methanes,” Journal of the American Chemical Society 134 (2012): 18217–18220. 10.1021/ja308891e 23082873

[19] J. Muñoz‐Bascón , C. Hernández‐Cervantes , N. M. Padial , et al., “Ti‐Catalyzed Straightforward Synthesis of Exocyclic Allenes,” Chemistry—A European Journal 20 (2014): 801–810. 10.1002/chem.201304033 24339337

[20] Y. Zhao and D. J. Weix , “Enantioselective Cross‐Coupling of *meso*‐Epoxides with Aryl Halides,” Journal of the American Chemical Society 137 (2015): 3237–3240. 10.1021/jacs.5b01909 PMC4415026 25716775

[21] W. Hao , X. Wu , J. Z. Sun , J. C. Siu , S. N. MacMillan , and S. Lin , “Radical Redox‐Relay Catalysis: Formal [3+2] Cycloaddition of *N* ‐Acylaziridines and Alkenes,” Journal of the American Chemical Society 139 (2017): 12141–12144. 10.1021/jacs.7b06723 28825816

[22] Y. Zhang , E. Vogelsang , Z. Qu , S. Grimme , and A. Gansäuer , “Titanocene‐Catalyzed Radical Opening of N‐Acylated Aziridines,” Angewandte Chemie International Edition 56 (2017): 12654–12657. 10.1002/anie.201707673 28833905

[23] X. Wu , W. Hao , K.‐Y. Ye , et al., “Ti‐Catalyzed Radical Alkylation of Secondary and Tertiary Alkyl Chlorides Using Michael Acceptors,” Journal of the American Chemical Society 140 (2018): 14836–14843. 10.1021/jacs.8b08605 PMC6530901 30303379

[24] W. Hao , J. H. Harenberg , X. Wu , S. N. MacMillan , and S. Lin , “Diastereo‐ and Enantioselective Formal [3 + 2] Cycloaddition of Cyclopropyl Ketones and Alkenes via Ti‐Catalyzed Radical Redox Relay,” Journal of the American Chemical Society 140 (2018): 3514–3517. 10.1021/jacs.7b13710 29465998

[25] V. K. Chenniappan , S. Silwal , and R. J. Rahaim , “Ni/Ti Dual Catalytic Cross‐Coupling of Nitriles and Organobromides To Access Ketones,” ACS Catalysis 8 (2018): 4539–4544.

[26] J. Weweler , S. L. Younas , and J. Streuff , “Titanium(III)‐Catalyzed Reductive Decyanation of Geminal Dinitriles by a Non‐Free‐Radical Mechanism,” Angewandte Chemie International Edition 58 (2019): 17700–17703. 10.1002/anie.201908372 PMC6899653 31513329

[27] Z. Zhang , R. B. Richrath , and A. Gansäuer , “Merging Catalysis in Single Electron Steps With Photoredox Catalysis—Efficient and Sustainable Radical Chemistry,” ACS Catalysis 9 (2019): 3208–3212.

[28] A. Gualandi , F. Calogero , M. Mazzarini , et al., “Cp_2_TiCl_2_‐Catalyzed Photoredox Allylation of Aldehydes With Visible Light,” ACS Catalysis 10 (2020): 3857–3863.

[29] Z. Lin , Y. Lan , and C. Wang , “Titanocene‐Catalyzed Reductive Domino Epoxide Ring Opening/Defluorinative Cross‐Coupling Reaction,” Organic Letters 22 (2020): 3509–3514. 10.1021/acs.orglett.0c00960 32275154

[30] Z. Zhang , T. Hilche , D. Slak , et al., “Titanocenes as Photoredox Catalysts Using Green‐Light Irradiation,” Angewandte Chemie International Edition 59 (2020): 9355–9359. 10.1002/anie.202001508 PMC7317808 32216162

[31] H. Xie , J. Guo , Y.‐Q. Wang , et al., “Radical Dehydroxylative Alkylation of Tertiary Alcohols by Ti Catalysis,” Journal of the American Chemical Society 142 (2020): 16787–16794. 10.1021/jacs.0c07492 32885964

[32] S. Lin , Y. Chen , F. Li , C. Shi , and L. Shi , “Visible‐Light‐Driven Spirocyclization of Epoxides *via* Dual Titanocene and Photoredox Catalysis,” Chemical Science 11 (2020): 839–844. 10.1039/c9sc05601g PMC8146098 34123060

[33] M. Parasram , B. J. Shields , O. Ahmad , T. Knauber , and A. G. Doyle , “Regioselective Cross‐Electrophile Coupling of Epoxides and (Hetero)Aryl Iodides via Ni/Ti/Photoredox Catalysis,” ACS Catalysis 10 (2020): 5821–5827. 10.1021/acscatal.0c01199 PMC7398156 32747870

[34] C. Kern , J. Selau , and J. Streuff , “A Titanium‐Catalyzed Reductive α‐Desulfonylation,” Chemistry—A European Journal 27 (2021): 6178–6182. 10.1002/chem.202005400 PMC8048938 33539578

[35] Z. Zhang , J. B. Stückrath , S. Grimme , and A. Gansäuer , “Titanocene‐Catalyzed [2+2] Cycloaddition of Bisenones and Comparison With Photoredox Catalysis and Established Methods,” Angewandte Chemie International Edition 60 (2021): 14339–14344. 10.1002/anie.202102739 PMC8251790 33871126

[36] X. Pang , P.‐F. Su , and X.‐Z. Shu , “Reductive Cross‐Coupling of Unreactive Electrophiles,” Accounts of Chemical Research 55 (2022): 2491–2509. 10.1021/acs.accounts.2c00381 35951536

[37] E. J. Corey and S. G. Pyne , “Conversion of ketones having δ,ε‐π‐functions to cyclopentanols by zinc‐trimethylchlorosilane,” Tetrahedron Letters 24 (1983): 2821–2824.

[38] G. A. Molander and C. N. Wolfe , “Intramolecular Ketone−Nitrile Reductive Coupling Reactions Promoted by Samarium(II) Iodide,” The Journal of Organic Chemistry 63 (1998): 9031–9036.

[39] Y. Yamamoto , D. Matsumi , R. Hattori , and K. Itoh , “The Cp_2_TiPh‐Mediated Reductive Radical Cyclization of Cyanoketones and Related Reactions. Efficient Trapping of Ketyl Radicals by Cp_2_TiPh‐Coordinated Polar Multiple Bonds,” The Journal of Organic Chemistry 64 (1999): 3224–3229. 10.1021/jo982492s 11674424

[40] G. A. Kraus and J. O. Sy , “A Synthetic Approach to Rocaglamide via Reductive Cyclization of δ‐ketonitriles,” The Journal of Organic Chemistry 54 (1989): 77–83.

[41] L. Zhou and T. Hirao , “Stereoselective Reductive Radical Cyclization of Ketonitriles Catalyzed by Cp_2_TiCl_2_ in the Presence of Chlorosilane and Zinc,” Tetrahedron 57 (2001): 6927–6933.

[42] J. Streuff , M. Feurer , P. Bichovski , G. Frey , and U. Gellrich , “Enantioselective Titanium(III)‐Catalyzed Reductive Cyclization of Ketonitriles,” Angewandte Chemie International Edition 51 (2012): 8661–8664. 10.1002/anie.201204469 22821705

[43] G. Frey , H.‐T. Luu , P. Bichovski , M. Feurer , and J. Streuff , “Convenient Titanium(III)‐Catalyzed Synthesis of Cyclic Aminoketones and Pyrrolidinones‐Development of a Formal [4+1] Cycloaddition,” Angewandte Chemie International Edition 52 (2013): 7131–7134. 10.1002/anie.201302460 23740827

[44] L. H. Leijendekker , J. Weweler , T. M. Leuther , and J. Streuff , “Catalytic Reductive Synthesis and Direct Derivatization of Unprotected Aminoindoles, Aminopyrroles, and Iminoindolines,” Angewandte Chemie International Edition 56 (2017): 6103–6106. 10.1002/anie.201702310 28444893

[45] J. Streuff , M. Feurer , G. Frey , et al., “Mechanism of the Ti^III^‐Catalyzed Acyloin‐Type Umpolung: A Catalyst‐Controlled Radical Reaction,” Journal of the American Chemical Society 137 (2015): 14396–14405. 10.1021/jacs.5b09223 26495878

[46] J. Streuff , D. Himmel , and S. L. Younas , “Understanding Titanium‐Catalysed Radical–Radical Reactions: A DFT Study Unravels the Complex Kinetics of Ketone–Nitrile Couplings,” Dalton Transactions 47 (2018): 5072–5082. 10.1039/C8DT00643A 29561012

[47] S. L. Younas and J. Streuff , “Kinetic Analysis Uncovers Hidden Autocatalysis and Inhibition Pathways in Titanium(III)‐Catalyzed Ketone‐Nitrile Couplings,” ACS Catalysis 11 (2021): 11451–11458.

[48] A. Gansäuer , C. Kube , K. Daasbjerg , et al., “Substituent Effects and Supramolecular Interactions of Titanocene(III) Chloride: Implications for Catalysis in Single Electron Steps,” Journal of the American Chemical Society 136 (2014): 1663–1671. 10.1021/ja4121567 24397383

[49] F. Sato , Y. Tomuro , H. Ishikawa , T. Oikawa , and M. Sato , “Dealuminoxylation of Aluminum Allyl or Benzyl Alkoxides and Deoxygenation of Allyl Ethers by Lithium Aluminum Hydride in the Presence of Titanium Catalyst,” Chemistry Letters 9 (1980): 103–106.

[50] A. F. Barrero , A. Rosales , J. M. Cuerva , A. Gansäuer , and J. E. Oltra , “Titanocene‐Catalysed, Selective Reduction of Ketones in Aqueous media. A Safe, Mild, Inexpensive Procedure for the Synthesis of Secondary Alcohols via Radical Chemistry,” Tetrahedron Letters 44 (2003): 1079–1082.

[51] H. R. Diéguez , A. López , V. Domingo , et al., “Weakening C−O Bonds: Ti(III), a New Reagent for Alcohol Deoxygenation and Carbonyl Coupling Olefination,” Journal of the American Chemical Society 132 (2010): 254–259. 10.1021/ja906083c 20000601

[52] A. Gansäuer , M. Behlendorf , A. Cangönül , et al., “H_2_O Activation for Hydrogen‐Atom Transfer: Correct Structures and Revised Mechanisms,” Angewandte Chemie International Edition 51 (2012): 3266–3270. 10.1002/anie.201107556 22337565

[53] A. Căciuleanu , F. Vöhringer , and I. Fleischer , “Titanium‐Catalysed Deoxygenation of Benzylic Alcohols and Lignin Model Compounds,” Organic Chemistry Frontiers 10 (2023): 2927–2935.

[54] Deposition Numbers 2299133 and 2545933 contain the supplementary crystallographic data for this paper. These data are provided free of charge by the joint Cambridge Crystallographic Data Centre and Fachinformationszentrum Karlsruhe Access Structures service.

[55] C. Hansch , A. Leo , and R. W. Taft , “A Survey of Hammett Substituent Constants and Resonance and Field Parameters,” Chemical Reviews 91 (1991): 165–195.

[56] M. Paradas , A. G. Campaña , R. E. Estévez , et al., “Unexpected Ti^III^/Mn‐Promoted Pinacol Coupling of Ketones,” The Journal of Organic Chemistry 74 (2009): 3616–3619. 10.1021/jo9005238 19334701

[57] Z. Zhou and M. A. Tius , “Synthesis of each Enantiomer of Rocaglamide by Means of a Palladium(0)‐Catalyzed Nazarov‐Type Cyclization,” Angewandte Chemie International Edition 54 (2015): 6037–6040. 10.1002/anie.201501374 25824525

[58] Z. Zhou , D. D. Dixon , A. Jolit , and M. A. Tius , “The Evolution of the Total Synthesis of Rocaglamide,” Chemistry—A European Journal 22 (2016): 15929–15936. 10.1002/chem.201603312 27717051

[59] A. Krasovskiy , F. Kopp , and P. Knochel , “Soluble Lanthanide Salts (LnCl_3_·2 LiCl) for the Improved Addition of Organomagnesium Reagents to Carbonyl Compounds,” Angewandte Chemie International Edition 45 (2006): 497–500. 10.1002/anie.200502485 16397856

[60] R. Liffert , J. Hoecker , C. K. Jana , et al., “Withanolide A: Synthesis and Structural Requirements for Neurite Outgrowth,” Chemical Science 4 (2013): 2851.

[61] Y. Ito , T. Hirao , and T. Saegusa , “Synthesis of α,β‐unsaturated Carbonyl Compounds by Palladium(II)‐catalyzed Dehydrosilylation of Silyl Enol Ethers,” The Journal of Organic Chemistry 43 (1978): 1011–1013.

[62] F. Neese , “Software Update: The ORCA Program System—Version 5.0,” Wiley Interdisciplinary Reviews: Computational Molecular Science 12 (2022): e1606.

[63] M. Cossi , N. Rega , G. Scalmani , and V. Barone , “Energies, Structures, and Electronic Properties of Molecules in Solution With the C‐PCM Solvation Model,” Journal of Computational Chemistry 24 (2003): 669–681. 10.1002/jcc.10189 12666158

[64] F. Weigend and R. Ahlrichs , “Balanced Basis Sets of Split Valence, Triple Zeta Valence and Quadruple Zeta Valence Quality for H to Rn: Design and Assessment of Accuracy,” Physical Chemistry Chemical Physics 7 (2005): 3297–3305. 10.1039/b508541a 16240044

[65] F. Weigend , “Accurate Coulomb‐Fitting Basis Sets for H to Rn,” Physical Chemistry Chemical Physics 8 (2006): 1057–1065. 10.1039/b515623h 16633586

[66] E. Caldeweyher , C. Bannwarth , and S. Grimme , “Extension of the D3 Dispersion Coefficient Model,” Journal of Chemical Physics 147 (2017): 034112. 10.1063/1.4993215 28734285

[67] E. Caldeweyher , S. Ehlert , A. Hansen , et al., “A Generally Applicable Atomic‐Charge Dependent London Dispersion Correction,” Journal of Chemical Physics 150 (2019): 154122. 10.1063/1.5090222 31005066

[68] Y. Zhao and D. G. Truhlar , “Design of Density Functionals That Are Broadly Accurate for Thermochemistry, Thermochemical Kinetics, and Nonbonded Interactions,” Journal of Physical Chemistry A 109 (2005): 5656–5667. 10.1021/jp050536c 16833898

[69] K. Eichkorn , O. Treutler , H. Öhm , M. Häser , and R. Ahlrichs , “Auxiliary Basis Sets to Approximate Coulomb Potentials,” Chemical Physics Letters 240 (1995): 283–290.

[70] K. Eichkorn , F. Weigend , O. Treutler , and R. Ahlrichs , “Auxiliary Basis Sets for Main Row Atoms and Transition Metals and Their Use to Approximate Coulomb Potentials,” Theoretical Chemistry Accounts: Theory, Computation, and Modeling (Theoretica Chimica Acta) 97 (1997): 119–124.

[71] F. Neese , F. Wennmohs , A. Hansen , and U. Becker , “Efficient, Approximate and Parallel Hartree–Fock and Hybrid DFT Calculations. A ‘Chain‐of‐Spheres’ Algorithm for the Hartree–Fock Exchange,” Chemical Physics 356 (2009): 98–109.

[72] S. Grimme , A. Hansen , S. Ehlert , and J.‐M. Mewes , “r^2^SCAN‐3c: A ‘Swiss Army Knife’ Composite Electronic‐Structure Method,” Journal of Chemical Physics 154 (2021): 064103. 10.1063/5.0040021 33588555

[73] L. Noodleman and J. G. Norman , “The *X*α Valence Bond Theory of Weak Electronic Coupling. Application to the Low‐lying States of Mo_2_Cl_8_ ^4−^ ,” Journal of Chemical Physics 70 (1979): 4903–4906.

[74] M. R. Morales , K. T. Mellem , and A. G. Myers , “Pseudoephenamine: A Practical Chiral Auxiliary for Asymmetric Synthesis,” Angewandte Chemie International Edition 51 (2012): 4568–4571. 10.1002/anie.201200370 PMC3854953 22461381

[75] A. Job , C. F. Janeck , W. Bettray , R. Peters , and D. Enders , “The SAMP‐/RAMP‐hydrazone Methodology in Asymmetric Synthesis,” Tetrahedron 58 (2002): 2253–2329.

[76] J. Gordon , S. Hildebrandt , K. R. Dewese , et al., “Demystifying Cp_2_Ti(H)Cl and Its Enigmatic Role in the Reactions of Epoxides With Cp_2_TiCl,” Organometallics 37 (2018): 4801–4809. 10.1021/acs.organomet.8b00793 PMC6363350 30733623

[77] M. J. Di Grandi , C. Bennett , K. Cagino , A. Muccini , C. Suraci , and S. Saba , “Direct Preparation of Amides From Amine Hydrochloride Salts and Orthoesters: a Synthetic and Mechanistic Perspective,” Synthetic Communications 45 (2015): 2601–2607.

[78] K. Zumbansen , A. Döhring , and B. List , “Morpholinium Trifluoroacetate‐Catalyzed Aldol Condensation of Acetone With Both Aromatic and Aliphatic Aldehydes,” Advanced Synthesis & Catalysis 352 (2010): 1135–1138.

[79] H. G. Steeds , J. P. Knowles , W. L. Yu , J. Richardson , K. G. Cooper , and K. I. Booker‐Milburn , “Rapid Access to Azabicyclo[3.3.1]nonanes by a Tandem Diverted Tsuji–Trost Process,” Chemistry—A European Journal 26 (2020): 14330–14334. 10.1002/chem.202003762 PMC7702095 32812670

[80] T. Drennhaus , Ö. H. L , S. Djalali , S. Höfmann , C. Müller , and J. Pietruszka D Worgull , “Enantioselective Ammonium Ylide Mediated One‐Pot Synthesis of Highly Substituted *γ*‐Butyrolactones,” Advanced Synthesis & Catalysis 362 (2020): 2385–2396.

[81] H. Lütjens , A. Zickgraf , H. Figler , J. Linden , R. A. Olsson , and P. J. Scammells , “2‐Amino‐3‐benzoylthiophene Allosteric Enhancers of a_1_ Adenosine Agonist Binding: New 3, 4‐, and 5‐Modifications,” Journal of Medicinal Chemistry 46 (2003): 1870–1877. 10.1021/jm020295m 12723950

[82] D. P. Sangi , M. R. Cominetti , A. B. Becceneri , et al., “Molecular Design, Synthesis and Evaluation of 2,3‐Diarylquinoxalines as Estrogen Receptor Ligands,” Medicinal Chemistry 11 (2015): 736–746. 10.2174/1573406411666150513093039 25967047

[83] K. Pal , M. L. Behnke , and L. Tong , “A General Stereocontrolled Synthesis of *cis*‐2,3 Disubstituted Pyrrolidines and Piperidines,” Tetrahedron Letters 34 (1993): 6205–6208.

[84] Y. Li , B. Liu , H.‐B. Li , Q. Wang , and J.‐H. Li , “Oxidative Radical 1,2‐alkylarylation of Alkenes With α‐C(sp^3^)–H Bonds of Acetonitriles Involving 1,2‐aryl Migration,” Chemical Communications 51 (2015): 1024–1026. 10.1039/c4cc08902b 25446150

[85] J. Vaitla , A. Bayer , and K. H. Hopmann , “Synthesis of Indoles and Pyrroles Utilizing Iridium Carbenes Generated From Sulfoxonium Ylides,” Angewandte Chemie International Edition 56 (2017): 4277–4281. 10.1002/anie.201610520 28319303

[86] A. I. Khalaf , J. K. Huggan , C. J. Suckling , et al., “Structure‐Based Design and Synthesis of Antiparasitic Pyrrolopyrimidines Targeting Pteridine Reductase 1,” Journal of Medicinal Chemistry 57 (2014): 6479–6494. 10.1021/jm500483b PMC4136963 25007262

[87] A. Takemiya and J. F. Hartwig , “Palladium‐Catalyzed Synthesis of Aryl Ketones by Coupling of Aryl Bromides With an Acyl Anion Equivalent,” Journal of the American Chemical Society 128 (2006): 14800–14801. 10.1021/ja064782t 17105278

[88] R. Gust and V. Lubczyk , “Investigations on the Influence of Terminal Groups at the C2‐propyl Side Chain of 1,1‐bis(4‐hydroxyphenyl)‐2‐phenylpent‐1‐ene and 1,1,2‐tris(4‐hydroxyphenyl)Pent‐1‐ene on the Estrogen Receptor Binding and the Estrogenic/Anti‐estrogenic Properties,” Journal of Steroid Biochemistry & Molecular Biology 86 (2003): 57–70. 10.1016/s0960-0760(03)00253-x 12943745

[89] R. B. Chhor , K. A. Singh , B. Nosse , and V. K. Tandon , “A Convenient One‐Pot Completely Stereoselective Synthesis of *Trans*‐4‐Hydroxystilbenes and Its Derivatives and X‐Ray Structure of Its Precursor,” Synthetic Communications 33 (2003): 2519–2530.

[90] H.‐P. Lin , N. Ibrahim , O. Provot , M. Alami , and A. Hamze , “PtO_2_/PTSA System Catalyzed Regioselective Hydration of Internal Arylalkynes Bearing Electron Withdrawing Groups,” RSC Advances 8 (2018): 11536–11542. 10.1039/c8ra00564h PMC9079276 35542815

[91] R. B. Watson and C. S. Schindler , “Iron‐Catalyzed Synthesis of Tetrahydronaphthalenes via 3,4‐Dihydro‐2*H*‐pyran Intermediates,” Organic Letters 20 (2018): 68–71. 10.1021/acs.orglett.7b03367 PMC6149531 29261323

[92] M. Luo , Y. Zhang , P. Fang , et al., “H_2_O_2_‐Mediated Room Temperature Synthesis of 2‐arylacetophenones From Arylhydrazines and Vinyl Azides in Water,” Organic & Biomolecular Chemistry 20 (2022): 630–635. 10.1039/d1ob02023d 34937078

[93] J. Mazuela , T. Antonsson , L. Knerr , S. P. Marsden , R. H. Munday , and M. J. Johansson , “Iridium‐Catalyzed Asymmetric Hydrogenation of *N*‐Alkyl α‐Aryl Furan‐Containing Imines: An Efficient Route to Unnatural *N*‐Alkyl Arylalanines and Related Derivatives,” Advanced Synthesis & Catalysis 361 (2019): 578–584.

[94] Y. Kang , R. Hou , X. Min , T. Wang , Y. Liang , and Z. Zhang , “An Oxidant‐ and Catalyst‐Free Synthesis of Dibenzo[*a*,*c*]carbazoles *via* UV Light Irradiation of 2,3‐Diphenyl‐1*H*‐indoles,” Synthesis 54 (2022): 1621–1632.

[95] B. Shu , X.‐T. Wang , Z.‐X. Shen , et al., “Iridium‐catalyzed Arylation of Sulfoxonium Ylides and Arylboronic Acids: A Straightforward Preparation of α‐aryl Ketones,” Organic Chemistry Frontiers 7 (2020): 1802–1808.

[96] T. Miura , S. Fujioka , N. Takemura , et al., “Synthesis of 6‐Substituted 3‐(Alkoxycarbonyl)‐5‐aryl‐α‐pyrones,” Synthesis 46 (2014): 496–502.

[97] C.‐C. Tseng , G. Baillie , G. Donvito , et al., “The Trifluoromethyl Group as a Bioisosteric Replacement of the Aliphatic Nitro Group in CB_1_ Receptor Positive Allosteric Modulators,” Journal of Medicinal Chemistry 62 (2019): 5049–5062. 10.1021/acs.jmedchem.9b00252 PMC7871173 31050898

[98] H. Sharma , M. Kumar , A. Sethi , and B. R. Poonam , “Metal‐Free Construction of Aminated Isoquinoline Frameworks from 2‐(2‐oxo‐2‐arylethyl) benzonitrile in an Aqueous Medium,” Green Chemistry 25 (2023): 167–171.

[99] L. Tang , S. Jiang , X. Huang , et al., “Cascade of C(sp^2^)–H Addition to Carbonyl and C(sp^2^)–CN/C(sp^2^)–H Coupling Enabled by Brønsted Acid: Construction of Benzo[*a*]Carbazole Frameworks,” Organic Letters 24 (2022): 3232–3237. 10.1021/acs.orglett.2c01027 35475641

[100] B. M. Trost , K. Lehr , D. J. Michaelis , J. Xu , and A. K. Buckl , “Palladium‐Catalyzed Asymmetric Allylic Alkylation of 2‐Acylimidazoles as Ester Enolate Equivalents,” Journal of the American Chemical Society 132 (2010): 8915–8917. 10.1021/ja103771w PMC2908042 20550121

[101] W.‐B. Wu , X. Yu , J.‐S. Yu , W.‐G. W. X Wang , and J. Zhou , “Constructing Tertiary Alcohols With Vicinal Stereocenters: Highly Diastereo‐ and Enantioselective Cyanosilylation of α‐Branched Acyclic Ketones and Their Kinetic Resolution,” CCS Chemistry 4 (2022): 2140–2152.

[102] V. C. Purohit , S. P. Allwein , and R. P. Bakale , “Catalytic Oxidative 1,2‐Shift in 1,1′‐Disubstituted Olefins Using Arene(iodo)Sulfonic Acid as the Precatalyst and Oxone as the Oxidant,” Organic Letters 15 (2013): 1650–1653. 10.1021/ol400432x 23489019

[103] N. Takeda , M. Furuishi , Y. Nishijima , et al., “Chiral Isoxazolidine‐Mediated Stereoselective Umpolung α‐Phenylation of Methyl Ketones,” Organic & Biomolecular Chemistry 16 (2018): 8940–8943. 10.1039/c8ob02480d 30451259

[104] L. Cotarca , P. Delogu , A. Nardelli , et al., “Efficient and Scaleable Methods for ω‐Functionalized Nonanoic Acids: Development of a Novel Process for Azelaic and 9‐Aminononanoic Acids (Nylon‐6,9 and Nylon‐9 Precursors),” Organic Process Research & Development 5 (2001): 69–76.

[105] M. J. Fink , R. Snajdrova , A. Winninger , and M. D. Mihovilovic , “Regio‐ and Stereoselective Synthesis of Chiral Nitrilolactones Using Baeyer–Villiger Monooxygenases,” Tetrahedron 72 (2016): 7241–7248.

[106] R. C. Gadwood , I. M. Mallick , and A. J. DeWinter , “Ring Expansion of Cyclobutanones to Cyclopentanones via *α*‐lithioalkyl Aryl Sulfoxides and Selenoxides,” The Journal of Organic Chemistry 52 (1987): 774–782.

[107] A. Gao , X.‐Y. Liu , C.‐H. Ding , and X.‐L. Hou , “Diastereoselective Palladium‐Catalyzed Conjugate Addition of Arylboronic Acids to α‐Substituted Cyclic Enones,” Synlett 28 (2017): 2829–2832.

[108] N. K. Hamer , “Photoreactions of 2‐hydroxyindan‐1‐ones,” Journal of the Chemical Society, Perkin Transactions 1 (1979): 1285–1289.

[109] N. Saito , Y. Sugimura , and Y. Sato , “A Facile Construction of Bi‐ or Tricyclic Skeletons by Nickel‐Catalyzed Stereoselective Cyclization of Alkynylcycloalkanone,” Organic Letters 12 (2010): 3494–3497. 10.1021/ol101329d 20590086

[110] M. A. Bau , Albert‐Ludwigs‐Universität Freiburg (DE), 2015.

[111] F. Neese , “Software Update: The ORCA Program System, Version 4.0,” Wiley Interdisciplinary Reviews: Computational Molecular Science 8 (2017): e1327.

[112] F. Neese , F. Wennmohs , U. Becker , and C. Riplinger , “The ORCA Quantum Chemistry Program Package,” Journal of Chemical Physics 152 (2020): 224108. 10.1063/5.0004608 32534543

[113] S. Grimme , “Exploration of Chemical Compound, Conformer, and Reaction Space With Meta‐Dynamics Simulations Based on Tight‐Binding Quantum Chemical Calculations,” Journal of Chemical Theory and Computation 15 (2019): 2847–2862. 10.1021/acs.jctc.9b00143 30943025

[114] P. Pracht , F. Bohle , and S. Grimme , “Automated Exploration of the Low‐energy Chemical Space with Fast Quantum Chemical Methods,” Physical Chemistry Chemical Physics 22 (2020): 7169–7192. 10.1039/c9cp06869d 32073075

[115] CREST version 2.11, Rheinische Friedrich‐Wilhelms Universität Bonn, Bonn (Germany), 2021, https://github.com/grimme‐lab/crest/releases.

[116] XTB version 6.4.0, Rheinische Friedrich‐Wilhelms Universität Bonn, Bonn (Germany), 2021, https://github.com/grimme‐lab/xtb.

[117] S. Grimme , F. Bohle , A. Hansen , P. Pracht , S. Spicher , and M. Stahn , “Efficient Quantum Chemical Calculation of Structure Ensembles and Free Energies for Nonrigid Molecules,” Journal of Physical Chemistry A 125 (2021): 4039–4054. 10.1021/acs.jpca.1c00971 33688730

[118] J. W. Furness , A. D. Kaplan , J. Ning , J. P. Perdew , and J. Sun , “Accurate and Numerically Efficient r^2^SCAN Meta‐Generalized Gradient Approximation,” Journal of Physical Chemistry Letters 11 (2020): 8208–8215. 10.1021/acs.jpclett.0c02405 32876454

[119] J. W. Furness , A. D. Kaplan , J. Ning , J. P. Perdew , and J. Sun , “Correction to 'Accurate and Numerically Efficient r^2^SCAN Meta‐Generalized Gradient Approximation,” Journal of Physical Chemistry Letters 11 (2020): 9248. 10.1021/acs.jpclett.0c03077 33073997

[120] H. Kruse and S. Grimme , “A Geometrical Correction for the Inter‐ and Intra‐Molecular Basis Set Superposition Error in Hartree‐Fock and Density Functional Theory Calculations for Large Systems,” Journal of Chemical Physics 136 (2012): 154101. 10.1063/1.3700154 22519309

[121] R. Bauernschmitt , M. Häser , O. Treutler , and R. Ahlrichs , “Calculation of Excitation Energies Within Time‐Dependent Density Functional Theory Using Auxiliary Basis Set Expansions,” Chemical Physics Letters 264 (1997): 573–578.

[122] P. Deglmann , K. May , F. Furche , and R. Ahlrichs , “Nuclear Second Analytical Derivative Calculations Using Auxiliary Basis Set Expansions,” Chemical Physics Letters 384 (2004): 103–107.

[123] C. K. Skylaris , L. Gagliardi , N. C. Handy , G. Ioannou , S. Spencer , and A. Willets , “On the Resolution of Identity Coulomb Energy Approximation in Density Functional Theory,” Journal of Molecular Structure: THEOCHEM 501 (2000): 229–239.

[124] C. Y. Legault , CYLview 2.0 (Build0001) (Université de Sherbrooke (CA, 2020), https://www.cylview.org.

[125] J.‐M. Mouesca , Methods and Protocols, ed. J. C. Fontecilla‐Camps and Y. Nicolet (Springer, 2014), 269–296.

[126] L. Noodleman , “Valence Bond Description of Antiferromagnetic Coupling in Transition Metal Dimers,” Journal of Chemical Physics 74 (1981): 5737–5743.

[127] P. S. Bagus and B. I. Bennett , “Singlet–triplet Splittings as Obtained from the *X*α‐scattered Wave Method: A Theoretical Analysis,” International Journal of Quantum Chemistry 9 (1975): 143–148.

