# Supplementary material for: Titanium‐Catalyzed Diastereoselective Keto‐ and Iminonitrile Cyclizations
Source: Chemistry. 2026 Jun 6;32(35):e71206. doi: 10.1002/chem.71206 (PMC13587650; doi:10.1002/chem.71206)
Supplement: Supplementary file 1 [file CHEM-32-e71206-s001.pdf]

Supporting Information

Titanium-Catalyzed Diastereoselective Keto- and Iminonitrile Cyclizations

Christoph Kern,[a,b,c] Ritu Singh,[a,c] Michael A. Unkrig-Bau,[b] Lukas Jakob[a] and Jan Streuff*[a]

[a] Department of Chemistry for Life Sciences, Uppsala University, Husargatan 3, 75237 Uppsala, Sweden

[b] Institut für Organische Chemie, Albert-Ludwigs-Universität Freiburg, Albertstr. 21, 79104 Freiburg im Breisgau, Germany

[c] These authors contributed equally to this work

* Corresponding author. E-mail: jan.streuff@kemi.uu.se

Table of Contents

Materials and Methods **S2**

Hydrochloride Synthesis **S3**

Substrate Synthesis **S5**
Ketonitriles S5
Iminonitriles S15

Titanium Catalyzed Cyclizations **S17**
Representative Procedure. S17
Extended Screening Results S17
Data for Hydroxyketone Products **2**, **3**, **5–22** S20

Hammett Correlation Data **S38**
Data for Aminoketones **22–24** S38

Synthesis of 25 for Structure Determination **S43**

Product Derivatization **S43**
1,2-Addition Reactions S43
Desaturation to a Cyclopentenone S47

Attempted Kinetic Resolution Using a Chiral Catalyst **S50**

X-Ray Report for 25 ..... **S51**

X-Ray Report for 26 ..... **S56**

Computational Details **S61**
Coordinates S63

NMR Spectra **S75**

References **S170**

Materials and Methods

All reactions have been carried out in flame-dried Schlenk tubes or Schlenk flasks under an argon atmosphere and using absolute solvents unless noticed otherwise. Absolute THF was dried over sodium and benzophenone under an argon atmosphere and freshly distilled prior to use. Diethyl ether was purchased in p.a. quality from VWR. *rac*-(ebthi)$ZrCl_2$ and (*R,R*)-(ebthi)$ZrCl_2$ were purchased from mcat (www.mcat.de). All remaining chemicals were purchased from Merck or VWR. An IKAmag temperature modulator in combination with an oil bath was used to control the reaction temperatures. Thin-layer chromatography (TLC) was performed using E. Merck silica gel 60 F254 precoated plates (0.25 mm) and visualized by UV fluorescence quenching or staining ($KMnO_4$). In general, Macherey-Nagel Silica gel 60 (particle size 0.04–0.063 mm) was used for flash chromatography. NMR spectra were recorded on an Agilent MR400-DD2, 400 MHz instrument or a Bruker Avance Neo, 500 MHz instrument. Chemical shifts are reported in parts per million (δ) and referenced to the residual ^1H or ^{13}C solvent peak [$CDCl_3$: δ_H = 7.26 ppm, δ_C = 77.16 ppm or C_6D_6: δ_H = 7.16 ppm, δ_C = 128.06 ppm]. The following abbreviations were used: s = singlet, d = doublet, t = triplet, q = quartet, m = multiplet. ^{13}C-NMR spectra were recorded with {^1H} decoupling. The following abbreviations were used: s = singlet, d = doublet, t = triplet, q = quartet, m = multiplet. NMR spectra were recorded at room temperature (298–300 K) unless noted otherwise. IR spectra were recorded on a Perkin Elmer Spectrum 100 or Perkin Elmer Spectrum Two FT-IR spectrometer and are reported in frequency of absorption. Low- and high-resolution mass analyses were performed by the service department at the Institute for Organic Chemistry, University of Freiburg using a Thermo Finnigan TSQ 700 for electron impact ionization (EI) at 70 eV, 200 °C. High resolution mass analyses (HRMS, APCI) were carried out on a Thermo Exactive with Orbitrap-Analyzer using atmospheric pressure chemical ionization. High resolution mass analyses (HRMS, ESI) were carried out on a Thermo LTQ-Velos Pro Orbitrap using electrospray ionization in positive mode at the Program of Analytical Chemistry of the Department of Chemistry for Life Sciences, Uppsala University.

Hydrochloride Synthesis

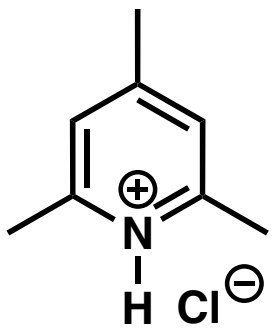

Coll·HCl

2,4,6-Collidine hydrochloride.[1] Synthesized from 2,4,6-collidine (5.29 ml, 40 mmol, 1.0 equiv), acetyl chloride (3.14 ml, 44 mmol, 1.1 equiv) and methanol (2.43 ml, 60 mmol, 1.5 equiv) following the literature procedure. The NMR data matched the literature values. ^{1}H-NMR (400 MHz, $CDCl_3$): δ = 2.52 (s, 3H), 2.91 (s, 6H), 7.19 (s, 2H).

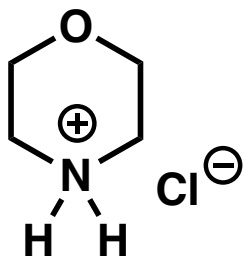

morph·HCl

Morpholine hydrochloride[2] was synthesized according to a literature procedure and obtained as white solid in 49% (709 mg, 4.89 mmol) yield. The NMR spectra matched the literature data.[3] ^{1}H-NMR (400 MHz, DMSO): δ = 3.00-3.11 (m, 4H), 3.71-3.83 (m, 4H), 9.49 (s, 2H). ^{13}C-NMR (101 MHz, DMSO): δ = 42.52, 63.13.

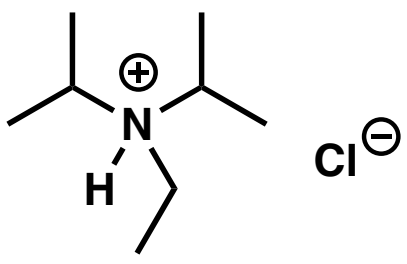

DIPEA·HCl

***N,N*-Diisopropylethylamine hydrochloride** was synthesized according to a literature procedure and obtained as white solid in 93 % (532 mg, 3.21 mmol) yield. The NMR spectra matched the literature data.[4] ^{1}H-NMR (400 MHz, $CDCl_3$): δ = 1.29-1.65 (m, 15H), 3.08 (s, 2H), 3.64 (s, 2H), 11.22 (s, 1H). ^{13}C-NMR (101 MHz, $CDCl_3$): δ = 12.22, 17.58, 18.83, 42.15, 53.79.

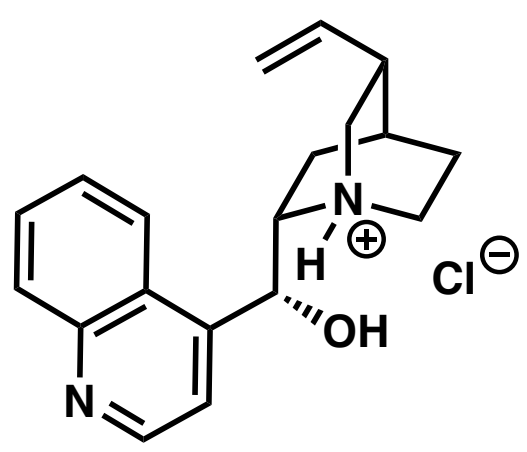

Cin·HCl

Cinchonidine hydrochloride was synthesized according to a modified literature procedure.[2] A two necked round bottom flask was equipped with a stir bar and a reflux condenser. Ammonium hydrochloride (0.134 g, 2.500 mmol, 1.000 equiv) and Cinchonidine (0.736 g, 2.500 mmol, 1.000 equiv) were suspended in absolute ethanol (9.0 mL) and the mixture was heated to reflux for 4 hours. After the yellow solution cooled down to rt a yellow precipitate formed. The precipitate was dissolved in chloroform (50 ml), filtered and the solvent was evaporated to give the pure salt as white solid in 82% (680 mg, 2.05 mmol) yield. ^{1}H-NMR (400 MHz, $CDCl_3$): δ = 1.30 (qt, J = 3.4, 10.8 Hz, 1H), 1.82 (t, J = 10.6 Hz, 1H), 2.03 (q, J = 3.8 Hz, 1H), 2.12 (dd, J = 7.2, 13.3 Hz, 1H), 2.26 (t, J = 10.9 Hz, 1H), 2.64 (q, J = 7.6 Hz, 1H), 3.02 (dd, J = 4.5, 14.6 Hz, 1H), 3.10 (dt, J = 6.1, 12.1 Hz, 1H), 3.28 (dd, J = 7.6, 9.9 Hz, 1H), 3.41 (dd, J = 10.9, 13.2 Hz, 1H), 4.52 (t, J = 11.5 Hz, 1H), 4.95 (s, 1H), 4.99 (d, J = 4.2 Hz, 1H), 5.51 (ddd, J = 6.7, 10.3, 17.1 Hz, 1H), 6.06 (br, 1H), 6.36 (s, 1H),

6.84 (t, *J* = 7.6 Hz, 1H), 7.20 (t, *J* = 7.7 Hz, 1H), 7.67 (d, *J* = 6.7 Hz, 2H), 7.91 (d, *J* = 8.4 Hz, 1H), 8.78 (d, *J* = 4.5 Hz, 1H). ^{13}C-NMR (101 MHz, $CDCl_3$): δ = 18.19, 24.53, 27.15, 37.44, 44.16, 54.94, 60.94, 66.44, 117.22, 118.72, 122.36, 124.18, 126.90, 128.73, 129.76, 137.49, 146.24, 147.40, 149.71. HRMS (pos. ESI): calcd for $C_{19}H_{23}ON_2$ $[M]^+$: 295.1805 found: 295.1804. IR (ATR): ν [cm^{-1}] = 3234, 2940, 2805, 2556, 1639, 1591, 1571, 1509, 1460, 1317, 1235, 1179, 1165, 1098, 923, 782, 753, 633.

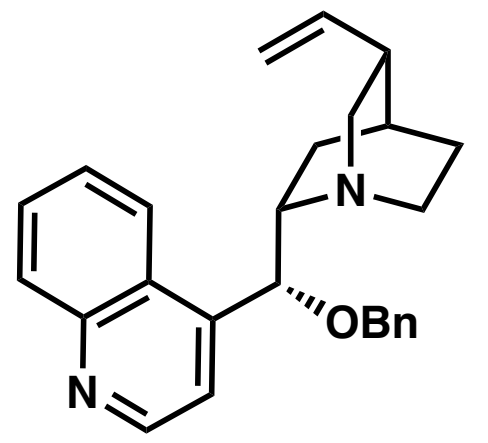

OBnCin

O-Benzylcinchonidine was synthesized according to a literature procedure and obtained as resin in 78% (460 mg, 1.2 mmol) yield. The NMR spectra matched the literature data.[5] ^{1}H-NMR (400 MHz, $CDCl_3$): δ = 1.43-1.56 (m, 1H), 1.57-1.69 (m, 1H), 1.69-1.88 (m, 3H), 2.26 (s, 1H), 2.56-2.75 (m, 2H), 3.03-3.21 (m, 2H), 3.31-3.47 (m, 1H), 4.37-4.48 (m, 2H), 4.86-4.99 (m, 2H), 5.30 (s, 1H), 5.65-5.82 (m, 1H), 7.28-7.39 (m, 5H), 7.53 (d, *J* = 4.4 Hz, 1H), 7.55-7.63 (m, 1H), 7.70-7.79 (m, 1H), 8.10-8.21 (m, 2H), 8.91 (d, *J* = 4.4 Hz, 1H). ^{13}C-NMR (101 MHz, $CDCl_3$): δ = 22.66, 27.94, 28.09, 40.20, 43.31, 57.23, 60.97, 71.46, 81.13, 114.37, 118.76, 123.36, 126.69, 126.84, 127.81, 127.90, 128.59, 129.25, 130.70, 138.00, 142.08, 146.61, 148.77, 150.35.

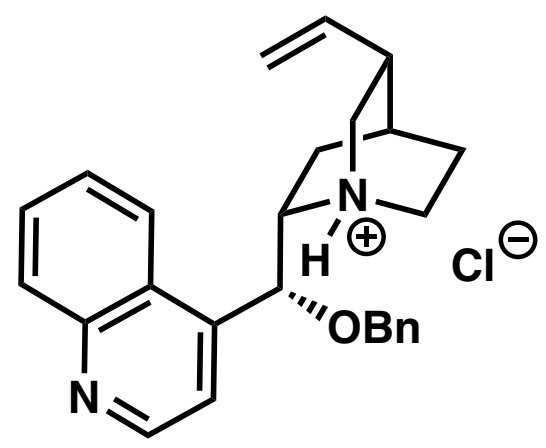

OBnCin·HCl

O-Benzylcinchonidine hydrochloride was synthesized according to a literature procedure of collidinium hydrochloride. A 100 ml Schlenk flask was flame dried and refilled with argon. O-Benzylcinchonidine (450 mg, 1.17 mmol, 1.00 equiv) was added and dissolved in 4 ml dry diethyl ether and methanol (0.071 mL, 1.75 mmol, 1.5 equiv). The mixture was cooled down to 0°C and acetyl chloride (0.092 mL, 1.28 mmol, 1.1 equiv) was added dropwise and a colorless precipitate formed. The mixture was allowed to warm up to rt and was stirred for further 30 minutes before the solid was filtered of and washed with ice cold diethyl ether (2 × 5 ml). The solid was dried under hv and the product was obtained as a pure white solid in 91% yield (451 mg, 1.07 mmol). ^{1}H-NMR (400 MHz, MeOH-d_4): δ = 1.70 (t, *J* = 12.2 Hz, 1H), 1.91-2.00 (m, 1H), 2.04 -2.14 (m, 2H), 2.30 (dd, *J* = 13.9, 7.1 Hz, 1H), 2.82 (br, 1H), 3.27-3.35 (m, 2H), 3.61 (dd, *J* = 13.1, 10.6 Hz, 1H), 3.75 (t, *J* = 9.0 Hz, 1H), 3.98 (t, *J* = 12.1 Hz, 1H), 4.57 (d, *J* = 10.9 Hz, 1H), 4.66 (d, *J* = 10.9 Hz, 1H), 5.02 (d, *J* = 10.4 Hz, 1H), 5.12 (d, *J* = 17.1 Hz, 1H), 5.76 (ddd, *J* = 17.3, 10.5, 7.1 Hz, 1H), 6.23 (br, 1H), 7.26-7.37 (m, 3H), 7.41-7.43 (m, 2H), 7.87 (t, *J* = 7.7 Hz, 1H), 7.94-7.99 (m, 2H), 8.19 (d, *J* = 8.6 Hz, 1H), 8.63 (d, *J* = 8.4 Hz, 1H), 9.02 (d, *J* = 4.8 Hz, 1H). ^{13}C-NMR (101 MHz, MeOH-d_4): δ = 20.01, 25.05, 28.20, 38.34, 45.24, 55.51, 61.35, 73.09, 76.45, 117.35, 120.75, 124.98, 127.35, 128.32, 129.47, 129.62, 129.75, 129.96, 132.72, 137.86, 138.98, 146.51, 147.84, 149.59. HRMS (pos. ESI): calcd for $C_{26}H_{29}N_2O^+$ $[M]^+$: 385.2274, found: 385.2275. IR (ATR): ν [cm^{-1}] = 3432, 3061, 3031, 2945, 2755, 2446, 1638, 1591, 1569, 1509, 1455, 1423, 1349, 1306, 1235, 1111, 1060, 1040, 923, 806, 753, 701, 638, 620, 600, 560.

Substrate Synthesis

Ketonitriles

The 4-substituted ketonitrile substrates were typically prepared in a two-step sequence consisting of a Friedel-Crafts acylation and a Michael addition to acrylonitrile as described in the following representative procedures for the synthesis of **1**.

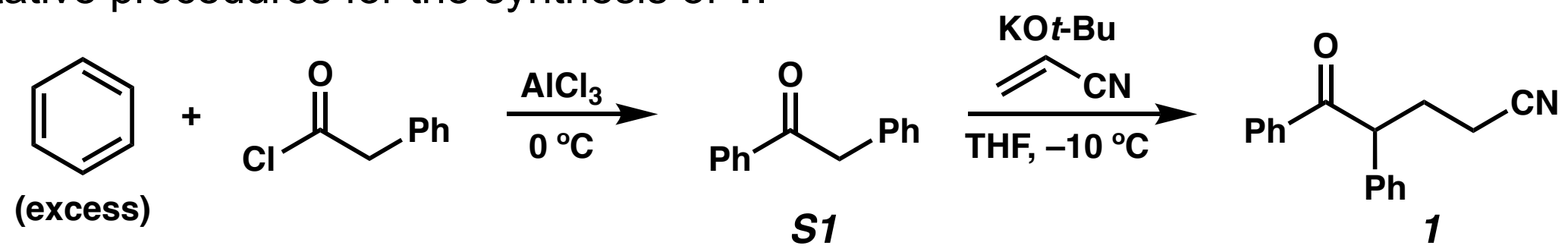

Representative procedure for the Friedel-Crafts acylation

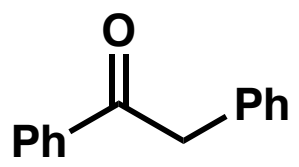

S1

1,2-Diphenylethan-1-one. The procedure was adapted from the literature.[6] A flame dried Schlenk flask was charged with phenyl acetylchloride (5.0 ml, 37.8 mmol, 1.0 equiv) and benzene (80 ml) as solvent. Aluminium chloride (8.5 g, 64.3 mmol, 1.7 equiv) was added in several portions at 0°C and the mixture was stirred at room temperature until complete conversion of the phenyl acetylchloride was observed by NMR reaction control, usually between 30 min and 2h. The mixture was poured into ice (200 ml) and was extracted with ethyl acetate (5 × 150 ml), dried over Na_2SO_4 filtered and the solvent was evaporated. The crude product was purified by crystallization from methanol (3 ml/g). The pure product was obtained as white solid in 55% yield (4.05 g, 21 mmol). The NMR data matched the literature values.[7] ^{1}H-NMR (400 MHz, $CDCl_3$): δ = 4.29 (s, 2H), 7.21-7.30 (m, 3H), 7.30-7.36 (m, 2H), 7.46 (dd, *J* = 8.4, 7.0 Hz, 2H), 7.52-7.59 (m, 1H), 8.02 (d, *J* = 8.2 Hz, 2H). ^{13}C-NMR (101 MHz, $CDCl_3$): δ = 45.66, 127.04, 128.76, 128.79, 128.82, 129.61, 133.30, 134.69, 136.76, 197.75.

Representative procedure for the Michael addition to acrylonitrile

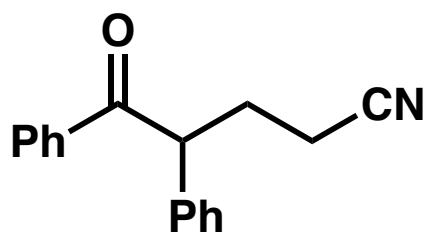

1

5-Oxo-4,5-diphenylpentanenitrile. Synthesized according to a modified literature procedure as follows.[8] A flame dried two necked Schlenk flask was charged with 1,2-diphenylethan-1-one (2.68 g, 13.7 mmol, 1.30 equiv) and dry THF (7 mL). At −10°C, KO*t*-Bu (0.10 g, 0.92 mmol, 0.09 equiv) was added, causing a color change to bright orange. A solution of acrylonitrile (0.69 mL, 10.5 mmol, 1.00 equiv) in 20 mL dry THF was added over 30 minutes and the reaction was kept at −10°C for another 15 minutes. The reaction was quenched by the addition of water (100 mL) and was diluted with ethyl acetate (100 mL). The layers were separated and the aqueous layer was washed with ethyl acetate (3 × 50 mL). The combined organic layers were dried over sodium sulfate, filtered and the solvent was evaporated under reduced pressure. The crude product was purified by column chromatography [pentane/diethyl ether, 4:1, R_F = 0.2] and was further purified by crystallization from ethanol. The pure product was obtained as white crystals in 50% yield (1.3 g, 5.3 mmol) based on acrylonitrile as limiting reagent. The NMR data matched the literature values.[9] ^{1}H-NMR (400 MHz, $CDCl_3$): δ = 2.14-2.31 (m, 2H), 2.36-2.52 (m, 2H), 4.73 (t, *J* = 7.2 Hz, 1H), 7.22-7.27 (m, 1H), 7.28-7.36 (m, 4H), 7.36-7.42 (m, 2H), 7.45-7.53 (m, 1H), 7.92-7.98 (m, 2H). ^{13}C-NMR (101 MHz, $CDCl_3$): δ = 15.37, 29.18, 52.10, 119.45, 127.98, 128.29, 128.80, 128.98, 129.59, 133.46, 136.15, 137.69, 198.34.

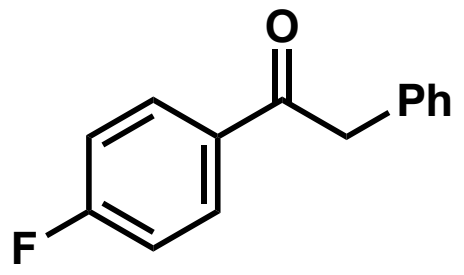

S2

1-(4-Fluorophenyl)-2-phenylethan-1-one was synthesized following the general procedure for Friedel-Crafts acylation using 2.32 g (15 mmol) of phenylacetyl chloride, 2.30 g (17.3 mmol) of $AlCl_3$ and 15 ml fluorobenzene. An additional step of washing the organic layers with 0.5 M aq. NaOH solution (2 × 50 ml) was added. After evaporation of the solvent the product was obtained as a pure white solid in 81% yield (2.60 g, 12.1 mmol). The NMR data matched the literature values.[10] ^{1}H-NMR (400 MHz, $CDCl_3$): δ = 4.26 (s, 2H), 7.12 (t, *J* = 8.6 Hz, 2H), 7.23-7.29 (m, 3H), 7.30-7.37 (m, 2H), 8.04 (dd, *J* = 8.7, 5.4 Hz, 2H).^{13}C-NMR (101 MHz, $CDCl_3$): δ = 45.65, 115.90 (d, *J* = 21.8 Hz), 127.14, 128.89, 129.52, 131.41 (d, *J* = 9.5 Hz), 133.13 (d, *J* = 3.0 Hz), 134.48, 165.89 (d, *J* = 254.9 Hz), 196.17. ^{19}F NMR (376 MHz, $CDCl_3$): δ = −105.04.

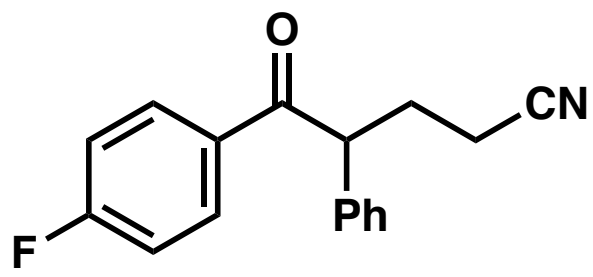

S3

5-(4-Fluorophenyl)-5-oxo-4-phenylpentanenitrile was synthesized on a 2.8 mmol scale from **S2** following the general procedure for the Michael addition to acrylonitrile. The crude product was purified by column chromatography [petroleum ether/diethyl ether, 6:1→4:1 R_f(petroleum ether/diethyl ether, 4:1) =0.2]. The pure product was obtained as white solid in 63% yield (0.47 g, 1.76 mmol). ^{1}H-NMR (400 MHz, $CDCl_3$): δ = 2.12-2.31 (m, 2H), 2.35-2.52 (m, 2H), 4.68 (t, *J* = 2.4 Hz, 1H), 6.98-7.10 (m, 2H), 7.20-7.40 (m, 5H), 7.92-8.03 (m, 2H).^{13}C-NMR (101 MHz, $CDCl_3$): δ = 15.25, 29.06, 52.06, 115.88 (d, *J* = 21.9 Hz), 119.36, 128.04, 128.15, 129.61, 131.61 (d, *J* = 9.5 Hz), 132.45 (d, *J* = 2.9 Hz), 137.51, 165.80 (d, *J* = 255.8 Hz), 196.67. ^{19}F NMR (376 MHz, $CDCl_3$) δ = −104.52-104.41 (m). HRMS (neg. ESI): calcd for $C_{17}H_{14}FNO$ $[M-H]^-$: 266.0987 found: 266.0988. MS (EI, 70 eV): m/z (%) =267.1 $[M]^+$, 267.1, 237.1, 207.0, 183.0, 144.1, 123.0, 95.0, 75.0 65.0, 51.1. IR (ATR): ν [cm^{-1}] = 3092, 3029, 2937, 2246, 1679, 1595, 1505, 1453, 1409, 1356, 1260, 1231, 1201, 1178, 1155, 1103, 1073, 966, 859, 843, 769, 743, 701, 604, 586, 551.

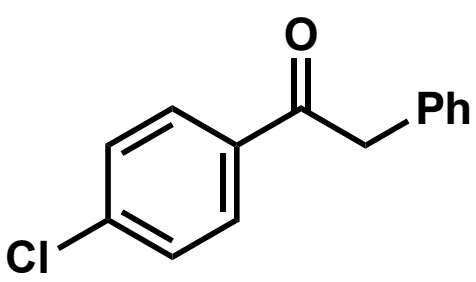

S4

1-(4-Chlorophenyl)-2-phenylethan-1-one was synthesized following the general procedure for Friedel-Crafts acylation using 1.55 g (10.0 mmol) phenylacetylchloride, 1.73 g (13.0 mmol) $AlCl_3$ and 10.0 mL chlorobenzene. The crude product was purified by recrystallization in methanol. The pure product was obtained as white solid in 52% yield (1.20 g, 5.20 mmol). The NMR data matched the literature values.[6] ^{1}H-NMR (400 MHz, $CDCl_3$): δ = 4.25 (s, 2H), 7.22-7.29 (m, 3H), 7.30-7.36 (m, 2H), 7.43 (d, *J* = 8.6 Hz, 2H), 7.95 (d, *J* = 8.6 Hz, 2H). ^{13}C-NMR (101 MHz, $CDCl_3$): δ = 45.69, 127.19, 128.91, 129.11, 129.51, 130.19, 134.32, 135.01, 139.77, 196.54.

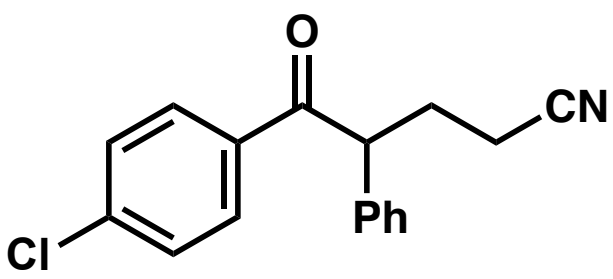

S5

5-(4-Chlorophenyl)-5-oxo-4-phenylpentanenitrile was synthesized on a 4.7 mmol scale from **S4** following the general procedure for the Michael addition to acrylonitrile. The crude product was purified by column chromatography [petroleum ether/ diethyl ether, 4:1 R_f = 0.3]. The pure product was obtained as colorless oil in 39 % yield (0.53 g, 1.8 mmol). ^{1}H-NMR (400 MHz, $CDCl_3$): δ = 2.09-2.32 (m, 2H), 2.33-2.52 (m, 2H), 4.61-4.71 (m, 1H), 7.22-7.30 (m, 3H), 7.30-7.39 (m, 4H), 7.88 (d, *J* = 8.7 Hz, 2H).^{13}C-NMR (101 MHz, $CDCl_3$): δ = 15.28, 29.02, 52.17, 119.35, 128.13, 128.20, 129.10, 129.69, 130.35, 134.37, 137.37, 139.91, 197.09. HRMS (neg. ESI): calcd for $C_{17}H_{14}ClNO$ $[M+Cl]^-$: 318.0458 found: 318.0459. MS (EI, 70 eV): m/z (%) =283.1 $[M]^+$, 165.1, 165, 141.1, 139.0, 111.0, 75.0. IR (ATR): ν[cm^{-1}] = 3062, 3029, 2937, 2245, 1679, 1586, 1570, 1489, 1453, 1424, 1400, 1359, 1317, 1260, 1233, 1202, 1173, 1092, 1011, 987, 963, 875, 856, 825, 769, 751, 731, 700, 678, 628.

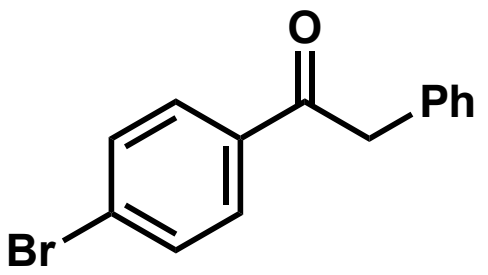

S6

1-(4-Bromophenyl)-2-phenylethan-1-one was synthesized following the general procedure for Friedel-Crafts acylation using 1.55 g (10.0 mmol) phenylacetylchloride, 1.73 g (13.0 mmol) $AlCl_3$ and 10.0 mL bromobenzene. The crude product was purified by column chromatography (petroleum ether/ethyl acetate, 8:1, R_f =0.4) The pure product was obtained as white solid in 56% yield (1.53 g, 5.5 mmol). The NMR data matched the literature values.[11] ^{1}H-NMR (400 MHz, $CDCl_3$): δ = 4.25 (s, 2H), 7.20-7.37 (m, 5H), 7.60 (d, *J* = 8.6 Hz, 2H), 7.87 (d, *J* = 8.6 Hz, 2H). ^{13}C-NMR (101 MHz, $CDCl_3$): δ = 45.67, 127.19, 128.51, 128.91, 129.51, 130.29, 132.10, 134.28, 135.40, 196.73.

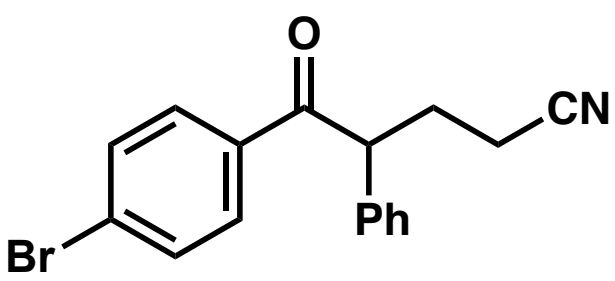

S7

5-(4-Bromophenyl)-5-oxo-4-phenylpentanenitrile was synthesized on a 2.8 mmol scale from **S6** following the general procedure for the Michael addition to acrylonitrile. The crude product was purified by column chromatography [petroleum ether/ diethyl ether, 6:1→4:1 R_f (petroleum ether/ diethyl ether, 4:1 = 0.2]. The pure product was obtained as white solid in 63% yield (0.578 g, 1.76 mmol). ^{1}H-NMR (400 MHz, $CDCl_3$): δ = 2.12-2.30 (m, 2H), 2.34-2.50 (m, 2H), 4.65 (t, *J* = 7.2 Hz, 1H), 7.22-7.29 (m, 3H), 7.30-7.36 (m, 2H), 7.51 (d, *J* = 8.6 Hz, 2H), 7.80 (d, *J* = 8.6 Hz, 2H).^{13}C-NMR (101 MHz, $CDCl_3$): δ = 15.25, 28.98, 52.14, 119.33, 128.12, 128.18, 128.67, 129.68, 130.43, 132.08, 134.76, 137.31, 197.27. HRMS (neg. ESI): calcd for $C_{17}H_{13}BrON$ $[M]^-$: 326.0186, found: 326.0190. MS (CI): m/z (%) = 328.0 $[M+H]^+$, 301.0, 267.1, 250.1, 199.9, 182.9, 165.0, 144.1, 105.0, 93.0, 76.0. IR (ATR): ν[cm^{-1}] = 3062, 3029,2936, 2245, 1740, 1679, 1583, 1487, 1453, 1396, 1355, 1258, 1230, 1173, 1070, 1008, 964, 822, 768, 745, 702, 590, 551, 516.

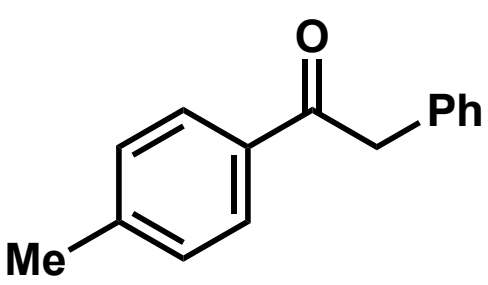

S8

2-Phenyl-1-(*p*-tolyl)ethan-1-one was synthesized following the general procedure for Friedel-Crafts acylation using 2.32 g (15.0 mmol) phenylacetylchloride, 2.20 g (16.0 mmol) $AlCl_3$ and 15.0 mL toluene. The crude product was purified by column chromatography (petroleum ether/ diethyl ether, 8:1, R_f =0.4). The pure product was obtained as white solid in 73% yield (2.30 g, 11 mmol). The NMR data matched the literature values.[6] ^{1}H-NMR (400 MHz, $CDCl_3$): δ = 2.40 (s, 3H), 4.26 (s,

2H), 7.22-7.36 (m, 7H), 7.92 (d, *J* = 7.9 Hz, 2H). ^{13}C-NMR (101 MHz, $CDCl_3$): δ = 21.62, 45.41, 126.79, 128.62, 128.75, 129.31, 129.41, 134.12, 134.77, 143.96, 197.26.

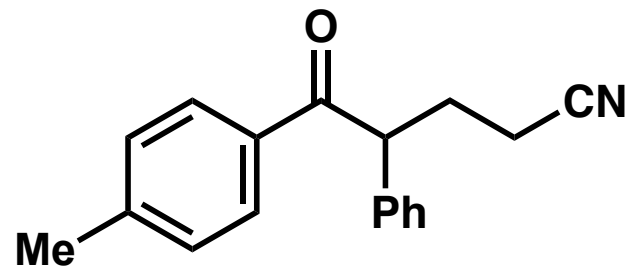

S9

5-Oxo-4-phenyl-5-(*p*-tolyl)pentanenitrile was synthesized on a 4.7 mmol scale from **S8** following the general procedure for the Michael addition to acrylonitrile. The crude product was purified by column chromatography [petroleum ether/diethyl ether, 4:1 R_f =0.3] and was further purified by recrystallization in ethanol. The pure product was obtained as white crystals in 54% yield (0.78 g, 2.9 mmol). ^{1}H-NMR (400 MHz, $CDCl_3$): δ =2.12-2.31 (m, 2H), 2.31-2.52 (m, 5H), 4.71 (t, *J* = 7.3 Hz, 1H), 7.18 (d, *J* = 8.0 Hz, 2H), 7.21-7.41 (m, 5H), 7.86 (d, *J* = 8.3 Hz, 2H). ^{13}C-NMR (101 MHz, $CDCl_3$): δ = 15.36, 21.74, 29.17, 51.92, 119.48, 127.86, 128.24, 129.09, 129.47, 129.51, 133.62, 137.94, 144.36, 197.90. HRMS (pos. ESI): calcd for $C_{18}H_{17}ON$ $[M+H]^+$: 264.1383 found: 264.1380. MS (EI, 70 eV): m/z (%) =263.1 $[M]^+$, 178, 165, 144, 119, 117, 115, 104, 91, 65. IR (ATR): ν [cm^{-1}] = 3092, 2937, 2245, 1675, 1605, 1572, 1492, 1453, 1424, 1408, 1344, 1320, 1300, 1263, 1237, 1199, 1174, 1119, 1072, 1032, 962, 875, 818, 788, 766, 740, 701, 605, 587, 551.

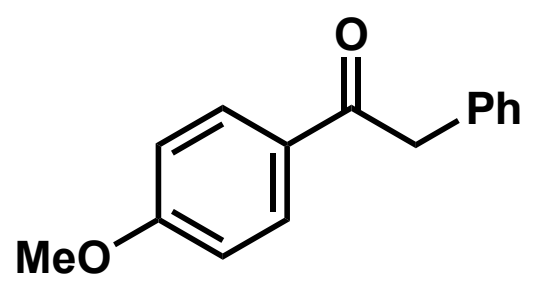

S10

1-(4-Methoxyphenyl)-2-phenylethan-1-one was synthesized according to a modified general procedure for Friedel-Crafts acylation. A flame dried Schlenk flask was charged with anisole (15 ml, 138 mmol, 9.0 equiv) and aluminium chloride (2.2 g, 17 mmol, 1.1 equiv). At 0°C phenyl acetylchloride (2.3 g, 15 mmol, 1.0 equiv) was added dropwise over 10 minutes and the reaction was stirred for 30 minutes until a full conversion of the starting material was observed by NMR reaction control. The mixture was poured into ice (100 ml) and was extracted with ethyl acetate (5 × 70 ml), dried over Na_2SO_4 filtered and the solvent was evaporated. The crude product was purified by recrystallization in methanol. The pure product was obtained as white solid in 76% yield (2.5 g, 11.3mmol). The NMR data matched the literature values.[12] ^{1}H-NMR (400 MHz, $CDCl_3$): δ = 3.85 (s, 3H), 4.24 (s, 2H), 6.93 (d, *J* = 8.8 Hz, 2H), 7.20-7.35 (m, 5H), 8.01 (d, *J* = 8.8 Hz, 2H). ^{13}C-NMR (101 MHz, $CDCl_3$): δ = 45.39, 55.59, 113.92, 126.89, 128.76, 129.51, 129.77, 131.07, 135.11, 163.64, 196.34.

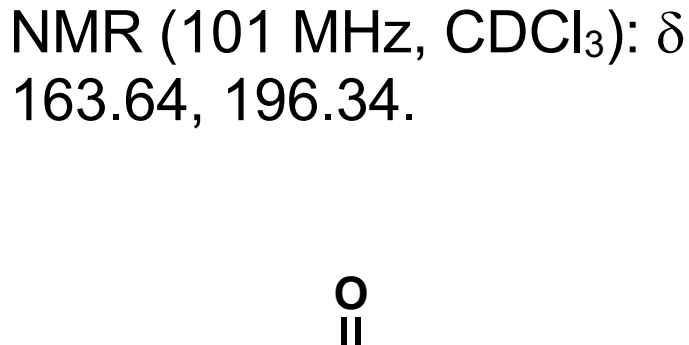

S11

5-(4-methoxyphenyl)-5-oxo-4-phenylpentanenitrile was synthesized on a 7.0 mmol scale from **S10** following the general procedure for the Michael addition to acrylonitrile. The crude product was purified by column chromatography [petroleum ether/ diethyl ether, 4:1.5 R_f =0.25]. The pure product was obtained as colorless oil in 82 % yield (1.6 g, 5.7 mmol). The NMR data matched the literature values.[13] ^{1}H-NMR (400 MHz, $CDCl_3$): δ = 2.09-2.31 (m, 2H), 2.33-2.50 (m, 2H), 3.79 (s, 3H), 4.68 (t, *J* = 7.2 Hz, 1H), 6.85 (d, *J* = 8.9 Hz, 2H), 7.19-7.27 (m, 1H), 7.28-7.34 (m, 4H), 7.94 (d, *J* = 8.9 Hz, 2H).^{13}C-NMR (101 MHz, $CDCl_3$): δ = 15.30, 29.16, 51.64, 55.51, 113.90, 119.48, 127.75, 128.12, 129.04, 129.42, 131.23, 138.14, 163.67, 196.68.

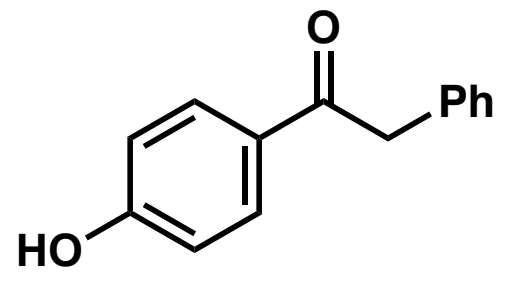

S12

1-(4-Hydroxyphenyl)-2-phenylethan-1-one was synthesized according to a modified literature procedure.[14] A flame dried Schlenk flask was charged with phenyl 2-phenylacetate (3.5 g, 16.5 mmol, 1.0 equiv) and 20 ml dichloroethane as solvent. Aluminium chloride (3.96 g, 29.7 mmol, 1.8 equiv) was added and the reaction was stirred for 5h at 60°C until a full conversion of the starting material was observed by NMR reaction control. The mixture was poured into ice (100 ml) and was extracted with ethyl acetate (5 × 70 ml), dried over Na_2SO_4 filtered and the solvent was evaporated. The crude product was purified by column chromatography (petroleum ether/ ethyl acetate, 3:1, R_f = 0.15). The pure product was obtained as colorless solid in 33 % yield (1.15 g, 5.4 mmol). The NMR data matched the literature values.[15] ^{1}H-NMR (400 MHz, $CDCl_3$): δ = 4.24 (s, 2H), 5.97 (s, 1H), 6.85 (d, J = 8.5 Hz, 2H), 7.14-7.38 (m, 5H), 7.95 (d, J = 8.8 Hz, 2H). ^{13}C-NMR (101 MHz, $CDCl_3$): δ = 45.40, 115.59, 127.00, 128.82, 129.54, 129.78, 131.48, 134.95, 160.43, 196.88.

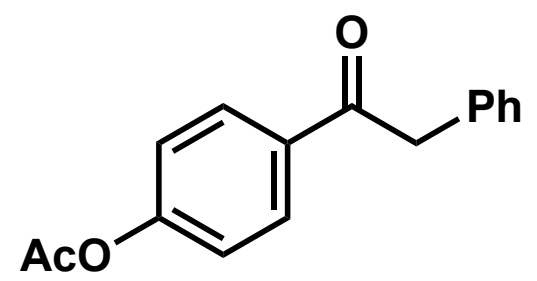

S13

4-(2-Phenylacetyl)phenyl acetate was synthesized from **S12** according to a literature.[16] The pure product was obtained as a white solid after the aqueous workup in 91% yield (500 mg, 1.97 mmol). The NMR data matches the literature values.[17] ^{1}H-NMR (400 MHz, $CDCl_3$): δ = 2.32 (s, 3H), 4.27 (s, 2H), 7.16-7.21 (m, 2H), 7.23-7.28 (m, 3H), 7.30-7.36 (m, 2H), 8.00-8.09 (m, 2H). ^{13}C-NMR (101 MHz, $CDCl_3$): δ = 21.31, 45.68, 121.98, 127.13, 128.89, 129.56, 130.42, 134.32, 134.49, 154.52, 168.96, 196.47.

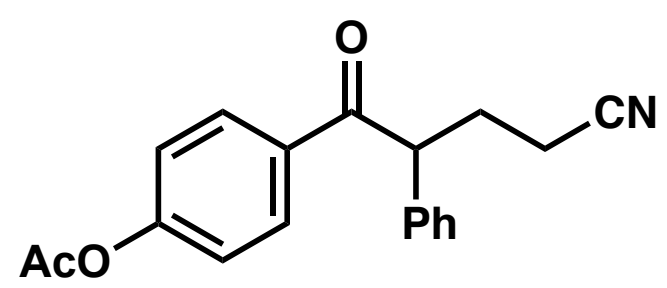

S14

4-(4-Cyano-2-phenylbutanoyl)phenyl acetate was synthesized on a 2.0 mmol scale from **S13** following the general procedure for the Michael addition to acrylonitrile. The crude product was purified by column chromatography [pentane/diethyl ether, 4:1→3:1, R_f(pentane/diethyl ether 3:1) = 0.15]. The pure product was obtained as yellow oil in 18 % yield (0.36 g, 0.358 mmol). ^{1}H-NMR (400 MHz, $CDCl_3$): δ = 2.09-2.27 (m, 2H), 2.29 (s, 3H), 2.32-2.50 (m, 2H), 4.68 (t, J = 7.2 Hz, 1H), 7.12 (d, J = 8.4 Hz, 2H), 7.22-7.36 (m, 5H), 7.99 (d, J = 8.5 Hz, 2H). ^{13}C-NMR (101 MHz, $CDCl_3$): δ = 15.29, 21.23, 29.15, 52.10, 77.16, 119.37, 121.93, 128.05, 128.19, 129.62, 130.62, 133.59, 137.44, 154.52, 168.81, 196.94. HRMS (pos. APCI): calcd for $C_{19}H_{18}NO_3$ $[M+H]^+$: 308.1281, found: 308.1276. MS (CI): m/z (%) = 325.2 $[M+NH_4]^+$, 308.1, 283.2, 266.1, 225.1, 180.1, 163.0, 121.0, 104.1 IR (ATR): ν [cm^{-1}] = 3063, 3029, 2939, 2245, 1760, 1679, 1598, 1498, 1453, 1411, 1368, 1319, 1299, 1194, 1162, 1073, 1109, 1012, 912, 857, 744, 702, 672, 591, 521.

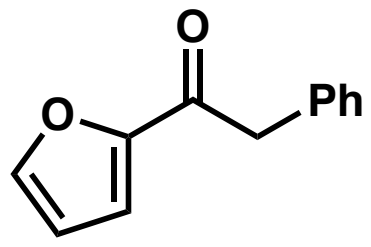

S15

1-(Furan-2-yl)-2-phenylethan-1-one was synthesized according to the literature on a 12 mmol scale. The crude product was purified using column chromatography (petroleum ether/ethyl acetate, 10:1, R_f = 0.25). The pure product was obtained as a white solid in 80% yield (1.79 g, 9.61 mmol). The NMR data matches the literature values.[18] ^{1}H-NMR (400 MHz, $CDCl_3$): δ = 4.12 (s, 2H), 6.49-6.56 (m, 1H), 7.19-7.36 (m, 6H), 7.57-7.63 (m, 1H). ^{13}C-NMR (101 MHz, $CDCl_3$): δ = 45.56, 112.54, 118.00, 127.14, 128.79, 129.64, 134.21, 146.69, 152.55, 186.73.

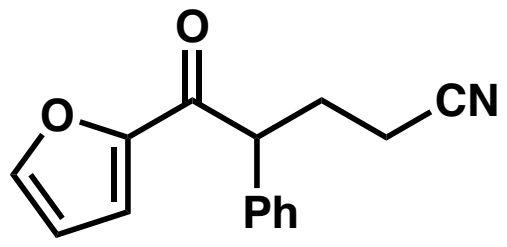

S16

5-(Furan-2-yl)-5-oxo-4-phenylpentanenitrile was synthesized on a 4.0 mmol scale from **S15** following the general procedure for the Michael addition to acrylonitrile. The crude product was purified by column chromatography, (pentane/diethyl ether, 4:1, R_f = 0.15). The pure product was obtained as yellow oil in 35% yield (0.34 g, 1.4 mmol). ^{1}H-NMR (400 MHz, $CDCl_3$): δ = 2.11-2.31 (m, 2H), 2.31-2.54 (m, 2H), 4.53 (t, J = 7.2 Hz, 1H), 6.43-6.49 (m, 1H), 7.15-7.21 (m, 1H), 7.23-7.29 (m, 1H), 7.30-7.36 (m, 4H), 7.49-7.56 (m, 1H). ^{13}C-NMR (101 MHz, $CDCl_3$): δ = 15.23, 28.16, 52.13, 112.54, 118.71, 119.24, 128.00, 128.33, 129.31, 137.17, 146.95, 151.97, 187.12. HRMS (pos. APCI): calcd for $C_{15}H_{13}NO_2$ $[M+H]^+$: 240.1019, found: 240.1025. MS (EI): m/z (%) = 239.1 $[M]^+$, 211.1, 186.1, 157.1, 144.1, 115.1, 104.1, 95.0, 91.0, 78.1, 51.0. IR (ATR): ν [cm^{-1}] = 3132, 3062, 3030, 2939, 2245, 1668, 1600, 1583, 1565, 1463, 1424, 1343, 1391, 1343, 1275, 1159, 1029, 1014, 882, 865, 842, 764, 737, 700, 591, 548, 505.

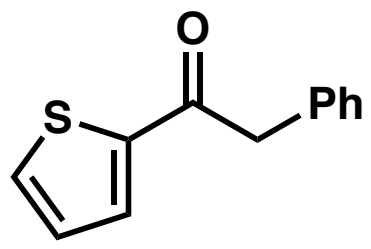

S17

2-Phenyl-1-(thiophen-2-yl)ethan-1-one was synthesized on a 10.0 mmol scale according to a literature procedure.[19] The crude product was dissolved in CH_2Cl_2 and washed twice with aq. NaOH (0.5 M). The combined organic phases were dried over Na_2SO_4 filtered and the solvent was evaporated. The pure product was obtained as yellow solid in 78% yield (1.58 g, 7.81 mmol). The NMR data matches the literature values.[20] ^{1}H-NMR (400 MHz, $CDCl_3$): δ = 4.20 (s, 2H), 7.12 (t, J = 4.5 Hz, 1H), 7.22-7.39 (m, 6H), 7.63 (d, J = 4.8 Hz, 1H), 7.77 (d, J = 3.8 Hz, 1H). ^{13}C-NMR (101 MHz, $CDCl_3$): δ = 46.54, 127.19, 128.30, 128.84, 129.53, 132.77, 134.16, 134.47, 144.04, 190.57.

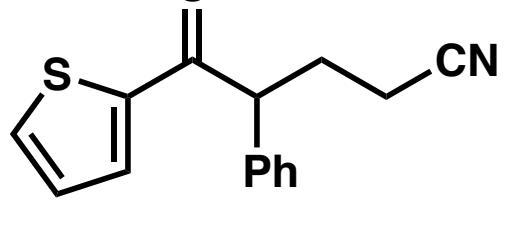

S18

5-Oxo-4-phenyl-5-(thiophen-2-yl)pentanenitrile was synthesized on a 3.0 mmol scale from **S17** following the general procedure for the Michael addition to acrylonitrile. The crude product was purified by column chromatography (CH_2Cl_2, R_f = 0.5). The pure product was obtained as yellow solid in 65% yield (0.501 g, 1.96 mmol). ^{1}H-NMR (400 MHz, $CDCl_3$): δ = 2.12-2.31 (m, 2H), 2.34-

2.53 (m, 2H), 4.56 (t, *J* = 7.2 Hz, 1H), 7.05 (t, *J* = 4.4 Hz, 1H), 7.23-7.38 (m, 5H), 7.59 (d, *J* = 5.3 Hz, 1H), 7.71 (d, *J* = 3.8 Hz, 1H).^{13}C-NMR (101 MHz, $CDCl_3$): δ = 15.30, 28.90, 53.42, 119.29, 128.10, 128.19, 128.38, 129.50, 133.16, 134.45, 137.71, 143.32, 191.17.HRMS (pos. APCI): calcd for $C_{15}H_{14}NOS$ $[M+H]^-$: 256.0791, found: 256.0797. MS (CI): m/z (%) = 273.1 $[M+NH_4]^+$, 256.1, 228.1, 203.1, 172.1, 128.0, 111.0, 91.1. IR (ATR): ν [cm^{-1}] = 3092, 3028, 2936, 2245, 1655, 1599, 1515, 1491, 1453, 1353, 1263, 1238, 1202, 1179, 1054, 912, 857, 798, 786, 764, 726, 699, 658, 583, 512.

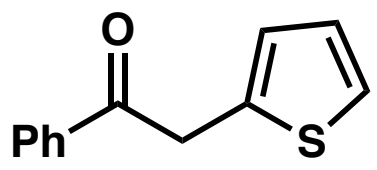

S19

1-Phenyl-2-(thiophen-2-yl)ethan-1-one was synthesized on a 8.0 mmol scale after a literature procedure.[21] The crude product was purified by recrystallization in methanol/pentane, 1:50. The pure product was obtained as colorless crystals in 71% yield (1.15 g, 5.69 mmol). The NMR data matched the literature values. ^{1}H-NMR (400 MHz, $CDCl_3$): δ = 4.50 (s, 2H), 6.89-7.02 (m, 2H), 7.19-7.29 (m, 1H), 7.44-7.52 (m, 2H), 7.55-7.63 (m, 1H), 7.98-8.08 (m, 2H).^{13}C-NMR (101 MHz, $CDCl_3$): δ = 39.53, 125.22, 126.94, 127.03, 128.71, 128.85, 133.52, 135.66, 136.25, 196.09.

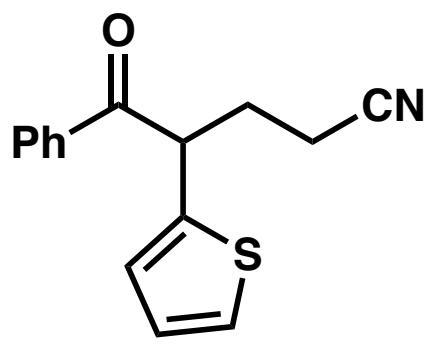

S20

5-Oxo-5-phenyl-4-(thiophen-2-yl)pentanenitrile was synthesized on a 3.0 mmol scale from **S19** following the general procedure for the Michael addition to acrylonitrile. The crude product was purified by column chromatography, (pentane/diethyl ether, 4:1 R_f = 0.15). The pure product was obtained as yellow oil in 47% yield (0.36 g, 1.4 mmol). ^{1}H-NMR (400 MHz, $CDCl_3$): δ = 2.21-2.39 (m, 2H), 2.39-2.53 (m, 2H), 5.03-5.09 (m, 1H), 6.89-7.00 (m, 2H), 7.19-7.25 (m, 1H), 7.40-7.48 (m, 2H), 7.51-7.59 (m, 1H), 7.94-8.05 (m, 2H). ^{13}C-NMR (101 MHz, $CDCl_3$): δ = 15.25, 29.77, 46.45, 77.16, 119.17, 125.98, 126.77, 127.49, 128.91, 128.96, 133.75, 135.67, 139.45, 197.18. HRMS (pos. APCI): calcd for $C_{15}H_{13}NOS$ $[M+H]^+$: 256.0791, found: 256.0796. MS (EI): m/z (%) = 255.1 $[M]^+$, 215.1, 150.1, 123.0, 110.0, 106.0, 105.0, 77.1. IR (ATR): ν [cm^{-1}] = 3069, 2940, 2245, 1736, 1680, 1596, 1579, 1529, 1447, 1356, 1322, 1283, 1237, 1179, 1076, 1041, 1001, 959, 851, 828, 793, 704, 688, 611, 554, 513.

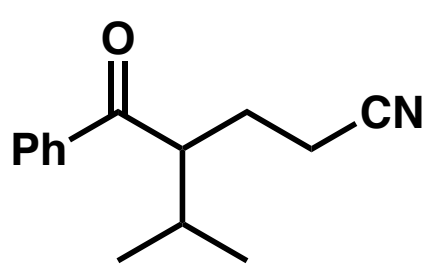

S21

4-Benzoyl-5-methylhexanenitrile. Synthesized from isovalerophenone according to the general procedure for acrylonitrile couplings. The crude product was purified using column chromatography (pentane/diethylether, 4:1 R_f = 0.25). The pure product was obtained as a clear oil in 93% yield (1.23 g, 5.73 mmol).^{1}H-NMR (400 MHz, $CDCl_3$): δ = 0.86 (d, *J* = 6.8 Hz, 3H), 1.00 (d, *J* = 6.8 Hz, 3H), 1.78-1.91 (m, 1H), 2.02-2.30 (m, 3H), 2.35-2.48 (m, 1H), 3.45-3.57 (m, 1H), 7.49 (t, *J* = 7.3 Hz, 2H), 7.58 (t, *J* = 6.4 Hz, 1H), 7.96 (d, *J* = 7.6 Hz, 2H). ^{13}C-NMR (101 MHz, $CDCl_3$): δ = 15.74, 18.71, 21.25, 23.07, 30.75, 50.85, 119.65, 128.41, 129.00, 133.55, 137.50, 202.70. HRMS (pos. ESI): calcd for $C_{14}H_{18}ON$ $[M+H]^+$: 216.1383, found: 216.1383. MS (CI): m/z (%) = 233.1 $[M+NH_4]^+$, 218.1, 216.1,

173.1, 105.1, 94.1, 77.0. IR (ATR): ν [cm^{-1}] = 3061, 2962, 2936, 2874, 2244, 1674, 1596, 1578, 1447, 1390, 1264, 1217, 1138, 1001, 972, 867, 846, 695, 587.

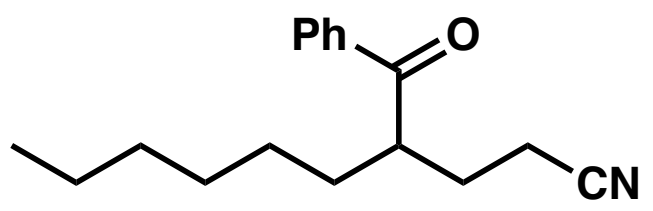

S22

4-Benzoyldecanenitrile was synthesized on a 3.0 mmol scale from commercially available octanophenone following the general procedure for the Michael addition to acrylonitrile. The crude product was purified by column chromatography, [pentane/ diethyl ether, 4:1 to 3:1, R_f(pentane/diethyl ether, 3:1) = 0.3]. The pure product was obtained as clear oil in 79% yield (0.610 g, 2.37 mmol). ^{1}H-NMR (400 MHz, $CDCl_3$): δ = 0.84 (t, *J* = 6.8 Hz, 3H), 1.12-1.36 (m, 8H), 1.41-1.60 (m, 1H), 1.69-1.80 (m, 1H), 1.80-1.93 (m, 1H), 2.13-2.34 (m, 2H), 2.36-2.48 (m, 1H), 3.59-3.69 (m, 1H), 7.50 (t, *J* = 7.7 Hz, 2H), 7.59 (t, *J* = 7.7 Hz, 1H), 7.97 (d, *J* = 7.7 Hz, 2H).^{13}C-NMR (101 MHz, $CDCl_3$): δ = 14.12, 15.49, 22.63, 26.83, 27.09, 29.39, 31.64, 32.75, 44.65, 119.56, 128.41, 129.01, 133.61, 136.84, 202.77. HRMS (pos. APCI): calcd for $C_{17}H_{24}ON$ $[M+H]^+$: 258.1852, found: 258.1858. MS (EI): m/z (%) = 258.2 $[M]^+$, 228.2, 200.1, 173.1, 145.1, 133.1, 105.0, 91.1, 77.0, 55.1. IR (ATR): ν [cm^{-1}] = 2928, 2857, 2245, 1678, 1596, 1579, 1447, 1378, 1226, 1180, 1074, 1001, 975, 827, 785, 704, 687, 652, 562.

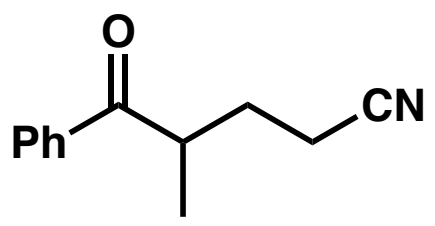

S23

4-methyl-5-oxo-5-phenylpentanenitrile was synthesized on a 11.7 mmol scale from propiophenone following the general procedure for the Michael addition to acrylonitrile. The crude product was purified by distillation (145 °C, 2×10^{-1} mbar) and subsequent column chromatography (petroleum ether/diethyl ether = 4:1, R_f = 0.4). The pure product was obtained as colorless oil in 49 % yield (0.98 g, 5.2 mmol). ^{1}H-NMR (400 MHz, $CDCl_3$): δ = 1.26 (d, *J* = 7.1 Hz, 3H), 1.75-1.86 (m, 1H), 2.17-2.28 (m, 1H), 2.31-2.50 (m, 2H), 3.61-3.74 (m, 1H), 7.46-7.53 (m, 2H), 7.56-7.63 (m, 1H), 7.98 (d, *J* = 7.9 Hz, 2H).^{13}C-NMR (101 MHz, $CDCl_3$): δ = 15.21, 17.92, 28.28, 39.25, 119.37, 128.36, 128.87, 133.47, 135.79, 202.42. HRMS (pos. ESI): calcd for $C_{12}H_{13}NO$ $[M+H]^+$: 188.1070 found: 188.1071 MS (EI, 70 eV): m/z (%) =187.1 $[M]^+$, 134.1 105.0, 102.0, 91.0, 77.0. IR (ATR): ν [cm^{-1}] = 3062, 2973, 2936, 2877, 2247, 1678, 1596, 1578, 1447, 1427, 1379, 1288, 1232, 1200, 1181, 1159, 1131, 1076, 1001, 976, 931, 793, 703, 687, 654, 557.

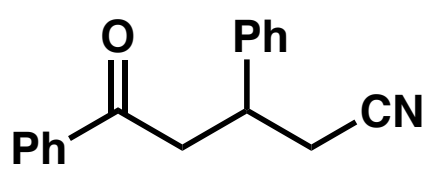

S24

5-Oxo-3,5-diphenylpentanenitrile. Literature-known compound that was synthesized on a 4 mmol scale from acetophenone and cinnamonitrile following the literature procedure.[22] The crude product was purified by column chromatography, [pentane/ethyl acetate, 5:1, R_f=0.35]. The pure product was obtained as yellow solid in 47% yield (469 mg, 1.88mmol). The NMR data matched the literature values. ^{1}H-NMR (400 MHz, $CDCl_3$): δ = 7.99–7.92 (m, 2H), 7.63-7.54 (m, 1H), 7.47 (t, J = 7.7 Hz, 2H), 7.41-7.24 (m, 3H), 3.76 (dq, J = 7.5, 6.0 Hz, 1H), 3.59-3.41 (m, 2H), 2.91-2.75 (m, 2H). ^{13}C-NMR (101 MHz, $CDCl_3$): δ = 197.35, 141.41, 136.53, 133.63, 129.11, 128.82, 128.10, 127.77, 127.26, 118.33, 42.86, 36.88, 24.39.

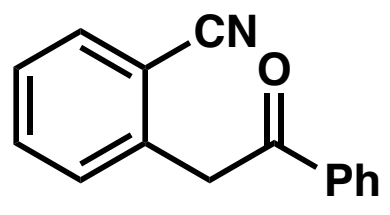

S25

2-(2-Oxo-2-phenylethyl)benzonitrile. Literature-known compound that was synthesized according to a modified version of the literature procedure.[23] In a flame-dried 100 mL Schlenk flask, sodium hydride (4.056 g, 0.101 mol, 4.000 eq) was added to 25 mL of dimethoxyethane under a nitrogen atmosphere and stirred at room temperature for 20 minutes. Subsequently, aryl ester methyl benzoate (3.2 mL, 25.352 mmol, 1.000 eq) and *o*-tolunitrile (3.1 mL, 25.352 mmol, 1.000 eq) were added dropwise via syringe, and the reaction mixture was stirred at 90 °C for 4 hours. Upon the complete consumption of the starting material (TLC control), the reaction was cooled to room temperature. The reaction was quenched slowly by the dropwise addition of 10 mL isopropanol. The mixture was then worked up with 9 mL brine and ethyl acetate (3 × 40 mL). The combined organic layers were dried over anhydrous Na_2SO_4, filtered, and concentrated under a rotary evaporator. The resulting residue was purified by column chromatography (petroleum ether/EtOAc = 10:1, R_f = 0.2), giving yellow crystals in a 64% yield. The analytical data matched the literature values. ^{1}H-NMR (400 MHz, $CDCl_3$): δ = 4.56 (s, 2H), 7.40 (t, *J* = 7.7 Hz, 2H), 7.51 (t, *J* = 7.4 Hz, 2H), 7.56-7.63 (m, 2H), 7.70 (d, *J* = 7.7 Hz, 1H), 8.06 (d, *J* = 7.7 Hz, 2H). ^{13}C-NMR (126 MHz, $CDCl_3$): δ = 43.73, 113.77, 118.07, 127.77, 128.55, 128.99, 131.19, 132.97, 133.85, 136.35, 138.66, 195.56. MS (CI): m/z (%) = 222 $[M+H]^+$ (100), 105 (30). HRMS (EI) calcd for $C_{15}H_{11}NO^+$ $[M+H]^+$: 222.0913, found: 222.0909.

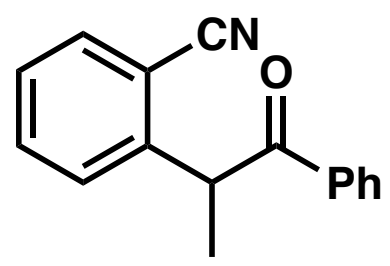

S26

2-(1-Oxo-1-phenylpropan-2-yl)benzonitrile. Literature-known compound that was synthesized from **S25** following a modification of the literature procedure.[24] In a flame-dried 50 mL Schlenk flask, a solution of 2-(2-oxo-2- phenylethyl)benzonitrile, 22, (1.106 g, 5.000 mmol, 1.000 eq) in 10 mL THF was added to a solution of KO*t*-Bu (0.617 g, 5.500 mmol, 1.100 eq) in 5 mL THF at 0 °C dropwise, forming a deep yellow solution. The resulting solution was stirred for 20 minutes, after which methyl iodide (0.311 mL, 5.000 mmol, 1.000 eq) was added. The mixture was stirred for 2 hours and warmed to room temperature. Subsequently, hydrochloric acid (1 M, 30 mL) and 10 mL of ethyl acetate were added. The organic layer was separated, and the aqueous layer was extracted with ethyl acetate (15 mL, 3 times). The combined organic layers were washed with saturated NaCl solution (15 mL) and dried with sodium sulfate. After filtering and removing the solvent in vacuo, the crude product was purified by automated flash column chromatography using a 25 g column (petroleum ether/EtOAc = 10:1, R_f = 0.4). The final product was obtained as a yellow solid in 68% yield. The analytical data matched the literature values. ^{1}H-NMR (400 MHz, $CDCl_3$): δ = 1.58 (d, *J* = 6.8 Hz, 3H), 5.17 (q, *J* = 6.8 Hz, 1H), 7.31 (t, *J* = 7.6 Hz, 1H), 7.37 (d, *J* = 7.9 Hz, 1H), 7.43 (t, *J* = 7.6 Hz, 2H), 7.48-7.54 (m, 2H), 7.67 (d, *J* = 7.7 Hz, 1H), 7.98 (d, *J* = 7.8 Hz, 2H). ^{13}C-NMR (126 MHz, $CDCl_3$): δ = 19.31, 45.42, 112.18, 118.05, 127.65, 128.17, 128.77, 128.93, 133.32, 133.52, 133.56, 135.94, 145.08, 199.13. ^{1}H-NMR (500 MHz, C_6D_6): δ = 1.37 (d, *J* = 6.8 Hz, 3H), 5.08 (q, *J* = 6.8 Hz, 1H), 6.47 (td, *J* = 1.0, 7.7 Hz, 1H), 6.71, (td, *J* = 1.3, 7.8 Hz, 1H), 6.94-7.00 (m, 4H), 7.12 (d, *J* = 8.0 Hz, 1H), 8.01-8.03 (m, 2H). ^{13}C-NMR (126 MHz, C_6D_6): δ = 19.13, 45.55, 112.61, 118.08, 127.27, 128.00, 128.82, 128.90, 133.04, 133.06, 133.17, 136.45, 145.37, 198.25. MS (CI): m/z (%) = 236 $[M+H]^+$ (100), 105 (27). HRMS (EI) calcd for $C_{16}H_{13}NO^+$ $[M+H]^+$: 236.1070, found: 236.1073.

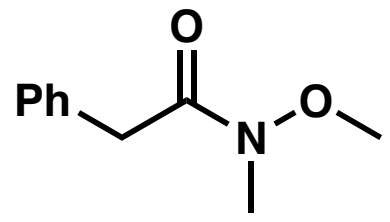

S27

***N*-methoxy-*N*-methyl-2-phenylacetamide.** Synthesized according to a literature procedure from phenacetyl chloride on a 30 mmol scale.[25] The Weinreb amide was received in 95% yield (5.10 g, 28.5 mmol). The NMR data matched the literature values.[25] ^{1}H-NMR (400 MHz, $CDCl_3$): δ = 3.19 (s, 3H), 3.60 (s, 3H), 3.77 (s, 2H), 7.22-7.34 (m, 5H). ^{13}C-NMR (101 MHz, $CDCl_3$): δ = 32.35, 39.52, 61.37, 126.86, 128.59,129.39, 135.06, 172.51.

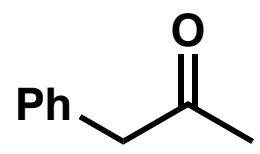

S28

1-Phenylpropan-2-one was synthesized from **S27** according to a literature procedure from *N*-methoxy-*N*-methyl-2-phenylacetamide (1.54 g, 8.59 mmol).[26] The pure product was obtained as a colorless oil in 87% yield (1.00 g, 7.45 mmol) and directly used in the next step. The NMR data matched the literature values.[27] ^{1}H-NMR (400 MHz, $CDCl_3$): δ = 2.15 (s, 3H), 3.70 (s, 2H), 7.18-7.23 (m, 2H), 7.25-7.30 (m, 1H), 7.31-7.37 (m, 2H).^{13}C-NMR (101 MHz, $CDCl_3$): δ = 29.40, 51.19, 127.21, 128.91, 129.53, 134.40, 206.51.

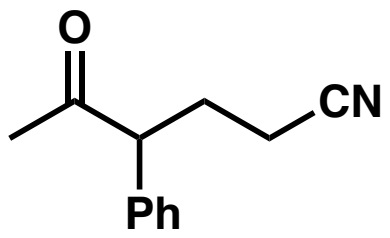

S29

5-Oxo-4-phenylhexanenitrile. A Schlenk tube was flame dried and a solution of 1-phenylpropan-2-one (**S28**) (0.100 mg, 0.745 mmol, 1.000 equiv) in 2.5 ml dry THF was added. Sodium hydride (60% in mineral oil) (29.8 mg, 0.745 mmol, 1.000 equiv) was added resulting in a color change from colorless to yellow. After 30 minutes of stirring, acrylonitrile (0.049 mL, 0.745 mmol, 1.000 equiv) was added dropwise, and the tube was sealed with a greased glass stopper. The reaction was stirred overnight at rt and was quenched by adding water (5 ml). The mixture was diluted with CH_2Cl_2 (50 ml) and water (40 ml). The organic phase was separated and the aq. phase was extracted CH_2Cl_2 (3 × 30 ml). The combined organic layers were dried over sodium sulphate and the solvent was removed. The crude product was purified using column chromatography [petroleum ether/diethyl ether, 3:1→2:1, $R_f(CH_2Cl_2)$ = 0.3]. The pure product was obtained as a colorless oil in 30% yield (42 mg, 0.224 mmol). The reaction was repeated on a 5.5 mmol scale to give 23% product (240 mg, 1.28 mmol). The NMR data matched the literature values.[28] ^{1}H-NMR (400 MHz, $CDCl_3$): δ = 1.92-2.02 (m, 1H), 2.05 (s, 3H), 2.08-2.21 (m, 1H), 2.26-2.37 (m, 2H), 3.79 (dd, *J* = 8.4, 6.2 Hz, 1H), 7.17-7.21 (m, 2H), 7.27-7.38 (m, 3H).^{13}C-NMR (101 MHz, $CDCl_3$): δ = 15.07, 27.28, 29.11, 57.62, 119.21, 128.13, 128.26, 129.49, 136.92, 206.63.

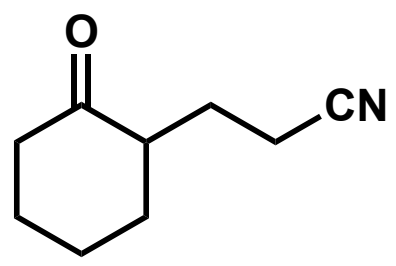

S30

3-(2-Oxocyclohexyl)propanenitrile. Synthesized on a 42.5 mmol scale according to a literature procedure.[29] The crude product was purified by distillation (165 °C, $p = 2\times10^{-1}$ mbar) and subsequent column chromatography (petroleum ether/diethyl ether, 4:1 R_f = 0.5). The pure product was obtained as colorless oil in 78 % yield (5.01 g, 33 mmol). The NMR data matched the literature values.[30] ^{1}H-NMR (400 MHz, $CDCl_3$): δ = 1.32-1.80 (m, 4H), 1.84-1.96 (m, 1H), 2.00-2.19 (m, 3H),

2.25-2.65 (m, 5H). ^{13}C-NMR (101 MHz, $CDCl_3$): δ = 15.40, 25.31, 25.74, 28.13, 34.44, 42.39, 49.00, 119.88, 211.92.

Iminonitriles

The iminonitriles were synthesized by condensation of the respective ketonitrile with a desired amine in presence of activated molecular sieves and *para*-toluenesulfonic acid following our previously published procedure.[31] The imine configuration was presumed to be *E* based on the usual thermodynamic preference of imine condensation reactions.

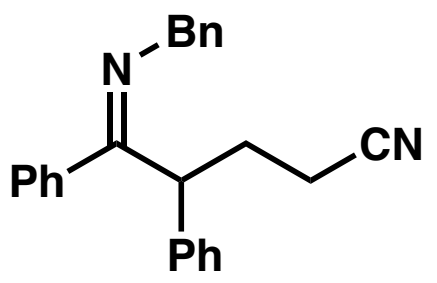

S31

(9% enamine content)

5-(Benzylimino)-4,5-diphenylpentanenitrile. Synthesized from **1** following our previously published procedure on a 0.75 mmol scale.[31] The reaction was heated to reflux until completion as determined by an NMR sample (C_6D_6, 72 h). The reaction was allowed to cool to room temperature, diluted with additional toluene and the molecular sieves were removed by filtration and rinsed several times with dichloromethane. The solvent was evaporated and the excess amine removed at a high-vacuum pump. The imine was received in 72.9% yield (185 mg, 0.547 mmol) and used without further purification. The material (presumably >99% *E*) contained ca. 5% of the enamine tautomer. ^{1}H-NMR (400 MHz, C_6D_6): δ = 1.61 (dt, *J* = 6.6, 16.0, Hz, 1H), 1.83 (tddd, *J* = 6.7, 7.1, 13.9 Hz, 1H), 1.93 (td, *J* = 6.6, 16.0 Hz, 1H,), 2.41 (tdd, *J* = 6.9, 7.4, 14.4 Hz, 1H), 3.82 (t, *J* = 7.5 Hz, 1H), 4.39 (d, *J* = 15.3 Hz, 1H), 4.42 (d, *J* = 15.3 Hz, 1H), 6.68-6.74 (m, 2H), 6.89-6.94 (m, 3H), 6.95-7.04 (m, 5H), 7.09-7.15 (m, 1H), 7.21-7.27 (m, 2H), 7.27-7.35 (m, 2H). ^{13}C-NMR (101 MHz, C_6D_6): δ = 15.15, 29.46, 55.17, 57.14, 119.60, 126.80, 126.98, 127.41, 127.95, 128.55, 128.71, 128.78, 128.95, 138.68, 139.97, 141.08, 171.36. IR (ATR): ν[cm^{-1}] = 3060, 3027, 2926, 2243, 1645, 1600, 1494, 1453, 1348, 1235, 1072, 1027, 761, 733, 698, 501.

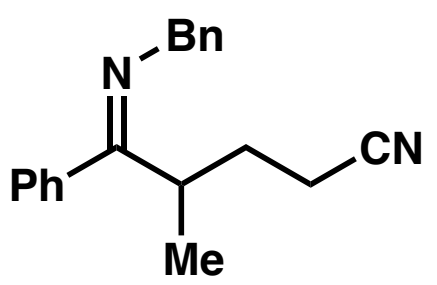

S32

(5% enamine content)

5-(Benzylimino)-4-methyl-5-phenylpentanenitrile. Synthesized from **S23** following our previously published procedure on a 0.5 mmol scale.[31] The reaction was heated to reflux until completion as determined by an NMR sample (C_6D_6, 72 h). The reaction was allowed to cool to room temperature, diluted with additional toluene and the molecular sieves were removed by filtration and rinsed several times with dichloromethane. The solvent was evaporated and the excess amine removed at a high-vacuum pump. The imine was received in >50:1 stereoselectivity (presumably *E* as major) in >99% yield (138 mg, 0.500 mmol). The material contained approx. 9% enamine tautomer and was used without further purification. ^{1}H-NMR (400 MHz, C_6D_6): δ = 0.83 (d, *J* = 7.0 Hz, 3H), 1.21-1.22 (m, 1H), 1.76 (dddd, *J* = 15.6, 6.9 , 4.9, 4.4 Hz, 1H), 1.87-1.96 (m, 1H), 1.97-2.07 (m, 1H), 2.59 (ddq, *J* = 7.0, 6.9 4.4 Hz, 1H), 4.35 (d, *J* = 15.0 Hz, 1H), 4.37 (d, *J* = 15.0 Hz, 1H), 6.84-6.92 (m, 2H), 7.01-7.12 (m, 4H), 7.14-7.19 (m, 1H), 7.21-7.27 (m, 3H). ^{13}C-NMR (101 MHz, C_6D_6): δ = 14.92, 18.41, 29.04, 42.87, 56.90, 119.84, 126.88, 126.91, 127.82, 128.49, 128.66, 128.78, 138.27, 141.20, 173.47. MS (EI, 70 eV): m/z (%) = 276 [M]$^+$ (<10), 223 (18), 91 (100). HRMS (pos. APCI): calcd for $C_{19}H_{20}N_2$ [M+H]$^+$ 277.1699, found 277.1696. IR (ATR): ν [cm^{-1}] = 3028, 2966, 2930, 2245, 1645, 1494, 1452, 1348, 1075, 1017, 770, 732, 703, 679.

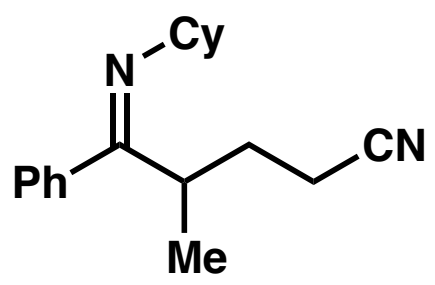

29

5-(Cyclohexylimino)-4-methyl-5-phenylpentanenitrile. To a 10 ml Schlenk tube equipped with PTFE stopcock and a magnetic stir bar, 4Å molecular sieves (637.0 mg) were added under an argon atmosphere. *para*-Toluenesulfonic acid monohydrate (10.2 mg, 0.053 mmol, 10 mol%) was added. The tube was evacuated and backfilled with argon. Then, dry toluene (3 ml), ketonitrile **S23** (100 mg, 0.534 mmol, 1.00 equiv) and cyclohexylamine (300.0 µl, 2.623 mmol, 4.91 equiv) were added. The reaction mixture was placed behind a blasting shield into a preheated heating block filled with sand (135 °C heating block temperature) and stirred. The reaction progress was monitored by letting the mixture cool down and drawing small samples which were analyzed by NMR-spectroscopy. After reaction completion (5 d) the mixture was allowed to cool to room temperature. It was filtered (celite/0.45 µm PTFE-Filter), rinsed with toluene (2 ml and 2 × 6 ml) and the solvent as well as the excess of cyclohexylamine were removed under high vacuum. The product was received in 95% yield (136.3 mg, 0.508 mmol) as a slightly yellow oil, which was stored in the freezer under argon atmosphere. The imine (presumably >98% *E*) contained traces of the enamine tautomers, the *other* imine stereoisomer, and a cyclized amidine byproduct arising from the addition of the enamine nitrogen to the nitrile. However, the material was found to be of suitable purity for the following cyclization. ^{1}H-NMR (400 MHz, C_6D_6): δ = 0.81 (d, *J* = 7.0 Hz, 3H), 0.90-1.01 (m, 1H), 1.02-1.19 (m, 2H,), 1.25 (dddd, *J* = 17.0, 7.1, 6.9, 4.4 Hz, 1H), 1.37-1.49 (m, 3H), 1.53-1.58 (m, 4H,), 1.81-1.90 (m, 1H), 1.94 (dddd, *J* = 13.3, 6.6, 5.8, 3.6 Hz, 1H), 2.14 (ddd, *J* = 16.1, 7.5, 7.5, 4.4 Hz, 1H,), 2.15 (dqd, *J* = 9.2, 7.0, 4.6 Hz, 1H), 3.16-3.22 (m, 1H), 6.90-6.97 (m, 2H), 7.00-7.05 (m, 1H), 7.06-7.11 (m, 2H). ^{13}C-NMR (101 MHz, C_6D_6): δ = 14.91, 18.54, 24.47, 24.50, 26.07, 28.98, 34.27, 34.50, 42.72, 60.46, 119.92, 126.87, 128.11, 128.66, 139.05, 169.90.

Titanium Catalyzed Cyclizations

Note: The *cis*/*trans* assignment is based on the CIP substituent priority:

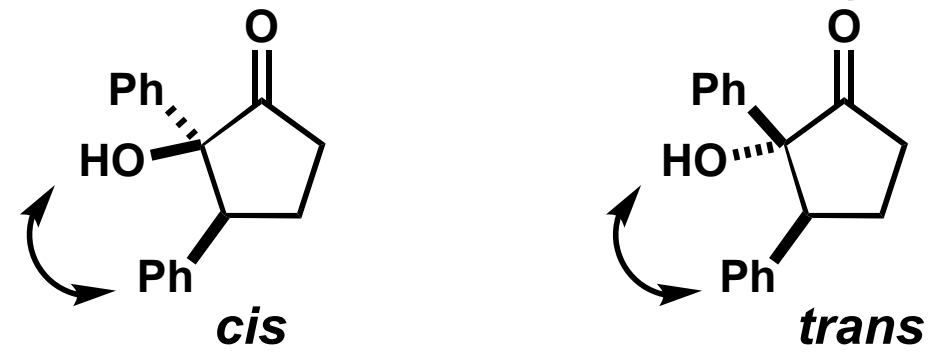

The determination of the diastereomeric ratio was usually possible by ^{1}H-NMR measured in C_6D_6 as solvent. Excerpts of the NMRs are appended to the data for the cyclization products.

Representative Procedure.

A flame-dried, argon-backfilled 10 ml Schlenk tube equipped with a magnetic stir bar was charged with morpholine hydrochloride (24.7 mg, 0.2 mmol, 1.00 equiv), Cp_2TiCl_2 (5.00 mg, 0.020 mmol, 10 mol%), and zinc dust (26.0 mg, 0.4 mmol, 2.0 equiv). The Schlenk tube was evacuated and backfilled with argon three times. THF (0.5 ml) was added to the tube and the solution was stirred for 10 min until the color turned from red to green. The desired ketonitrile (0.2 mmol, 1.0 eq) and TMSCl (51 µl, 0.400 mmol, 2.0 eq,) were added subsequently. The tube was closed with a Teflon cap, immersed into a preheated oil bath at 35°C, and then stirred for 24 h unless noted otherwise. The green-black reaction was quenched with 1 ml of methanol and the now red solution was transferred into a flask containing 2 M aq. HCl (50 ml). The suspension was stirred for 1 h before the pH was brought to 10–11 by addition of solid potassium carbonate. The solution was transferred into a separation funnel and was washed with ethyl acetate (4 × 40 ml). The combined organic layers were dried over sodium sulfate, filtered, and the solvent was removed under reduced pressure to give the crude product. The diastereomeric ratio was determined by ^{1}H-NMR and purification was carried out as described below.

Extended Screening Results

Table S1. Screening of different reaction conditions.

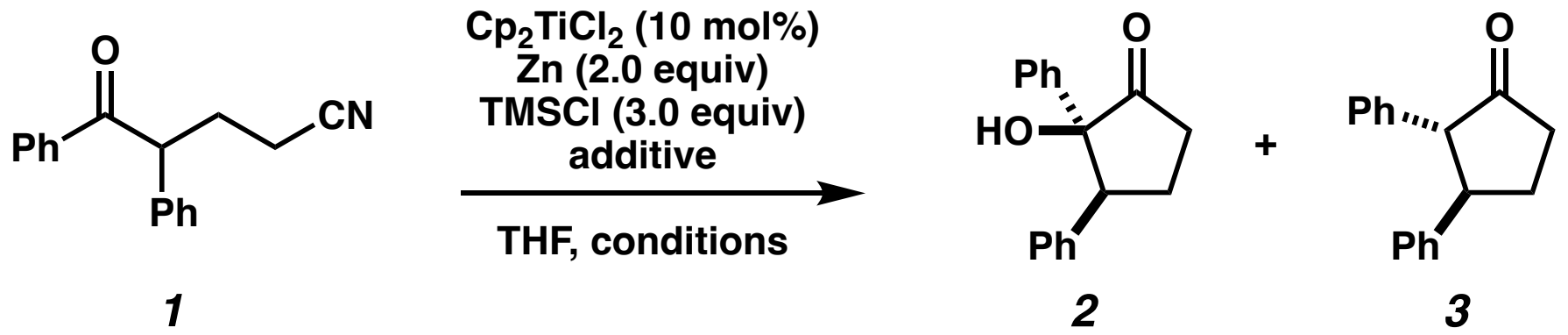

Entry	*T* / °C	Additive (equiv)	*t* / d	d.r. (2)[a]	Yield (2) / %[b]	Conversion (3)[a]
1	35	Et_3N•HCl (0.5)	2	6/1	88	4
2	35	Et_3N•HCl (0.5)	1	6/1	86	3
3	35	Coll•HCl (0.5)	1	15/1	73	8
4	35	Coll•HCl (1.0)	1	11/1	56	10
5	35	Coll•HCl (2.0)	1	14/1	68	14
6	35	Coll•HCl (0.25)	1	13/1	70	8
7	35	Coll•HCl (0.1)	1	7/1	49	4
8	35	Coll•HCl (0.5)	0.5	15/1	67	7
9	35	Coll•HCl (0.25)	0.5	6/1	56	5
10	30	Coll•HCl (0.5)	1	12/1	59	8
11	40	Coll•HCl (0.5)	1	10/1	74	8

[a] Determined by ^{1}H-NMR of the crude reaction mixture. [b] Yield of isolated product.

Table S2. Additional screening of hydrochlorides.

Entry	Additive (equiv)	*t* / d	d.r. (2)[a]	Yield (2) / %[b]	Conversion (3)[a]
1	DIPEA•HCl (0.5)	1	13/1	81	5
2	NH_4Cl (0.5)	1	10/1	58	6
3	Morph•HCl (0.5)	1	17/1	83	5
4	$NHMe_2$•HCl (0.5)	1	15/1	75	9
5	NH_2(*n*-Pr)•HCl (0.5)	1	8/1	61	4
6	OBnCin•HCl (0.5)	1	15/1	75	9
7	Morph•HCl (1.0)	1	18/1	85	6

[a] Determined by ^{1}H-NMR of the crude reaction mixture. [b] Yield of isolated product.

Table S3. Screening of other additives.

Entry	Additive (equiv)	*t* / d	d.r. (2)[a]	Yield (2) / %[b]	Conversion (3)[a]
1	$ZnCl_2$ (1.0)	1	--	51	--
2	Morpoline (1.0)	1	13/1	60	5
3	H_2O (0.5)	1	8/1	49	24
4	MS 4Å (0.5)	1	18/1	86	4

[a] Determined by ^{1}H-NMR of the crude reaction mixture. [b] Yield of isolated product.

Table S4. Screening of different reducing agents.

Entry	Reductant	Additive (equiv)	*t* / d	d.r. (2)[a]	Yield (2) / %[b]	Conversion (3)[a]
1	Zn	none	1	13/1	51	6
2	Mg	none	1	8/1	60	10
3	Mn	none	1	6/1	31	5
**4**	**Zn**	**Morph•HCl (1.0)**	**1**	**18/1**	**85**	**6**
5	Mn	Morph•HCl (1.0)	1	8/1	79	4
6	Mg	Morph•HCl (1.0)	1	9/1	61	14

[a] Determined by ^{1}H-NMR of the crude reaction mixture. [b] Yield of isolated product.

Table S5. Final optimization.

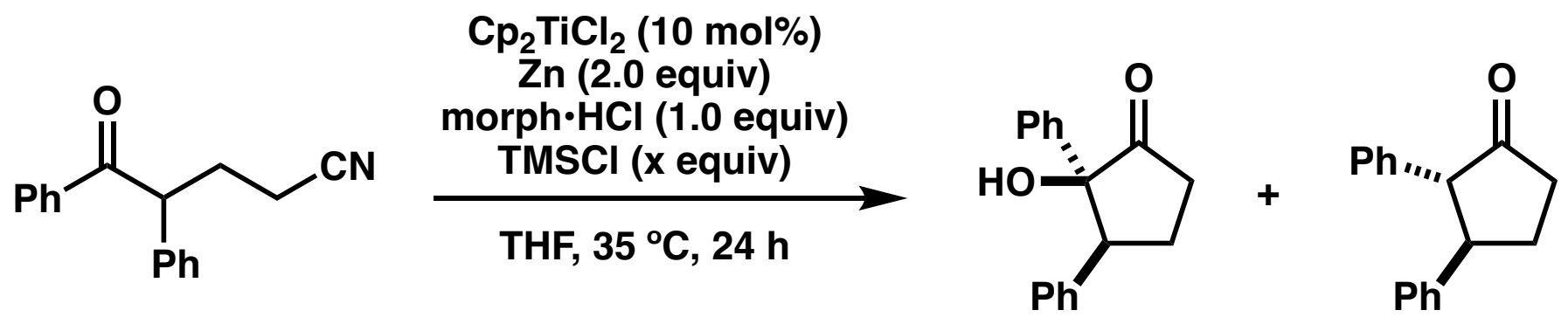

1 *2* *3*

Entry	*c* / mol L^{-1}	x	d.r. (2)[a]	Yield (2) / %[b]	Conversion (3)[a]
1	0.8	3	16/1	84	4
2	0.4	3	18/1	85	6
3	0.2	3	16/1	79	7
4	0.4	1	14/1	79	5
5	0.4	1.5	17/1	82	5
**6**	**0.4**	**2**	**22/1**	**84**	**5**

[a] Determined by ^{1}H-NMR of the crude reaction mixture. [b] Yield of isolated product.

Table S6. Comparison of titanium catalysts.

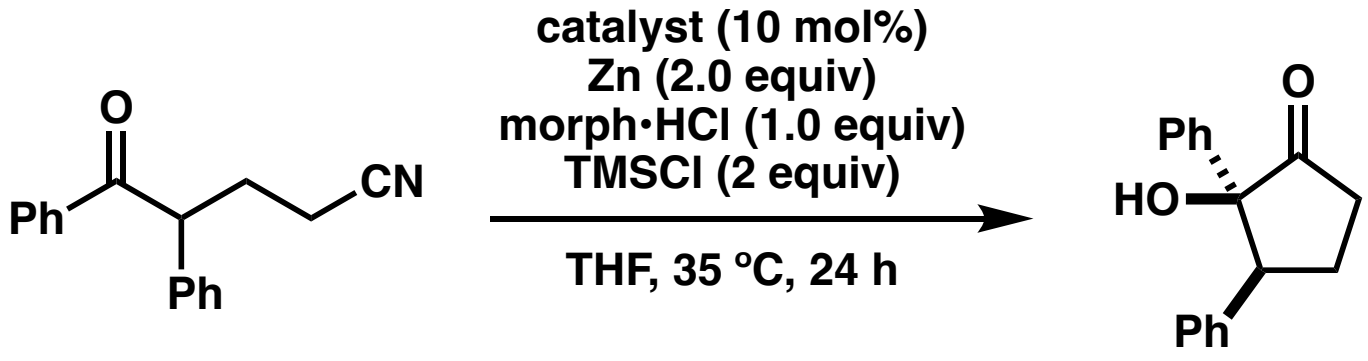

1 *2*

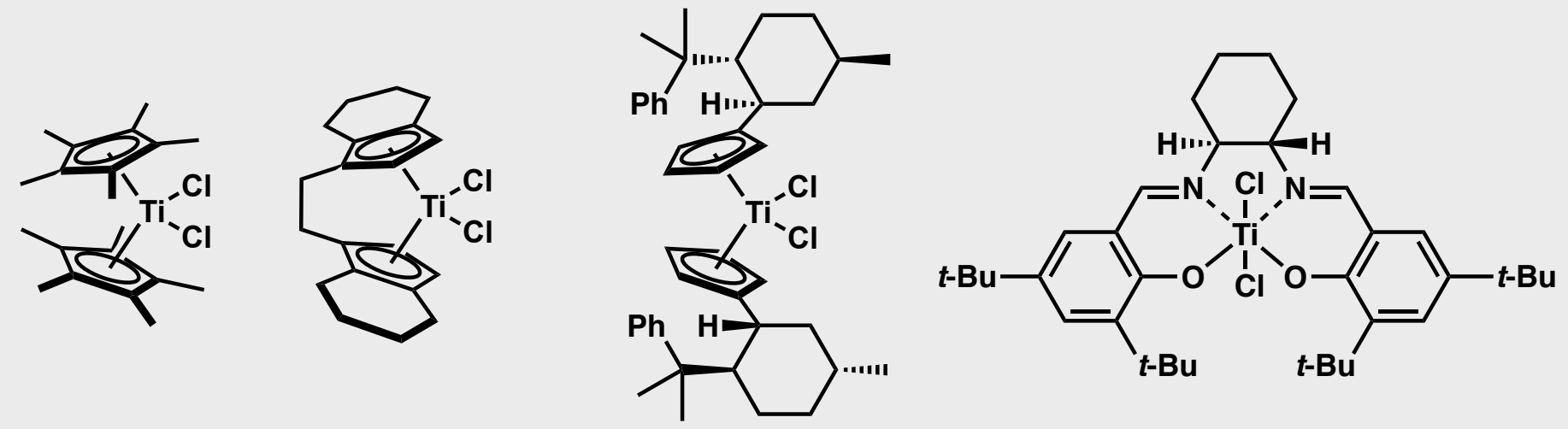

*Cp*₂TiCl₂* *(ebthi)TiCl₂* *(8-PhMenthCp)₂TiCl₂* (salen)TiCl₂

Entry	Catalyst	Conversion / %	d.r. (2)[a]
**1**	**Cp_2TiCl_2**	**95**	**21:1**
2	$Cp^*{}_2TiCl_2$	27	6:1
3	*rac*-(ebthi)$TiCl_2$	38	4:1
4	(8-PhMenthCp)$_2TiCl_2$	34	5:1
5	(*S*,*S*)-salen-$TiCl_2$	23	1:1

[a] Determined by ^{1}H-NMR of the crude reaction mixture.

Data for Hydroxyketone Products 2, 3, 5–22

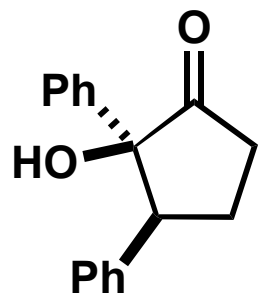

2

***cis*-2-Hydroxy-2,3-diphenylcyclopentan-1-one.** Synthesized following the representative procedure on a 0.2 mmol scale. The crude product was obtained as a 22:1 *cis*/*trans* mixture of diastereomers as determined by ^{1}H-NMR. Purification using column chromatography, [CH_2Cl_2→pentane/ethyl acetate 5:1, R_f(CH_2Cl_2) = 0.25] gave the product as a white solid in 84% yield (42 mg, 168 mmol) and 33:1 d.r. (^{1}H-NMR). A repetition of the experiment gave again a 22:1 *cis*/*trans* ratio (crude NMR) and the purified product in 90% yield (45 mg, 0.18 mmol, 40:1 d.r.) If desired, the pure *trans* diastereomer can be obtained by crystallization from ethanol.
R_f =0.3 (hexanes/EtOAc, 3:1). ^{1}H-NMR (400 MHz, C_6D_6): δ = 1.62-1.74 (m, 1H), 1.87-2.01 (m, 1H), 2.04 (s, 1H), 2.14-2.31 (m, 1H), 2.34-2.45 (m, 1H), 2.91-3.00 (m, 1H), 6.94-7.01 (m, 4H), 7.01-7.11 (m, 6H). ^{13}C-NMR (101 MHz, C_6D_6): δ = 24.90, 37.21, 56.14, 81.12, 126.67, 127.30, 127.62, 127.94, 128.10, 129.79, 137.25, 141.38, 214.43. ^{1}H-NMR (300.1 MHz, $CDCl_3$): δ = 2.25 (d, *J* = 0.9 Hz, 1H, OH), 2.28-2.38 (m, 1H), 2.54-2.68 (m, 2H), 2.80-2.95 (m, 1H), 3.55 (dd, *J* = 6.7, 11.1 Hz, 1H), 7.04-7.10 (m, 2H), 7.13-7.18 (m, 2H), 7.25-7.37 (m, 6H). ^{13}C-NMR (100.6 MHz, $CDCl_3$): δ = 24.5, 37.4, 55.5, 81.3, 126.1, 127.5, 127.9, 128.2, 128.4, 129.3, 136.6, 140.7, 215.9. HRMS (APCI) calcd for $C_{17}H_{20}NO_2^+$ $[M+NH_4]^+$: 270.14886, found: 270.14941. IR (ATR): ν [cm^{-1}] = 3452, 3060, 3028, 2952, 2886, 1739, 1268, 1448, 1403, 1327, 1276, 1139, 1090, 1062, 1025, 961, 923, 846, 828, 770, 752, 698, 667, 636, 620.

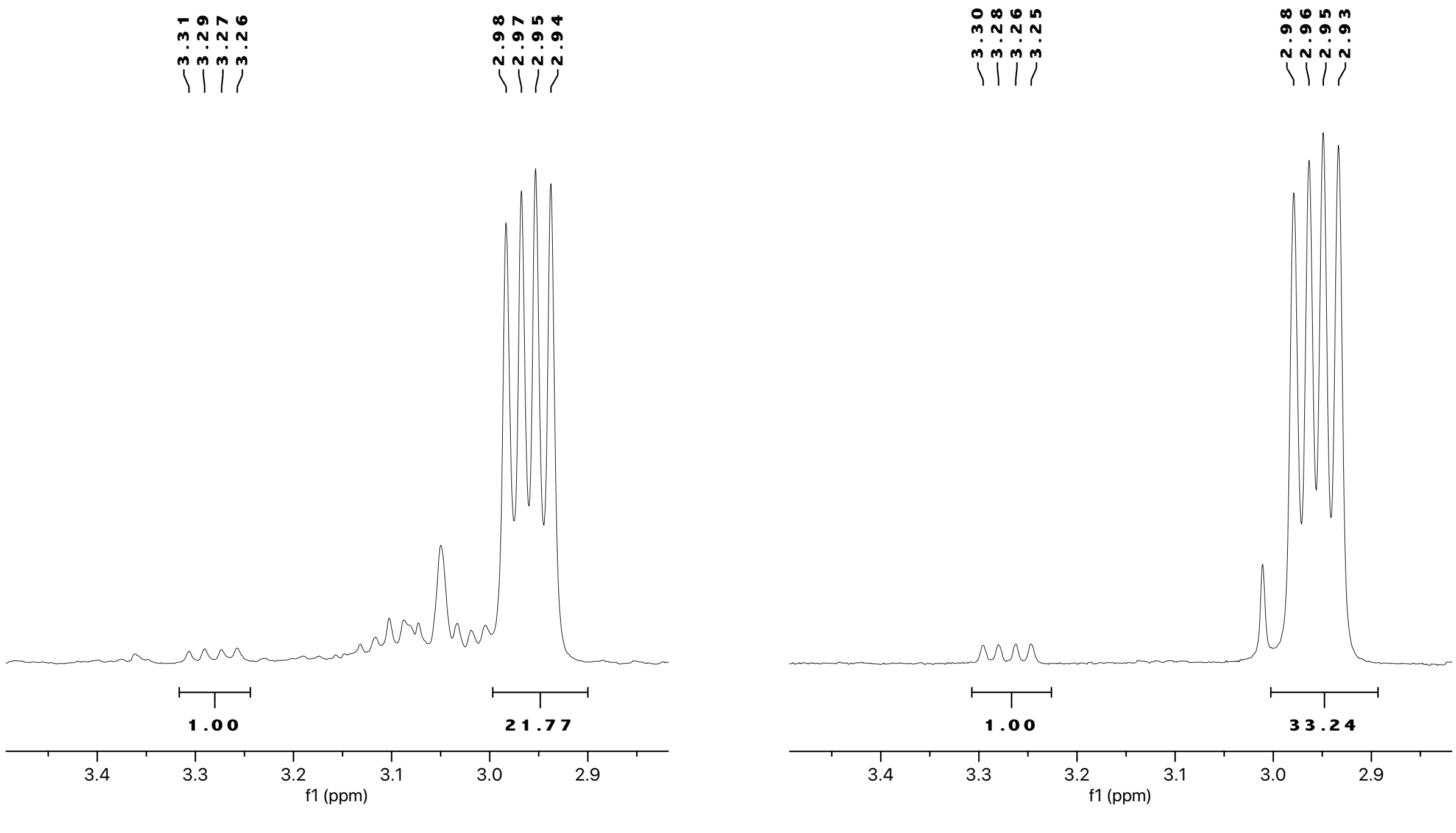

Figure S1. Excerpt of the ^{1}H-NMR (400 MHz, C_6D_6) spectrum of crude (left) and column-purified (right) **2** used for the determination of d.r.

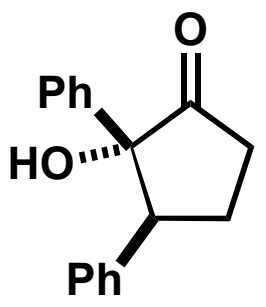

2'

***trans*-2-Hydroxy-2,3-diphenylcyclopentan-1-one**. The isolation and assignment of the *trans*-diasteromer was possible on a more enriched sample that could be obtained from an experiment with triethylamine hydrochloride as additive carried out on a 0.65 mmol scale. Here, a 5:1 *cis*/*trans* mixture was obtained. Purification using column chromatography, (petroleum ether/ethyl acetate 10:1, R_f = 0.03) gave the *trans* product as a white solid in 3% yield (4.3 mg, 0.017 mmol) and 12:1 d.r. (GC: 18:1 d.r.).
^{1}H-NMR (400 MHz, C_6D_6): δ = 1.50-1.61 (m, 1H), 1.68-1.84 (m, 1H), 1.98-2.11 (m, 1H), 2.12-2.24 (m, 1H), 2.99 (s, 1H), 3.27 (dd, *J* = 13.3, 6.4 Hz, 1H), 6.71-6.78 (m, 2H), 6.82-6.95 (m, 5H), 6.95-7.05 (m, 3H). ^{1}H-NMR (400 MHz, $CDCl_3$): δ = 2.17-2.32 (0xm, 2H), 2.61-2.87 (m, 2H), 3.11 (s, 1H), 3.51-3.61 (m, 1H), 6.74-6.81 (m, 2H), 6.89-6.98 (m, 2H), 7.07-7.22 (m, 6H).^{13}C-NMR (101 MHz, $CDCl_3$): δ = 21.80, 36.34, 53.83, 84.51, 126.81, 127.15, 127.82, 127.95, 128.01, 128.58, 137.85, 138.72, 220.20. HRMS (neg. ESI): calcd for $C_{17}H_{15}O_2$ $[M-H]^-$: 251.1078, found: 251.1080. MS (EI): m/z (%) = 252.1 $[M]^+$, 234.1, 196.1 178.1, 147.1, 118.1, 104.1, 91.1, 77.1, 65.1, 51.0 IR (ATR): ν [cm^{-1}] = 3428, 3087, 3062, 3030, 2953, 2920, 2887, 1744, 1602, 1582, 1496, 1447, 1401, 1375, 1280, 1213, 1179, 1133, 1062, 1042, 980, 966.

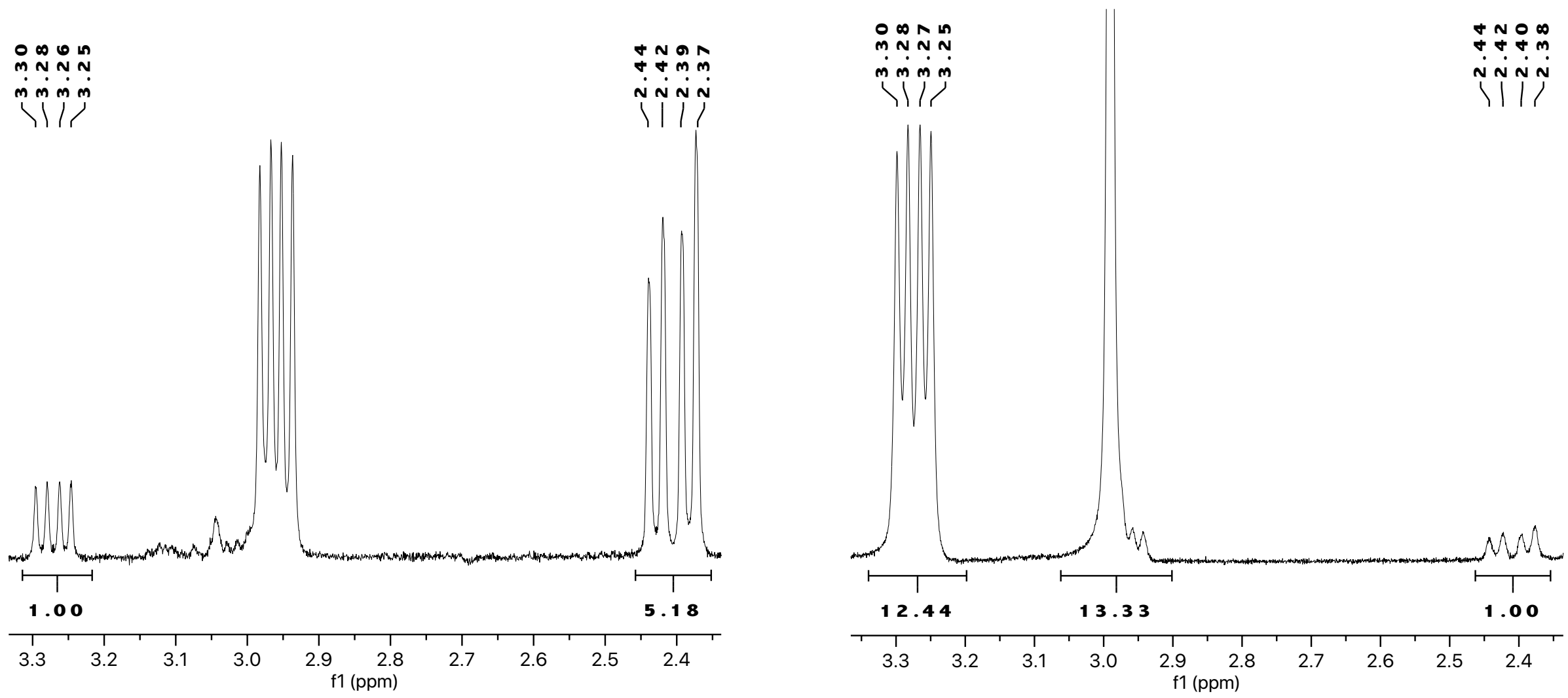

Figure S2. Excerpt of the ^{1}H-NMR (400 MHz, C_6D_6) spectrum of crude (left) and column-purified (right) **2'** used for the determination of the d.r.

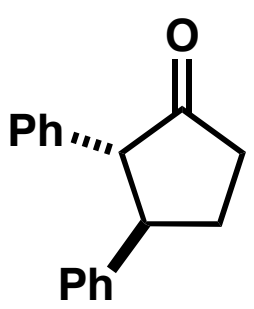

3

***trans*-2,3-Diphenylcyclopentan-1-one** was observed and isolated as a side product in the ketonitrile cyclization using 1.0 equivalent of water as an additive. The side product was purified by column chromatography, [pentane/ethyl acetate, 10:1, R_f(pentane/ethyl acetate, 2:1) = 0.4] and was further purified by recrystallization in ethanol. The pure side product was obtained as white crystals in 17% yield (8 mg, 0.034 mmol). The data and spectra matched the literature values for the *trans*-diastereomer.[32] ^{1}H-NMR (400 MHz, $CDCl_3$): δ = 2.01-2.14 (m, 1H), 2.41-2.51 (m, 2H), 2.68 (dd, *J* = 8.3, 16.9 Hz, 1H), 3.43 (d, *J* = 12.4 Hz, 1H), 3.50 (dt, *J* = 5.1, 11.9 Hz, 1H), 7.01 (d, *J* = 7.3 Hz, 2H), 7.14- 7.24 (m, 8H).

^{13}C-NMR (101 MHz, $CDCl_3$): δ = 29.67, 38.95, 50.58, 62.93, 127.04, 127.18, 127.22, 128.70, 128.77, 128.81, 137.21, 141.77, 216.67. ^{1}H-NMR (400 MHz, C_6D_6): δ = 1.41-1.57 (m, 1H), 1.75-1.99 (m, 2H), 2.13-2.26 (m, 1H), 2.95-3.12 (m, 2H), 6.82-6.92 (m, 2H), 6.94-7.12 (m, 8H). ^{13}C-NMR (101 MHz, C_6D_6): δ = 29.59, 38.62, 50.51, 62.41, 126.95, 127.03, 127.39, 128.60, 128.76, 129.16, 138.00, 142.37, 213.66. HRMS (pos. ESI): calcd for $C_{17}H_{16}$ $[M+H]^+$: 237.1274 found: 237.1275 MS (EI, 70 eV): m/z (%) =236.1 $[M]^+$, 179.1, 165.1, 152.1, 145.1, 118.1, 105.1, 91.1. IR (ATR): ν [cm^{-1}] = 3060, 3028, 2960, 1742, 1602, 1497, 1451, 1404, 1280, 1118, 1074, 909, 861, 727, 753, 697.

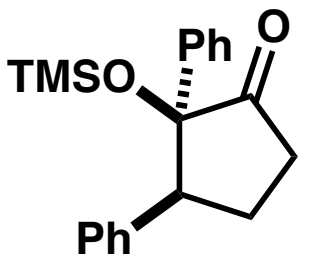

5

***cis*-2,3-Diphenyl-2-((trimethylsilyl)oxy)cyclopentan-1-one** was synthesized according to the general catalytic procedure, but was treated with a different workup. The reaction was quenched with 1 ml of water and two drops of glacial acid where added. The reddish mixture was stirred for 2 h at room temperature before DCM (15 mL) and water (10 ml) were added. The layers where separated and the aq. layer was washed with CH_2Cl_2 (3 × 10 mL). The combined organic layers were dried over sodium sulfate and the solvent was evaporated. The crude product was purified using column chromatography [petroleum ether/diethyl ether 50:1, R_f = 0.5]. The product (single diastereomer) was obtained as a clear oil in 80% yield (52 mg, 0.16 mmol).^{1}H-NMR (400 MHz, $CDCl_3$): δ = −0.01 (s, 9H), 2.13-2.26 (m, 1H), 2.43-2.58 (m, 2H), 2.72-2.86 (m, 1H), 3.08 (dd, *J* = 11.7, 6.2 Hz, 1H), 6.91– 6.92 (m, 2H), 6.97-7.04 (m, 2H), 7.16-7.27 (m, 6H). ^{13}C-NMR (101 MHz, $CDCl_3$): δ = 1.25, 25.01, 37.76, 58.92, 83.48, 126.66, 126.99, 127.27, 127.44, 127.60, 129.93, 137.24, 140.56, 215.95. HRMS (neg. ESI): calcd for $C_{20}H_{23}O_2Si^-$ $[M-H]^-$: 323,1473, found: 323,1473. MS (EI): m/z (%) = 324.2 $[M]^+$, 268.2, 253.1, 235.1, 205.1, 177.1, 152.1, 135.1, 115.1, 105.1, 73.1. IR (ATR): ν [cm^1] = 3087, 3061, 3028, 2955, 2895, 1745, 1602, 1496, 1454, 1404, 1250, 1209, 1144, 1096, 1072, 1042, 1029, 1009, 972, 887, 845, 754, 698, 638, 563.

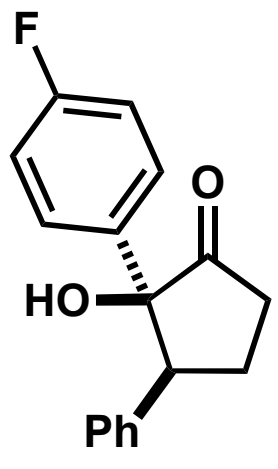

6

***cis*-2-(4-Fluorophenyl)-2-hydroxy-3-phenylcyclopentan-1-one** was synthesized according to the general procedure for the ketonitrile cyclization. The crude product was obtained as a 23:1 *cis*/*trans* mixture of diastereomers as determined by ^{1}H-NMR. Purification using column chromatography [CH_2Cl_2/ethyl acetate 200:1→100:1, R_f(CH_2Cl_2/ethyl acetate, 100:1) = 0.3]. gave the product as a white solid in 65% yield (35.0 mg, 0.129 mmol) and 31:1 d.r. (^{1}H-NMR). ^{1}H-NMR (400 MHz, C_6D_6): δ = 1.58-1.70 (m, 1H), 1.83-1.97 (m, 1H), 2.11 (s, 1H), 2.14-2.28 (m, 1H), 2.31-2.42 (m, 1H), 2.78 (dd, *J* = 12.2, 6.2 Hz, 1H), 6.67-6.78 (m, 4H), 6.89-6.97 (m, 2H), 7.02-7.11 (m, 3H). ^{1}H-NMR (400 MHz, $CDCl_3$): δ = 2.22-2.37 (m, 2H), 2.47-2.67 (m, 2H), 2.78-2.92 (m, 1H), 3.43 (dd, *J* = 11.0, 6.5 Hz, 1H), 6.96-7.13 (m, 6H), 7.23-7.30 (m, 3H).^{13}C-NMR (101 MHz, $CDCl_3$): δ = 24.41, 37.25, 55.74, 80.84, 115.25 (d, *J* = 21.5 Hz), 127.59, 127.96 (d, *J* = 8.3 Hz), 128.25, 129.29, 136.19, 136.31 (d, *J* = 3.1 Hz), 162.41 (d, *J* = 246.5 Hz), 215.78. ^{19}F NMR (376 MHz, $CDCl_3$) δ = -114.87 (ddd, *J* = 14.1, 8.2, 6.0 Hz). HRMS (neg. ESI): calcd for $C_{17}H_{14}O_2F$ [M-H$^+$]$^-$: 269.0983, found: 269.0984. MS (EI): m/z (%) = 270.1 [M]$^+$, 252.1, 214.1, 196.1, 165.1, 147.1, 123.0, 104.1, 95.0, 75.0. IR (ATR): ν [cm^{-1}] = 3450, 3069, 3030, 2951, 2886, 1739, 1602, 1508, 1455, 1404, 1354, 1264, 1223, 924, 817, 767, 718, 698, 597, 557.

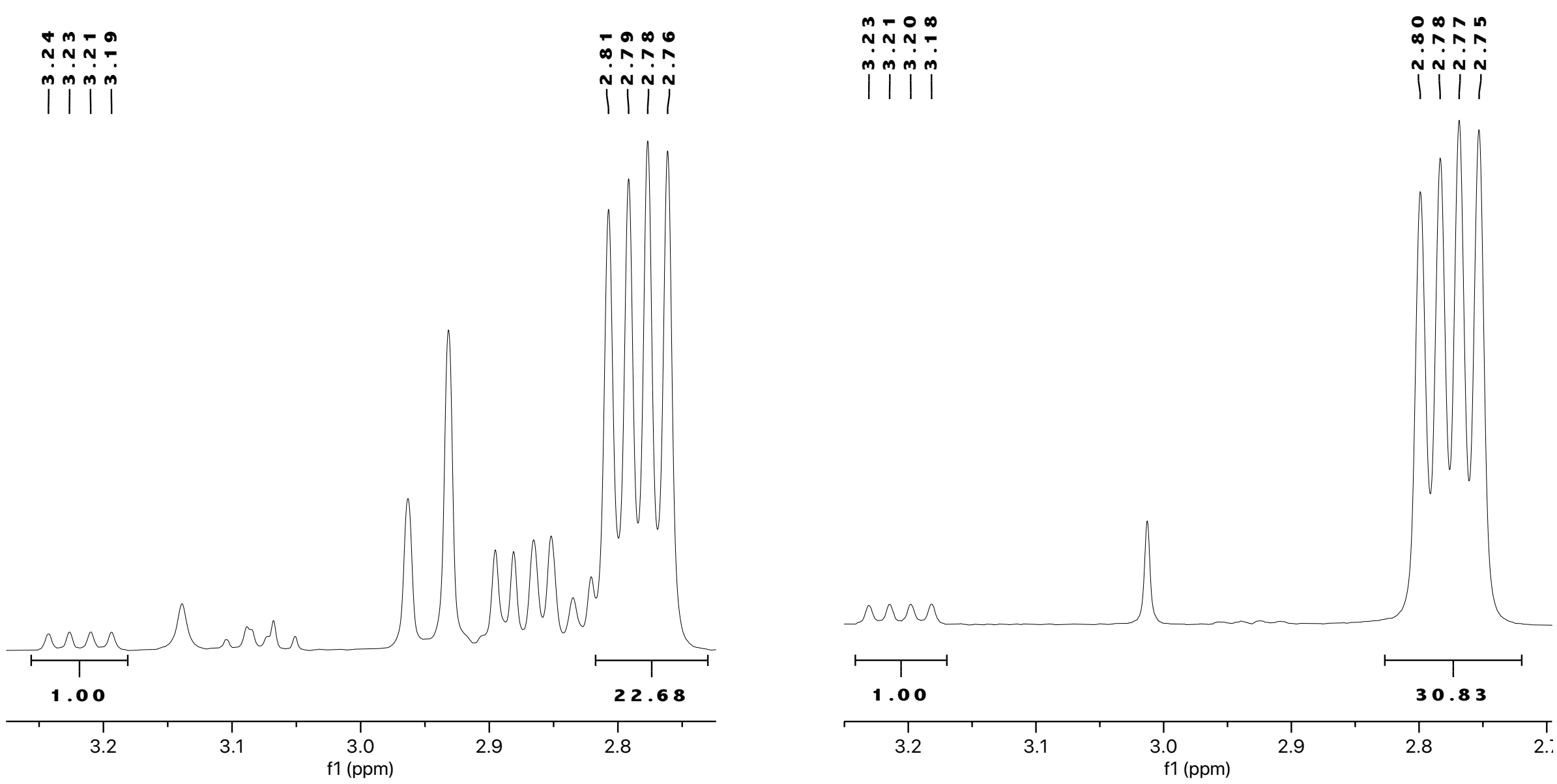

Figure S3. Determination of dr of **6** via ^{1}H-NMR, left crude mixture, right after purification.

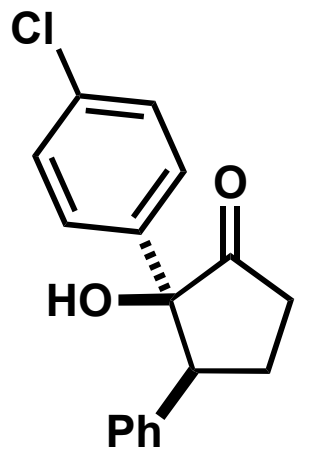

7

***cis*-2-(4-Chlorophenyl)-2-hydroxy-3-phenylcyclopentan-1-one** was synthesized according to the general procedure, for the ketonitrile cyclization. The crude product was obtained as a 14:1 *cis*/*trans* mixture of diastereomers as determined by ^{1}H-NMR. Purification using column chromatography [CH_2Cl_2/ethyl acetate 200:1→100:1, R_f(CH_2Cl_2/ethyl acetate 100:1) = 0.35] gave the product as a white solid in 83% yield (47.7 mg, 0.166 mmol) and 28:1 d.r. (^{1}H-NMR). ^{1}H-NMR (400 MHz, C_6D_6): δ = 1.55-1.69 (m, 1H), 1.80-1.96 (m, 2H), 2.12-2.27 (m, 1H), 2.30-2.43 (m, 1H), 2.76 (dd, *J* = 12.2, 6.3 Hz, 1H), 6.63-6.74 (m, 2H), 6.87-6.95 (m, 2H), 7.01-7.10 (m, 5H). ^{1}H-NMR (400 MHz, $CDCl_3$): δ = 2.22-2.37 (m, 2H), 2.50-2.67 (m, 2H), 2.79-2.93 (m, 1H), 3.44 (dd, *J* = 11.0, 6.5 Hz, 1H), 7.00-7.09 (m, 4H), 7.23-7.33 (m, 5H). ^{13}C-NMR (101 MHz, $CDCl_3$): δ = 24.43, 37.33, 55.70, 80.90, 127.64, 127.65, 128.30, 128.51, 129.27, 133.82, 136.02, 139.15, 215.48. HRMS (neg. ESI): calcd for $C_{17}H_{14}O_2Cl$ $[M-H]^-$: 285.0688, found: 285.0693. MS (EI): m/z (%) = 286.2 $[M]^+$, 268.1, 230.1, 205.1, 178.1, 165.1, 139.0, 104.1, 91.1, 77.0, 51.0. IR (ATR): ν [cm^{-1}] = 3446, 3030, 2951, 2885, 1738, 1599, 1491, 1454, 1398, 1374, 1352, 1282, 1212, 1140, 1091, 1065, 1014, 923, 826, 811, 735, 716, 697, 645, 621, 569, 515.

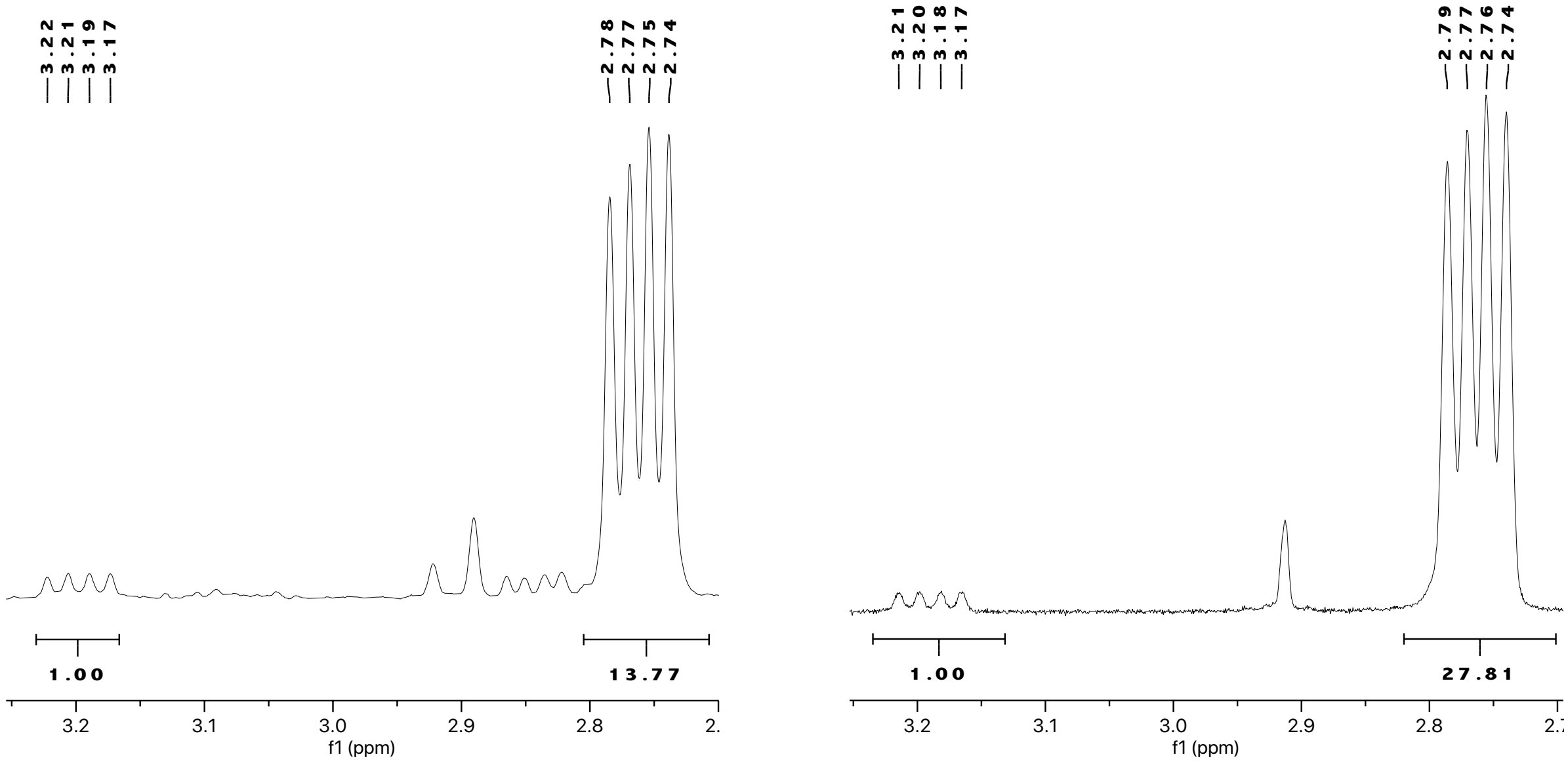

Figure S4. Determination of dr of **7** via ^{1}H-NMR, left crude mixture, right after purification.

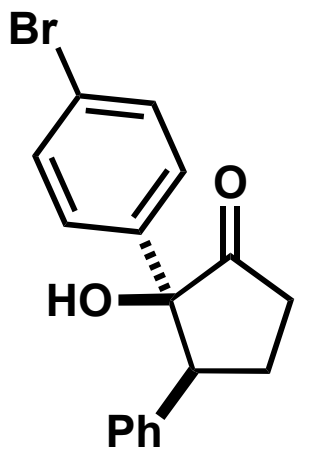

8

***cis*-2-(4-Bromophenyl)-2-hydroxy-3-phenylcyclopentan-1-one** was synthesized according to the general procedure, for the ketonitrile cyclization. The crude product was obtained as a 14:1 *cis*/*trans* mixture of diastereomers as determined by ^{1}H-NMR. Purification using column chromatography [CH_2Cl_2→CH_2Cl_2/ethyl acetate 100:1 R_f(CH_2Cl_2)= 0.3] gave the product as a white solid in 85% yield (56 mg, 0.169 mmol) and 25:1 d.r. (^{1}H-NMR). ^{1}H-NMR (400 MHz, c_6d_6) δ = 1.54-1.68 (m, 1H), 1.81-1.94 (m, 1H), 2.02 (s, 1H), 2.12-2.26 (m, 1H), 2.30-2.40 (m, 1H), 2.75 (dd, *J* = 12.3, 6.2 Hz, 1H), 6.60-6.66 (m, 2H), 6.87-6.94 (m, 2H), 7.05 (dt, *J* = 5.6, 2.0 Hz, 3H), 7.18-7.22 (m, 2H).^{1}H-NMR (400 MHz, $CDCl_3$): δ = 2.20-2.33 (m, 1H), 2.35 (s, 1H), 2.49-2.66 (m, 2H), 2.78-2.92 (m, 1H), 3.37-3.47 (m, 1H), 6.98 (d, *J* = 8.3 Hz, 2H), 7.01-7.08 (m, 2H), 7.22-7.30 (m, 3H), 7.43 (d, *J* = 8.8 Hz, 2H).^{13}C-NMR (101 MHz, $CDCl_3$): δ = 24.44, 37.34, 55.68, 80.94, 121.99, 127.64, 127.97, 128.29, 129.27, 131.42, 135.99, 139.69, 215.47. HRMS (neg ESI): calcd for $C_{17}H_{14}O_2Br$ [M-H]$^-$: 329.0183, found: 329.0187. MS (EI): m/z (%) = 330.0 [M]$^+$, 314.0, 274.0, 182.9, 165.1, 118.1, 104.1, 91.1 77.1. IR (ATR): ν [cm^{-1}] = 3440, 3065, 3029, 2950, 2884, 1736, 1597, 1486, 1455, 1395, 1351, 1281, 1211, 1067, 1011, 923, 826, 807, 768, 720, 695, 565, 509.

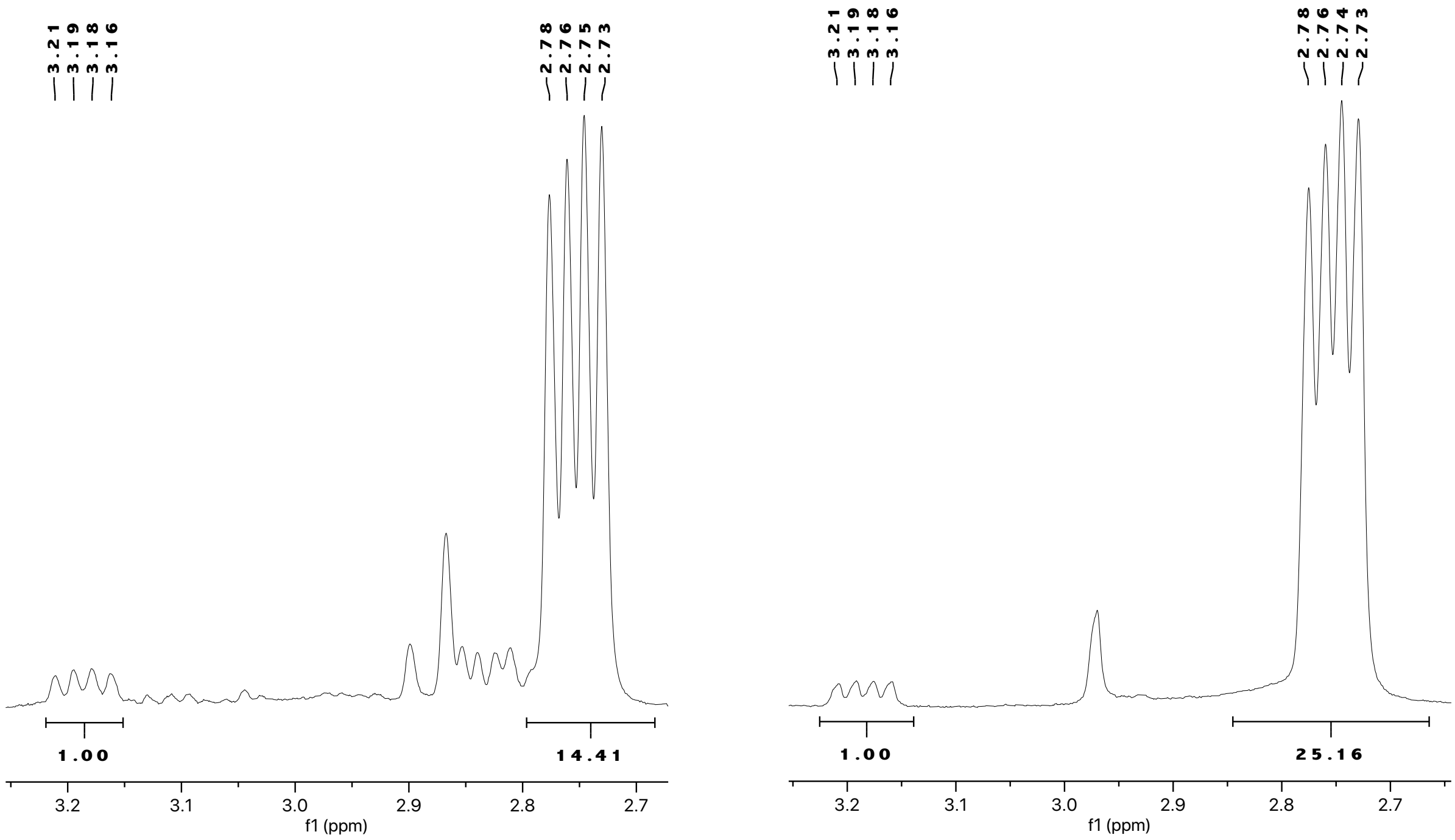

Figure S5. Determination of dr of **8** via ^{1}H-NMR, left crude mixture, right after purification.

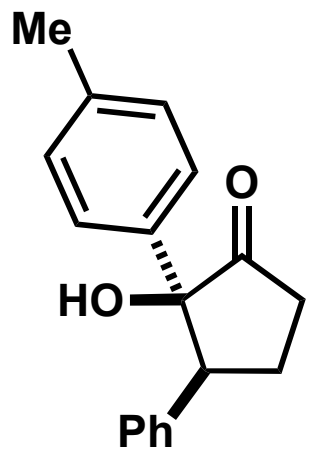

9

***cis*-2-Hydroxy-3-phenyl-2-(p-tolyl)cyclopentan-1-one** was synthesized according to the general procedure, for the ketonitrile cyclization. The crude product was obtained as a 30:1 *cis*/*trans* mixture of diastereomers as determined by ^{1}H-NMR. Purification using column chromatography [CH_2Cl_2/ethyl acetate pure→50:1, R_f(CH_2Cl_2) = 0.2] gave the product in as a white solid in 78% yield (41.5 mg, 0.156 mmol) and a 32:1 d.r. (^{1}H-NMR). ^{1}H-NMR (400 MHz, C_6D_6): δ = 1.64-1.74 (m, 1H), 1.90-2.03 (m, 1H), 2.09 (s, 3H), 2.19-2.31 (m, 2H), 2.35-2.46 (m, 1H), 2.99 (dd, *J* = 12.0, 6.2 Hz, 1H), 6.89-6.96 (m, 4H), 7.01-7.11 (m, 5H).^{13}C-NMR (101 MHz, C_6D_6): δ = 21.08, 24.91, 37.17, 56.10, 81.11, 126.65, 127.24, 128.89, 129.83, 137.04, 137.48, 138.48, 214.76, 214.79. HRMS (pos. ESI): calcd for $C_{18}H_{22}O_2N$ [M+NH_4]$^+$: 284.1645, found: 284.1649. MS (EI): m/z (%) = 266.2 [M]$^+$, 248.2, 238.1 223.1, 210.1, 191.1, 165.1 152.1, 134.1, 119.1 104.1 91.1 77.1 65.1 51.1. IR (ATR): ν [cm^{-1}] = 3457, 3028, 2950, 1740, 1603, 1513, 1498, 1454, 1404, 1276, 1212, 1139, 1064, 1035, 961, 921, 828, 802, 767, 700, 600, 506. 563.

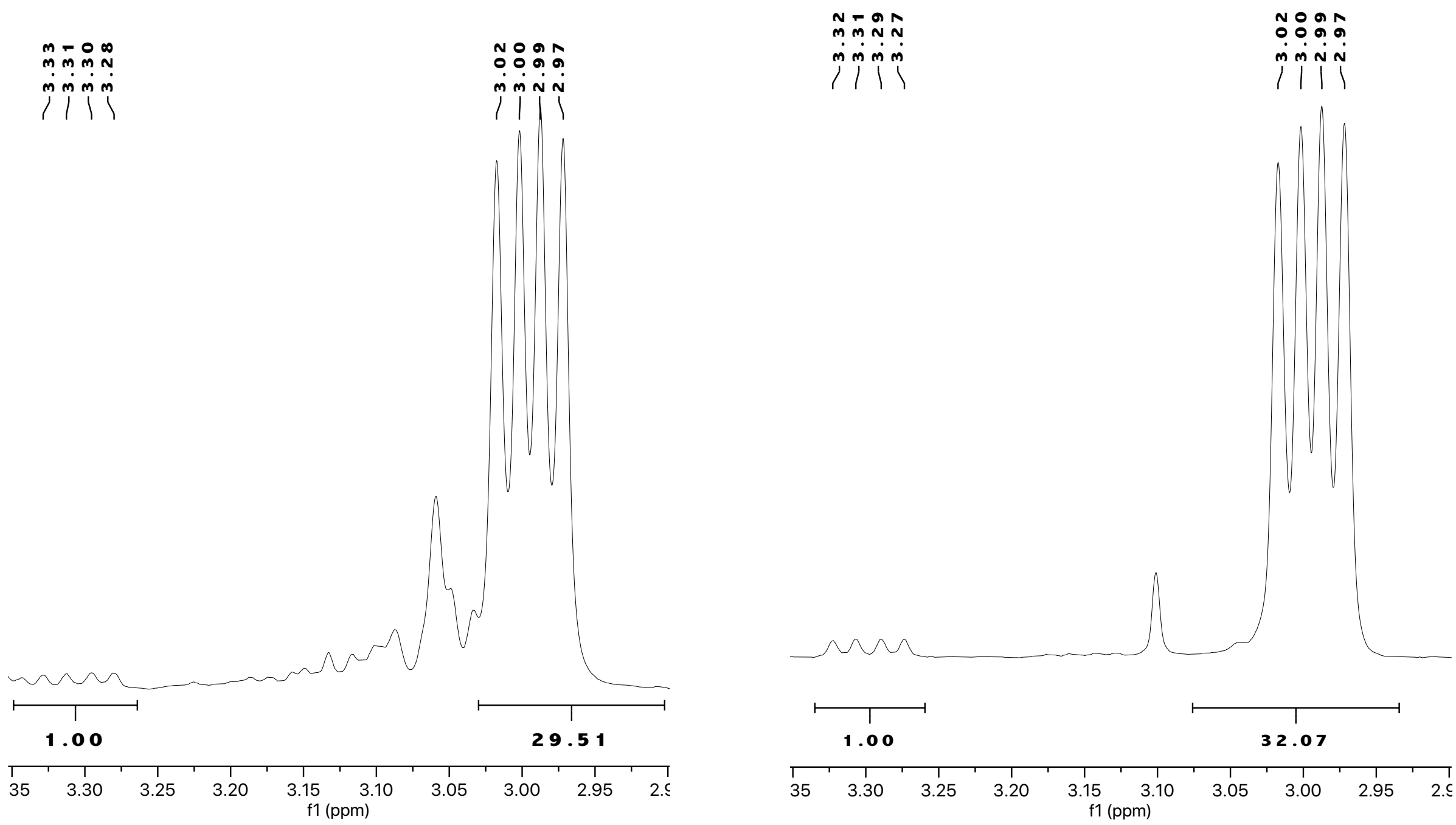

Figure S6. Determination of dr of **9** via ^{1}H-NMR, left crude mixture, right after purification.

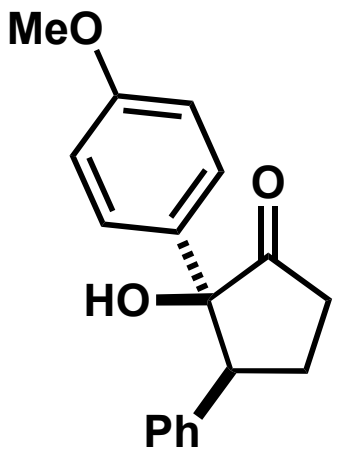

10

***cis*-(2-Hydroxy-2-(4-methoxyphenyl)-3-phenylcyclopentan-1-one** was synthesized according to the general procedure, for the ketonitrile cyclization and was stirred for 48h and at 45°C. The crude product was obtained as a 26:1 *cis*/*trans* mixture of diastereomers as determined by ^{1}H-NMR. Purification using column chromatography [CH_2Cl_2/methanol 1000:1→100:1, R_f(100:1) = 0.25] gave the product as a white solid in 76% yield (27.6 mg, 0.10 mmol) and 28:1 d.r. (^{1}H-NMR). ^{1}H-NMR (400 MHz, $CDCl_3$): δ = 2.19 (s, 1H), 2.22-2.34 (m, 1H), 2.49-2.63 (m, 2H), 2.76-2.90 (m, 1H), 3.45-3.53 (m, 1H), 3.80 (s, 3H), 6.85 (d, *J* = 8.8 Hz, 2H), 7.02-7.12 (m, 4H), 7.22-7.31 (m, 3H).^{13}C-NMR (101 MHz, $CDCl_3$): δ = 24.35, 37.09, 55.23, 55.39, 80.99, 113.82, 127.35, 127.41, 128.21, 129.28, 132.59, 136.80, 159.27, 216.23. HRMS (pos. ESI): calcd for $C_{18}H_{19}O_3$ $[M+H]^+$: 283.1329, found: 283.1332. MS (EI): m/z (%) = 282.2 $[M]^+$, 264.2, 226.1, 221.1, 205.1, 178.1, 165.1, 150.1 135.1 118.1 91.1 77.1 63.1. IR (ATR): ν [cm^{-1}] = 3438, 3030, 2953, 2837, 1738, 1610, 1583, 1511, 1455, 1346, 1299, 1246, 1178, 1030, 960, 910, 815, 767, 729, 699, 635, 563.

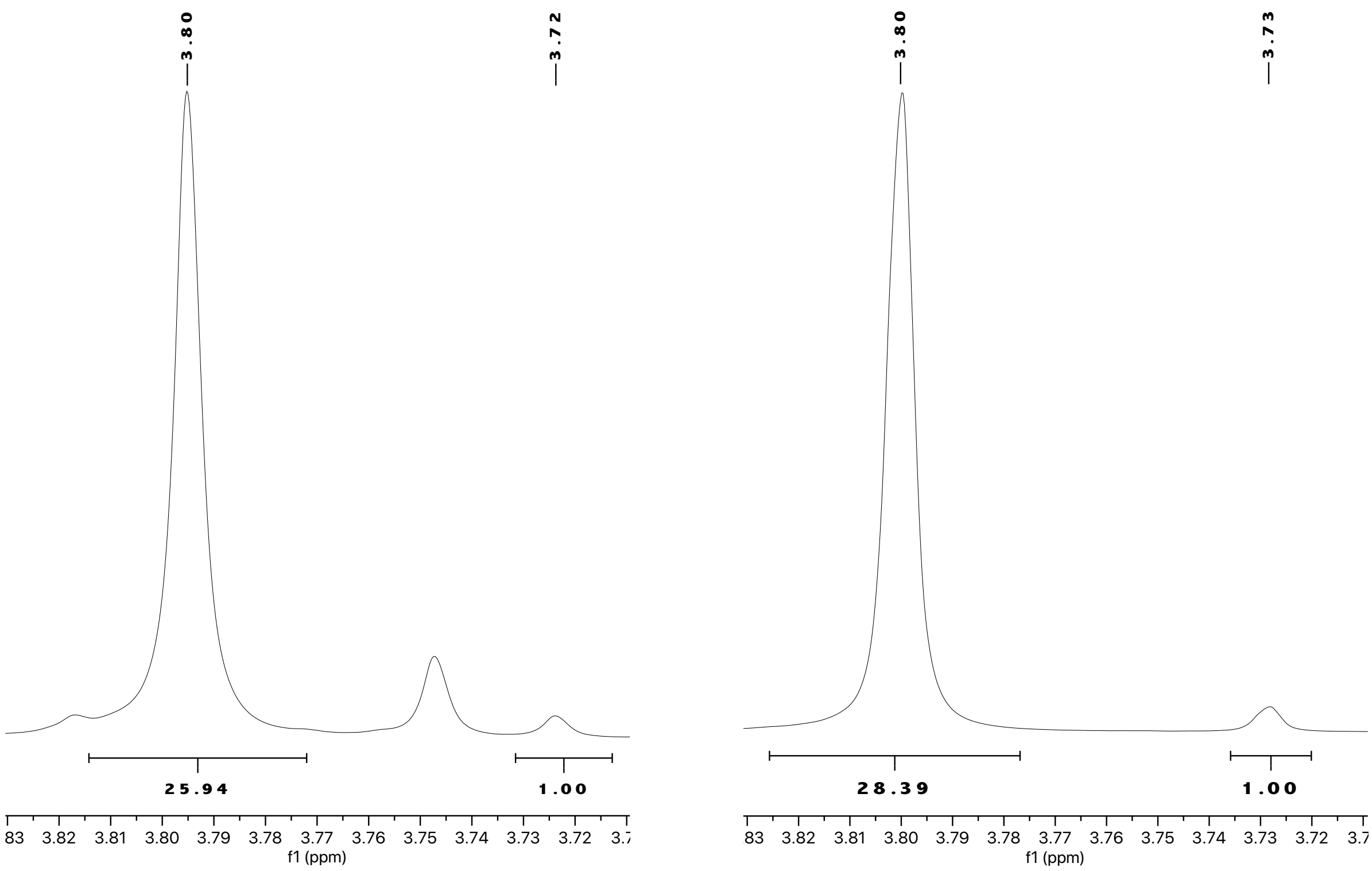

Figure S7. Determination of dr of **10** via ^{1}H-NMR, left crude mixture, right after purification.

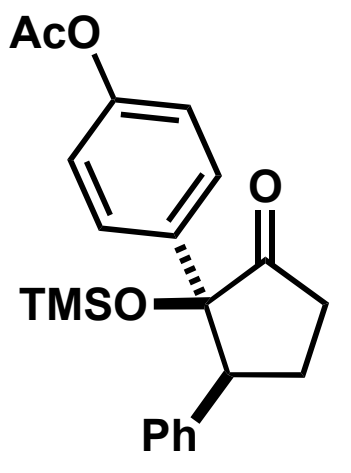

11

***cis*-2-Oxo-5-phenyl-1-((trimethylsilyl)oxy)cyclopentyl)phenyl acetate** was synthesized according to the general procedure, for the ketonitrile cyclization but was treated with a different workup. The reaction was quenched with 1 ml of water and two drops of glacial acid where added. The reddish mixture was stirred for 2h at room temperature before DCM (15 mL) and water (10 ml) were added. The layers where separated and the aq. layer was washed with CH_2Cl_2 (3 × 10 mL). The combined organic layers were dried over sodium sulfate and the solvent was evaporated. The crude product (*cis*/*trans* = ~17:1 d.r.) was purified using column chromatography [dichloromethane, R_f = 0.35]. The product was obtained as a clear oil in 47% yield (22 mg, 0.071 mmol). ^{1}H-NMR (400 MHz, C_6D_6) δ = 0.04 (s, 9H), 1.62-1.71 (m, 4H), 1.89-2.02 (m, 1H), 2.15-2.28 (m, 1H), 2.41 (dd, *J* = 19.3, 7.9 Hz, 1H), 2.66 (dd, *J* = 12.1, 6.1 Hz, 1H), 6.90-7.01 (m, 6H), 7.05-7.14 (m, 3H). ^{1}H-NMR (400 MHz, $CDCl_3$): δ = -0.04 (s, 9H), 2.08-2.25 (m, 1H), 2.27 (s, 3H), 2.41-2.57 (m, 2H), 2.69-2.84 (m, 1H), 3.03 (dd, *J* = 11.7, 6.3 Hz, 1H), 6.86-6.98 (m, 4H), 6.98-7.04 (m, 2H), 7.16-7.24 (m, 3H). ^{13}C-NMR (101 MHz, $CDCl_3$): δ = 1.24, 21.33, 25.00, 37.67, 58.84, 83.02, 120.63, 127.09, 127.53, 127.74, 129.99, 137.01, 138.02, 149.91, 169.26, 215.66. IR (ATR): ν [cm^{1}] = 3400, 3096, 3030, 2957, 1749, 1604, 1506, 1454, 1407, 1396, 1250, 1199, 1095, 1010, 973, 888, 843, 797, 777, 754, 698, 642, 555.

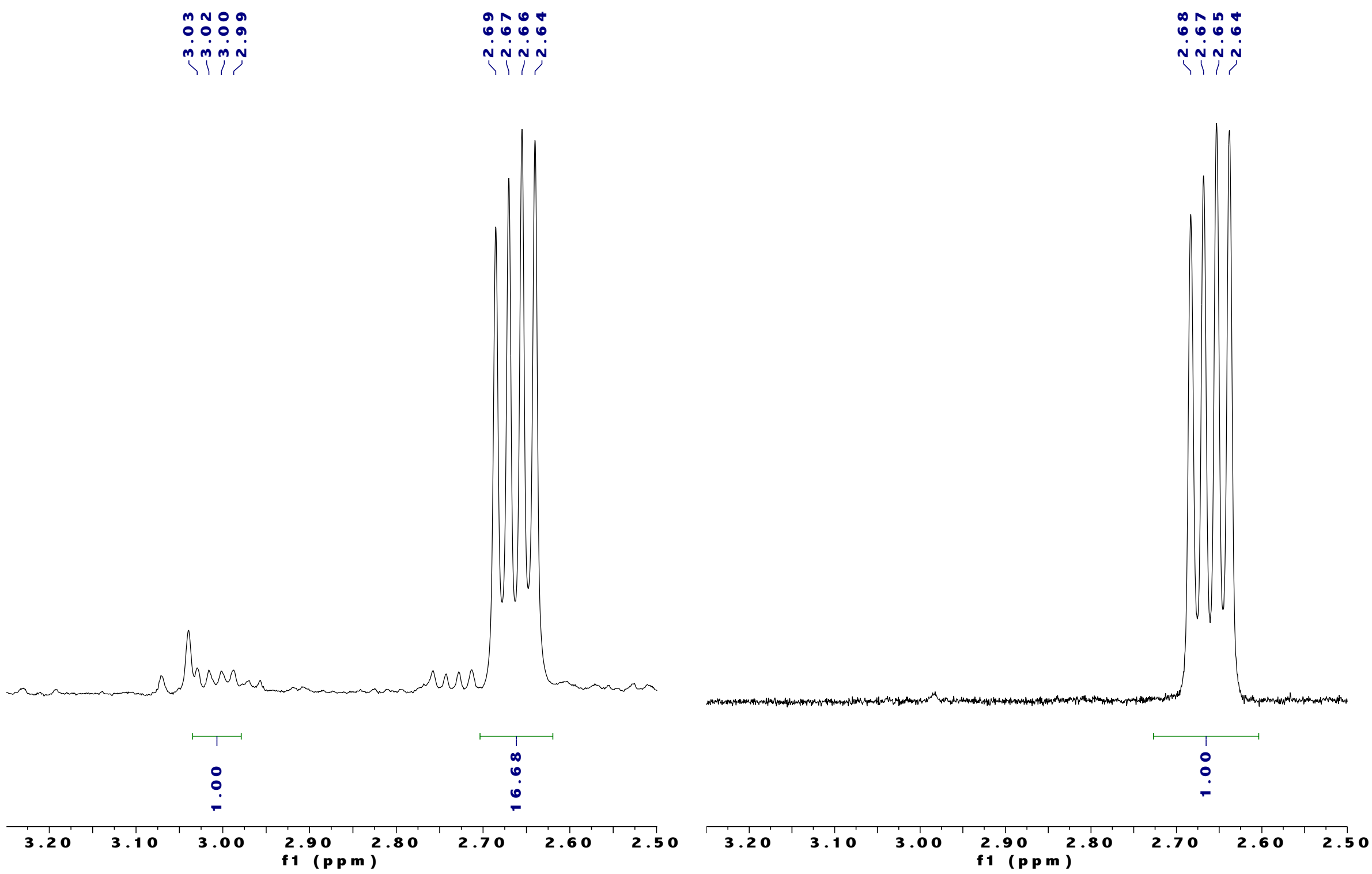

Figure S8. Determination of dr of **11** via ^{1}H-NMR, left crude mixture, right after purification.

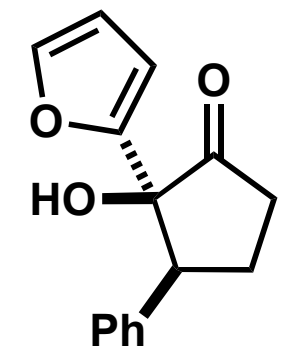

12

***cis*-2-(Furan-2-yl)-2-hydroxy-3-phenylcyclopentan-1-one** was synthesized according to the general procedure, for the ketonitrile cyclization. The crude product was obtained as a 18:1 *cis/trans* mixture of diastereomers as determined by ^{1}H-NMR. Purification using column chromatography [CH_2Cl_2→CH_2Cl_2/ethyl acetate 100:1 R_f(CH_2Cl_2) = 0.25] gave the product as a white solid in 81% yield (39 mg, 0.16 mmol) and a 22:1 d.r. (^{1}H-NMR). ^{1}H-NMR (400 MHz, C_6D_6) δ = 1.64-1.74 (m, 1H), 1.92-2.05 (m, 1H), 2.05-2.15 (m, 1H), 2.21-2.30 (m, 2H), 3.52 (dd, *J* = 10.9, 6.5 Hz, 1H), 5.99 (dd, *J* = 3.3, 1.9 Hz, 1H), 6.09 (d, *J* = 3.3 Hz, 1H), 7.00-7.13 (m, 6H).^{1}H-NMR (400 MHz, $CDCl_3$): δ = 2.28-2.38 (m, 1H), 2.41 (s, 1H), 2.43-2.65 (m, 2H), 2.69-2.79 (m, 1H), 3.87 (dd, *J* = 10.3, 6.6 Hz, 1H), 6.33 (d, *J* = 3.4 Hz, 1H), 6.35-6.38 (m, 1H), 7.12-7.19 (m, 2H), 7.22-7.35 (m, 3H), 7.44 (d, *J* = 2.1 Hz, 1H). ^{13}C-NMR (101 MHz, $CDCl_3$): δ = 23.85, 35.83, 51.06, 77.16, 78.47, 108.72, 110.76, 127.57, 128.53, 128.97, 136.90, 143.00, 152.99, 213.03. HRMS (pos. APCI): calcd for $C_{15}H_{14}O_3$ $[M]^+$: 242.0943, found: 242.0942. MS (EI): m/z (%) = 242.1 $[M]^+$, 224.1, 186.1, 157.1, 141.1, 129.1, 110.0, 104.0, 95.0, 77.1, 51.0. IR (ATR): ν [cm^{-1}] = 3450, 3030, 2959, 2891, 1746, 1602, 1499, 1455, 1402, 1339, 1279, 1159, 1130, 1093, 1065, 1014, 995, 957, 884, 868, 809, 739, 701, 662, 638, 552.

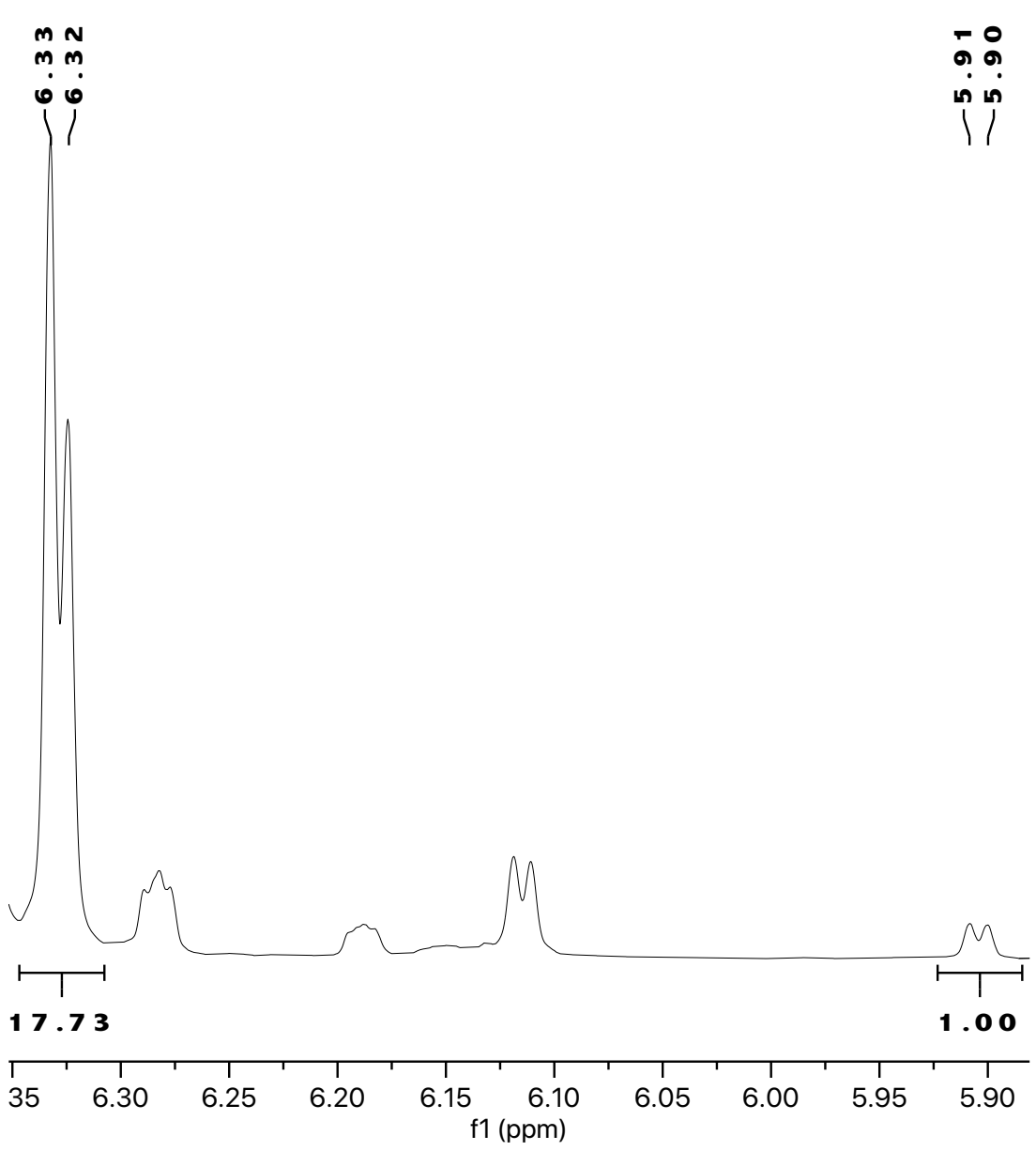

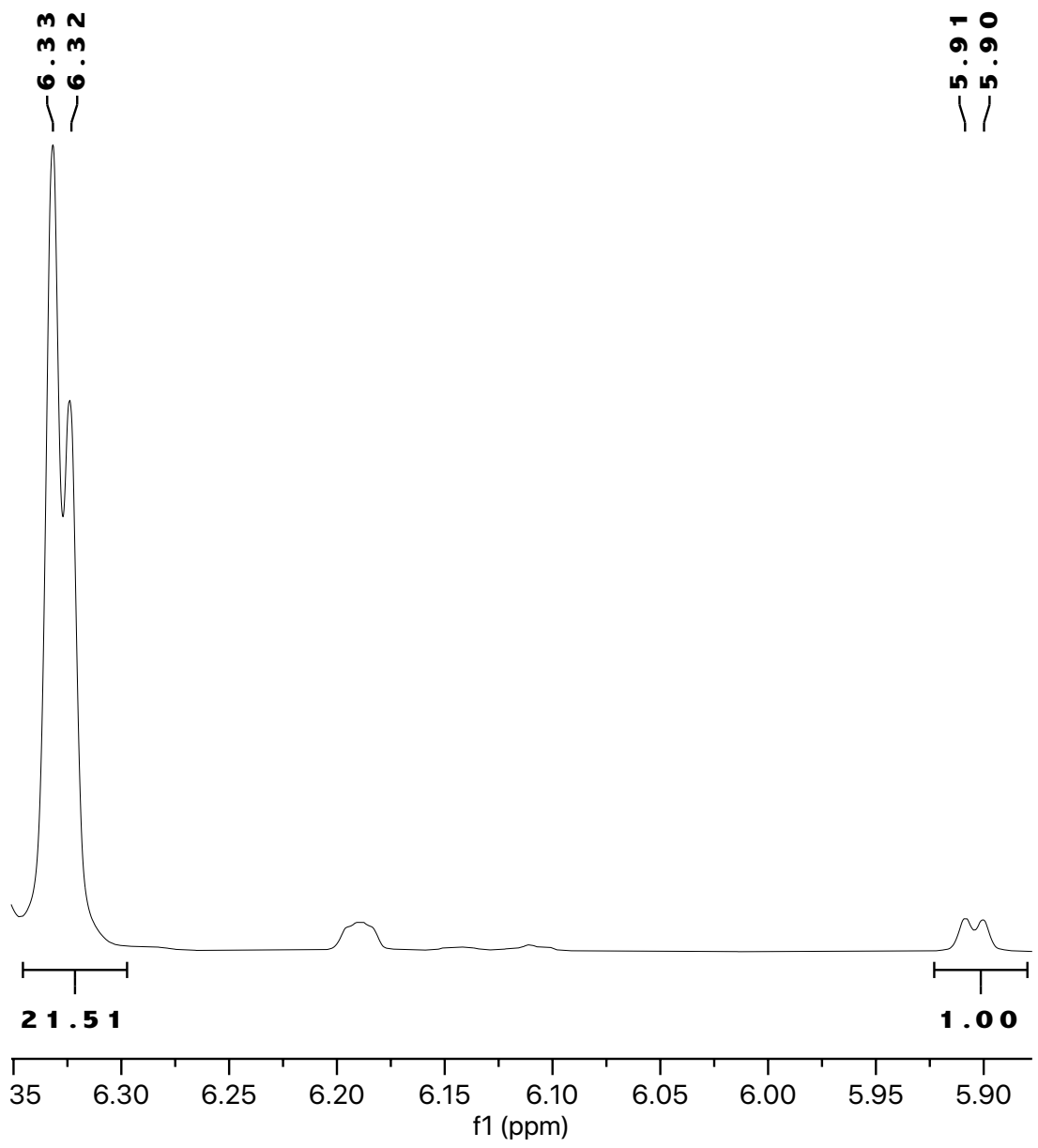

Figure S9. Determination of dr of **12** via ^{1}H-NMR, left crude mixture, right after purification.

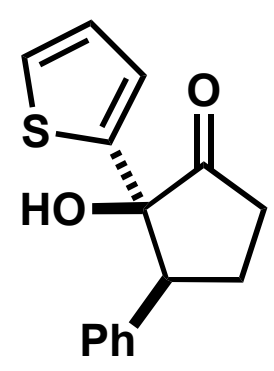

13

***cis*-2-Hydroxy-3-phenyl-2-(thiophen-2-yl)cyclopentan-1-one** was synthesized according to the general procedure, for the ketonitrile cyclization. The crude product was obtained as a 6:1 *cis*/*trans* mixture of diastereomers as determined by ^{1}H-NMR. Purification using column chromatography [CH_2Cl_2→CH_2Cl_2/ethyl acetate 100:1, R_f(CH_2Cl_2)= 0.25] gave the product as a white solid in 79% yield (41 mg, 0.159 mmol) and 6:1 d.r. (determined by ^{1}H-NMR). The NMR data is given for the mixture (ma = major; mi = minor). ^{1}H-NMR (400 MHz, C_6D_6) δ = 1.53-1.62 (m, 0.17H), 1.64-1.74 (m, 1H), 1.75-1.86 (m, 0.11H), 1.87-2.01 (m, 1H), 2.05-2.17 (m, 1H), 2.17-2.22 (m, 0.11H), 2.31 (s, 1H), 2.54 (s, 1H), 3.14 (dd, *J* = 11.1, 6.6 Hz, 1H), 3.26 (dd, *J* = 13.0, 6.4 Hz, 0.14H), 3.51 (s, 0.14H), 6.36-6.39 (m, 0.15H), 6.50 (dd, *J* = 5.0, 3.7 Hz, 0.15H), 6.62-6.66 (m, 0.13H), 6.67-6.72 (m, 1H), 6.76 (d, *J* = 3.6 Hz, 1H), 6.85 (d, *J* = 5.0 Hz, 1H), 7.05-7.22 (m, 5H). ^{1}H-NMR (400 MHz, $CDCl_3$): δ = 2.27-2.41 (m, 1H), 2.44-2.68 (m, 3H), 2.72-2.86 (m, 1H), 3.65 (dd, *J* = 10.0, 6.6 Hz, 1H), 6.95 (d, *J* = 3.6 Hz, 1H), 7.00 (dd, *J* = 5.1, 3.6 Hz, 1H), 7.16-7.36 (m, 6H).^{13}C-NMR (101 MHz, $CDCl_3$): δ = 21.21 (mi), 24.49 (ma), 34.44 (mi), 35.57 (ma), 52.37 (mi), 54.85 (ma), 79.43 (ma), 82.72 (mi), 124.74 (mi), 124.98 (ma), 125.75 (ma), 126.03 (mi), 126.22 (mi), 127.04 (ma), 127.27 (mi), 127.56 (ma), 128.06 (mi), 128.32 (mi), 128.36 (ma), 129.17 (ma), 136.88 (ma), 137.52 (mi), 141.08 (mi), 144.33 (ma), 213.42 (ma), 216.42 (mi). HRMS (pos. APCI): calcd for $C_{15}H_{15}O_2S$ $[M+H]^+$: 259.0787, found: 259.0768. MS (EI): m/z (%) = 258.1 $[M]^+$, 240.1, 202.1, 178.1, 152.1, 126.1, 111.0, 91.1. IR (ATR): ν [cm^{-1}] = 3440, 3030, 2955, 1743, 1602, 1497, 1454, 1401, 1329, 1277, 1239, 1141, 1114, 1065, 1047, 992, 956, 886, 838, 767, 700, 635, 619, 595, 552, 507.

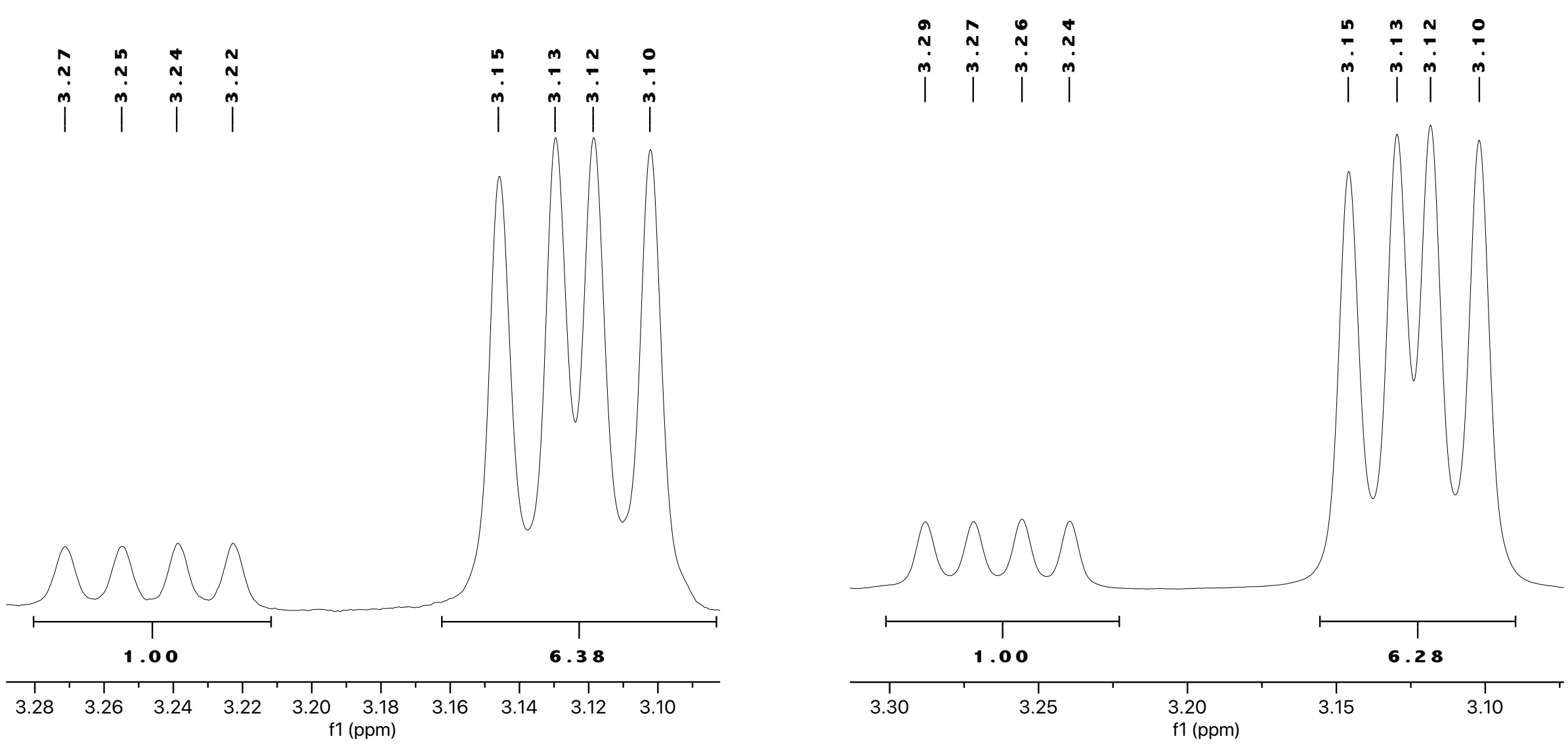

Figure S10. Determination of dr of **13** via ^{1}H-NMR, left crude mixture, right after purification.

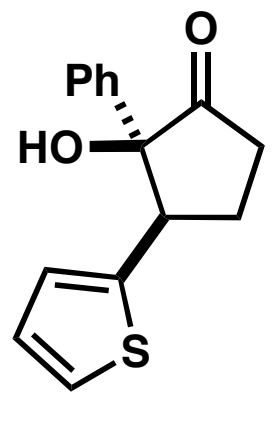

14

***cis*-2-Hydroxy-2-phenyl-3-(thiophen-2-yl)cyclopentan-1-one** was synthesized according to the general procedure, for the ketonitrile cyclization. The crude product was obtained as a 28:1 *cis*/*trans* mixture of diastereomers as determined by ^{1}H-NMR. Purification using column chromatography [CH_2Cl_2→CH_2Cl_2/ethyl acetate 100:1 R_f(CH_2Cl_2) = 0.35] gave the product as a white solid in 81% yield (42 mg, 0.16 mmol) and a 28:1 d.r. (^{1}H-NMR). ^{1}H-NMR (400 MHz, C_6D_6) δ =1.70-1.92 (m, 2H), 2.05-2.21 (m, 2H), 2.32 (dd, *J* = 18.4, 8.0 Hz, 1H), 3.26 (dd, *J* = 11.9, 6.3 Hz, 1H), 6.54 (d, *J* = 3.6 Hz, 1H), 6.67 (dd, *J* = 5.0, 3.6 Hz, 1H), 6.81 (d, *J* = 5.2 Hz, 1H), 7.04-7.14 (m, 5H). ^{1}H-NMR (400 MHz, $CDCl_3$): δ = 2.39 (s, 1H), 2.40-2.62 (m, 3H), 2.78-2.89 (m, 1H), 3.83-3.89 (m, 1H), 6.76 (d, *J* = 3.5 Hz, 1H), 6.95 (dd, *J* = 5.1, 3.5 Hz, 1H), 7.18-7.21 (m, 1H), 7.22-7.27 (m, 2H), 7.29-7.40 (m, 3H). ^{13}C-NMR (101 MHz, $CDCl_3$): δ = 26.49, 36.87, 50.87, 77.16, 80.98, 125.05, 126.23, 126.33, 126.75, 128.16, 128.46, 139.30, 139.79, 214.72. HRMS (pos. APCI): calcd for $C_{15}H_{14}O_2S$ $[M]^+$: 258.0715, found: 258.0714. MS (EI): m/z (%) = 258.1 $[M]^+$, 240.1, 207.0, 178.1, 153.1, 124.0, 110.0, 105.1 77.1 66.1. IR (ATR): ν [cm^{-1}] = 3456, 3382, 2945, 2885, 1740, 1494, 1447, 1399, 1283, 1263, 1213, 1130, 1065, 1027, 988, 950, 854, 751, 726, 696, 561, 511.

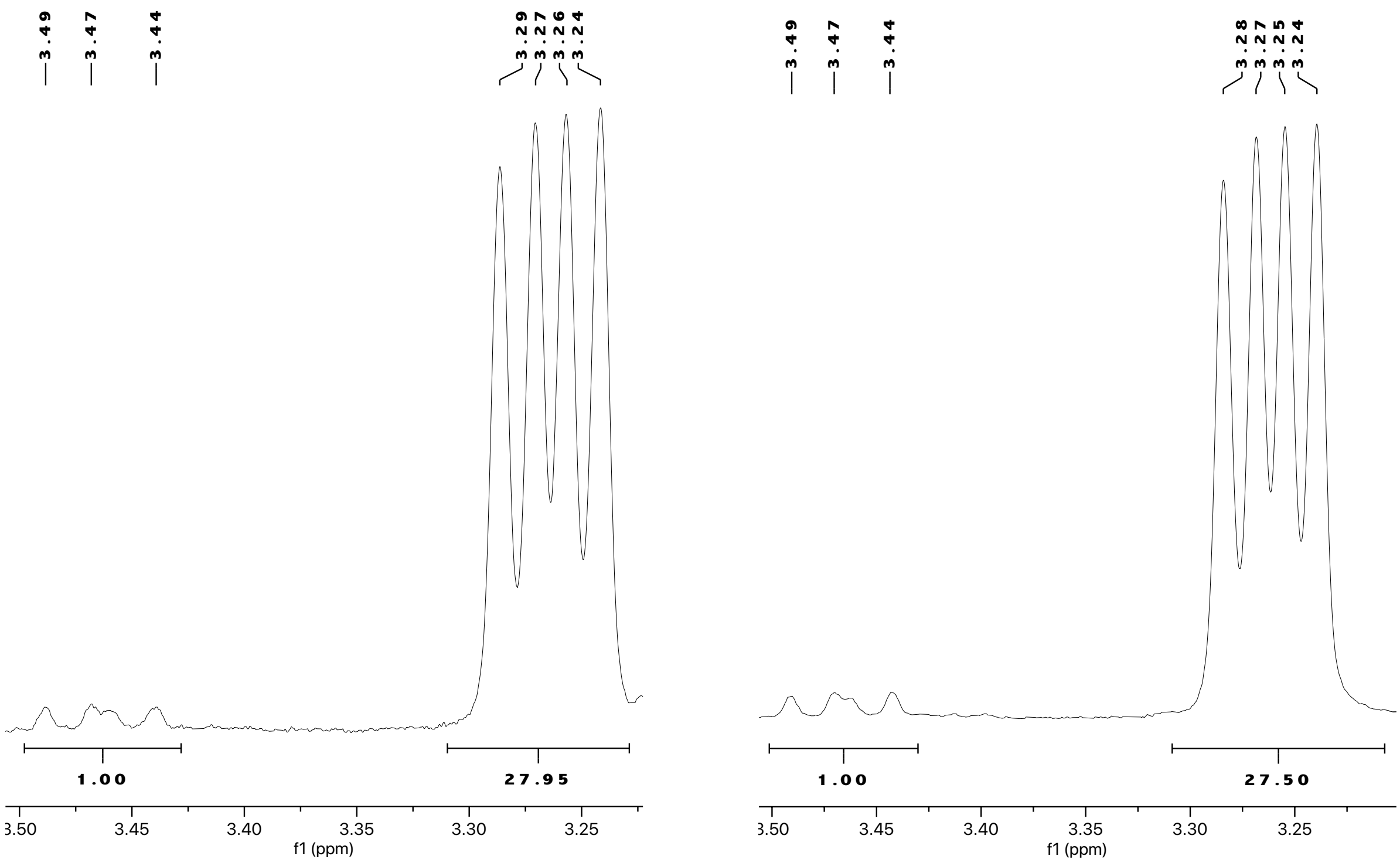

Figure S11. Determination of dr of **14** via ^{1}H-NMR, left crude mixture, right after purification.

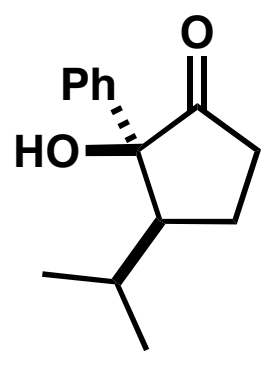

15

***cis*-2-Hydroxy-3-isopropyl-2-phenylcyclopentan-1-one** was synthesized according to the general procedure, for the ketonitrile cyclization. The crude product was obtained as a 27:1 *cis*/*trans* mixture of diastereomers as determined by ^{1}H-NMR. Purification using column chromatography [CH_2Cl_2/ethyl acetate 100:1→33:1, R_f(CH_2Cl_2/ethyl acetate 100:1) = 0.2] gave the product as a white solid in 76% yield (36.4 mg, 0.167 mmol) and a 39:1 d.r. (^{1}H-NMR). ^{1}H-NMR (400 MHz, $CDCl_3$): δ = 0.71 (d, J = 6.7 Hz, 3H), 0.94 (d, J = 6.7 Hz, 3H), 1.79-1.93 (m, 1H), 2.04 (dq, J = 13.6, 6.7 Hz, 1H), 2.09-2.25 (m, 2H), 2.37-2.50 (m, 1H), 2.52-2.64 (m, 2H), 7.18-7.42 (m, 5H). ^{13}C-NMR (101 MHz, $CDCl_3$): δ = 20.69, 22.50, 22.84, 27.57, 37.09, 55.18, 81.53, 125.40, 127.52, 128.41, 142.97, 218.55. HRMS (neg. ESI): calcd for $C_{14}H_{17}O_2$ [M-H]$^-$: 217.1234, found: 217.1232. MS (EI): m/z (%) = 218.0 [M]$^+$, 190.2, 162.2, 147.1, 120.1, 105.1, 77.1. IR (ATR): ν [cm^{-1}] = 3447, 3027, 2957, 2871, 1738, 1602, 1496, 1471, 1447, 1403, 1385, 1366, 1334, 1278, 1216, 1186, 1128, 1096, 963, 913, 827, 751, 715, 698, 663, 579, 498.

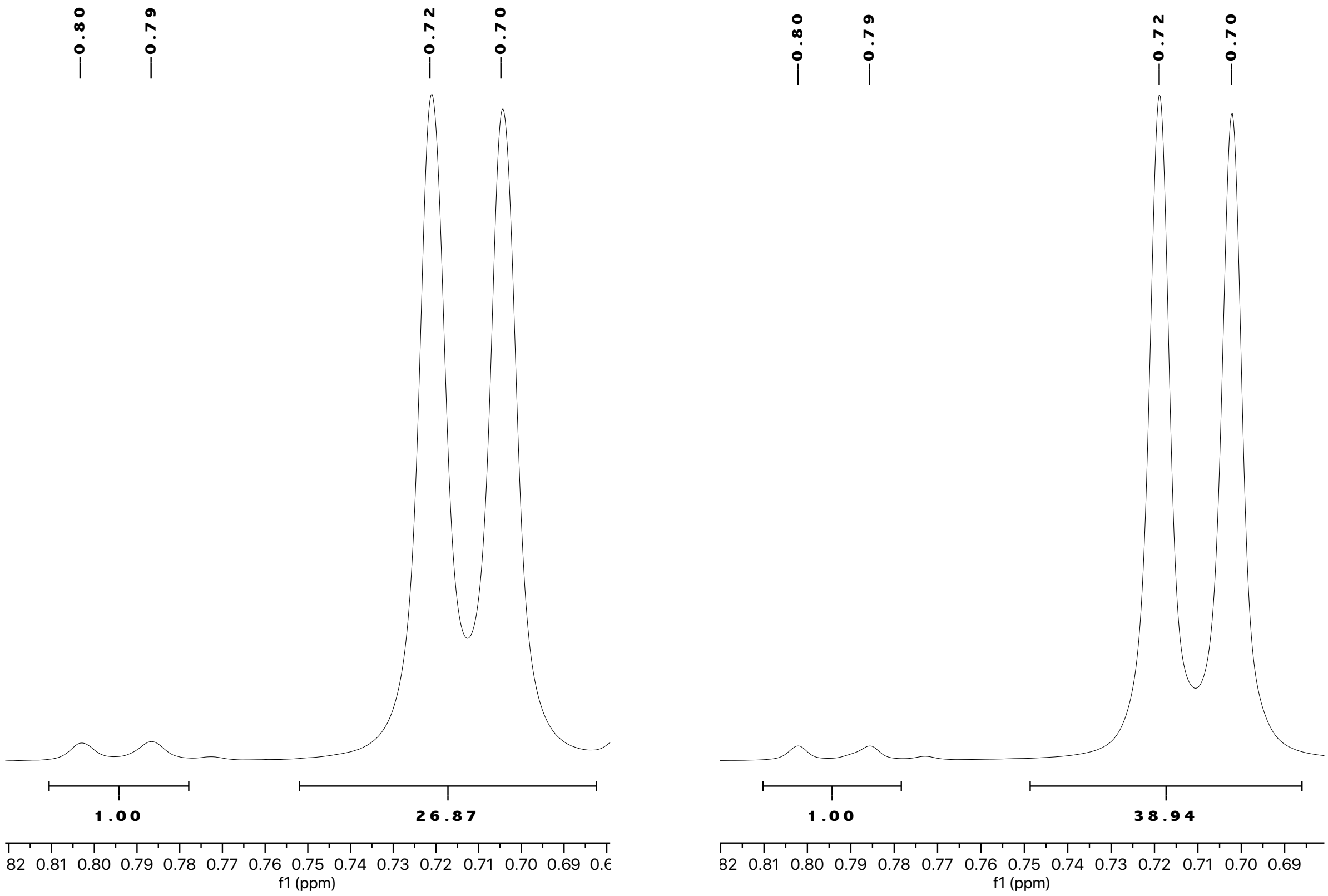

Figure S12. Determination of dr of **15** via ^{1}H-NMR, left crude mixture, right after purification.

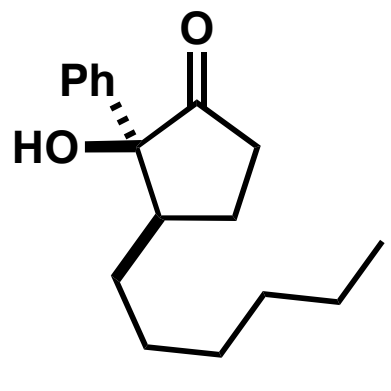

16

cis**-3-*****n*****-Hexyl-2-hydroxy-2-phenylcyclopentan-1-one** was synthesized according to the general procedure, for the ketonitrile cyclization. The crude product was obtained as a 11:1 *cis*/*trans* mixture of diastereomers as determined by ^{1}H-NMR. Purification using column chromatography [CH_2Cl_2→CH_2Cl_2/ethyl acetate 100:1 (R_f(CH_2Cl_2) = 0.2) gave the product as a clear oil in 76% yield (39.5 mg, 0.150 mmol) and 22:1 d.r. (by ^{1}H-NMR; 26:1 d.r. by GCMS). ^{1}H-NMR (400 MHz, $CDCl_3$): δ = 0.85 (t, J = 6.9 Hz, 3H), 1.09-1.44 (m, 9H), 1.47-1.62 (m, 1H), 1.73-1.89 (m, 1H), 2.05-2.15 (m, 1H), 2.19-2.31 (m, 1H), 2.34-2.46 (m, 2H), 2.54-2.66 (m, 1H), 7.25-7.32 (m, 3H), 7.32-7.39 (m, 2H). ^{13}C-NMR (101 MHz, $CDCl_3$): δ = 14.19, 22.73, 24.20, 26.99, 27.49, 29.57, 31.88, 36.43, 49.06, 82.00, 125.89, 127.82, 128.46, 141.46, 218.34. HRMS (pos. APCI): calcd for $C_{17}H_{25}O_2$ [M+H]$^+$: 261.1849, found: 261.1845. MS (EI): m/z (%) = 260.2 [M]$^+$, 242.2, 204.2, 171.1, 148.1, 133.1 120.1 105.1, 91.1, 77.1, 67.1, 55.1. IR (ATR): ν [cm^{-1}] = 3437, 3061, 3028, 2954, 2924, 2855, 1739, 1603, 1495, 1485, 1447, 1402, 1376, 1279, 1214, 1153, 1103, 1061, 1031, 996, 934, 913, 869, 825, 753, 714, 698, 661, 625, 576, 548.

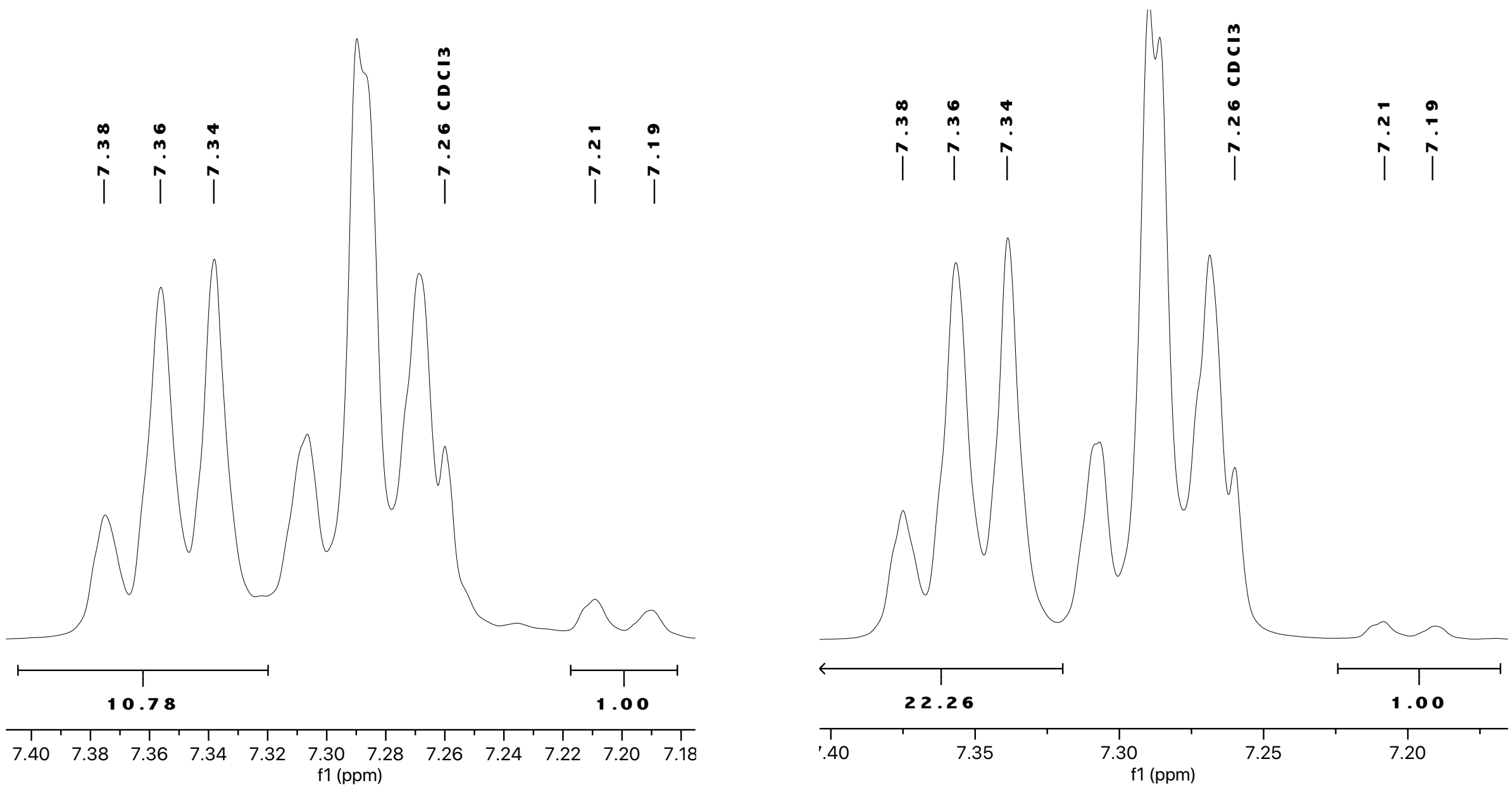

Figure S13. Determination of dr of **16** via ^{1}H-NMR, left crude mixture, right after purification.

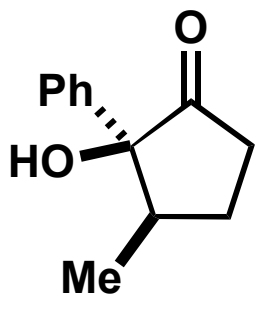

17

cis-2-Hydroxy-3-methyl-2-phenylcyclopentan-1-one.

Synthesized following the representative procedure on a 0.2 mmol scale. The crude product mixture showed a diastereomeric ratio of 14:1 *cis*/*trans* as determined by ^{1}H-NMR in C_6D_6. Purification using column chromatography [CH_2Cl_2/ethyl acetate 100:1→33:1, R_f(CH_2Cl_2/ethyl acetate 33:1) = 0.15] gave the product as a white solid in 63% yield (24.0 mg, 0.126 mmol) and a 22:1 d.r.(GCMS). Major diastereomer: ^{1}H-NMR (500.3 MHz, $CDCl_3$): δ = 1.05 (d, *J* = 6.9 Hz, 3H), 1.62 (br, OH), 1.83 (dq, *J* = 8.6, 12.7 Hz, 1H), 2.06 (dddd, *J* = 3.7, 6.4, 9.2, 12.8 Hz, 1H), 2.38-2.45 (m, 2H), 2.64 (ddd, *J* = 3.7, 8.8, 19.5 Hz, 1H), 7.29-7.32 (m, 3H), 7.35-7.39 (m, 2H). ^{13}C-NMR (125.8 MHz, $CDCl_3$): δ = 12.1, 26.3, 36.5, 43.9, 81.9, 125.8, 127.9, 128.4, 140.6, 219.4. MS (EI, 70 eV): m/z (%) = 190.1 [M]$^+$ (20), 162.1 (45), 134.1 (100), 133.1 (87), 120 (5), 115 (10), 105 (75), 91 (9), 79.1 (6), 77 (38), 55 (13), 51 (7). HRMS (APCI) calcd for $C_{12}H_{18}NO_2^+$ [M+NH$_4$]$^+$: 208.13321, found: 208.13279. IR (ATR): ν [cm^{-1}] = 3434, 3028, 2961, 2879, 1737, 1602, 1494, 1450, 1401, 1374, 1382, 1279, 1216, 1176, 1150, 1126, 1089, 1061, 1025, 989, 963, 930, 906, 879, 813, 751, 698, 574, 543, 509, 461.

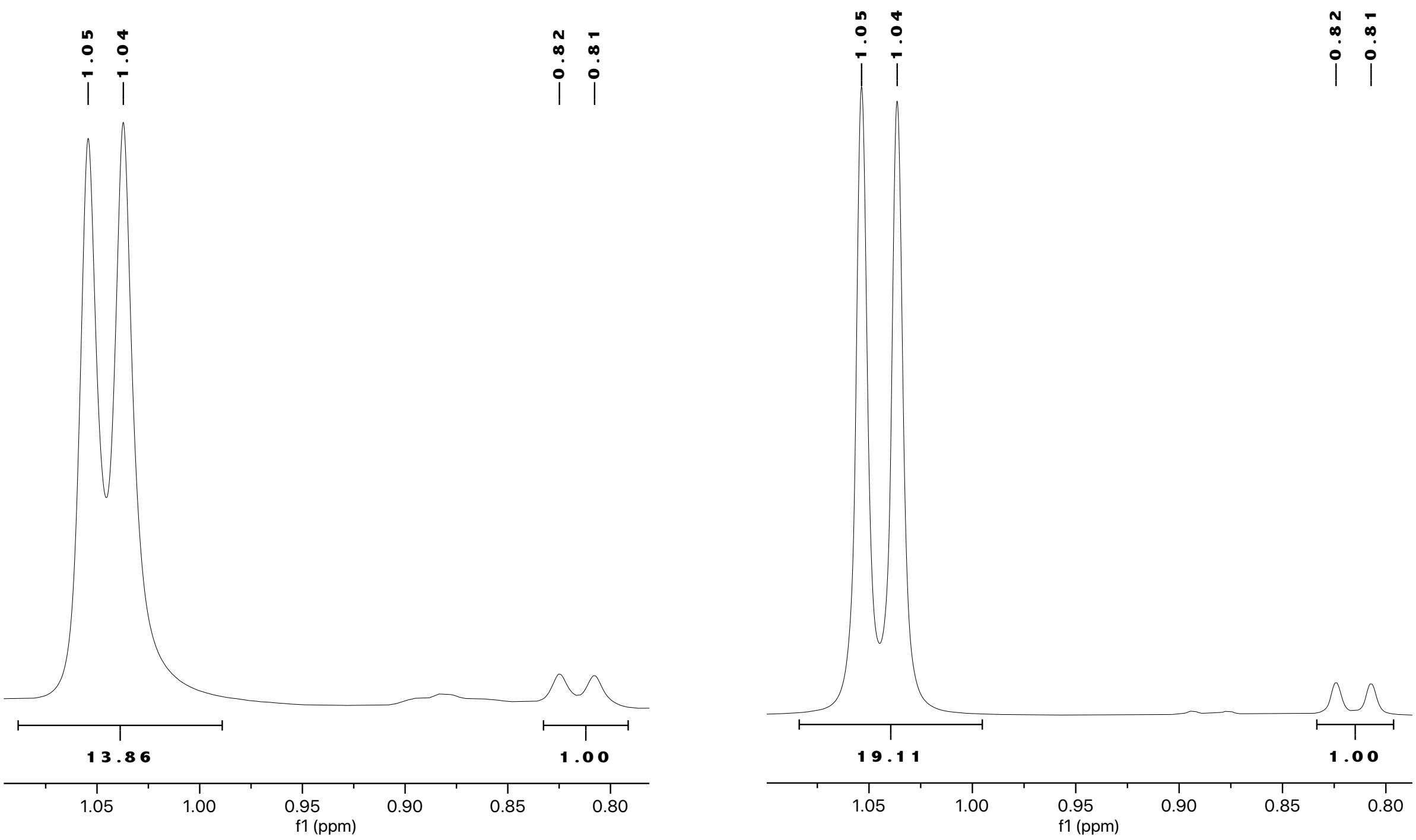

Figure S14. Determination of dr of **17** via ^{1}H-NMR, left crude mixture, right after purification.

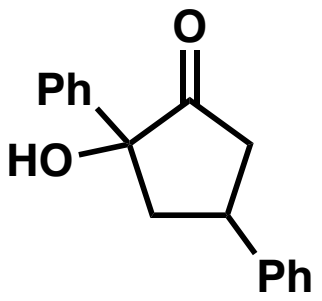

18
(1.2:1 d.r.)

2-Hydroxy-2,4-diphenylcyclopentan-1-one. Prepared according to the general procedure for the ketonitrile cyclization on a 0.2 mmol scale with Et_3N•HCl instead of morph•HCl. ^{1}H-NMR analysis of the crude mixture after workup showed a 1.2:1 diastereomeric mixture. Purification using flash chromatography (pentane:EtOAc, 8:1) gave the product as a mixture of diastereomers in 69% yield (34.8 mg, 0.14 mmol) as a white solid. The purification led to partial separation of the diastereomers and fractions, which allowed for the characterization of the two diastereomers by NMR.

Diastereomer 1: ^{1}H-NMR (400 MHz, $CDCl_3$): δ = 7.45-7.22 (m, 10H), 3.79 (tt, J = 12.5, 6.6 Hz, 1H), 3.05 (ddd, J = 18.1, 7.0, 2.5 Hz, 1H), 2.77 (ddd, J = 14.0, 6.2, 2.5 Hz, 1H), 2.71-2.59 (m, 2H), 2.29 (ddd, J = 13.9, 11.9, 1.8 Hz, 1H). ^{13}C-NMR (101 MHz, $CDCl_3$): δ = 215.76, 142.23, 141.43, 128.77, 128.52, 127.96, 126.91, 126.77, 125.38, 80.74, 47.94, 45.83, 37.89. HRMS (pos. APCI): calcd for $C_{17}H_{16}O_2$ $[M+NH_4]^+$: 270.1489, found 270.1489. MS (CI, 70 eV): m/z = 270.1 $[M+NH_4]^+$ (100), 254.1 (20), 235.1 (95), 224.1 (21), 120.0 (35), 105.0 (20), 78.0 (15). IR(ATR): ν[cm^{-1}] = 3429, 3029, 1746, 1602, 1496, 1448, 1401, 1158, 1129, 1074, 1013, 923, 758, 698.

Diastereomer 2: ^{1}H-NMR (400 MHz, $CDCl_3$): δ = 7.49-7.31 (m, 8H), 7.26 (td, J = 7.0, 3.4 Hz, 4H), 3.39-3.21 (m, 1H), 3.18 (s, 1H), 3.05-2.89 (m, 2H), 2.55 (dd, J = 19.7, 10.7 Hz, 1H), 2.39 (t, J = 12.8 Hz, 1H). ^{13}C-NMR (101 MHz, $CDCl_3$): δ = 217.59, 142.36, 140.24, 129.01, 128.79, 128.68, 126.95, 126.85, 126.12, 82.57, 44.53, 43.45, 35.63.

HRMS (pos. APCI): calcd for $C_{17}H_{16}O_2$ $[M+NH_4]^+$: 270.1489, found 270.1489. MS (CI, 70 eV): m/z = 270.1 $[M+NH_4]^+$, 254.1, 236.1, 235.1, 224.1, 209.1, 193.1, 131.0, 120.0, 105.0, 78.0. IR(ATR): ν [cm^{-1}] = 3397, 3028, 1740, 1602, 1496, 1447, 1402, 1141, 1068, 1014, 924, 756.52, 698.

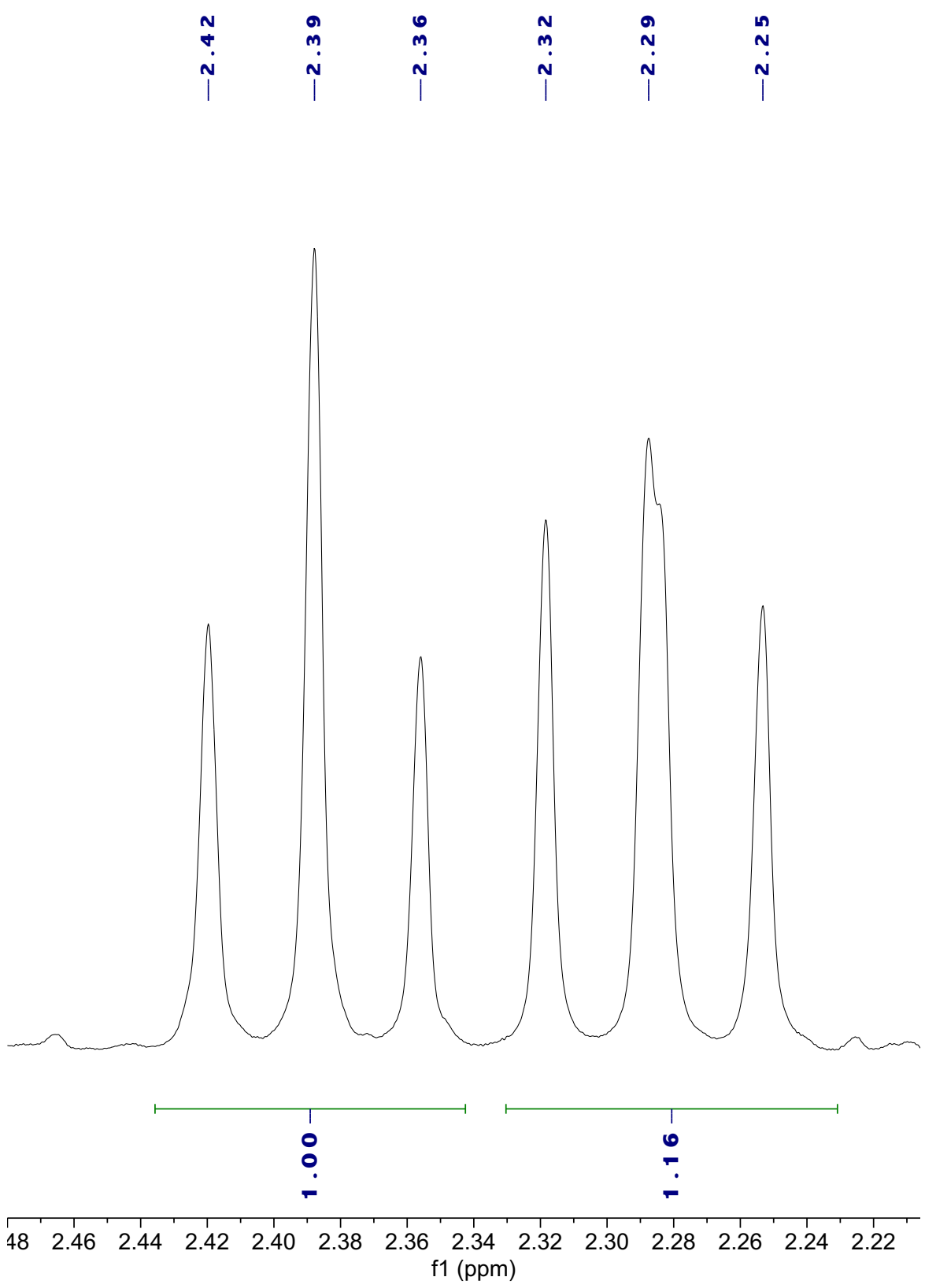

Figure S15. Excerpt of the ^{1}H-NMR (400 MHz, $CDCl_3$) spectrum of crude **18** used for the determination of the d.r.

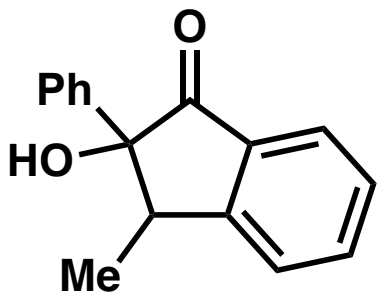

19
(1.1:1 d.r.)

2-Hydroxy-3-methyl-2-phenyl-2,3-dihydro-1*H*-inden-1-one. Literature-known compound.[33] Synthesized following the general ketonitrile cyclization procedure. The ratio of product diastereomers determined from the crude mixture was 1.1:1. Purification via flash chromatography (petroleum ether/EtOAc, 10:1) allowed partial separation of the diastereomers giving diastereomer 1 (7 mg, 0.029 mmol, R_f = 0.3) and diastereomer 2 (5 mg, 0.021 mmol, R_f = 0.2) and a fraction containing a mixture of both (15 mg, 0.063 mmol) in a combined yield of 57% (0.113 mmol).

<u>*Diastereomer 1:*</u> ^{1}H-NMR (400 MHz, $CDCl_3$): δ = 1.22 (d, *J* = 7.2 Hz, 3H), 3.06 (br, 1H), 3.35 (q, *J* = 7.2 Hz, 1H), 6.88 (t, *J* = 7.5 Hz, 1H), 6.92 (d, *J* = 7.7 Hz, 1H), 6.98-7.06 (m, 3H), 7.10 (t, *J* = 7.5 Hz, 1H), 7.29 (d, *J* = 7.9 Hz, 2H), 7.69 (d, *J* = 7.6 Hz, 1H). ^{13}C-NMR (101 MHz, $CDCl_3$): δ = 16.96, 48.02, 82.76, 125.07, 125.42, 125.60, 127.63, 128.20, 128.64, 134.11, 135.66, 143.92, 158.40, 205.70.

<u>*Diastereomer 2:*</u> ^{1}H-NMR (400 MHz, $CDCl_3$): δ = 0.86 (d, *J* = 7.3 Hz, 3H), 3.06 (br, 1H), 3.47 (q, *J* = 7.3 Hz, 1H), 6.89-6.92 (m, 2H), 6.96-6.99 (m, 3H), 7.10 (dt, *J* = 1.2, 7.6 Hz, 1H), 7.74 (d, *J* = 7.6 Hz, 1H). ^{13}C-NMR (101 MHz, $CDCl_3$): δ = 14.53, 46.74, 85.50, 124.32, 125.10, 126.13, 127.74, 127.95, 128.37, 135.60, 135.70, 141.29, 156.29, 206.03.

MS (EI, 70 eV, diastereomer mixture): m/z (%) = 238.2 [M]$^+$ (100), 223.2 (20), 219.2 (8), 193.2 (13), 178.2 (12), 165.1 (15), 133.1 (18), 105.1 (93), 77.1 (37). HRMS (pos. APCI): calcd for $C_{16}H_{14}O_2$ [M]$^+$: 238.0988, found 238.0985. IR (ATR, diastereomer mixture): ν [cm^{-1}] = 3432, 3060, 2969, 2931, 1714, 1605, 1494, 1468, 1447, 1376, 1325, 1291, 1227, 1156, 1134, 1071, 1025, 984, 908, 871, 752, 700, 660, 632, 587, 497.

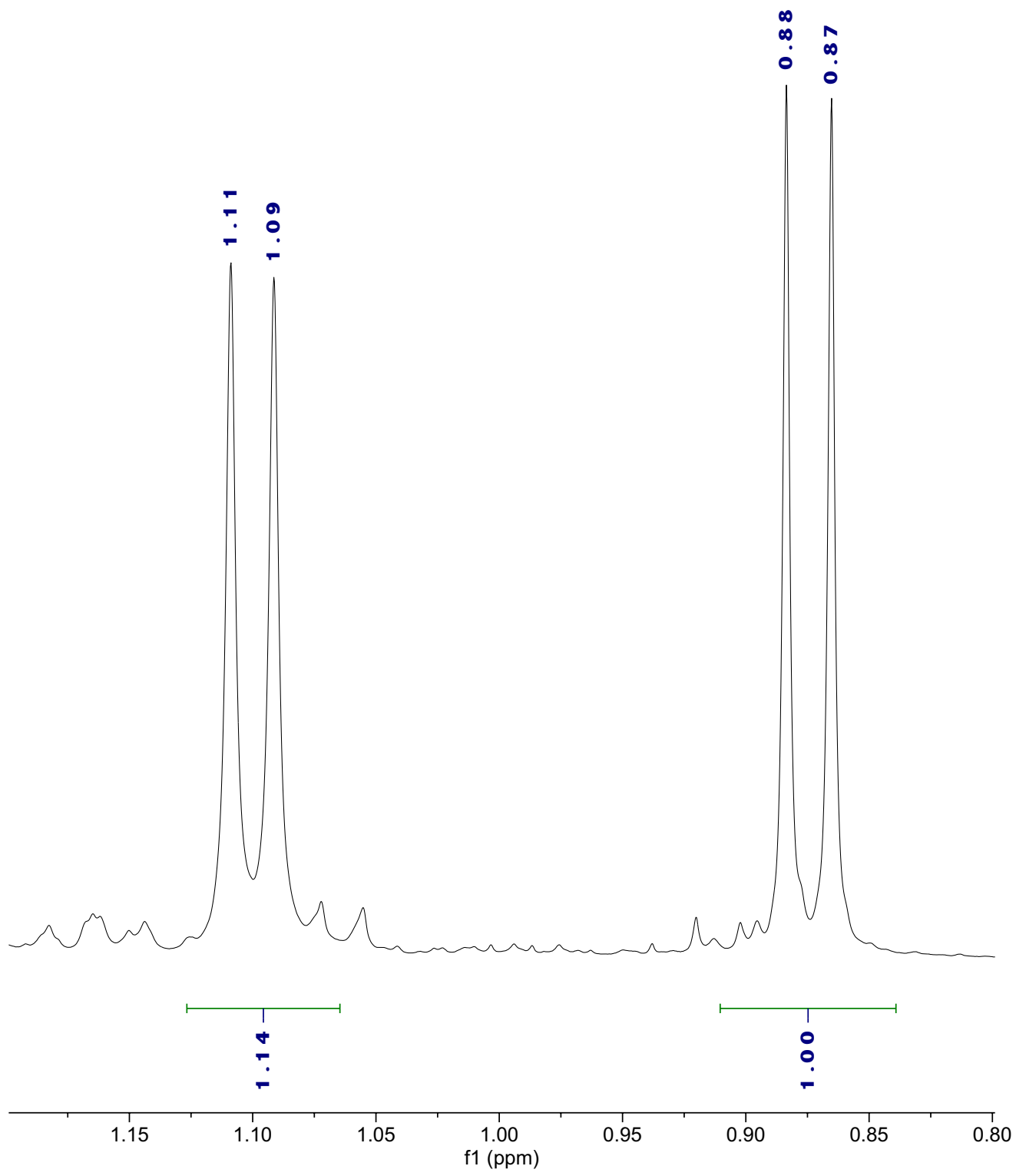

Figure S16. Excerpt of the ^{1}H-NMR (400 MHz, $CDCl_3$) spectrum of crude **19** for determination of the d.r.

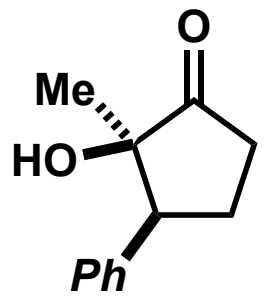

20

***cis*-2-Hydroxy-2-methyl-3-phenylcyclopentan-1-one** was synthesized according to the general procedure for the ketonitrile cyclization. ^{1}H-NMR analysis of the crude mixture after workup showed a 1.3:1 diastereomeric mixture (presumably *cis*/*trans*, assigned in analogy to **2**). The crude product was purified using column chromatography [CH_2Cl_2/ethyl acetate 100:1→33:1 R_f(CH_2Cl_2/ethyl acetate 33:1) = 0.3], which allowed a separation of the two diastereomers. Diastereomer 1 (presumably *cis*) was obtained as a white solid in 13% yield (5.1 mg, 0.027 mmol). Diastereomer 2 (presumably *trans*) was obtained as a white solid in 8% yield (3.0 mg, 0.016 mmol).
^{1}H-NMR (400 MHz, $CDCl_3$): δ = 0.90 (s, 3H), 2.10-2.33 (m, 2H), 2.36-2.51 (m, 1H), 2.56-2.71 (m, 2H), 3.29-3.39 (m, 1H), 7.22-7.42 (m, 5H). ^{13}C-NMR (101 MHz, $CDCl_3$): δ = 18.82, 21.40, 33.63, 51.11, 80.03, 127.22, 128.28, 128.45, 138.14, 220.00. HRMS (neg. ESI): calcd for $C_{12}H_{13}O_2$ $[M-H]^-$: 189.0921, found: 189.0916. MS (EI): m/z (%) = 190.1 $[M]^+$, 172.1, 147.1, 134.1 129.1, 119.1, 115.1 104.1, 91.1, 77.1 65.0. IR (ATR): ν [cm^{-1}] = 3446, 3061, 3030,2967, 2924, 1748, 1601, 1497, 1451, 1399, 1365, 1286, 1119, 1077, 1056, 769, 701, 603, 491.

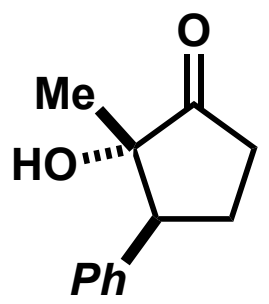

20'

***trans*-2-Hydroxy-2-methyl-3-phenylcyclopentan-1-one.** Isolated as minor fraction from the ketonitrile cyclization to **21** above. ^{1}H-NMR (400 MHz, $CDCl_3$): δ = 1.26 (s, 3H), 2.15-2.27 (m, 1H), 2.31-2.51 (m, 2H), 2.61-2.74 (m, 1H), 2.97-3.08 (m, 1H), 7.27-7.42 (m, 5H). ^{13}C-NMR (101 MHz, $CDCl_3$): δ = 21.59, 24.46, 35.17, 53.37, 76.68, 127.54, 128.58, 129.21, 137.95, 216.72. HRMS (neg. ESI): calcd for $C_{12}H_{13}O_2$ $[M-H]^-$: 189.0921, found: 189.0916. MS (EI): m/z (%) = 190.1 $[M]^+$, 172.1, 134.1, 115.0, 104.1, 91.1, 77.1, 65.1. IR (ATR): ν [cm^{-1}] = 3444, 3061, 3029, 2970, 2884, 1741, 1602, 1497, 1453, 1402, 1375, 1279, 1215, 1092, 1072, 1052, 927, 887, 828, 800, 770, 705, 604, 491.

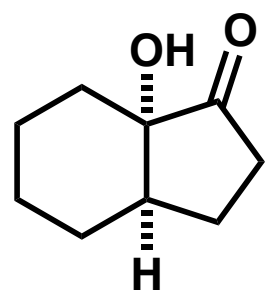

21

7a-Hydroxyoctahydro-1*H*-inden-1-one. Literature-known compound.[34] Synthesized according to the general ketonitrile cyclization procedure. The crude product was purified using flash chromatography [CH_2Cl_2/ethyl acetate 50:1→25:1 R_f(CH_2Cl_2/ethyl acetate 25:1) = 0.25]. The pure product was obtained as a colorless oil in 16% yield (5.0 mg, 0.032 mmol). The analytic data was in agreement with the literature values. ^{1}H-NMR (400 MHz, $CDCl_3$): δ = 1.22-1.34 (m, 1H), 1.39-1.60 (m, 6H), 1.74-1.95 (m, 3H), 2.05-2.17 (m, 1H), 2.22-2.34 (m, 2H), 2.38-2.50 (m, 1H). ^{13}C-NMR (101 MHz, $CDCl_3$): δ = 20.83, 20.89, 21.11, 24.55, 29.59, 33.33, 41.00, 77.97, 219.85.

Hammett Correlation Data

Table S7. Hammett plot data.

Substituent	σ_p	d.r. (x:1)[a]	ln(d.r.)
AcO	0.31	17	2.83
Br	0.23	14	2.64
Cl	0.23	14	2.64
F	0.06	23	3.14
H	0	22	3.09
Me	−0.17	30	3.40
OMe	−0.27	28	3.33

[a] Diastereomeric ratios determined by ^{1}H-NMR of the crude product.

Data for Aminoketones 22–24

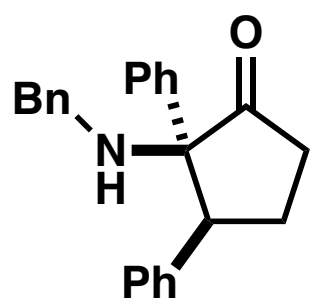

22

2-(Benzylamino)-2,3-diphenylcyclopentan-1-one. The reaction was set up analogous to the general procedure for the ketonitrile cyclizations but with modified workup: A flame-dried 10 ml Schlenk tube, filled with argon and a magnetic stir bar was charged with morpholine hydrochloride (14.3 mg, 0.116 mmol, 1.00 equiv), Cp_2TiCl_2 (2.9 mg, 0.012 mmol, 10 mol%), and zinc dust (15.2 mg, 0.232 mmol, 2.00 equiv). The tube was evacuated and backfilled with argon again. THF (0.2 ml) was added while stirring and the mixture turned lime-green. After 20 minutes, iminonitrile **S31** (39.3 mg, 0.116 mmol, 1.00 equiv) dissolved in 0.4 ml THF was added to the reaction mixture, followed by TMSCl (29.5 µl, 0.232 mmol, 2.00 equiv). The tube was sealed with a greased glass stopper and the reaction mixture heated to 35 °C for 24 h while stirring. Afterwards, the green mixture was allowed to cool to room temperature, 1N HCl was added, and the mixture was transferred into a separatory funnel using overall 4 ml of 1N HCl and 10 ml Et_2O, followed by rinsing with 3 ml CH_2Cl_2. The organic layer was extracted with 1N HCl (6 × 4 ml). The combined aqueous layers were then basified (pH 11) with solid K_2CO_3 at which point the solution became turbid. Extraction with CH_2Cl_2 (5×20 ml) followed by drying (Na_2SO_4), filtration, and concentration provided **22** as pure product. The original organic layer, however, contained additional product and was basified with K_2CO_3, dried (Na_2SO_4), and concentrated. The diastereomeric ratio was determined from the crude product to be 4.3:1. Purification by preparative TLC (CH_2Cl_2, R_f = 0.61). The desired product **22** was obtained as a mixture of diastereomers (5.7:1 d.r.) in 47% overall yield (18.6 mg, 0.054 mmol) as a yellow solid. ^{1}H-NMR (400 MHz, C_6D_6): δ = 1.55-1.67 (m, 1H), 1.59 (br, 1H) 2.06 (dddd, J = 1.9, 9.1, 9.1, 18.3 Hz, 1H), 2.29-2.45 (m, 1H), 2.55 (ddd, J = 1.9, 8.6, 18.6 Hz, 1H), 3.05 (ddd, J = 2.0, 6.3, 12.0 Hz, 1H), 3.53 (d, J = 14.3 Hz, 1H), 3.72 (d, J =14.2 Hz, 1H), 6.73-6.79 (m, 2H), 6.85-6.90 (m, 1H), 6.92-6.97 (m, 2H), 6.98-7.06 (m, 5H), 7.09-7.14 (m, 5H). ^{13}C-NMR (101 MHz, C_6D_6): δ = 23.68, 37.39, 46.97, 57.69, 71.15, 126.99,127.19, 127.54, 127.91, 128.20, 128.23, 128.38,128.59,129.66, 136.89, 139.66, 141.42, 211.84. HRMS (pos. APCI): calcd for $C_{24}H_{23}NO^+$ [M+H]$^+$: 342.1852; found: 342.1859. MS (EI, 70 eV): m/z (%) = 341 [M]$^+$ (<10), 313 (11), 286 (23), 285 (100), 284 (57), 194 (23), 193 (16), 167 (24), 165 (13), 104 (17), 91 (47). MS (CI, NH_3): m/z (%) = 342 [M+H]$^+$ (100), 285 (14). IR (ATR): ν [cm^{-1}] = 3029, 2950, 1735, 1601, 1495, 1452, 1265, 1116, 1028, 917, 734, 697.

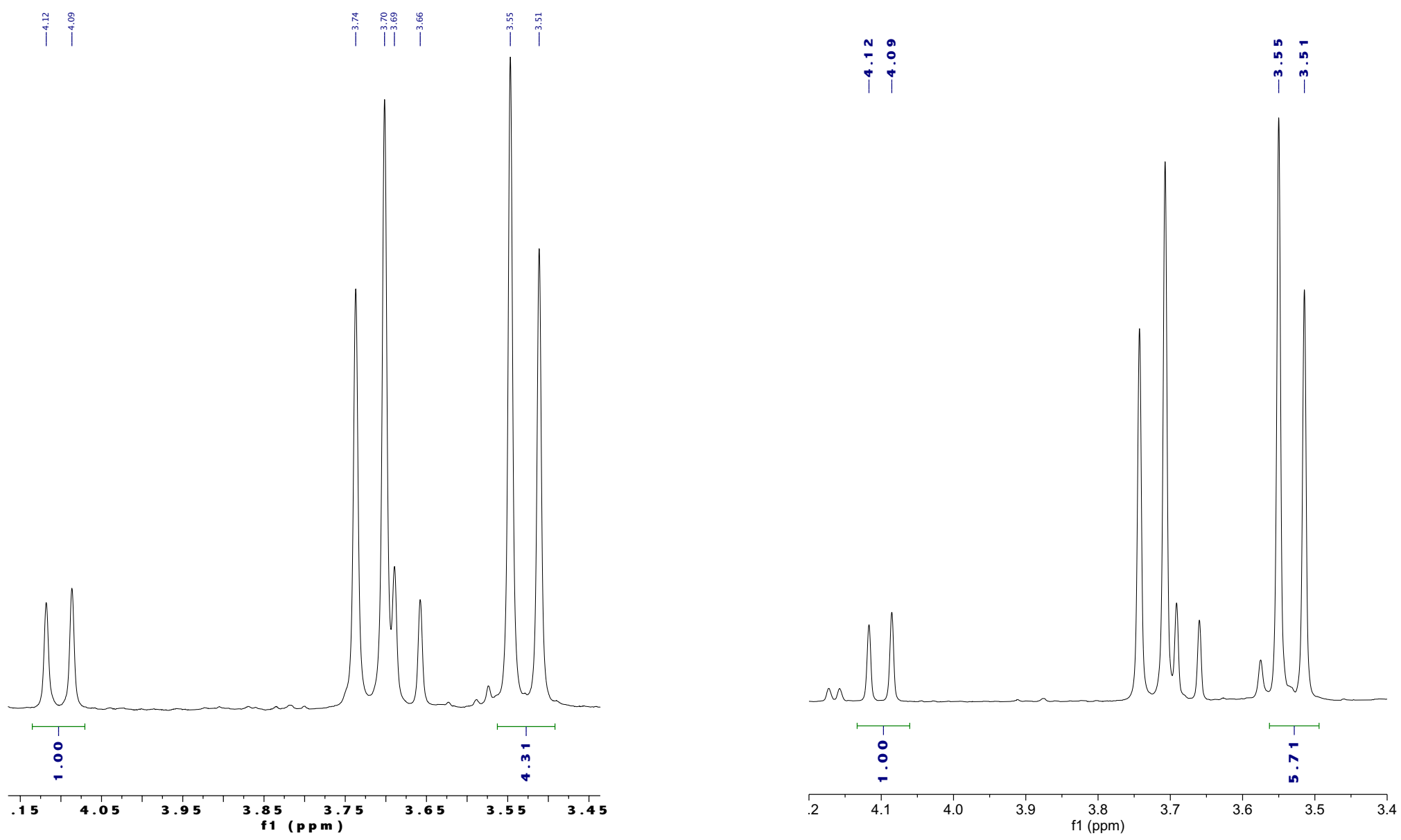

Figure S17. Excerpt of the ^{1}H-NMR (400 MHz, $CDCl_3$) spectrum of the crude product and the isolated product mixture.

The stereochemical assignment of the major diastereomer is proposed to be *cis* in analogy to the hydroxyketone products based on a NOESY NMR experiment (Figure S18). The minor diastereomer shows a NOE correlation between the benzylic methine proton and the benzylic methylene group. This suggests that the benzylamine substituent and the methine proton are on the same side of the ring for this compound. This correlation is absent in the major diastereomer suggesting the opposite configuration. Although not a definite proof, this assignment was in agreement with the diastereoselectivity observed for the ketonitrile cyclization.

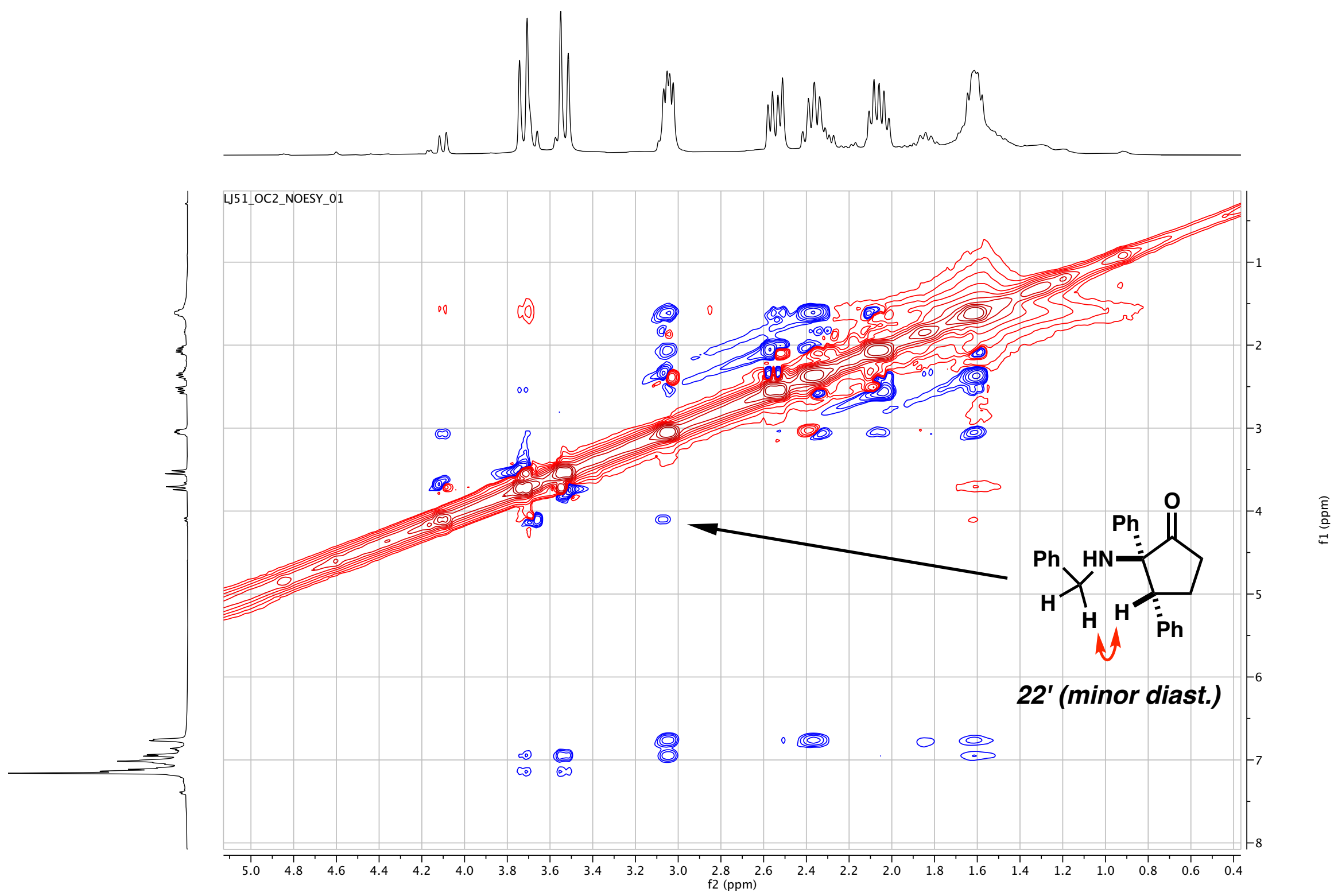

Figure S18. NOESY NMR showing an interaction of the benzylic CH_2 group at 4.15 ppm with the tertiary CH at 3.05 ppm for the minor diastereomer. This interaction does not occur for the major diastereomer.

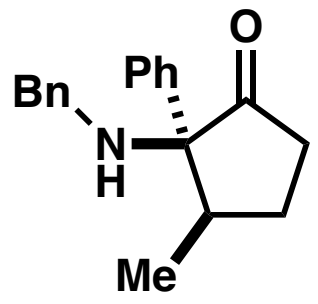

23

2-(Benzylamino)-3-methyl-2-phenylcyclopentan-1-one. Synthesized in analogy to **22** above from **S32** (32.4 mg, 0.117 mmol). The combined organic extracts were dried (Na_2SO_4), filtered, and concentrated. The crude yellow oil consisted of a 3.8:1 diastereomeric mixture as determined by ^{1}H-NMR. It was purified by preparative TLC (CH_2Cl_2, R_f = 0.60) and the desired compound was obtained as a 3.5:1 mixture of diastereomers in 65% yield (21.4 mg, 0.077 mmol) in form of a white solid.
^{1}H-NMR (400 MHz, C_6D_6): δ = 0.79 (d, *J* = 7.0 Hz, 3H), 1.19-1.25 (m, 1H), 1.41-1.52 (m, 1H), 1.59 (br, 1H), 1.90 (ddd, *J* = 8.6, 8.6, 19.4 Hz, 1H), 1.97-2.09 (m, 1H), 2.19 (ddd, *J* = 3.8, 9.2, 19.4 Hz, 1H), 3.52 (d, *J* = 13.7 Hz, 1H), 3.56 (d, *J* = 13.7 Hz, 1H), 7.05-7.14 (m, 3H), 7.19-7.23 (m, 2H), 7.24-7.28 (m, 2H), 7.29-7.38 (m, 3H). ^{13}C-NMR (101 MHz, C_6D_6): δ = 12.81, 26.06, 35.83, 43.48, 47.59, 71.62, 127.15, 127.33, 127.96, 128.24, 128.58, 128.63, 139.85, 141.27, 213.07. HRMS (pos. APCI): calcd for $C_{19}H_{21}NO$ $[M+H]^+$: 280.1696, found 280.1703. MS (EI, 70 eV): m/z (%) = 280 $[M+H]^+$ (13), 251 (35), 224 (16), 223 (98), 222 (100), 208 (10), 104 (15), 91 (61). MS (CI, NH_3): m/z (%) = 280 $[M+H]^+$ (100), 223 (25), 222 (13). IR (ATR): ν [cm^{-1}] = 3028, 2958, 2925, 1728, 1603, 1494, 1452, 1404, 1377, 1145, 1069, 1028, 922, 753, 699.

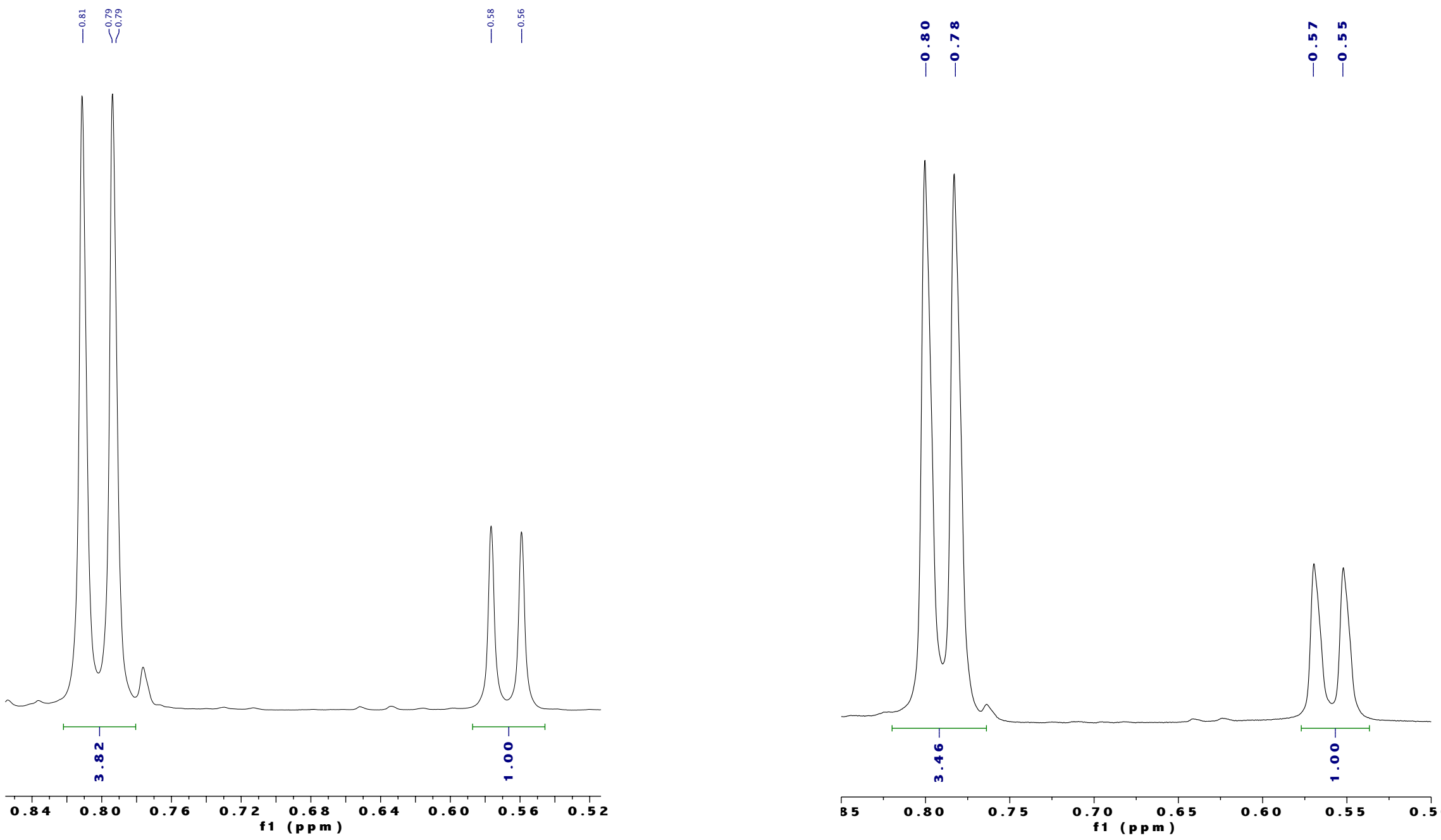

Figure S19. Excerpt of the ^{1}H-NMR (400 MHz, $CDCl_3$) spectrum of the crude product and the isolated product mixture.

The stereochemical assignment of the major diastereomer is proposed to be *cis* in analogy to the hydroxyketone products based on a NOESY NMR experiment (Figure S20). The major diastereomer shows a NOE correlation between a N-C*H*$_2$Ph proton and the methyl group. A corresponding correlation was not visible for the minor diastereomer. In addition, a NOE correlation between the tertiary CH and the ortho protons of one of the Ph groups is visible.
This suggests that the benzylamine substituent and the methyl group are on the same side of the molecule. Although not a definite proof, this assignment was in agreement with the diastereoselectivity observed for the ketonitrile cyclization.

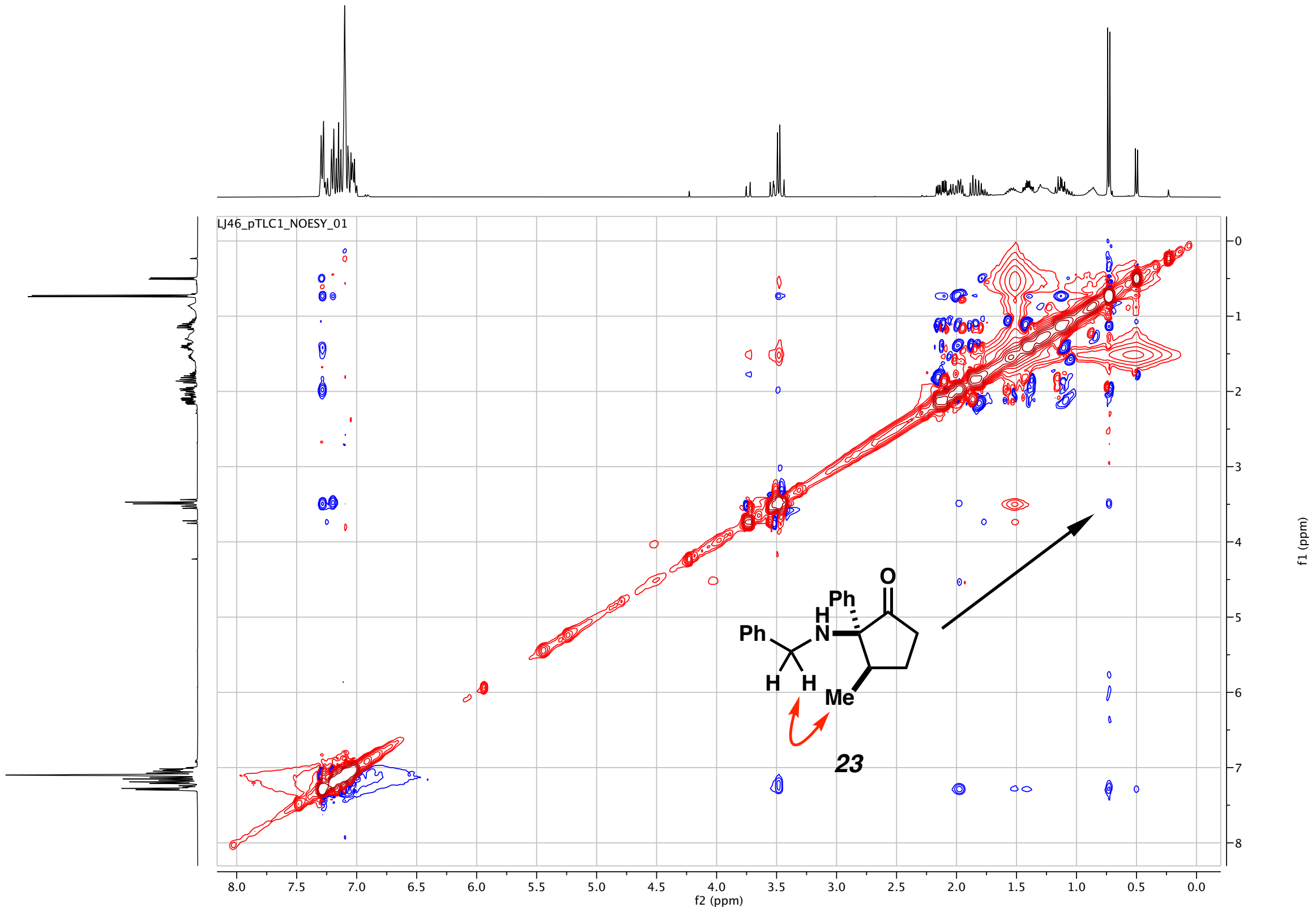

Figure S20. NOESY NMR showing an interaction of the benzylic CH_2 group at 3.5 ppm with the methyl group at 0.7 ppm. This interaction is absent for the minor diastereomer.

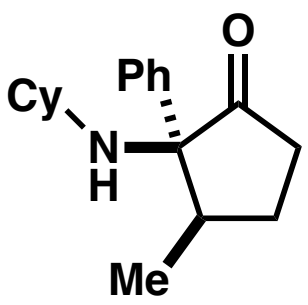

24

2-(Cyclohexylamino)-3-methyl-2-phenylcyclopentan-1-one. Synthesized in analogy to **22** on a 0.24 mmol scale with the following modification. A flame-dried argon-backfilled 10 ml Schlenk tube containing a stir bar was charged with morpholine·HCl (30.2mg, 0.244 mmol, 1.00 equiv), Cp_2TiCl_2 (6.1 mg, 0.024 mmol, 10 mol%), and zinc dust (32.0 mg, 0.489 mmol, 2.00 equiv). The tube was evacuated and backfilled with argon followed by addition of 0.2 ml THF while stirring. The suspension was stirred for 20 min after which it had turned lime-green. Meanwhile **29** (65.6 mg, 0.244 mmol, 1.00 equiv) was dissolved in 0.5 ml dry THF. It was added to the reaction mixture followed by TMSCl (62 µl, 0.489 mmol, 2.00 equiv) directly afterward. The tube was sealed and immersed into a preheated heating block filled with sand at 35 °C on top of an IKAmag temperature modulator. The reaction was stirred for 24 h after which it was allowed to cool to room temperature. The mixture was quenched with 1 M HCl (4 ml) and transferred into a separatory funnel with the aid of 10 ml Et_2O, followed by rinsing with 3 ml CH_2Cl_2. The organic layer was extracted with 6 × 4 ml 1N HCl. The combined aqueous layers were basified (pH 11) with solid K_2CO_3 during which the solution became turbid and then extracted with 5 × 20 ml CH_2Cl_2. The combined organic layers were dried over Na_2SO_4, filtered, and concentrated. The crude yellow oil was purified by preparative TLC (CH_2Cl_2, R_f = 0.37). The desired product was obtained as a slightly yellow oil that slowly solidified in the freezer to become an off-white solid and as a single diastereomer (>40:1 d.r.) in 64% yield (42.1 mg, 0.155 mmol). ^{1}H-NMR (400 MHz, C_6D_6): δ = 0.88 (d, *J* = 7.0 Hz, 3H), 0.90-1.11 (m, 5H), 1.25-1.34 (m, 2H), 1.46-1.58 (m, 5H), 1.79 (br d, *J* = 12.2 Hz,1H), 1.88 (ddd, *J* = 6.4, 9.7, 19.4 Hz, 1H), 2.17 (ddd, *J* = 6.1, 9.1, 19.6 Hz, 1H), 2.18-2.25 (m, 1H), 2.26-2.33 (m, 1H), 7.08 (tt, *J* = 1.2, 7.3 Hz, 1H), 7.19 (t, *J* = 7.7 Hz, 2H), 7.42 (dd, *J* = 1.3, 8.4 Hz, 2H). ^{13}C-NMR (101 MHz, C_6D_6): δ = 13.9,

25.39, 25.57, 25.60, 26.19, 34.32, 35.40, 35.67, 42.34, 51.96, 71.88, 127.30, 128.17, 128.40, 140.35, 214.62. HRMS (pos. APCI): calcd for $C_{18}H_{25}NO$ $[M+H]^+$ 272.2009, found 272.2013. MS (EI, 70 eV): m/z (%) = 271 $[M]^+$ (<10), 243 (23), 216 (16), 215 (100), 214 (84), 200 (11), 186 (41), 172 (11), 158 (10), 144 (16), 134 (68), 132 (21), 131 (10), 117 (17), 115 (23), 104 (52), 91 (18), 77 (11), 55 (20), 41 (11). MS (CI, NH_3): m/z (%) = 273 (28), 272 $[M+H]^+$ (100), 215 (18). IR (ATR): ν [cm^{-1}] = 2925, 2852, 1732, 1447, 1406, 1377, 1264, 1145, 1081, 736, 700, 572.

The stereochemical assignment of the major diastereomer is proposed to be *cis* in analogy to the hydroxyketone products based on a NOESY NMR experiment (Figure S21). A strong NOE correlation between the methine proton and the ortho protons of the phenyl group is visible. Furthermore, only a weak correlation is observed for the methyl group and the ortho protons. This suggests that the methyl and phenyl group are on opposite sides of the ring. Although not a definite proof, this assignment was in agreement with the diastereoselectivity observed for the ketonitrile cyclization.

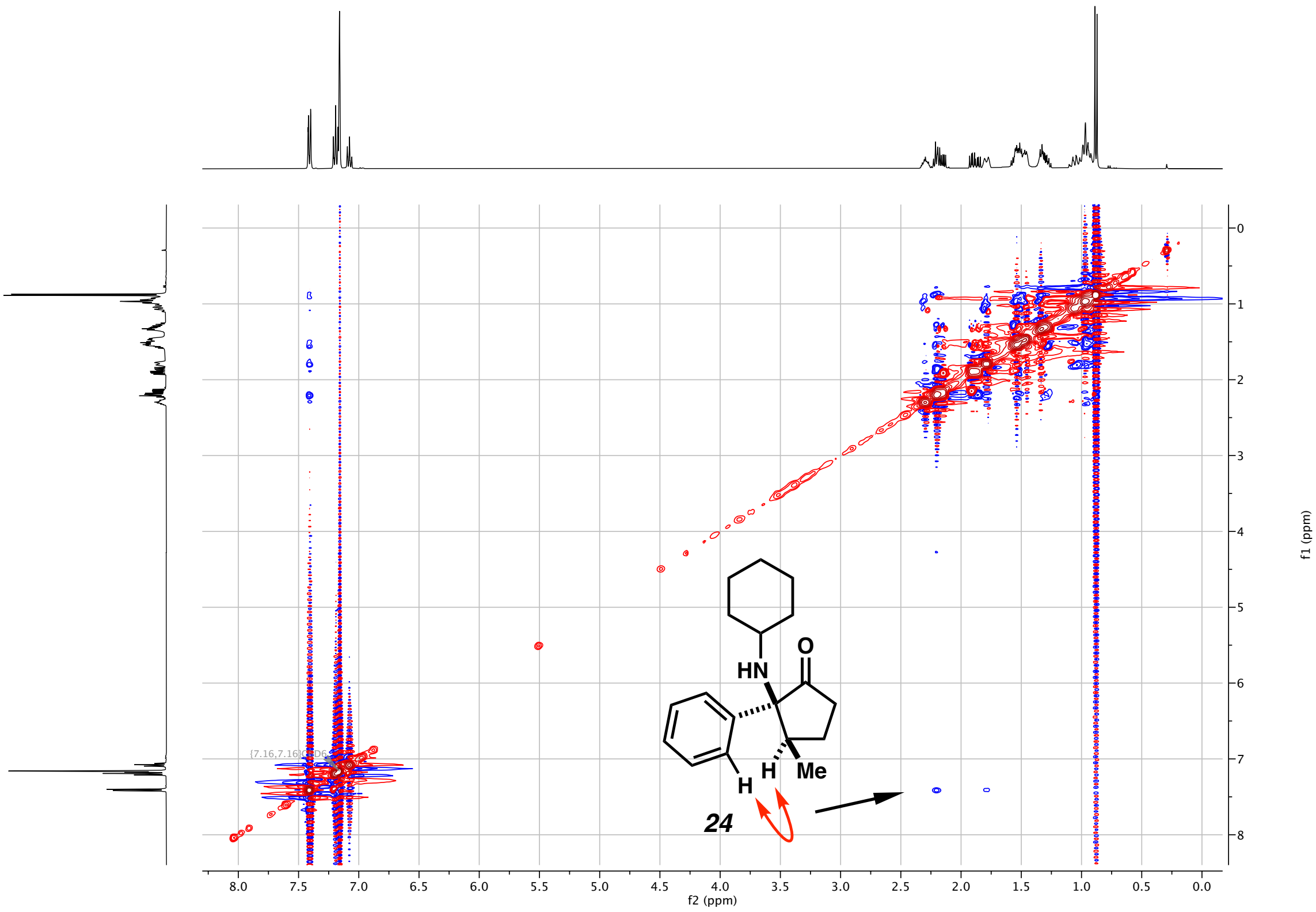

Figure S21. NOESY NMR showing a strong interaction between the tertiary proton and the *ortho*-CH of the phenyl group. Only a weak NOE correlation is observed between the *ortho*-CH and the the CH_3 group.

Synthesis of 25 for Structure Determination

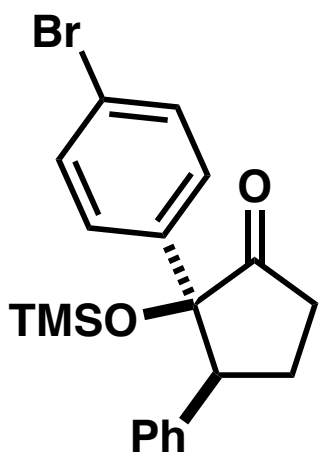

25

***cis*-2-(4-Bromophenyl)-3-phenyl-2-((trimethylsilyl)oxy)cyclopentan-1-one**. Prepared for assignment of the relative configuration by X-ray diffraction analysis at an early stage of the project. The representative procedure was carried out on a 1 mmol scale and Et_3N•HCl was used as hydrochloride additive. The workup was modified as follows to hydrolyze the imine but prevent hydrolysis of the silyl ether:

The reaction was quenched by addition of water (5 ml). Glacial acetic acid (10 drops) was added and the mixture was stirred at room temperature for 6 h. The layers were separated and the aqueous layer extracted with CH_2Cl_2 (3 × 50 ml). The combined organic layers were dried (Na_2SO_4), filtered, and the solvent removed under reduced pressure. The residue was crystallized from hot *i*-PrOH to afford the desired product as a brown solid in 46% yield (186 mg, 0.46 mmol). R_f = 0.5 (hexanes:EtOAc, 3:1). ^{1}H-NMR (300.1 MHz, $CDCl_3$): δ = –0.01 (s, 9H), 2.14-2.26 (m, 1H), 2.43-2.59 (m, 2H), 2.72-2.87 (m, 1H), 2.94-3.03 (m, 1H), 6.76 (ddd, *J* = 2.0, 2.4, 8.8 Hz, 2H), 6.98-7.03 (m, 2H), 7.17-7.26 (m, 3H), 7.34 (ddd, *J* = 2.1, 2.5, 8.8 Hz, 2H). ^{13}C-NMR (100.6 MHz, $CDCl_3$): δ = 1.2, 25.0, 37.7, 59.0, 83.1, 121.4, 127.2, 127.6, 128.5, 129.9, 130.8, 136.8, 139.7, 215.3. MS (EI, 70 eV): m/z (%) = 404 $[M]^+$ (49), 402 $[M]^+$ (48), 346 (90), 282.9 (19), 267.1 (21), 257 (10), 204.1 (25), 178.1 (16), 105.1 (14), 73.1 (100), 45 (14). HRMS (APCI) calcd for $C_{20}H_{27}{}^{79}BrNO_2Si^+$ $[M+NH_4]^+$: 420.09889, found: 420.09885. IR (ATR): ν [cm^{-1}] = 3088, 3062, 3029, 2956, 2896, 1745, 1488, 1455, 1403, 1251, 1146, 1094, 1072, 1040, 1006, 920, 887, 843, 808, 752, 698.

Product Derivatization

1,2-Addition Reactions

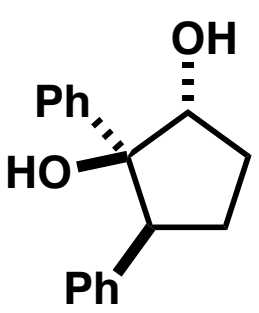

26

***cis,trans*-1,5-Diphenylcyclopentane-1,2-diol.** Synthesized following our earlier procedure as follows.[35] A flame-dried argon-filled Schlenk tube was charged with a solution of L-Selectride (1 M in THF, 1.7 ml, 1.7 mmol, 2.1 equiv) in additional THF (3 mL) and cooled to −78 ºC. A solution of **2** (202 mg, 0.80 mmol, 1.0 equiv) in THF (3 mL) was added dropwise over 10 min, and the mixture was stirred for 1 h at −78 ºC. The solution was allowed to warm to room temperature, then cooled to 0 ºC, and quenched by the addition of water (4 mL) and EtOH (4 mL), and then allowed to warm to room temperature again. Aqueous H_2O_2-solution (30%, 4 mL) and aq. NaOH (3 M, 4 mL) were added, and the mixture was stirred for an additional 1.5 h at 22 ºC. The mixture was extracted with CH_2Cl_2 (4 × 20 mL), and the combined organic layers were washed with sat. aq. $Na_2S_2O_3$-solution (2 × 20 mL), brine (2 × 20 mL), dried over Na_2SO_4, filtered, and concentrated. The product was isolated as a white solid in analytically pure form in 83% yield (168 mg, 0.661 mmol). Crystallization from 2-propanol gave a single crystal suitable for X-ray crystallography. ^{1}H-NMR (400 MHz, C_6D_6): δ = 0.90 (s, 1H), 1.53 (s, 1H), 1.83 (dt, *J* = 7.9, 14.8 Hz, 1H), 2.17 (psq, *J* = 8.5 Hz, 2H), 2.45 (dq, *J* = 6.6, 13.8 Hz, 1H), 3.88-3.92 (m, 2H), 6.88-6.99 (m, 3H), 7.04 (t, *J* = 7.2 Hz, 1H), 7.08-7.14 (m, 4H), 7.47 (d, *J* = 7.8 Hz, 2H). ^{13}C-NMR (101 MHz, C_6D_6): δ = 28.56, 32.22, 50.94, 81.71, 86.29,

126.98, 127.40, 127.55, 128.19, 128.61, 129.41, 139.22, 142.28. MS (EI, 70 eV): m/z (%) = 254.2 [M]$^+$ (90), 236.2 (7), 177.1 (11), 165.1 (10), 131.1 (93), 105.1 (100), 91.1 (18), 77.1 (32), 43.1 (8). HRMS (APCI) calcd for $C_{17}H_{18}O_2$ [M]$^+$: 254.1301, found: 254.1299. IR (ATR): ν[cm^{-1}] = 3535, 3435, 3058, 3026, 2945, 1709, 1601, 1494, 1445, 1364, 1303, 1270, 1228, 1091, 1047, 1032, 1016, 969, 891, 848, 761, 698, 653, 619, 556, 531.

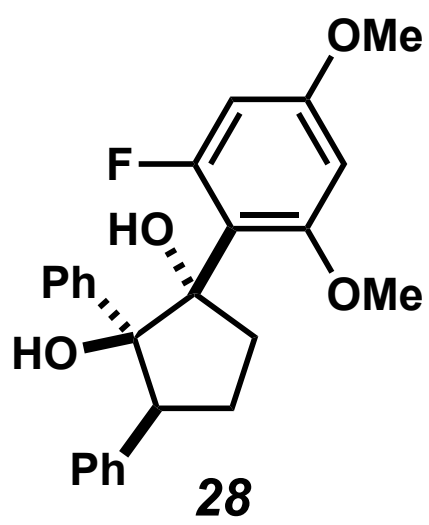

28

***cis,cis*-1-(2-Fluoro-4,6-dimethoxyphenyl)-2,3-diphenylcyclopentane-1,2-diol.** The procedure for the organolanthanide addition published in the rocaglamide synthesis by Zhou and Tius was applied as follows.[36]

In a 25 ml flame-dried, argon-backfilled Schlenk tube equipped with a magnetic stir bar, 1,3-dimethoxy-5-fluorobenzene (0.11 ml, 0.82 mmol, 4.1 equiv) was dissolved in THF (1.8 ml) and the solution was cooled to –78 ºC while being stirred. *n*-Butyllithium (2.47 M in hexane, 0.28 ml, 0.69 mmol, 3.5 equiv) was added dropwise and the mixture was stirred at –78 ºC for 2 h. $LaCl_3$•2LiCl (0.6 M in THF, 1.2 ml, 0.72 mmol, 3.6 equiv) was added dropwise and the mixture was stirred at –78 ºC for another 1.5 h. Next, a solution of *cis*-2-hydroxy-2,3-diphenylcyclopentanone (50.8 mg, 0.2 mmol) in THF (1.2 ml) was added dropwise and the mixture was warmed to –26 ºC over 3 h. The reaction was quenched by addition of sat. aq. NH_4Cl (2 ml). The mixture was filtered through Celite and rinsed with EtOAc. The filtrate was dried (Na_2SO_4), filtered, and concentrated under reduced pressure. Flash chromatography of the crude product (hexanes/EtOAc, 4:1) afforded the desired product in 64% yield as a single diastereomer in form of a colorless solid.

R_f = 0.2 (hexanes/EtOAc, 4:1). ^{1}H-NMR (500.3 MHz, $CDCl_3$): δ = 2.32-2.48 (m, 3H), 2.89-2.96 (m, 1H), 2.99 (br, 3H), 3.78 (s, 3H), 4.22 (br, 1H), 5.67 (br s, 1H), 5.88 (brdd, *J* = 1.6, 2.3 Hz, 1H), 6.33 (dd, *J* = 2.3, 14.7 Hz, 1H), 6.98-7.07 (m, 6H), 7.11 (tt, *J* = 1.4, 7.4 Hz, 2H), 7.27 (dd, *J* = 1.7, 7.2 Hz, 2H). ^{13}C-NMR (125.8 MHz, $CDCl_3$): δ = 29.1 (d, *J* = 7.2 Hz), 37.5 (d, *J* = 10.2 Hz), 52.2, 55.66, 55.73, 86.6, 88.7 (d, *J* = 3.6 Hz), 94.5, 94.7, 95.5 (d, *J* = 2.1 Hz), 111.4 (d, *J* = 14.0 Hz), 126.0, 126.4, 126.6, 127.6, 129.5, 140.2, 142.2, 159.2 (d, *J* = 11.3 Hz), 160.4 (d, *J* = 16.4 Hz), 161.6 (d, *J* = 244.1 Hz). ^{19}F NMR (470.7 MHz, $CDCl_3$): δ = –106.77 (br d, *J* = 6.9 Hz). MS (EI, 70 eV): m/z (%) = 408.2 [M]$^+$ (11), 390.2 (54), 372.2 (32), 286.2 (28), 258.1 (11), 243.1 (18), 211.1 (52), 198.1 (14), 196.1 (14), 195.1 (13), 182.1 (100), 169.1 (61), 155.1 (17), 153.1 (16), 139.1 (25), 129.1 (20), 115.1 (12), 105.1 (55), 91.1 (20), 77.1 (34). HRMS (ESI) calcd for $C_{25}H_{25}O_4FNa^+$ [M+Na]$^+$: 431.16346, found: 431.16306. IR (ATR): ν[cm^{-1}] = 3435, 3087, 3057, 3025, 3001, 2948, 2840, 1622, 1578, 1491, 1456, 1434, 1384, 1340, 1217, 1198, 1141, 1102, 1065, 1001, 939, 734, 698.

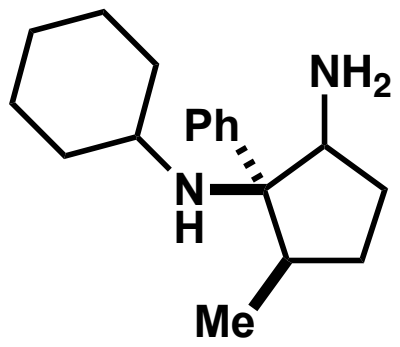

31
(6:1 d.r.)

***N*[1]-Cyclohexyl-5-methyl-1-phenylcyclopentane-1,2-diamine** (presumably *cis/cis* configured). Iminonitrile **29** was submitted to the iminonitrile cyclization conditions as described for the preparation of **24** on a 0.16 mmol scale. After stirring for 24 h, the reaction mixture was allowed to cool to room temperature and $NaBH_3CN$ (60.3 mg, 0.959 mmol, 6.00 equiv) was added in one portion which turned the reaction grass-green. After stirring at room temperature (23 ºC) for 24 h, 1N HCl (5 ml) was added (attention: possible HCN evolution!) and it was vigorously stirred for 25 min. The mixture gradually lost its color, became colorless, and finally slightly yellow. Solid K_2CO_3 was added (pH 11) and stirring was continued for another 15 min. The mixture was extracted with CH_2Cl_2 (6 × 20 ml) and the combined organic extracts were dried (Na_2SO_4), filtered, and concentrated. The crude yellow oil was purified by preparative TLC (CH_2Cl_2/MeOH, 8:1, R_f = 0.24) and then a second time by preparative TLC (CH_2Cl_2/MeOH/Et_3N, 40:5:1) without pretreatment of the TLC plate. The desired diamine was obtained as a 6:1 diastereomeric mixture in form of a colorless oil in 33% yield (14.5 mg, 0.053 mmol). The NMR data is given for the major diastereomer.
^{1}H-NMR (400 MHz, C_6D_6): δ = 0.87-1.41 (m, 6H), 1.26 (d, J = 7.2 Hz, 3H), 1.51-1.69 (m, 4H), 1.74-1.85 (m, 4H), 2.30 (dq, J = 7.5, 7.5 Hz, 1H), 2.37 (tt, J = 3.6, 10.2 Hz, 1H), 2.95 (br, s, 3H), 3.35 (t, J = 7.3 Hz, 1H), 7.09 (t, J = 7.3 Hz, 1H), 7.21 (t, J = 7.6 Hz, 2H), 7.47 (d, J = 8.5 Hz, 2H). ^{13}C-NMR (101 MHz, C_6D_6): δ = 16.48, 25.79, 25.94, 26.35, 29.91, 30.43, 36.25, 37.08, 42.00, 52.80, 61.35, 68.77, 126.54, 127.94, 128.18, 146.47. HRMS (pos. APCI): calcd for $C_{18}H_{28}N_2$ $[M+H]^+$: 273.2325; found 273.2333. MS (EI, 70 eV): *m*/z (%) = 272 $[M]^+$ (62), 229 (25), 228 (100), 189 (62), 188 (99), 173 (39), 172 (80), 158 (33), 146 (81), 134 (63), 132 (26), 131 (28), 129 (31), 117 (28), 115 (32), 106 (37), 104 (78), 91 (30), 56 (66), 55 (30). MS (CI, NH_3): *m*/z (%) = 273 $[M+H]^+$ (100), 174 (28). IR (ATR): ν[cm^{-1}] = 2925, 2852, 1599, 1446, 1264, 1098, 735, 702.

A NOESY experiment showed a strong correlation between the tertiary C*H*-NH_2 and C*H*-CH_3 protons as well as of both of them with the *ortho*-C*H* of the aryl group. This suggests a *cis*/*cis* configuration with the two amino groups and the methyl group on the same side. This would mean that the reduction to diol **26** and diamine **31** occur with opposite stereoselectivity, but further structural proof is required for confirmation before making this assignment. To date, crystallization attempts (also of the TFA salt) were unsuccessful.

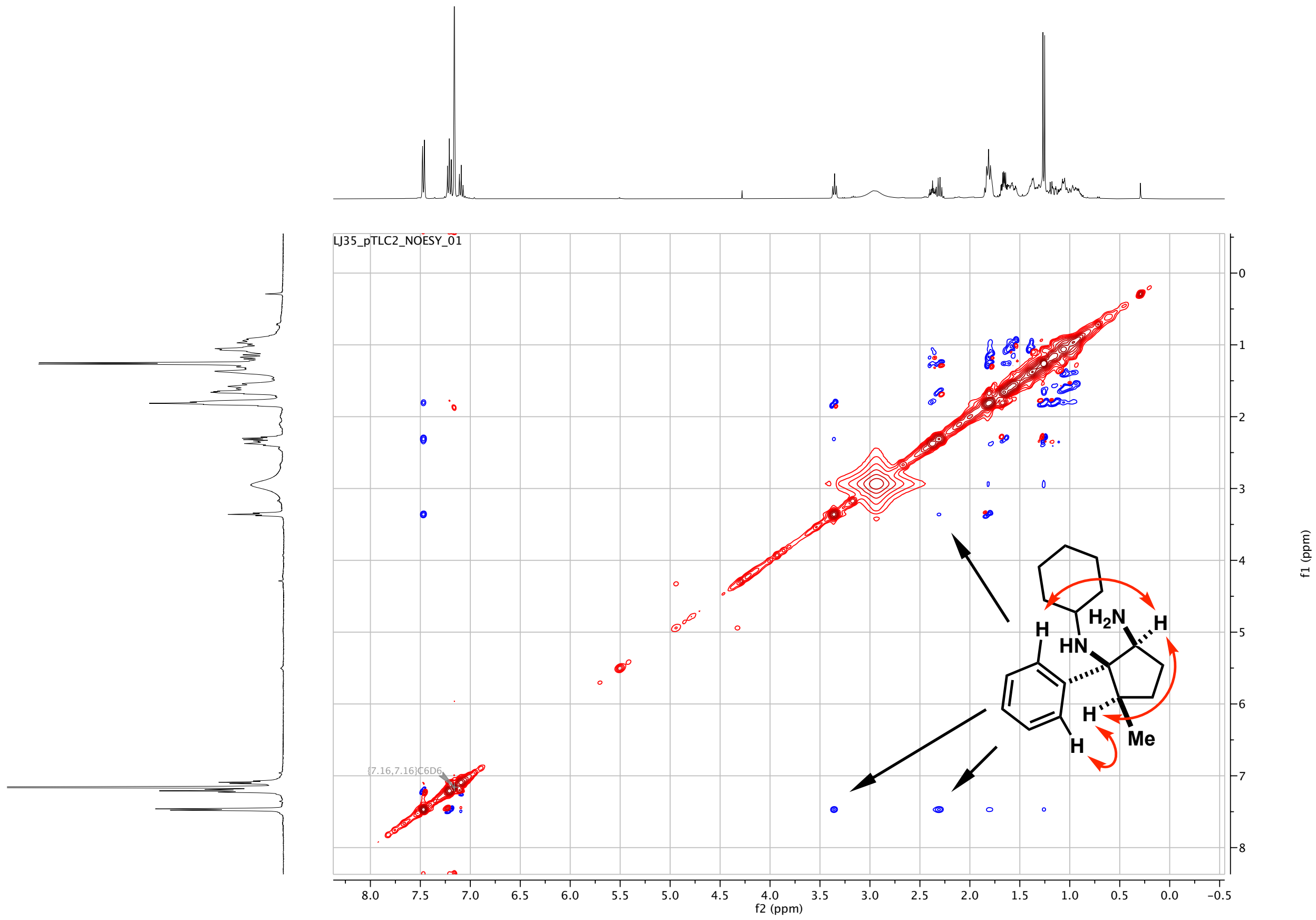

Figure S22. NOESY NMR showing a strong interaction between the tertiary C*H*-NH_2 and C*H*-CH_3 protons as well as of both of them with the *ortho*-C*H* of the aryl group.

Desaturation to a Cyclopentenone

Established protocols for the Saegusa oxidation of **5** or the direct desaturation of **2** using IBX were unsuccessful. Instead, the route via selenoxide pyrolysis proved to be feasible.

a) Attempted Saegusa oxidation:

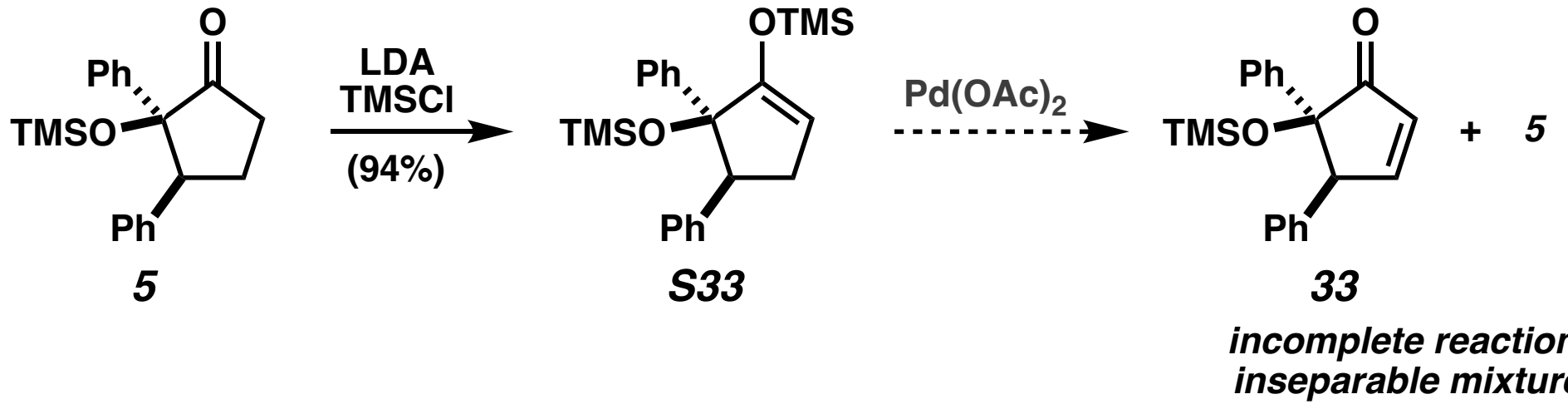

Formation of silyl enol ether **S33** was achieved without problems using LDA and TMSCl at low temperature. The following treatment with $Pd(OAc)_2$ according to a literature procedure by Gademann *et al.* for sterically demanding substrates,[37] however, led to an inseparable mixture of the desired product **33** with precursors **5** and **S33**.

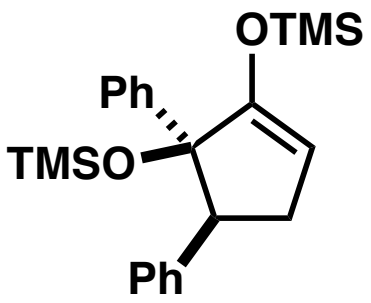

S33

***cis*-Trimethylsilyl 2-trimethylsiloxy-2,3-diphenylcyclopentenol ether.** In a 25 ml flame-dried, argon-backfilled Schlenk tube equipped with a magnetic stir bar, freshly distilled diisopropyl amine (0.16 ml, 1.14 mmol, 1.1 equiv) was dissolved in abs. THF (5 ml). The solution was stirred and cooled to −78 ºC. A solution of *cis*-2-(trimethylsiloxy)-2,3-diphenylcyclopentanone (324 mg, 1.0 mmol) in THF (1 ml) was added dropwise and the mixture was stirred at −78 ºC for 1.5 h. Chlorotrimethylsilane (0.13 ml, 1.02 mmol, 1.0 equiv) was added dropwise and the reaction allowed to cool to room temperature. After stirring for another 3 h, *n*-pentane (6 ml) was added, which led to an immediate formation of a precipitate. The mixture was filtered over celite (rinsed with *n*-pentane) and the filtrate was concentrated to afford the silyl enol ether as a colorless oil in 94% yield (371 mg, 0.94 mmol). ^{1}H-NMR (400.1 MHz, C_6D_6): δ = 0.13 (s, 9H), 0.15 (s, 9H), 2.42 (ddd, J = 2.9, 7.7, 15.1 Hz, 1H), 2.72 (ddd, *J* = 2.1, 7.9, 15.1 Hz, 1H), 3.34 (t, *J* = 7.8 Hz, 1H), 4.91 (dd, *J* = 2.3, 2.7 Hz, 1H), 7.06-7.21 (m, 8H), 7.27-7.31 (m, 2H). ^{13}C-NMR (100.6 MHz, C_6D_6): δ = 0.2, 1.9, 33.0, 59.0, 87.7, 106.0, 126.6, 126.7, 126.8, 127.5, 127.8, 130.4, 139.8, 146.2, 154.8. MS (EI, 70 eV): m/z (%) = 396.2 $[M]^+$ (28), 305.2 (7), 217.1 (7), 215.1 (5), 147 (19), 143 (6), 115 (5), 105 (7), 91.1 (7), 75.1 (14), 73.1 (100), 45 (11). HRMS (ESI) calcd for $C_{23}H_{32}O_2Si_2Na^+$ $[M+Na]^+$: 419.18385, found: 419.18335. IR (ATR): ν [cm^{-1}] = 3087, 3062, 3028, 2957, 2859, 1644, 1447, 1240, 1106, 1074, 1037, 985, 951, 888, 868, 835, 754, 696.

*b) Attempted dehydrogenation of **2** with IBX:*

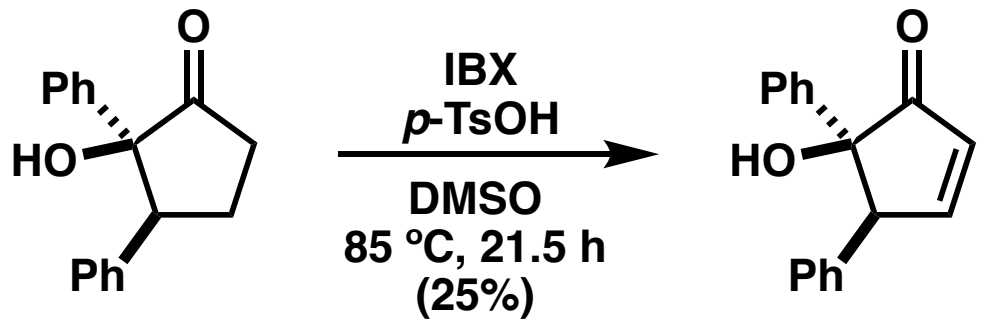

2 ***S34***

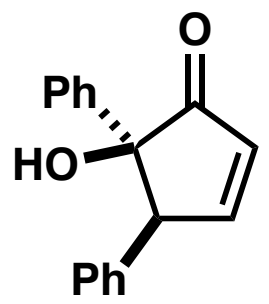

S34

***cis*-2-Hydroxy-2,3-diphenylcyclopenta-4-en-1-one.** A flame-dried 50 ml Schlenk flask equipped with a magnetic stir bar was charged with **2** (253 mg, 1.01 mmol), evacuated, and back-filled with argon. DMSO (13.5 ml) was added and the mixture was stirred at room temperature until the solid had dissolved. Then, 2-iodoxybenzoic acid (IBX, 1.12 g, 4.00 mmol, 4.0 equiv) was added and stirring was continued until a homogeneous solution had formed. The flask was sealed with a greased glass stopper and the reaction mixture was heated (85 ºC oil bath temperature) for 21.5 h. The reaction was allowed to cool down and transferred into a separation funnel. The reaction vessel was rinsed the Et_2O (3 × 4 ml) and aq. $NaHCO_3$ solution (5% v/v, 10 ml). Et_2O (88 ml) and aq. $NaHCO_3$ solution (5% v/v, 30 ml) were added and, after shaking, the layers were separated. The organic layer was subsequently washed with additional aq. $NaHCO_3$ solution (5% v/v, 3 × 40 ml) and with water (40 ml). The combined aqueous layers were extracted with Et_2O (3 × 50 ml). The combined organic layers were dried ($MgSO_4$), filtered, and concentrated under reduced pressure. The residue was crystallized (cyclohexane/Et_2O, 5:1) and the desired product was received as a yellow solid in 25% yield (61.8 mg, 0.25 mmol). R_f = 0.2 (cyclohexane/EtOAc = 5:1). ^{1}H-NMR ($CDCl_3$, 400.1 MHz): δ = 2.44 (br s, 1H), 4.41 (t, J = 2.4 Hz, 1H), 6.63 (dd, J = 2.2, 6.0 Hz, 1H), 7.09 (dd, J = 1.7, 7.6 Hz, 2H), 7.30-7.42 (m, 8H), 7.89 (dd, J = 2.8, 6.0 Hz, 1H). ^{13}C-NMR ($CDCl_3$, 100.6 MHz): δ = 61.3, 79.3, 125.0, 128.0, 128.2, 128.8, 128.9, 129.5, 132.6, 135.7, 142.7, 164.6, 208.6. MS (EI, 70 eV): m/z (%) = 250.1 [M]$^+$ (6), 145.1 (9), 117.1 (11), 115.1 (21), 105 (100), 91.1 (11), 77 (16). HRMS (ESI) calcd for $C_{17}H_{14}O_2Na$ [M+Na]$^+$: 273.08860, found: 273.08884. IR (ATR): ν [cm^{-1}] = 3346, 3060, 3027, 1699, 1578, 1493, 1450, 1336, 1201, 1121, 1069, 853, 798, 751, 697, 665, 616.

c) Successful selenoxide pyrolysis:

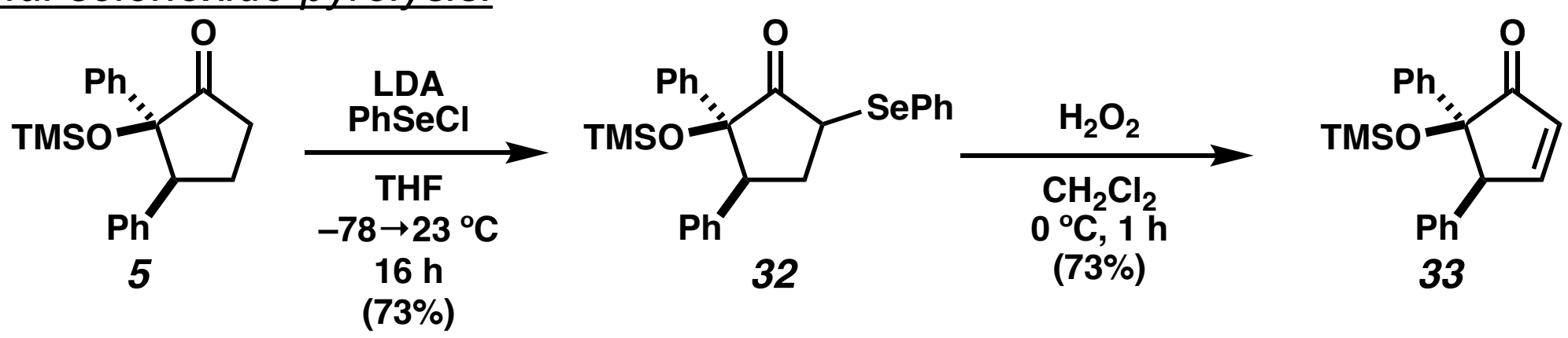

5 ***32*** ***33***

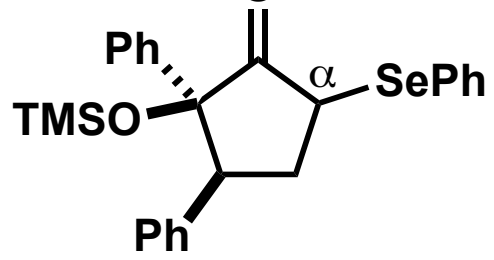

32
(Cα: 6.2:1 d.r.)

***cis*-2,3-Diphenyl-5-(phenylselanyl)-2-((trimethylsilyl)oxy)cyclopentan-1-one**. In a 100 ml flame-dried, argon-backfilled Schlenk tube equipped with a magnetic stir bar, freshly distilled diisopropylamine (0.42 ml, 2.99 mmol, 1.5 equiv) was dissolved in abs. THF (10 ml) and the solution was cooled to −78 ºC while stirred. *n*-Butyllithium (2.47 M in hexane, 1.2 ml, 2.96 mmol, 1.5 equiv) was added dropwise, the mixture was stirred for 15 min without cooling and then cooled again to

−78 °C. A solution of *cis*-2-(trimethylsiloxy)-2,3-diphenylcyclopentanone (653 mg, 2.01 mmol) in THF (2.3 ml) was added dropwise. The mixture was stirred for 16 h and allowed to warm to room temperature during that time. The reaction was quenched by addition of sat. aq. NH_4Cl solution (20 ml) and the mixture stirred for 10 min at room temperature. The mixture was transferred into a separation funnel (rinsed with 2 × 6 ml EtOAc). Water (50 ml) and EtOAc (40 ml) were added and, after shaking, the layers were separated. The aqueous layer was extracted with EtOAc (2 × 50 ml) and the combined organic layers were washed with brine (50 ml), dried (Na_2SO_4), filtered, and concentrated. The crude product was purified by flash chromatography (cyclohexane/EtOAc, 50:1) to afford the desired product as a 6.2:1 mixture of α-diastereomers (not assigned) in 73% combined yield (707 mg, 1.47 mmol) in form of an orange oil. R_f = 0.3 (cyclohexane/EtOAc, 40:1). The ^{1}H-NMR data is given for the diastereomeric mixture. ^{1}H-NMR (500.3 MHz, $CDCl_3$): δ = −0.05 (s, 9H, major), −0.04 (s, 9H, minor), 2.16 (ddd, J = 0.8, 5.7, 13.7 Hz, 1H, major), 2.36 (ddd, J = 0.9, 5.7, 14.3 Hz, 1H, minor), 2.89-3.03 (m, 1H, both diast.), 3.35 (dd, J=5.6, 12.9Hz, 1H, major), 3.49 (dd, J=5.7, 13.0 Hz, 1H, minor), 4.28 (dt, J = 0.5, 7.0 Hz, 1H; major), 4.57 (dt, J = 0.7, 6.4 Hz, 1H, minor), 6.92-7.05 (m, 4H, both diast.), 7.16-7.41 (m, 9H, both diast.), 7.59-7.64 (m, 2H, minor), 7.71-7.76 (m, 2H, major), 7.86-7.88 (m, 2H, minor). ^{13}C-NMR (125.8 MHz, $CDCl_3$, only major diastereomer): δ = 1.3, 32.7, 45.3, 56.3, 84.0, 126.8, 127.2, 127.4, 127.6, 129.0, 129.5, 130.1, 135.6, 136.4, 137.1, 137.2, 140.4, 210.3. MS (EI, 70 eV): m/z (%) = 480.2 $[M]^+$ (2), 478.2 $[M]^+$ (1), 323.2 (49), 281.2 (11), 268.1 (100), 236.2 (15), 233.1 (17), 205.1 (10), 178.1 (11), 118.1 (6), 115 (6), 105 (56), 91.1 (7), 77.1 (8), 75.1 (8), 73.1 (23), 55.1 (7). HRMS (ESI) calcd for $C_{26}H_{28}O_2NaSeSi$ $[M+Na]^+$: 503.09215, found: 503.09167. IR (ATR): ν[cm^{-1}] = 3060, 3029, 2956, 2897, 1735, 1438, 1250, 1101, 1008, 919, 873, 846, 760, 741, 698.

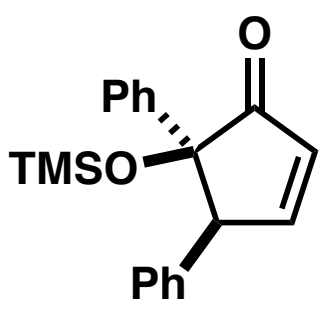

33

***cis*-4,5-Diphenyl-5-((trimethylsilyl)oxy)cyclopent-2-en-1-one.** To a 10 ml flame-dried, argon-backfilled Schlenk tube equipped with a magnetic stir bar was added a solution of phenylselanylcyclopentenone **32** (48.6 mg, 0.1 mmol) in CH_2Cl_2 (0.3 ml). The solution was stirred and cooled to 0 °C. Aqueous 30% hydrogen peroxide solution (10 µl, 0.1 mmol, 1.0 equiv) was added and the biphasic mixture stirred at 0 °C. The reaction progress was followed by TLC. After 40 min, additional aq. hydrogen peroxide solution (10 µl, 0.1 mmol, 1.0 equiv) was added and stirring was continued at 0 °C for another 20 min. Aq. sat. $NaHCO_3$ solution (2 ml) was added and the excess peroxide quenched by addition of Na_2SO_3 (13.4 mg, 0.11 mmol, 1.1 equiv) and stirring for 40 min. The mixture was transferred into a separation funnel (rinsing with 2 × 4 ml EtOAc). EtOAc (2 ml) and water (15 ml) were added. After shaking, the layers were separated and the aqueous layer extracted with EtOAc (2 × 15 ml). The combined organic layers were washed with brine (15 ml), dried (Na_2SO_4), filtered, and the solvent was removed under reduced pressure. The crude residue was purified by flash chromatography (cyclohexane/EtOAc, 30:1) and the desired product obtained as a colorless oil in 73% yield (24 mg, 0.07 mmol). R_f = 0.2 (cyclohexane/EtOAc, 30:1). ^{1}H-NMR (400.1 MHz, $CDCl_3$): δ = −0.20 (s, 9H), 4.25 (t, *J* = 2.5 Hz, 1H), 6.56 (dd, *J* = 2.2, 6.1 Hz, 1H), 7.01-7.03 (m, 2H), 7.26-7.36 (m, 8H), 7.80 (dd, *J* = 2.7, 6.1 Hz, 1H). ^{13}C-NMR (100.6 MHz, $CDCl_3$): δ = 1.5, 62.7, 82.4, 125.5, 127.3, 127.5, 128.1, 128.4, 129.7, 133.2, 137.3, 144.3, 164.1, 208.4. MS (EI, 70 eV): m/z (%) = 322.1 [M]+ (47), 307.1 (100), 294.1 (18), 293.1 (12), 203.1 (12), 201.1 (13), 105 (13), 73 (42). HRMS (APCI) calcd for $C_{20}H_{26}O_2NSi^+$ $[M+NH_4]^+$: 340.17273, found: 340.17245.

Attempted Kinetic Resolution Using a Chiral Catalyst

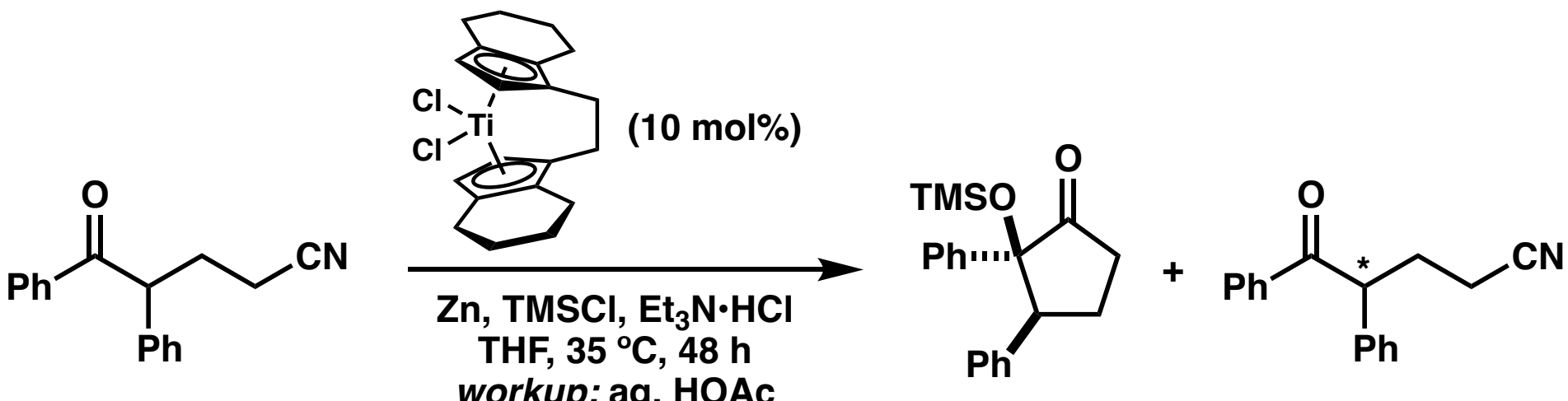

2 (rac)

5 *44% ee* *33%, >98:2 d.r.*

2 *26% ee* *47%*

A flame-dried argon-backfilled 10 ml Schlenk tube equipped with a magnetic stir bar was charged with (*R*,*R*)-ethylenebis(4,5,6,7-tetrahydro-1-indenyl)titanium(IV) dichloride (7.6 mg, 0.02 mmol, 10 mol%), triethylamine hydrochloride (55.7 mg, 0.40 mmol, 2.0 equiv), and zinc dust (28.2 mg, 0.43 mmol, 2.2 equiv). The tube was evacuated while stirring was started and after 1 minute back-filled with argon. THF (0.5 ml) was added and the mixture was stirred at room temperature (23 ºC) until a color change occurred from red to green (about 1 min). 5-Oxo-4,5-diphenylpentanenitrile (49.8 mg, 0.20 mmol) and chlorotrimethylsilane (76 µL, 65 mg, 0.6 mmol, 3.0 equiv) were successively added and the reaction vessel sealed with a greased glass stopper. The mixture was stirred for 48 h at 35 ºC. The reaction was quenched by addition of H_2O (2 ml) and then EtOAc (2 ml) and AcOH (4 drops, approx. 4 × 50 µl) were added. The mixture was stirred for another 3 h at room temperature and then transferred into a separatory funnel using EtOAc (2 × 4 ml) for rinsing. EtOAc (2 ml) and water (10 ml) were added and, after shaking, the two layers were separated. The aqueous layer was extracted with EtOAc (3 × 15 ml) and the combined organic layers dried (Na_2SO_4), filtered, and concentrated under reduced pressure. The residue was purified by flash chromatography (cyclohexane:EtOAc = 25:1→10:1). The cyclized product and the remaining ketonitrile were isolated in pure form in 33% (21.1 mg, 0.07 mmol) and 47% (23.3 mg, 0.09 mmol), respectively.

Determination of enantiomeric excess[38]

5: 44% *ee*. Chiral HPLC. Daicel Chiracel OD-3, 150×4.6 mm, *n*-Hept:*i*-PrOH = 500:1, 1.0 mL/min, 215 nm, 22 °C. t_{major} = 5.0 min, t_{minor} = 6.8 min.

1: 26% *ee*. Chiral HPLC. Phenomenex Lux 3u Amylose-2, 150×4.6 mm, *n*-Hept:EtOH = 95:5, 1.0 mL/min, 247 nm, 22°C. t_{major} = 33.0 min, t_{minor} = 30.3 min.

X-Ray Report for 25

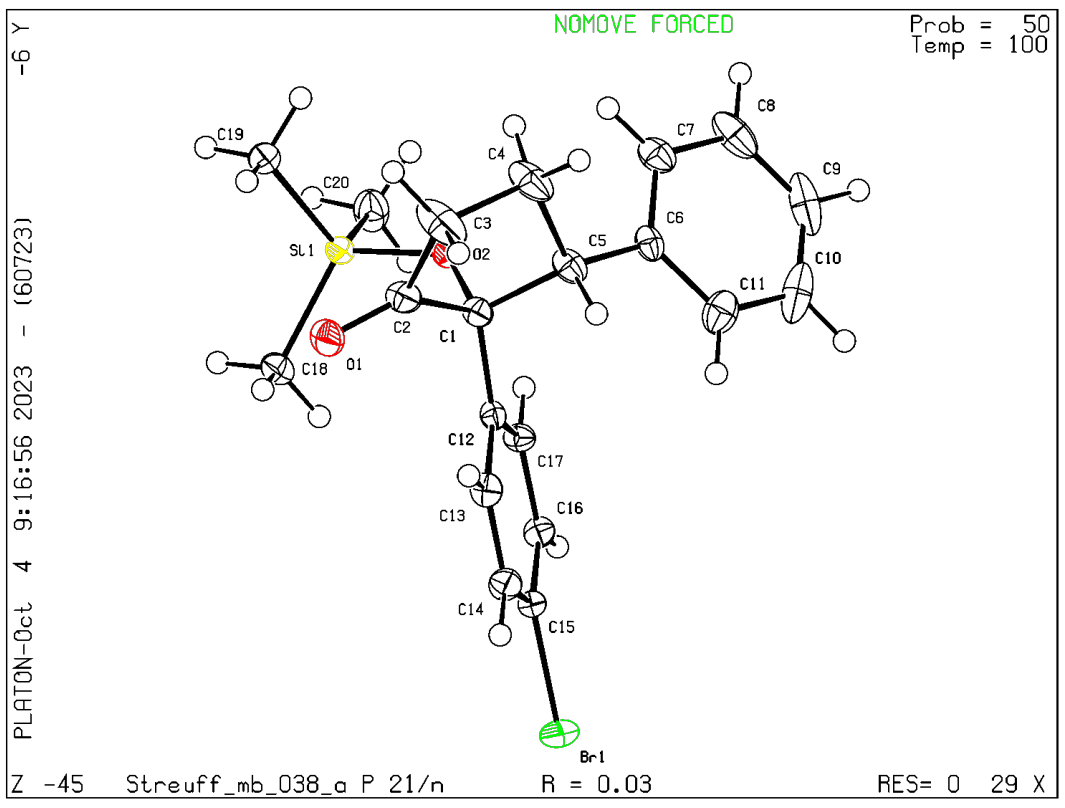

A colourless, block-shaped crystal of Streuff_mb_038_a was mounted on a MiTeGen micromount with perfluoroether oil. Data were collected from a shock-cooled single crystal at 100(2) K on a Bruker APEX2 QUAZAR three-circle diffractometer with a microfocus sealed X-ray tube using a mirror optics as monochromator and a Bruker APEXII detector. The diffractometer was equipped with an Oxford Cryostream 800 low temperature device and used MoK_{α} radiation (λ = 0.71073 Å). All data were integrated with SAINT and a multi-scan absorption correction using SADABS was applied.[1,2] The structure was solved by direct methods using SHELXT and refined by full-matrix least-squares methods against F^2 by SHELXL-2019/2.[3,4] All non-hydrogen atoms were refined with anisotropic displacement parameters. All hydrogen atoms were refined isotropic on calculated positions using a riding model with their U_{iso} values constrained to 1.5 times the U_{eq} of their pivot atoms for terminal sp^3 carbon atoms and 1.2 times for all other carbon atoms. Crystallographic data for the structures reported here have been deposited with the Cambridge Crystallographic Data Centre.[5] CCDC 2299133 contains the supplementary crystallographic data for this paper. These data can be obtained free of charge from The Cambridge Crystallographic Data Centre via www.ccdc.cam.ac.uk/structures. This report and the CIF file were generated using FinalCif.[6]

Table S8. Crystal data and structure refinement for Streuff_mb_038_a.

CCDC number	2299133
Empirical formula	$C_{20}H_{23}BrO_2Si$
Formula weight	403.38
Temperature [K]	100(2)
Crystal system	monoclinic
Space group (number)	$P2_1/n$ (14)
a [Å]	10.329(3)
b [Å]	8.954(2)
c [Å]	20.632(7)
α [°]	90
β [°]	96.829(10)
γ [°]	90
Volume [Å^3]	1894.5(9)
Z	4
ρ_{calc} [gcm^{-3}]	1.414
μ [mm^{-1}]	2.242
F(000)	832
Crystal size [mm^3]	0.30×0.27×0.20
Crystal colour	colourless
Crystal shape	block
Radiation	MoK_{α} (λ=0.71073 Å)
2θ range [°]	3.98 to 61.13 (0.70 Å)
Index ranges	$-14 \le h \le 14$<br>$-12 \le k \le 12$<br>$-28 \le l \le 25$
Reflections collected	27571
Independent reflections	5417<br>R_{int} = 0.0200<br>R_{sigma} = 0.0192
Completeness to θ = 25.242°	100.0 %
Data / Restraints / Parameters	5417/0/220
Goodness-of-fit on F^2	1.049
Final R indexes [$I \geq 2\sigma(I)$]	R_1 = 0.0276<br>wR_2 = 0.0688
Final R indexes [all data]	R_1 = 0.0345<br>wR_2 = 0.0710
Largest peak/hole [eÅ^{-3}]	0.49/-0.60

Table S9. Atomic coordinates and U_{eq} [Å^2] for Streuff_mb_038_a.

Atom	*x*	*y*	*z*	U_{eq}
Br1	0.14031(2)	0.12837(2)	0.48186(2)	0.02838(6)
Si1	0.42707(3)	0.80322(4)	0.64633(2)	0.01894(8)
O1	0.32759(10)	0.56900(13)	0.75615(5)	0.0265(2)
O2	0.52005(9)	0.65012(10)	0.65847(5)	0.01836(19)
C1	0.48985(12)	0.50725(15)	0.68292(6)	0.0178(2)
C2	0.43768(13)	0.53320(17)	0.74971(7)	0.0230(3)
C3	0.54656(16)	0.5105(3)	0.80436(8)	0.0444(5)
H3A	0.531586	0.419384	0.829588	0.053
H3B	0.553683	0.597283	0.834325	0.053
C4	0.66963(15)	0.4947(2)	0.77040(8)	0.0359(4)
H4A	0.734425	0.429422	0.795771	0.043
H4B	0.709840	0.593321	0.764357	0.043
C5	0.61970(13)	0.42362(17)	0.70430(7)	0.0221(3)
H5	0.595873	0.318147	0.713560	0.027
C6	0.71485(12)	0.41648(16)	0.65424(7)	0.0193(3)
C7	0.81014(13)	0.52438(18)	0.64963(7)	0.0252(3)
H7	0.813028	0.610081	0.676910	0.030
C8	0.90109(15)	0.5086(2)	0.60571(8)	0.0352(4)
H8	0.967422	0.581560	0.604193	0.042
C9	0.89549(18)	0.3869(2)	0.56411(9)	0.0410(5)
H9	0.957726	0.376185	0.534032	0.049
C10	0.7992(2)	0.2814(2)	0.56659(9)	0.0416(4)
H10	0.793655	0.199030	0.537363	0.050
C11	0.70992(17)	0.29496(17)	0.61172(8)	0.0299(3)
H11	0.644874	0.220587	0.613603	0.036
C12	0.39995(12)	0.41980(14)	0.63381(6)	0.0156(2)
C13	0.31910(13)	0.30836(15)	0.65397(7)	0.0197(3)
H13	0.317314	0.290921	0.699276	0.024
C14	0.24138(13)	0.22280(15)	0.60882(7)	0.0219(3)
H14	0.185920	0.147785	0.622853	0.026
C15	0.24584(12)	0.24832(14)	0.54308(7)	0.0183(3)
C16	0.32462(13)	0.35799(14)	0.52120(7)	0.0183(3)
H16	0.326205	0.374539	0.475818	0.022
C17	0.40144(12)	0.44342(14)	0.56723(6)	0.0177(2)
H17	0.455856	0.519197	0.552942	0.021
C18	0.25109(13)	0.76101(17)	0.62297(8)	0.0247(3)
H18A	0.202853	0.854513	0.614228	0.037
H18B	0.216762	0.707665	0.658728	0.037
H18C	0.241096	0.698630	0.583661	0.037
C19	0.45061(14)	0.91758(18)	0.72189(8)	0.0296(3)
H19A	0.544064	0.931989	0.735146	0.044
H19B	0.411681	0.866161	0.756768	0.044
H19C	0.408513	1.014935	0.713621	0.044
C20	0.49512(16)	0.90022(17)	0.57872(9)	0.0305(3)
H20A	0.477748	0.840990	0.538685	0.046
H20B	0.589460	0.912408	0.589747	0.046
H20C	0.454178	0.998608	0.571927	0.046

U_{eq} is defined as 1/3 of the trace of the orthogonalized U_{ij} tensor.

Table S10. Anisotropic displacement parameters [Å^2] for Streuff_mb_038_a.
The anisotropic displacement factor exponent takes the form: $-2\pi^2[h^2(a^*)^2U_{11} + k^2(b^*)^2U_{22} + \ldots + 2hka^*b^*U_{12}]$

Atom	U_{11}	U_{22}	U_{33}	U_{23}	U_{13}	U_{12}
Br1	0.02611(8)	0.02633(8)	0.03165(10)	−0.00951(6)	−0.00091(6)	−0.00747(5)
Si1	0.01467(16)	0.02105(17)	0.02127(19)	−0.00529(14)	0.00282(14)	−0.00126(13)
O1	0.0228(5)	0.0362(6)	0.0219(5)	−0.0060(4)	0.0078(4)	−0.0005(4)
O2	0.0146(4)	0.0208(4)	0.0200(5)	−0.0036(4)	0.0034(4)	−0.0016(3)
C1	0.0163(6)	0.0236(6)	0.0137(6)	−0.0009(5)	0.0028(5)	−0.0009(5)
C2	0.0207(6)	0.0333(7)	0.0153(6)	−0.0048(6)	0.0037(5)	−0.0040(5)
C3	0.0228(7)	0.0959(16)	0.0144(7)	−0.0057(9)	0.0014(6)	0.0004(9)
C4	0.0193(7)	0.0711(12)	0.0168(7)	−0.0049(7)	−0.0006(6)	−0.0001(7)
C5	0.0177(6)	0.0319(7)	0.0163(7)	0.0037(6)	0.0002(5)	0.0019(5)
C6	0.0169(6)	0.0259(6)	0.0147(6)	0.0032(5)	0.0001(5)	0.0066(5)
C7	0.0201(6)	0.0354(7)	0.0192(7)	0.0023(6)	−0.0013(5)	0.0006(6)
C8	0.0195(7)	0.0589(11)	0.0272(8)	0.0164(8)	0.0026(6)	0.0040(7)
C9	0.0364(9)	0.0614(12)	0.0276(9)	0.0163(8)	0.0146(7)	0.0296(8)
C10	0.0630(12)	0.0365(9)	0.0265(9)	0.0011(7)	0.0106(8)	0.0281(9)
C11	0.0391(8)	0.0240(7)	0.0264(8)	0.0016(6)	0.0030(7)	0.0070(6)
C12	0.0143(5)	0.0178(5)	0.0149(6)	−0.0003(5)	0.0027(5)	0.0021(4)
C13	0.0226(6)	0.0212(6)	0.0161(6)	0.0026(5)	0.0052(5)	0.0002(5)
C14	0.0221(6)	0.0195(6)	0.0255(7)	−0.0001(5)	0.0083(6)	−0.0038(5)
C15	0.0155(5)	0.0174(5)	0.0216(7)	−0.0046(5)	0.0003(5)	−0.0002(4)
C16	0.0200(6)	0.0207(6)	0.0142(6)	−0.0004(5)	0.0015(5)	0.0003(5)
C17	0.0178(6)	0.0192(6)	0.0162(6)	0.0001(5)	0.0027(5)	−0.0031(5)
C18	0.0173(6)	0.0293(7)	0.0268(8)	−0.0038(6)	−0.0004(5)	0.0007(5)
C19	0.0202(6)	0.0334(7)	0.0351(9)	−0.0166(7)	0.0026(6)	−0.0009(6)
C20	0.0296(8)	0.0268(7)	0.0369(9)	0.0035(6)	0.0112(7)	0.0022(6)

Table S11. Bond lengths and angles for Streuff_mb_038_a.

Atom–Atom	Length [Å]
Br1–C15	1.8999(13)
Si1–O2	1.6754(10)
Si1–C20	1.8514(17)
Si1–C19	1.8567(16)
Si1–C18	1.8629(15)
O1–C2	1.2038(17)
O2–C1	1.4232(16)
C1–C12	1.5094(18)
C1–C5	1.5535(18)
C1–C2	1.5557(19)
C2–C3	1.509(2)
C3–C4	1.529(2)
C3–H3A	0.9900
C3–H3B	0.9900
C4–C5	1.537(2)
C4–H4A	0.9900
C4–H4B	0.9900
C5–C6	1.509(2)
C5–H5	1.0000
C6–C7	1.390(2)
C6–C11	1.395(2)
C7–C8	1.388(2)
C7–H7	0.9500
C8–C9	1.384(3)
C8–H8	0.9500
C9–C10	1.377(3)
C9–H9	0.9500
C10–C11	1.391(3)
C10–H10	0.9500
C11–H11	0.9500
C12–C17	1.3918(19)
C12–C13	1.3960(18)
C13–C14	1.386(2)
C13–H13	0.9500
C14–C15	1.382(2)
C14–H14	0.9500
C15–C16	1.3848(19)
C16–C17	1.3918(18)
C16–H16	0.9500
C17–H17	0.9500
C18–H18A	0.9800
C18–H18B	0.9800
C18–H18C	0.9800
C19–H19A	0.9800
C19–H19B	0.9800
C19–H19C	0.9800
C20–H20A	0.9800
C20–H20B	0.9800
C20–H20C	0.9800

Atom–Atom–Atom	Angle [°]
O2–Si1–C20	103.55(6)
O2–Si1–C19	108.07(7)
C20–Si1–C19	110.51(8)
O2–Si1–C18	113.39(6)
C20–Si1–C18	110.27(8)
C19–Si1–C18	110.82(7)
C1–O2–Si1	129.98(8)
O2–C1–C12	111.82(11)
O2–C1–C5	108.41(10)
C12–C1–C5	112.46(11)
O2–C1–C2	107.06(11)
C12–C1–C2	114.95(11)
C5–C1–C2	101.46(11)
O1–C2–C3	125.68(13)
O1–C2–C1	124.66(13)
C3–C2–C1	109.64(12)
C2–C3–C4	104.86(13)
C2–C3–H3A	110.8
C4–C3–H3A	110.8
C2–C3–H3B	110.8
C4–C3–H3B	110.8
H3A–C3–H3B	108.9
C3–C4–C5	103.61(12)
C3–C4–H4A	111.0
C5–C4–H4A	111.0
C3–C4–H4B	111.0
C5–C4–H4B	111.0
H4A–C4–H4B	109.0
C6–C5–C4	116.58(12)
C6–C5–C1	115.87(11)
C4–C5–C1	103.87(12)
C6–C5–H5	106.6
C4–C5–H5	106.6
C1–C5–H5	106.6
C7–C6–C11	118.18(14)
C7–C6–C5	122.76(13)
C11–C6–C5	119.04(14)
C8–C7–C6	120.89(15)
C8–C7–H7	119.6
C6–C7–H7	119.6
C9–C8–C7	120.24(17)
C9–C8–H8	119.9
C7–C8–H8	119.9
C10–C9–C8	119.61(16)
C10–C9–H9	120.2
C8–C9–H9	120.2
C9–C10–C11	120.26(17)
C9–C10–H10	119.9
C11–C10–H10	119.9
C10–C11–C6	120.76(16)
C10–C11–H11	119.6
C6–C11–H11	119.6
C17–C12–C13	118.69(12)
C17–C12–C1	120.37(11)
C13–C12–C1	120.85(12)
C14–C13–C12	120.92(13)
C14–C13–H13	119.5
C12–C13–H13	119.5
C15–C14–C13	118.96(12)
C15–C14–H14	120.5
C13–C14–H14	120.5
C14–C15–C16	121.79(12)
C14–C15–Br1	118.41(10)
C16–C15–Br1	119.80(10)
C15–C16–C17	118.47(12)
C15–C16–H16	120.8
C17–C16–H16	120.8
C12–C17–C16	121.17(12)
C12–C17–H17	119.4
C16–C17–H17	119.4
Si1–C18–H18A	109.5
Si1–C18–H18B	109.5
H18A–C18–H18B	109.5
Si1–C18–H18C	109.5
H18A–C18–H18C	109.5
H18B–C18–H18C	109.5
Si1–C19–H19A	109.5
Si1–C19–H19B	109.5
H19A–C19–H19B	109.5
Si1–C19–H19C	109.5
H19A–C19–H19C	109.5
H19B–C19–H19C	109.5
Si1–C20–H20A	109.5
Si1–C20–H20B	109.5
H20A–C20–H20B	109.5
Si1–C20–H20C	109.5
H20A–C20–H20C	109.5
H20B–C20–H20C	109.5

Table S12. Torsion angles for Streuff_mb_038_a.

Atom–Atom–Atom–Atom	Torsion Angle [°]
C20–Si1–O2–C1	−151.88(11)
C19–Si1–O2–C1	90.88(12)
C18–Si1–O2–C1	−32.39(13)
Si1–O2–C1–C12	72.78(14)
Si1–O2–C1–C5	−162.69(9)
Si1–O2–C1–C2	−53.96(14)
O2–C1–C2–O1	81.41(17)
C12–C1–C2–O1	−43.4(2)
C5–C1–C2–O1	−165.06(15)
O2–C1–C2–C3	−97.14(15)
C12–C1–C2–C3	138.00(15)
C5–C1–C2–C3	16.39(17)
O1–C2–C3–C4	−169.99(17)
C1–C2–C3–C4	8.5(2)
C2–C3–C4–C5	−30.5(2)
C3–C4–C5–C6	170.19(15)
C3–C4–C5–C1	41.43(17)

O2–C1–C5–C6	−51.60(15)	C5–C6–C11–C10	−177.50(14)
C12–C1–C5–C6	72.55(15)	O2–C1–C12–C17	28.65(16)
C2–C1–C5–C6	−164.12(12)	C5–C1–C12–C17	−93.59(14)
O2–C1–C5–C4	77.59(14)	C2–C1–C12–C17	150.98(12)
C12–C1–C5–C4	−158.26(12)	O2–C1–C12–C13	−154.87(11)
C2–C1–C5–C4	−34.92(14)	C5–C1–C12–C13	82.88(15)
C4–C5–C6–C7	−32.4(2)	C2–C1–C12–C13	−32.55(17)
C1–C5–C6–C7	90.28(16)	C17–C12–C13–C14	−0.16(19)
C4–C5–C6–C11	145.83(15)	C1–C12–C13–C14	−176.69(12)
C1–C5–C6–C11	−91.45(16)	C12–C13–C14–C15	0.6(2)
C11–C6–C7–C8	−2.5(2)	C13–C14–C15–C16	−0.7(2)
C5–C6–C7–C8	175.76(13)	C13–C14–C15–Br1	179.23(10)
C6–C7–C8–C9	2.2(2)	C14–C15–C16–C17	0.4(2)
C7–C8–C9–C10	−0.1(2)	Br1–C15–C16–C17	−179.60(10)
C8–C9–C10–C11	−1.6(2)	C13–C12–C17–C16	−0.22(19)
C9–C10–C11–C6	1.2(2)	C1–C12–C17–C16	176.33(12)
C7–C6–C11–C10	0.9(2)	C15–C16–C17–C12	0.1(2)

Bibliography (X-ray report)

[1] Bruker, *SAINT*, *V8.40B*, Bruker AXS Inc., Madison, Wisconsin, USA.

[2] L. Krause, R. Herbst-Irmer, G. M. Sheldrick, D. Stalke, *J. Appl. Cryst.* **2015**, *48*, 3–10, doi:10.1107/S1600576714022985.

[3] G. M. Sheldrick, *Acta Cryst.* **2015**, *A71*, 3–8, doi:10.1107/S2053273314026370.

[4] G. M. Sheldrick, *Acta Cryst.* **2015**, *C71*, 3–8, doi:10.1107/S2053229614024218.

[5] C. R. Groom, I. J. Bruno, M. P. Lightfoot, S. C. Ward, *Acta Cryst.* **2016**, *B72*, 171–179, doi:10.1107/S2052520616003954.

[6] D. Kratzert, *FinalCif*, *V125*, https://dkratzert.de/finalcif.html.

X-Ray Report for 26

Table S13. Crystal data and structure refinement for StC_BMC_RS212_autored.

Identification code	StC_BMC_RS212_autored
CCDC number	2545933
Empirical formula	$C_{17}H_{18}O_2$
Formula weight	254.31
Temperature/K	120.00(10)
Crystal system	triclinic
Space group	P-1
a/Å	9.17590(10)
b/Å	11.9105(2)
c/Å	12.2034(2)
α/°	93.1670(10)
β/°	90.2170(10)
γ/°	91.3510(10)
Volume/Å^3	1331.28(3)
Z	4
ρ_{calc}g/cm^3	1.269
μ/mm^{-1}	0.645
F(000)	544.0
Crystal size/mm^3	0.92 × 0.14 × 0.11
Radiation	Cu Kα (λ = 1.54184)
2Θ range for data collection/°	7.256 to 153.734
Index ranges	-11 ≤ h ≤ 11, -15 ≤ k ≤ 15, -15 ≤ l ≤ 15
Reflections collected	51439
Independent reflections	5510 [R_{int} = 0.0235, R_{sigma} = 0.0096]
Data/restraints/parameters	5510/0/347
Goodness-of-fit on F^2	1.049
Final R indexes [I>=2σ (I)]	R_1 = 0.0364, wR_2 = 0.0889
Final R indexes [all data]	R_1 = 0.0369, wR_2 = 0.0892
Largest diff. peak/hole / e Å^{-3}	0.31/-0.24

Table S14. Fractional Atomic Coordinates (×10^4) and Equivalent Isotropic Displacement Parameters (Å^2×10^3) for StC_BMC_RS212_autored. U_{eq} is defined as 1/3 of the trace of the orthogonalised U_{IJ} tensor.

Atom	*x*	*y*	*z*	U(eq)
O18	2686.1(8)	3733.1(6)	1020.5(6)	18.47(15)
O37	2467.8(8)	8789.6(6)	1073.0(6)	19.08(16)
O38	2722.5(8)	5953.3(6)	1966.8(6)	20.70(16)
O19	2355.2(8)	976.1(6)	2119.2(6)	21.14(16)
C1	1663.8(11)	3296.7(8)	2778.6(8)	16.07(19)
C7	4404.1(11)	2913.6(8)	2284.5(8)	17.8(2)
C8	2782.2(11)	2996.0(8)	1896.3(8)	15.32(19)
C12	5185.4(10)	4048.6(8)	2377.7(8)	16.7(2)
C20	3282.4(11)	8232.1(8)	2853.5(9)	18.3(2)
C27	2279.0(11)	7968.9(8)	1870.2(8)	15.59(19)
C26	621.9(11)	7786.2(8)	2127.7(8)	17.8(2)
C31	-151.0(10)	8893.1(9)	2279.3(8)	17.4(2)
C13	5297.1(11)	4652.6(9)	3388.8(8)	20.2(2)

C17	5752.6(11)	4552.9(9)	1456.9(8)	18.9(2)
C28	2667.1(11)	6855.4(8)	1242.7(8)	17.9(2)
C36	-702.1(11)	9448.9(9)	1395.3(9)	19.9(2)
C16	6406.1(11)	5619.0(9)	1542.1(9)	20.9(2)
C9	2473.7(11)	1820.1(8)	1332.9(8)	18.5(2)
C15	6502.5(11)	6209.6(9)	2555.6(9)	21.6(2)
C2	393.0(12)	3824.1(9)	2469.3(9)	22.3(2)
C14	5945.9(12)	5724.3(9)	3478.2(9)	22.6(2)
C35	-1325.0(12)	10500.9(9)	1542.2(9)	22.8(2)
C6	1801.1(12)	3019.9(9)	3867.3(9)	21.5(2)
C34	-1389.5(12)	11027.8(9)	2582.8(10)	23.8(2)
C33	-850.8(12)	10488.2(10)	3472.2(9)	25.5(2)
C32	-250.8(11)	9428.7(10)	3321.2(9)	22.6(2)
C30	46.6(12)	6943.0(9)	1196.8(10)	23.8(2)
C11	5080.8(12)	2017.3(9)	1467.9(10)	25.4(2)
C29	1368.8(12)	6619.2(9)	484.9(9)	22.9(2)
C10	3851.7(13)	1596.1(9)	678.6(9)	25.0(2)
C25	2994.0(13)	7861.4(10)	3895.3(9)	25.7(2)
C21	4594.2(12)	8817.9(9)	2698.1(11)	27.2(2)
C5	712.8(14)	3283.6(10)	4627.8(10)	28.1(2)
C4	-532.4(13)	3814.9(10)	4305.3(11)	30.1(3)
C3	-691.6(12)	4078.1(10)	3225.1(11)	28.9(3)
C24	3968.8(15)	8089.9(12)	4761.8(10)	36.0(3)
C23	5253.8(16)	8679.5(11)	4594.1(12)	41.2(4)
C22	5567.6(15)	9034.6(10)	3562.0(13)	39.2(3)

Table S15. Anisotropic Displacement Parameters (Å^2×10^3) for StC_BMC_RS212_autored. The Anisotropic displacement factor exponent takes the form: $-2\pi^2[h^2a^{*2}U_{11}+2hka^*b^*U_{12}+\ldots]$.

Atom	U_{11}	U_{22}	U_{33}	U_{23}	U_{13}	U_{12}
O18	24.1(4)	15.1(3)	16.4(3)	3.3(3)	-2.4(3)	-0.1(3)
O37	24.4(4)	14.3(3)	18.8(4)	3.7(3)	2.5(3)	0.2(3)
O38	22.0(4)	14.8(3)	25.5(4)	3.1(3)	-4.9(3)	1.3(3)
O19	22.5(4)	14.7(3)	26.5(4)	3.0(3)	4.4(3)	0.0(3)
C1	16.6(5)	12.4(4)	18.9(5)	-0.7(4)	-0.1(4)	-1.7(3)
C7	15.8(5)	17.5(5)	20.6(5)	4.0(4)	0.7(4)	1.5(4)
C8	17.0(5)	14.0(4)	15.1(4)	2.3(4)	-1.0(4)	0.4(3)
C12	12.7(4)	18.3(5)	19.3(5)	2.4(4)	-0.7(4)	2.0(4)
C20	18.1(5)	14.8(4)	21.6(5)	-2.9(4)	-2.5(4)	4.4(4)
C27	16.6(5)	13.9(4)	16.5(5)	2.9(4)	0.5(4)	0.2(3)
C26	16.2(5)	17.4(5)	20.2(5)	4.5(4)	-0.8(4)	-0.2(4)
C31	13.3(4)	19.6(5)	19.6(5)	2.3(4)	0.2(4)	-0.4(4)
C13	17.5(5)	26.1(5)	17.1(5)	2.6(4)	1.2(4)	1.3(4)
C17	17.7(5)	21.7(5)	17.1(5)	0.0(4)	0.5(4)	0.1(4)
C28	20.4(5)	14.6(5)	18.7(5)	0.7(4)	0.2(4)	0.7(4)
C36	20.6(5)	21.1(5)	18.1(5)	0.2(4)	-1.6(4)	2.0(4)
C16	18.4(5)	22.7(5)	22.0(5)	4.9(4)	1.6(4)	-0.4(4)
C9	22.5(5)	14.6(5)	18.4(5)	0.5(4)	0.1(4)	-0.6(4)
C15	17.2(5)	18.0(5)	29.4(6)	-0.6(4)	-0.7(4)	0.2(4)
C2	19.8(5)	19.4(5)	28.0(6)	2.8(4)	-0.7(4)	2.0(4)
C14	19.9(5)	25.6(5)	21.7(5)	-5.1(4)	-0.4(4)	1.7(4)
C35	21.6(5)	22.3(5)	25.0(5)	4.3(4)	-1.9(4)	3.1(4)
C6	21.5(5)	22.6(5)	20.4(5)	1.7(4)	0.4(4)	0.1(4)

C34	18.8(5)	20.3(5)	31.8(6)	-2.5(4)	1.7(4)	2.3(4)
C33	22.2(5)	31.5(6)	21.9(5)	-6.6(4)	1.1(4)	1.0(4)
C32	19.4(5)	30.6(6)	18.1(5)	2.5(4)	-0.7(4)	1.8(4)
C30	20.0(5)	17.4(5)	33.7(6)	0.1(4)	-7.1(4)	-0.7(4)
C11	20.7(5)	17.5(5)	38.0(6)	0.6(4)	7.4(4)	3.1(4)
C29	27.7(6)	18.1(5)	22.6(5)	-1.2(4)	-6.5(4)	0.3(4)
C10	30.8(6)	17.5(5)	26.3(6)	-2.1(4)	9.0(5)	0.8(4)
C25	25.2(5)	31.0(6)	21.3(5)	0.8(4)	-2.9(4)	8.8(4)
C21	22.5(5)	18.6(5)	40.2(7)	0.8(5)	-5.5(5)	-0.7(4)
C5	33.6(6)	28.7(6)	21.6(5)	-0.9(4)	6.9(5)	-4.0(5)
C4	28.8(6)	22.0(5)	38.6(7)	-5.1(5)	15.6(5)	-1.6(4)
C3	19.8(5)	21.5(5)	45.4(7)	1.6(5)	5.0(5)	4.4(4)
C24	45.0(8)	39.1(7)	24.0(6)	-6.0(5)	-11.9(5)	21.5(6)
C23	45.9(8)	26.3(6)	50.0(8)	-15.1(6)	-31.9(7)	14.9(6)
C22	29.2(6)	21.1(6)	66.2(10)	-4.0(6)	-21.7(6)	-0.9(5)

Table S16. Bond Lengths for StC_BMC_RS212_autored.

Atom	Atom	Length/Å	Atom	Atom	Length/Å
O18	C8	1.4238(11)	C31	C32	1.3950(15)
O37	C27	1.4248(11)	C13	C14	1.3942(15)
O38	C28	1.4301(12)	C17	C16	1.3900(15)
O19	C9	1.4301(12)	C28	C29	1.5169(14)
C1	C8	1.5244(13)	C36	C35	1.3923(15)
C1	C2	1.3979(14)	C16	C15	1.3902(16)
C1	C6	1.3924(14)	C9	C10	1.5191(14)
C7	C8	1.5671(13)	C15	C14	1.3873(16)
C7	C12	1.5137(14)	C2	C3	1.3867(16)
C7	C11	1.5609(14)	C35	C34	1.3871(16)
C8	C9	1.5453(13)	C6	C5	1.3953(15)
C12	C13	1.3957(14)	C34	C33	1.3868(16)
C12	C17	1.3994(14)	C33	C32	1.3919(16)
C20	C27	1.5239(14)	C30	C29	1.5390(16)
C20	C25	1.3930(15)	C11	C10	1.5372(17)
C20	C21	1.3959(15)	C25	C24	1.3934(17)
C27	C26	1.5665(13)	C21	C22	1.3884(17)
C27	C28	1.5441(13)	C5	C4	1.3852(18)
C26	C31	1.5145(14)	C4	C3	1.3798(19)
C26	C30	1.5563(15)	C24	C23	1.380(2)
C31	C36	1.3958(14)	C23	C22	1.380(2)

Table S17. Bond Angles for StC_BMC_RS212_autored.

Atom	Atom	Atom	Angle/˚	Atom	Atom	Atom	Angle/˚
C2	C1	C8	118.40(9)	C14	C13	C12	121.14(10)
C6	C1	C8	123.52(9)	C16	C17	C12	121.34(10)
C6	C1	C2	117.98(10)	O38	C28	C27	111.11(8)
C12	C7	C8	112.44(8)	O38	C28	C29	107.22(8)
C12	C7	C11	115.84(8)	C29	C28	C27	103.34(8)
C11	C7	C8	104.75(8)	C35	C36	C31	121.48(10)
O18	C8	C1	110.46(8)	C17	C16	C15	120.08(10)
O18	C8	C7	110.39(8)	O19	C9	C8	111.38(8)
O18	C8	C9	103.61(8)	O19	C9	C10	107.17(8)
C1	C8	C7	116.75(8)	C10	C9	C8	103.18(8)

C1	C8	C9	112.13(8)	C14	C15	C16	119.45(10)
C9	C8	C7	102.49(8)	C3	C2	C1	121.14(10)
C13	C12	C7	120.46(9)	C15	C14	C13	120.23(10)
C13	C12	C17	117.76(9)	C34	C35	C36	120.04(10)
C17	C12	C7	121.71(9)	C1	C6	C5	120.75(10)
C25	C20	C27	123.09(10)	C33	C34	C35	119.35(10)
C25	C20	C21	117.92(10)	C34	C33	C32	120.28(10)
C21	C20	C27	118.89(10)	C33	C32	C31	121.28(10)
O37	C27	C20	110.75(8)	C29	C30	C26	106.71(8)
O37	C27	C26	110.46(8)	C10	C11	C7	106.66(8)
O37	C27	C28	103.69(8)	C28	C29	C30	104.11(8)
C20	C27	C26	116.37(8)	C9	C10	C11	103.63(9)
C20	C27	C28	111.92(8)	C20	C25	C24	121.07(12)
C28	C27	C26	102.66(8)	C22	C21	C20	120.76(12)
C31	C26	C27	111.63(8)	C4	C5	C6	120.28(11)
C31	C26	C30	117.00(8)	C3	C4	C5	119.52(10)
C30	C26	C27	104.82(8)	C4	C3	C2	120.31(11)
C36	C31	C26	122.24(9)	C23	C24	C25	120.13(13)
C32	C31	C26	120.09(9)	C24	C23	C22	119.49(11)
C32	C31	C36	117.55(10)	C23	C22	C21	120.61(13)

Table S18. Hydrogen Bonds for StC_BMC_RS212_autored.

D	H	A	d(D-H)/Å	d(H-A)/Å	d(D-A)/Å	D-H-A/°
O18	H18	O38	0.84	1.99	2.8259(10)	175.3
O37	H37	O19[1]	0.84	2.00	2.8405(10)	173.8

Table S19. Hydrogen Atom Coordinates (Å×10⁴) and Isotropic Displacement Parameters (Å²×10³) for StC_BMC_RS212_autored.

Atom	*x*	*y*	*z*	U(eq)
H18	2732.19	4403.05	1274.44	28
H37	2366.17	9434.78	1372.89	29
H38	3438.11	6057.47	2394.59	31
H19	1578.37	1057.31	2473.75	32
H7	4397.12	2602.66	3029.09	21
H26	551.5	7401.77	2834.27	21
H13	4924.58	4327.9	4027.25	24
H17	5689.72	4158.43	759.54	23
H28	3593.69	6933.19	821.88	22
H36	-651.05	9101.52	677.17	24
H16	6787.28	5944.58	906.82	25
H9	1594	1815.79	846.4	22
H15	6945.83	6939.37	2616.02	26
H2	270.22	4011.68	1728.52	27
H14	6006.97	6123.67	4173.15	27
H35	-1706.17	10858	929.52	27
H6	2645.37	2647.3	4094.91	26
H34	-1798.51	11751.12	2685.21	29
H33	-891.59	10843.22	4187.55	31

H32	97.55	9063.16	3939.76	27
H30A	-396.34	6267.41	1511.53	29
H30B	-699.9	7297.88	750.96	29
H11A	5890.9	2357.65	1056.53	30
H11B	5463.16	1386.53	1869.98	30
H29A	1308.4	5814.75	233.58	27
H29B	1432.06	7081.23	-163.97	27
H10A	3863.91	2016.63	1.08	30
H10B	3939.55	783.77	478.18	30
H25	2118.53	7445.95	4017.04	31
H21	4823.6	9071.3	1993.17	33
H5	826.23	3098.03	5370.01	34
H4	-1271.3	3996.62	4824.16	36
H3	-1548.23	4435.04	2998.06	35
H24	3748.18	7839.23	5469.27	43
H23	5917.31	8839.65	5184.69	49
H22	6458	9431.05	3441.41	47

Experimental

Single crystals of $C_{17}H_{18}O_2$ [StC_BMC_RS212_autored] were obtained by crystallization from 2-propanol. A suitable crystal was selected and [loop] on a XtaLAB Synergy, Single source at home/near, HyPix diffractometer. The crystal was kept at 120.00(10) K during data collection. Using Olex2 [1], the structure was solved with the SHELXT [2] structure solution program using Intrinsic Phasing and refined with the SHELXL [3] refinement package using Least Squares minimisation.

Crystal Data for $C_{17}H_{18}O_2$ (*M* =254.31 g/mol): triclinic, space group P-1 (no. 2), *a* = 9.17590(10) Å, *b* = 11.9105(2) Å, *c* = 12.2034(2) Å, *α* = 93.1670(10)°, *β* = 90.2170(10)°, *γ* = 91.3510(10)°, *V* = 1331.28(3) Å^3, *Z* = 4, *T* = 120.00(10) K, μ(Cu Kα) = 0.645 mm^{-1}, *Dcalc* = 1.269 g/cm^3, 51439 reflections measured (7.256° ≤ 2Θ ≤ 153.734°), 5510 unique (R_{int} = 0.0235, R_{sigma} = 0.0096) which were used in all calculations. The final R_1 was 0.0364 (I > 2σ(I)) and wR_2 was 0.0892 (all data).

References (X-ray report):

[1] Dolomanov, O.V., Bourhis, L.J., Gildea, R.J, Howard, J.A.K. & Puschmann, H. (2009), J. Appl. Cryst. 42, 339-341.

[2] Sheldrick, G.M. (2015). Acta Cryst. A71, 3-8.

[3] Sheldrick, G.M. (2015). Acta Cryst. C71, 3-8.

Computational Details

General remarks

The Orca 5.0.4 program package was used for the DFT calculations.[39] Initial conformer searches were carried out using the CREST 2.12 program package using the GFN-FF level of theory with implicit solvation (THF), combined with subsequent treatment using the CENSO program 1.2.0 (based on ENSO version 2.0.1).[40] An additional manual search for potentially lower conformers was carried out as well. The structure optimizations were finalized using the r^2SCAN-3c method,[41] which makes use of the re-regularized SCAN exchange functional by Furness *et al.*,[42] the geometrical counterpoise correction gCP,[43] the D4 atom-pairwise dispersion correction,[44] and the def2-mTZVPP basis set with the matching auxiliary basis set def2-mTZVPP/J.[41] For optimizations, the *defgrid3* grid was applied.
The conductor-like polarizable continuum model (CPCM)[45] was applied for the optimizations using THF as solvent. Frequency analyses were carried out analytically.
Stationary points (minimum structures) were characterized by the absence of imaginary frequencies, transition states were characterized by the presence of one imaginary frequency that matched the transition state trajectory. The correction to the Gibbs Free Energy was obtained from the Orca output of the frequency calculation.
Single-point calculations were carried out using the PW6B95[46] functional together with the def2-QZVP basis set[47] and matching auxiliary basis sets.[48] The single point calculations were carried out with the *NormalSCF* option and CPCM(THF) solvation. The RI-J approximation for Coulomb integrals and the COSX numerical integration for HF exchange (RIJCOSX) were applied.[49,50] Furthermore, the D4 dispersion correction was applied.[44] The correction $\Delta G^{*\rightarrow o}_{solv}$ (= 1.90 kcal mol^{-1}, see Born-Haber cycle in Scheme S1) was added to obtain the energy in solution. CYLview 2.0 was used for visualization.[51]

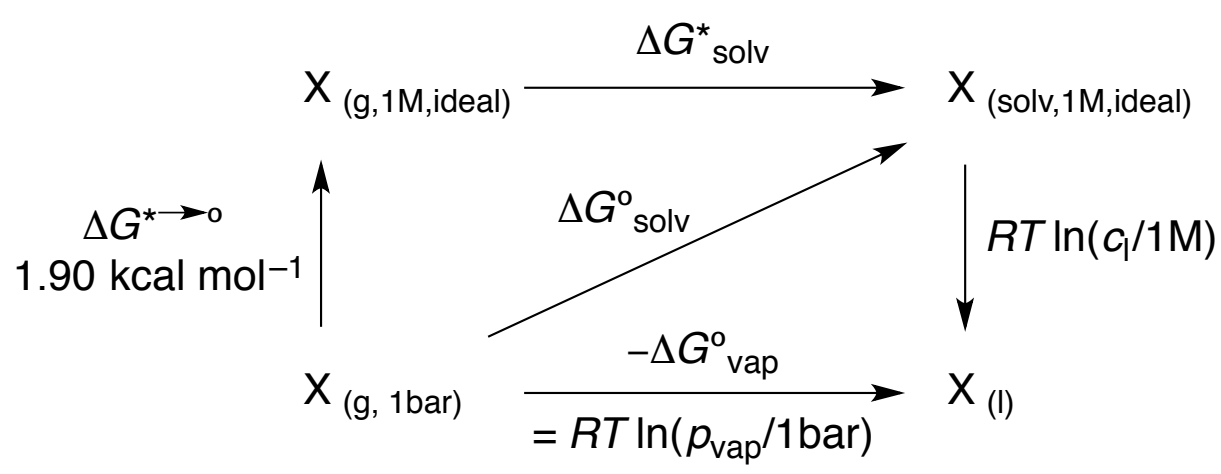

Scheme S1. Interconversion Scheme for the calculation of solvation energies for a compound X.

Transition state optimizations

The transition state optimization was carried out on the broken symmetry hypersurface, using the *brokensym 1,1* option.[52] First, a relaxed scan or a structure optimization with a fixed C-C bond length was carried out, followed by a final transition state optimization using the *TSOpt* option. For the lowest energy conformers, a frequency calculation and single point calculation were added to determine the Gibbs Free Energies in solution. The lowest free energy conformer was then chosen as the lowest-lying transition state conformer. Note: switching off the TRAH SCF solver facilitated SCF convergence of the broken symmetry transition state optimizations.
We used the broken symmetry formalism in the calculation of titanium(III)-catalyzed ketone-nitrile couplings previously and more details on the approach are given in our earlier work.[53]

Energies and Imaginary Frequencies (TS Structures)

Table S20. Computational data.

structure	r^2SCAN-3c / Hartree	PW6B95-D4-CPCM/def2-QZVP / Hartree	correction to G / kcal mol^{-1}	G_{solv} / kcal mol^{-1}	ΔG / kcal mol^{-1}	ν_{imag} / cm^{-1}
**1**	–786.6498274	–788.2166629	145.07	–494466.45	**0***	
**Cp_2TiCl**	–1696.742176	–1698.523159	82.84	–1065754.63		
**A**	–4180.183821	–4185.306752	343.55	–2625974.19	**1.53**	
**TS_{A-B}**	–4180.172869	–4185.283458	342.97	–2625960.15	**15.57**	–160.63
**$TS_{A-B'}$**	–4180.173852	–4185.28132	344.23	–2625957.55	**18.17**	–229.84
**B**	–4180.213783	–4185.320148	347.89	–2625978.25	**–2.53**	
**B'**	–4180.211855	–4185.317709	347.51	–2625977.10	**–1.38**	
**4**	–1196.504148	–1198.633619	219.76	–751932.29	**–10.41**	
**4'**	–1196.505382	–1198.634146	219.84	–751932.54	**–10.66**	
**Cp_2TiCl_2**	–2156.985232	–2159.16042	83.86	–1354807.86		
**morph•HCl**	–748.5585276	–749.5254697	72.92	–470259.51		
**morph**	–287.7307083	–288.2922976	66.3	–180837.95		
**TMSCl**	–869.4488457	–870.4117148	49.38	–546140.32		

* Refers to substrate and two Cp_2TiCl entities.

Additional results for higher-energy conformers that have been calculated can be found in the calc_results.xlsx file and the orca_additional_conformers folder of in the Zenodo data repository (see the link in the manuscript).

Transition State Structures

Note: The images of **TS_{A-B}** Figure S23 and in manuscript Figure 2 were flipped vertically (the enantiomeric structures were calculated) to match the drawings in manuscript Figure 2a. This further allows an easier comparison with **$TS_{A-B'}$** and has no effect on the reported data and the conclusions.

A conformer of **TS_{A-B}** with a smaller Cl-Ti-C-C dihedral angle as in **$TS_{A-B'}$** was calculated and is shown in Figure S23 as **TS_{A-B}-2**. It was found to be 2.4 kcal mol^{-1} higher in energy.

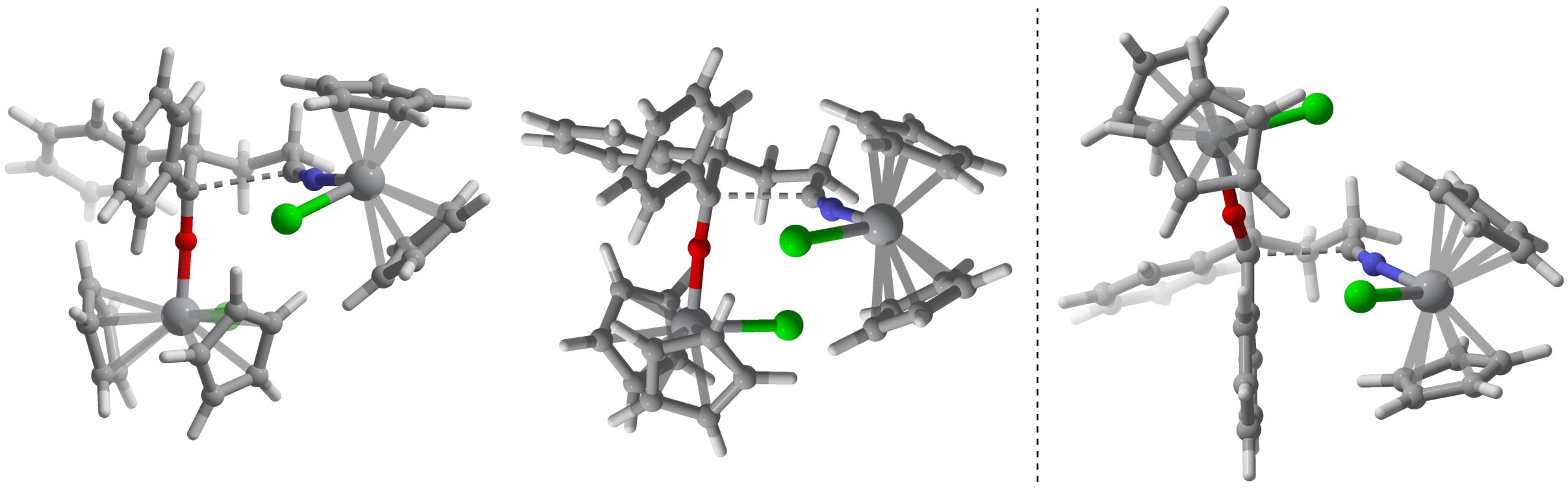

TS_{A-B} **TS_{A-B}-2** (+2.4 kcal mol^{-1}) **$TS_{A-B'}$** (+2.6 kcal mol^{-1})

Figure S23. Enlarged transition state structures **TS_{A-B}** (left) and the conformer **TS_{A-B}-2** (middle) with a similar dihedral angle as **$TS_{A-B'}$** (right).

Coordinates

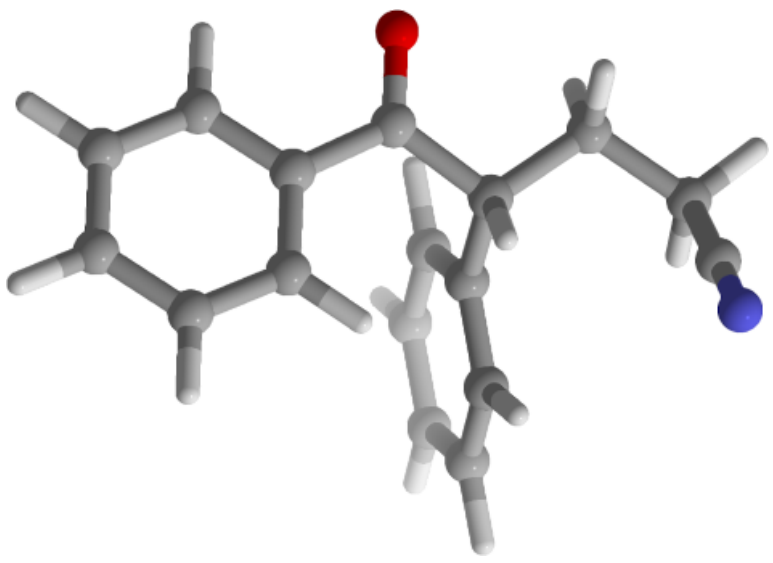

1

C	-3.51527307194711	1.27908353127579	-1.00372565702438
C	0.54376391902021	1.23667868734105	0.74652611887721
C	-3.33718308888513	0.97797443289659	0.40715442706079
C	-1.96107335285480	1.41818162301366	0.93475980269806
H	-3.47793032579507	-0.10178144310539	0.54019340029356
H	-4.13355182326800	1.48281620234852	0.96563620693587
C	-0.79685677303853	0.75213007892226	0.18979352751046
H	-1.91612112191076	1.16227778322043	1.99745141279422
H	-1.86707215045817	2.50613892755737	0.85690096419144
C	-0.86485574812081	-0.76360205796441	0.27025508588230
H	-0.84803491787496	1.04211698946018	-0.86882287647128
C	-0.59456364088183	-1.41205440183652	1.47897559005836
C	-1.23222709005587	-1.52610935644076	-0.84052378938449
C	-1.32942424292087	-2.91222273076686	-0.74587065838736
C	-1.05492076479548	-3.55187453011115	0.46056437330683
C	-0.68725053244228	-2.79753656758372	1.57311951493696
H	-0.30791664576743	-0.83002813972328	2.35251247262594
H	-0.46954539050927	-3.28926531176838	2.51721988207598
H	-1.61964848769694	-3.49258766349422	-1.61736033632921
H	-1.12669433824722	-4.63338328958851	0.53382332766808
H	-1.44534932703097	-1.03051672516861	-1.78548366832303
C	1.79596929930165	0.88005721841826	0.01976498591919
C	3.02001705550013	1.08004992954832	0.67323873787831
C	1.79395792695593	0.36597453185758	-1.28363561814874
C	2.99323161137165	0.06516135606090	-1.91984615884196
C	4.20393854492454	0.26226569204330	-1.25986806831841
C	4.21467585329208	0.76934906594372	0.03973325439603
H	5.15767555350105	0.92089224467687	0.55708400587215
H	3.01550810209857	1.47603855307284	1.68398743215219
H	2.98206306715962	-0.32510295049819	-2.93331956289272
H	5.13928337570687	0.02146854655633	-1.75754522964022
H	0.86273726941519	0.20493642148755	-1.81568386739385
N	-3.63012603564881	1.52449770966334	-2.12808154771737
O	0.58851546660286	1.87552316958512	1.78884357543909

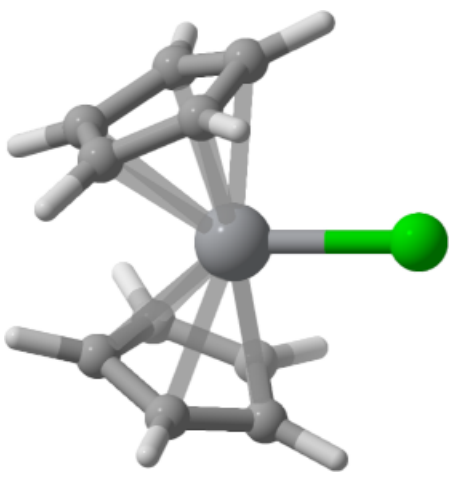

Cp_2TiCl

C	-2.32507978740517	0.40483358929020	0.86046515375555
C	-1.86739994370979	1.19021603129920	-0.22903047362575
C	-1.46451544826850	0.29901523050019	-1.25559996614585
C	-1.64938880337474	-1.03020916411449	-0.79157996027698
H	-1.84458610821346	2.27135081406919	-0.27583845740941
C	-2.18837714530919	-0.96082502694910	0.51391245353944
H	-2.67198692418970	0.78333924289643	1.81189358162532
H	-2.40936976109147	-1.80364587514357	1.15668698663856
Ti	-0.00000015576509	-0.00000199165420	0.57439454782889
C	1.64938708955993	1.03020901838120	-0.79158285557957
C	2.18837204345189	0.96082781284945	0.51391130872507

C	1.46451718277392	-0.29901611941098	-1.25560120940886
C	1.86740371865445	-1.19021509719327	-0.22902999351604
C	2.32507935101046	-0.40482921226139	0.86046535910836
H	1.06447584147113	-0.58697390500302	-2.21801588113266
H	1.40619057548712	1.93432462486907	-1.33512718131063
H	2.40936267187657	1.80364880911999	1.15668569981402
H	1.84459397617992	-2.27135014521981	-0.27583644569763
H	2.67199047130251	-0.78333259285852	1.81189276116883
Cl	-0.00000185394873	-0.00001335100954	2.95007305297602
H	-1.06447310917603	0.58697292103659	-2.21801454849131
H	-1.40619388131605	-1.93432561349362	-1.33512403258534

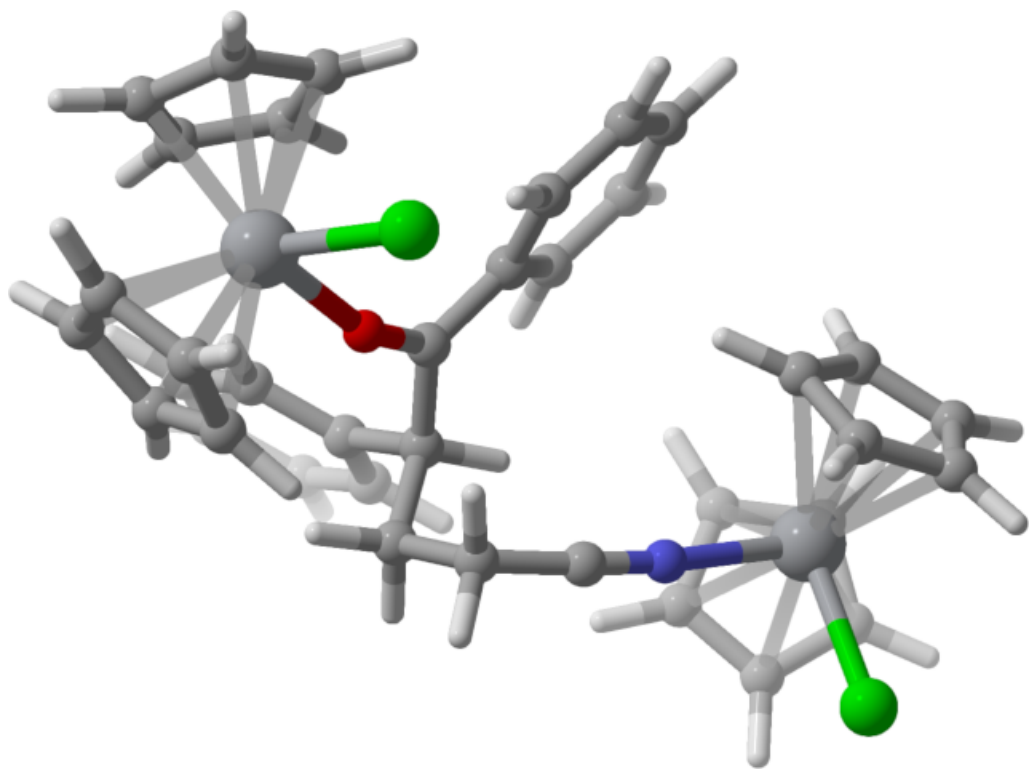

A

C	1.49354877414487	-0.99887137352696	-1.79727153708476
C	-1.06160271361109	-0.02601669884508	0.30244295024708
C	0.24309114886683	-1.72606238806832	-1.83696835906294
C	-0.94179300651906	-0.77377931314249	-2.11238193441183
H	0.12483310631560	-2.23498740183160	-0.87161000233537
H	0.30168373284890	-2.50045119832906	-2.61036961906858
C	-0.98285250919241	0.42053094819557	-1.14725614324490
H	-1.86313002956111	-1.35671347954358	-2.04006101575874
H	-0.86971976547578	-0.38771165712668	-3.13449284416824
C	-2.11399627398199	1.37570617690509	-1.49717320615483
H	-0.04596191332990	0.97703032943742	-1.27262330743673
C	-1.85399982868788	2.46090548877677	-2.33952637172201
C	-3.41195112507594	1.19083403156234	-1.01415345678724
C	-4.43119251692747	2.06895556449622	-1.37455324351284
C	-4.16617732791262	3.14319663221000	-2.22030448647466
C	-2.87301317297130	3.33699691742330	-2.70162537647035
H	-0.84348549903020	2.62082566213848	-2.71059053415545
H	-2.65463701450147	4.17560570828739	-3.35709225086715
H	-5.43499285802549	1.91345580215785	-0.98845760284815
H	-4.96122832397342	3.82900148215272	-2.49900850518941
H	-3.63033112486317	0.36120515906923	-0.34842387726377
C	-0.32523314002498	0.72584462218080	1.31424905401334
C	0.02634897820557	2.07412908565758	1.10668469796787
C	0.09042655206117	0.08974320515008	2.49772932438785
C	0.82940170436764	0.78300214487023	3.44430569071890
C	1.15922793837418	2.12329638030057	3.23731635408909
C	0.75154431525310	2.76436785922484	2.06744018936748
H	1.00254127602047	3.80858916471345	1.90334002541736
H	-0.29803279280325	2.59282271489894	0.20934347702266
H	1.16238535873266	0.27364209009733	4.34437328540958
H	1.73874952049818	2.66365806832314	3.98036861186315
H	-0.14184893201070	-0.96116632254351	2.63994489688064
N	2.42661275715280	-0.32046613332509	-1.73469553367724
O	-1.74892803421390	-1.04965944421191	0.57657318235193
Cl	-1.06806163177608	-3.42438711221017	2.57359290858079
H	-5.79726113740336	-2.32459649615623	2.98753097270389
H	-1.94644223128641	-3.59590481382579	-0.88117684399698
H	-3.69302041812726	-2.93724006970516	4.53746496648143
C	-4.88791025013492	-1.74765171507218	3.08021047639453
C	-2.89025807560824	-3.56979896041109	-0.35396895032569
C	-3.78541716587426	-2.06834437948304	3.89932209024624
H	-2.68763827178402	-5.26800567498519	1.07063516665625

Ti	-3.03114925587094	-2.23765916357230	1.64314736701154
C	-3.28118836342328	-4.45701659490808	0.67475468519246
C	-3.93591212770330	-2.62739127338439	-0.54175272746991
C	-4.58273620190763	-0.55605014426109	2.36955606562627
H	-3.94968891885554	-1.81220785219282	-1.25240424615128
H	-5.21828481319503	-0.06533310109579	1.64349582704973
C	-2.80897231743060	-1.04849689042370	3.72723925519347
C	-4.54632021523799	-4.05358422740018	1.14740765084620
C	-3.30912439195957	-0.11449771461070	2.79841312836597
C	-4.95686525340327	-2.92582101364773	0.38400822845773
H	-1.84281885055496	-1.01510807649251	4.20824054622717
H	-5.09948183415471	-4.51459970134145	1.95507357421628
H	-5.88440545204191	-2.38209665581786	0.49873018610788
H	-2.78858341302108	0.76103734788398	2.43376573885636
Cl	5.31404841052405	-0.49279724797457	-2.98756368712378
H	5.70225132588482	2.99228997989151	-3.20879665609795
H	3.88576362460608	1.54630334664356	-4.57282902679186
C	4.65718456865365	2.87522768558972	-2.95344562915624
C	5.75219332128902	0.39597544439634	0.02715315448718
C	3.69443295969487	2.12708371682299	-3.68216348544709
H	6.22664062418357	2.56812245635439	0.10626379037595
Ti	4.03832467628036	1.09746753939024	-1.51706371838240
C	5.51945255266497	1.76450618996123	0.26706185079200
C	4.55802625981766	-0.30995319967211	0.31400452417022
C	3.99856409876710	3.43929885214863	-1.83212377059550
H	4.40411343824898	-1.37337123566073	0.18859228568669
H	4.45415453829088	4.07201914857091	-1.08324328838923
C	2.45947642017406	2.22340994488593	-3.00930504161809
C	4.18199052542634	1.90718936994051	0.73469366412398
C	2.64297584046322	3.01569304805656	-1.85049120164337
C	3.58865339887345	0.62974632348232	0.76827611000472
H	1.54158420089430	1.73052708022569	-3.30088405726384
H	3.69318321583853	2.83686277092544	0.99291307514142
H	2.57097476992726	0.40889612902727	1.06283460245493
H	1.88752308517421	3.25630340632437	-1.11334360738595
H	6.66178799492814	-0.03231563915174	-0.36908587825381

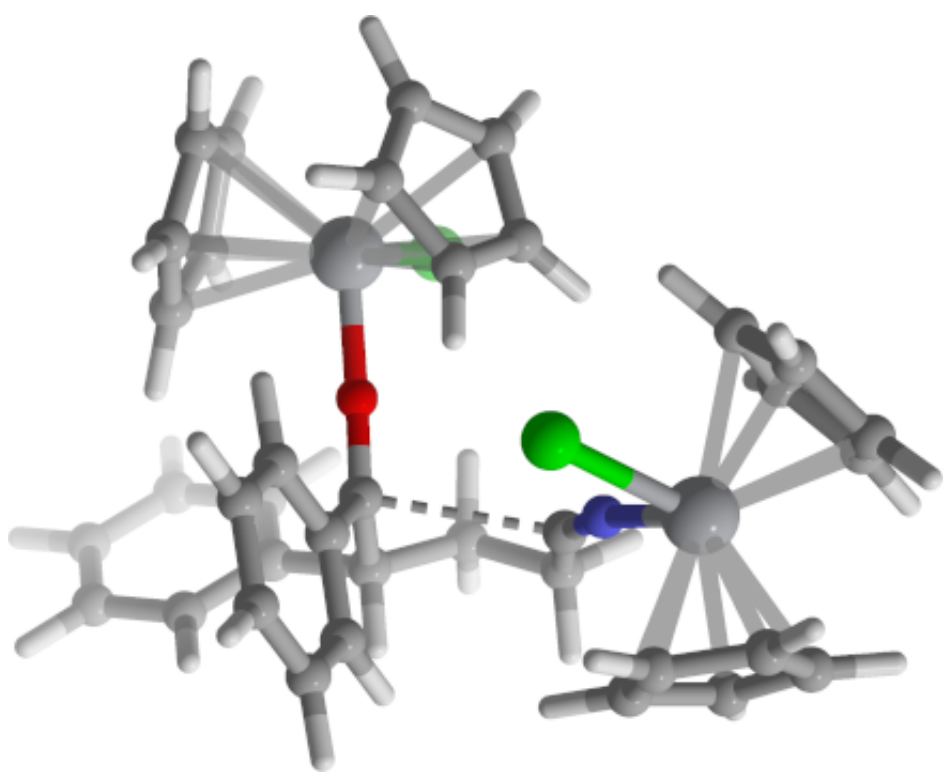

TS$_{A\text{-}B}$

C	0.91534234101531	1.00193939319142	1.61144485462328
C	-1.17228152494700	1.08699402802216	-0.04334465592469
C	0.04426083493324	1.49510875465776	2.68414394233635
C	-1.45661768939266	1.40017838856610	2.41227990904084
H	0.33425605048783	2.54451796821268	2.83779280990549
H	0.31630925870881	0.94618908252946	3.59503381068500
C	-1.77526181504478	1.97623689924888	1.03129250536456
H	-1.98679867463547	1.95748854007204	3.19218172670340
H	-1.77571132653063	0.35488908735696	2.46715244617011
C	-3.25742660054008	2.15061739952614	0.74438291945296
H	-1.31527295140529	2.96768306570768	0.95888475466106
C	-3.64841368754645	3.04553106950611	-0.25923074280719
C	-4.24163131624171	1.41339140751159	1.40517941558203
C	-5.58640786048507	1.56888926642234	1.07078288700250
C	-5.96558915394662	2.45711159006275	0.06901886141322
C	-4.98877402920681	3.19820567979828	-0.59619392117308
H	-2.88915445051421	3.62317776490426	-0.78284947711681

H	-5.27285198139984	3.89925542383224	-1.37641946077258
H	-6.33976312265979	0.98718448677019	1.59576591198305
H	-7.01393477211093	2.57531995763630	-0.19058978992557
H	-3.96603957567892	0.70309218683764	2.17888025281229
C	-0.50920417847119	1.61101890975582	-1.21547654925151
C	0.12218174139950	2.87728953085929	-1.21502785813027
C	-0.48415543610906	0.87219493860763	-2.41938973450837
C	0.13080382384464	1.37230975336311	-3.55629448247016
C	0.75438328389956	2.62043808639517	-3.53607098967490
C	0.74299231508580	3.36479138705823	-2.35370235277894
H	1.23174347940892	4.33505376716589	-2.31894152381967
H	0.14799806933056	3.47439050272283	-0.30865478624203
H	0.12734672837681	0.78150099092756	-4.46857387049965
H	1.24538394588909	3.00637542638698	-4.42438206727264
H	-0.96501614040678	-0.09799563151161	-2.45410130121107
N	1.93833796113978	0.69834034109406	1.13039391366563
O	-1.60018743712931	-0.14940695549326	-0.02838154152566
Ti	-2.10639344682959	-1.94855547735246	-0.21110097962794
Cl	-2.74434715370105	-2.23028234796262	2.12327248005031
C	0.09712202066764	-2.54808732363288	0.57561235222704
C	-3.30703633386971	-1.65112801135648	-2.26988339857682
C	-3.95601033370646	-0.82225260194811	-1.31847983806857
C	-4.50872869992863	-1.65001657710205	-0.32893100413840
C	-4.23677113236330	-3.00690483564323	-0.67828804258999
C	-3.52962703112547	-3.00193480617961	-1.89425154781310
H	-3.18340763483986	-3.86881318574006	-2.43824881171874
H	-4.51635310364899	-3.87951174186109	-0.10251696991891
C	-0.64692138535847	-3.75548430910527	0.50191872341978
C	0.25591584550316	-2.05268263718687	-0.73495580967562
C	-0.43724393531246	-2.91389111321324	-1.61602304015729
C	-0.97224703965905	-3.98818792112241	-0.84551921352792
H	-0.94629827440527	-4.36872807702798	1.33973261426148
H	0.76180670982996	-1.13565976907448	-1.00644904291063
H	-0.52239971959793	-2.79505307677615	-2.68840884224564
H	-1.53390576125185	-4.83126206044312	-1.22086588512384
H	-3.94880421314795	0.25996738640472	-1.30725196021751
H	-5.01934623261898	-1.31647899477476	0.56187544267628
Ti	3.95146703113370	0.42412458407629	0.63822745250562
Cl	3.36647210550556	-0.11682634003190	-1.71811371346764
C	5.05334782569754	-1.67444726253039	0.52090909904890
C	5.80173926280282	1.89890096267758	0.24088047253612
C	4.74800802704030	2.39426219596720	-0.55703370164404
C	5.41141449937705	2.02819661018093	1.60371972849266
C	4.10602994262639	2.56391406514217	1.64007092462965
C	3.69460999506103	2.78683293181591	0.29403280990842
H	4.72305898993690	2.40337908115459	-1.63684978985318
H	3.52102830228687	2.76223452948596	2.52876970315723
H	6.00608818717823	1.74734265041886	2.46159521585503
C	3.70633279776987	-1.93259736459007	0.89560602627640
C	5.66304828156302	-0.95707032302570	1.57134842665952
C	4.69524379024257	-0.76188515843027	2.59528129821128
C	3.49894475795685	-1.38567143602230	2.18384678644071
H	2.96360731390326	-2.43373668163547	0.29083415546127
H	6.68197209053562	-0.59397739900010	1.58407781753614
H	4.84554820562381	-0.22454168171587	3.52234662185200
H	2.73015670704562	3.15730661832808	-0.02577097304079
H	2.57131480299317	-1.39805979141983	2.73947971600596
H	-2.77949946124657	-1.31929345234969	-3.15448993150968
H	5.51561237977487	-1.94919599233335	-0.41723217897907
H	6.73954824190961	1.49549241997597	-0.11882794128259
H	0.44644815622891	-2.07203634924508	1.48177668517912

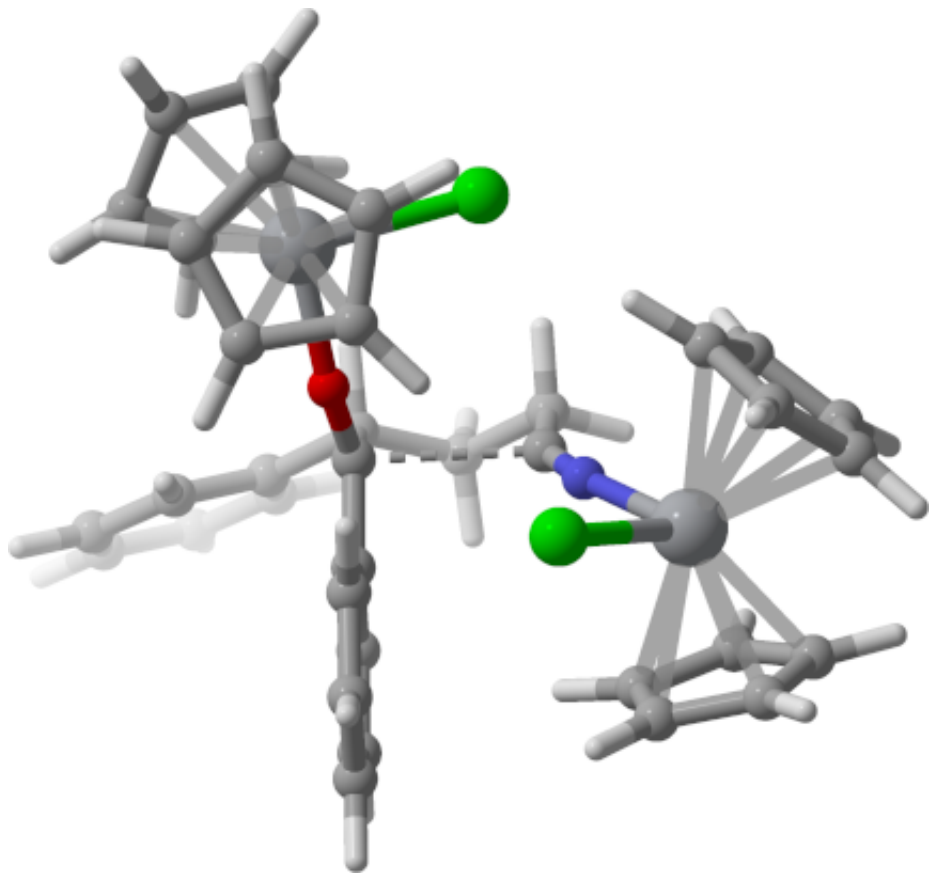

$TS_{A\text{-}B'}$

C	0.79678806777702	-0.60800666786324	-1.35229580442759
C	-1.29640586386564	-0.75715405695500	-0.06243306086849
C	0.08031603284151	-1.00464077321640	-2.56588298074310
C	-1.08077343089957	-1.95489820696791	-2.28942170715767
H	0.80655897203584	-1.45523379445967	-3.25570120554910
H	-0.29051113638981	-0.07443976514259	-3.01716211862800
C	-2.04251154282358	-1.32756333408546	-1.26209629844351
H	-0.69713177906282	-2.91932890809319	-1.94268310842351
H	-1.60337999356668	-2.12939682996739	-3.23425091574271
C	-3.20578430086118	-2.23360050804197	-0.86746214606013
H	-2.47049583194516	-0.44771067323009	-1.76010201901483
C	-3.85562028862314	-2.05520051754414	0.36041519193774
C	-3.67903386993217	-3.23378471354863	-1.72297282575542
C	-4.76640396642687	-4.02766892374426	-1.36229009163998
C	-5.40371050236145	-3.83811325297786	-0.13989611511735
C	-4.94223928304780	-2.84508439064554	0.72103750839433
H	-3.49750865213205	-1.29840465633320	1.05443674331901
H	-5.42501977831114	-2.68689478448171	1.68156192567097
H	-5.11353639830221	-4.79957596923241	-2.04396199198094
H	-6.24938180597859	-4.45950920098406	0.14102668551291
H	-3.20221541035271	-3.40926036539771	-2.68203386316088
C	-0.66948830260776	-1.62993471731774	0.92953181928965
C	-0.00073432441945	-1.06817364315148	2.03365760779361
C	-0.68952574197265	-3.03703494640051	0.82205563706612
C	-0.06601178846819	-3.83332541052525	1.77301672263259
C	0.59529109303164	-3.25882264675532	2.85905846710008
C	0.62050373240232	-1.86934780383344	2.97992688613345
H	1.13886904424646	-1.40424031232256	3.81420648330221
H	0.04782666423506	0.00927764750324	2.12694259881537
H	-0.10326744318934	-4.91446478494780	1.66848049285509
H	1.08373285523212	-3.88551499231129	3.59979909046464
H	-1.22080984076007	-3.51900397786680	0.01000102066089
N	1.76096571059097	-0.31167793539065	-0.75047496919255
O	-1.46204400440702	0.49234429206417	0.23633091423433
Cl	-0.52014782410955	2.55417456731840	-1.91682358505662
H	-3.24248732089635	4.19236034087126	-2.09441123150982
H	-1.50586937351857	1.59672171410925	2.93123258128920
H	-3.24377984493572	1.57446419776461	-2.70952779989179
C	-3.59916729690402	3.39179935886450	-1.46039865641022
C	-1.32411805329610	2.46402630557940	2.31053166134611
C	-3.60325959938737	2.00498925932205	-1.78550040808365
H	0.68719517502257	2.05776098160242	1.41300237401572
Ti	-2.02759387533689	2.34920812919515	0.02768671719032
C	-0.17406941712316	2.70516371103189	1.51547355256790
C	-2.22412833756927	3.53321698773265	2.10765743937669
C	-4.12691698686399	3.53014226290703	-0.16229049365735
H	-3.19916857281870	3.63591079486232	2.56402227973646
H	-4.26048788126065	4.45919044013173	0.37307988430580
C	-4.12187269566852	1.30209418689620	-0.68333354365049
C	-0.36324059313019	3.93242231415102	0.83958833849654
C	-4.41008477195357	2.23304786142909	0.34241828518896
C	-1.62915111346059	4.44260280670204	1.19147262383054

H	-4.24844198774520	0.23209878828268	-0.60526310434451
H	0.32336372533787	4.37769583423051	0.13430510941195
H	-2.07107619612807	5.35598596825656	0.81663734604643
H	-4.80369929246811	1.99888358019464	1.32302391373824
Cl	3.13792851310307	1.02400743524951	2.03585160577522
H	6.22981007337201	-0.93220302065020	1.25182727123594
H	3.93916030060881	-1.67759208199860	2.44777627695459
C	5.37407978012550	-1.38495703605181	0.76744509249613
C	4.74023053114558	2.16950594966939	-0.34152004559954
C	4.17436771519963	-1.79381023595576	1.40013199659986
H	6.51694140843827	0.95735592138424	-0.90307596369651
Ti	3.69230706264558	0.02751298653898	-0.14206405379812
C	5.49945541955798	1.26403422196682	-1.10645950365702
C	3.44856042431402	2.26460772323586	-0.92535006535904
C	5.25507850320220	-1.68458888224955	-0.61633115446287
H	2.61718707440935	2.84436547541531	-0.54670605984234
H	6.01205846193825	-1.51259263480265	-1.36807345218097
C	3.30476223188301	-2.29799316398004	0.41620009738869
C	4.68255273159185	0.80045701980033	-2.17838154927726
C	3.96803002526471	-2.22270441785060	-0.84152117530020
C	3.43058768917626	1.44030359872226	-2.07684038764000
H	2.29139980794126	-2.63727070793437	0.58538555091944
H	4.96913943941529	0.08374047119103	-2.93623602639106
H	2.58676339420441	1.29114025163838	-2.73605370791453
H	3.55734586742171	-2.51903747988476	-1.79809547100378
H	5.06585878756949	2.66480773927879	0.56188286753984

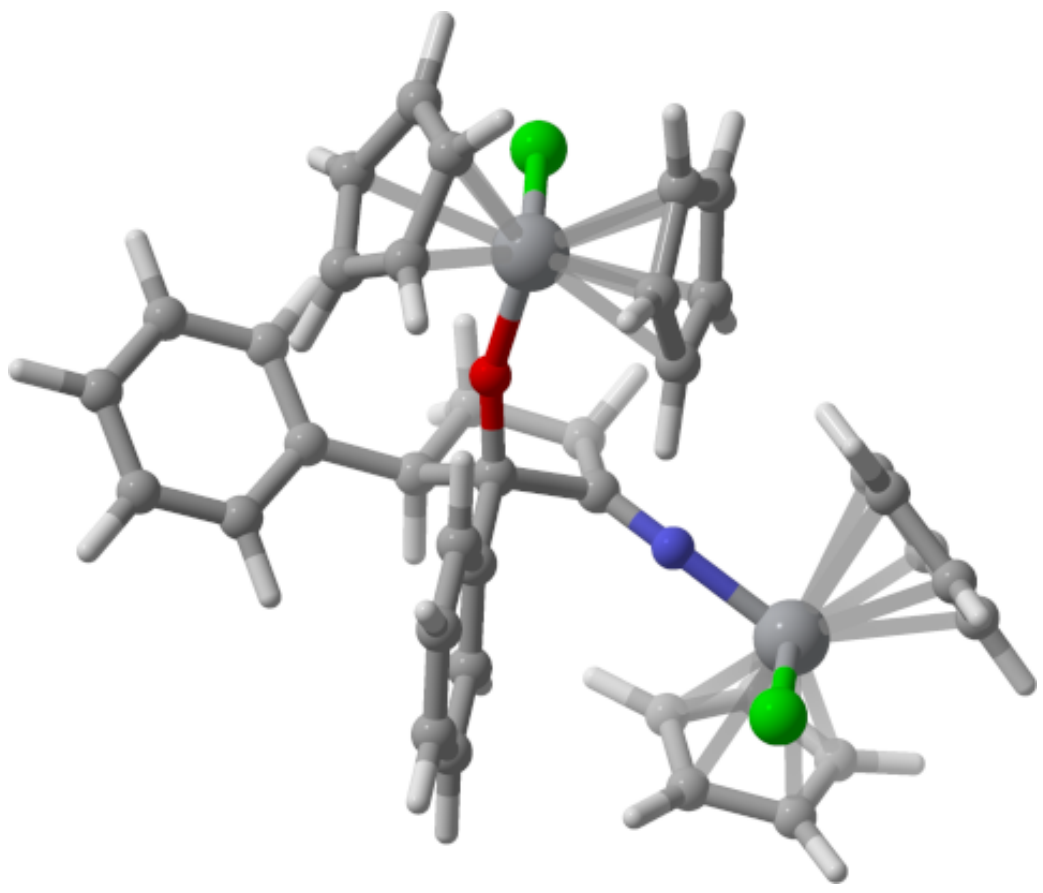

B

C	0.61392166305644	0.64691497056269	1.06897778460242
C	-0.67588567444467	0.89432725235519	0.19808516669817
C	0.21710642978318	0.76332549339122	2.53419890963381
C	-1.23557116872065	1.25682468242397	2.53288672354770
H	0.90327830859982	1.47019506965013	3.01725908058323
H	0.33962707519365	-0.20120969343068	3.04176754054167
C	-1.42082244691965	1.90177100886371	1.15183342435197
H	-1.44124418152123	1.96607913004889	3.34049149532704
H	-1.90676441689949	0.40208047578917	2.64463078654708
C	-2.83064505357596	2.21825740416945	0.72144618552844
H	-0.83704086619385	2.83349416301761	1.12843505922491
C	-3.04746742921194	3.11955396802893	-0.32987933653953
C	-3.94479429268052	1.63228920115121	1.32770582236562
C	-5.23486394516926	1.92410322204839	0.88812995785361
C	-5.43544254903225	2.80429603621665	-0.17058656275560
C	-4.33329006327063	3.40494315817158	-0.77721811962580
H	-2.19541215691544	3.60022224466021	-0.80354382009033
H	-4.47539555069930	4.10408258506198	-1.59705863505220
H	-6.08601737794427	1.45569348520748	1.37564918358040
H	-6.44128396569774	3.02840795337896	-0.51510204374796
H	-3.81539281535551	0.93606110197888	2.15002467370061
C	-0.35732768672735	1.47499797310281	-1.16629960869499
C	0.36438082690165	2.66995439956148	-1.26589413254497
C	-0.77493187138708	0.85555300144303	-2.34139269857174
C	-0.48440762318379	1.41049981740934	-3.58720257793690

C	0.23100961958845	2.59920298535303	-3.67520869483458
C	0.65444451771570	3.22759703308380	-2.50475646547586
H	1.21620863744790	4.15645082116176	-2.55694751959870
H	0.70315327451629	3.17826003327610	-0.36877957326884
H	-0.82215475104954	0.90769827837427	-4.48950027404817
H	0.45921114629063	3.03399767475423	-4.64425618308993
H	-1.33520494853214	-0.06900122718104	-2.28612750961930
N	1.78114254858332	0.54003676782233	0.63639850024108
O	-1.46933162143969	-0.24765079459775	0.11864439232348
Ti	-1.98390053998410	-2.01205209240350	-0.05282946138766
Cl	-2.47542837045942	-2.32585947211764	2.31178927188102
C	0.24426288050934	-2.64957501208053	0.50983021513982
C	-3.33301935359663	-1.78854305667932	-2.04036254472554
C	-3.86237997556812	-0.84196326660093	-1.12823513386169
C	-4.38172858481341	-1.54189106931977	-0.03039854521217
C	-4.22516126420947	-2.94042838664828	-0.27922533352718
C	-3.61258039146828	-3.08835817948585	-1.53235373931800
H	-3.36812414350717	-4.02000648061897	-2.02089759836604
H	-4.51384924788631	-3.73951876757030	0.39048914961712
C	-0.51135174221402	-3.84786576694810	0.55996981358882
C	0.26293379748904	-2.20059632584766	-0.83022238561845
C	-0.52194908640007	-3.08863901353984	-1.59854811248007
C	-0.98708451315825	-4.12118920216856	-0.73302018765224
H	-0.72493605454580	-4.42513272771015	1.44767023981768
H	0.73755874411610	-1.29737533641037	-1.19064649278811
H	-0.72100190739321	-3.00797160871890	-2.65879881775360
H	-1.59926442267821	-4.96607916687382	-1.01310858220317
H	-3.77480555509200	0.23334130670549	-1.20607609892202
H	-4.79714183362259	-1.10302573752847	0.86462523437972
Ti	3.68503065344079	0.59920958642755	0.49267604012136
Cl	3.79243324982624	0.54738776917435	-1.97913058618616
C	4.85567717173778	-1.48551159837145	0.03196328928176
C	5.26502655178624	2.11381500058746	1.50413468604489
C	5.07053265905035	2.52519911249422	0.16968994284850
C	4.03485169734438	2.26416771642828	2.19556931115598
C	3.09995994750788	2.82475233542041	1.29437849894070
C	3.72616304797883	2.96877067574962	0.04061170990011
H	5.80435638292384	2.48466482708717	-0.62372048320570
H	2.07220151352188	3.06329072840329	1.52680029060373
H	3.84665240381483	2.01026638844537	3.23041175917840
C	3.55457605313274	-1.79941527665412	0.48570240766645
C	5.54035940626797	-0.82925553980764	1.07108458859704
C	4.66370468918306	-0.75441672474648	2.19273499813256
C	3.44428659051030	-1.36348466924471	1.83336105208769
H	2.78157916213953	-2.27729040625021	-0.09777781247066
H	6.55233239988466	-0.45070677659571	1.02342416065097
H	4.89360981676652	-0.30914324949865	3.15082042211384
H	3.25842838676000	3.31472554706109	-0.86989165867036
H	2.57605124240230	-1.45198910745927	2.47146881795414
H	-2.85957525672489	-1.57215260475181	-2.98928812591937
H	5.23957145539953	-1.66881197410258	-0.96050008260460
H	6.18241852032922	1.72572580726286	1.92344080618979
H	0.68732371509348	-2.14937545730263	1.35828189842485

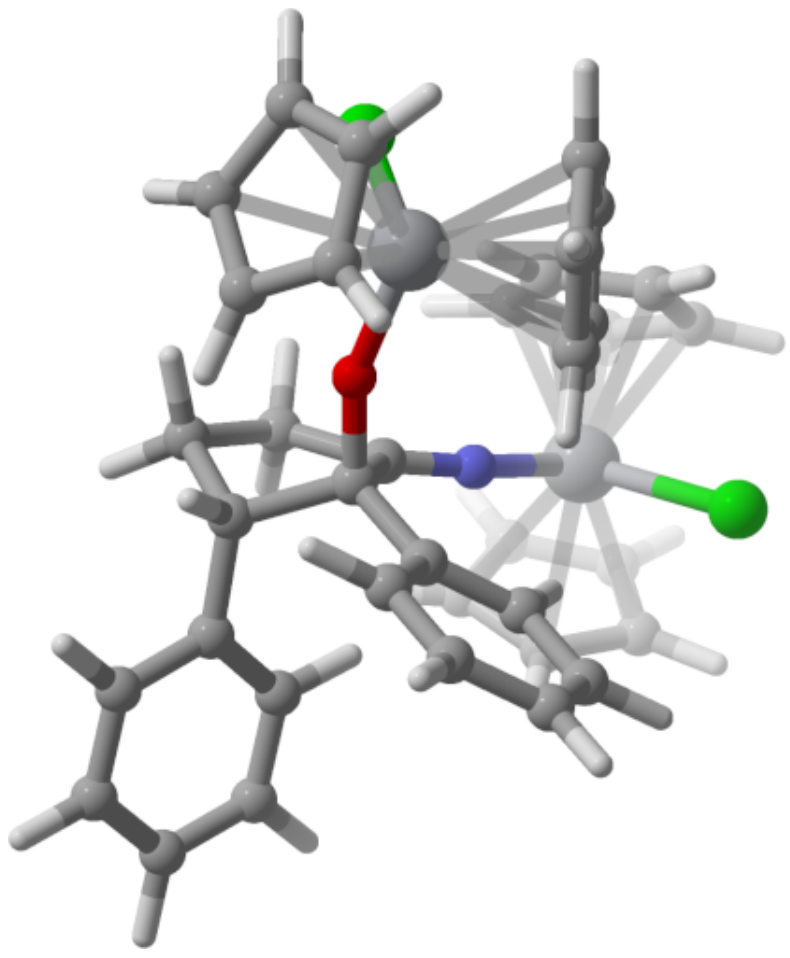

B'

C	-0.15214474875620	-0.61567718630849	-0.80624131992121
C	-1.45007018066387	-0.52108563735712	0.06584391419853
C	-0.57726120758278	-0.54965477012907	-2.26911275609916
C	-2.10822202869640	-0.49535495287921	-2.26958276796451
H	-0.20000962605515	-1.44349203033667	-2.78040807759485
H	-0.10851658625972	0.31586621405975	-2.75123333020328
C	-2.52561957123791	-1.09768031124768	-0.90641225732265
H	-2.54916792466973	-1.04584734185607	-3.10522503584264
H	-2.45232907834339	0.54209778703555	-2.32080365410271
C	-2.62051745019922	-2.60543408696054	-0.94678450060906
H	-3.50281465427070	-0.70507795785574	-0.60838962289423
C	-3.87552246878674	-3.19210536201431	-1.14560275720368
C	-1.51016937433435	-3.44935313264705	-0.83579305092578
C	-1.64858803094984	-4.83197272646177	-0.92839606583094
C	-2.90287031443752	-5.40110973822544	-1.13275264706084
C	-4.01855151079758	-4.57355616294414	-1.23922102077748
H	-4.75278879031031	-2.55330396592736	-1.22542804298515
H	-5.00473256269653	-5.00429963855651	-1.39079413299909
H	-0.77021436374622	-5.46525058984737	-0.83366420496782
H	-3.01117001950844	-6.47992831550849	-1.20146278508354
H	-0.52419118595409	-3.03387237857735	-0.65396426667856
C	-1.48435758432476	-1.16299744643879	1.44128327039722
C	-0.37810917554094	-1.72488808459059	2.07576617807174
C	-2.71015098529071	-1.14875740160652	2.12115130277380
C	-2.82440321539234	-1.67903798944937	3.40006981500662
C	-1.71035295916149	-2.23491388348562	4.02912426148880
C	-0.49095208877746	-2.25440249908702	3.36143152777163
H	0.38754284180489	-2.67982966457840	3.83945192582271
H	0.58555297118043	-1.73049625085246	1.58203757539299
H	-3.78470602369842	-1.65732571850893	3.90826017056705
H	-1.79574976662943	-2.64713273216809	5.03085203468155
H	-3.58408647907297	-0.70814024420983	1.64997536830162
N	1.03112222038354	-0.71326272383460	-0.41237966263172
O	-1.73909179346656	0.85644777688925	0.17634464396255
Cl	-0.63640832123017	3.47064308736925	-1.20394821119457
H	-2.89007067055801	5.21130960353498	-0.32457814788631
H	-1.15947369415430	0.84914651548765	3.28529974896491
H	-3.73370048959255	2.93087336339881	-1.48233391605577
C	-3.26234578830049	4.33880308934149	0.19396111896631
C	-0.72005783652547	1.73615217742782	2.84802840995300
C	-3.71783402793183	3.13317901463346	-0.42008358464238
H	0.83894365299159	0.89954610068452	1.48484806901553
Ti	-1.67288248916704	2.57818552924460	0.81714144496518
C	0.34056643901453	1.76323880161867	1.90453684610511
C	-1.10938551210586	3.06979627761114	3.10470887076612
C	-3.34805674630290	4.18020411793885	1.58323059858875
H	-1.88491716532746	3.37839267115914	3.79101120772445
H	-3.06573200051321	4.91510022785822	2.32379896672645
C	-4.09382122868402	2.24551205400822	0.59885512348204
C	0.57841232751044	3.10654033954285	1.56165416498489
C	-3.81576582882458	2.85728027032363	1.84466532777296

C	-0.33313216480708	3.92124483867403	2.28296626510334
H	-4.44197868531710	1.23195902684664	0.46109917265479
H	1.29941337748155	3.46346550609906	0.84125199314377
H	-0.41414706728124	4.99847376745462	2.21653636894748
H	-3.97877554425612	2.41644386572189	2.81959097677650
Cl	3.26728967639942	-0.86781186089786	2.00991607197188
H	4.30221942386456	-3.28573231726290	0.96764578372990
H	1.70173742826260	-3.58394185043243	0.38358115190573
C	3.84278706422189	-3.09031095728862	0.01021901486072
C	2.56272903483767	1.42230993464760	-1.09820435671354
C	2.47046509641044	-3.24130658082637	-0.29671906938192
H	3.29719206492445	1.84523696283441	0.95610968017392
Ti	2.93447126096440	-0.88158597007576	-0.43505679570814
C	3.47655413680433	1.43940231776750	-0.02896298405029
C	3.16535902798987	0.73020673991070	-2.18053555589565
C	4.49452899314015	-2.56867443199600	-1.12417115894535
H	2.70538738270918	0.52444860423288	-3.13845204210559
H	5.54517071728414	-2.32053663393405	-1.19474768561296
C	2.28078310195220	-2.84825308000956	-1.64766794066327
C	4.66016546481138	0.77098281464197	-0.44548800679100
C	3.52295690290315	-2.42044736969529	-2.15648095734532
C	4.47638796598724	0.36572921615670	-1.78553365857274
H	1.34792022074671	-2.86056431603997	-2.19294406700233
H	5.53790192775577	0.59273867216483	0.16160968055869
H	5.19761900509192	-0.16326115142061	-2.39241933359067
H	3.70377006637700	-2.04687615544255	-3.15499210204450
H	1.55915721668576	1.82683631345193	-1.08801651237807

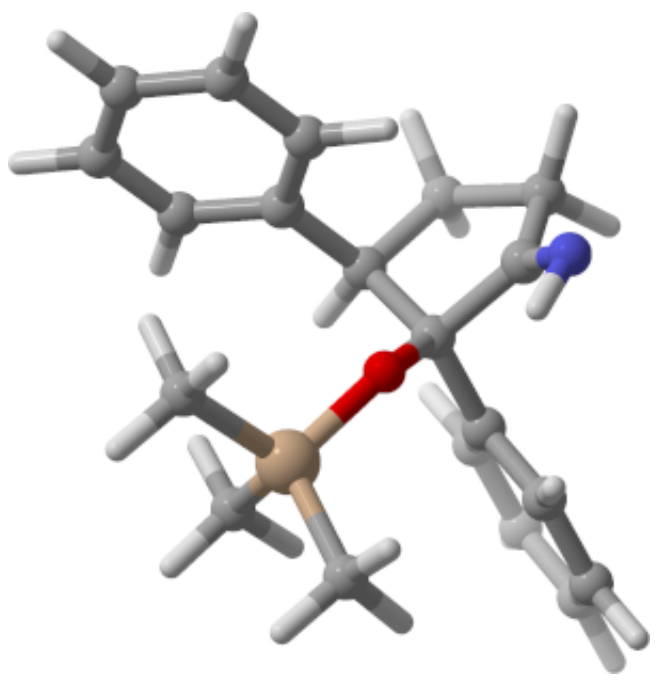

4

C	0.52203296071225	-0.28245760920369	2.11042584157475
C	0.33616420355132	0.19694178169325	0.65853146394187
C	-0.29694099524729	0.60326312658287	3.00594055596555
C	-1.38248687123190	1.22344276728252	2.09283549567204
H	0.34981476738790	1.37620799536095	3.43967204031178
H	-0.70716250055196	0.02389253624846	3.83755752743911
C	-1.14882993531902	0.66588084632068	0.65237703347843
H	-1.31761366920736	2.31445623352773	2.09572057107700
H	-2.38614334810515	0.95820018217176	2.43473388349657
C	-2.12417239776445	-0.43323512443843	0.29756078613748
H	-1.28270673437284	1.45888794784726	-0.08920210168555
C	-2.17181874043017	-1.64748319407145	0.99293816000159
C	-3.04526845991958	-0.22448494348229	-0.73192098205088
C	-3.98132463611473	-1.20005917794976	-1.07107467680752
C	-4.00847518227176	-2.40833779856211	-0.38197629966361
C	-3.10035827463983	-2.62592024190595	0.65373764544124
H	-1.47369954279617	-1.84157989495753	1.80252122320669
H	-3.11513116655732	-3.56464317856607	1.20123561454196
H	-4.68642878510881	-1.01416397681009	-1.87693662800124
H	-4.73229011172854	-3.17455399739523	-0.64561629238845
H	-3.03064508860678	0.71677071337753	-1.27705732241840
C	1.33599204267487	1.34643174703663	0.45531975803437
C	2.69461490178086	1.01232900859368	0.38667076772728
C	0.97520615885513	2.69043442746611	0.34711610010579
C	1.94628527137356	3.67248415875580	0.15184133988968
C	3.29070225759841	3.32788763440486	0.06878566215967
C	3.66196740573932	1.98916018049033	0.18863896763294
H	4.70923218954184	1.70529577495387	0.12986012111770

H	2.99339413336307	-0.02807765787977	0.47702664803513
H	1.64277035106769	4.71184303517192	0.06095927692862
H	4.04488765638257	4.09440743327814	-0.08605169353486
H	-0.06359280655766	2.99742754992271	0.39666048577544
N	1.19564413205900	-1.28692099773823	2.49221747133361
O	0.63080242254071	-0.87487717354324	-0.20508284836476
H	1.61641186836657	-1.73815963330290	1.67374321148812
Si	0.72446649599428	-0.94260000120682	-1.88156564802651
C	-0.36528822403259	-2.37423888795731	-2.39145670673944
H	-0.04127550846174	-3.29910807705371	-1.89990560232040
H	-0.31758679622025	-2.53304084018085	-3.47526732889590
H	-1.40881662440448	-2.18911078083416	-2.11329360314140
C	2.50389086414998	-1.31271031633485	-2.33669839952486
H	2.58179326792375	-1.55550042363008	-3.40325568926989
H	2.88046853954141	-2.17101239052157	-1.76796216265856
H	3.15600217823640	-0.45621738443002	-2.13398225993685
C	0.17670255991955	0.65363378629431	-2.69456149643637
H	0.26070737898027	0.54018200925335	-3.78268765584899
H	0.80693467399996	1.49746426202733	-2.39407037849362
H	-0.86483377709029	0.90064272879405	-2.46546475870638

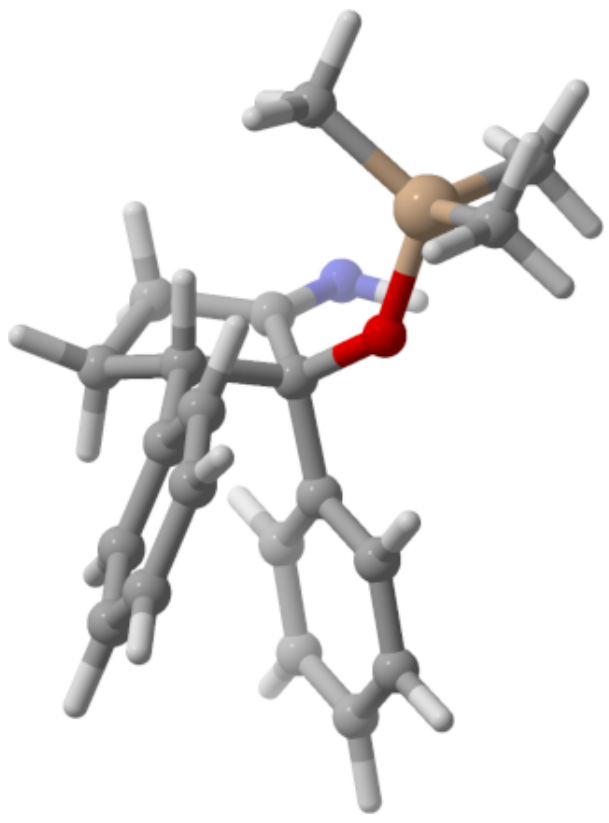

4'

C	1.31791901216766	0.77010113059556	-1.53413994514321
C	0.23492008305943	0.33571293259715	-0.52483963335934
C	0.72799877059734	1.74931042142453	-2.51997531879147
C	-0.78253821594905	1.73551283480174	-2.24071820771886
H	1.00176934026588	1.49797626403716	-3.54954759694394
H	1.14820999908196	2.74202725298817	-2.30723420470789
C	-0.86454052498180	1.44111202577578	-0.73754261025112
H	-1.26653974947390	0.94386516096384	-2.82164883584160
H	-1.27142741730078	2.68078590237899	-2.49251741139145
C	-2.21167671239591	1.11973615680777	-0.15482502904262
H	-0.47567196404563	2.32307731729550	-0.21203746248828
C	-2.48932080588408	1.49727411755440	1.16314470440392
C	-3.18933921505428	0.41291833336645	-0.86297848878103
C	-4.40402383145804	0.08714440997925	-0.26604296340240
C	-4.66267646656954	0.45699213794601	1.05222455690754
C	-3.69953136644302	1.16590823929085	1.76642605220628
H	-1.74294131347040	2.05809883113017	1.72128989453923
H	-3.89253636841146	1.46738132471214	2.79258672059053
H	-5.15053651510798	-0.46261163864044	-0.83318686458585
H	-5.61053816516152	0.19986892551095	1.51705569888049
H	-3.00155854374120	0.09926689192140	-1.88586151640439
C	-0.30179511147654	-1.04721426155039	-0.87960948331078
C	-0.24195761432205	-1.55260240175706	-2.18049630019392
C	-0.93128654661157	-1.80931931613907	0.10985103252789
C	-1.49338720911831	-3.04328533407961	-0.19750898041813
C	-1.44211200033623	-3.53487512780820	-1.50074878607040
C	-0.81274975803210	-2.78610794461153	-2.49008653740701
H	-0.75413841779642	-3.16352267497385	-3.50726557993705
H	0.26135303831683	-0.99623359380276	-2.96646742289860
H	-1.97784822807096	-3.62197809869024	0.58444115732287
H	-1.88290448929064	-4.49858143845061	-1.74066850749210
H	-0.98222970886686	-1.42618196608941	1.12381680334104

N	2.52824077837458	0.38819256163164	-1.54203503739739
O	0.70101587207306	0.28448807422236	0.80640149296830
H	2.69132162595041	-0.27541821675699	-0.77951073013815
Si	1.66308130765278	1.28485185599230	1.74996639146175
C	3.43499185665302	0.67060669615778	1.74124944436070
H	3.98200666928263	1.12506926617056	2.57646722919921
H	3.96366810920775	0.92999363195395	0.81808249130734
H	3.47436772868829	-0.41742138403535	1.86924955845300
C	1.62544664360010	3.08250300283103	1.21601107919421
H	0.64488847837943	3.54563205107312	1.37019265421663
H	1.91175510825840	3.21186394075605	0.16619230504147
H	2.35312109411687	3.63453426614609	1.82444041894585
C	0.95468521077136	1.08646453028855	3.46957622803281
H	-0.08080935119480	1.44069478355117	3.51969971017459
H	1.54576408619344	1.65533403393215	4.19677658861022
H	0.96837079787391	0.03356409160099	3.77411124143112

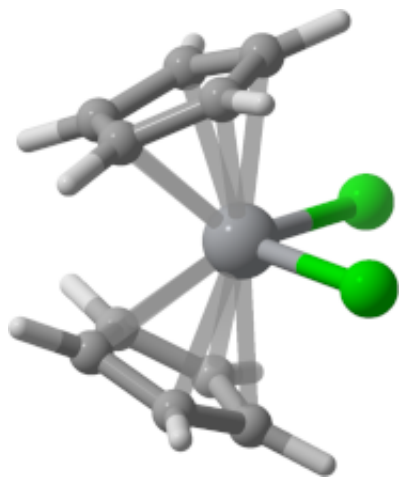

Cp_2TiCl_2

C	-0.15122905641618	2.41532552496160	-0.01331496739331
C	-1.06588561295372	1.92288224594333	0.95720315883491
C	-0.32458376605035	1.29042227272129	1.97362514489475
C	1.05322564593721	1.36765593667926	1.62422385522025
H	-2.14409379143633	1.98909813179011	0.90608163527829
C	1.15108311000530	2.09614117557605	0.41575514526886
H	-0.41258640392740	2.91182031390251	-0.93749143984017
H	2.06571858206812	2.31472497529470	-0.11518823328484
Ti	-0.00209498062470	0.00067913349555	-0.00461530954607
C	-0.94764961497802	-1.60247156127901	1.46808802492793
C	-1.13002246141804	-2.14325364478824	0.17380254302605
C	0.45091619582885	-1.56852385720177	1.73085484954504
C	1.11927126142548	-2.03613823947149	0.58305840008672
C	0.13889700019456	-2.38512126542097	-0.38561218107319
H	0.91706300347212	-1.22640426488907	2.64398469154309
H	-1.73360116114821	-1.29523162286358	2.14503411999611
H	-2.07942974419507	-2.28713862168014	-0.32032259682345
H	2.19128151737748	-2.08617404589778	0.44953817885008
H	0.33515324717197	-2.73628137387042	-1.38912857521824
Cl	1.68076814118372	0.00459383945766	-1.68647282611510
H	-0.73056235941232	0.81456566379010	2.85509166940336
H	1.88114101913124	0.96565814684359	2.19266658708496
Cl	-1.80317584594634	0.24067527193434	-1.54043040902814

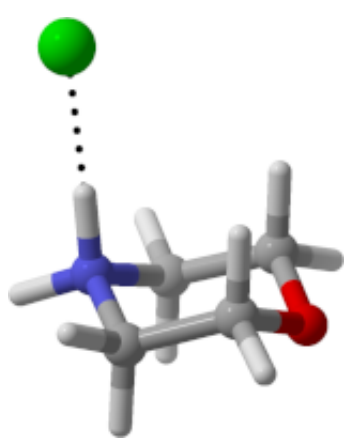

morph•HCl

C	-2.00385088335433	1.76418411004414	0.39000692692873
C	-3.03959014179892	0.76657545593904	-0.09700677709654
O	-0.68546707711392	1.36006786994983	0.01516283238190
H	-2.06808399862052	1.87729546296868	1.48419743034588
H	-2.19071901363726	2.73504923070521	-0.07677418655995
C	-0.36330275293111	0.10903910619966	0.62593011750828
C	-1.30215714818466	-0.98666256025989	0.15341320964387
H	-0.40578318982092	0.20191169475932	1.72303272355285

H	0.65966791823358	-0.14134694045758	0.33232624905205
N	-2.71481915725458	-0.58698814781459	0.46170962686010
H	-1.09684824143957	-1.92984302843228	0.66881182492769
H	-1.22293233109259	-1.13154780279288	-0.92892906043854
H	-3.02866037271375	0.69095698593263	-1.18919412193185
H	-4.04187940074736	1.04267915154010	0.24432566979844
H	-3.36350110176343	-1.28234159278232	0.08976825103949
H	-2.86058923645892	-0.57801292217749	1.52022330626717
Cl	-3.18773387130172	-0.58320607332161	3.48831597772043

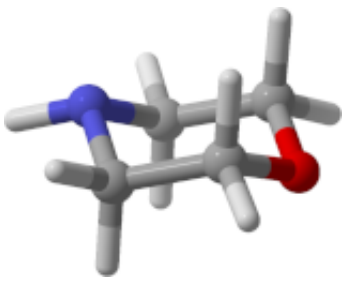

morph (= morpholine)

C	-2.02027674726860	1.75240944045910	0.42653708370347
C	-3.03360501576499	0.71254648471608	-0.02081272546684
O	-0.68783509758351	1.36416967620867	0.06588745011555
H	-2.08147217904651	1.88778216554319	1.51990066787101
H	-2.20644198704911	2.71351247118593	-0.06335549556058
C	-0.36279418079455	0.09441986054622	0.64739074805641
C	-1.34349621828393	-0.97768281470335	0.20346805779091
H	-0.37443473161890	0.17911908246002	1.74752881551510
H	0.65312507212922	-0.14646620934981	0.31847980410282
N	-2.69967059684848	-0.57512327337300	0.60633272785662
H	-1.08491711672576	-1.92863756799716	0.68613164847905
H	-1.25136984910595	-1.11112833751227	-0.89159867055381
H	-3.02103183531937	0.65823780180426	-1.12650084297272
H	-4.03740164929925	1.02459026061416	0.29392775949677
H	-3.36178786742030	-1.27839904060203	0.29426297156623

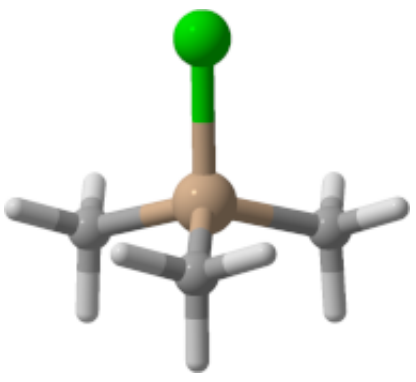

TMSCl

Si	0.45840042711056	-0.00111991474289	0.00005496122286
C	0.99094363626093	-1.78494880914638	-0.11603823646163
H	0.62186709715470	-2.36319707398905	0.73755356457849
H	2.08623850200644	-1.84410525706981	-0.12122322783831
H	0.61961391449439	-2.24899883707475	-1.03579609242603
C	0.98595038859890	0.99201822347871	-1.48810375477547
H	2.08112211287136	1.02744036719561	-1.53928649515171
H	0.61361561337588	2.02015796666676	-1.42856008751701
H	0.61513834808641	0.54204626405424	-2.41504362577222
C	0.99047657377721	0.79190576137194	1.60226708777693
H	0.62160150133316	0.22629790434574	2.46435321855516
H	0.61870533559615	1.81926314663028	1.67665570017918
H	2.08580008209872	0.81991780373546	1.65480111960429
Cl	-1.66825774046481	-0.00366876405585	0.00303901212547

NMR Spectra

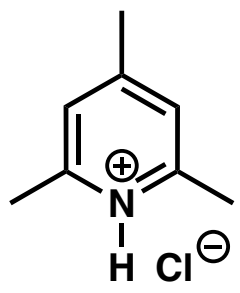

Coll·HCl

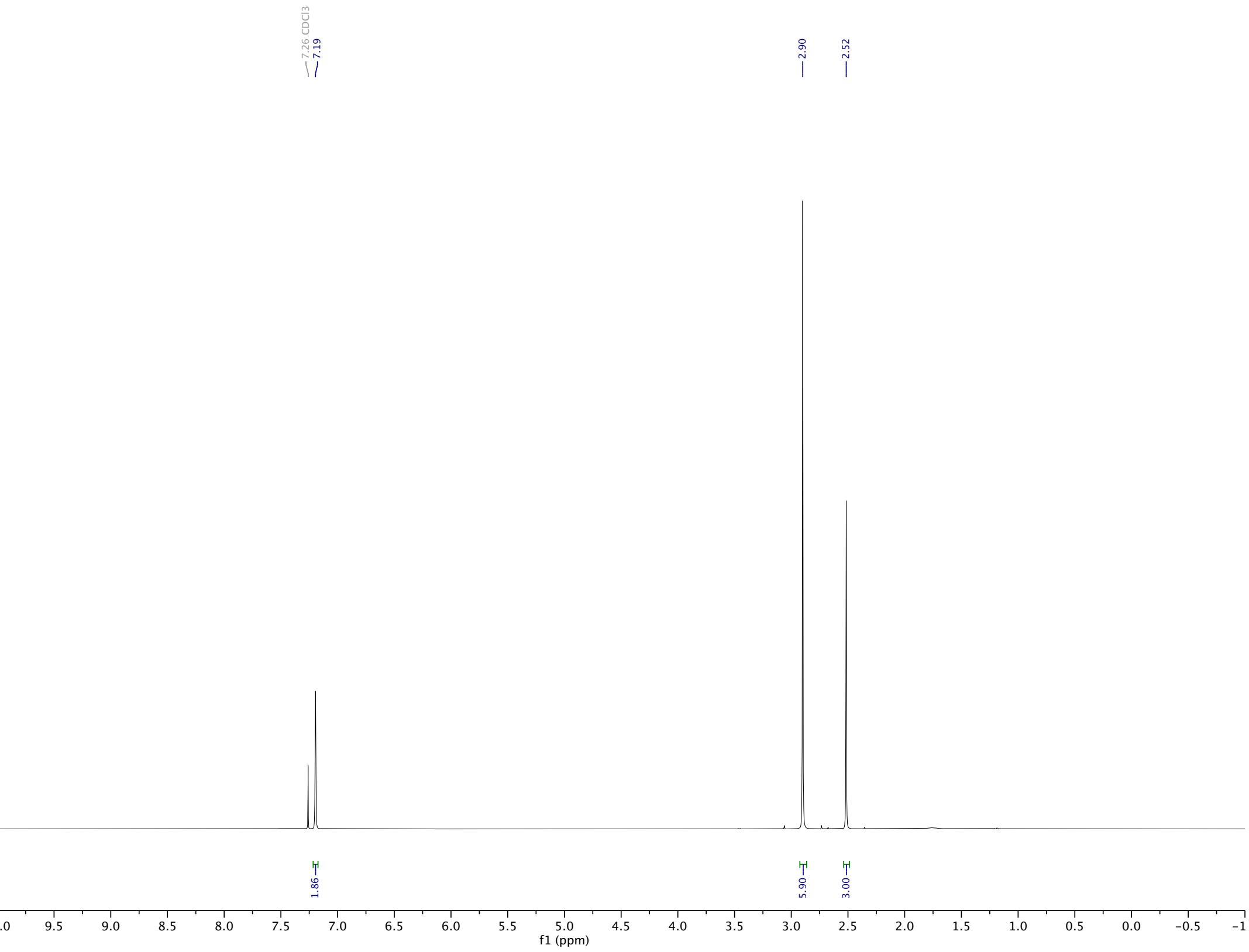

^{1}H NMR (400 MHz, $CDCl_3$) spectrum of 2,4,6-collidine hydrochloride.

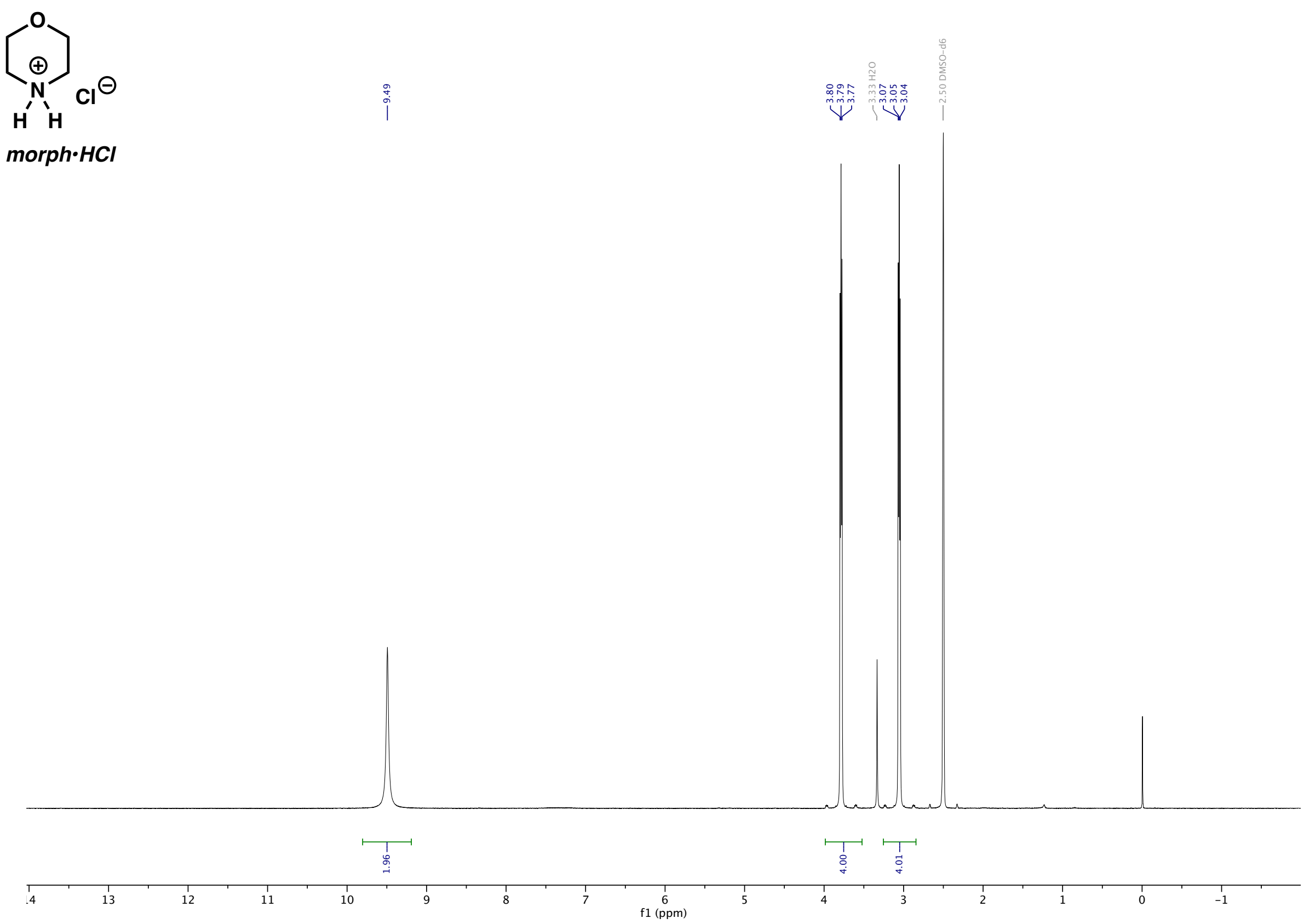

^1H NMR (400 MHz, DMSO-d$_6$) spectrum of morpholine hydrochloride.

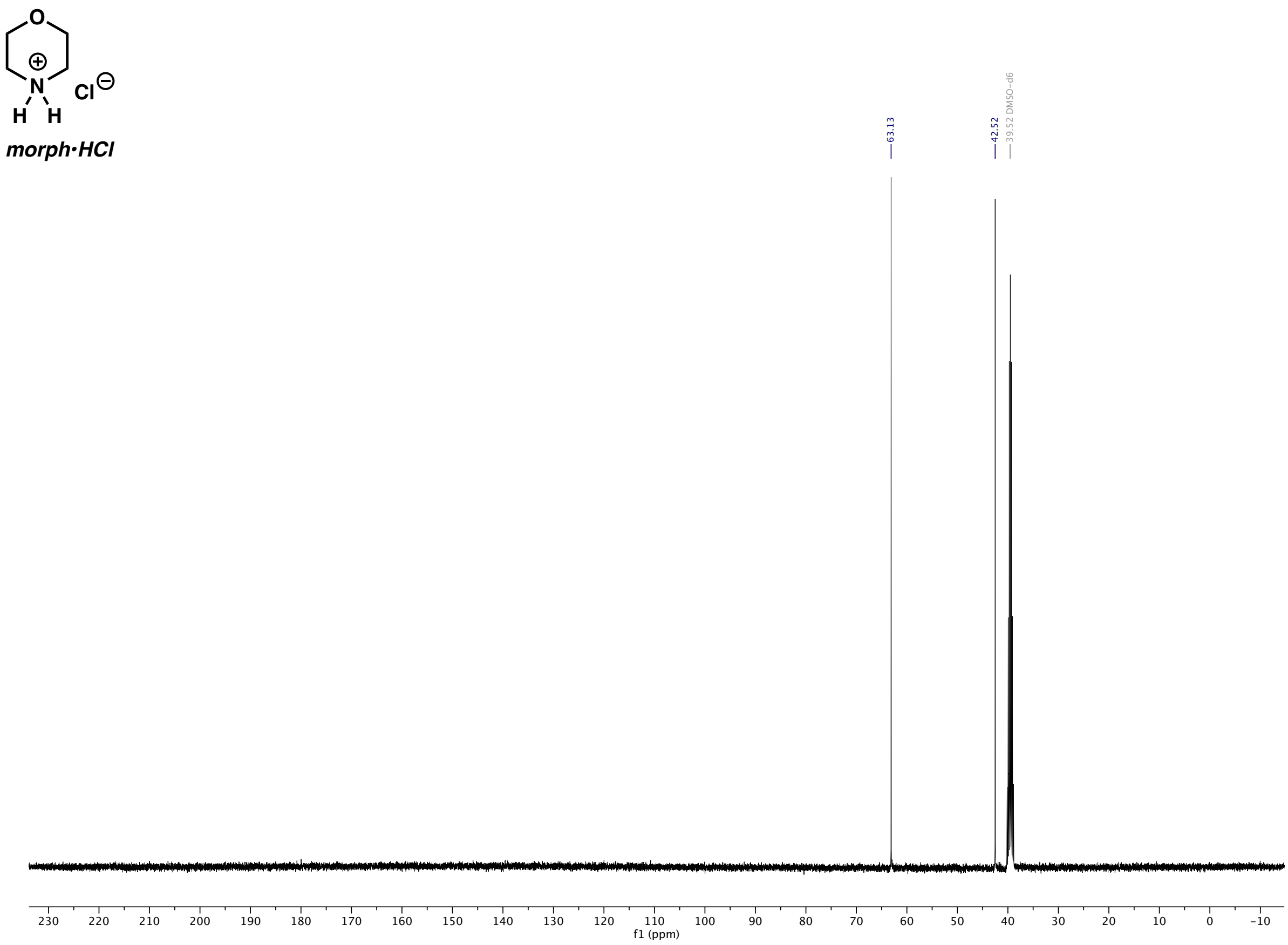

^{13}C NMR (101 MHz, DMSO-d$_6$) spectrum of morpholine hydrochloride.

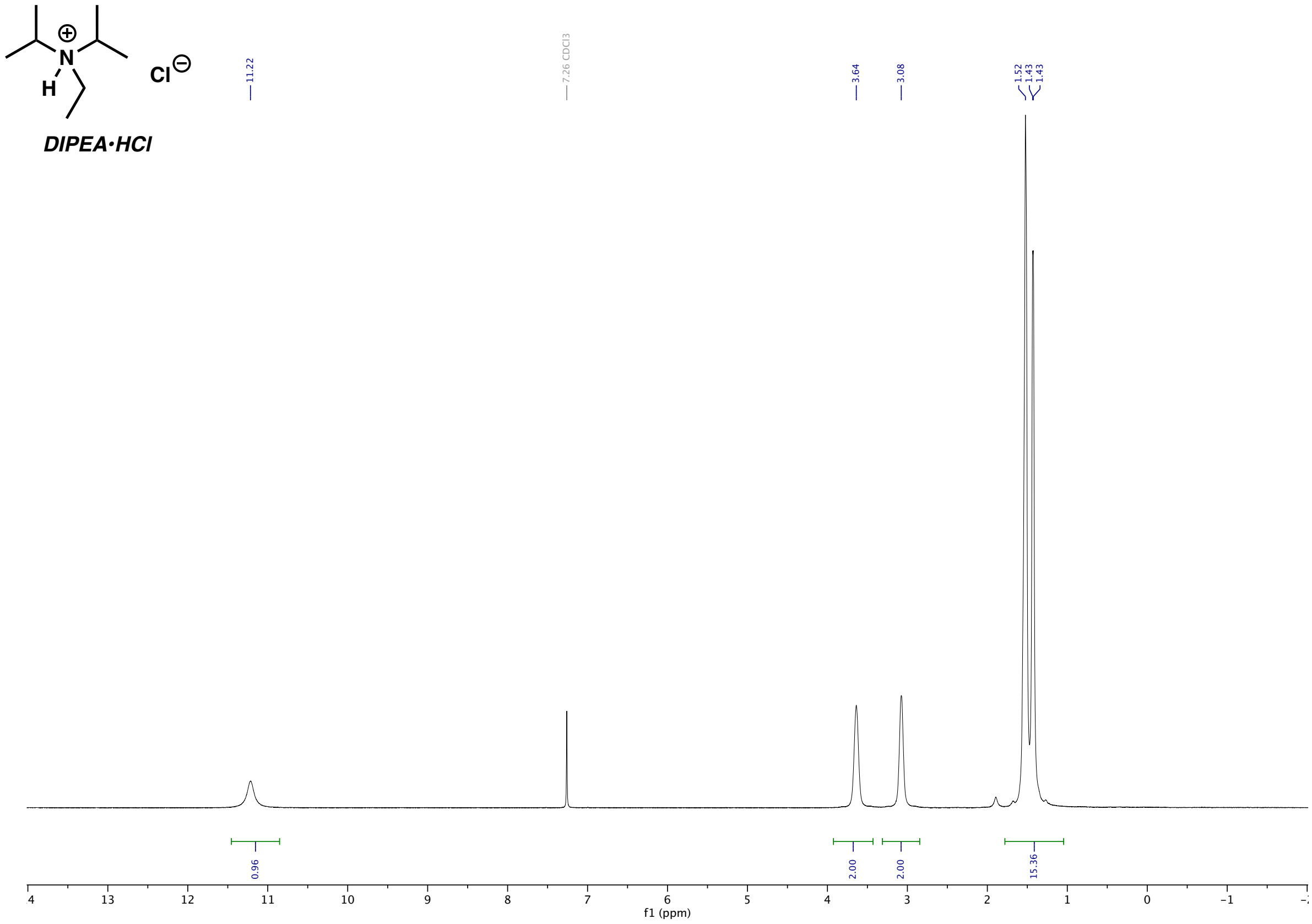

^{1}H NMR (400 MHz, $CDCl_3$) spectrum of DIPEA•HCl.

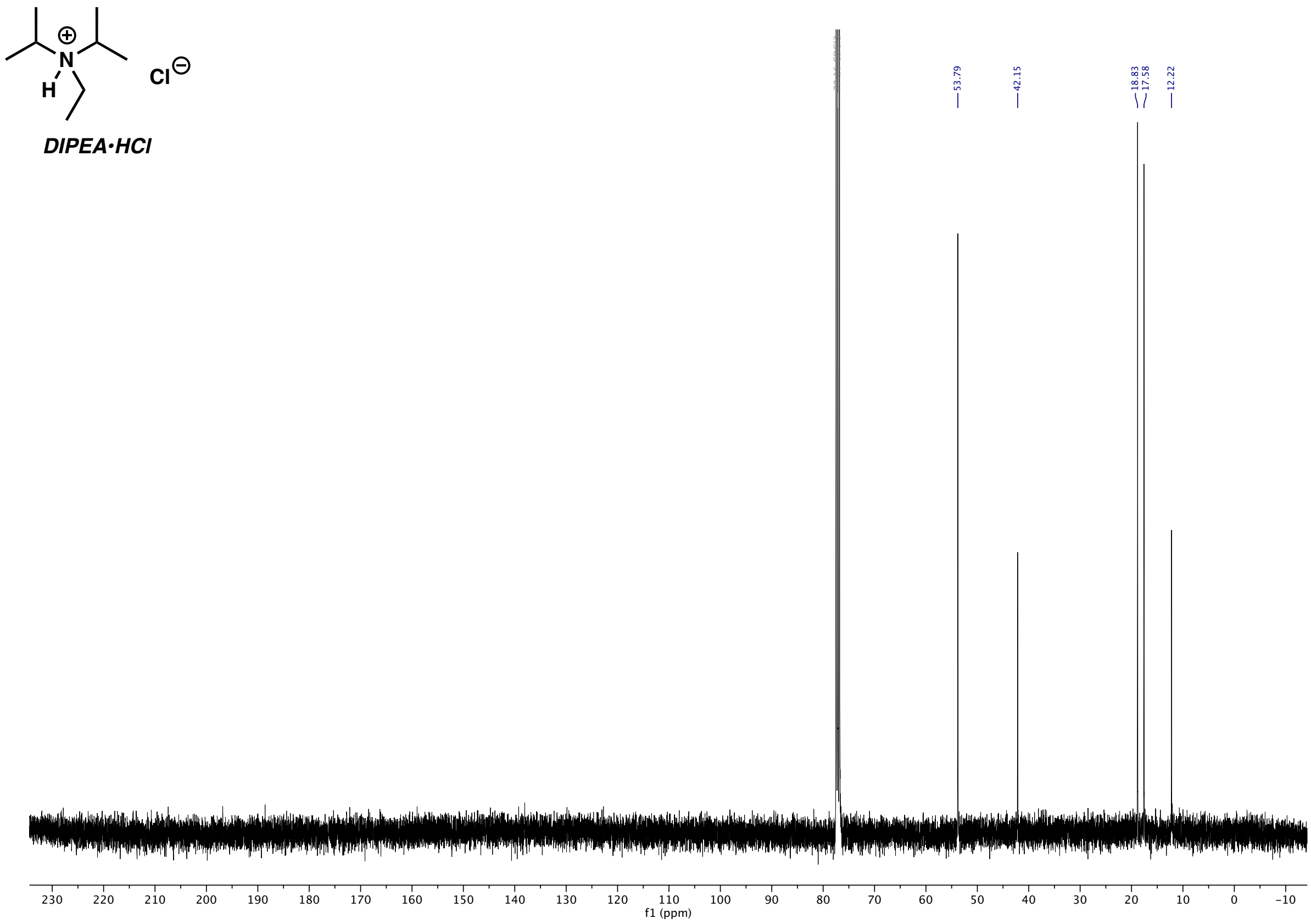

^{13}C NMR (101 MHz, $CDCl_3$) spectrum of DIPEA•HCl.

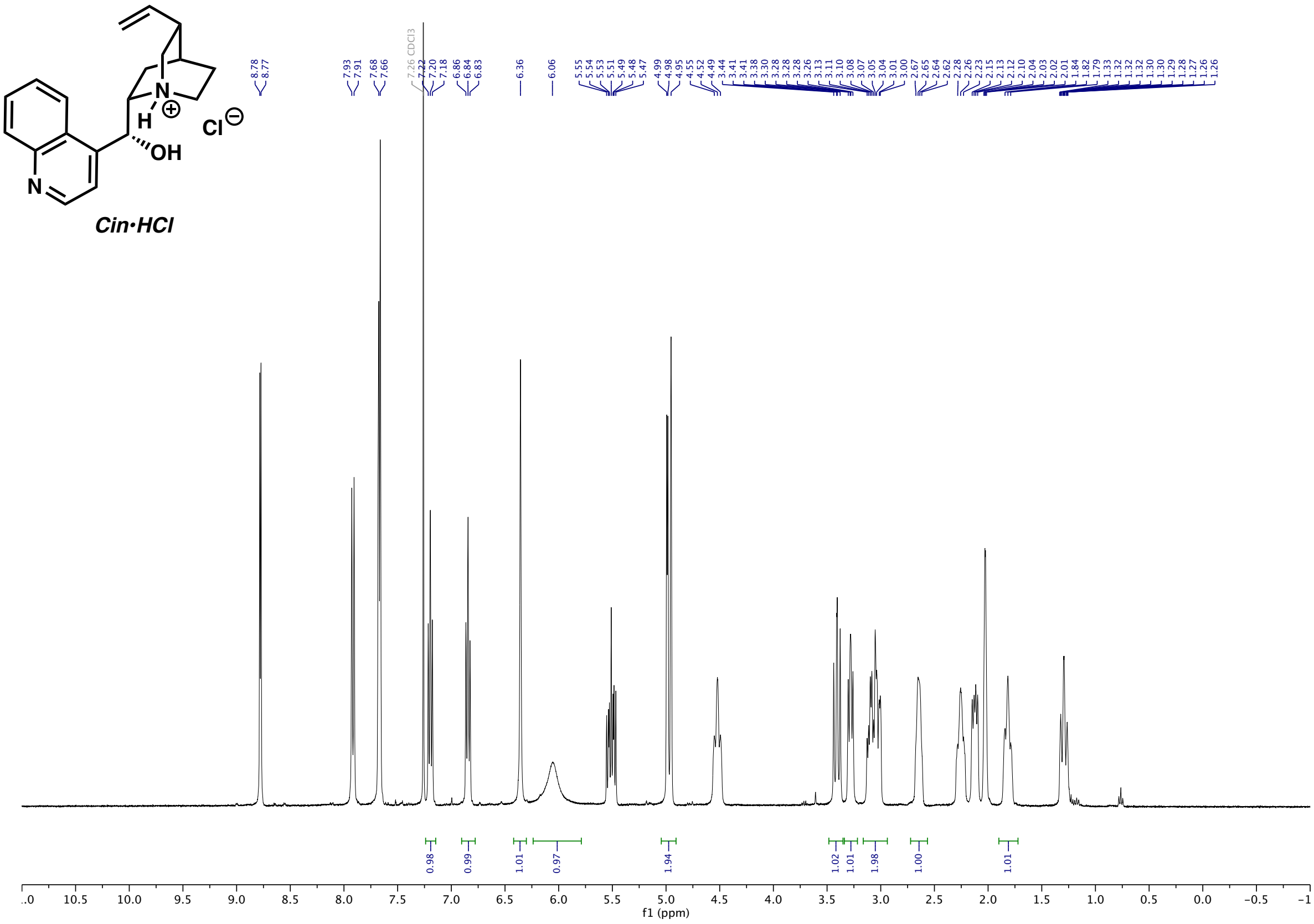

^{1}H NMR (400 MHz, $CDCl_3$) spectrum of Cin•HCl.

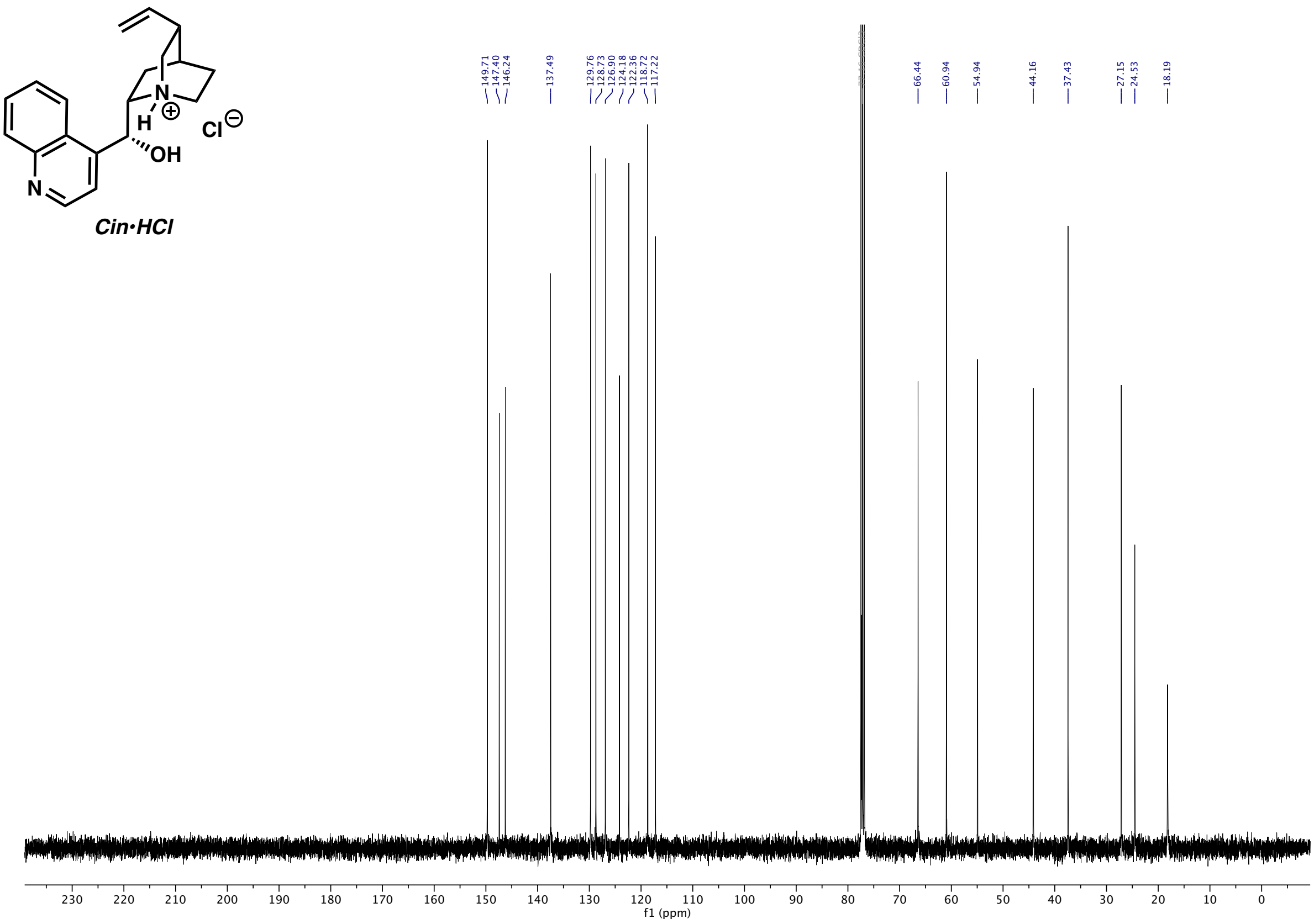

^{13}C NMR (101 MHz, $CDCl_3$) spectrum of Cin•HCl.

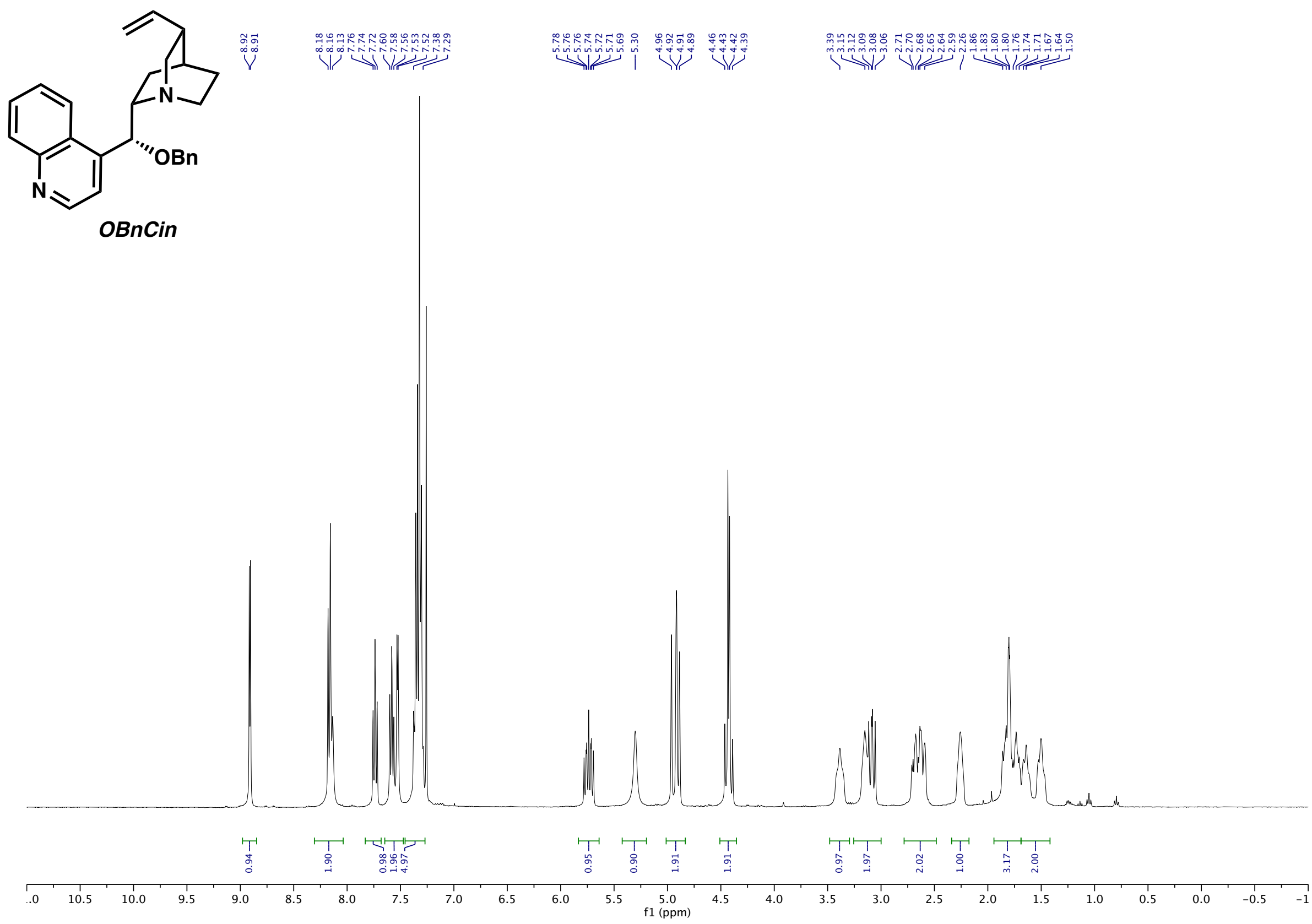

^{1}H NMR (400 MHz, $CDCl_3$) spectrum of OBnCin.

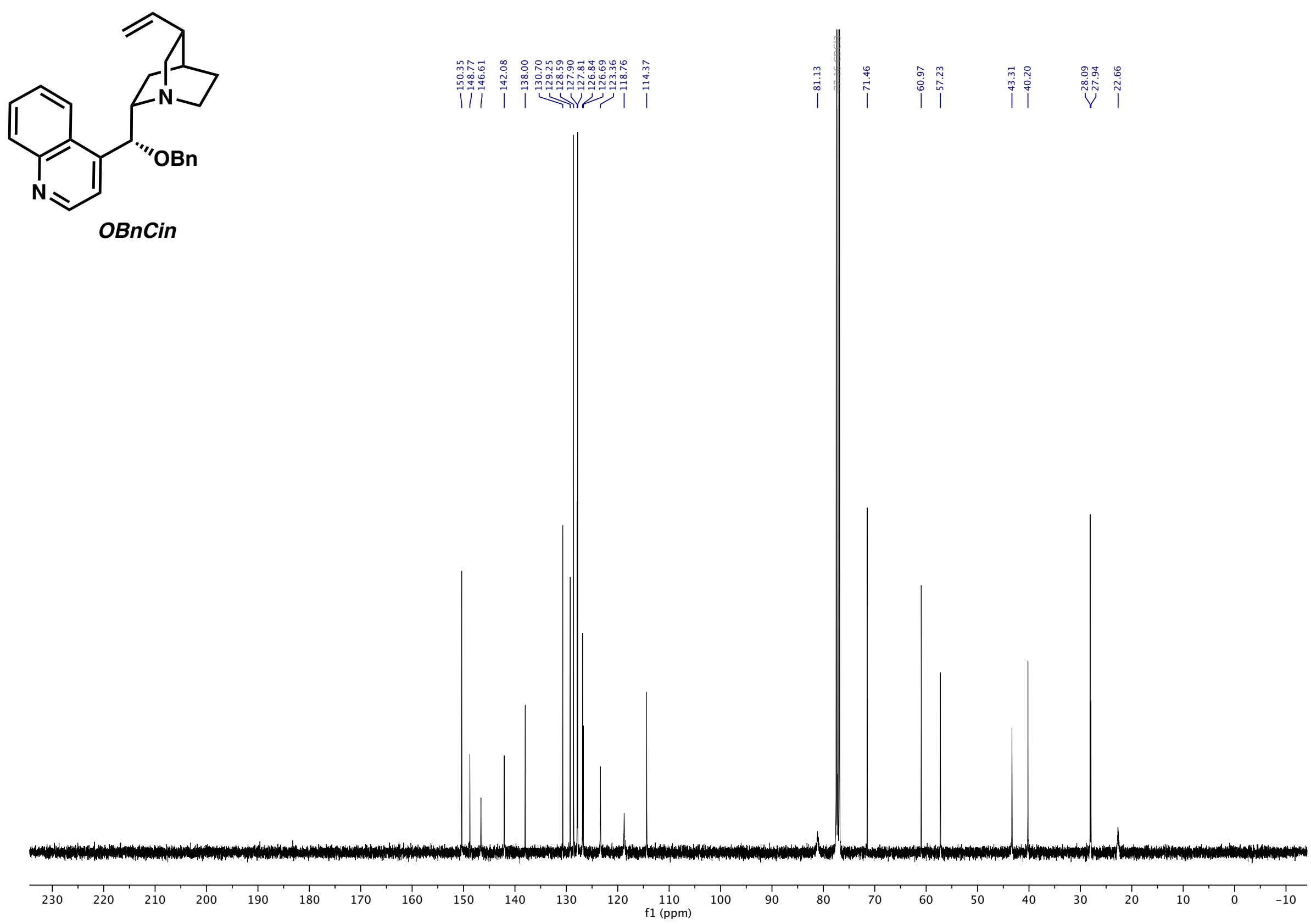

^{13}C NMR (101 MHz, $CDCl_3$) spectrum of OBnCin.

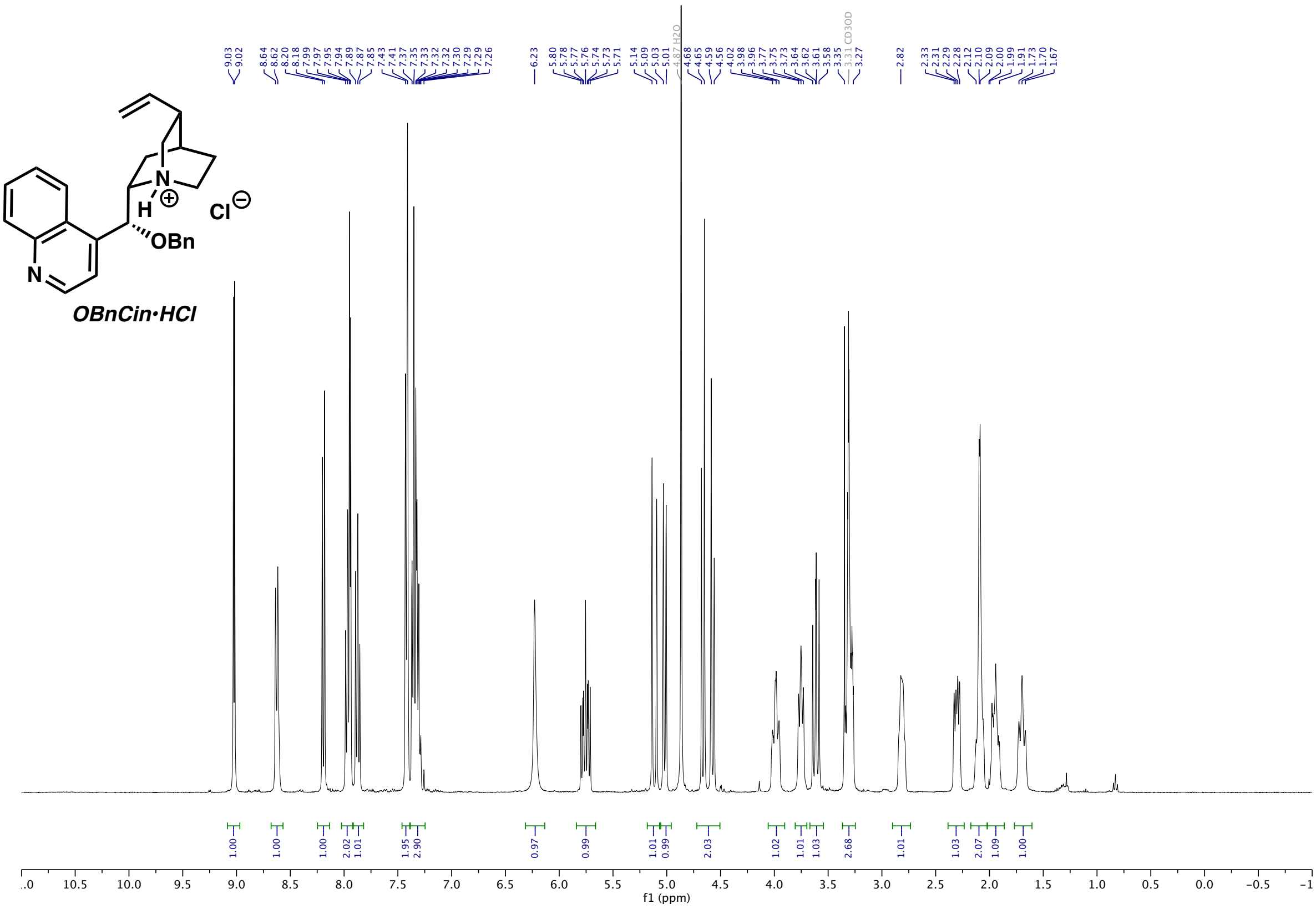

^{1}H NMR (400 MHz, MeOH-d_4) spectrum of OBnCin•HCl.

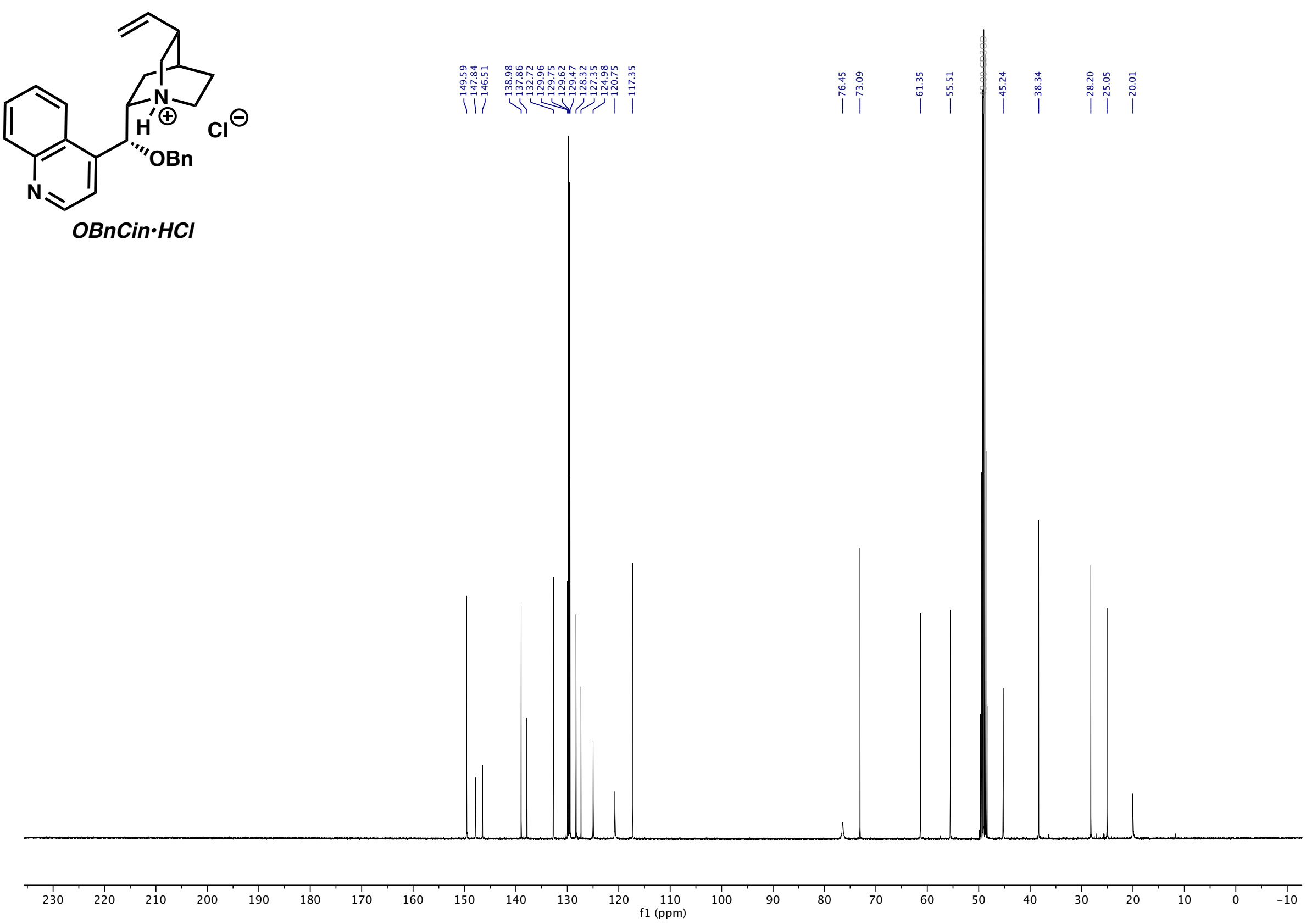

^{13}C NMR (101 MHz, MeOH-d_4) spectrum of OBnCin•HCl.

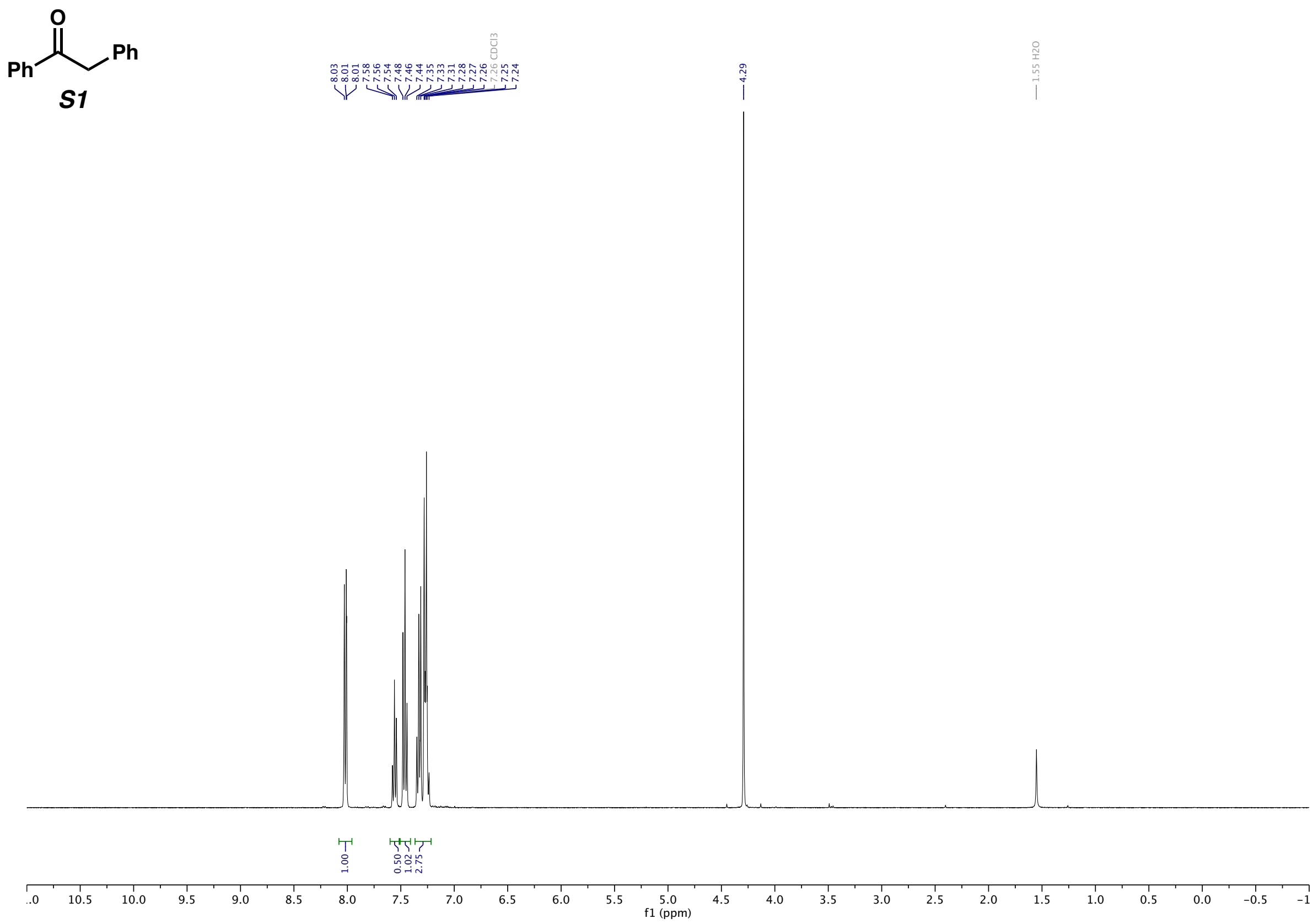

^{1}H NMR (400 MHz, $CDCl_3$) spectrum of compound **S1**.

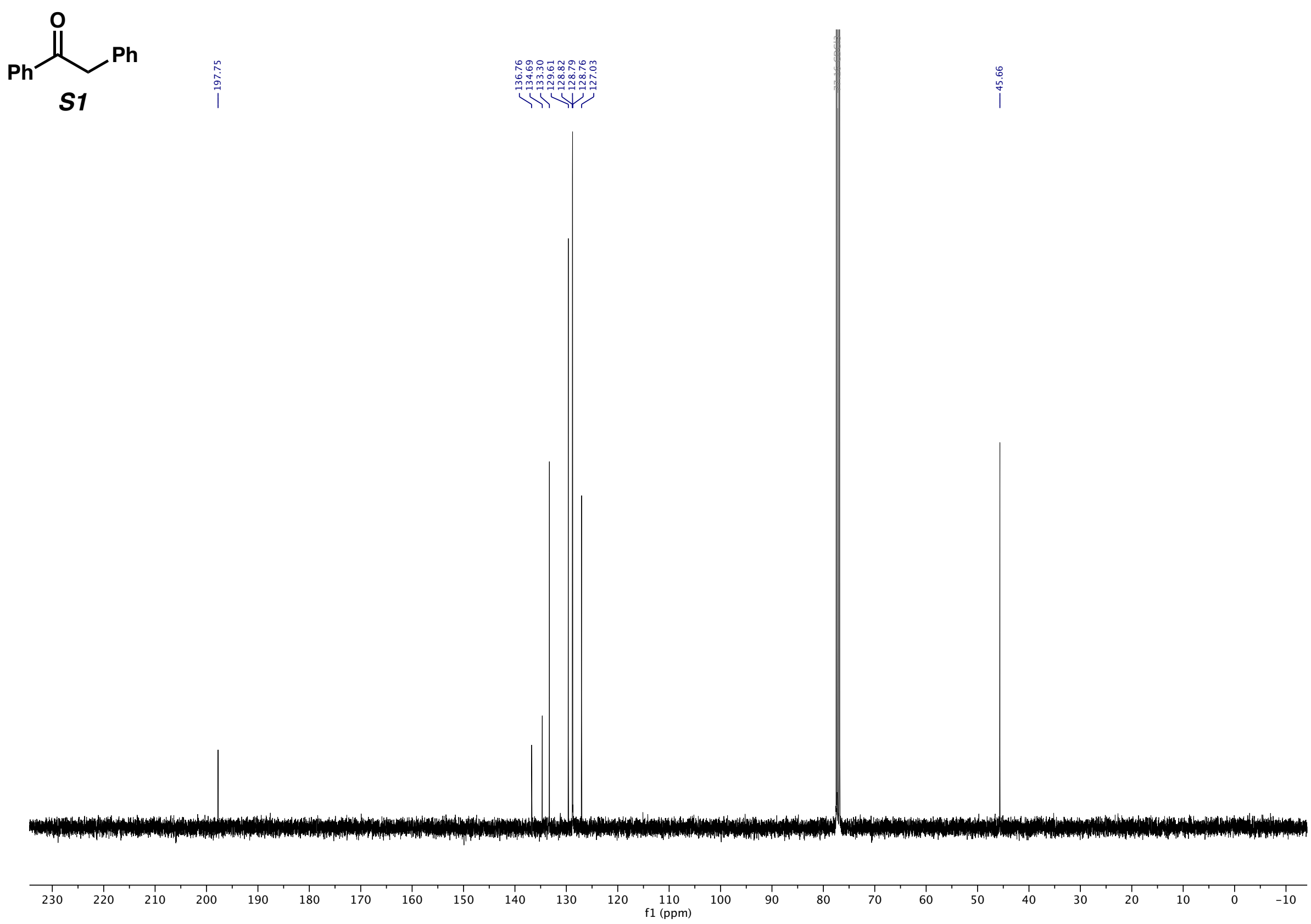

^{13}C NMR (101 MHz, $CDCl_3$) spectrum of compound **S1**.

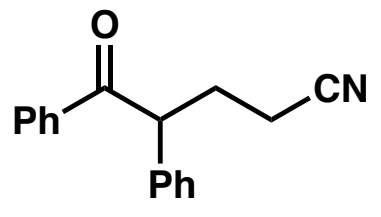

1

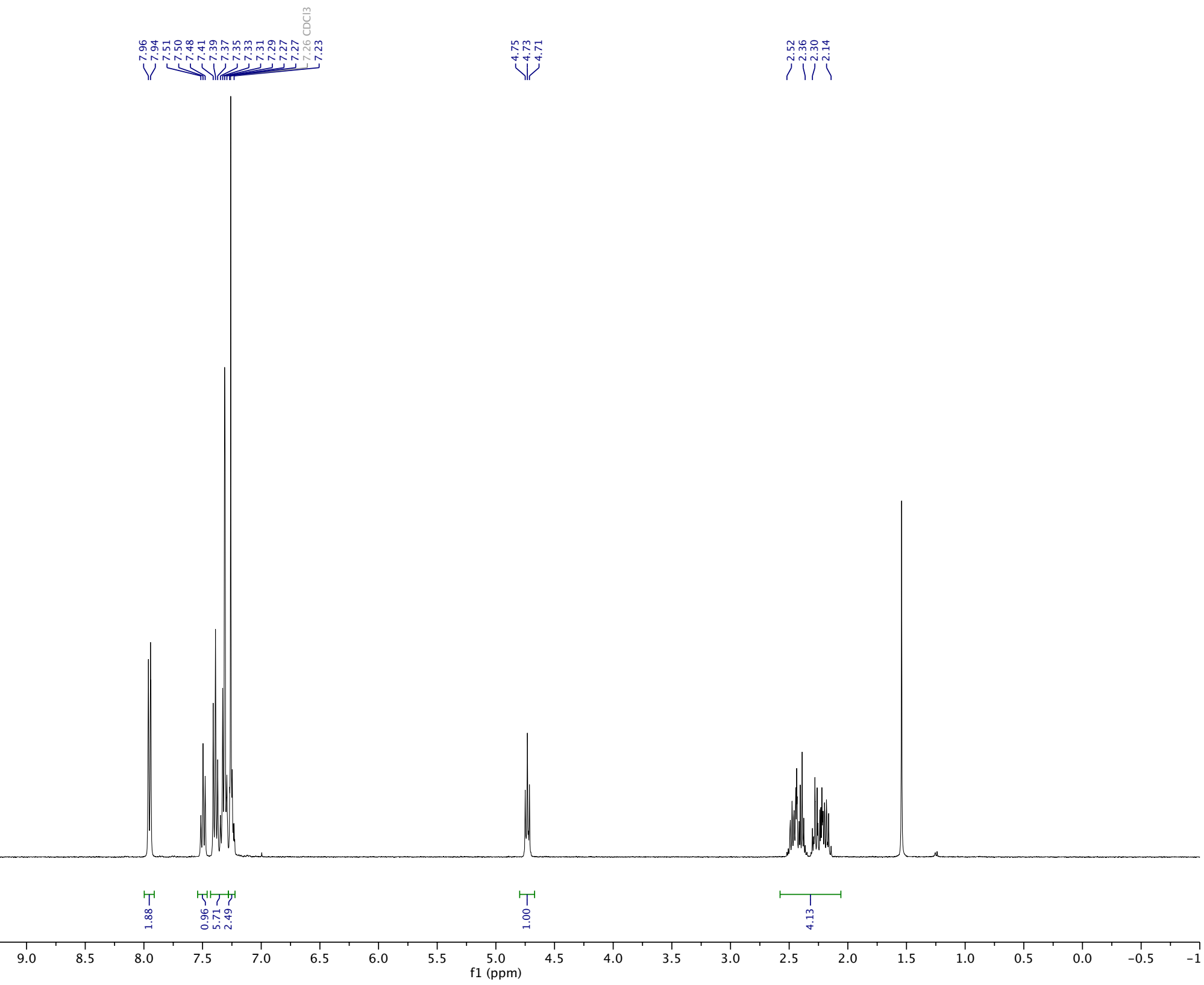

^{1}H NMR (400 MHz, $CDCl_3$) spectrum of compound **1**.

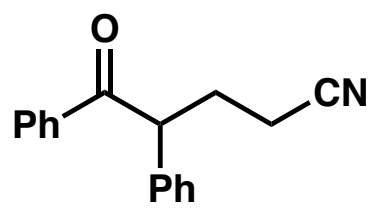

1

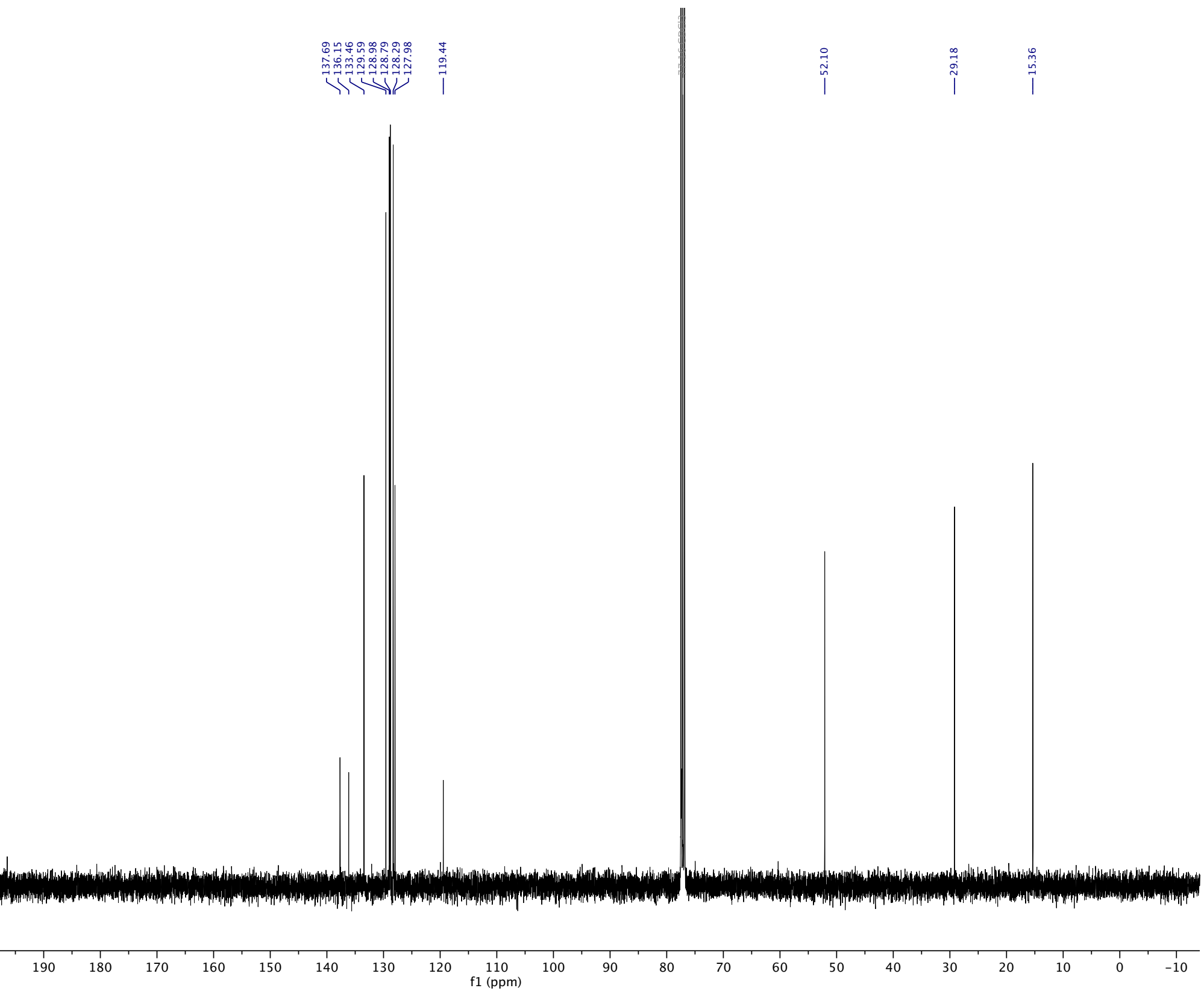

^{13}C NMR (101 MHz, $CDCl_3$) spectrum of compound **1**.

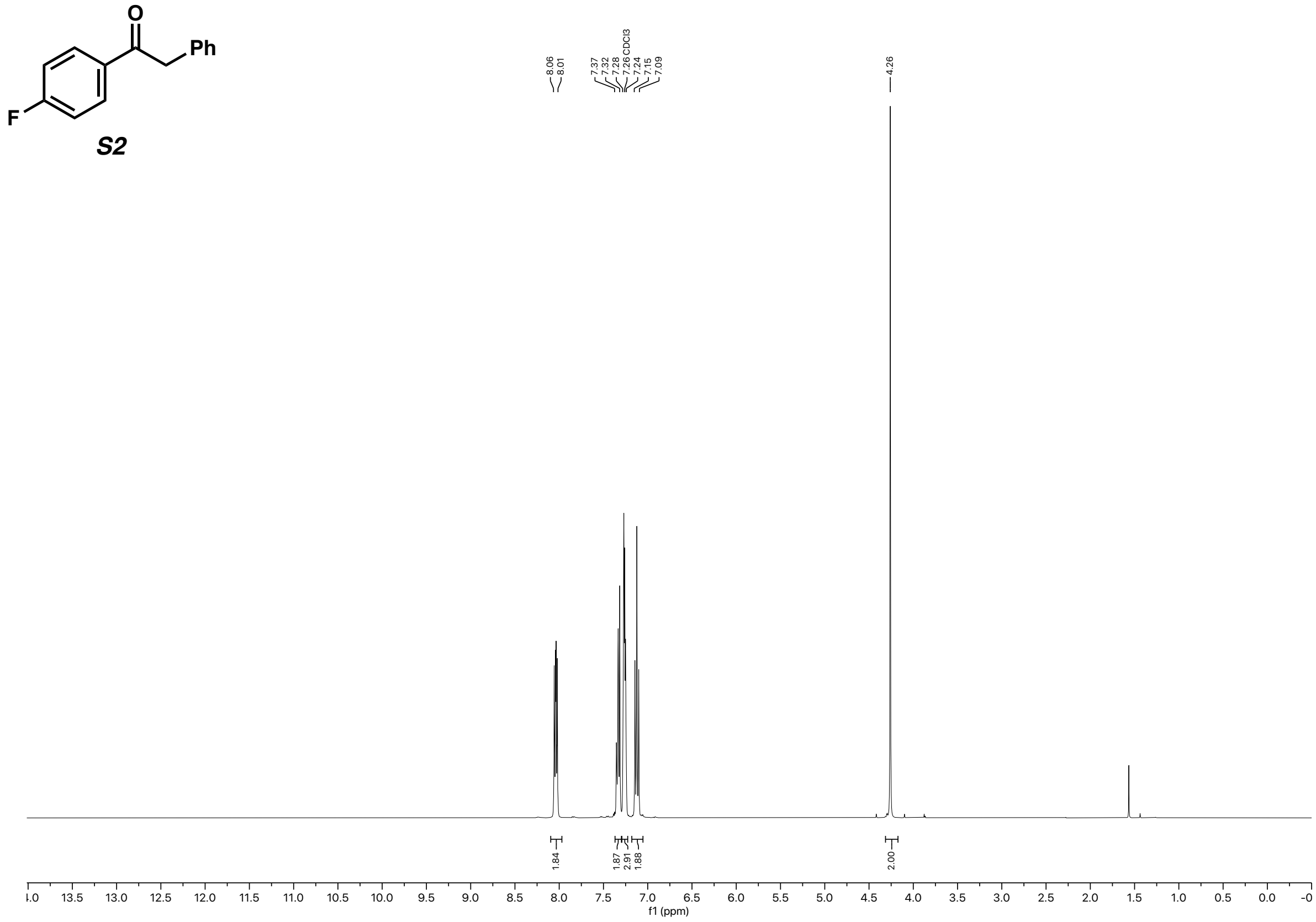

^{1}H NMR (400 MHz, $CDCl_3$) spectrum of compound **S2**.

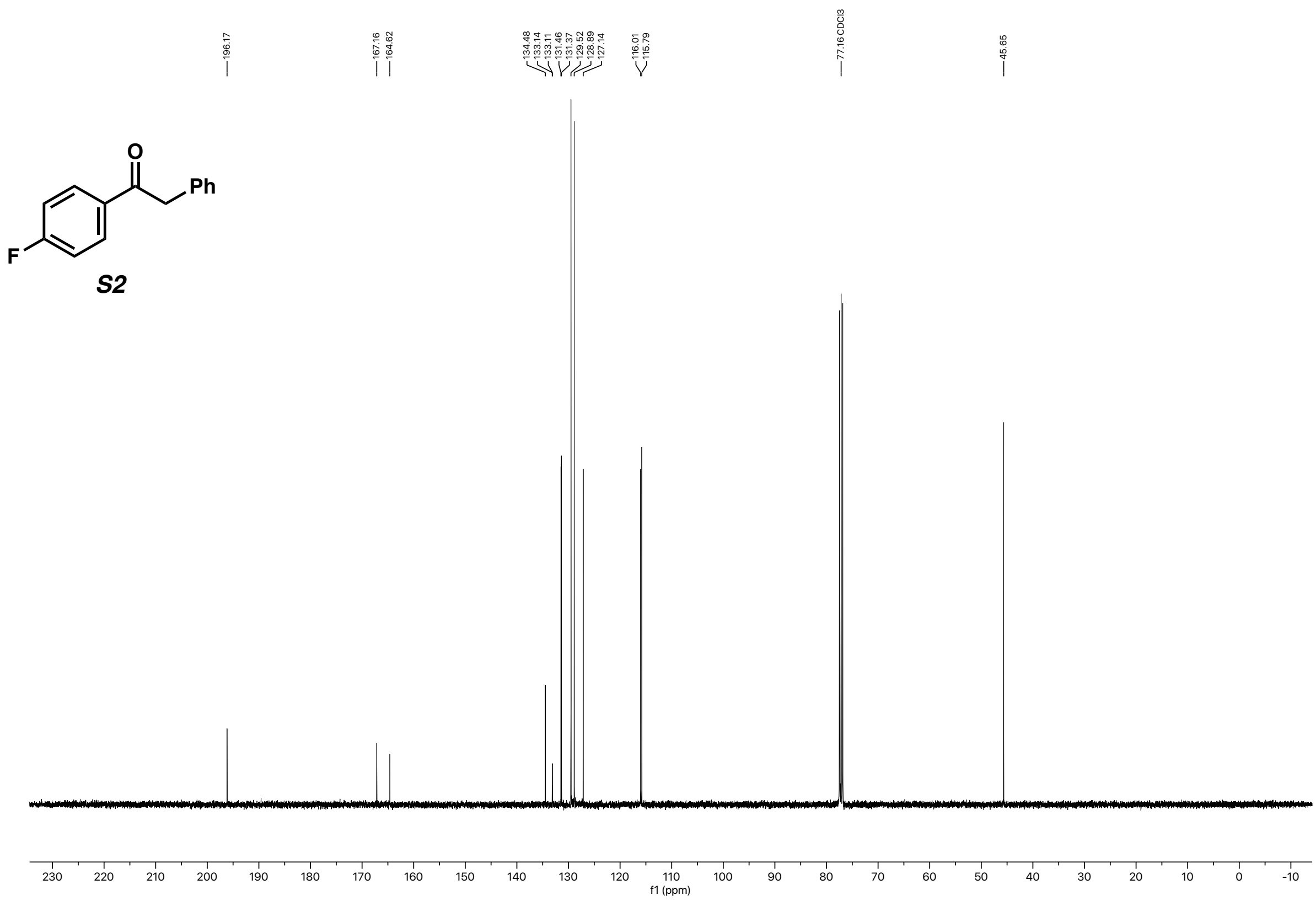

^{13}C NMR (101 MHz, $CDCl_3$) spectrum of compound **S2**.

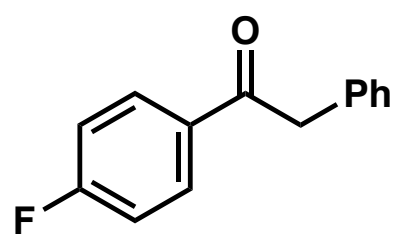

S2

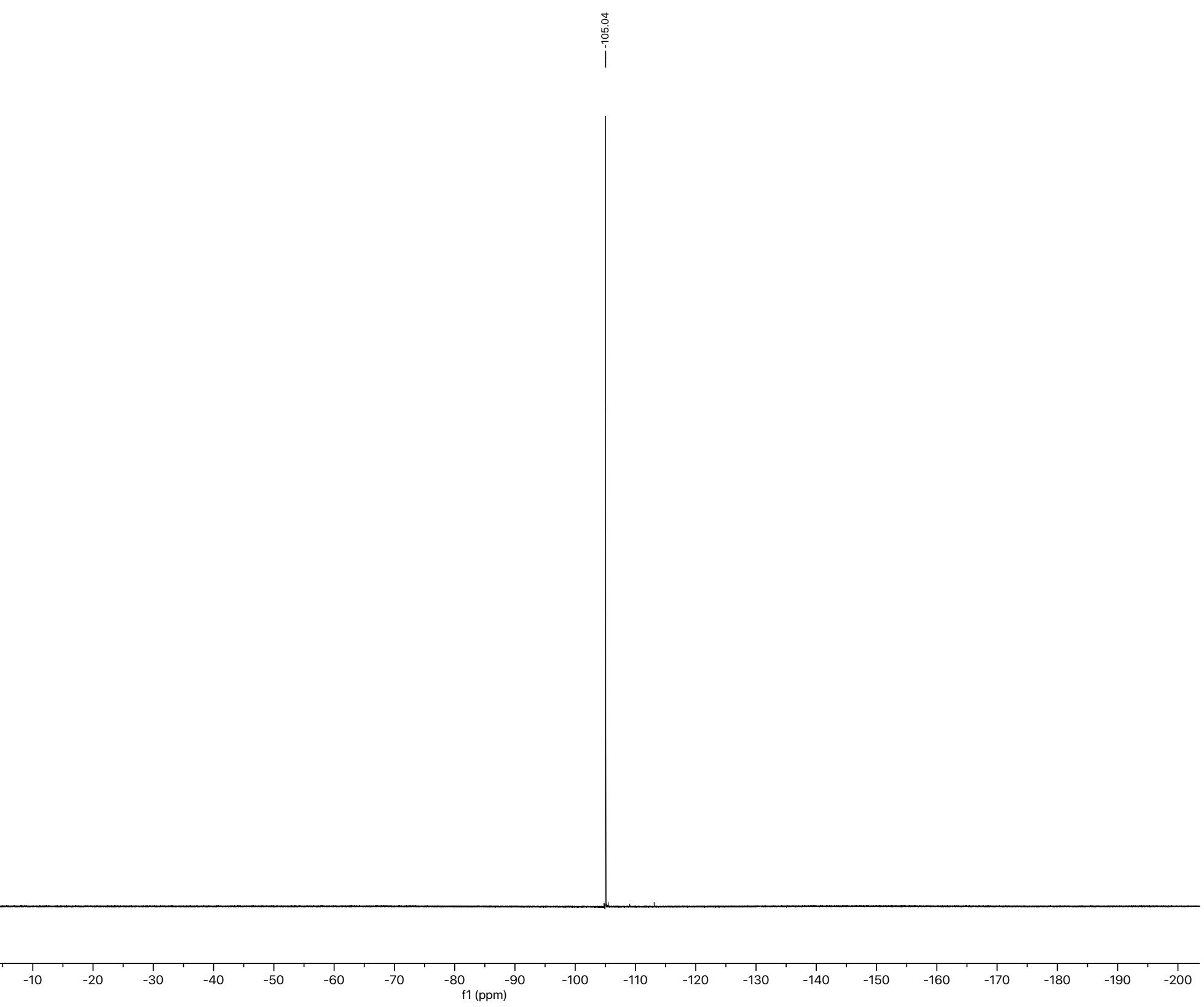

^{19}F NMR (376 MHz, $CDCl_3$) spectrum of compound **S2**.

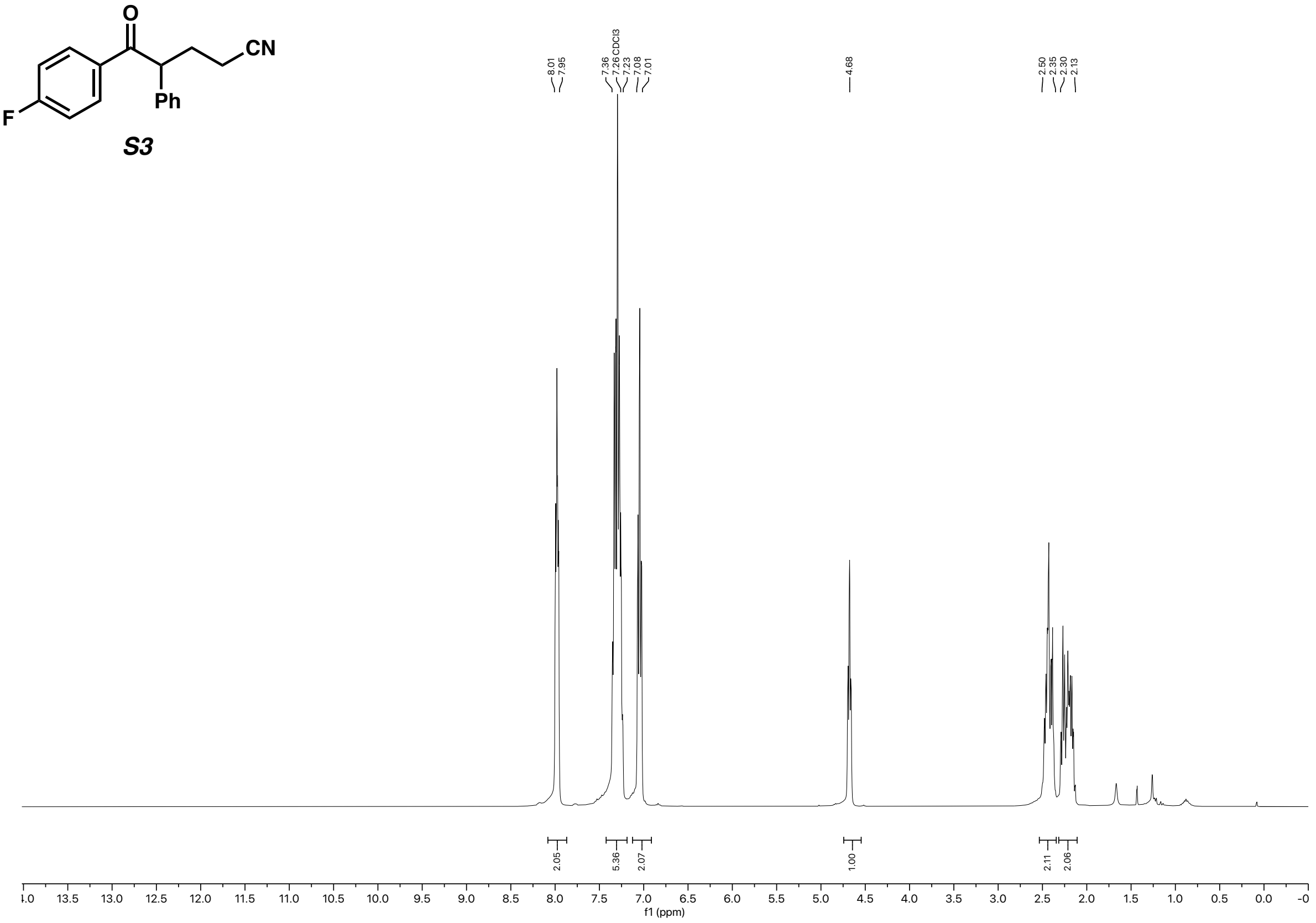

^{1}H NMR (400 MHz, $CDCl_3$) spectrum of compound **S3**.

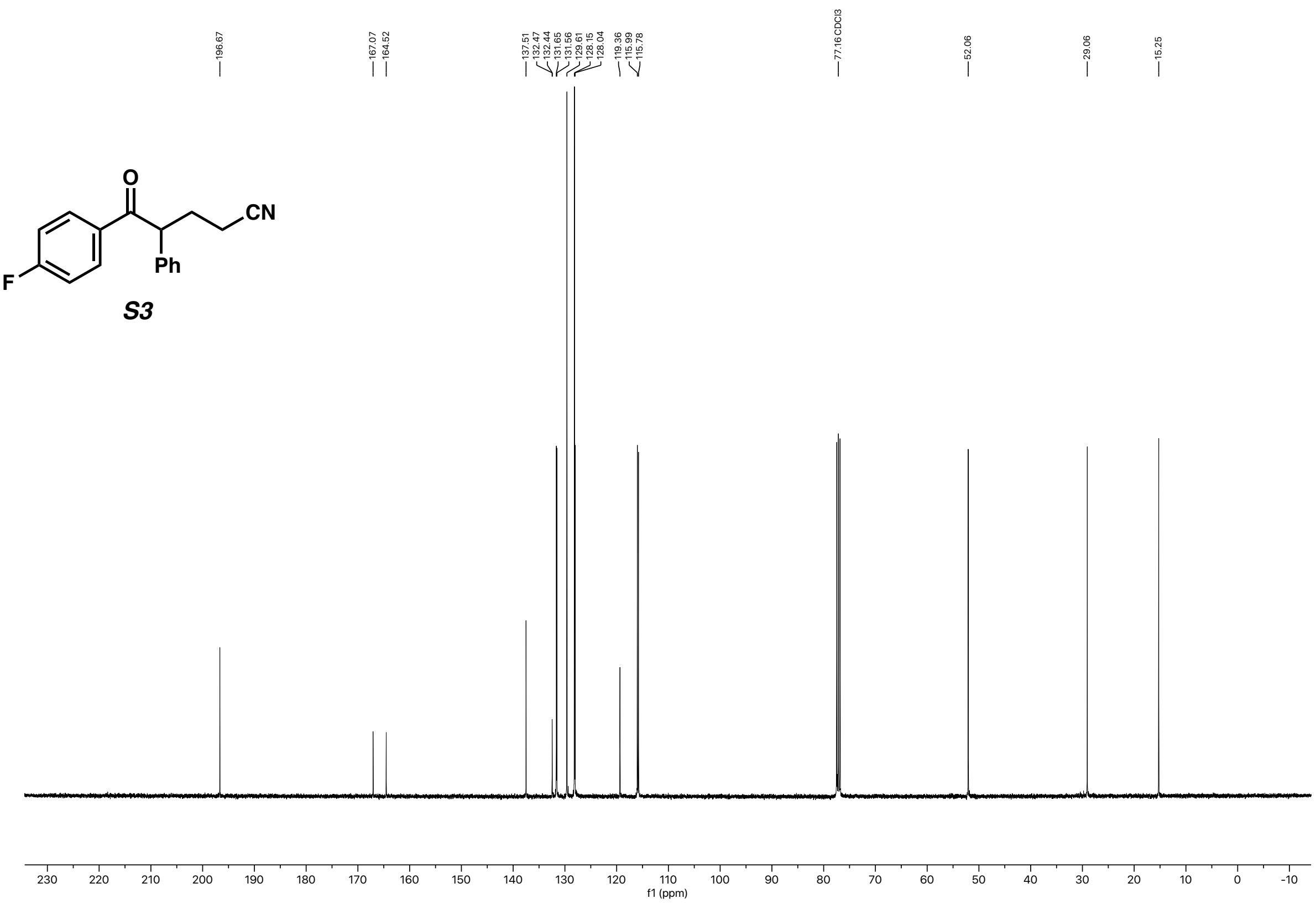

^{13}C NMR (101 MHz, $CDCl_3$) spectrum of compound **S3**.

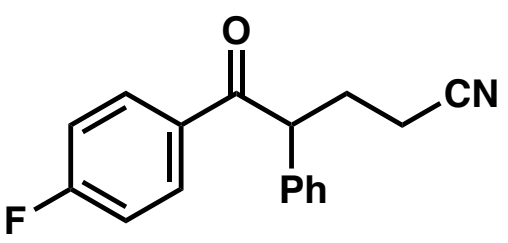

S3

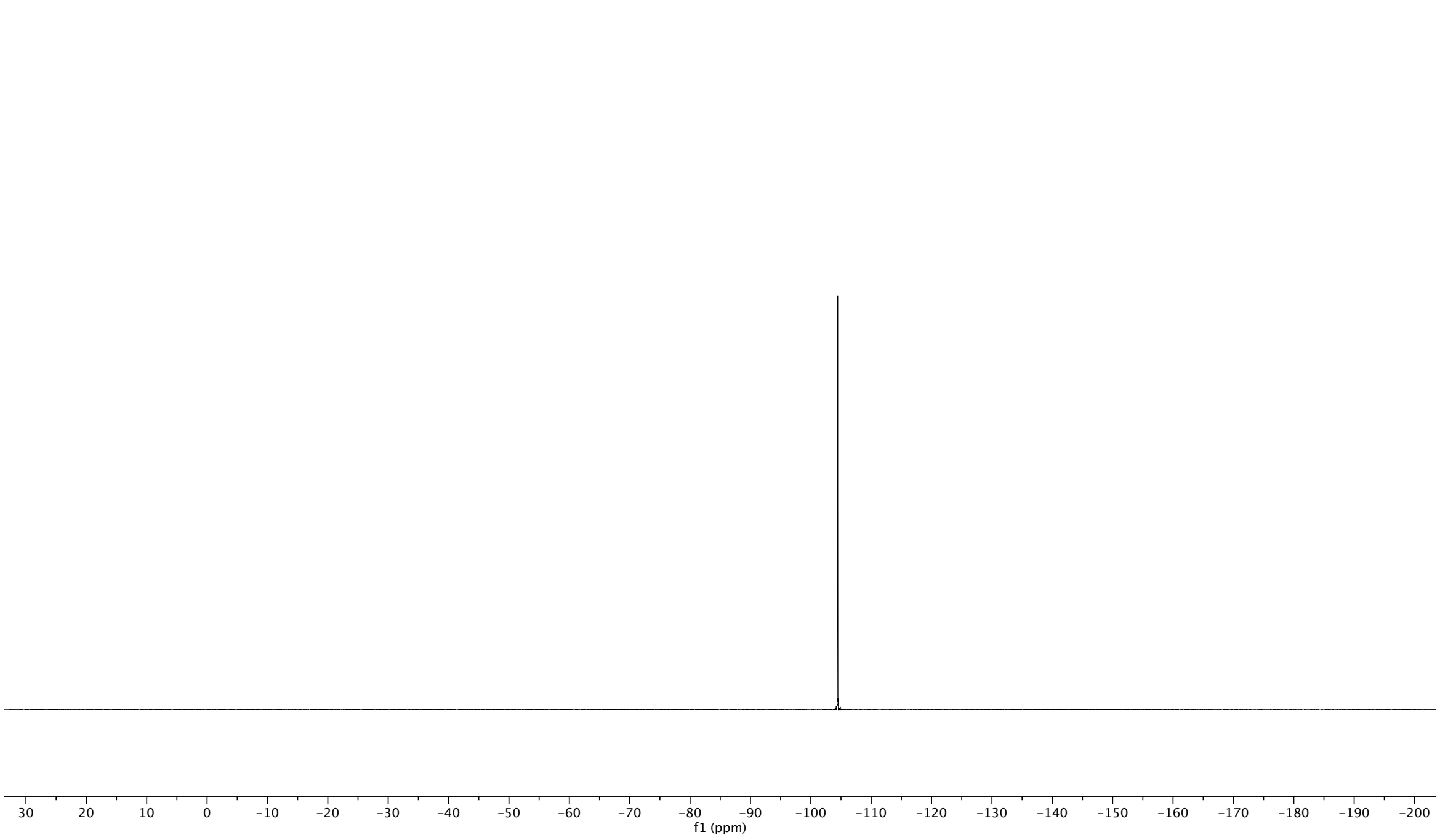

^{19}F NMR (376 MHz, $CDCl_3$) spectrum of compound **S3**.

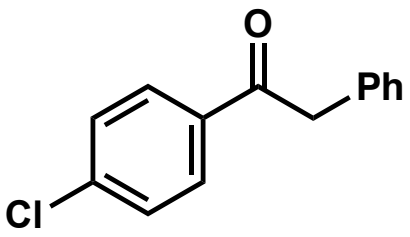

S4

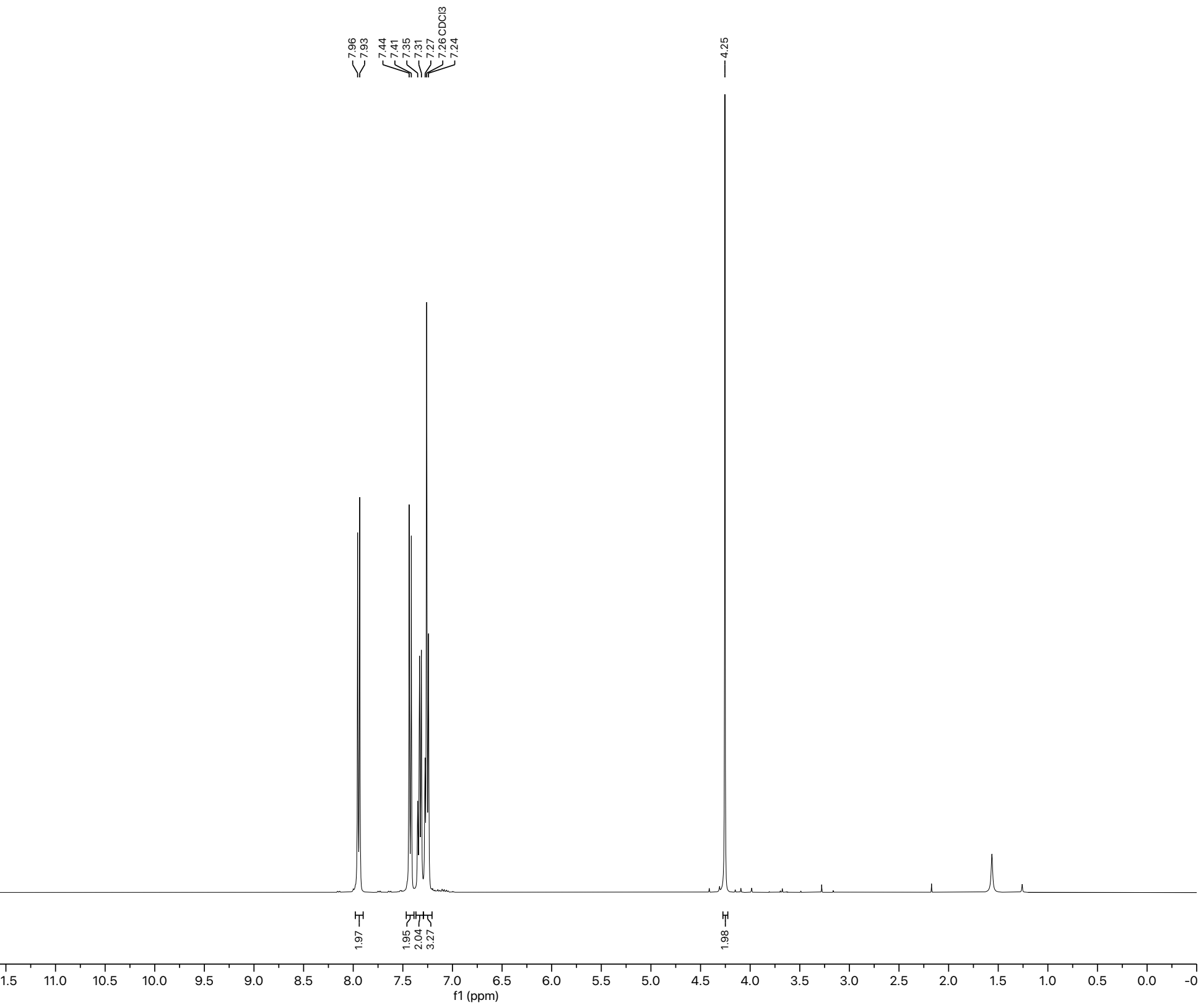

^{1}H NMR (400 MHz, $CDCl_3$) spectrum of compound **S4**.

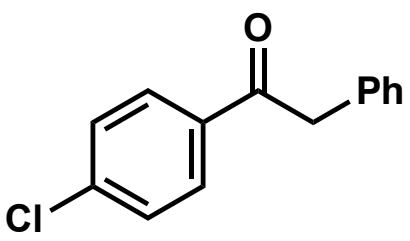

S4

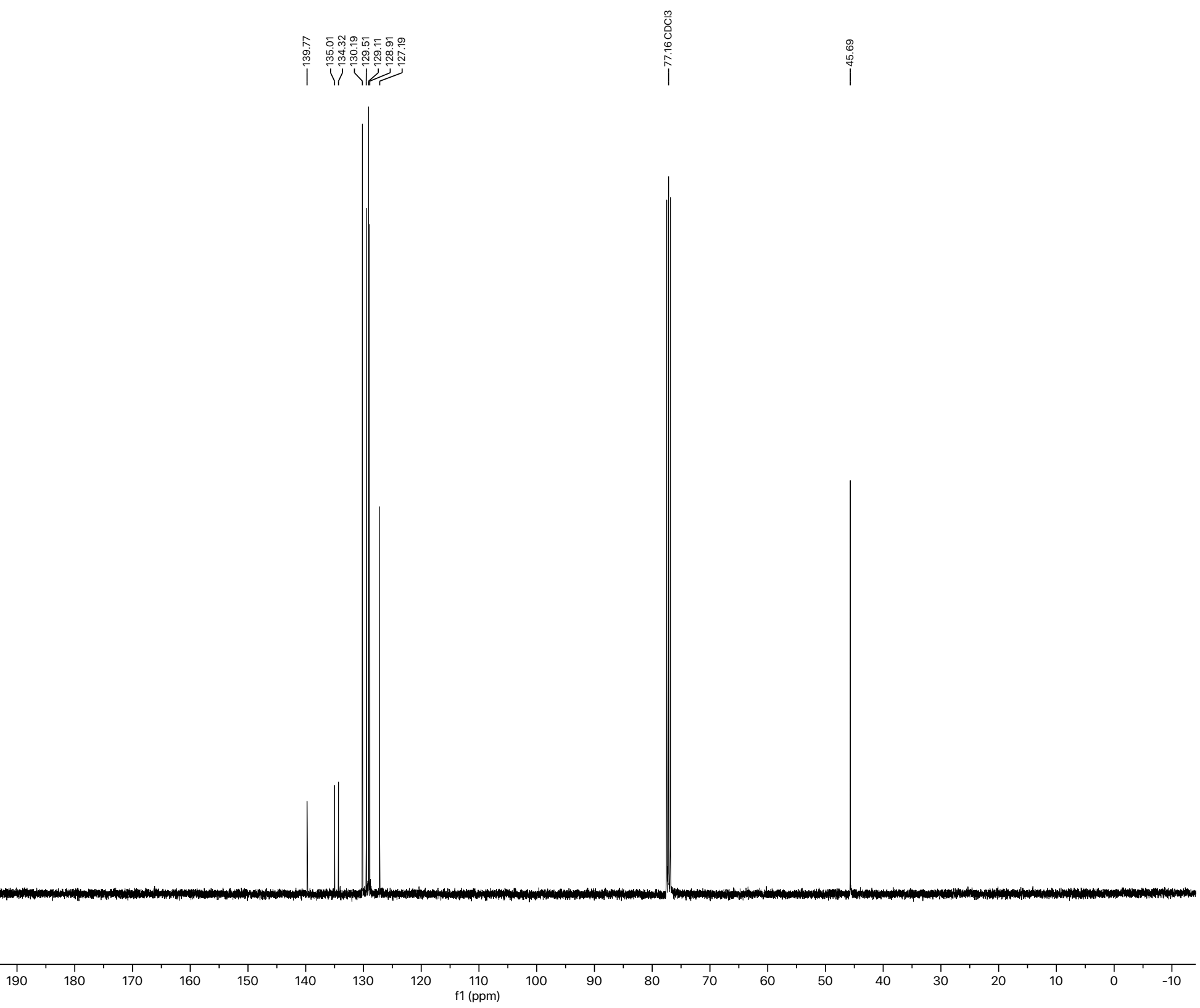

^{13}C NMR (101 MHz, $CDCl_3$) spectrum of compound **S4**.

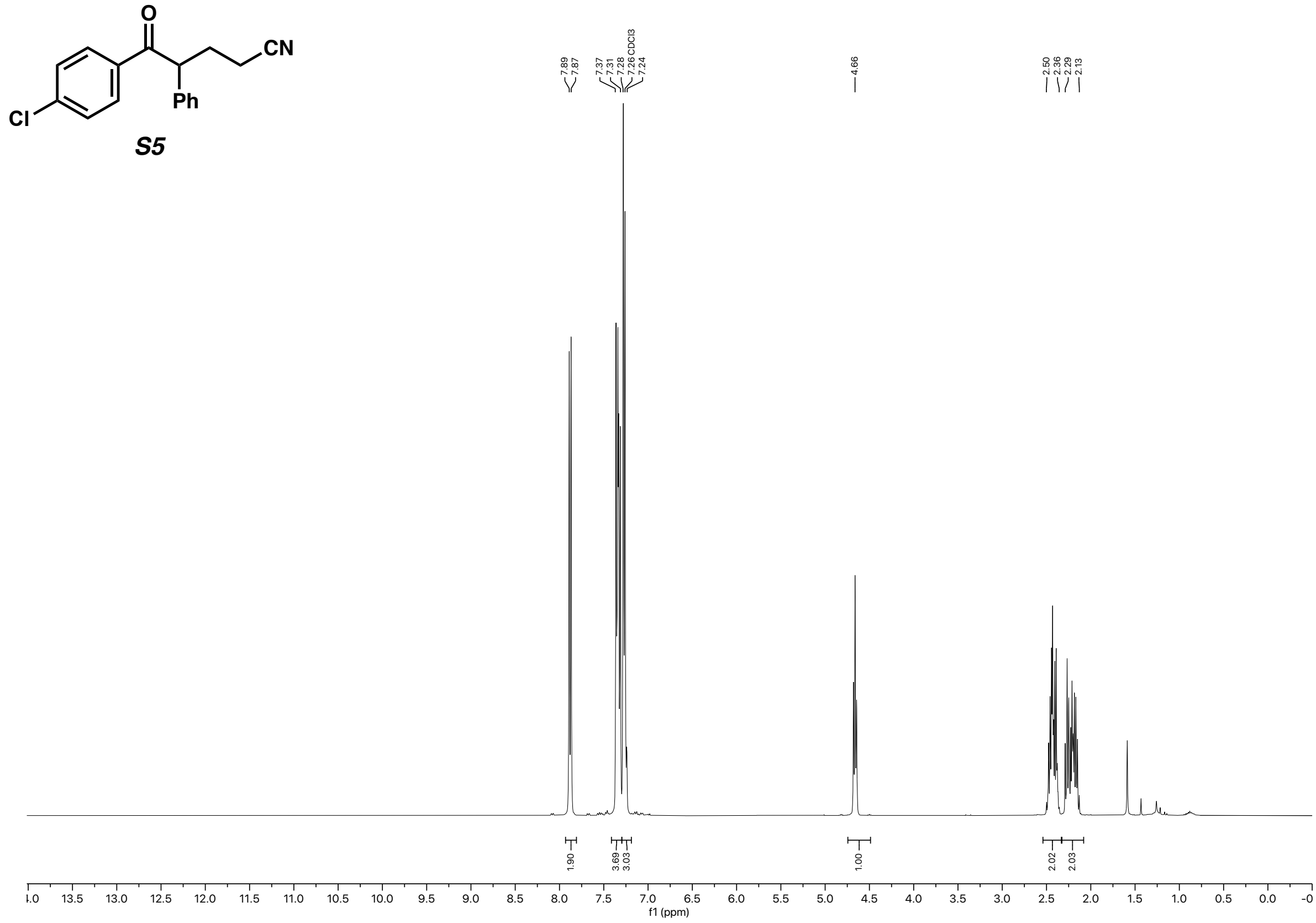

^{1}H NMR (400 MHz, $CDCl_3$) spectrum of compound **S5**.

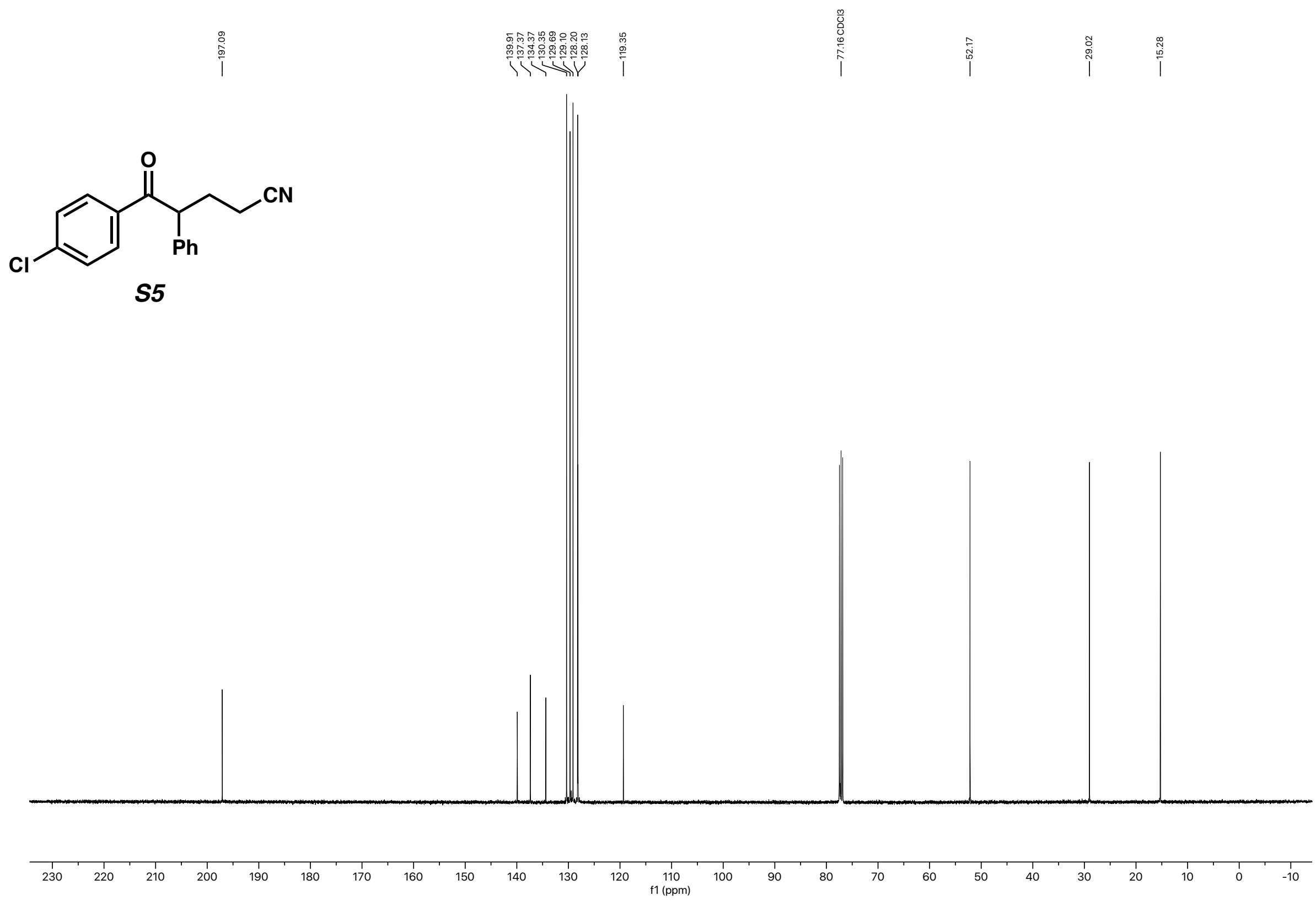

^{13}C NMR (101 MHz, $CDCl_3$) spectrum of compound **S5**.

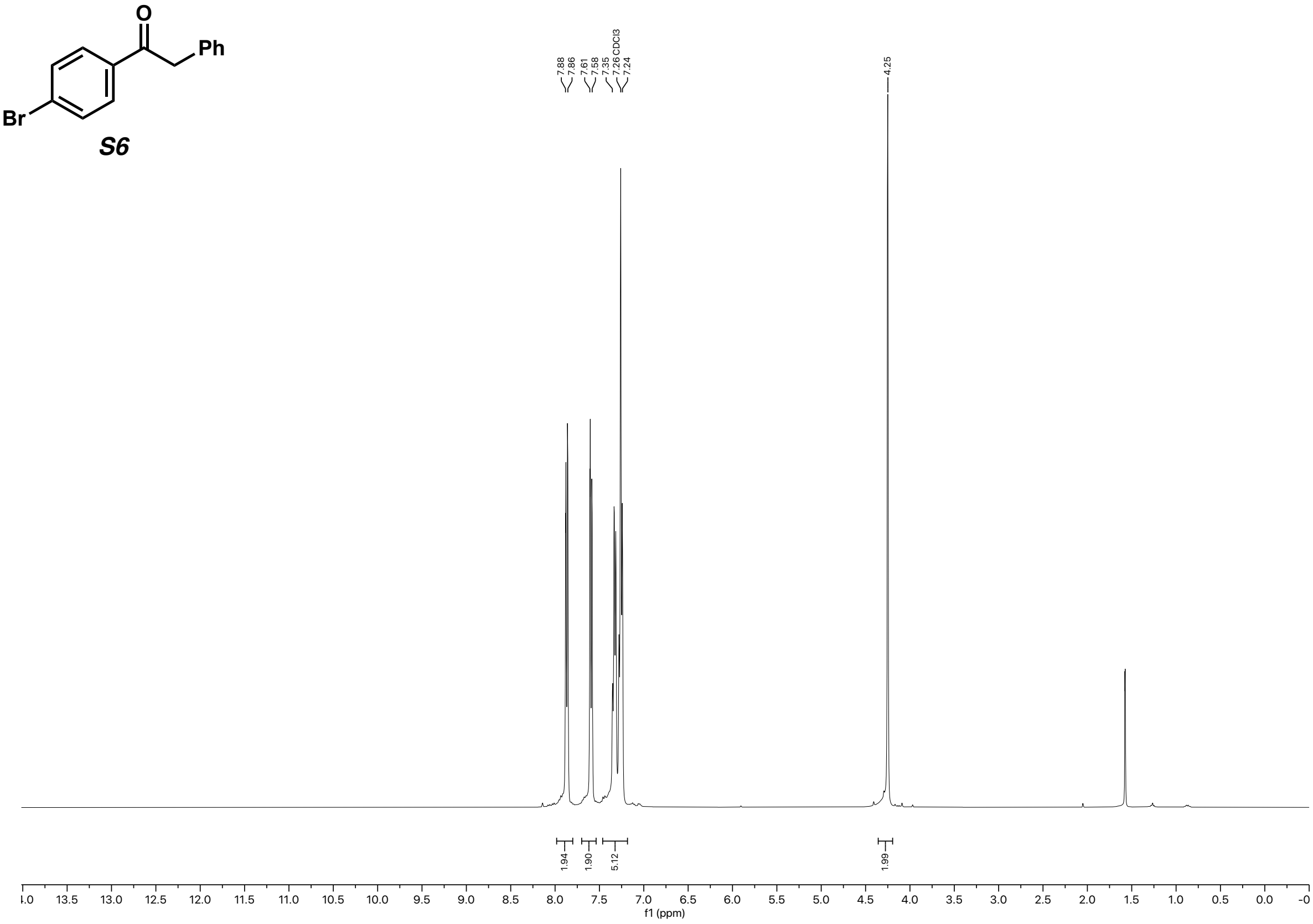

^{1}H NMR (400 MHz, $CDCl_3$) spectrum of compound **S6**.

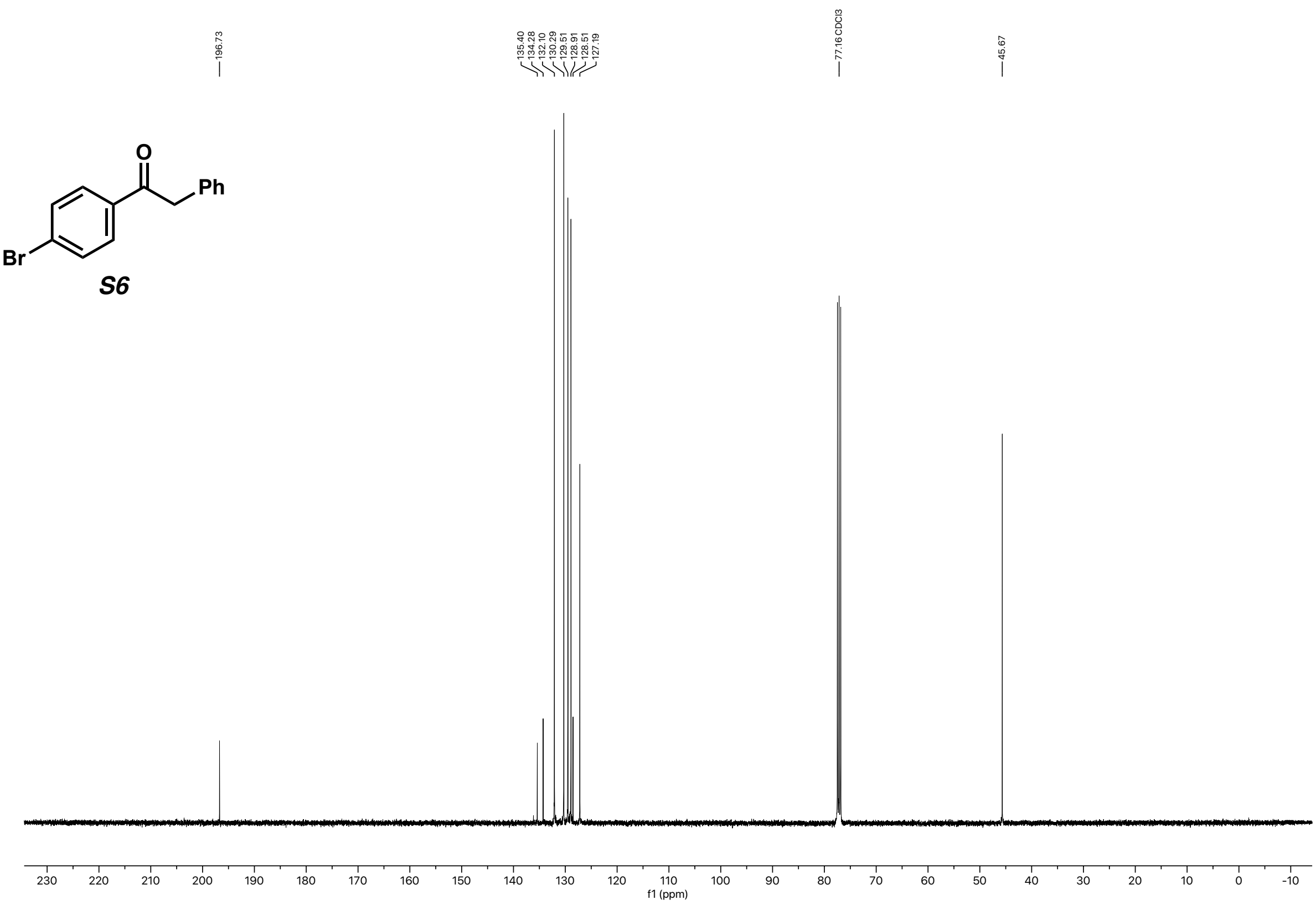

^{13}C NMR (101 MHz, $CDCl_3$) spectrum of compound **S6**.

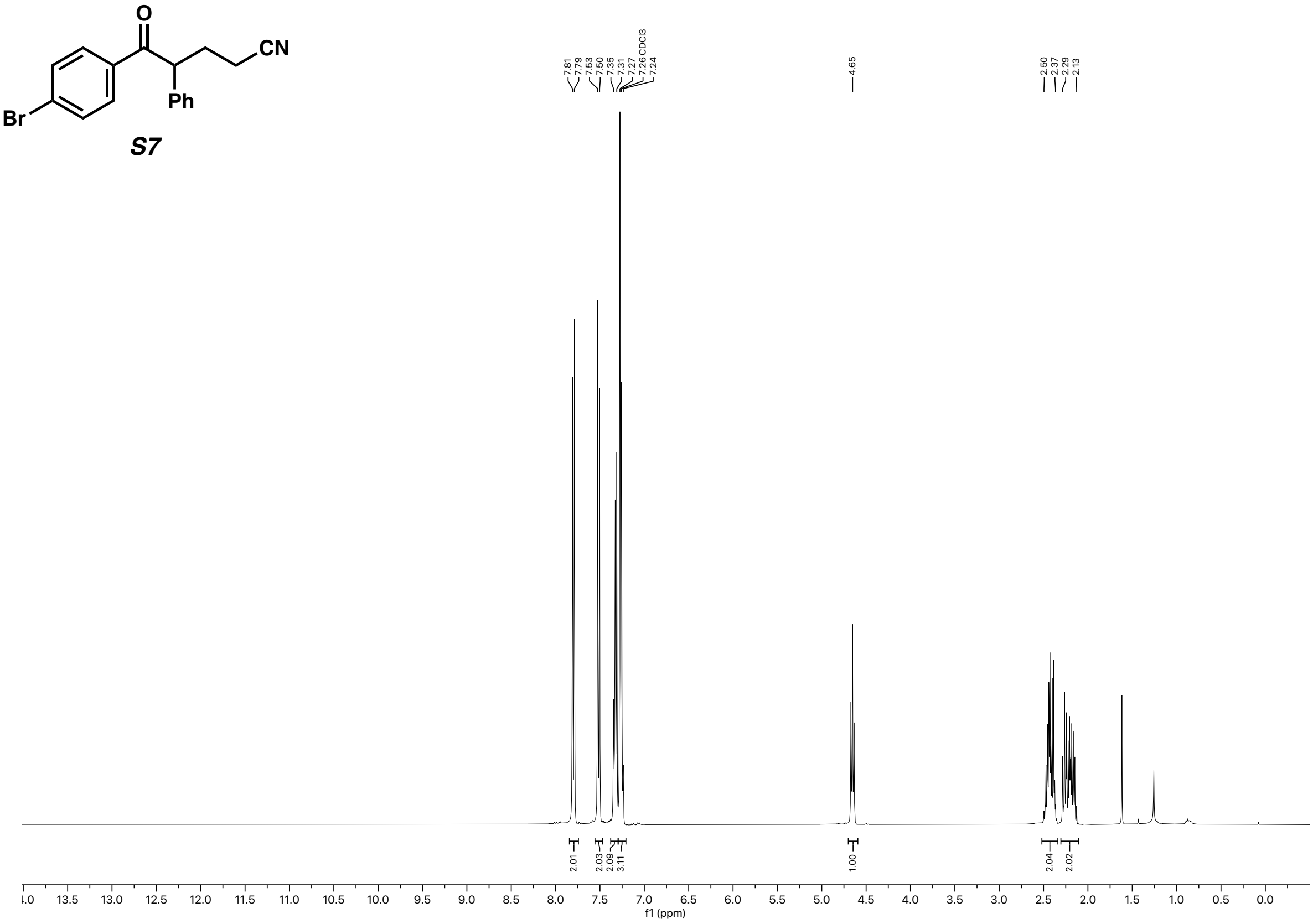

^{1}H NMR (400 MHz, $CDCl_3$) spectrum of compound **S7**.

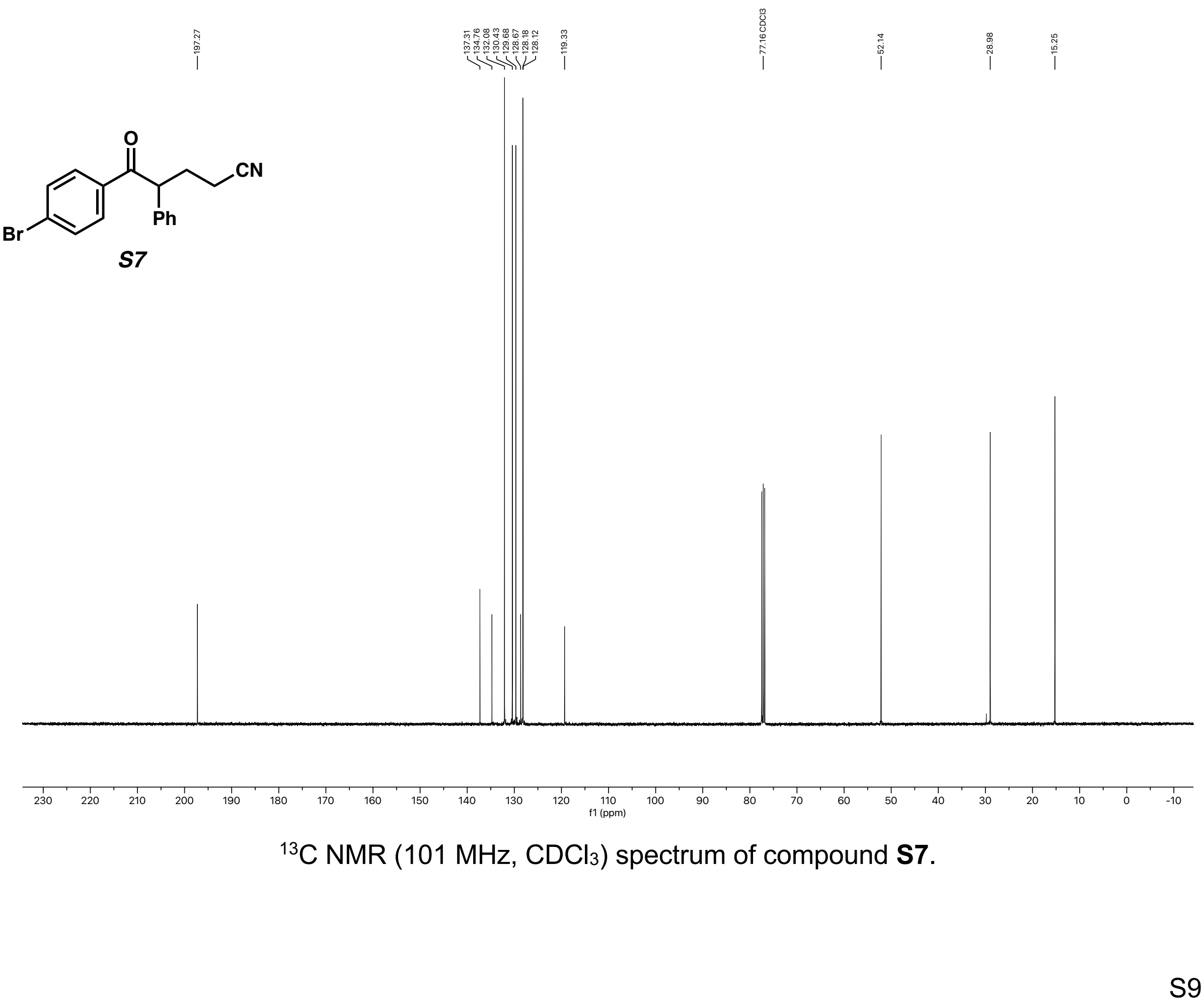

^{13}C NMR (101 MHz, $CDCl_3$) spectrum of compound **S7**.

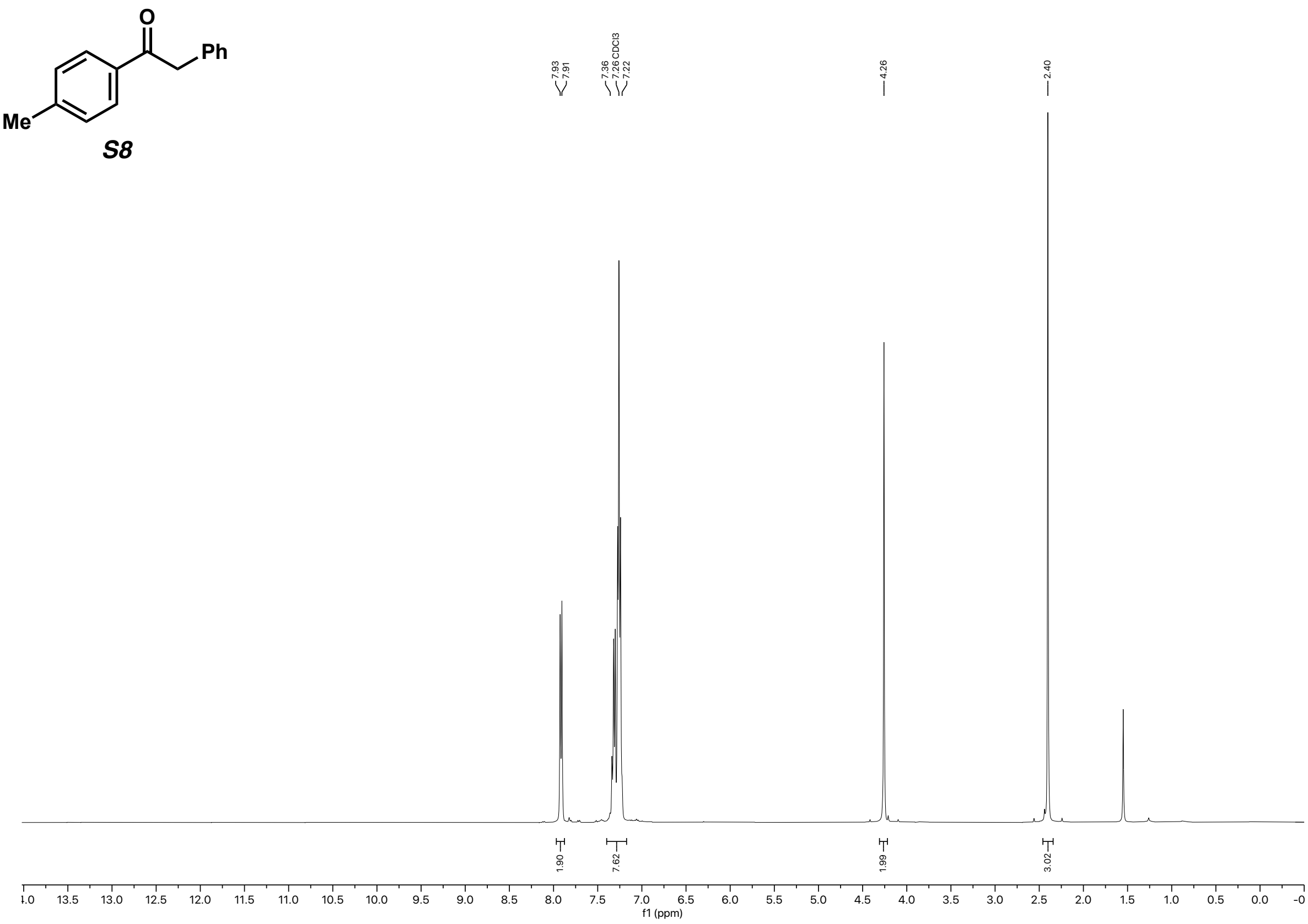

^{1}H NMR (400 MHz, $CDCl_3$) spectrum of compound **S8**.

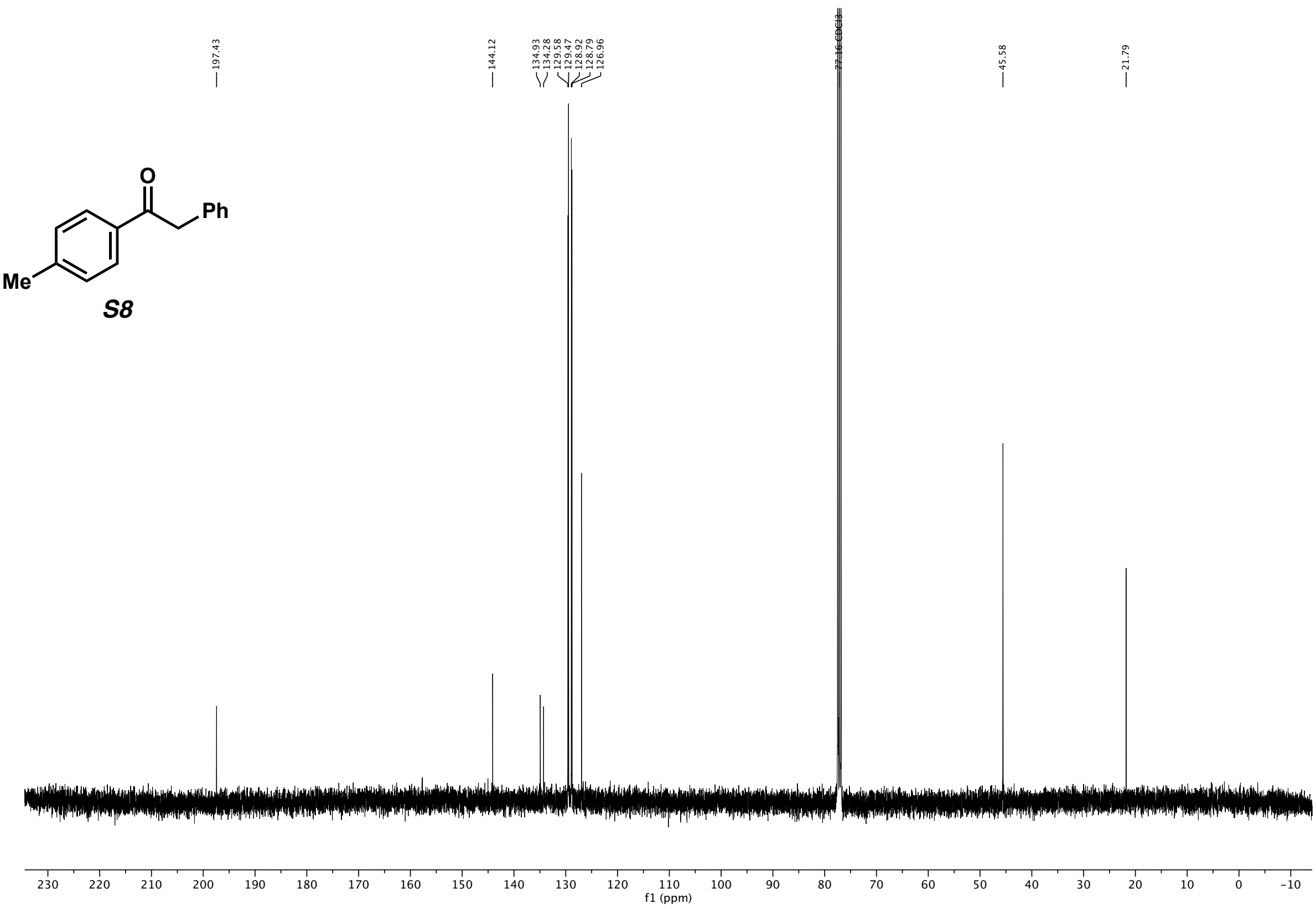

^{13}C NMR (101 MHz, $CDCl_3$) spectrum of compound **S8**.

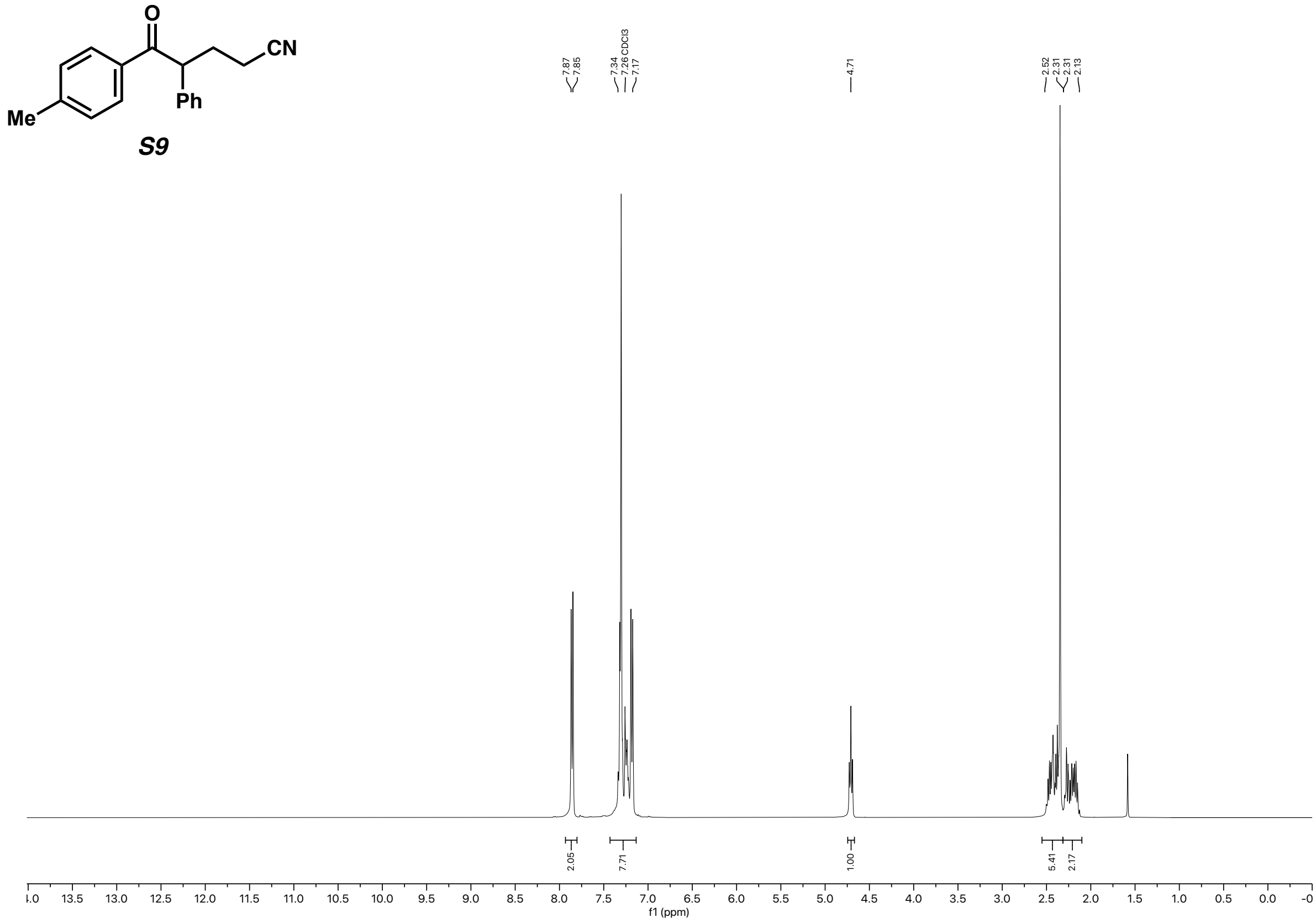

^{1}H NMR (400 MHz, $CDCl_3$) spectrum of compound **S9**.

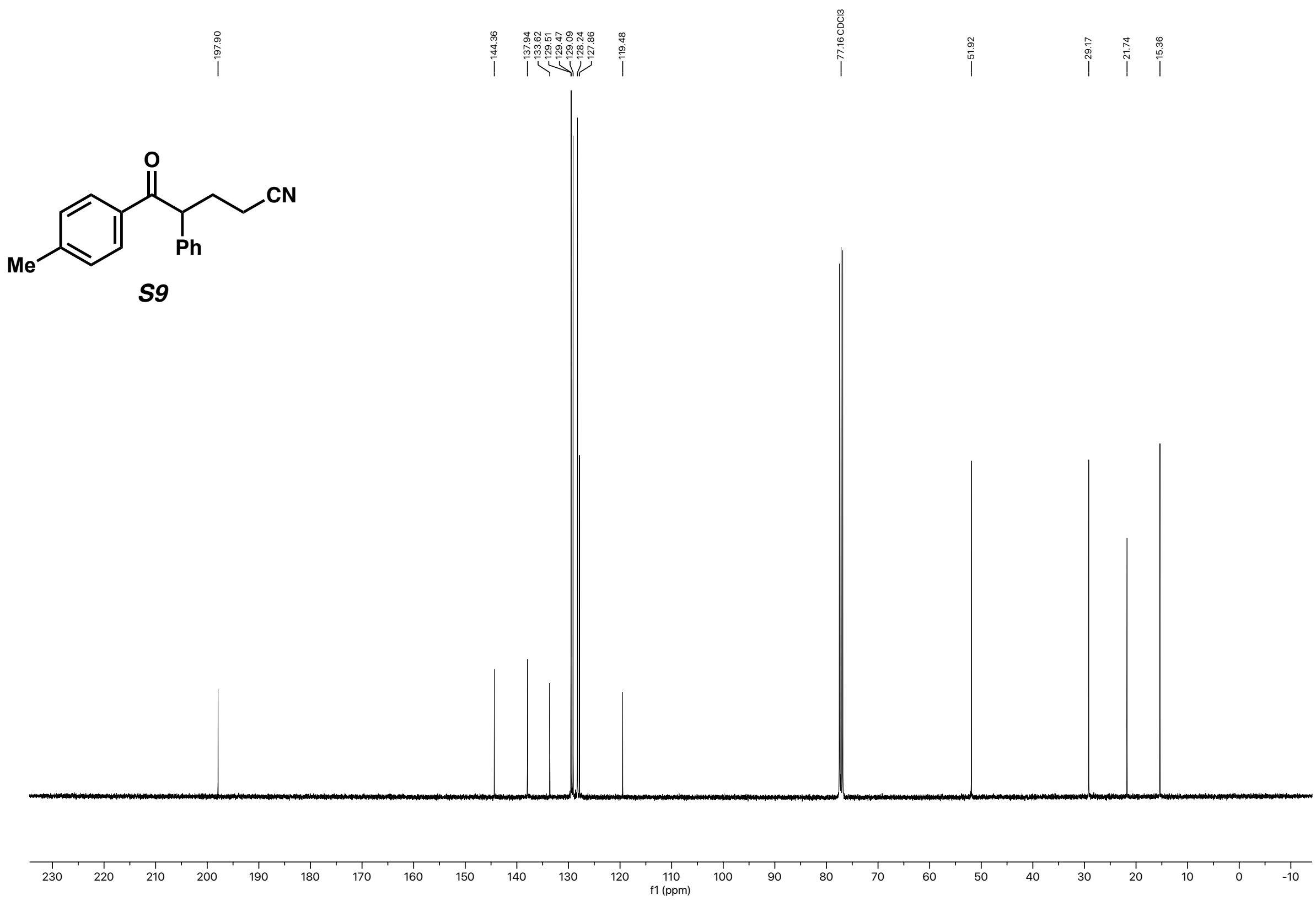

^{13}C NMR (101 MHz, $CDCl_3$) spectrum of compound **S9**.

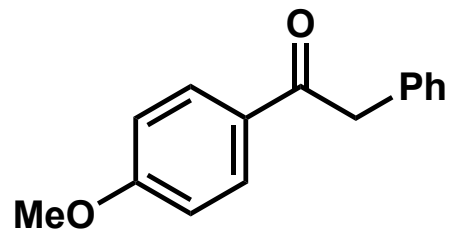

S10

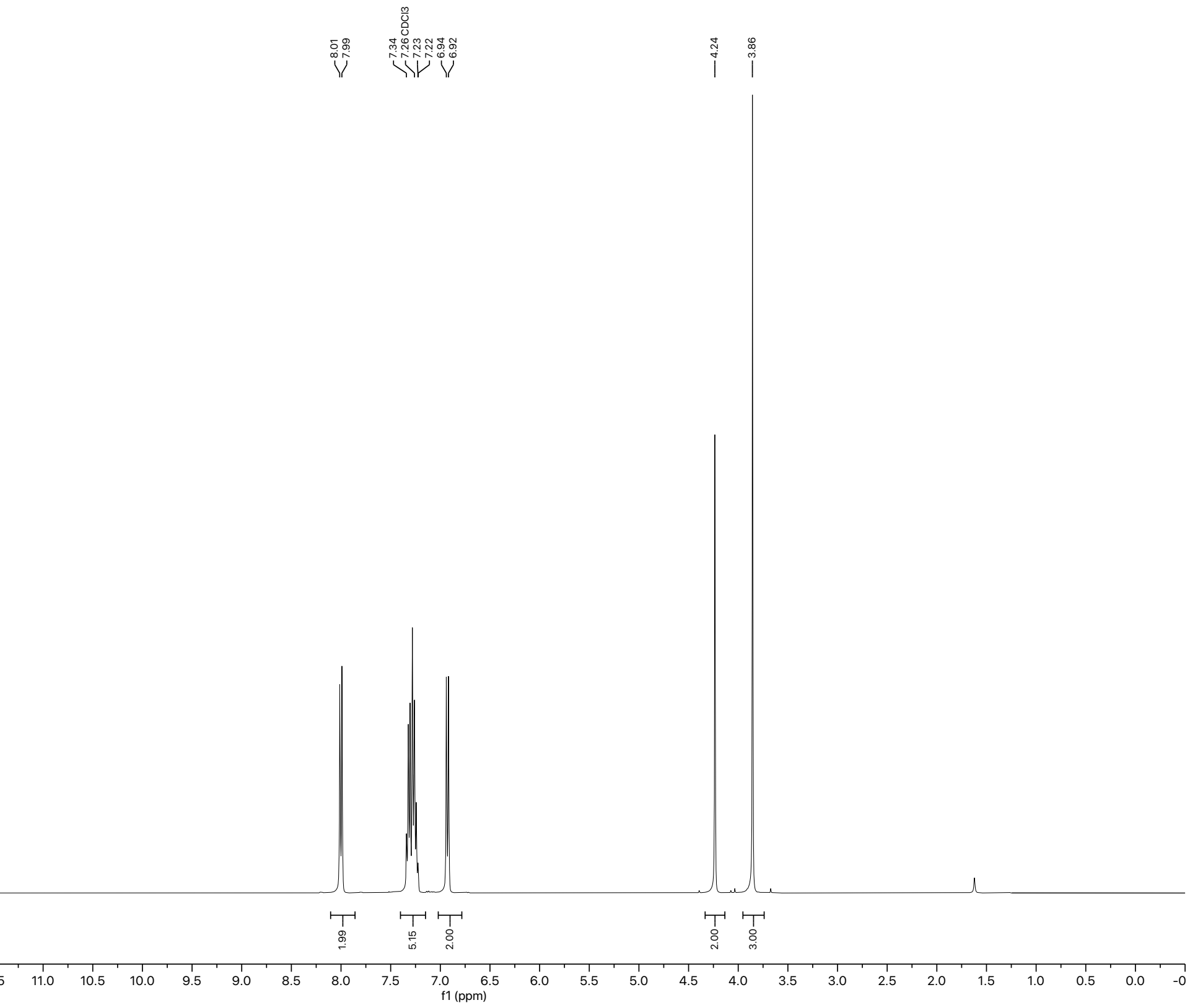

^{1}H NMR (400 MHz, $CDCl_3$) spectrum of compound **S10**.

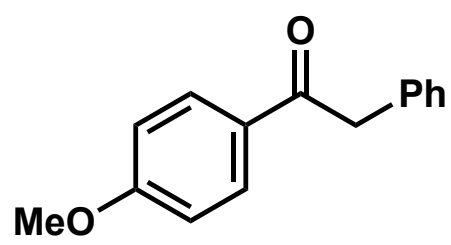

S10

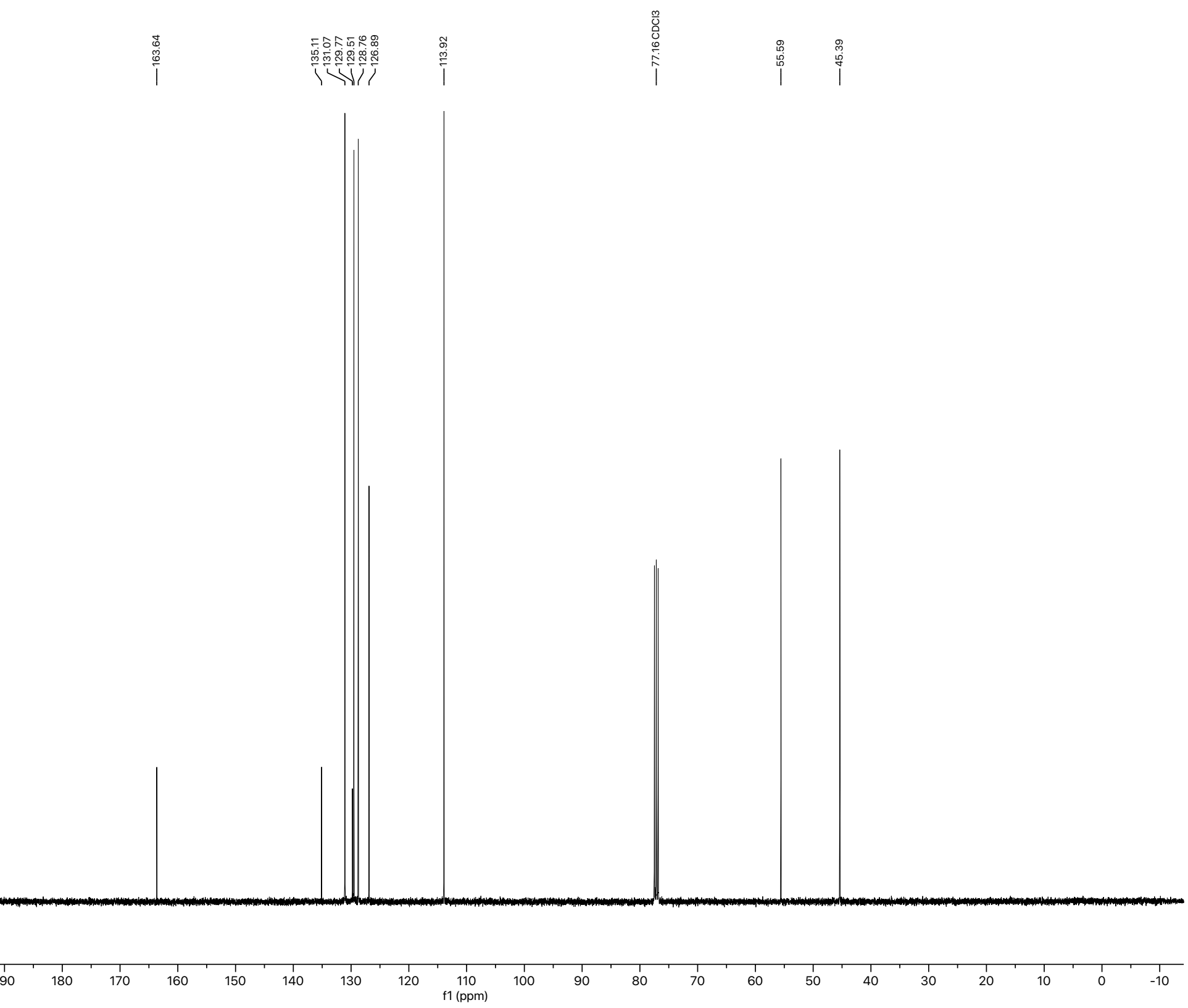

^{13}C NMR (101 MHz, $CDCl_3$) spectrum of compound **S10**.

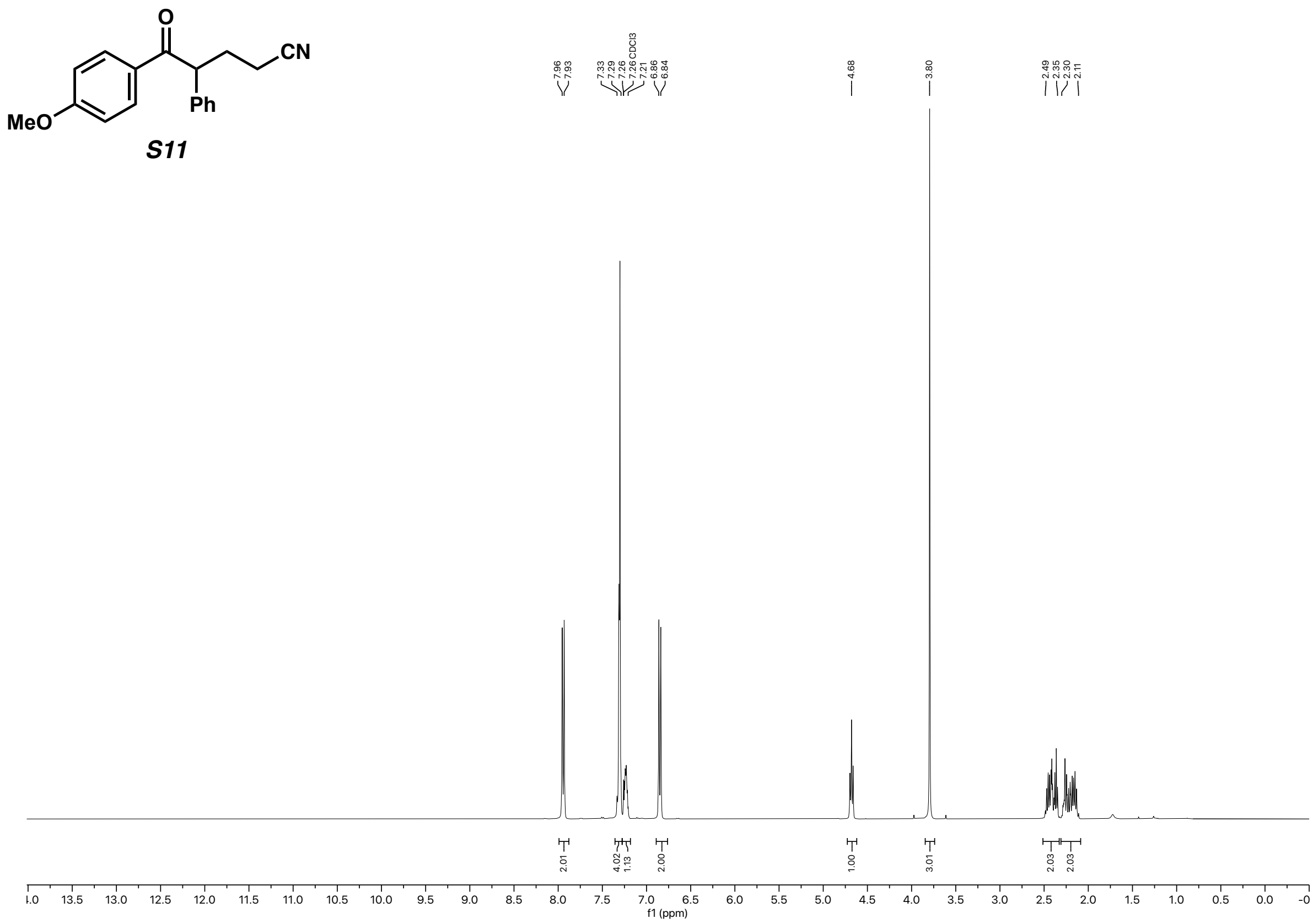

^{1}H NMR (400 MHz, $CDCl_3$) spectrum of compound **S11**.

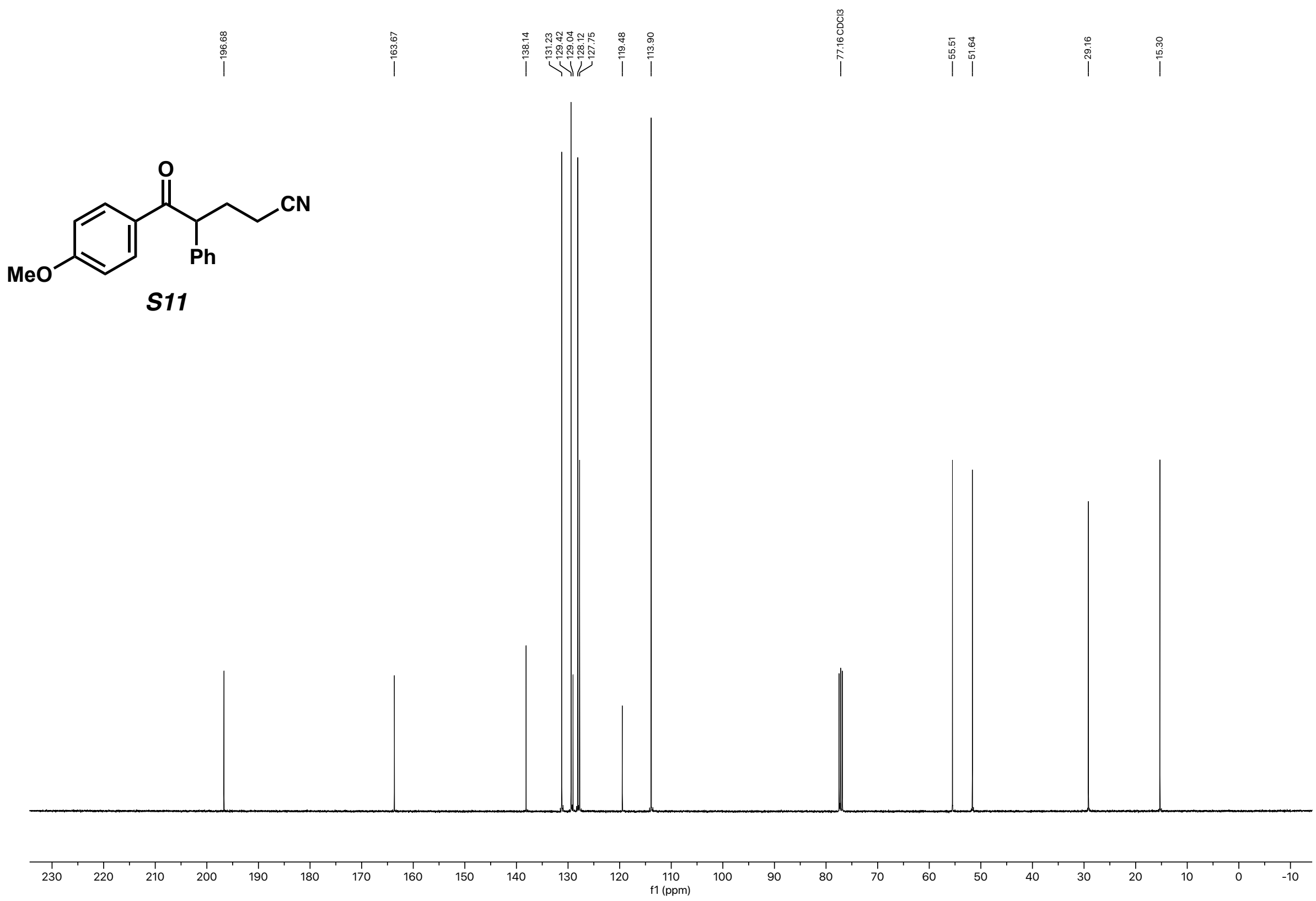

^{13}C NMR (101 MHz, $CDCl_3$) spectrum of compound **S11**.

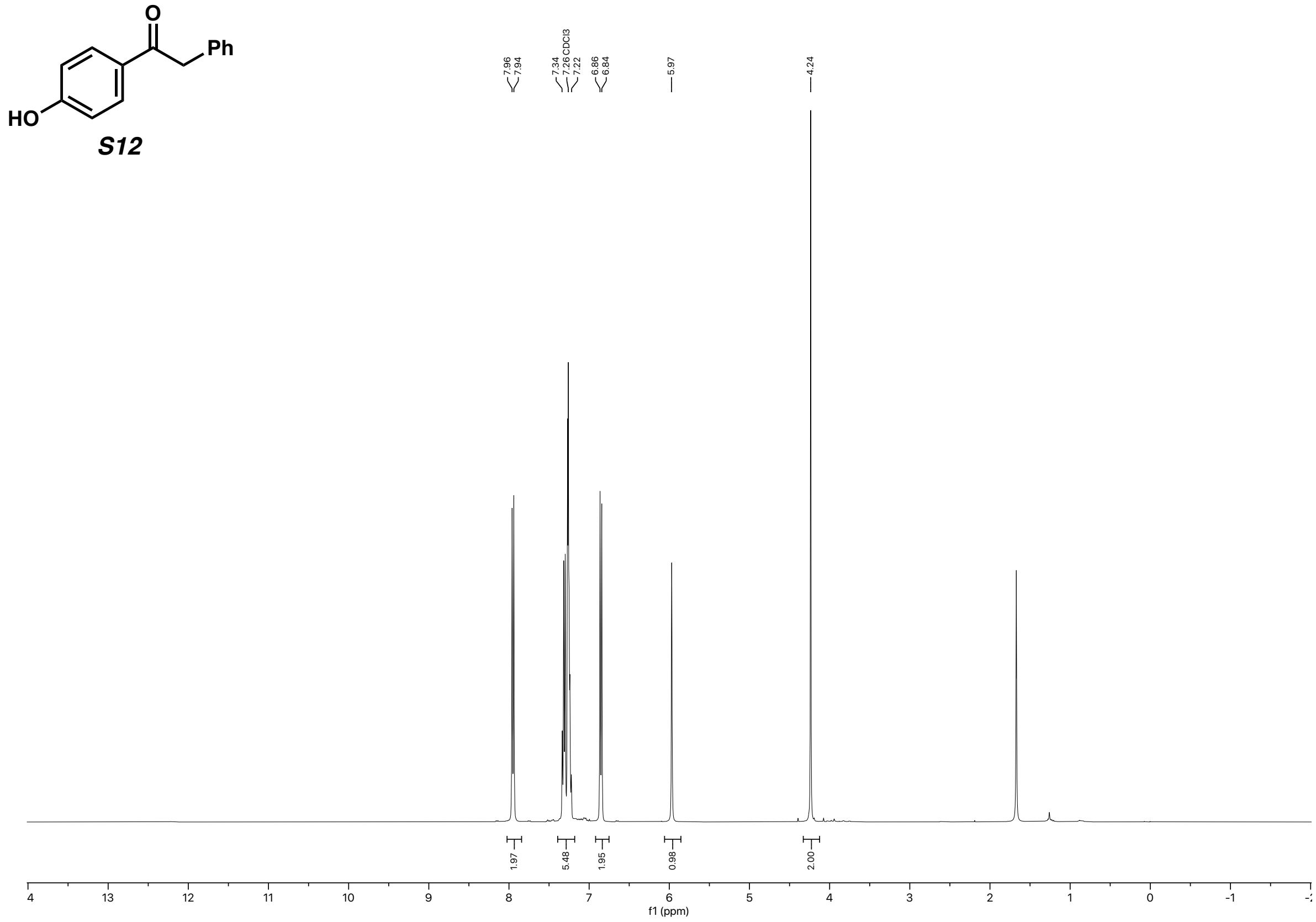

^{1}H NMR (400 MHz, $CDCl_3$) spectrum of compound **S12**.

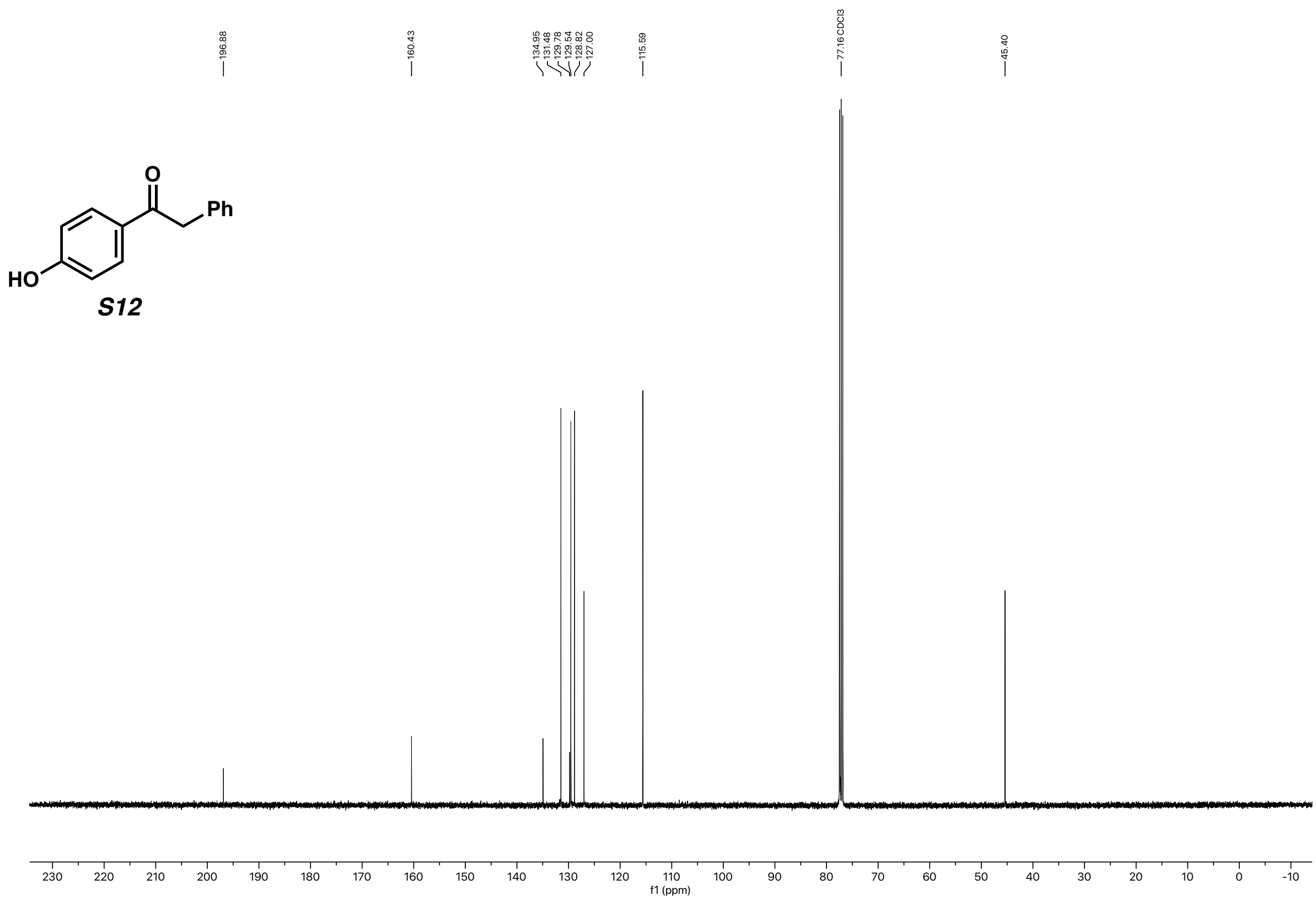

^{13}C NMR (101 MHz, $CDCl_3$) spectrum of compound **S12**.

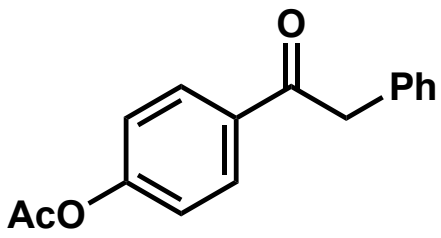

S13

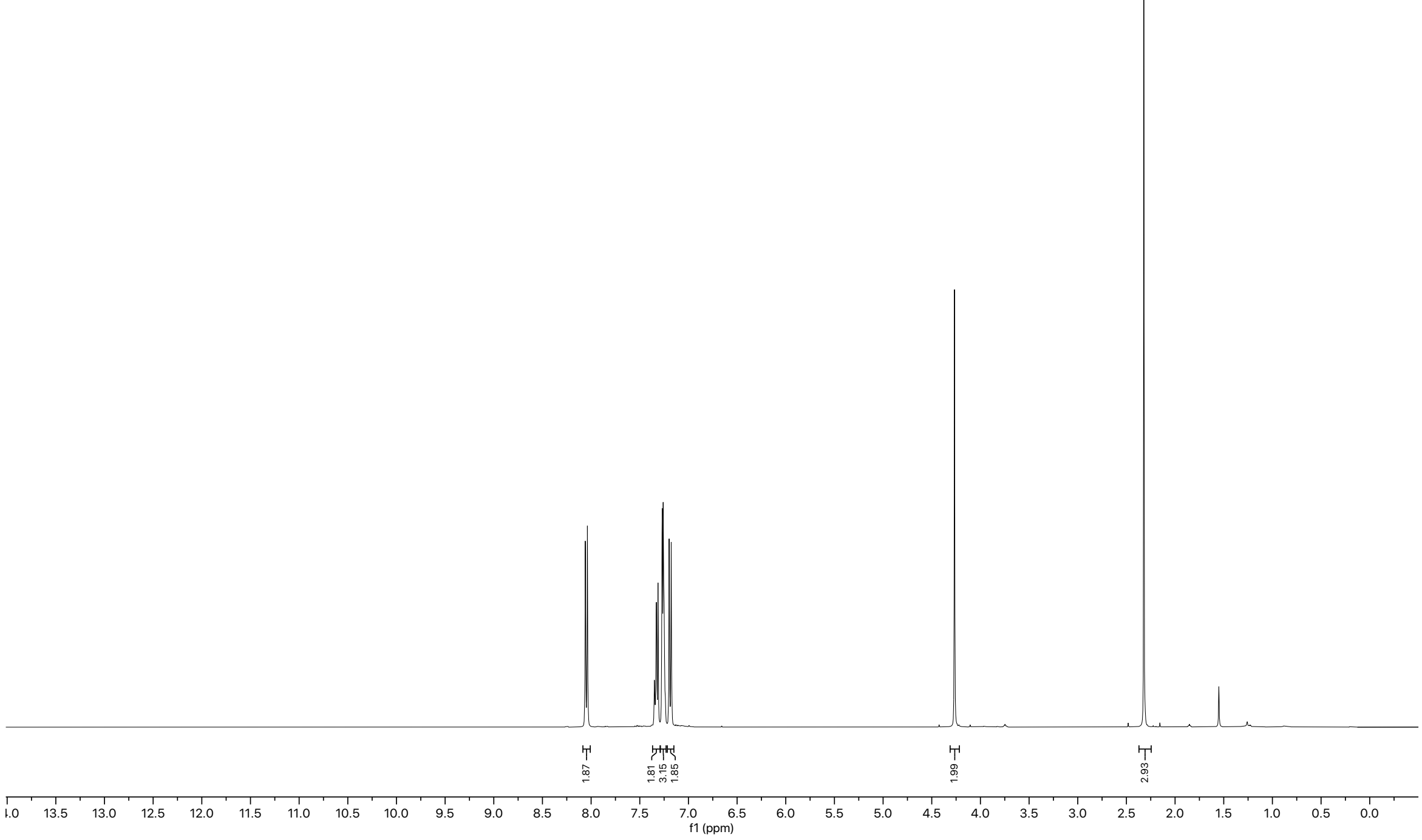

^{1}H NMR (400 MHz, $CDCl_3$) spectrum of compound **S13**.

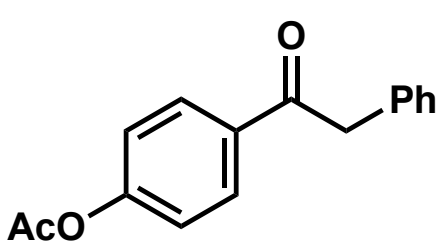

S13

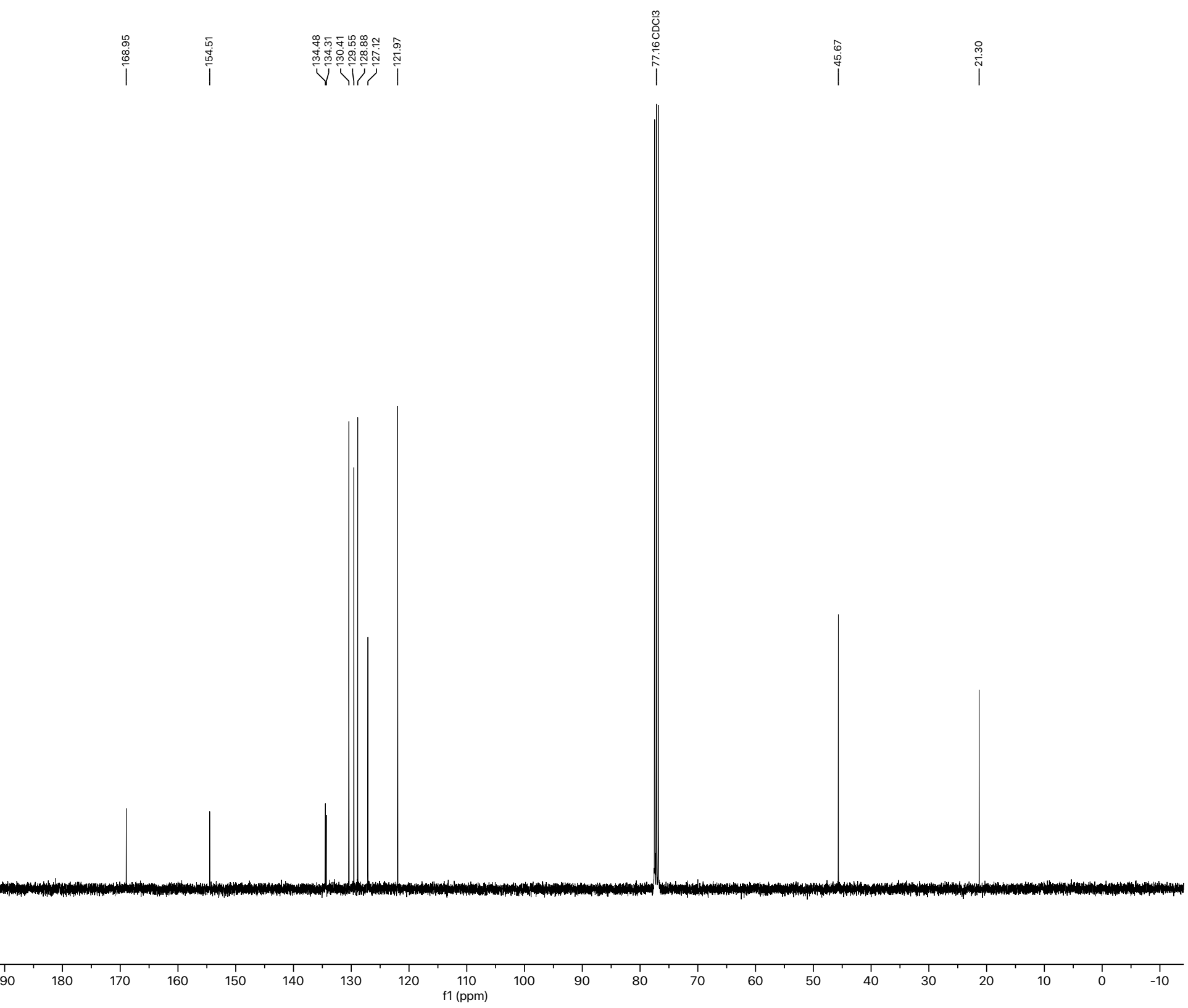

^{13}C NMR (101 MHz, $CDCl_3$) spectrum of compound **S13**.

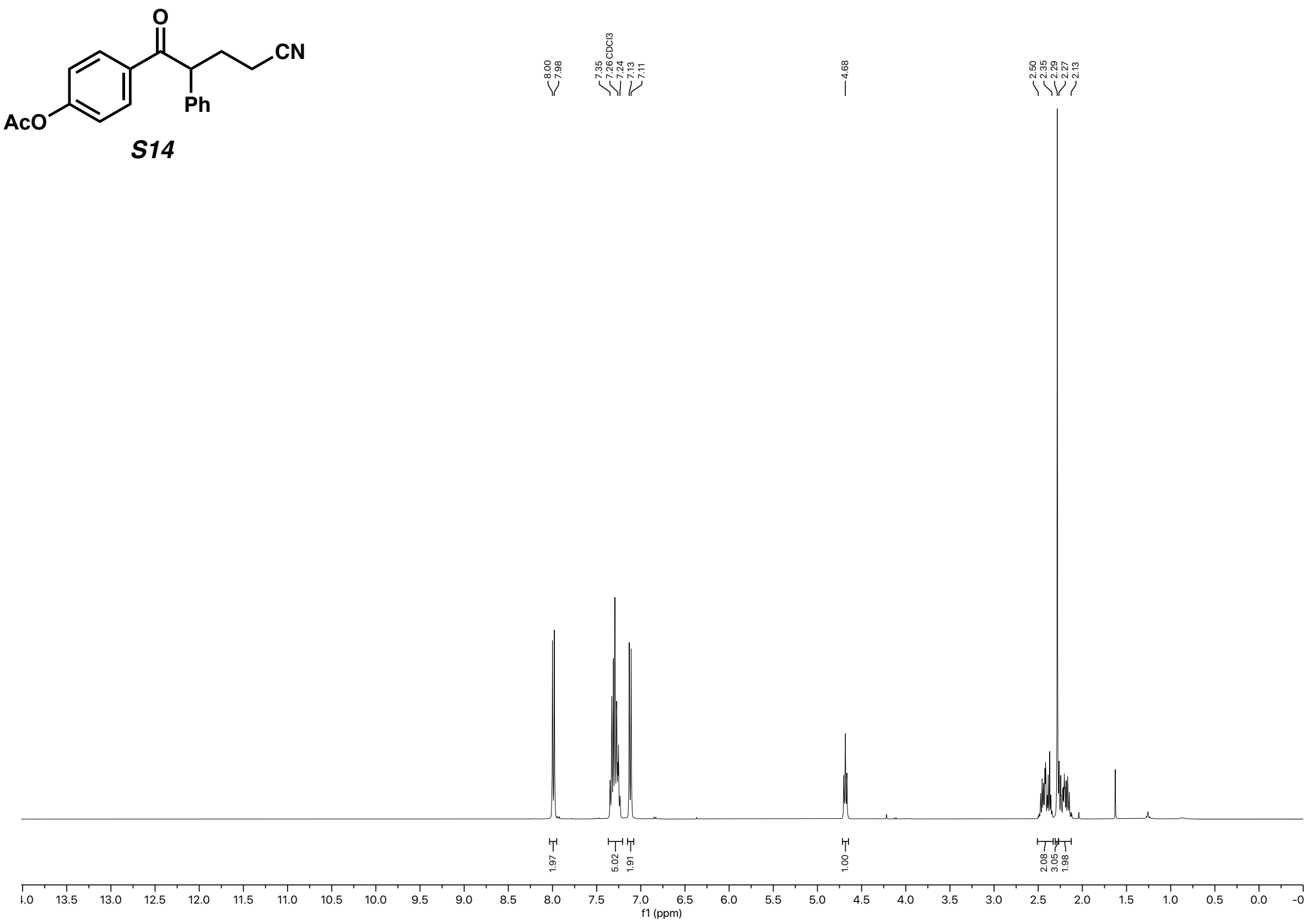

^{1}H NMR (400 MHz, $CDCl_3$) spectrum of compound **S14**.

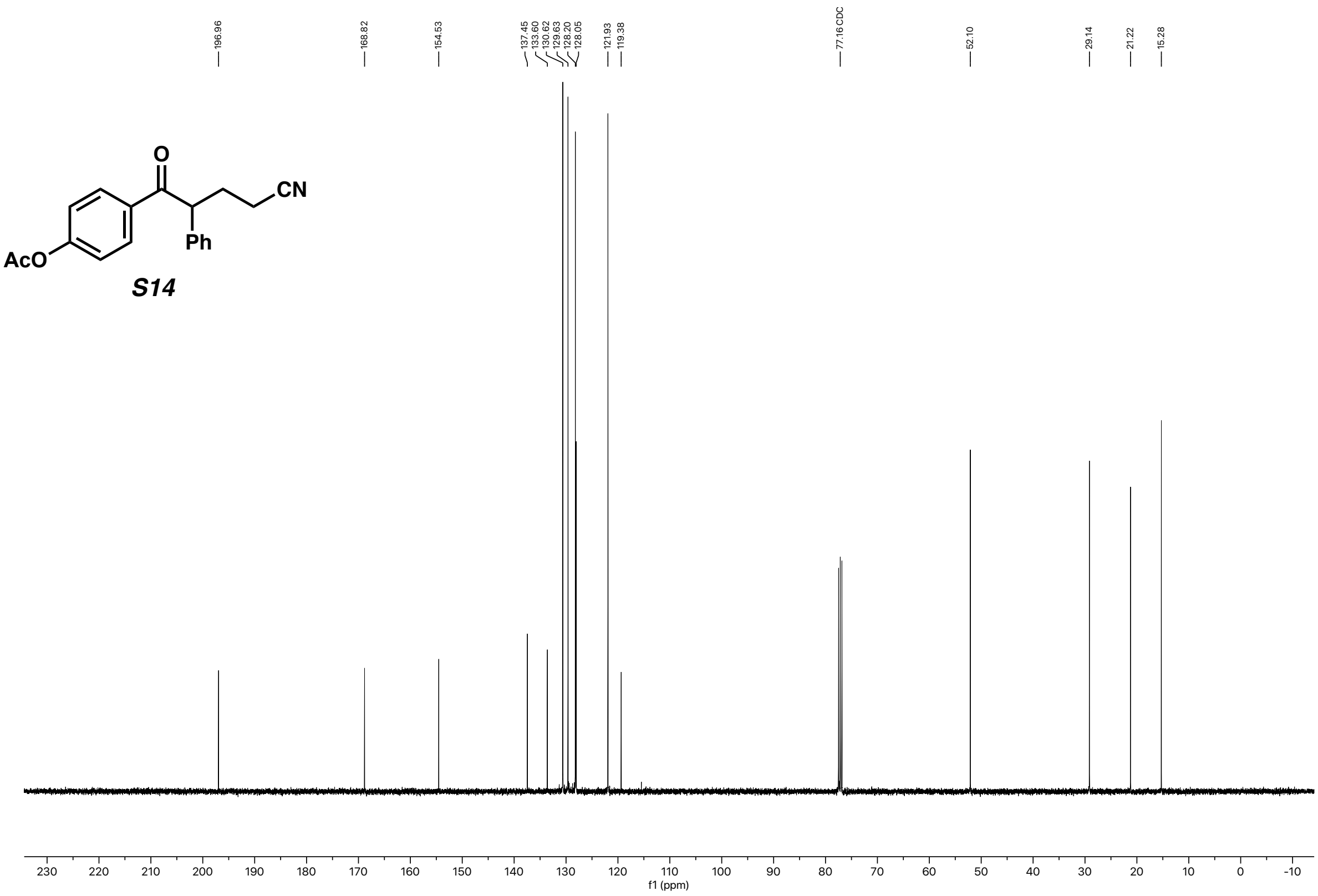

^{13}C NMR (101 MHz, $CDCl_3$) spectrum of compound **S14**.

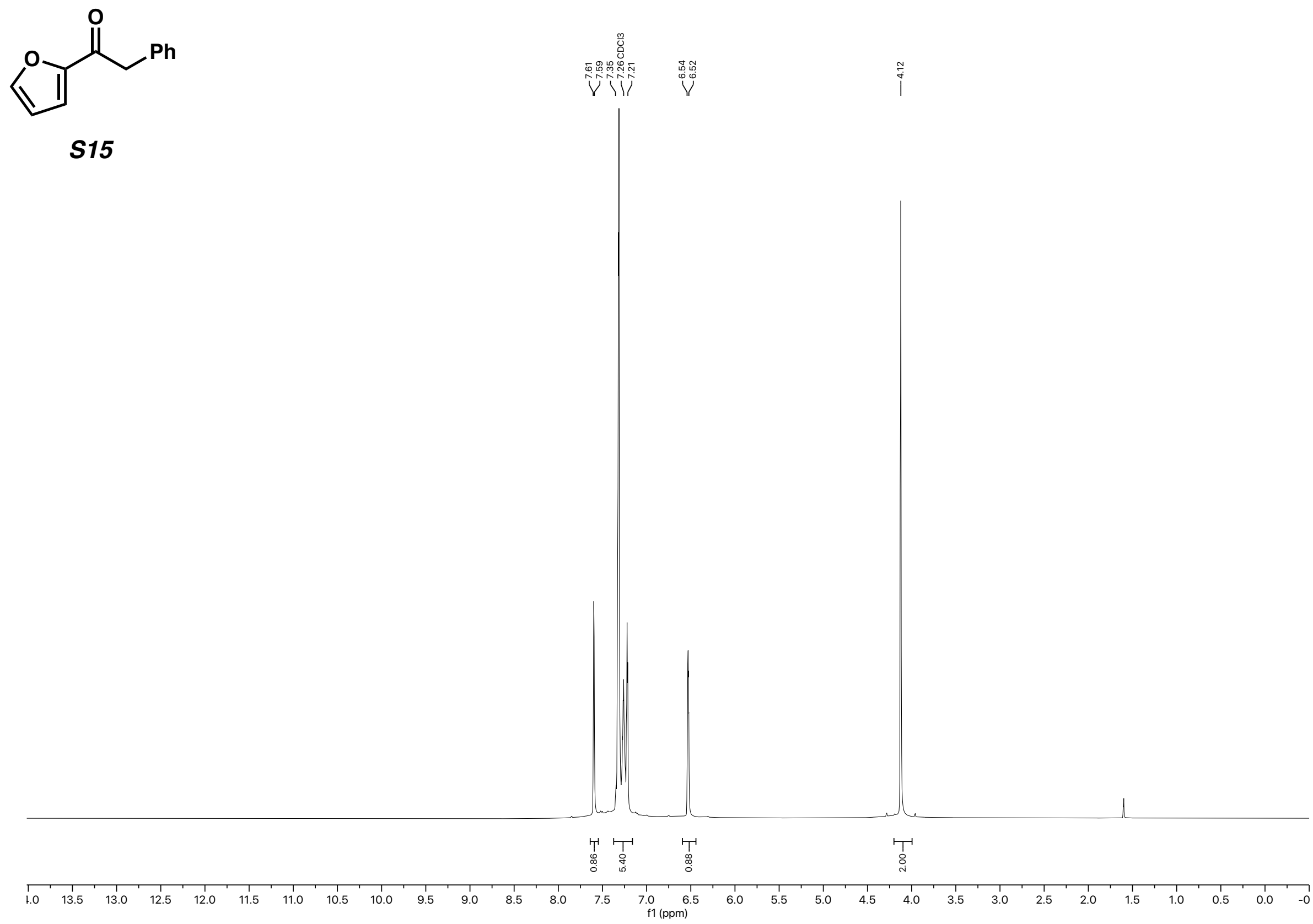

^{1}H NMR (400 MHz, $CDCl_3$) spectrum of compound **S15**.

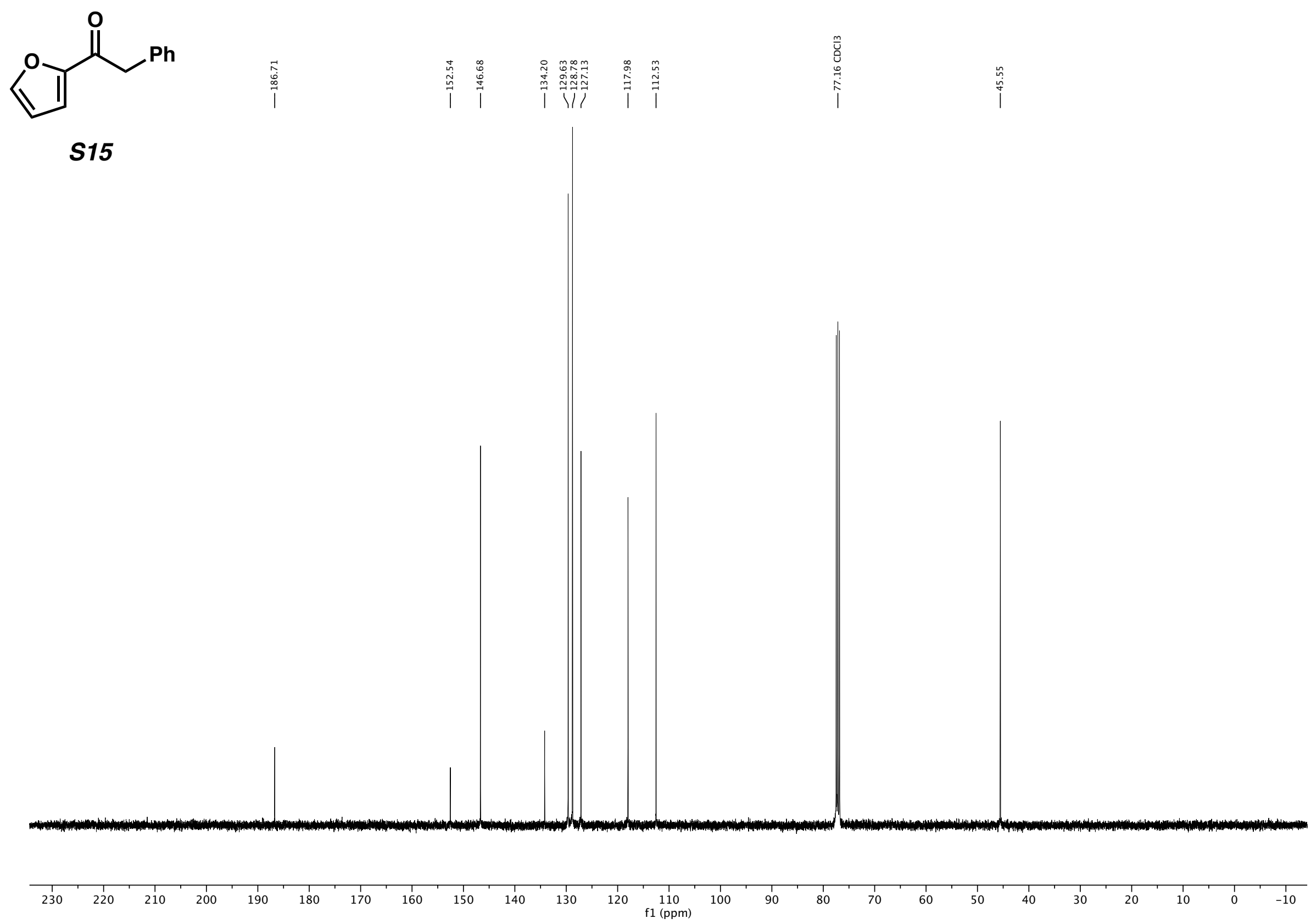

^{13}C NMR (101 MHz, $CDCl_3$) spectrum of compound **S15**.

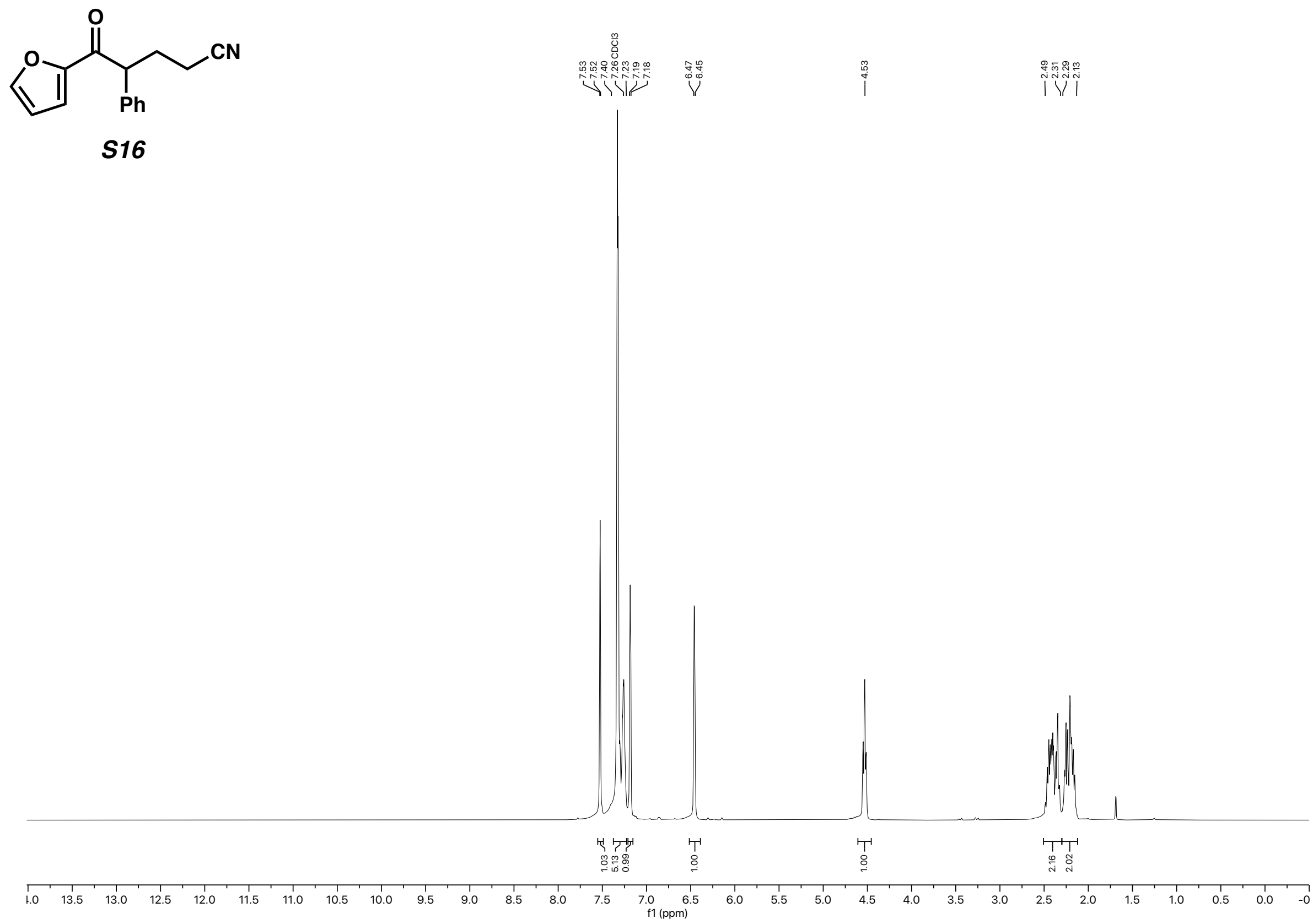

^1H NMR (400 MHz, $CDCl_3$) spectrum of compound **S16**.

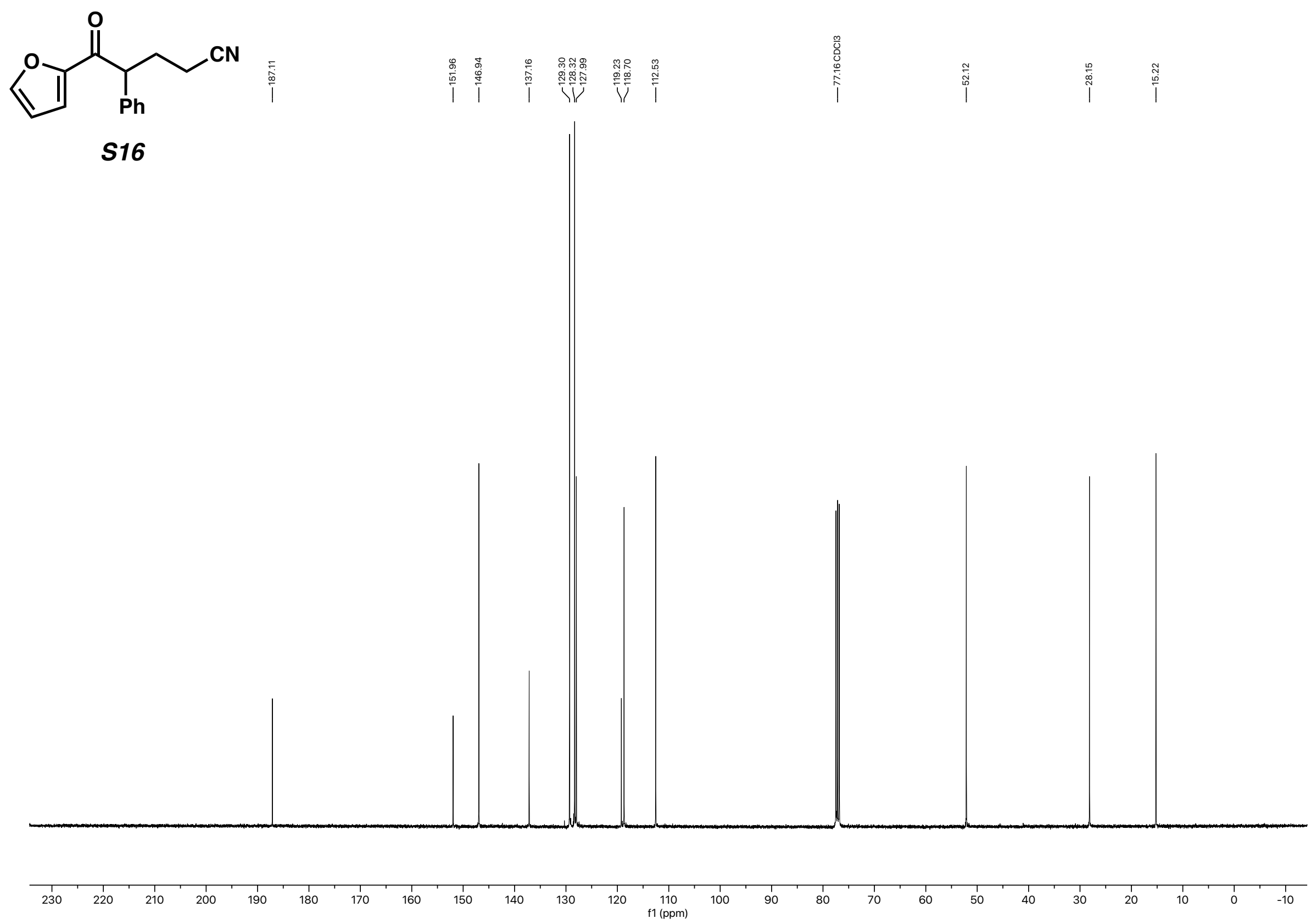

^{13}C NMR (101 MHz, $CDCl_3$) spectrum of compound **S16**.

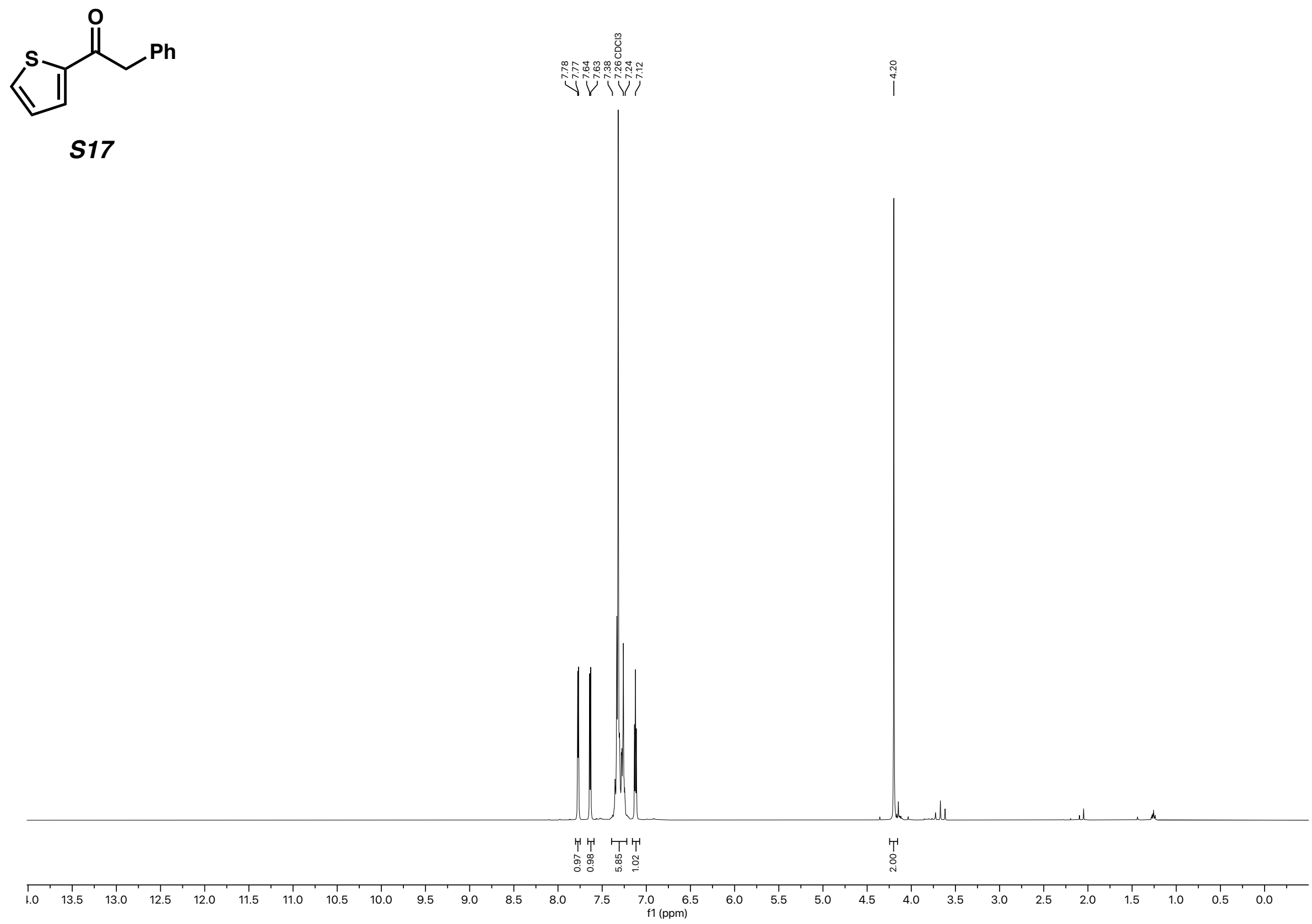

^{1}H NMR (400 MHz, $CDCl_3$) spectrum of compound **S17**.

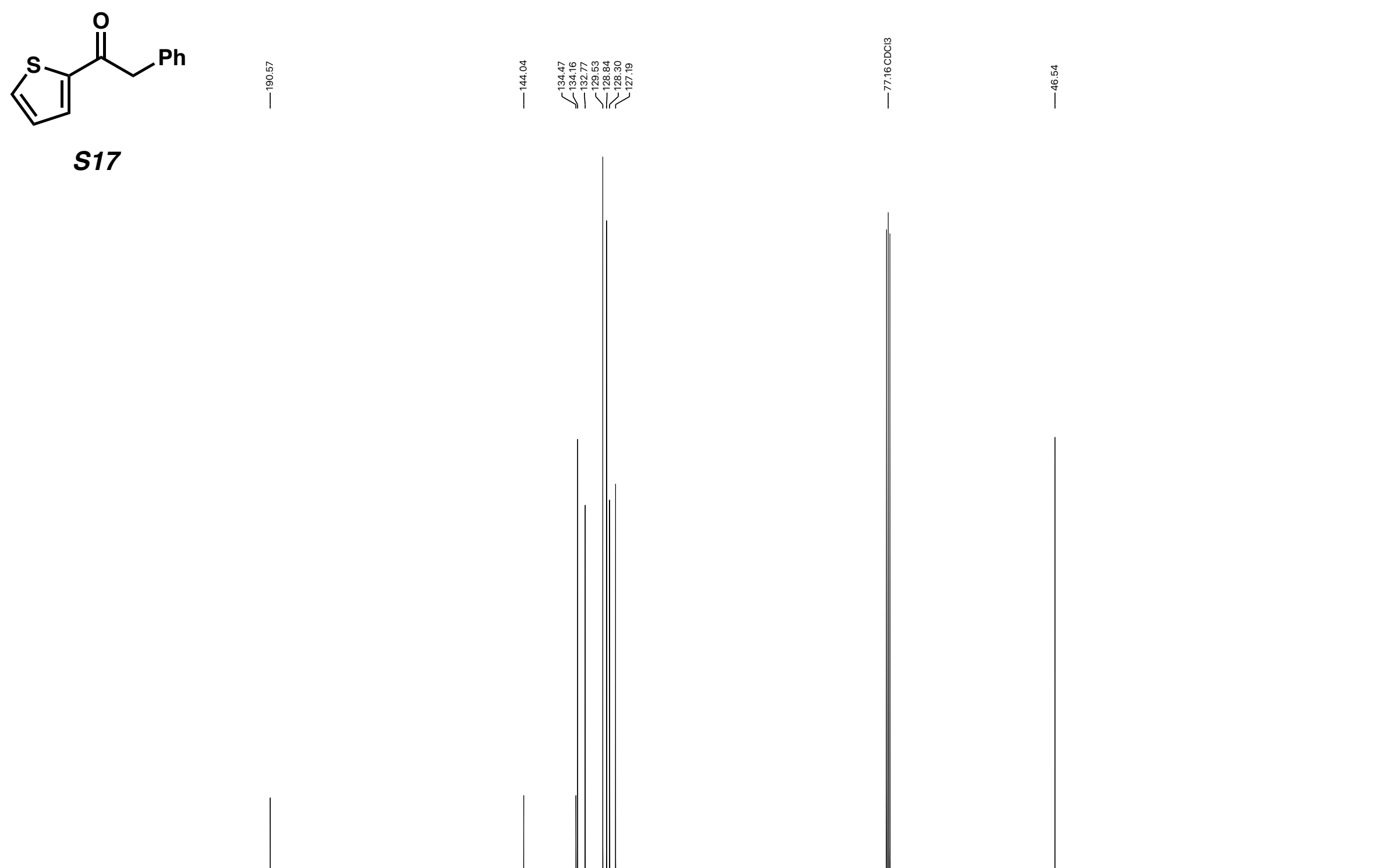

^{13}C NMR (101 MHz, $CDCl_3$) spectrum of compound **S17**.

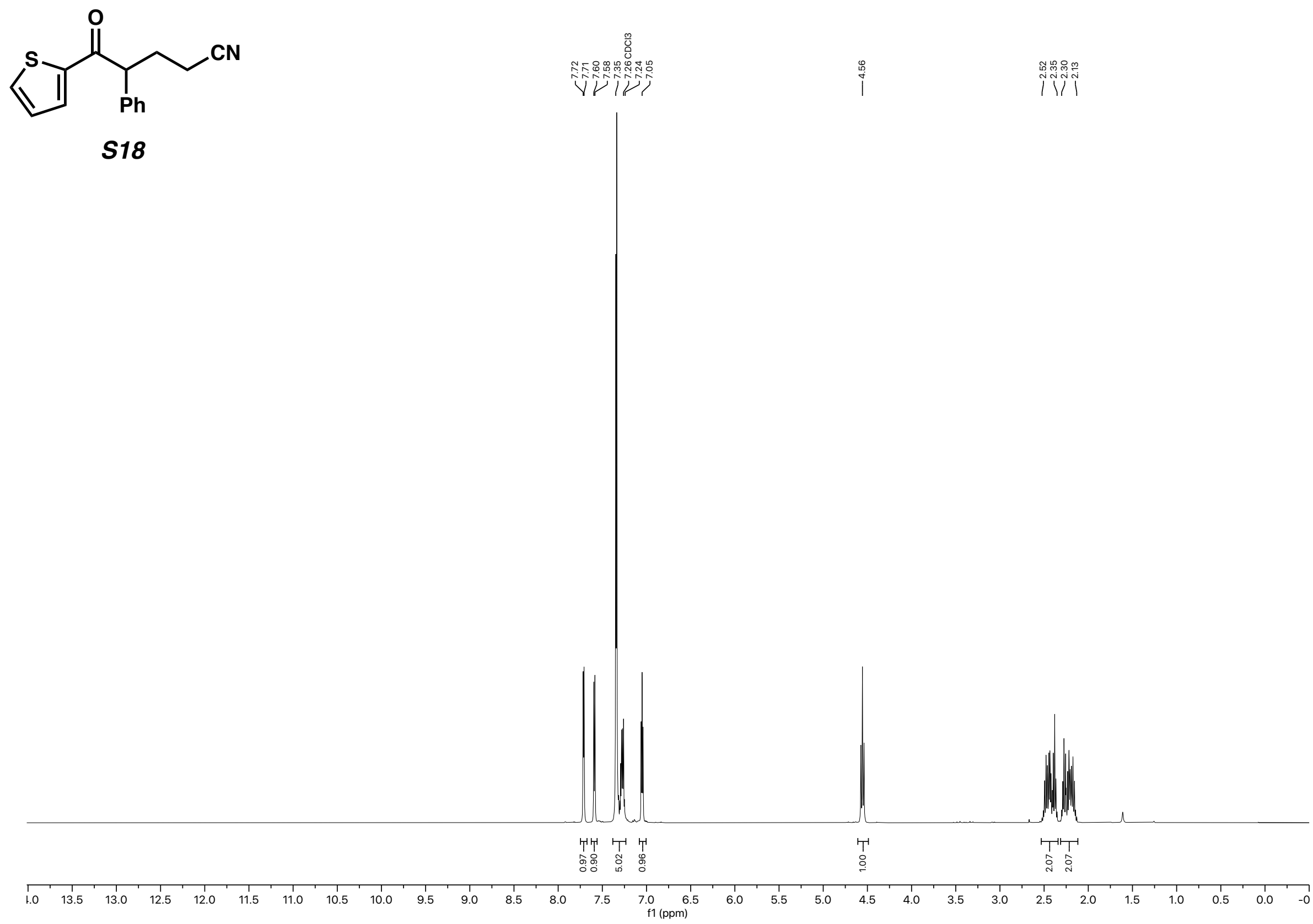

^{1}H NMR (400 MHz, $CDCl_3$) spectrum of compound **S18**.

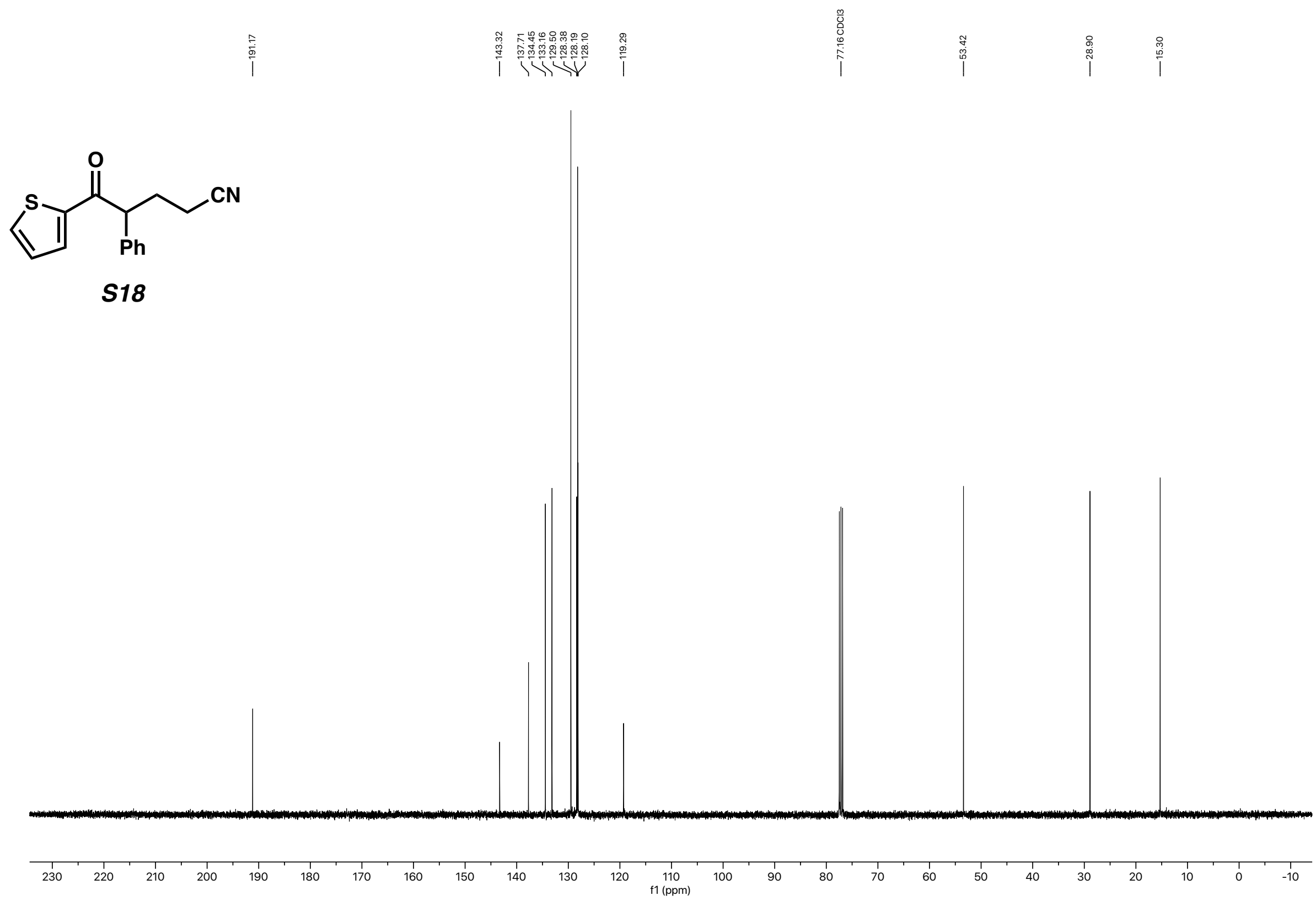

^{13}C NMR (101 MHz, $CDCl_3$) spectrum of compound **S18**.

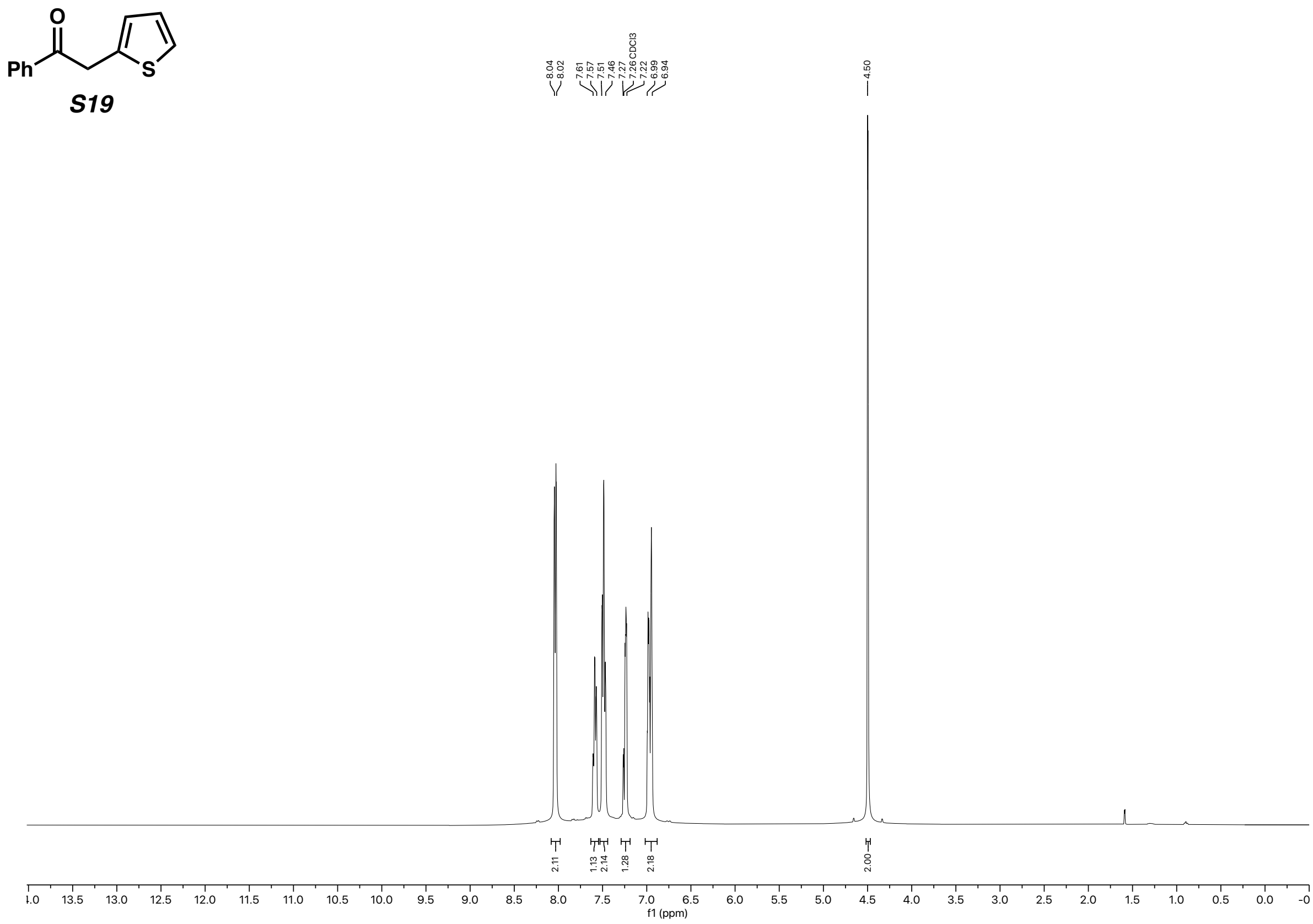

^{1}H NMR (400 MHz, $CDCl_3$) spectrum of compound **S19**.

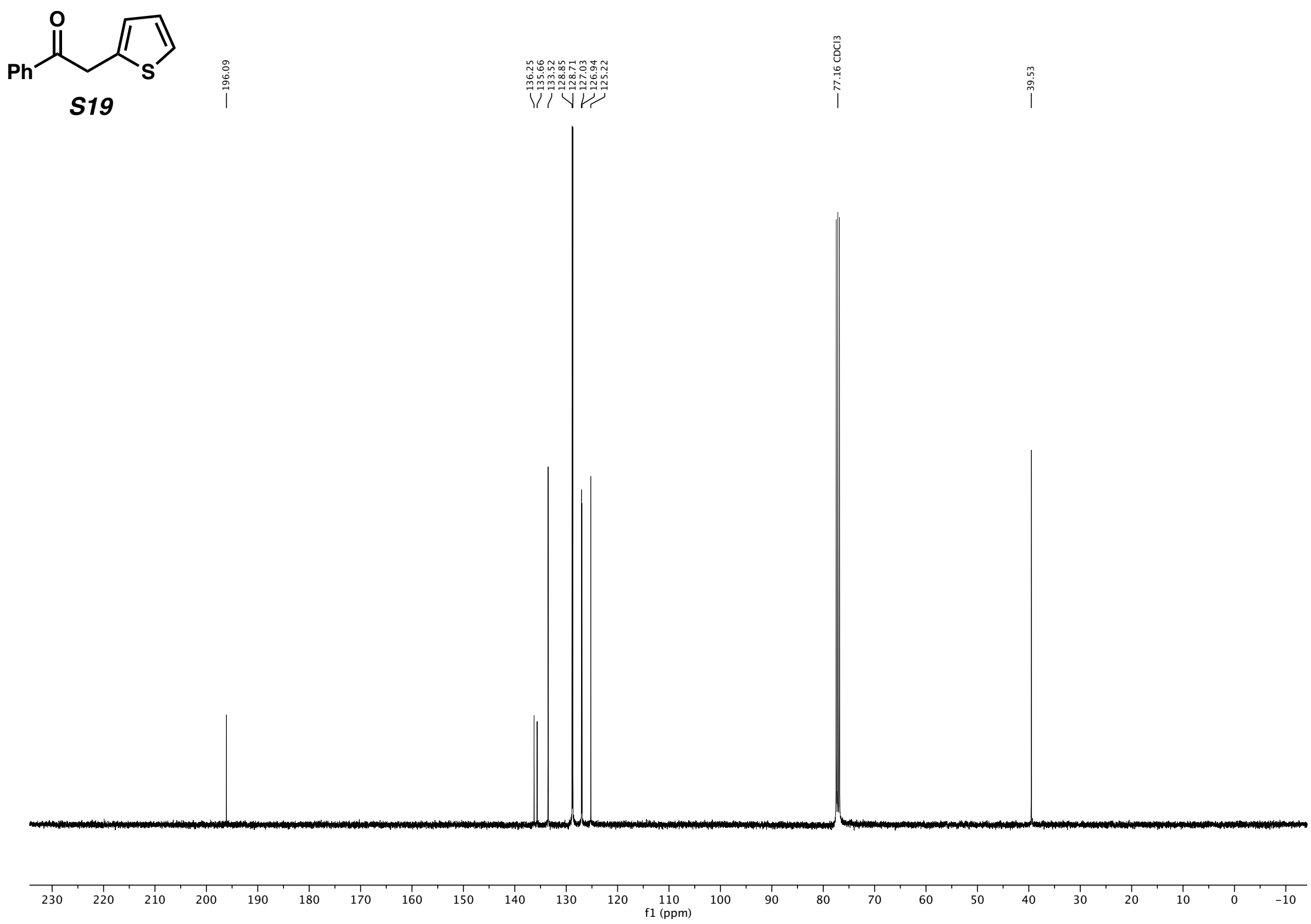

^{13}C NMR (101 MHz, $CDCl_3$) spectrum of compound **S19**.

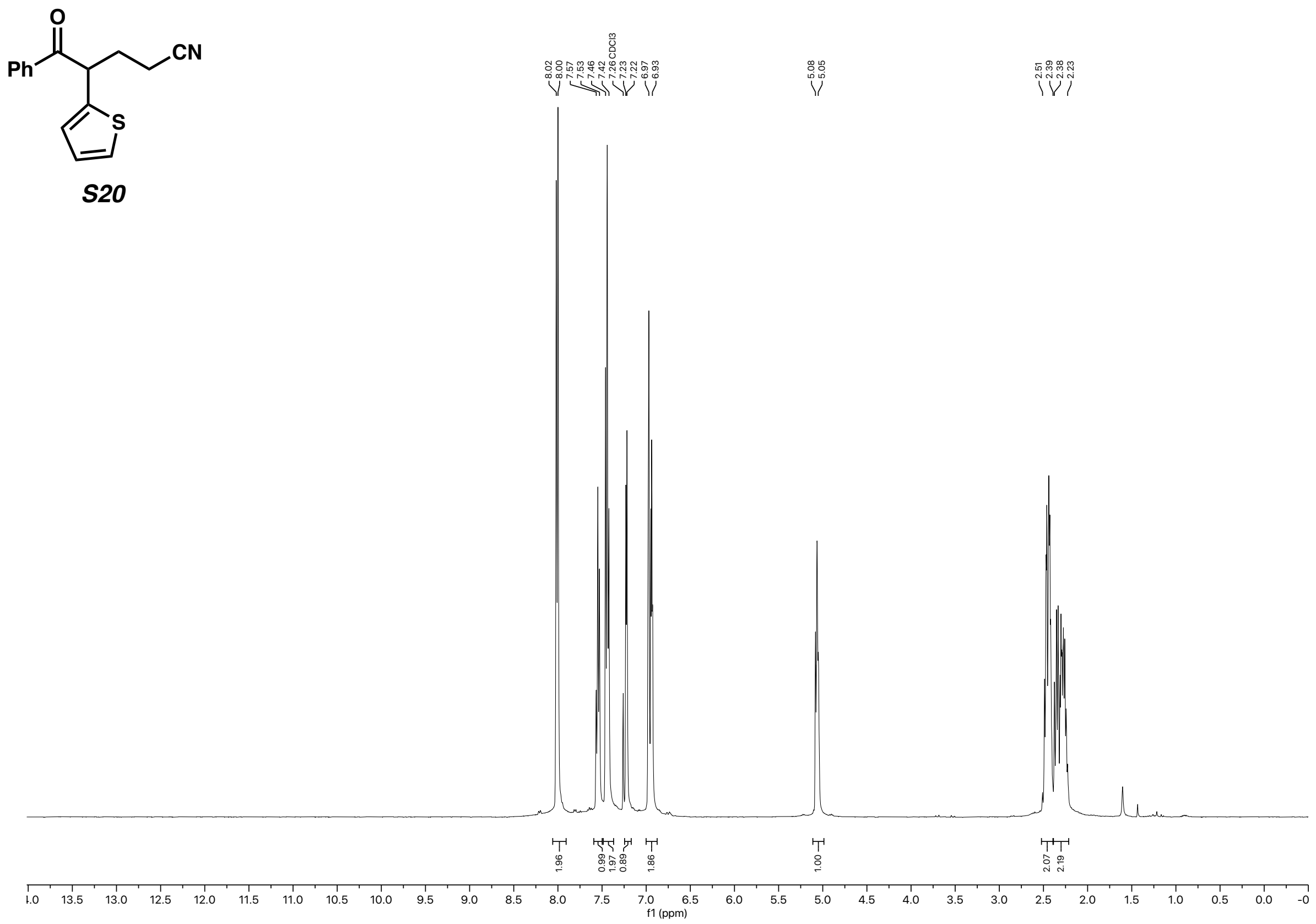

^{1}H NMR (400 MHz, CDCl₃) spectrum of compound **S20**.

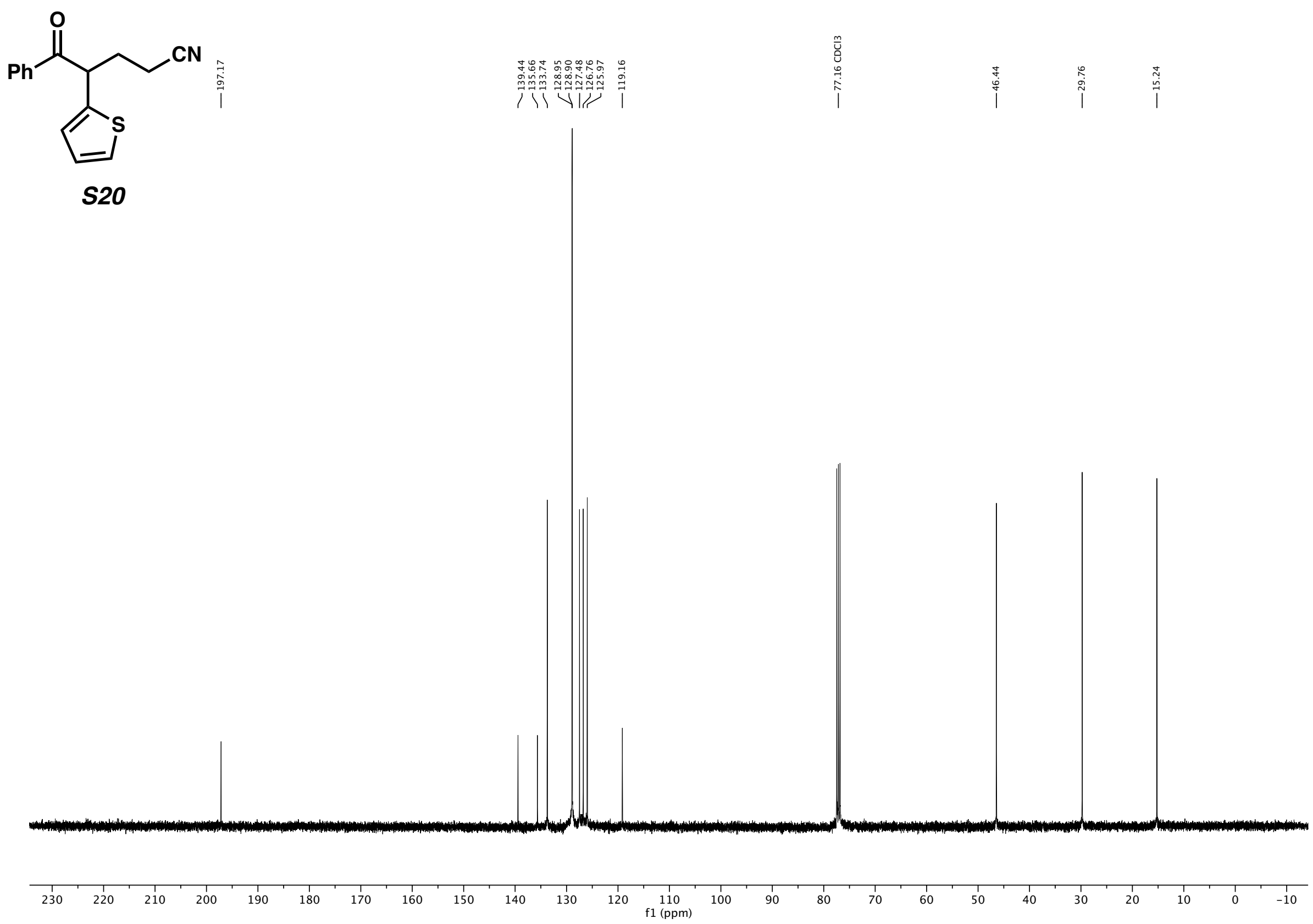

^{13}C NMR (101 MHz, CDCl₃) spectrum of compound **S20**.

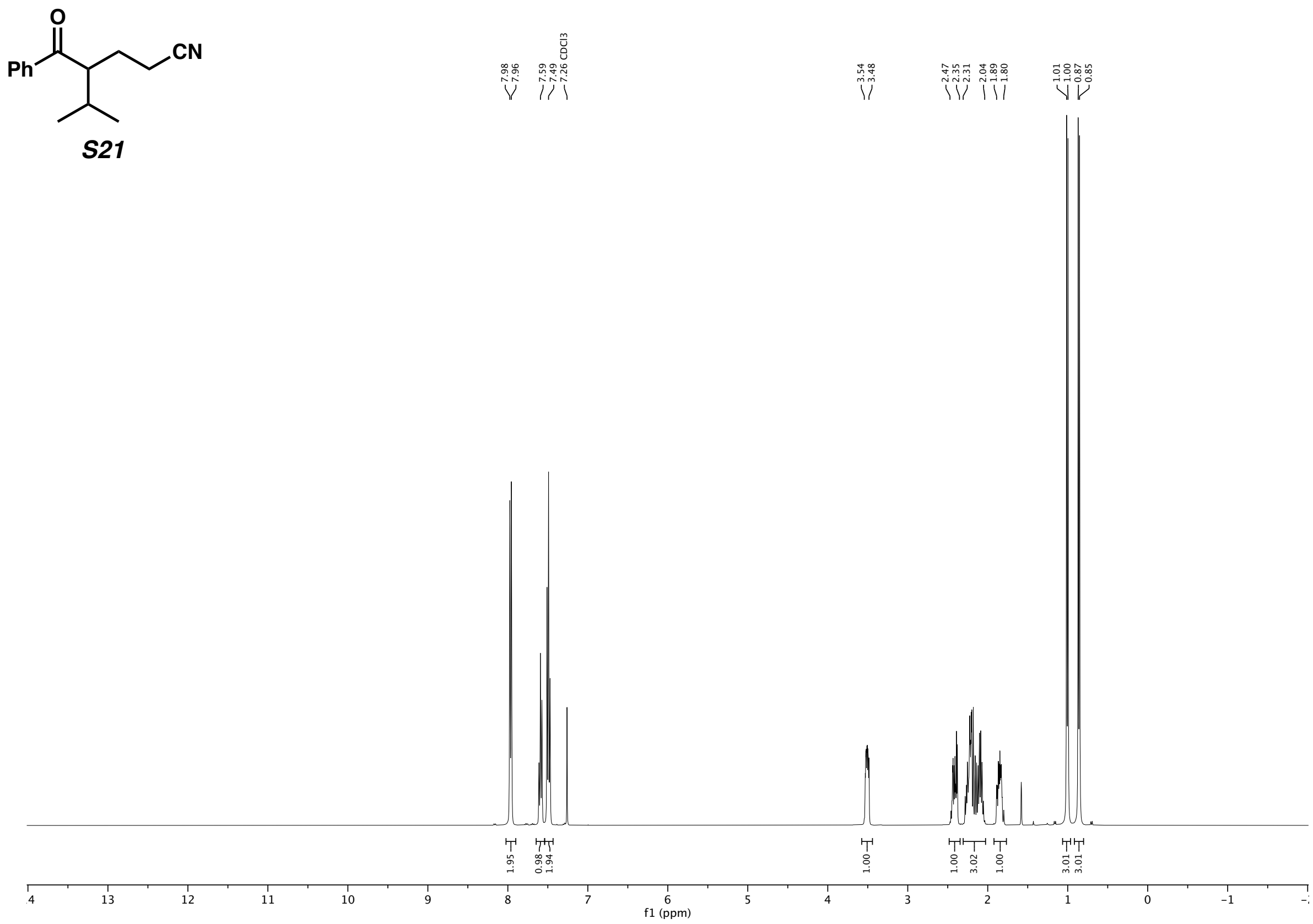

^{1}H NMR (400 MHz, $CDCl_3$) spectrum of compound **S21**.

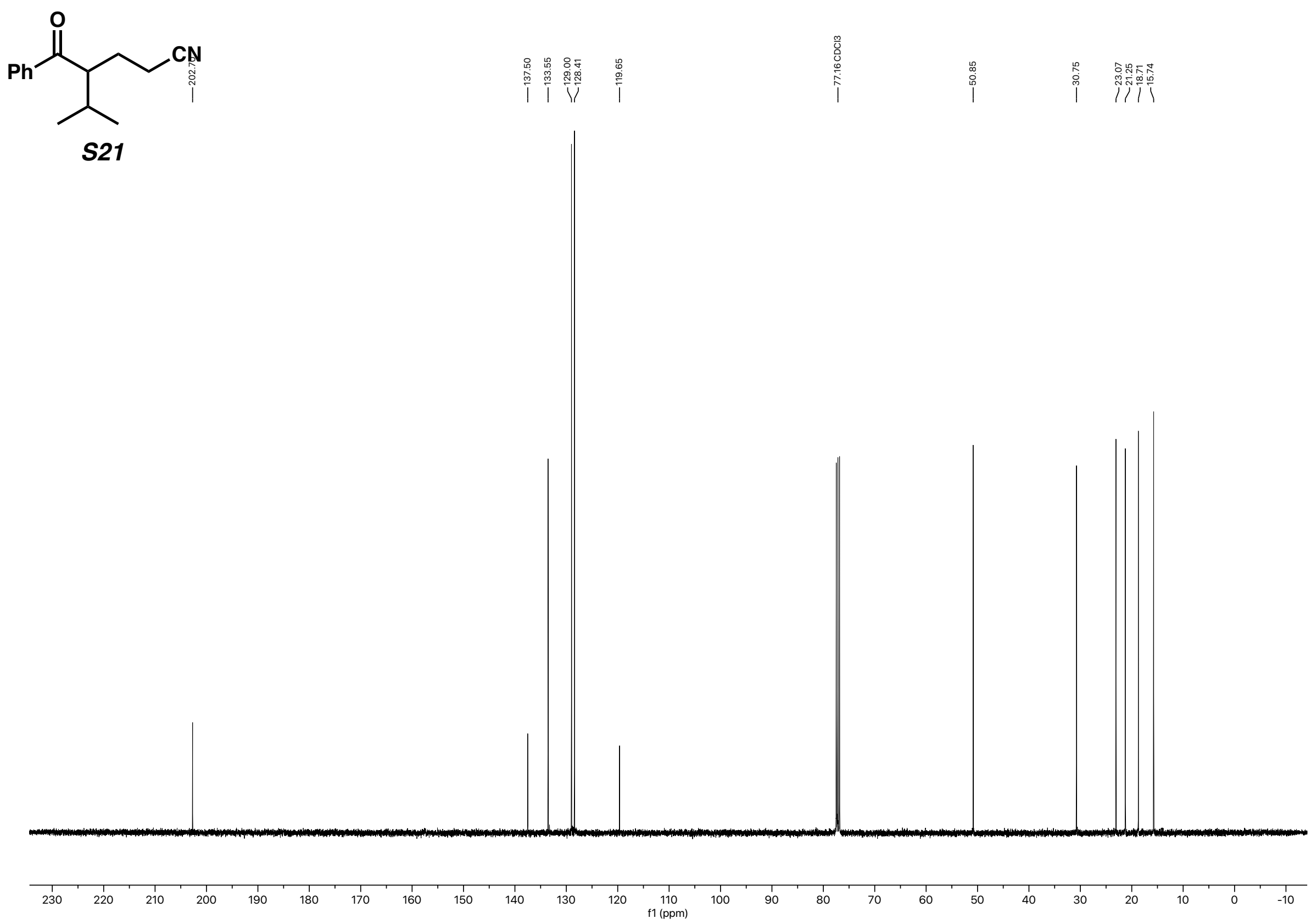

^{13}C NMR (101 MHz, $CDCl_3$) spectrum of compound **S21**.

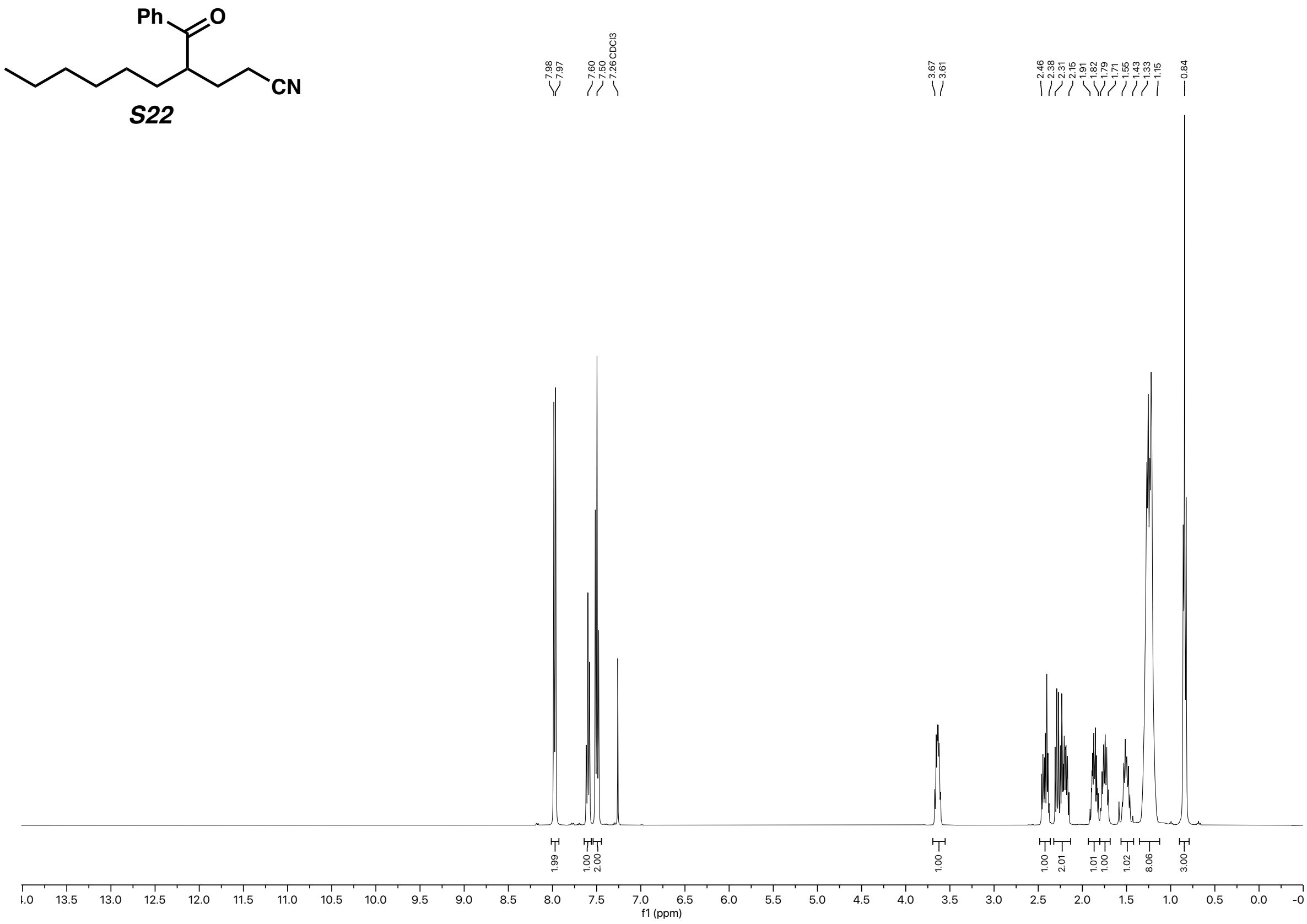

^{1}H NMR (400 MHz, $CDCl_3$) spectrum of compound **S22**.

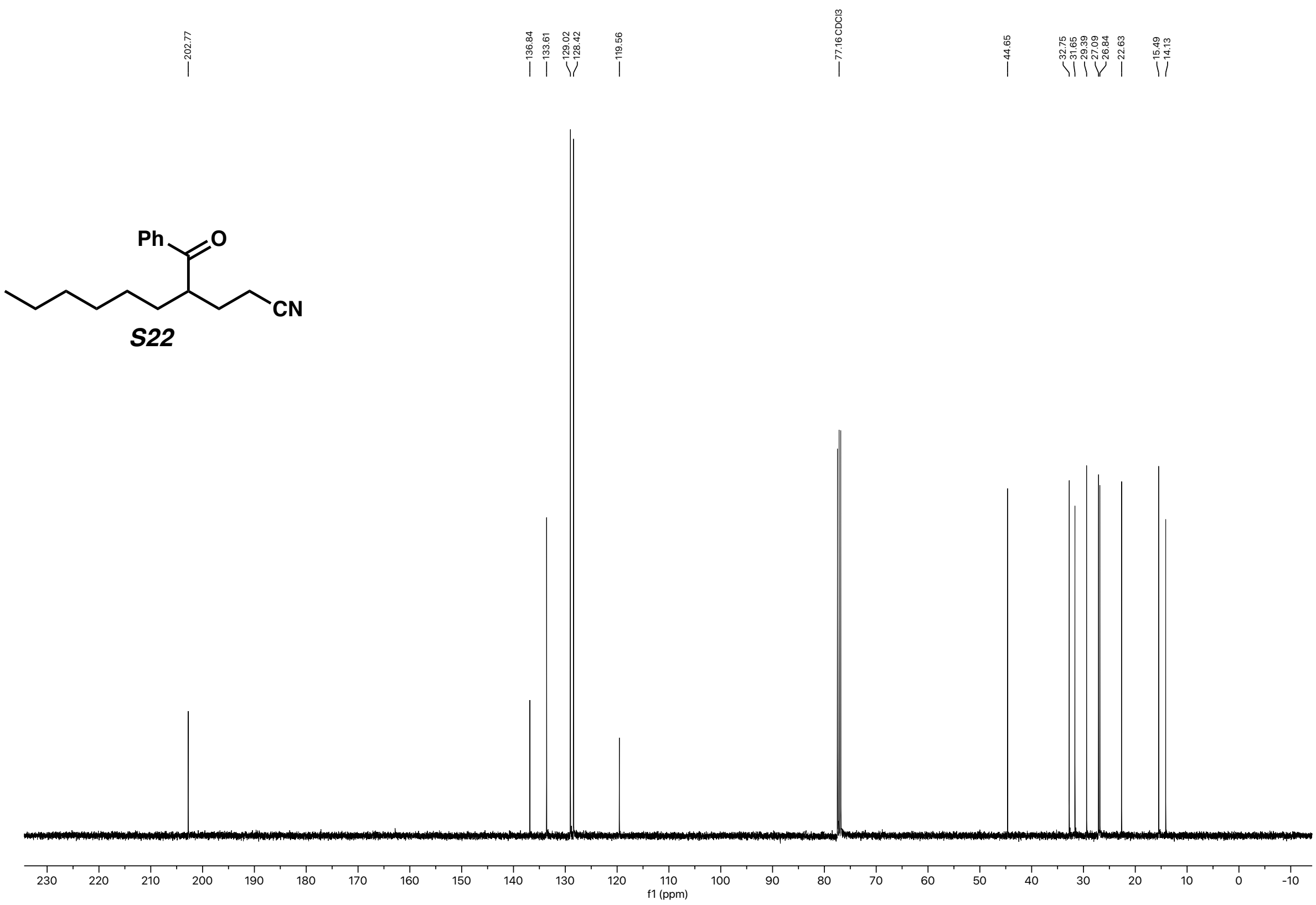

^{13}C NMR (101 MHz, $CDCl_3$) spectrum of compound **S22**.

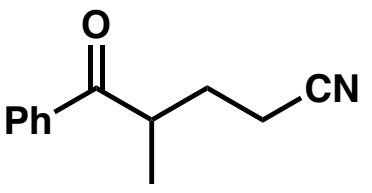

S23

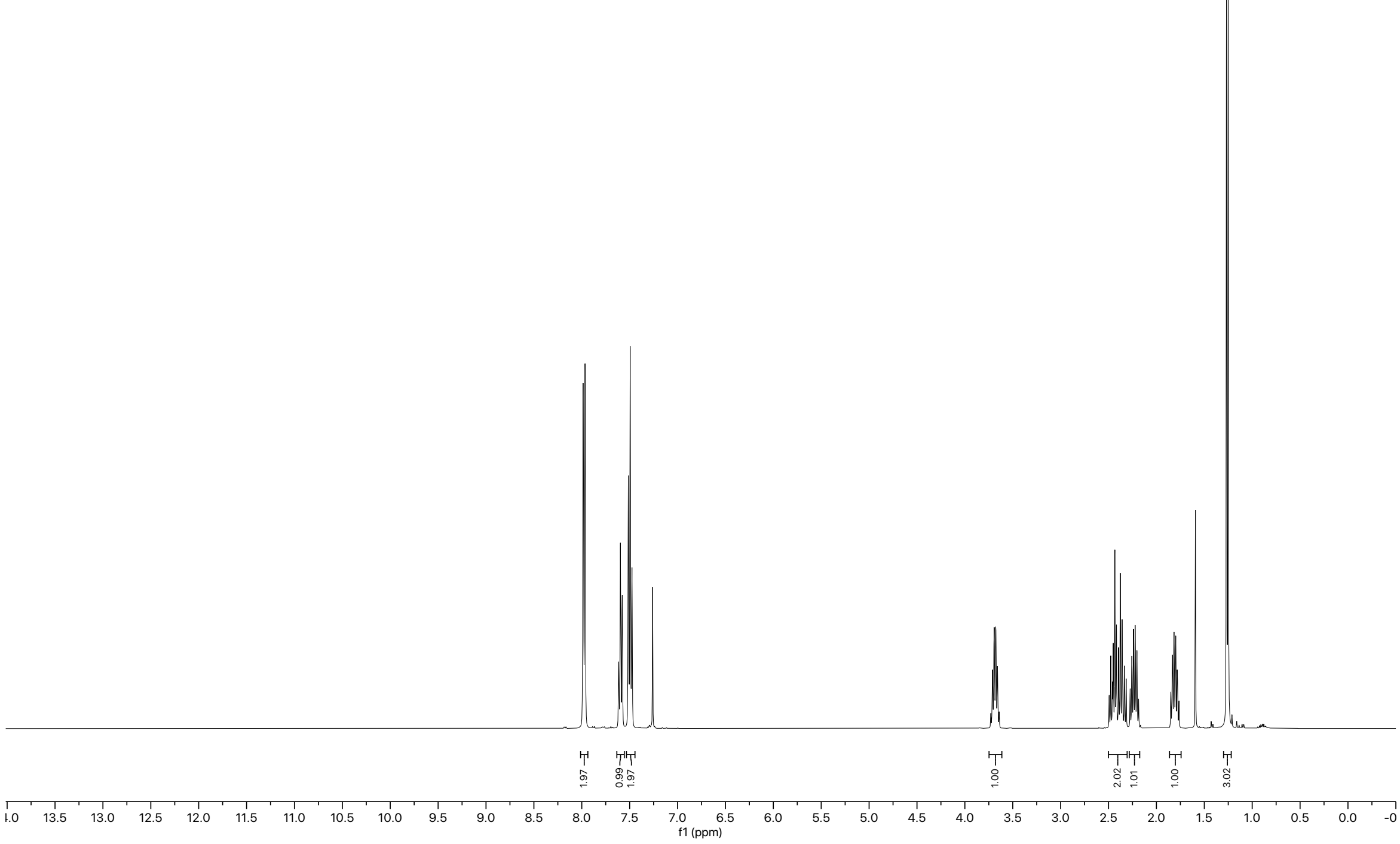

^{1}H NMR (400 MHz, $CDCl_3$) spectrum of compound **S23**.

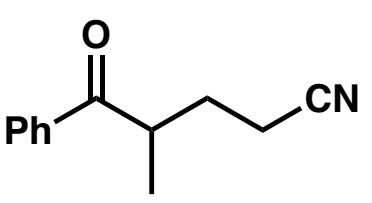

S23

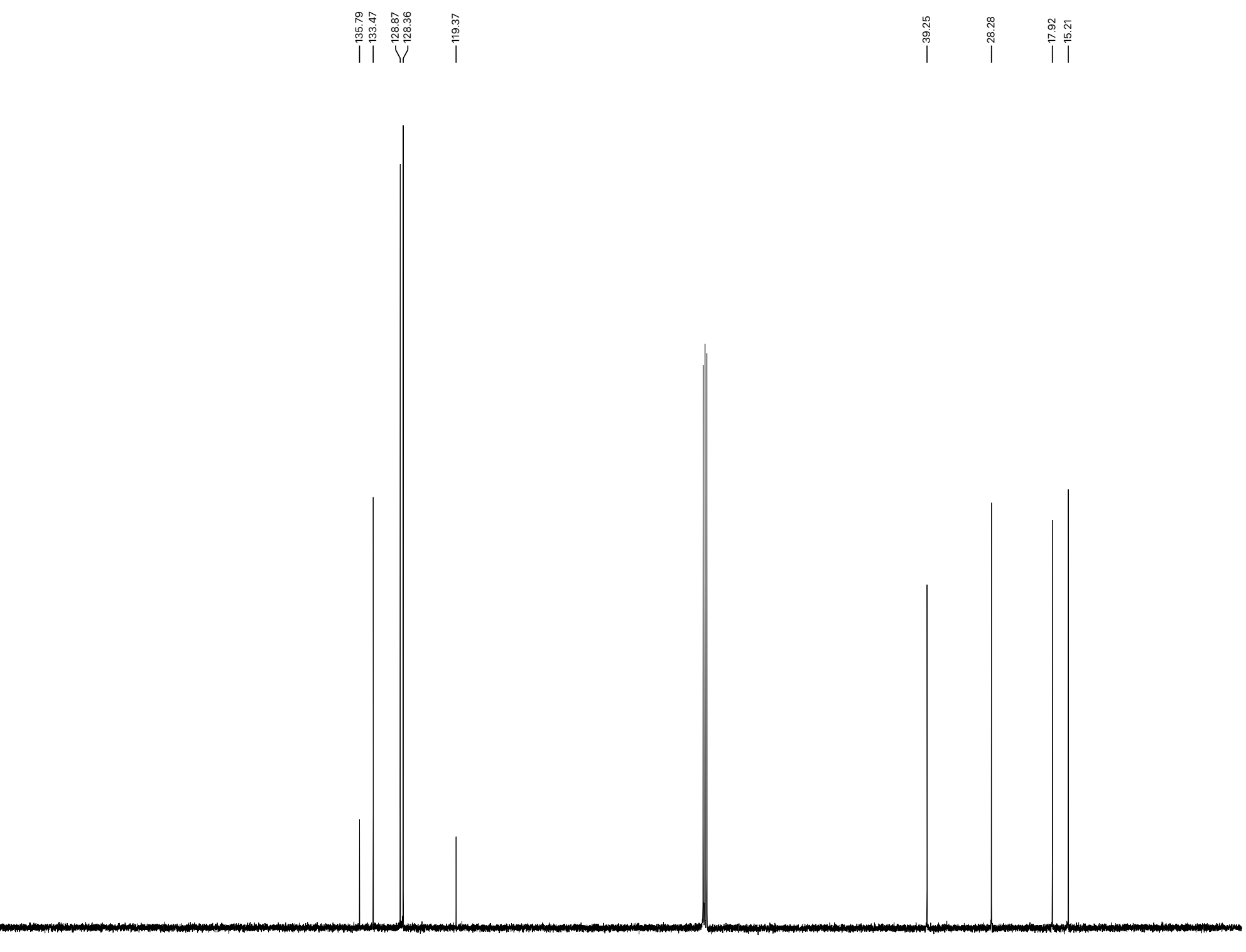

^{13}C NMR (101 MHz, $CDCl_3$) spectrum of compound **S23**.

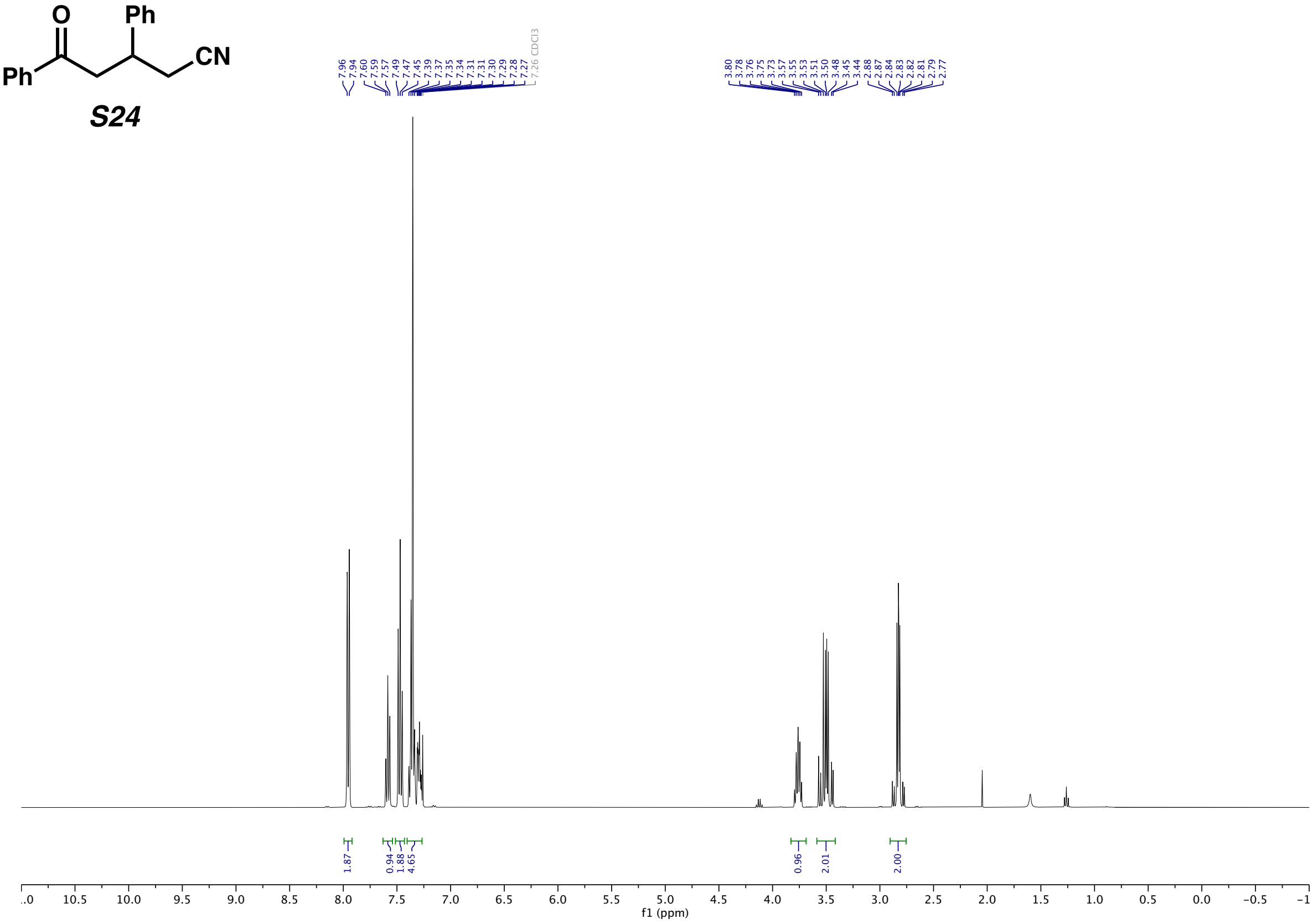

^{1}H NMR (400 MHz, $CDCl_3$) spectrum of compound **S24**.

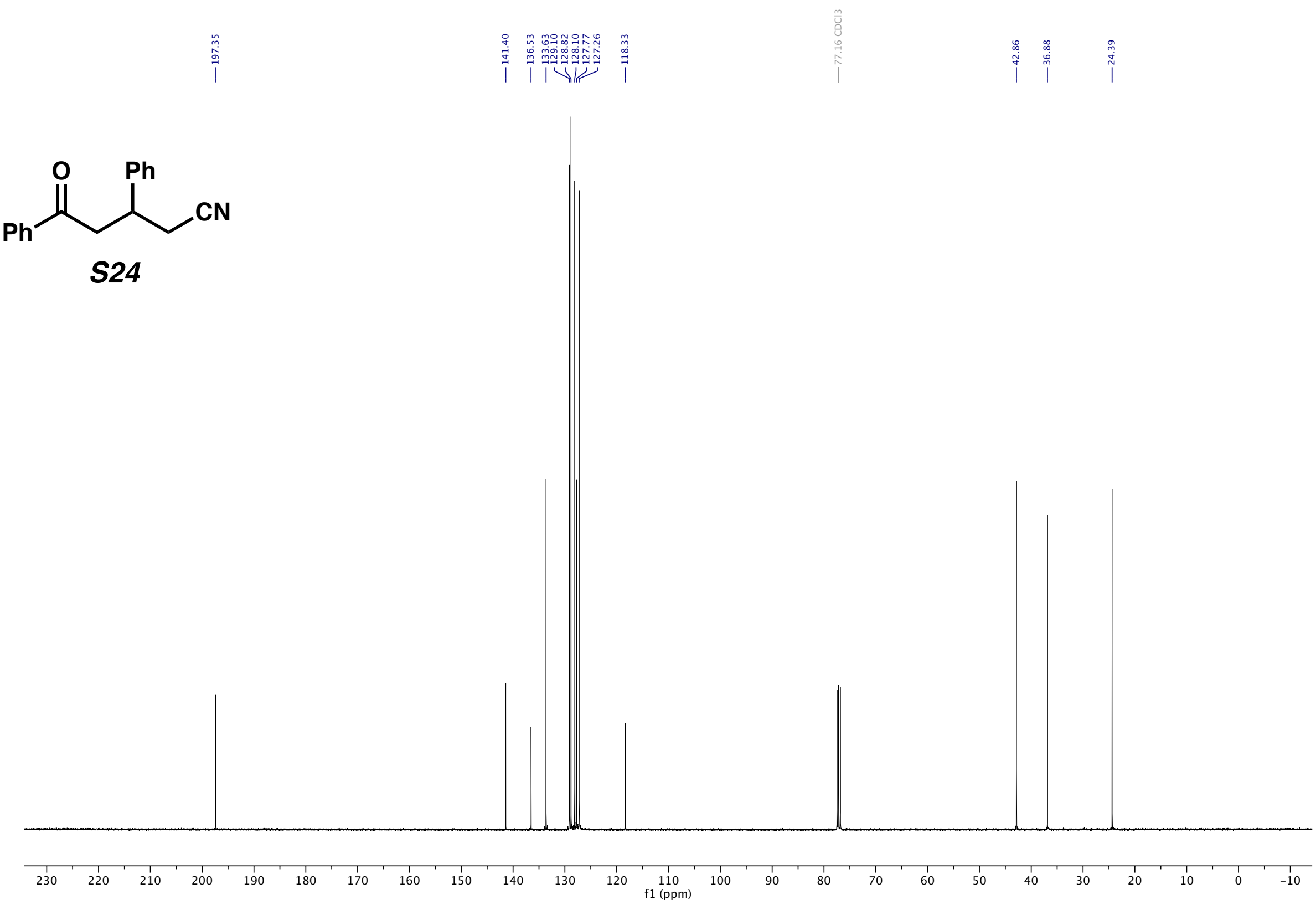

^{13}C NMR (101 MHz, $CDCl_3$) spectrum of compound **S24**.

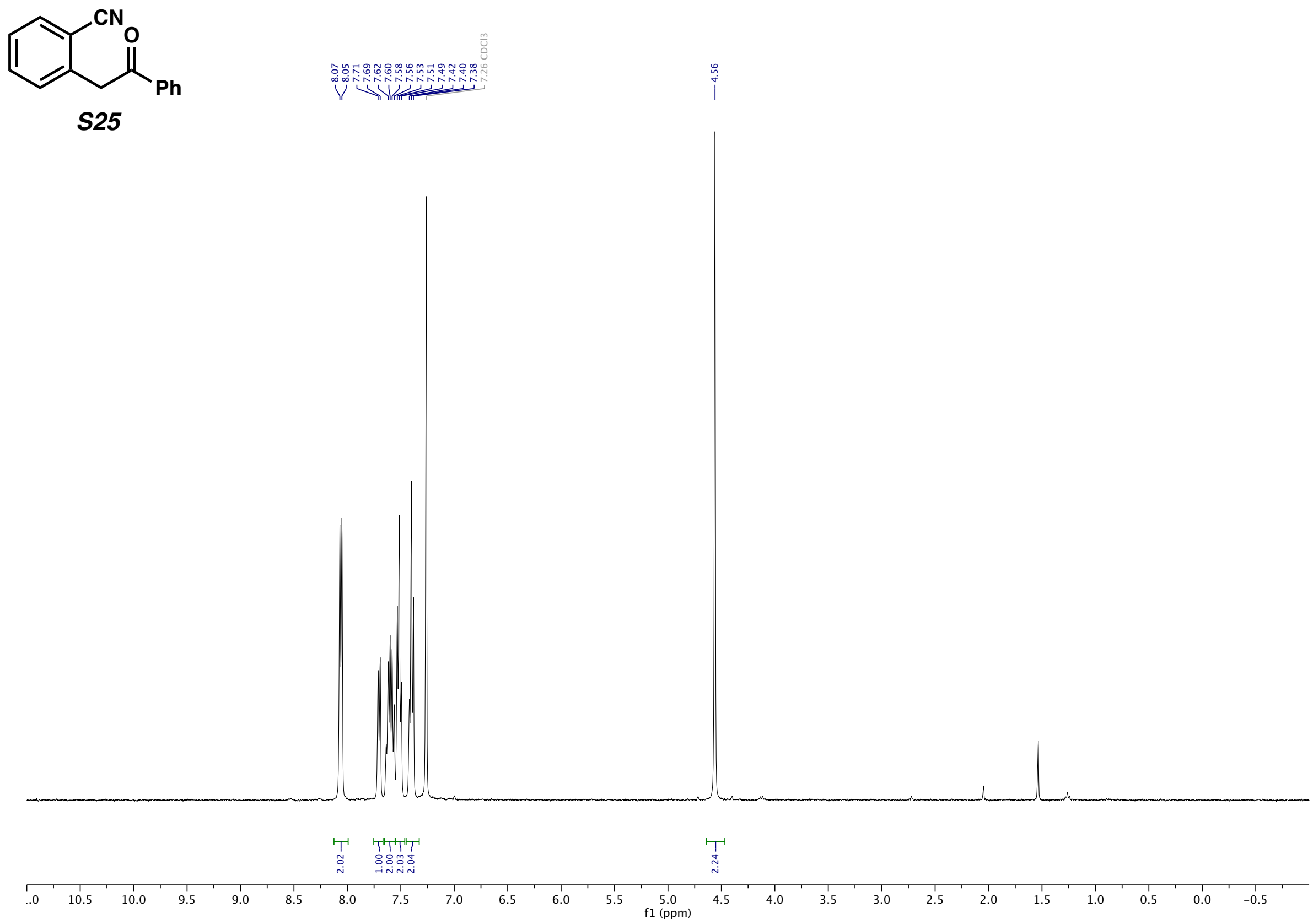

^{1}H NMR (400 MHz, $CDCl_3$) spectrum of compound **S25**.

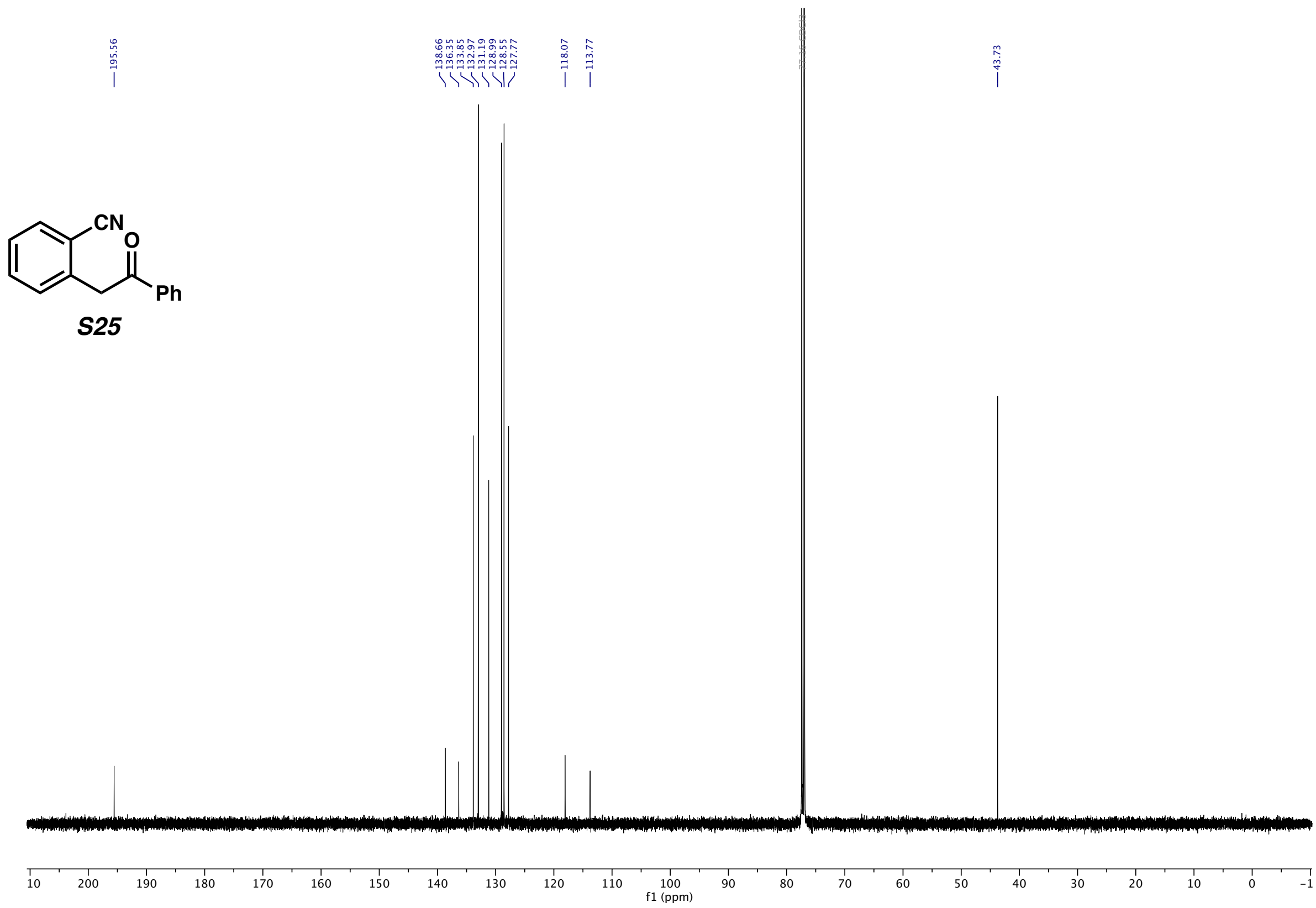

^{13}C NMR (126 MHz, $CDCl_3$) spectrum of compound **S25**.

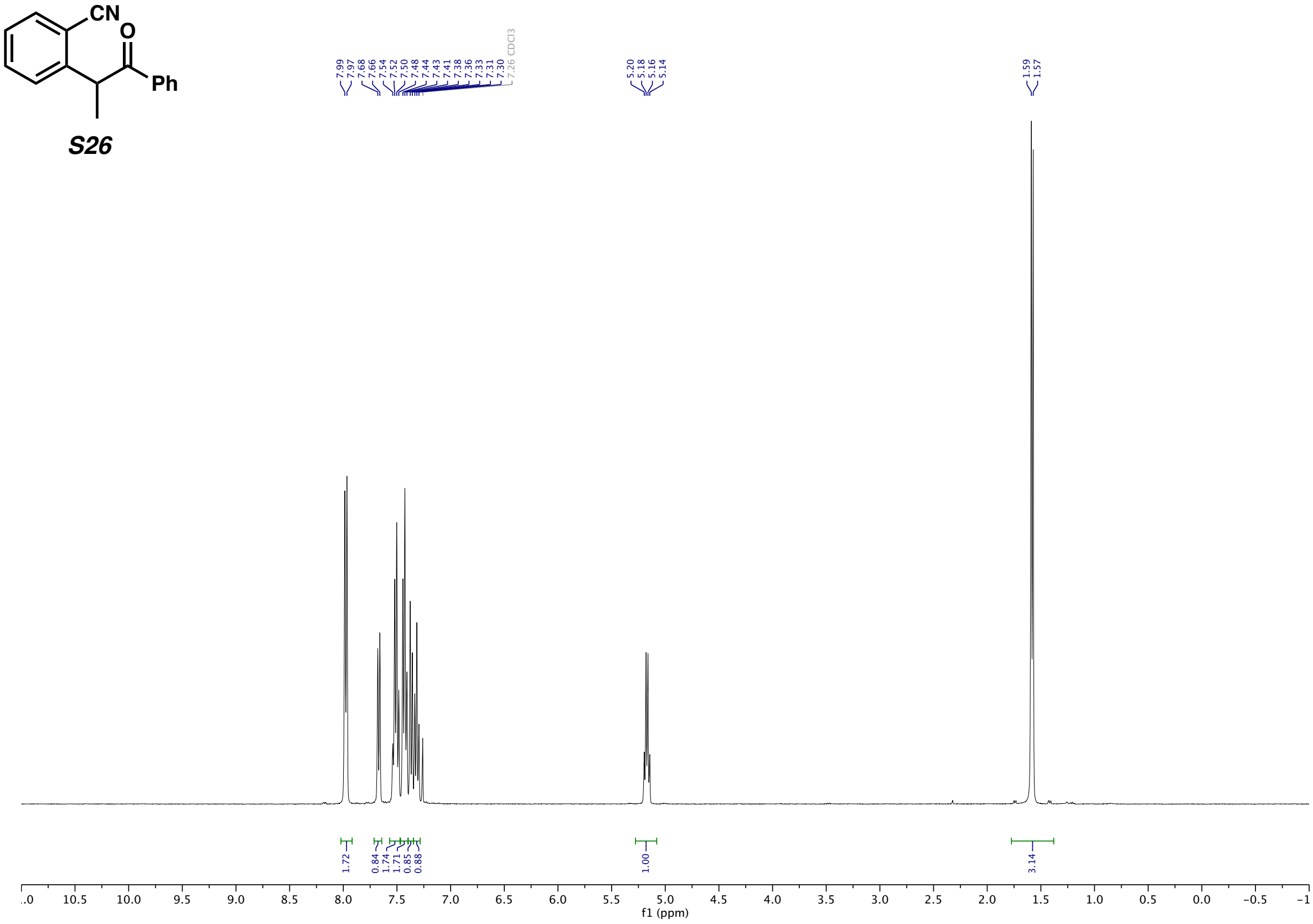

^{1}H NMR (400 MHz, $CDCl_3$) spectrum of compound **S26**.

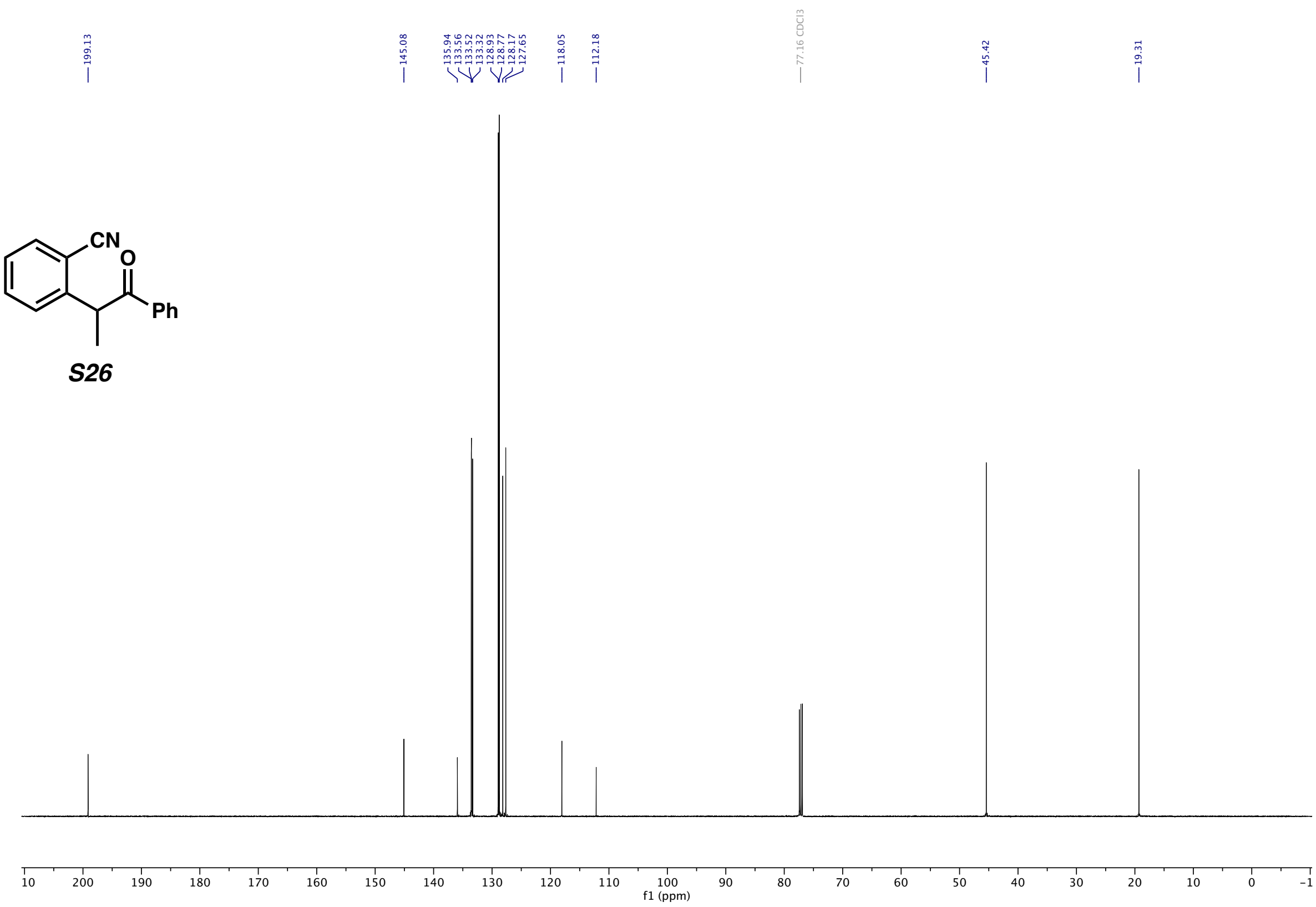

^{13}C NMR (126 MHz, $CDCl_3$) spectrum of compound **S26**.

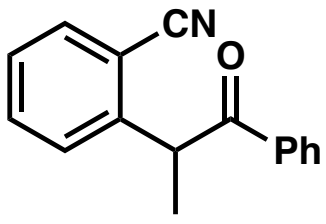

S26

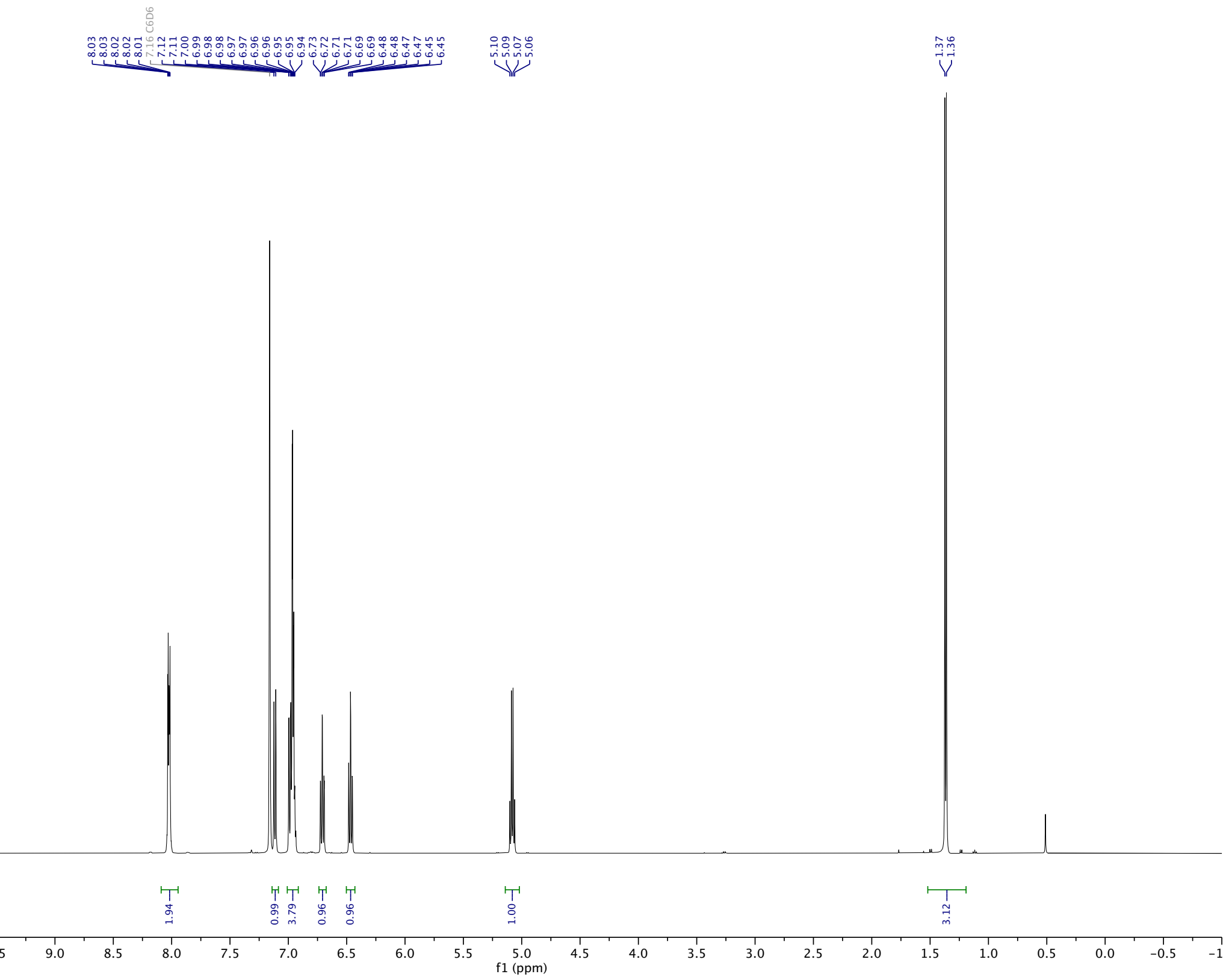

^{1}H NMR (500 MHz, C_6D_6) spectrum of compound **S26**.

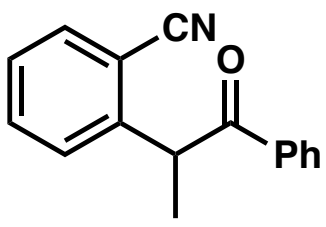

S26

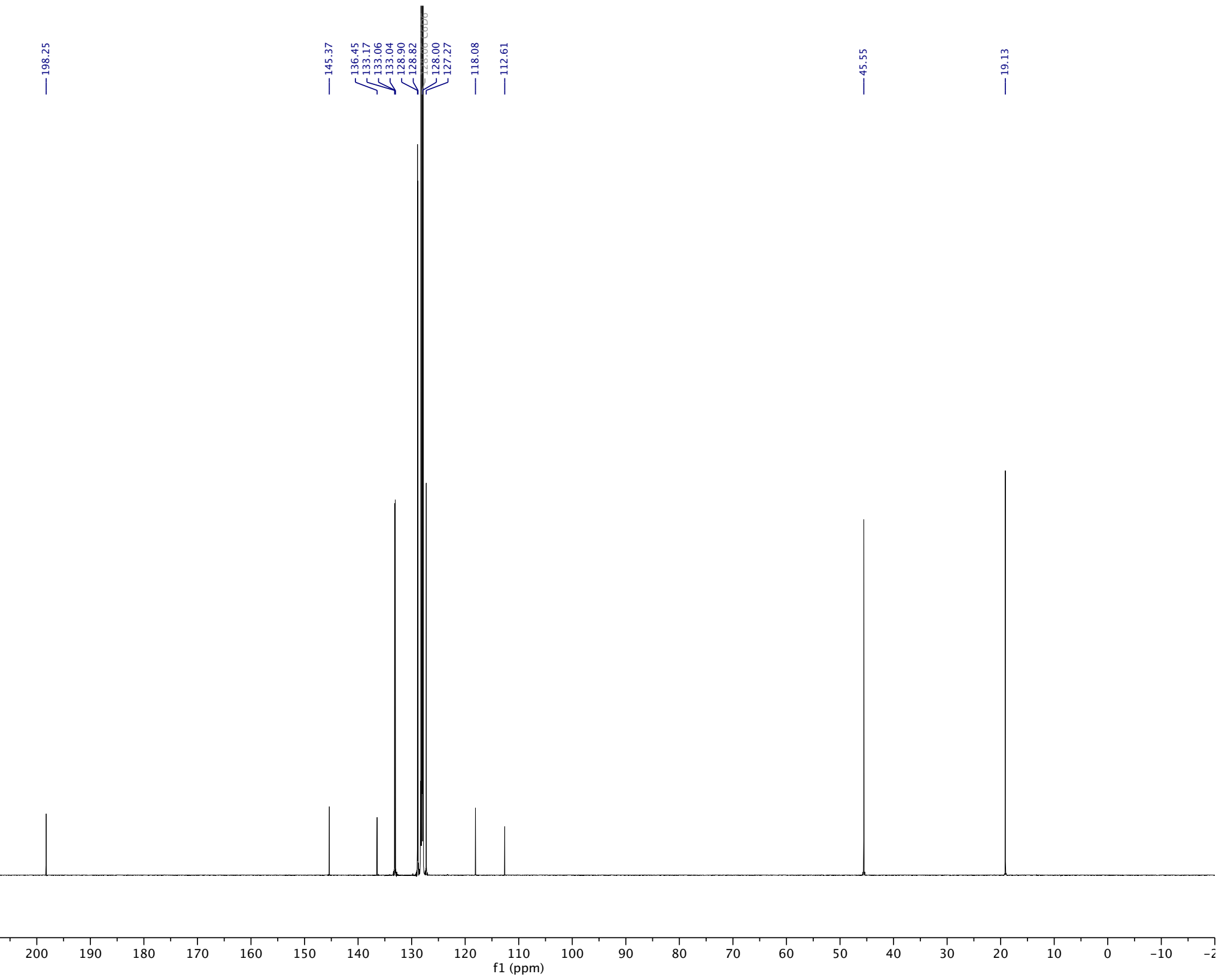

^{13}C NMR (126 MHz, C_6D_6) spectrum of compound **S26**.

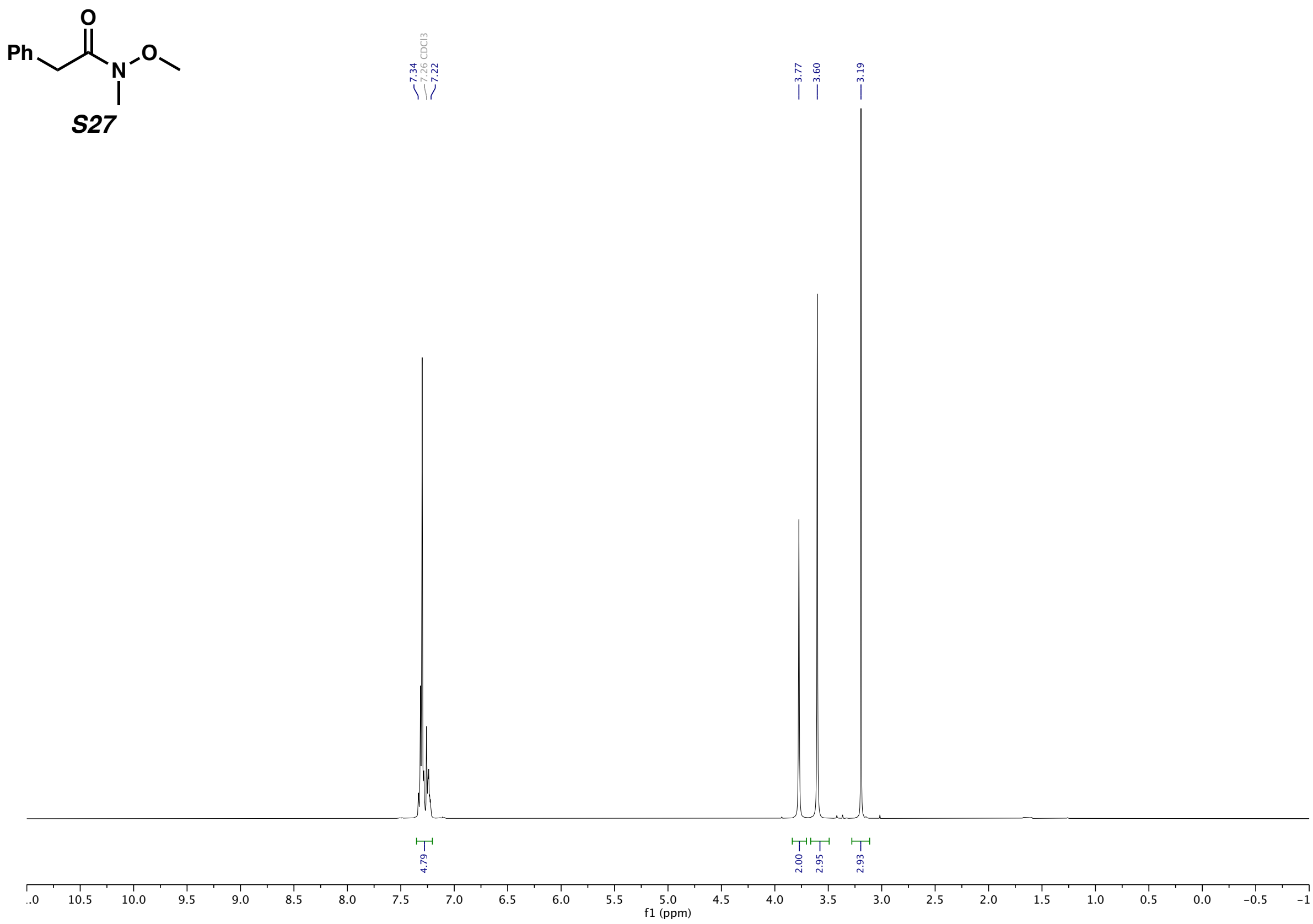

^{1}H NMR (400 MHz, $CDCl_3$) spectrum of compound **S27**.

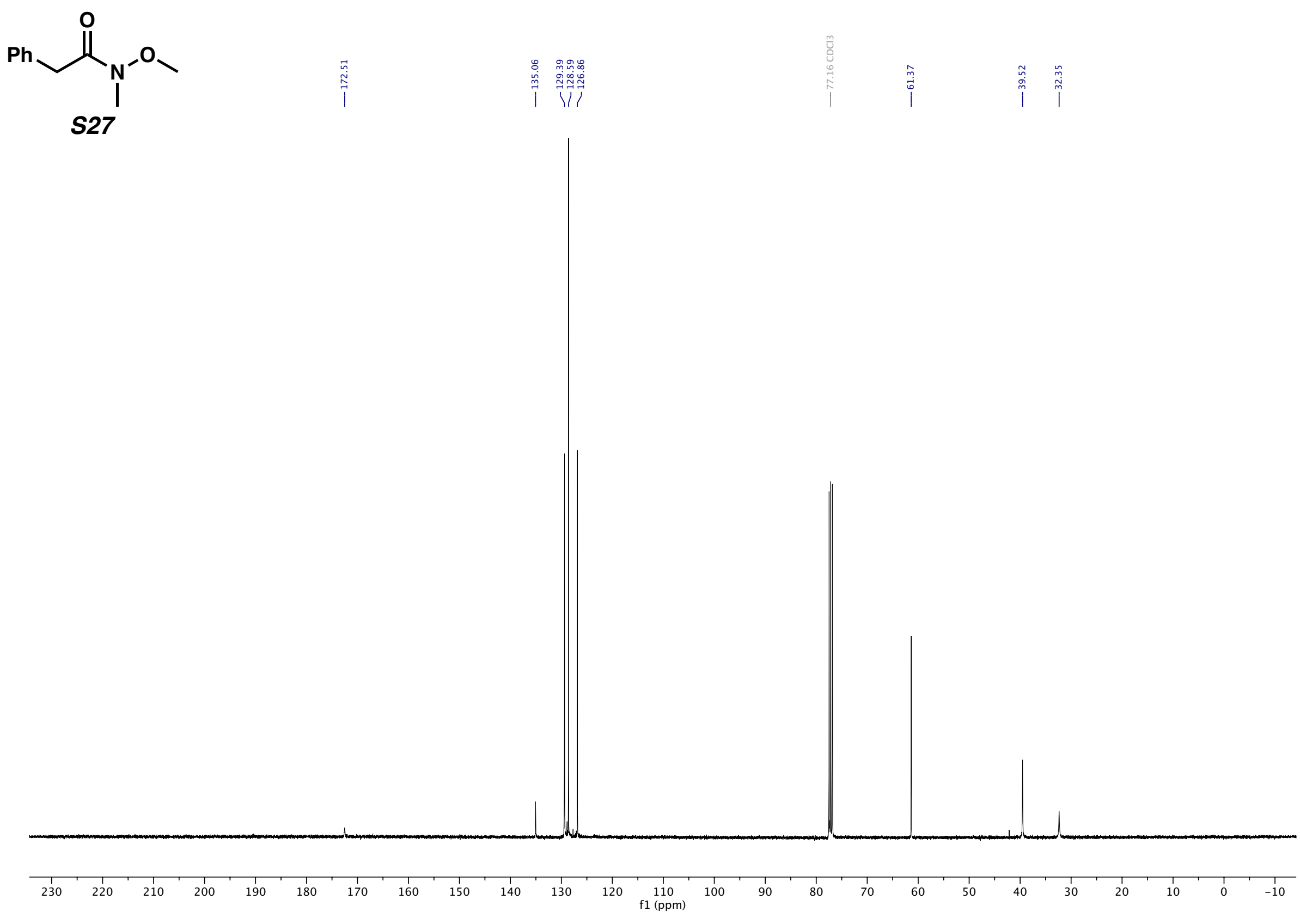

^{13}C NMR (101 MHz, $CDCl_3$) spectrum of compound **S27**.

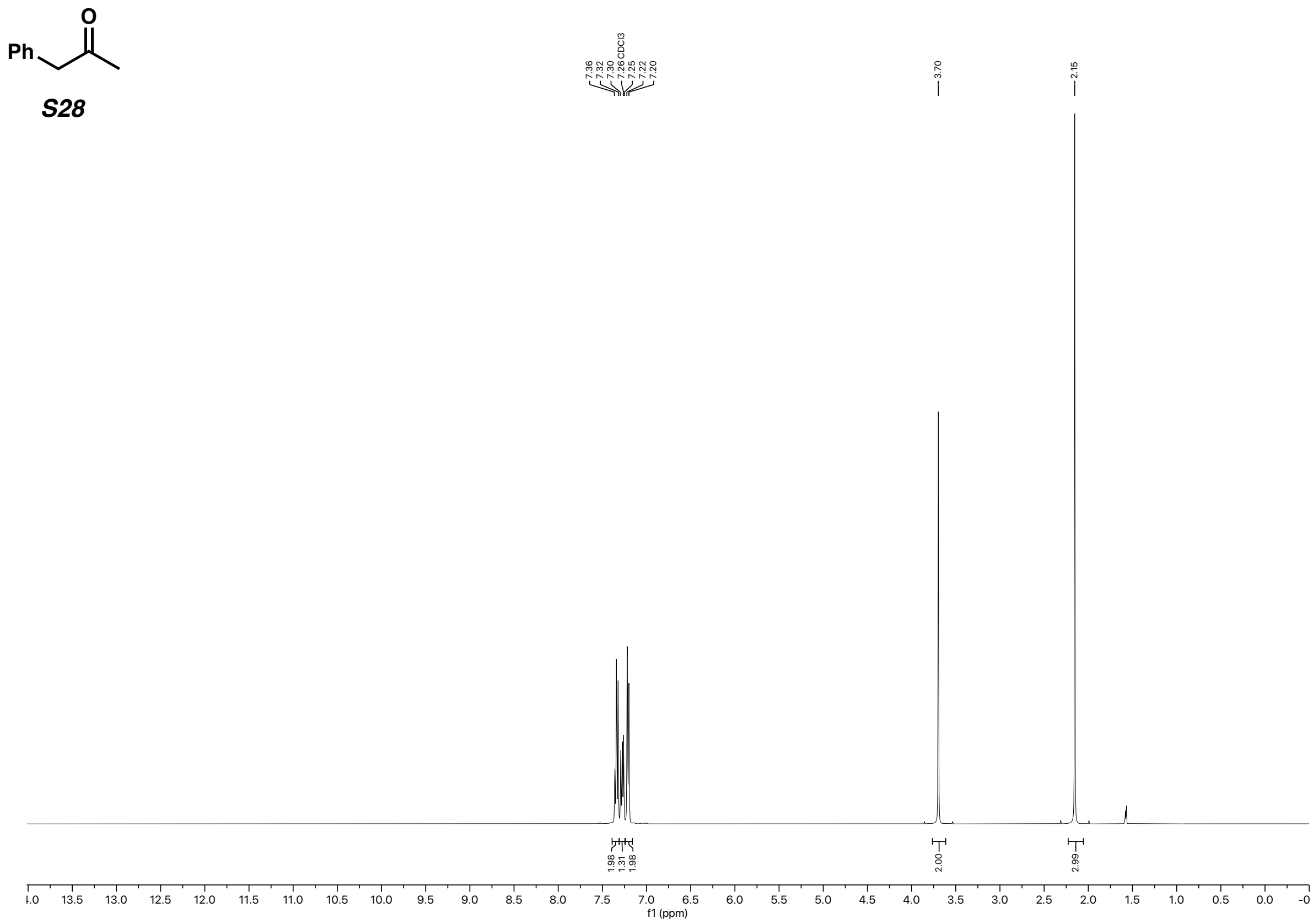

^{1}H NMR (400 MHz, $CDCl_3$) spectrum of compound **S28**.

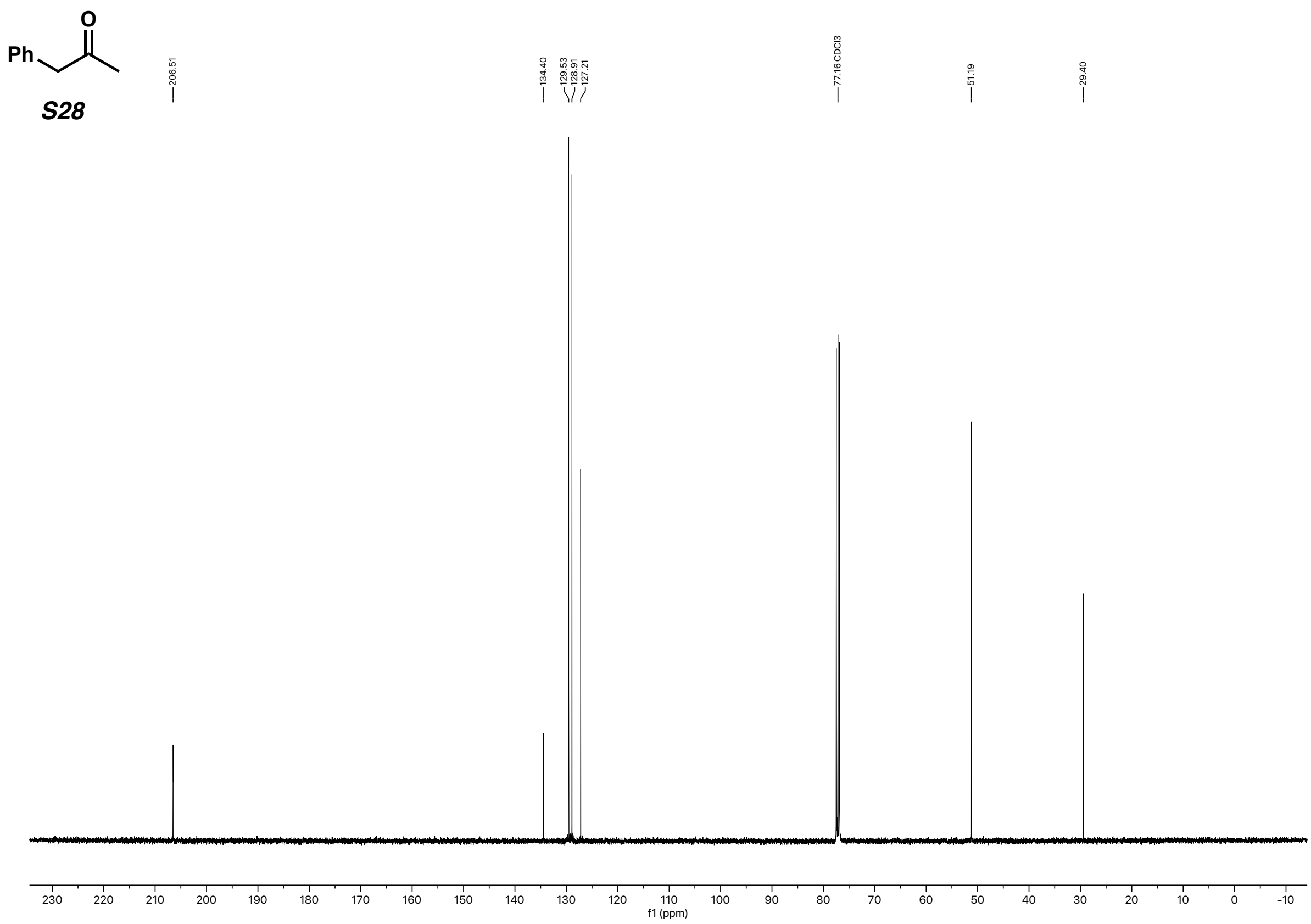

^{13}C NMR (101 MHz, $CDCl_3$) spectrum of compound **S28**.

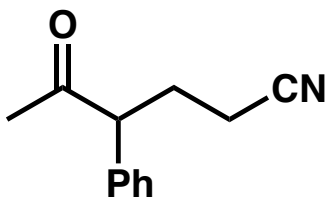

S29

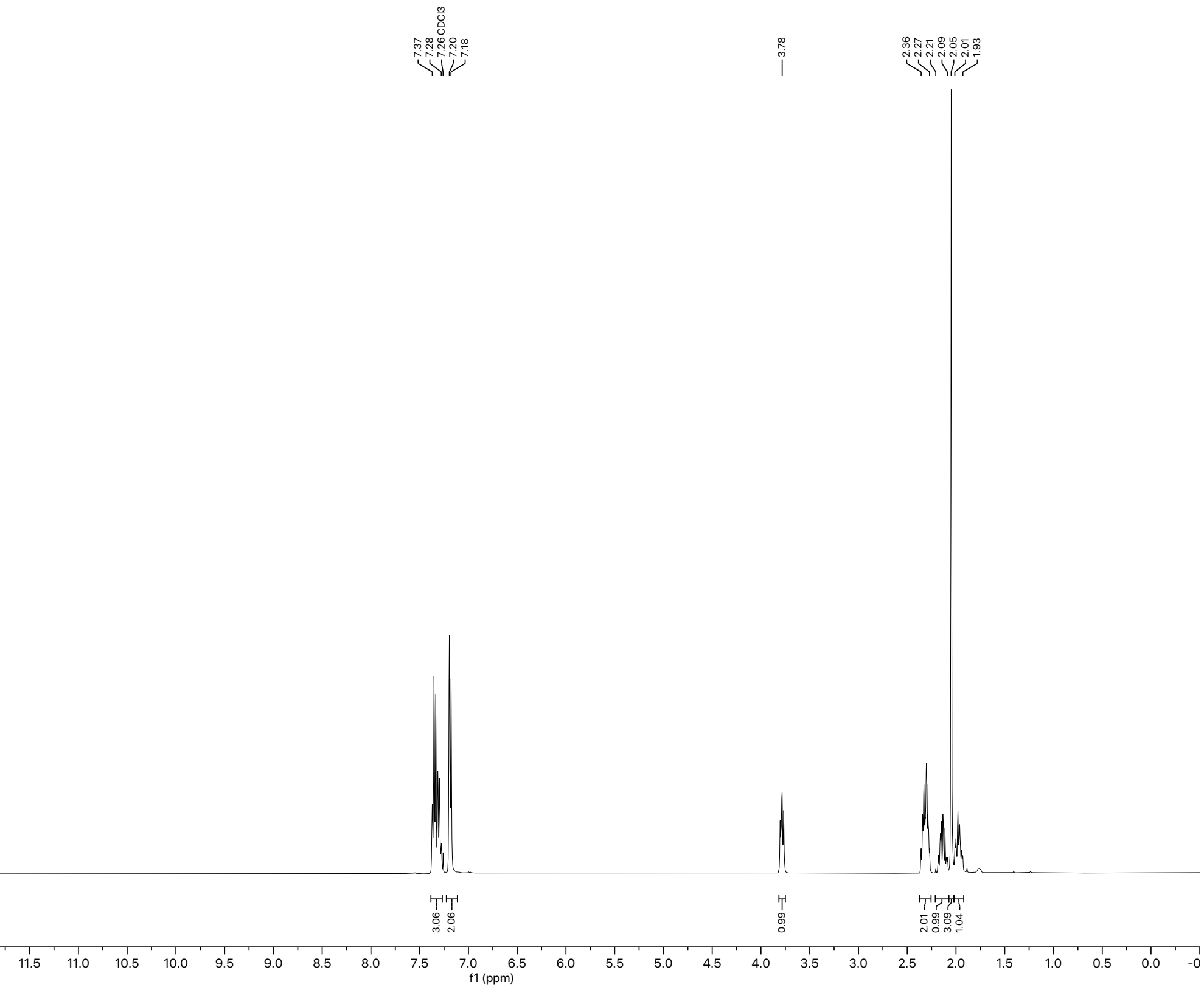

^{1}H NMR (400 MHz, $CDCl_3$) spectrum of compound **S29**.

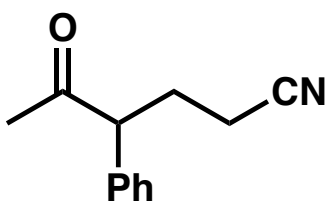

S29

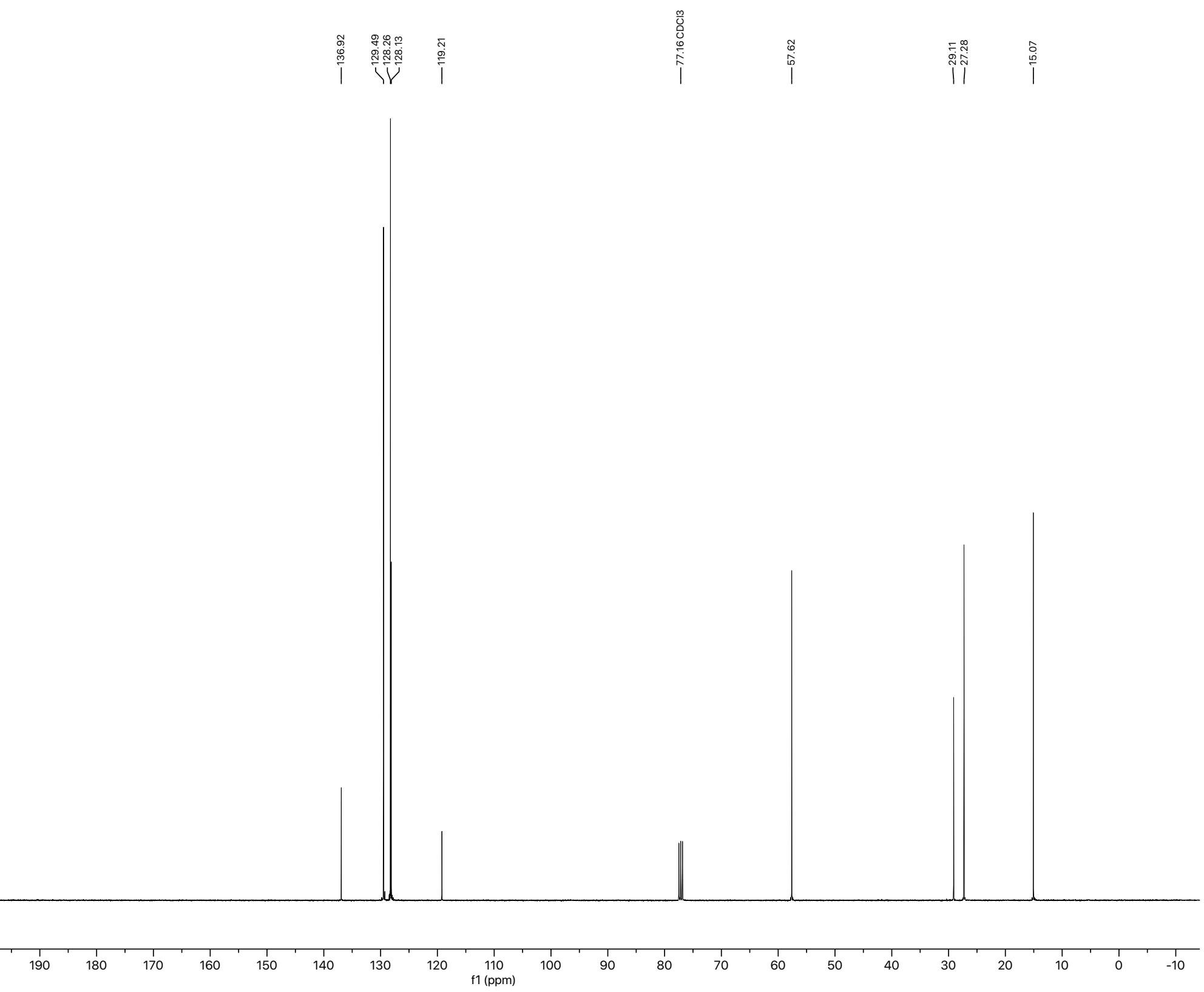

^{13}C NMR (101 MHz, $CDCl_3$) spectrum of compound **S29**.

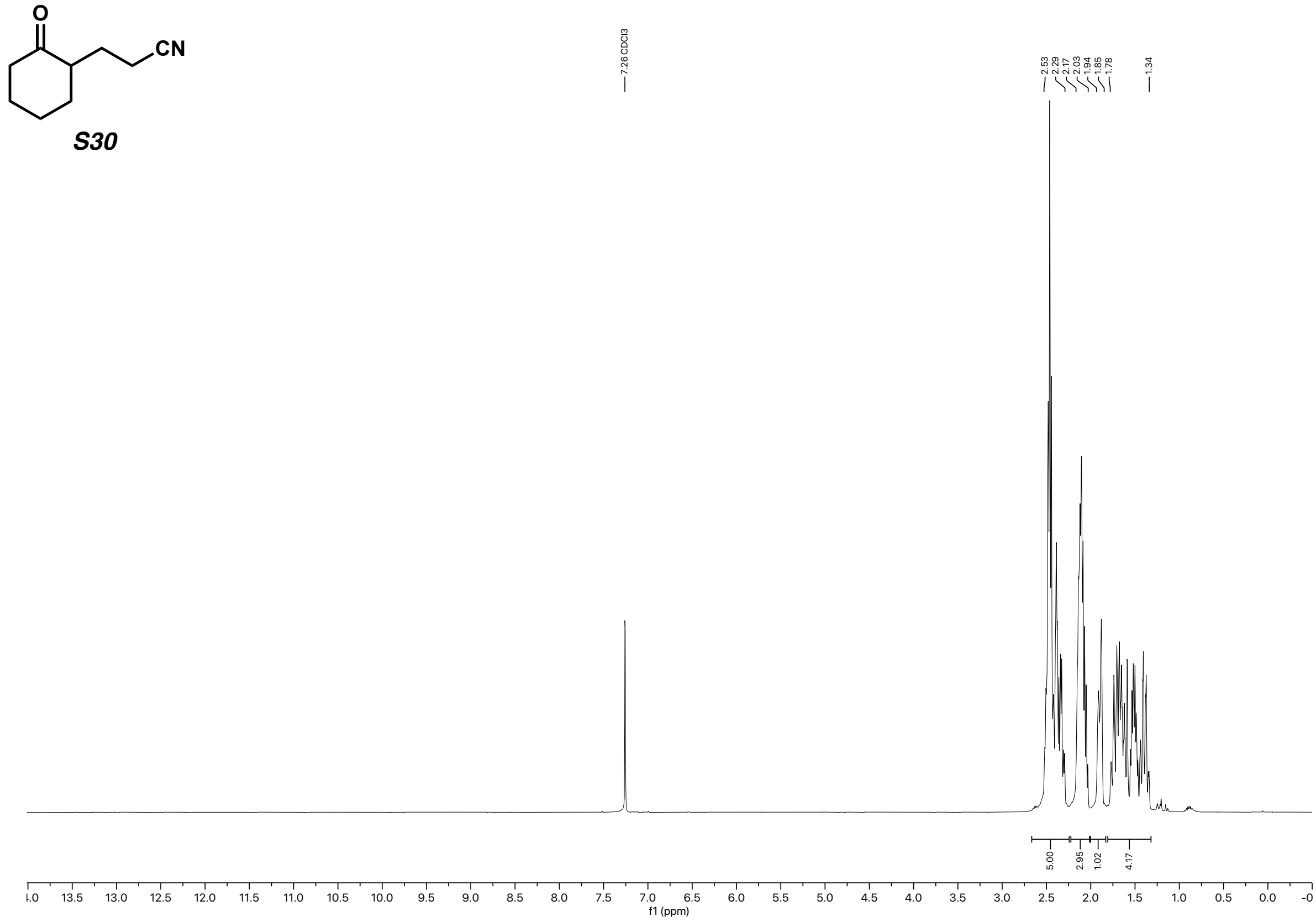

^{1}H NMR (400 MHz, $CDCl_3$) spectrum of compound **S30**.

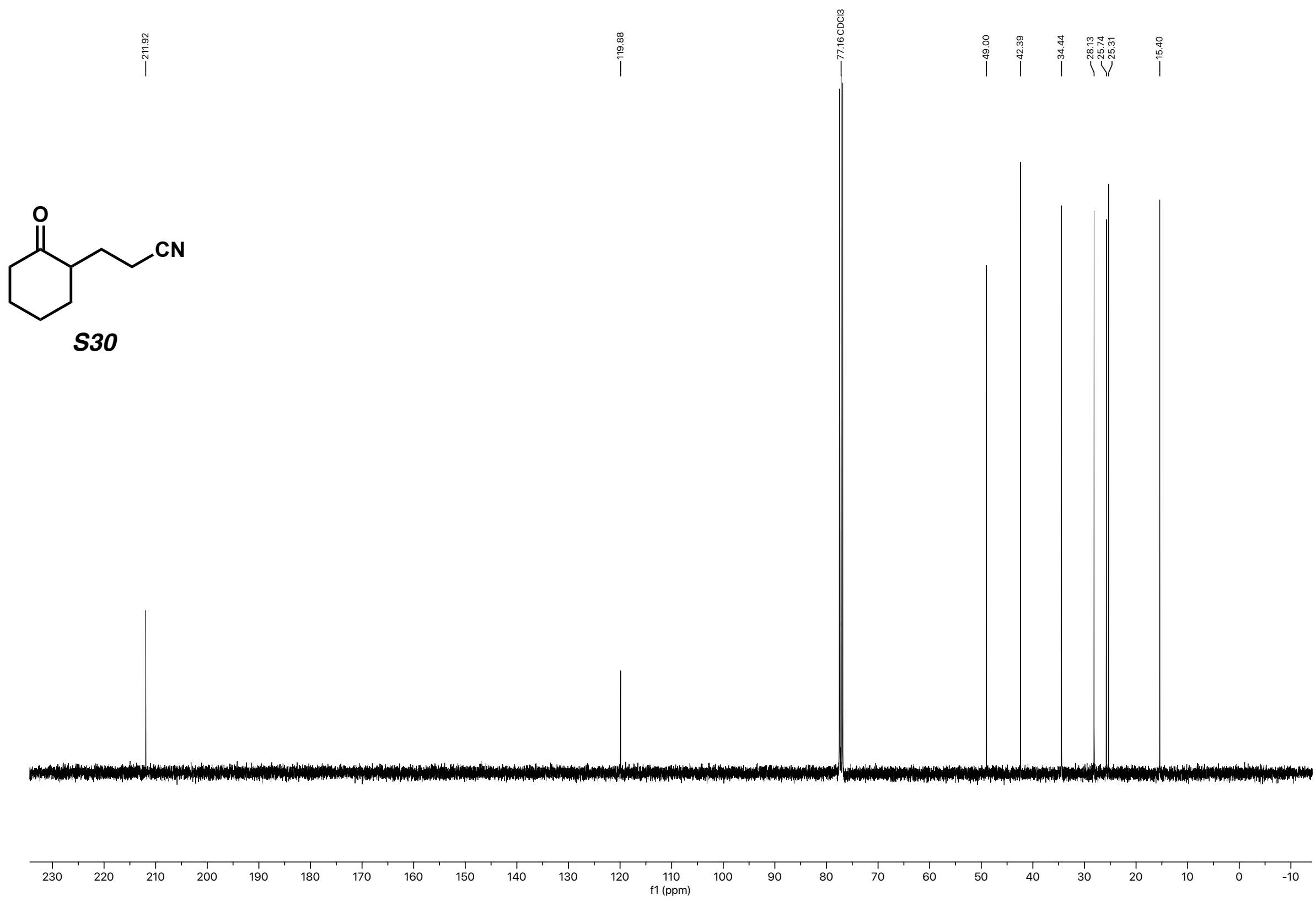

^{13}C NMR (101 MHz, $CDCl_3$) spectrum of compound **S30**.

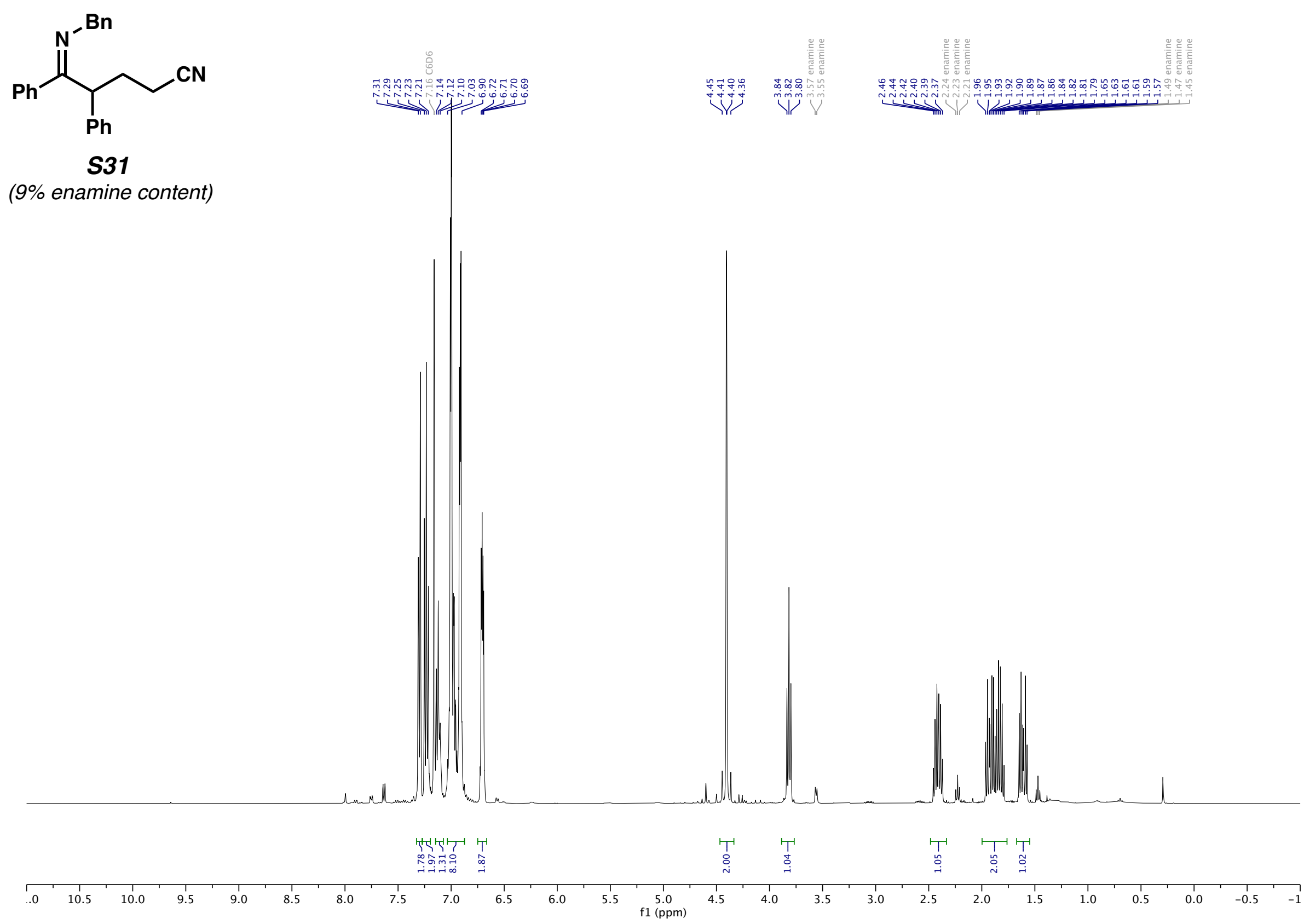

^{1}H NMR (400 MHz, C_6D_6) spectrum of compound **S31**.

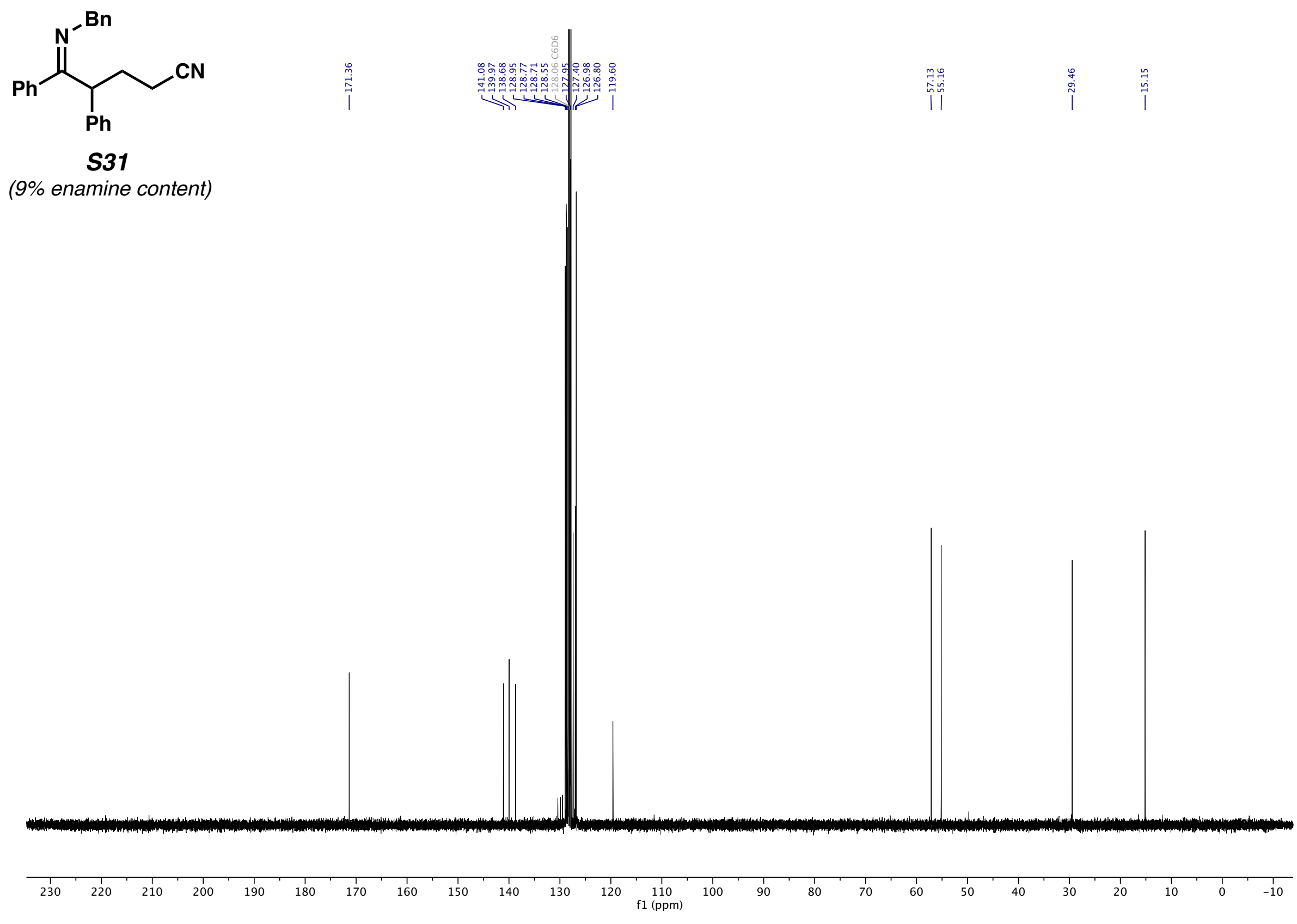

^{13}C NMR (101 MHz, C_6D_6) spectrum of compound **S31**.

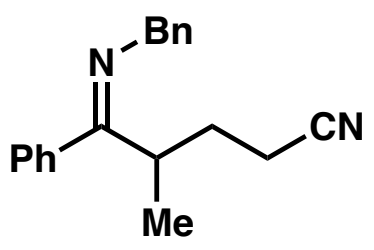

S32

(5% enamine content)

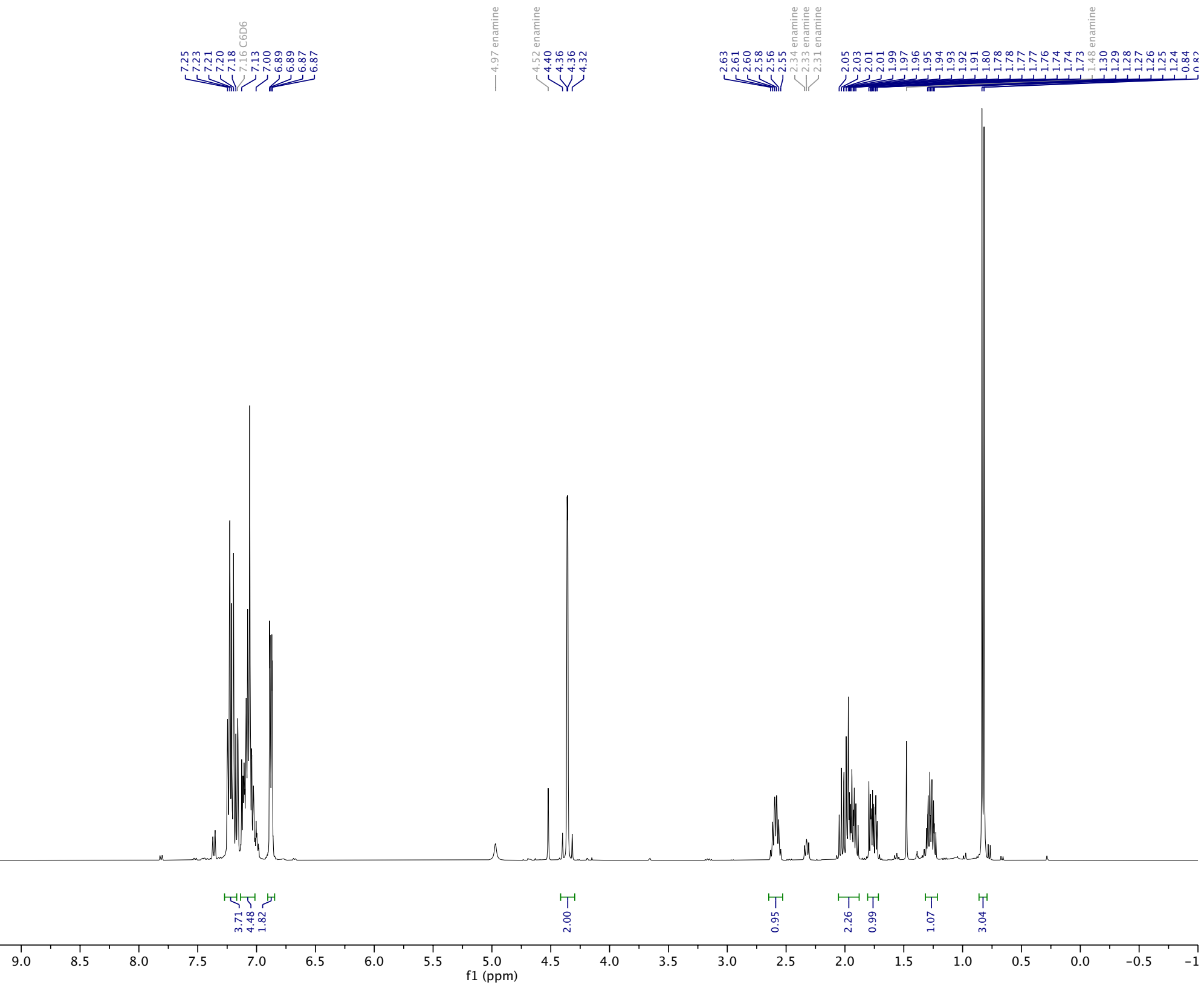

^{1}H NMR (400 MHz, C_6D_6) spectrum of compound **S32**.

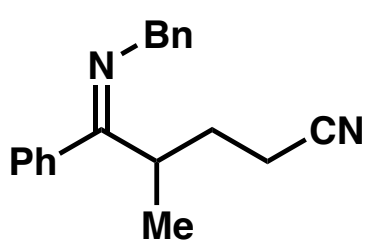

S32

(5% enamine content)

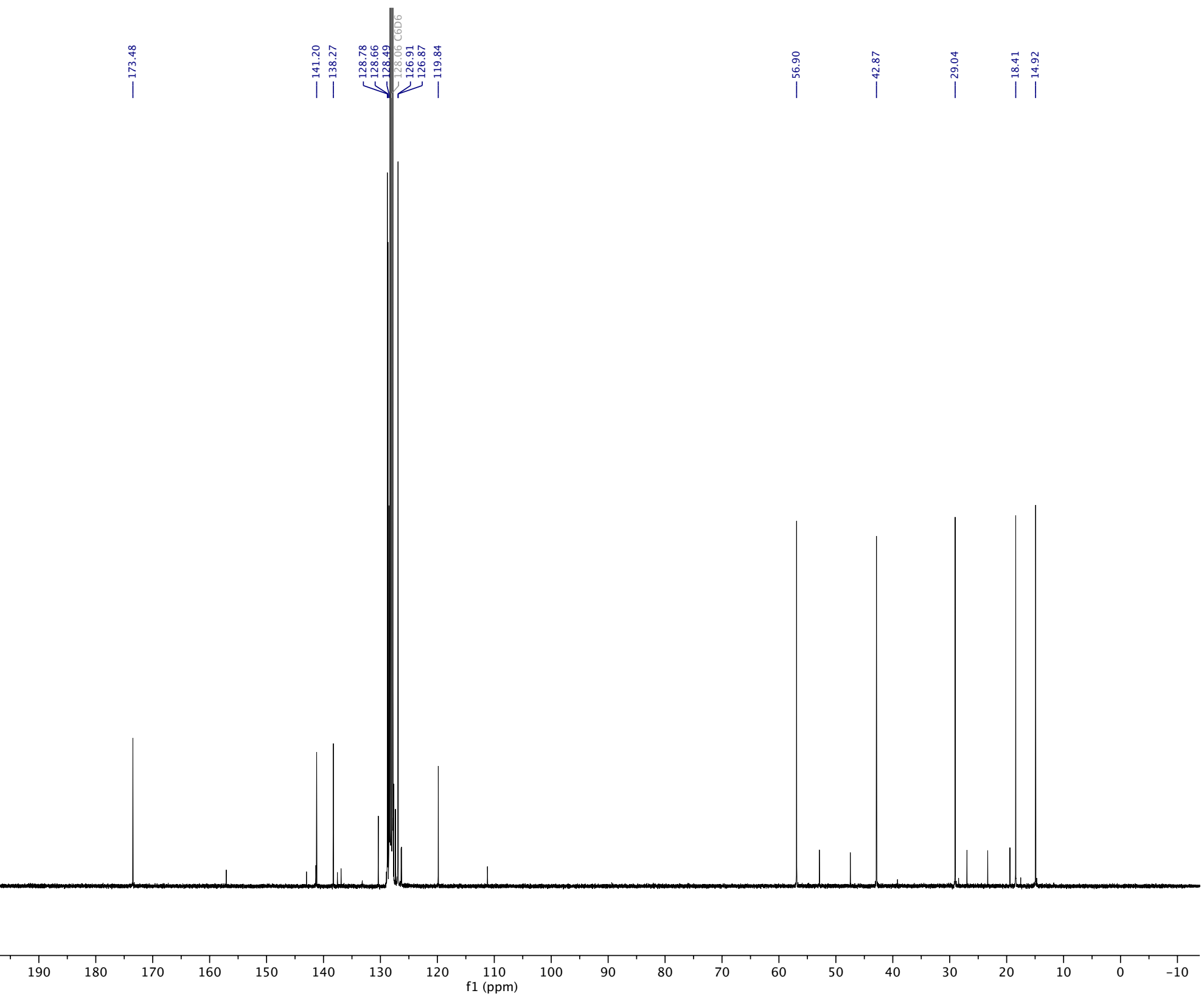

^{13}C NMR (101 MHz, C_6D_6) spectrum of compound **S32**.

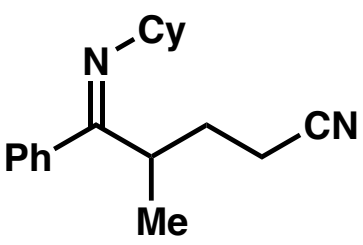

29

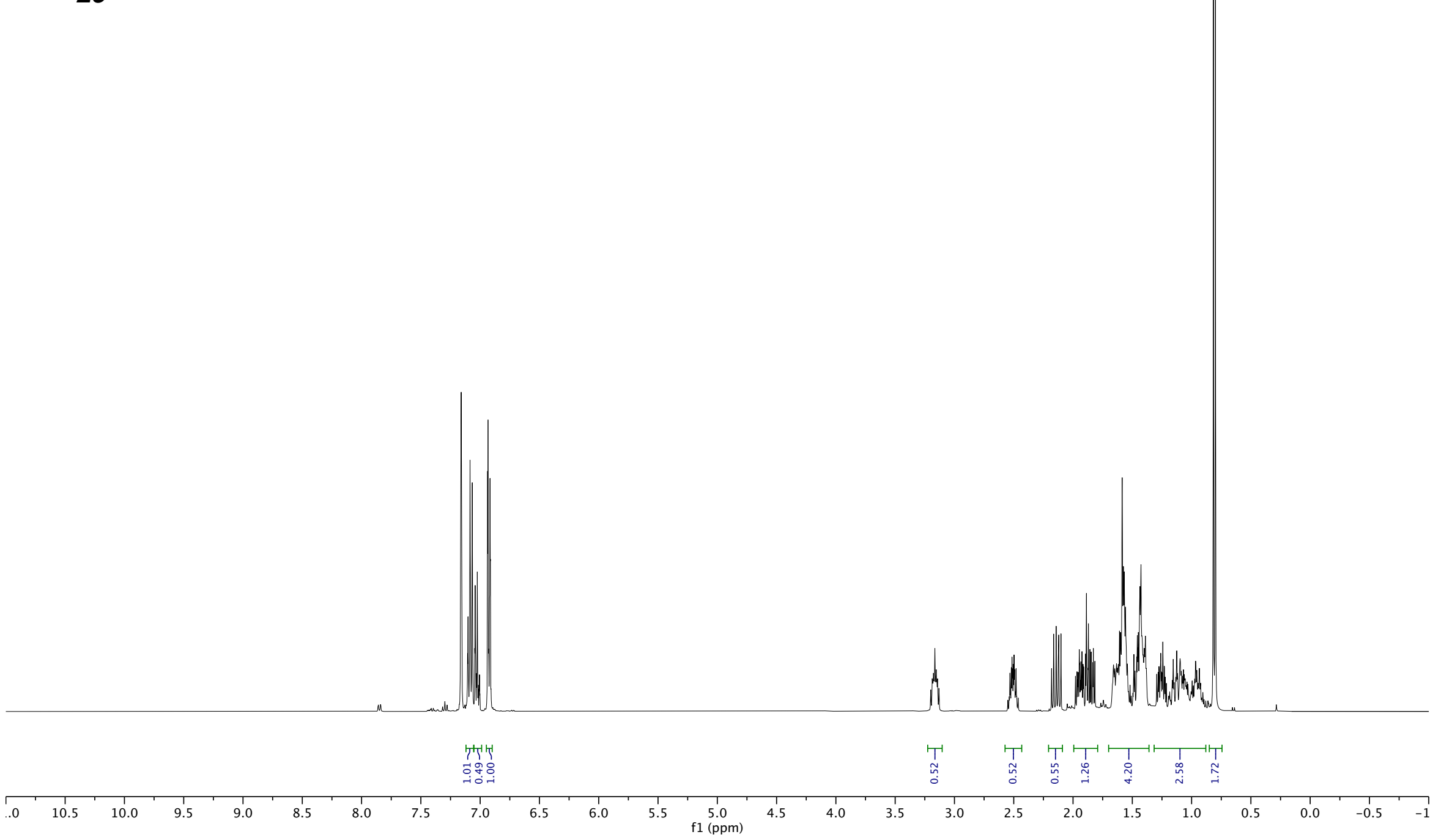

^{1}H NMR (400 MHz, C_6D_6) spectrum of compound **29**.

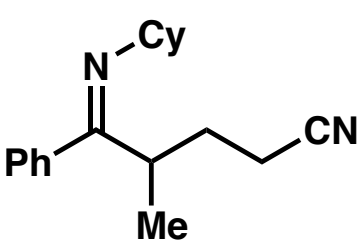

29

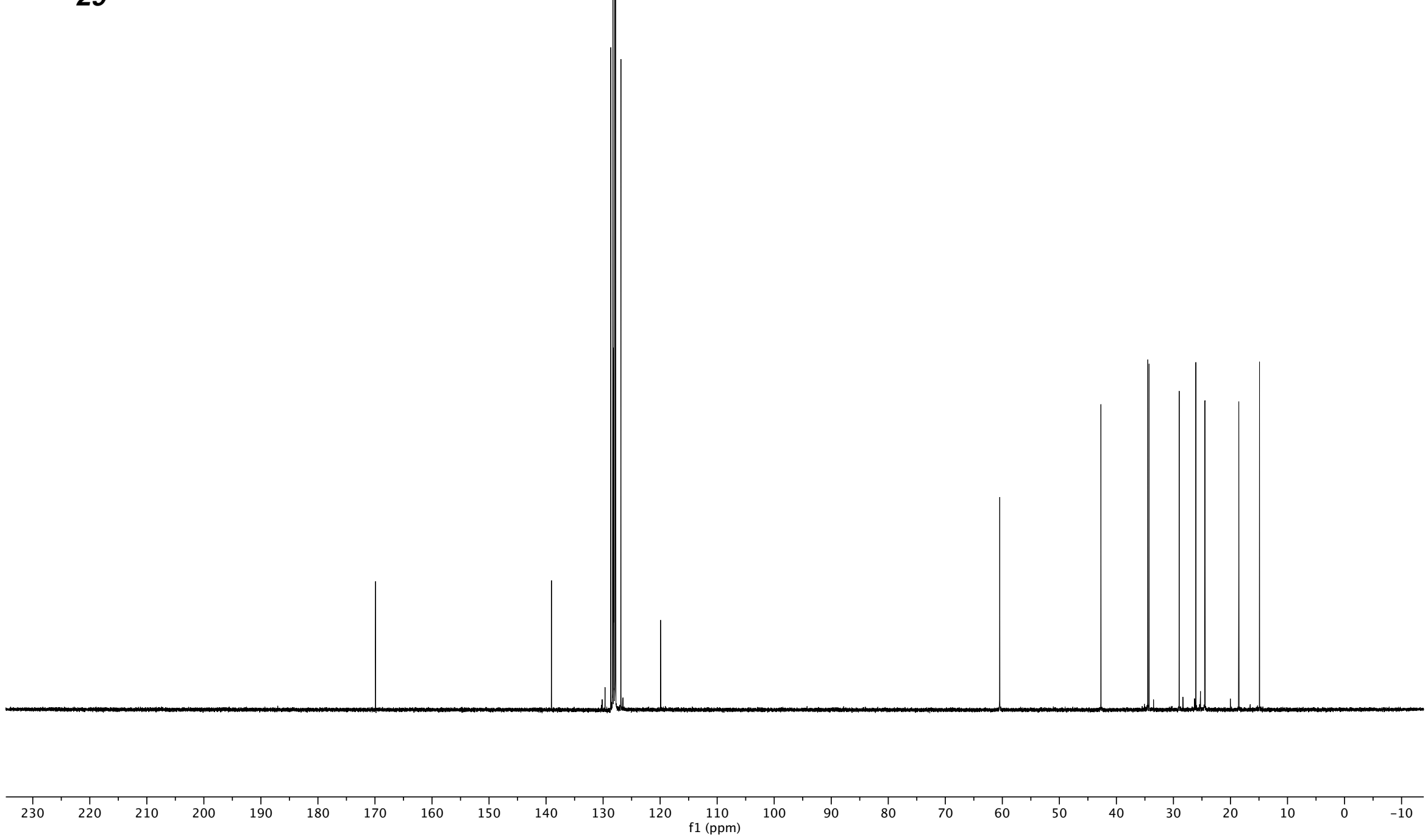

^{13}C NMR (101 MHz, C_6D_6) spectrum of compound **29**.

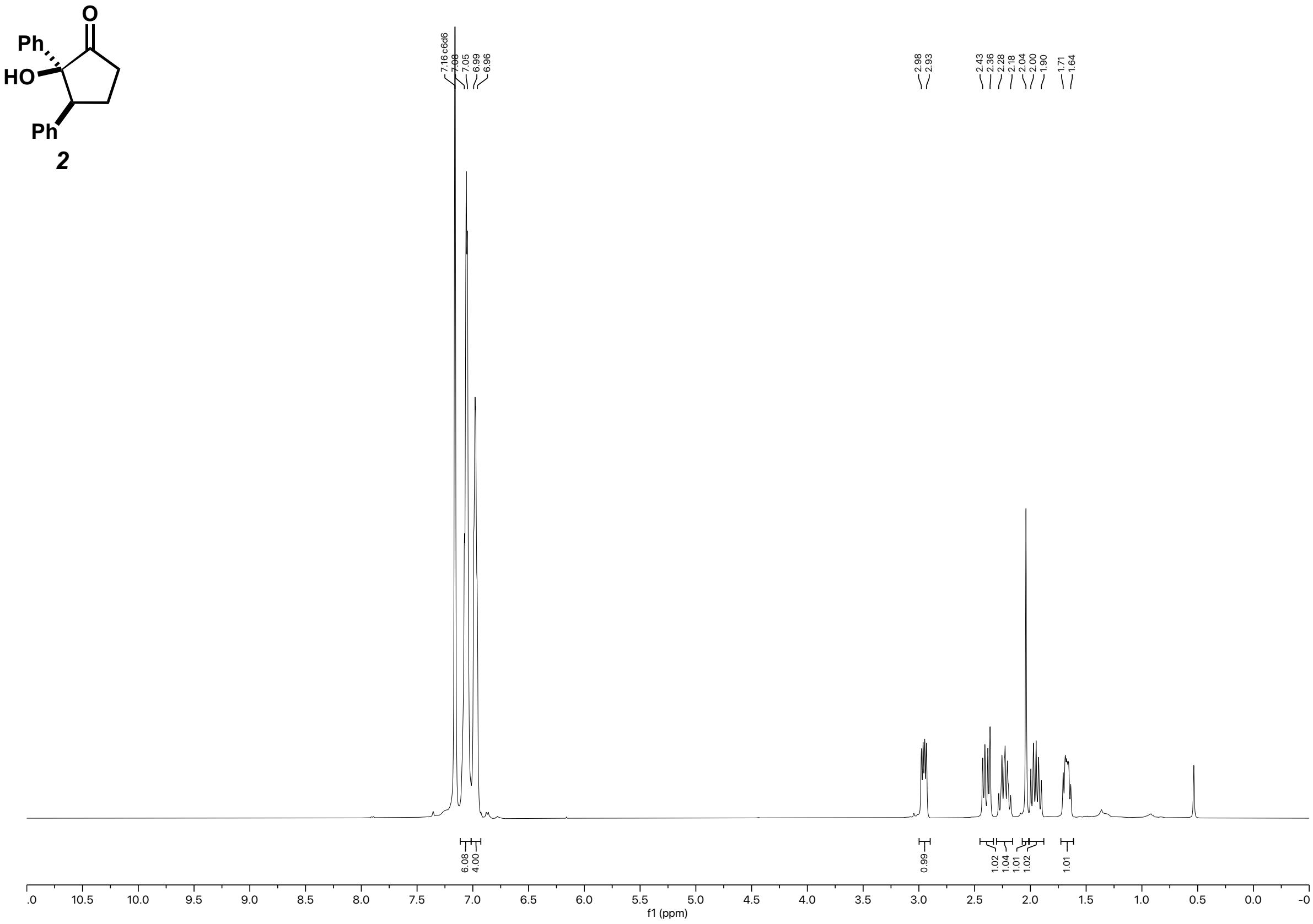

^{1}H NMR (400 MHz, C_6D_6) spectrum of compound **2**.

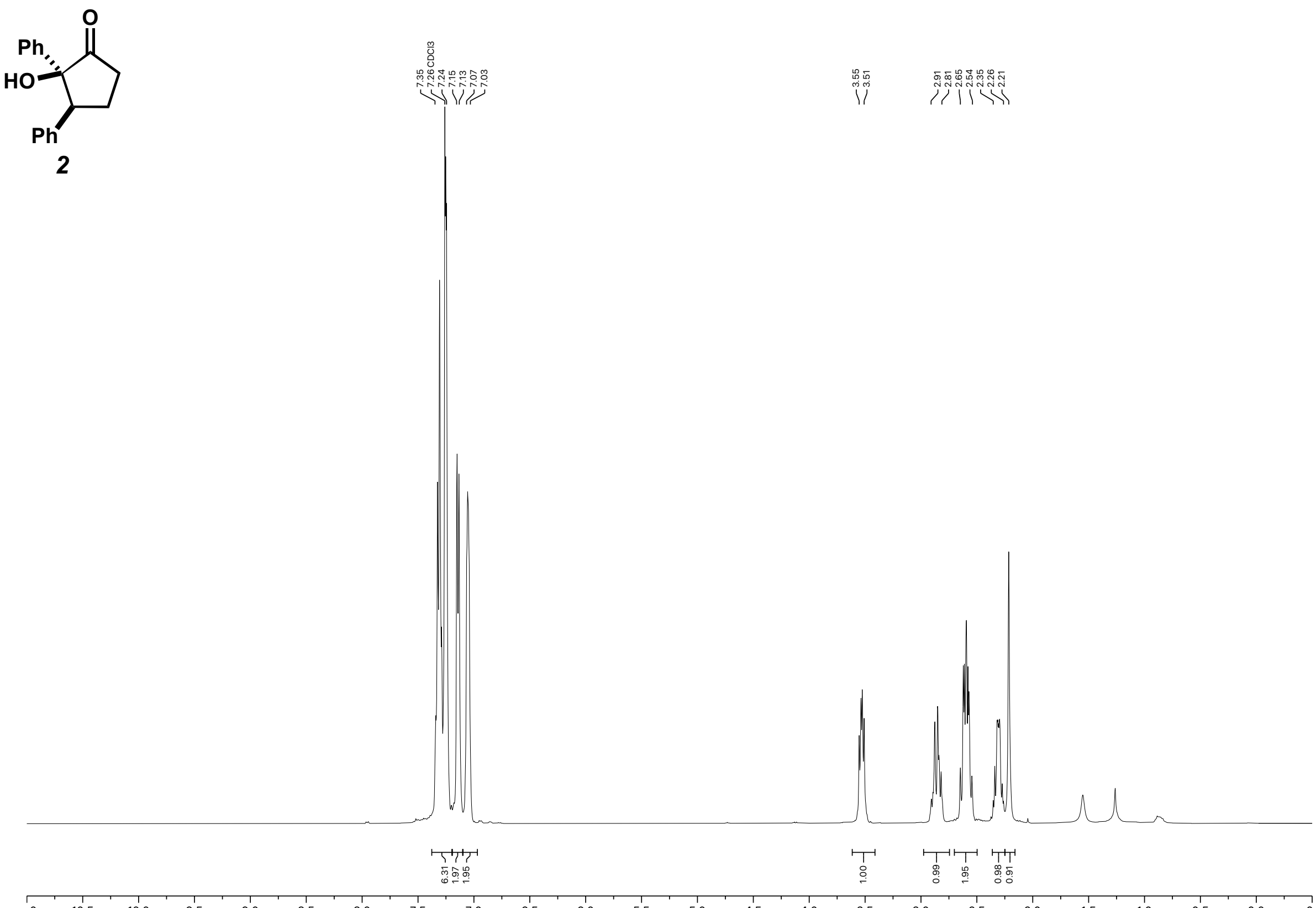

^{1}H NMR (400 MHz, $CDCl_3$) spectrum of compound **2**.

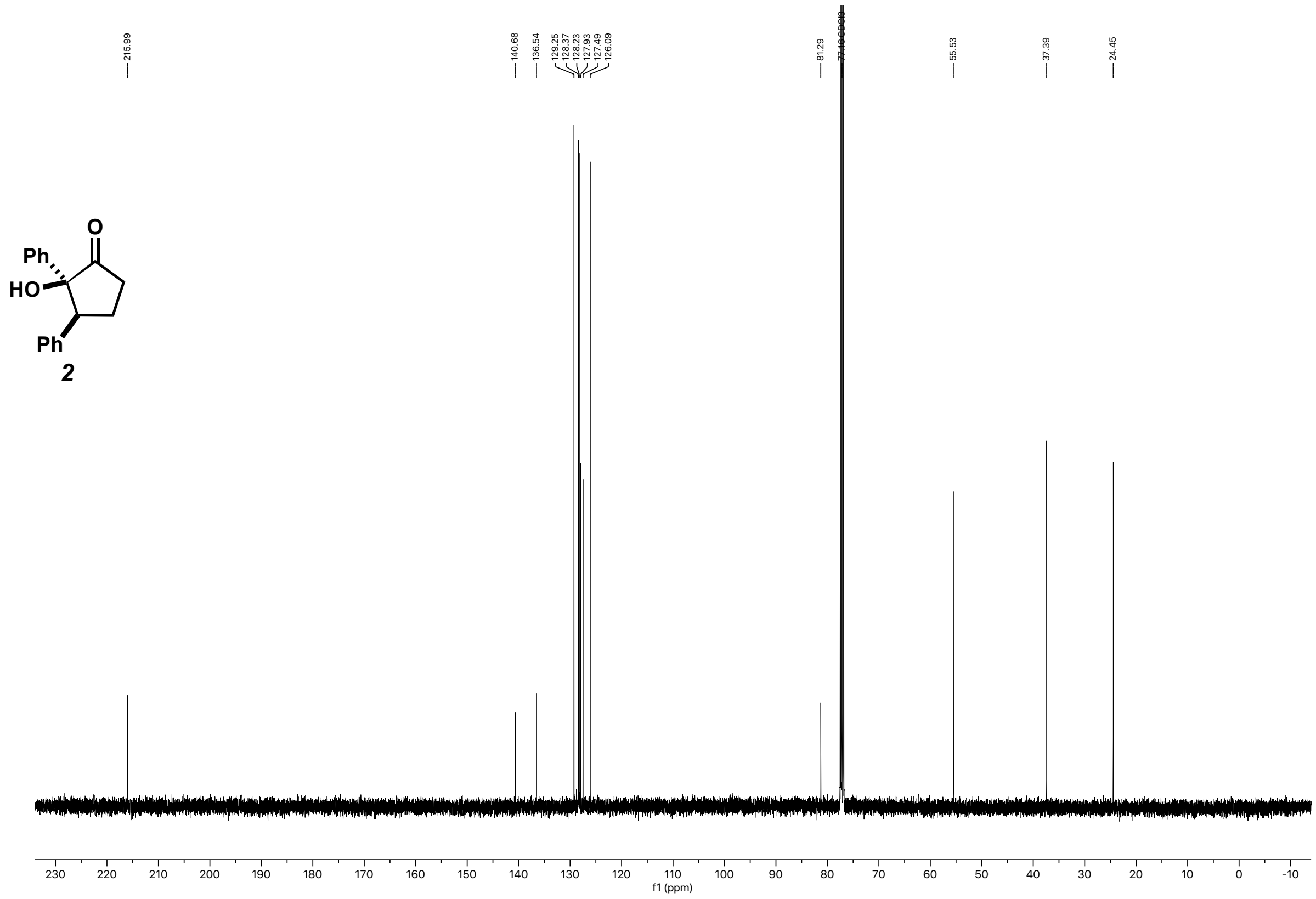

^{13}C NMR (101 MHz, $CDCl_3$) spectrum of compound **2**.

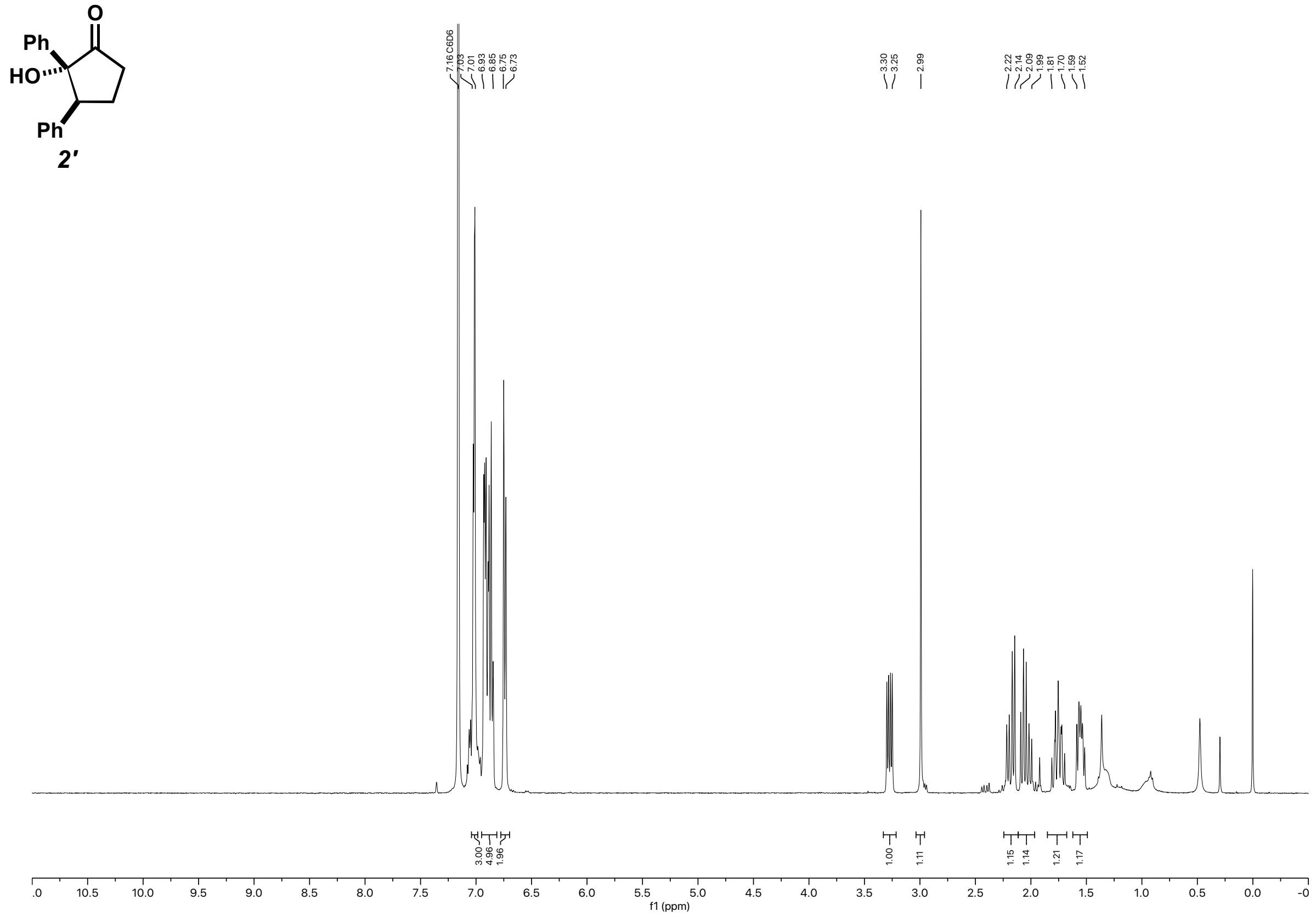

¹H NMR (400 MHz, C_6D_6) spectrum of compound **2'**.

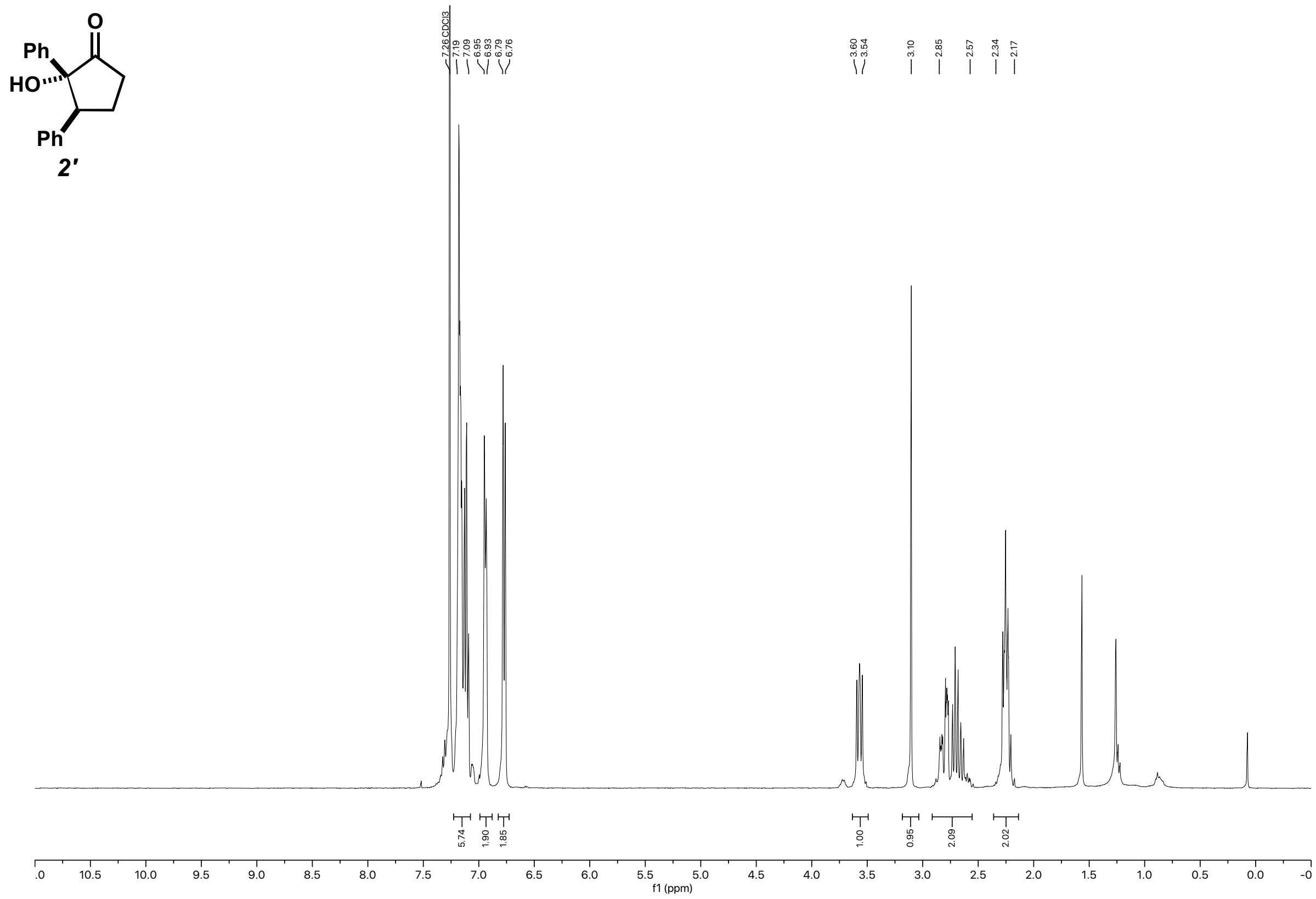

¹H NMR (400 MHz, $CDCl_3$) spectrum of compound **2'**.

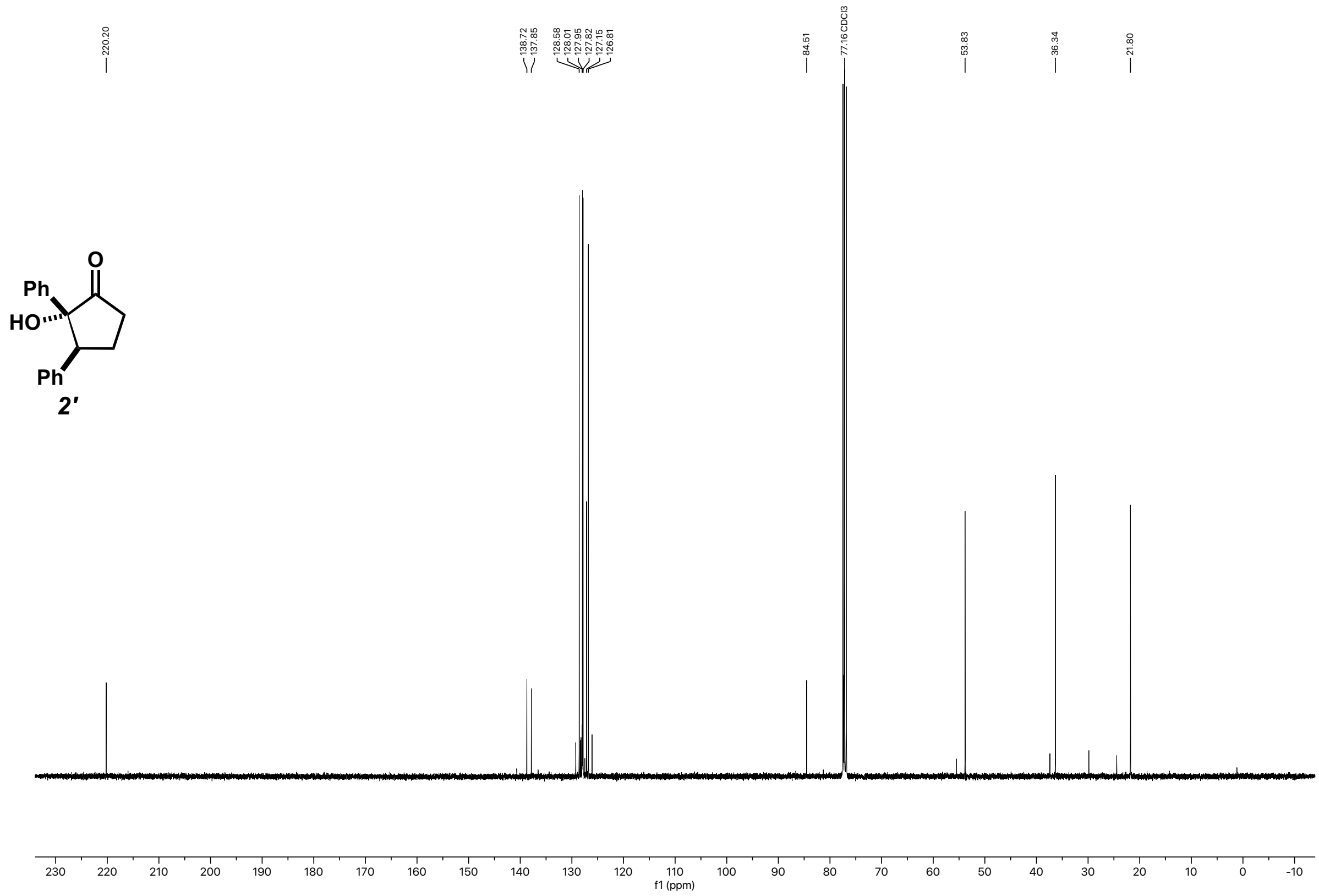

^{13}C NMR (101 MHz, $CDCl_3$) spectrum of compound **2'**.

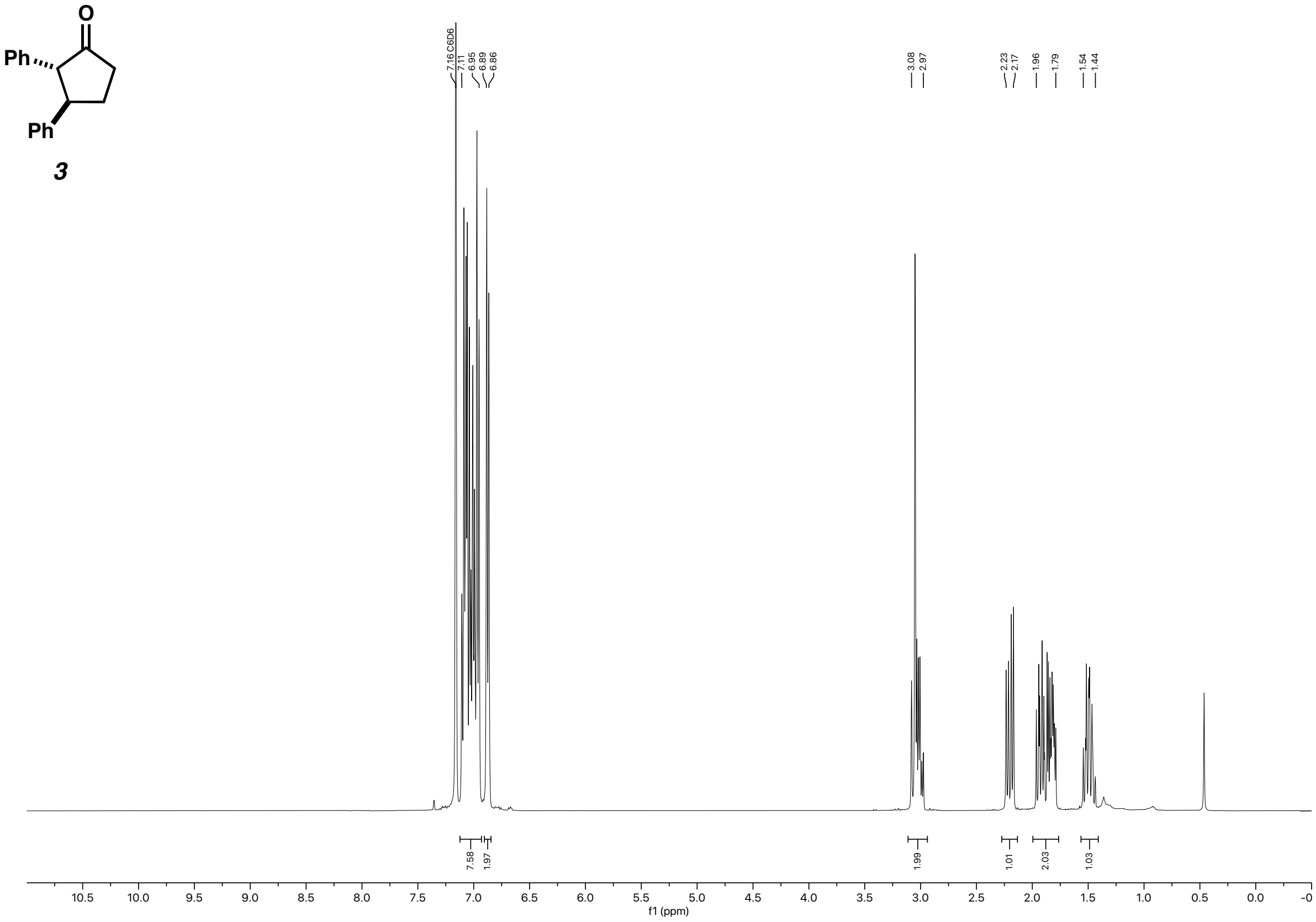

^{1}H NMR (400 MHz, C_6D_6) spectrum of compound **3**.

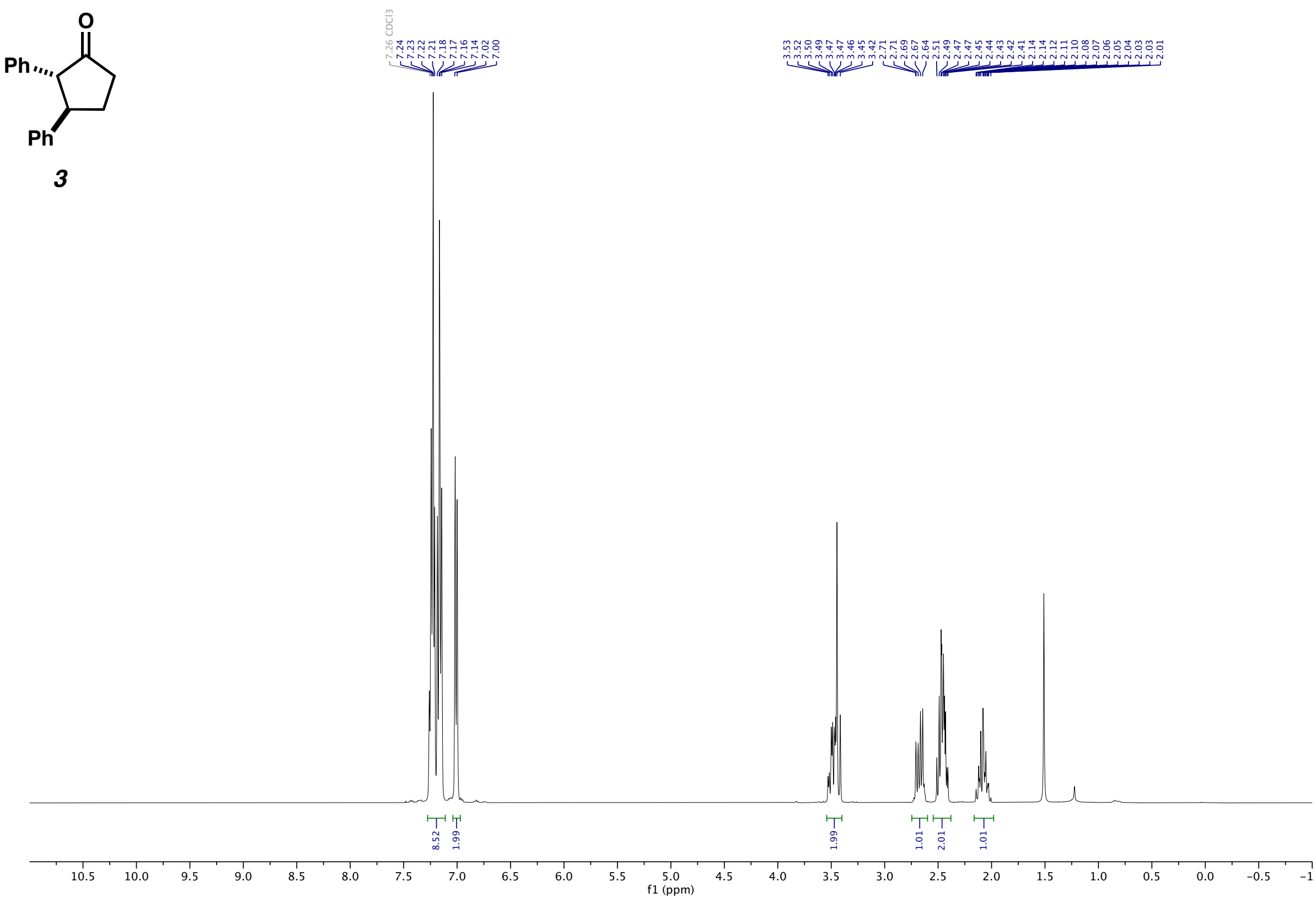

^{1}H NMR (400 MHz, $CDCl_3$) spectrum of compound **3**.

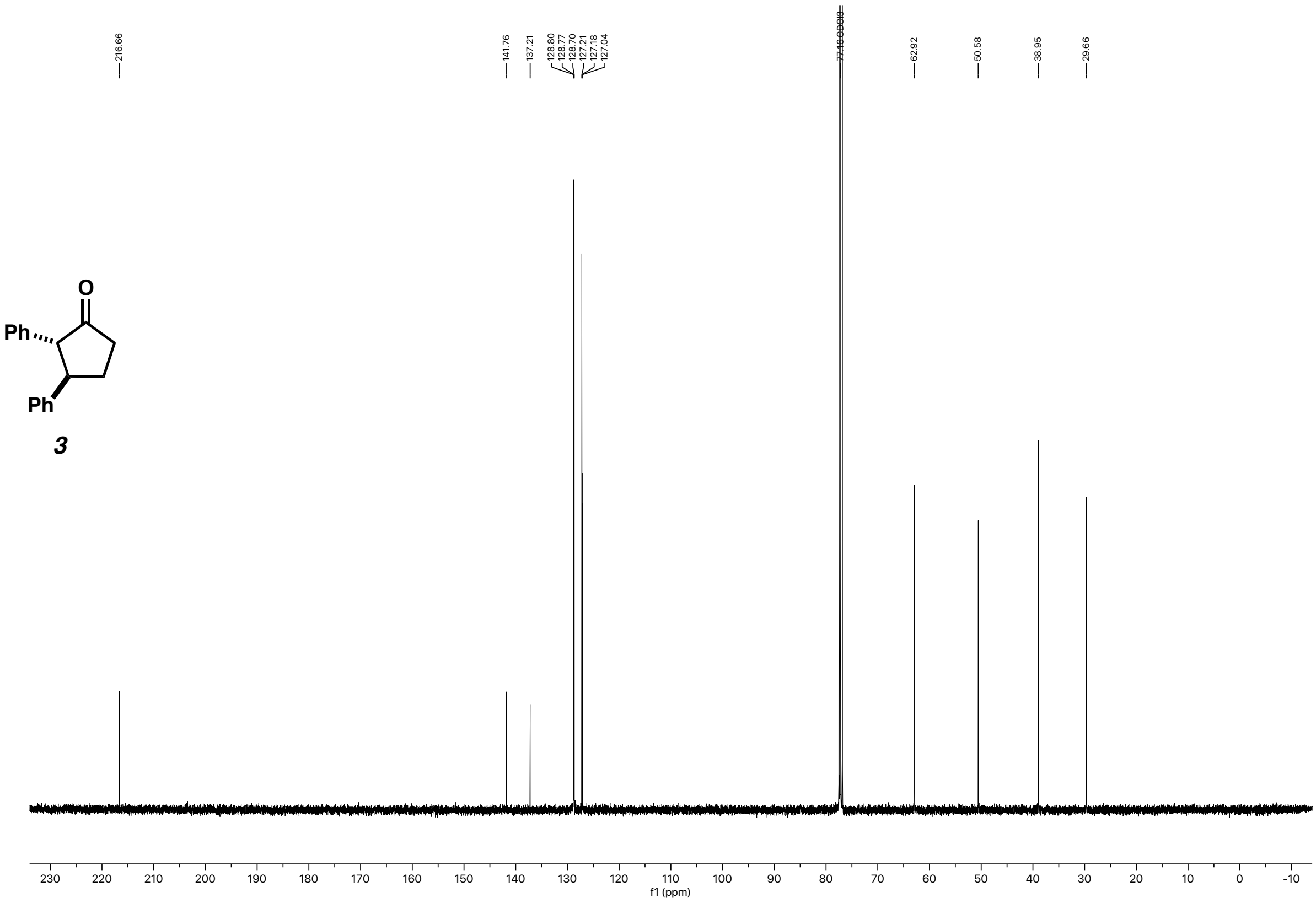

^{13}C NMR (101 MHz, $CDCl_3$) spectrum of compound **3**.

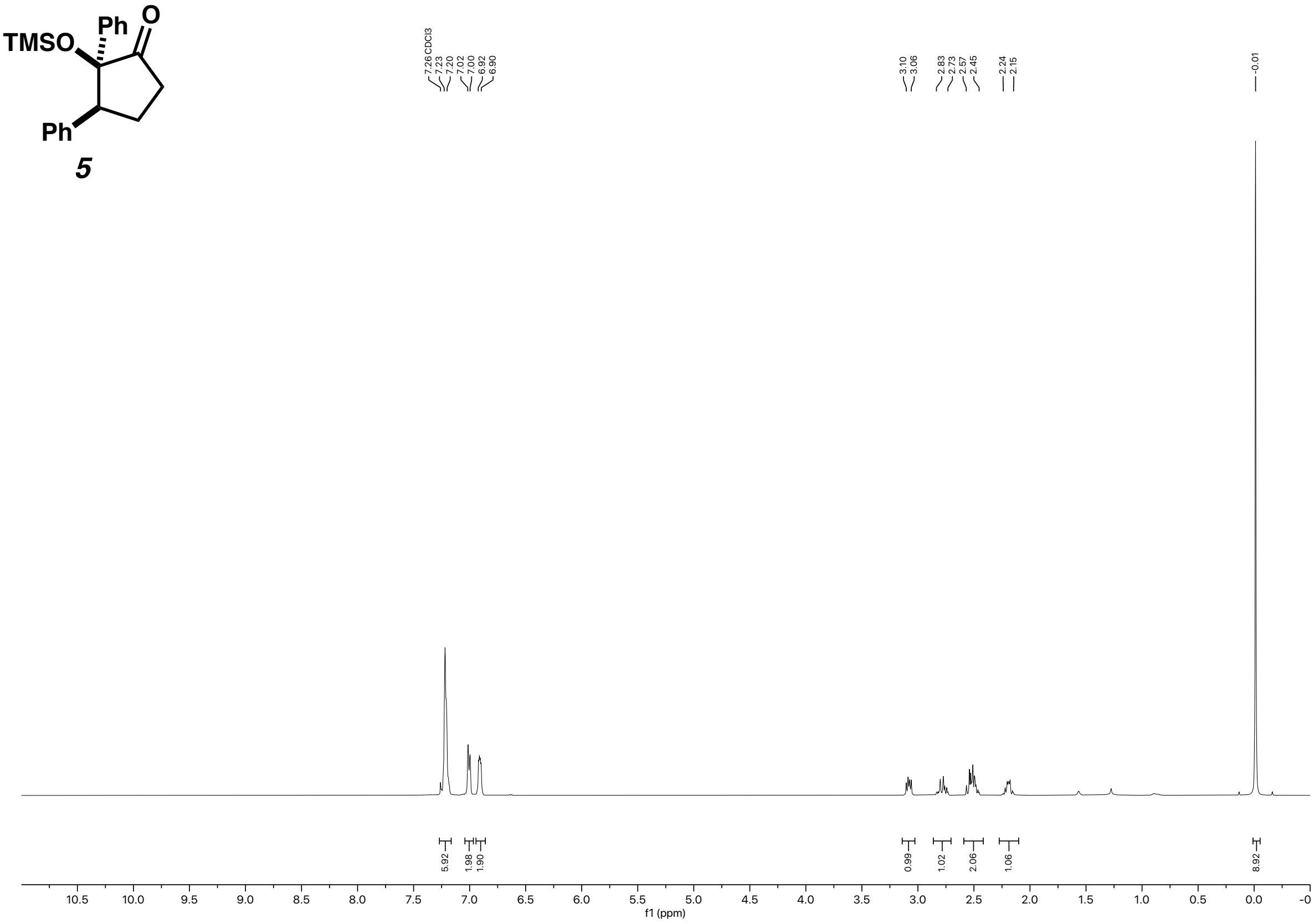

^{1}H NMR (400 MHz, $CDCl_3$) spectrum of compound **5**.

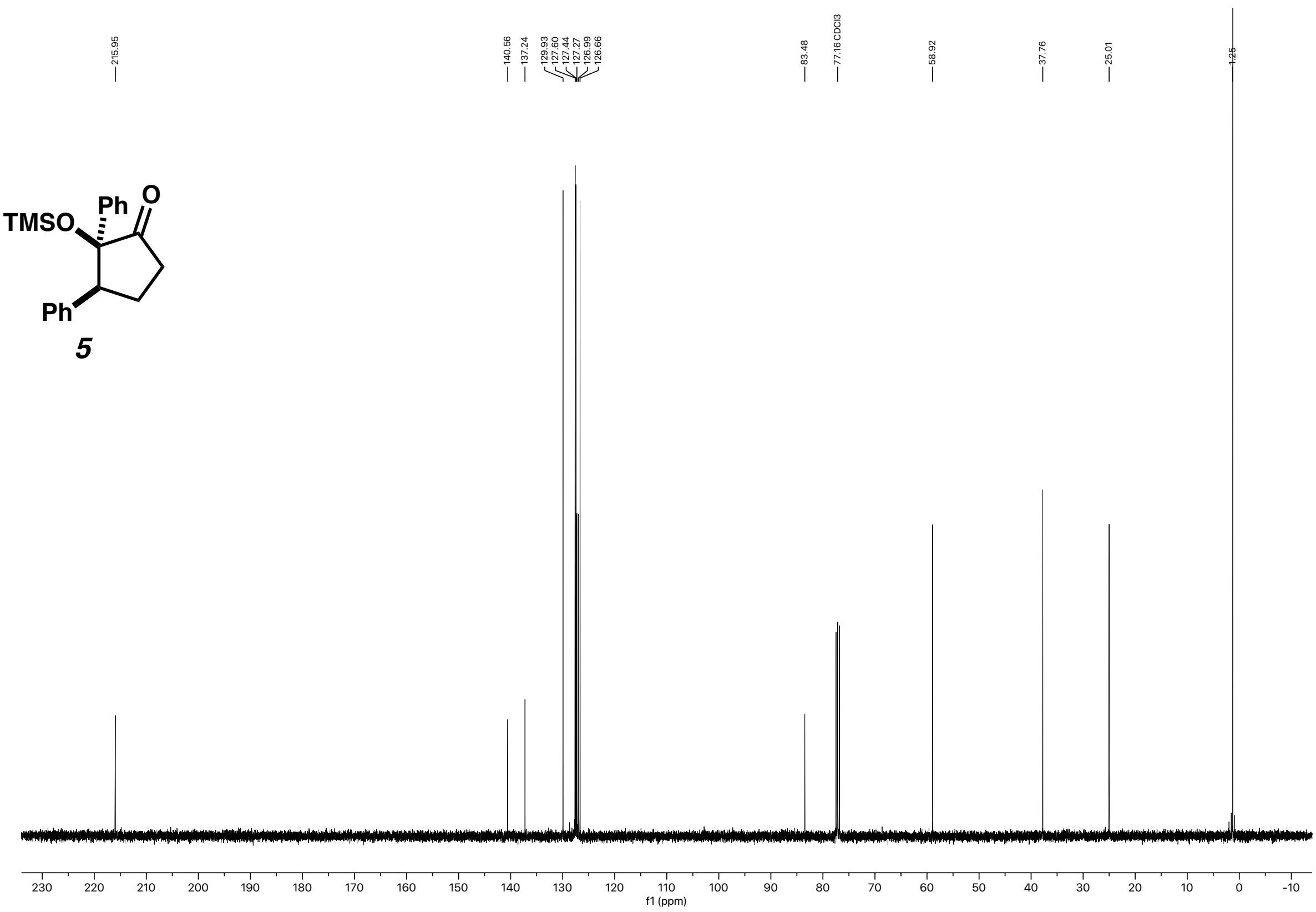

^{13}C NMR (101 MHz, $CDCl_3$) spectrum of compound **5**.

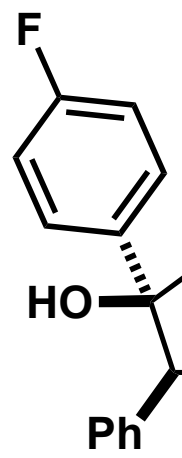

6

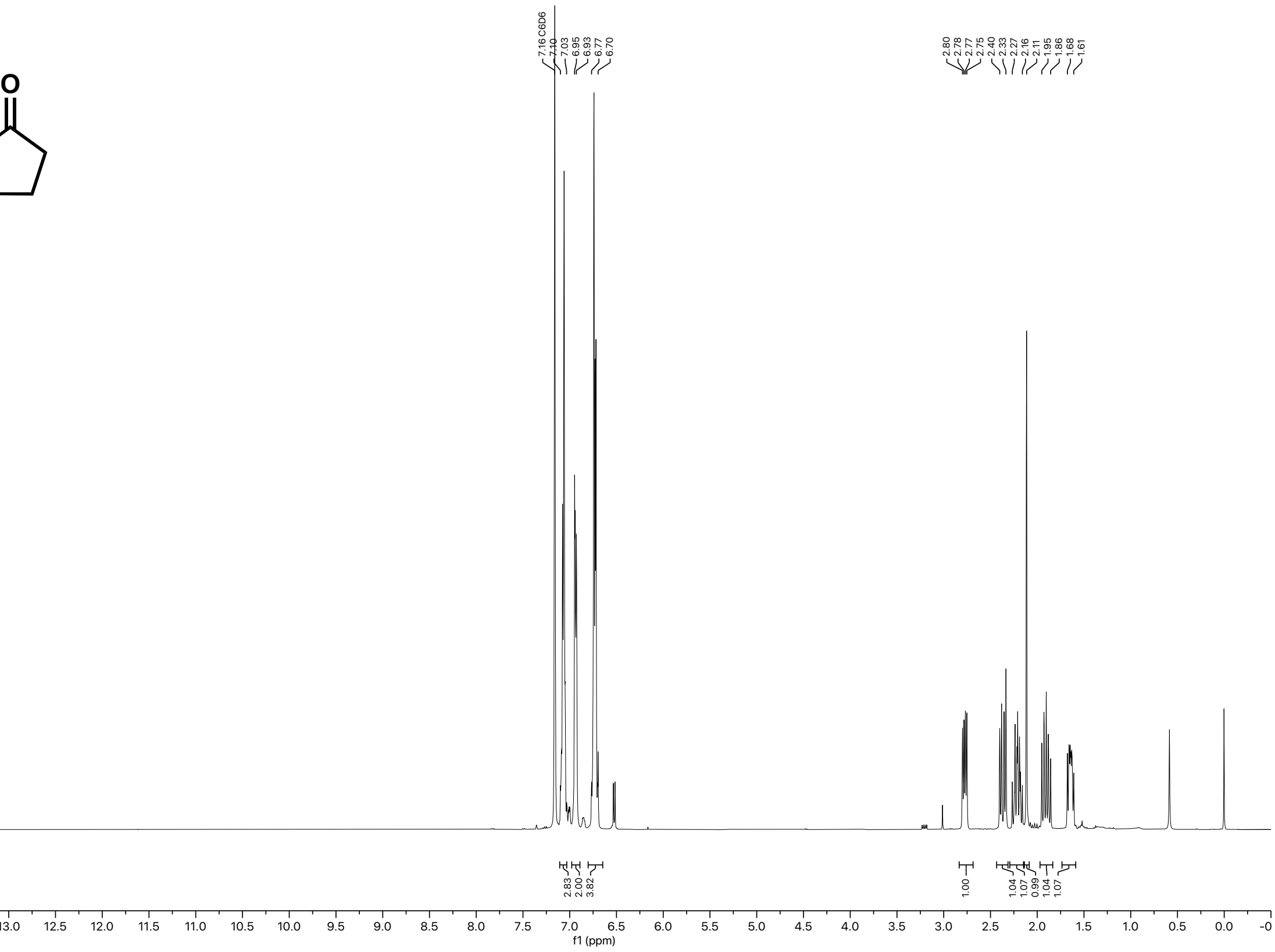

^{1}H NMR (400 MHz, C_6D_6) spectrum of compound **6**.

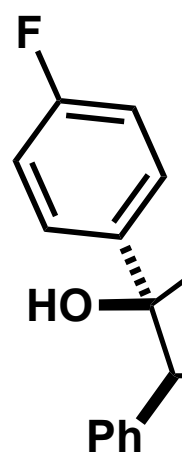

6

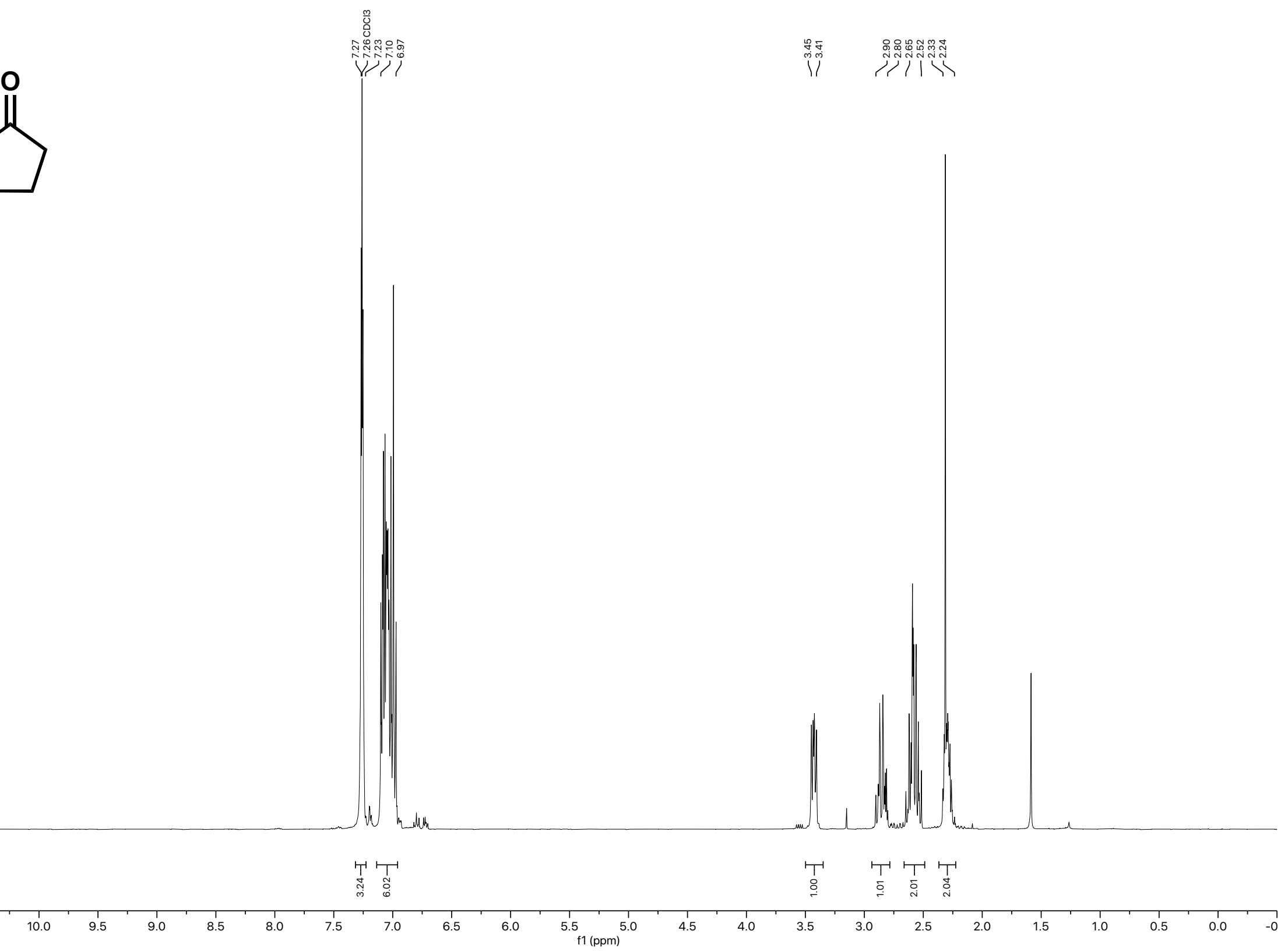

^{1}H NMR (400 MHz, $CDCl_3$) spectrum of compound **6**.

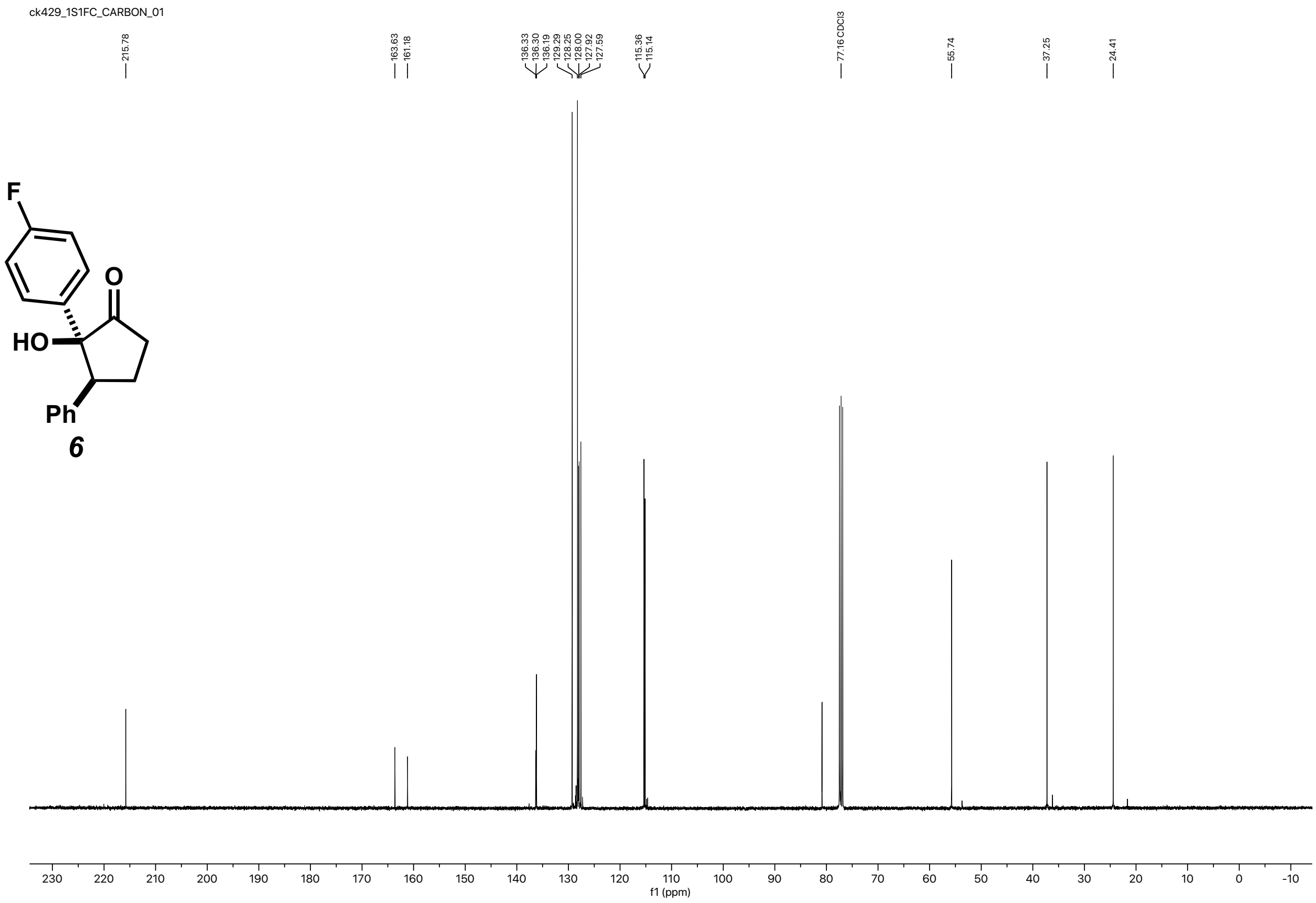

^{13}C NMR (101 MHz, CDCl$_3$) spectrum of compound **6**.

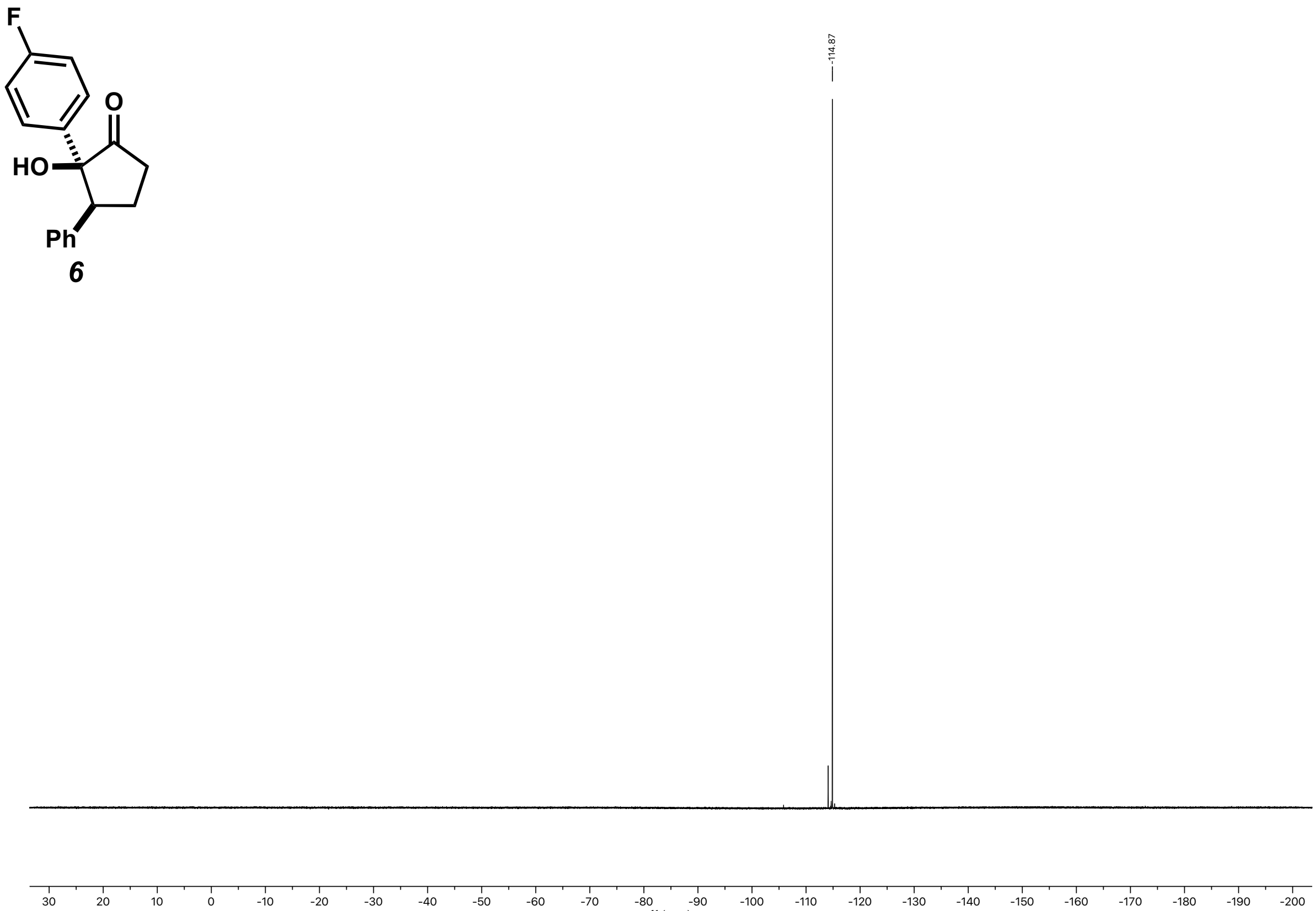

^{19}F NMR (376 MHz, CDCl$_3$) spectrum of compound **6**.

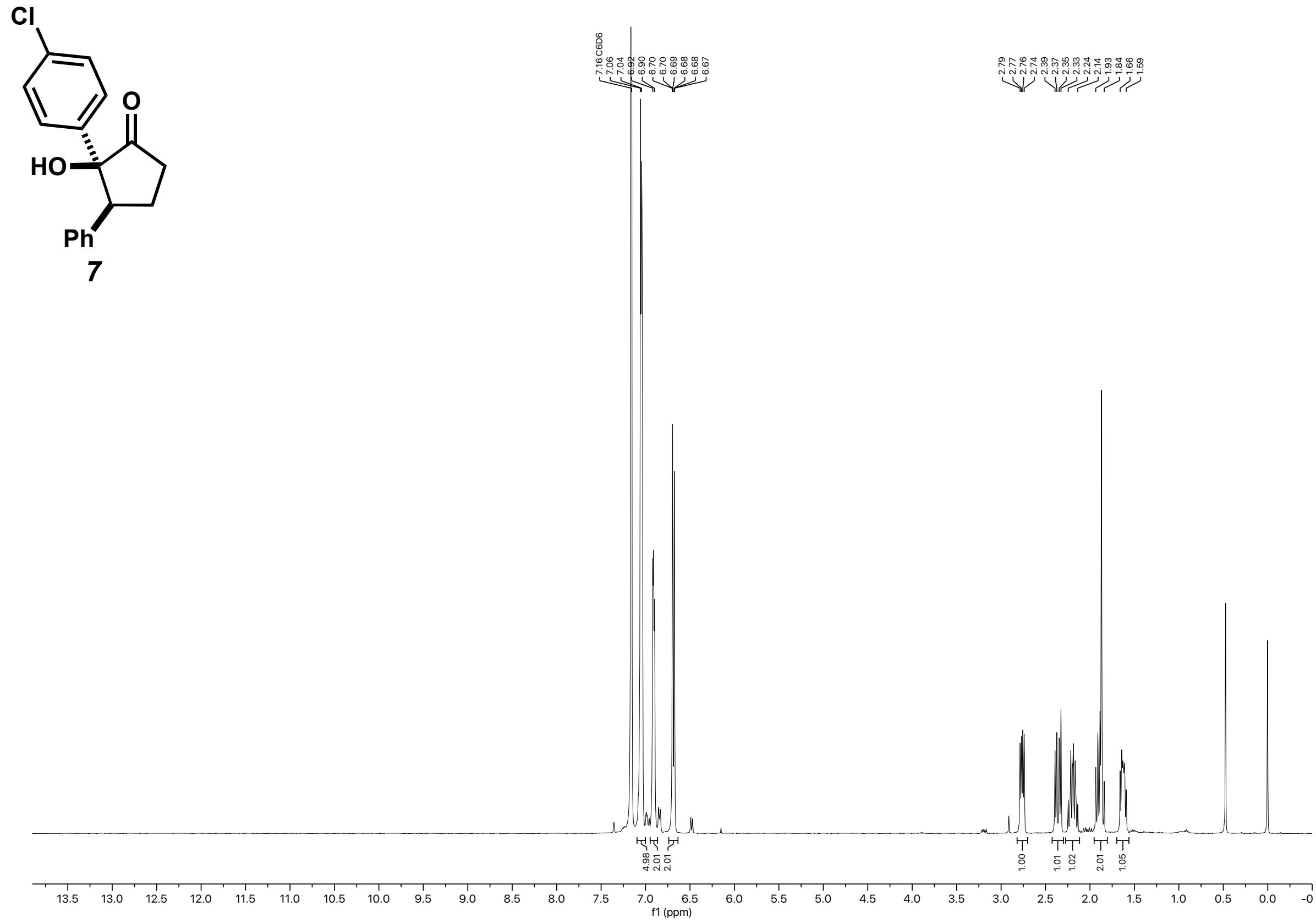

^{1}H NMR (400 MHz, C_6D_6) spectrum of compound **7**.

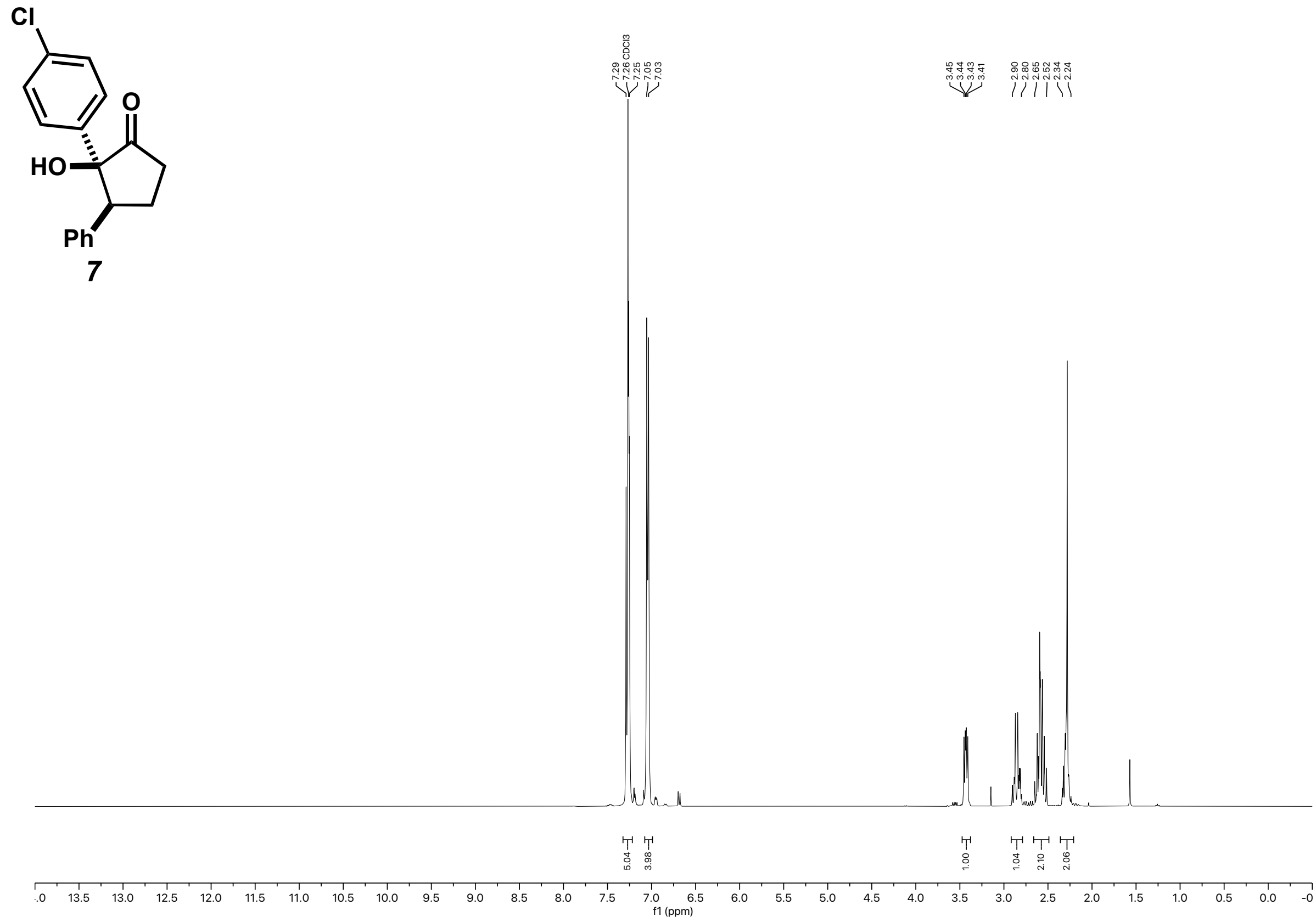

^{1}H NMR (400 MHz, $CDCl_3$) spectrum of compound **7**.

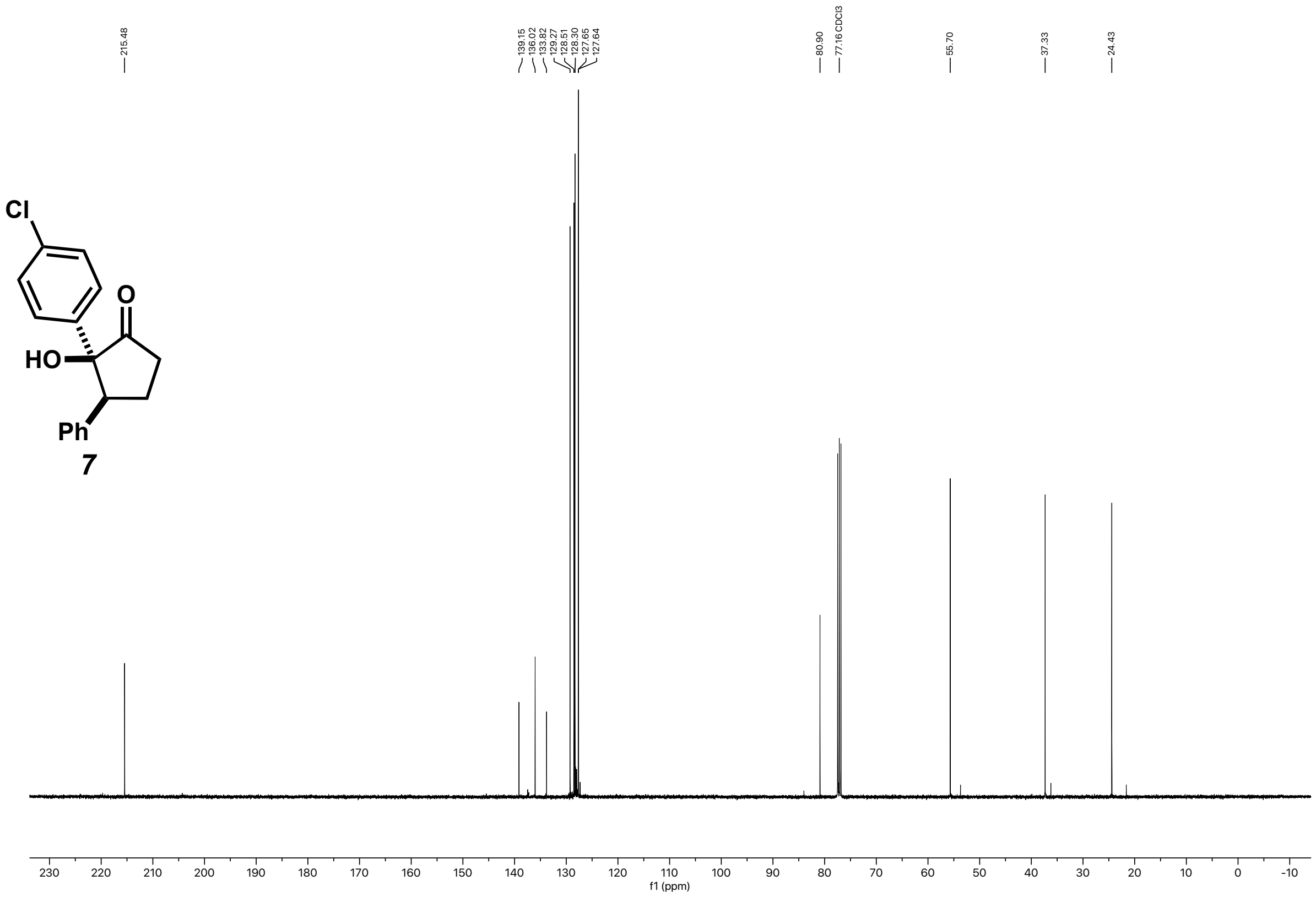

^{13}C NMR (101 MHz, $CDCl_3$) spectrum of compound **7**.

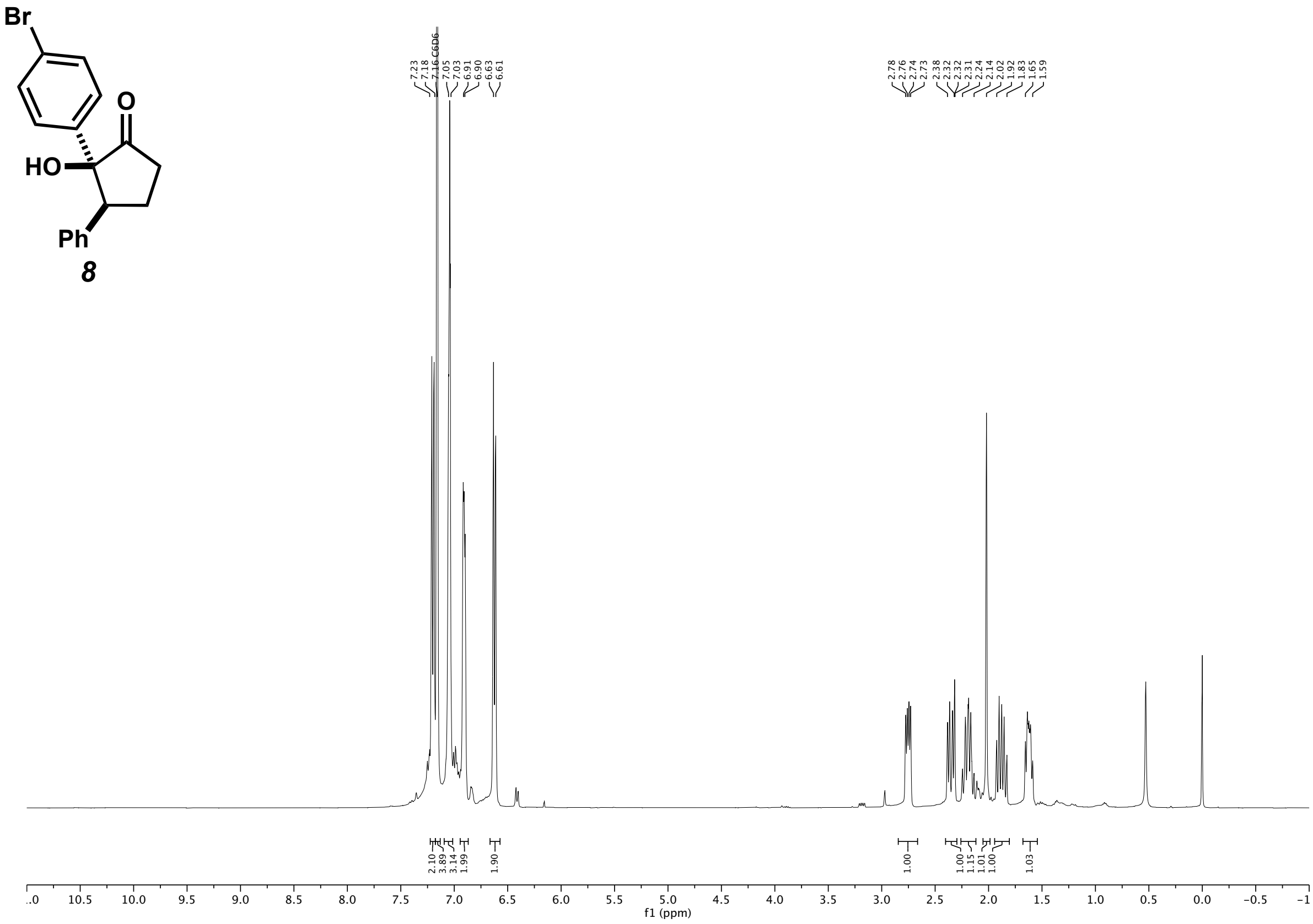

^{1}H NMR (400 MHz, C_6D_6) spectrum of compound **8**.

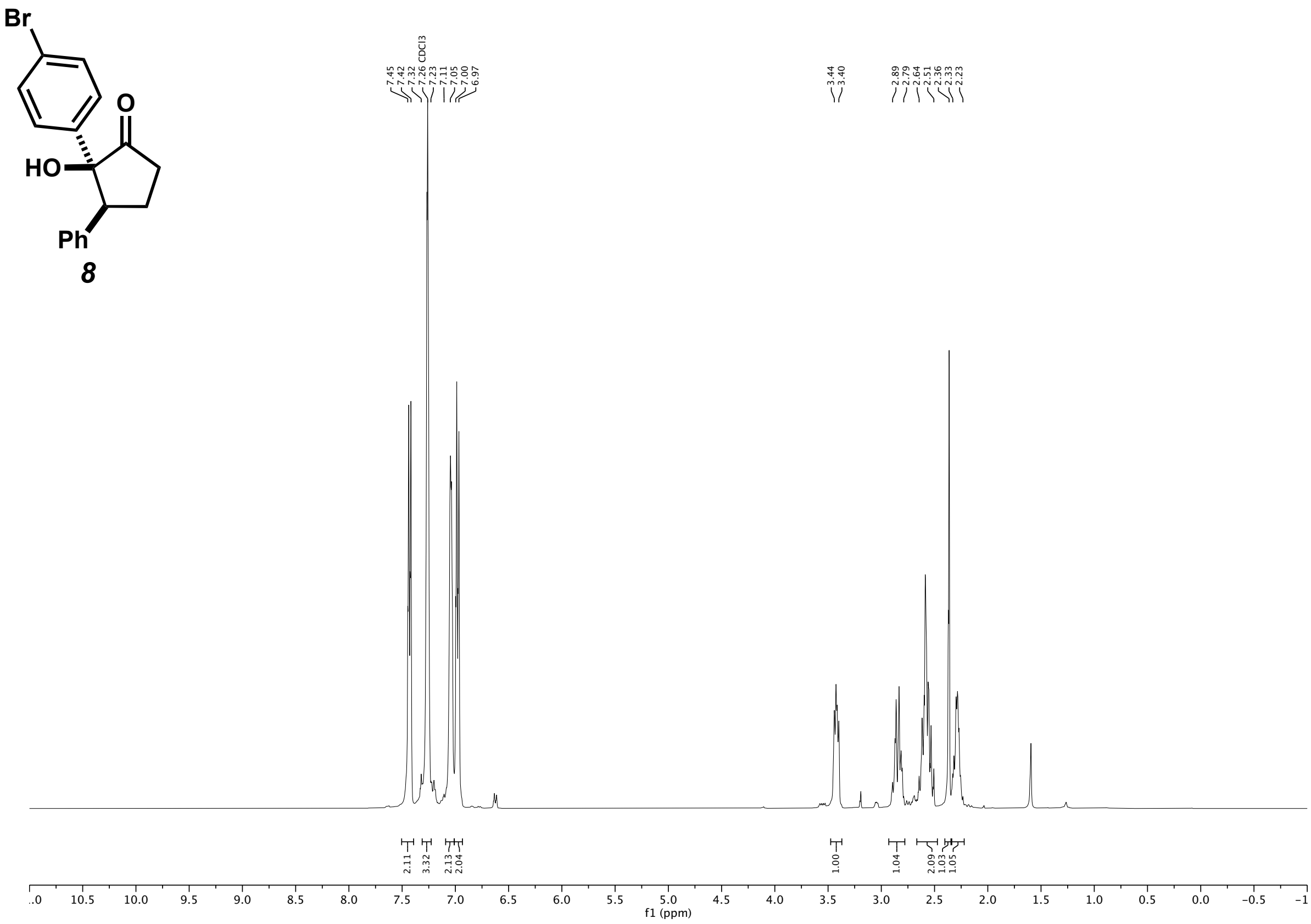

^{1}H NMR (400 MHz, $CDCl_3$) spectrum of compound **8**.

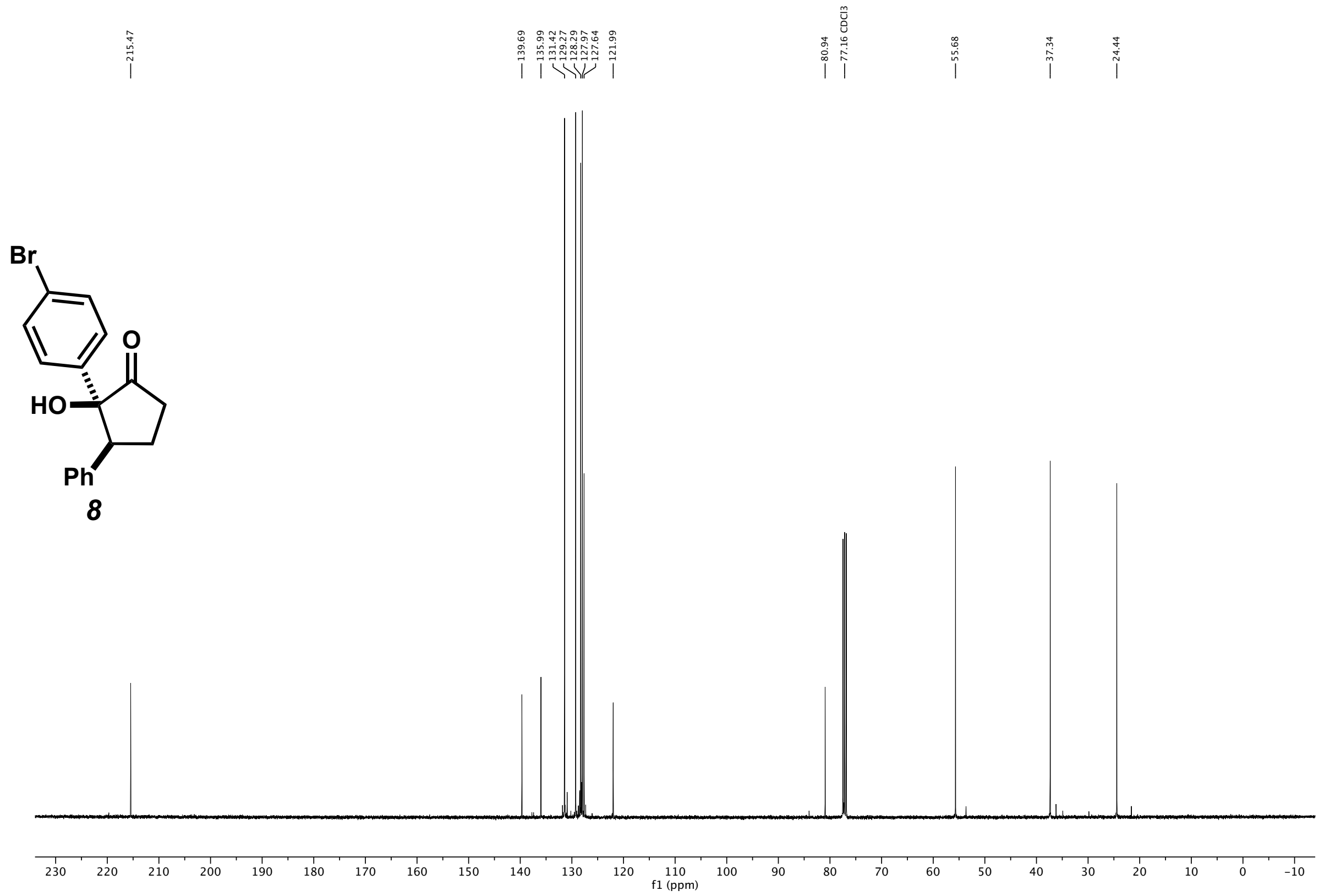

^{13}C NMR (101 MHz, $CDCl_3$) spectrum of compound **8**.

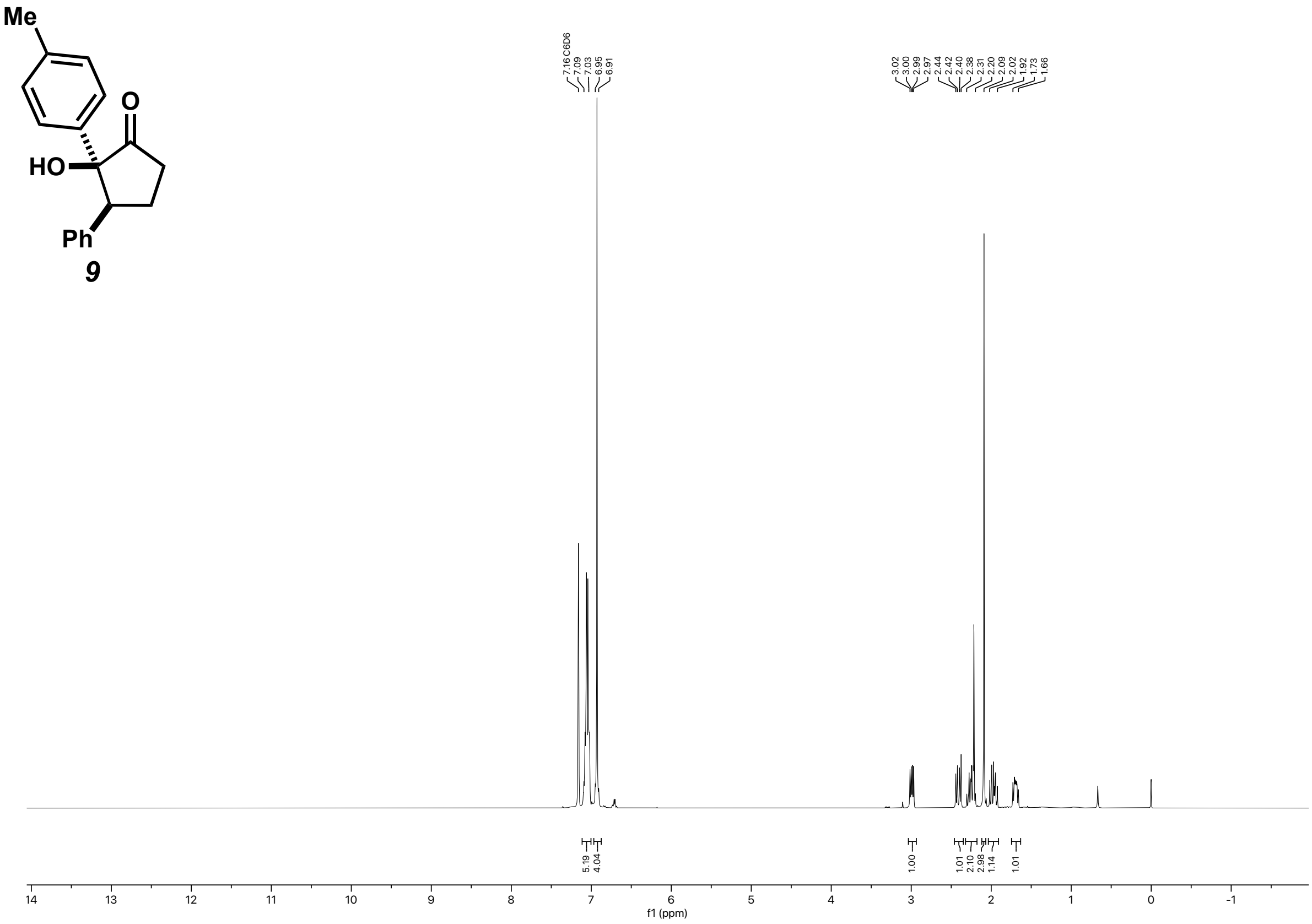

^{1}H NMR (400 MHz, C_6D_6) spectrum of compound **9**.

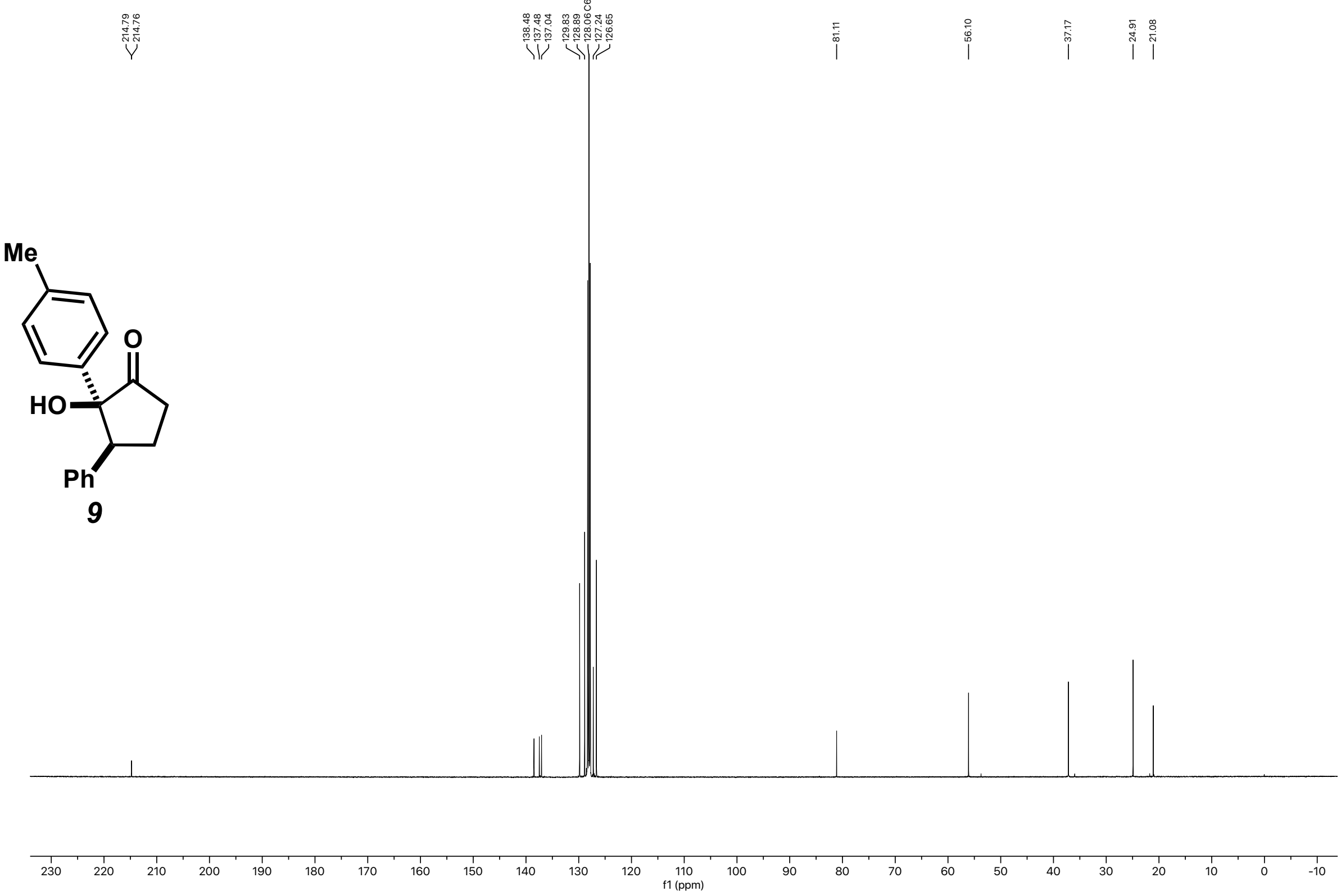

^{13}C NMR (101 MHz, C_6D_6) spectrum of compound **9**.

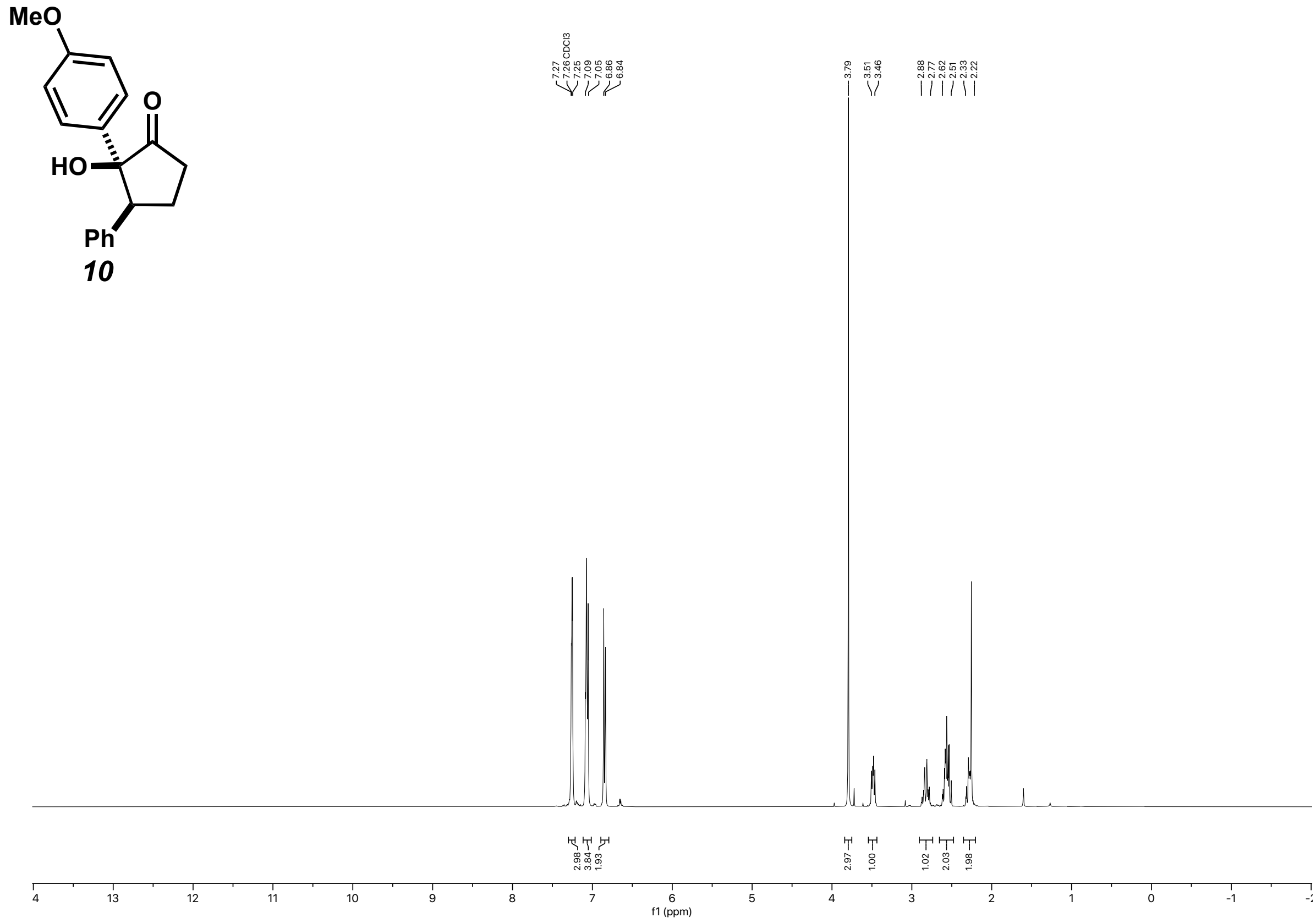

^{1}H NMR (400 MHz, $CDCl_3$) spectrum of compound **10**.

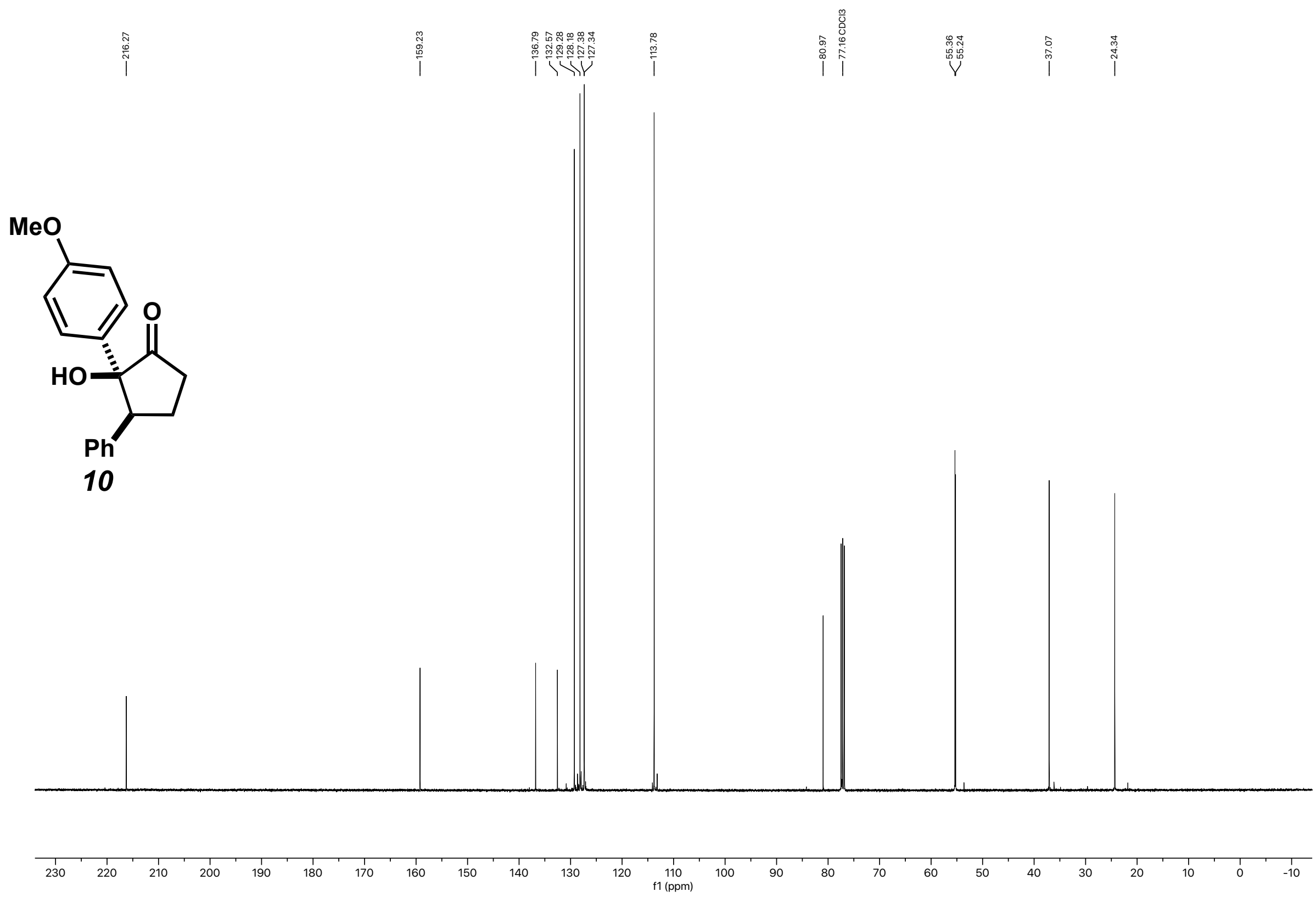

^{13}C NMR (101 MHz, $CDCl_3$) spectrum of compound **10**.

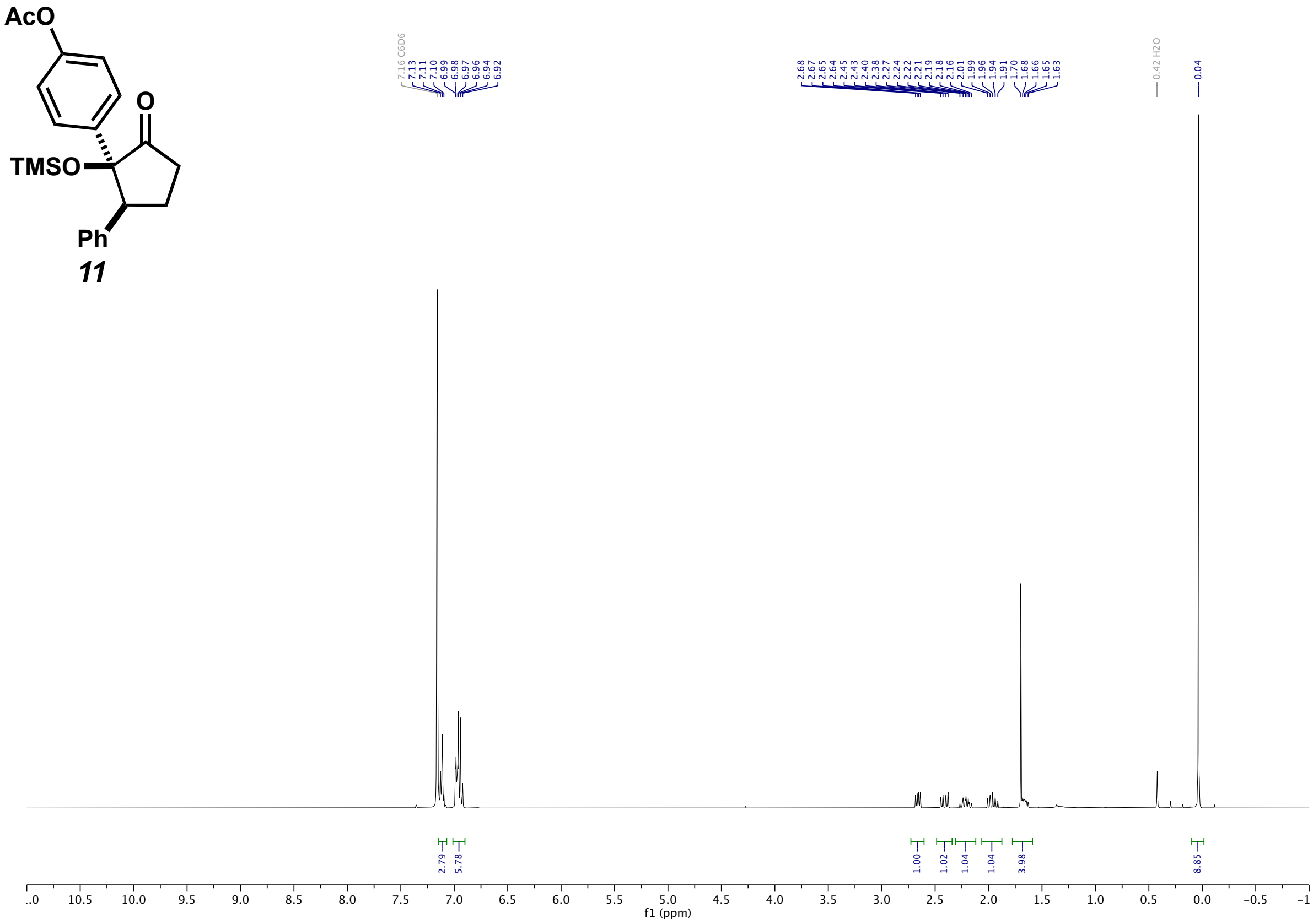

^{1}H NMR (400 MHz, C_6D_6) spectrum of compound **11**.

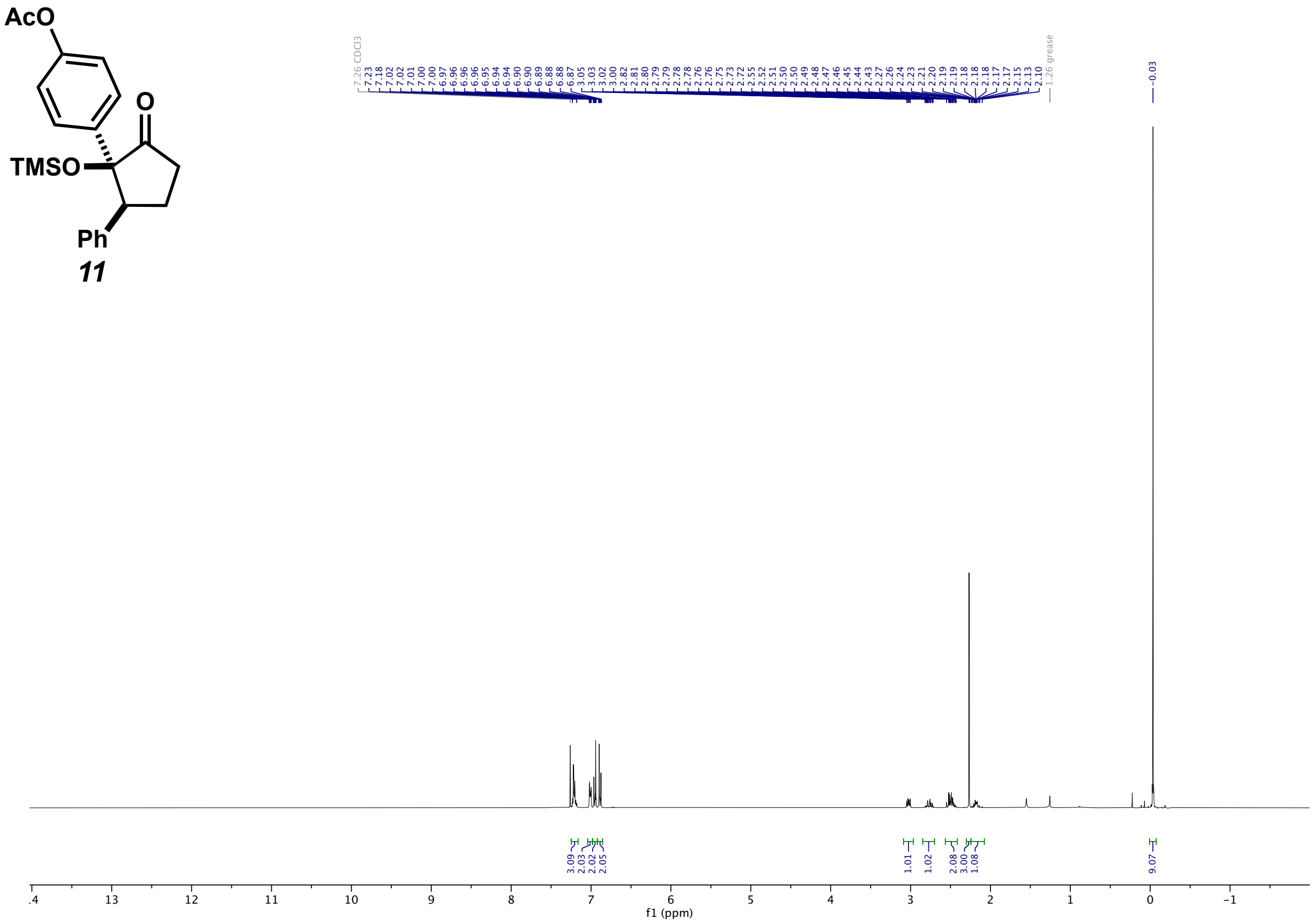

^{1}H NMR (400 MHz, $CDCl_3$) spectrum of compound **11**.

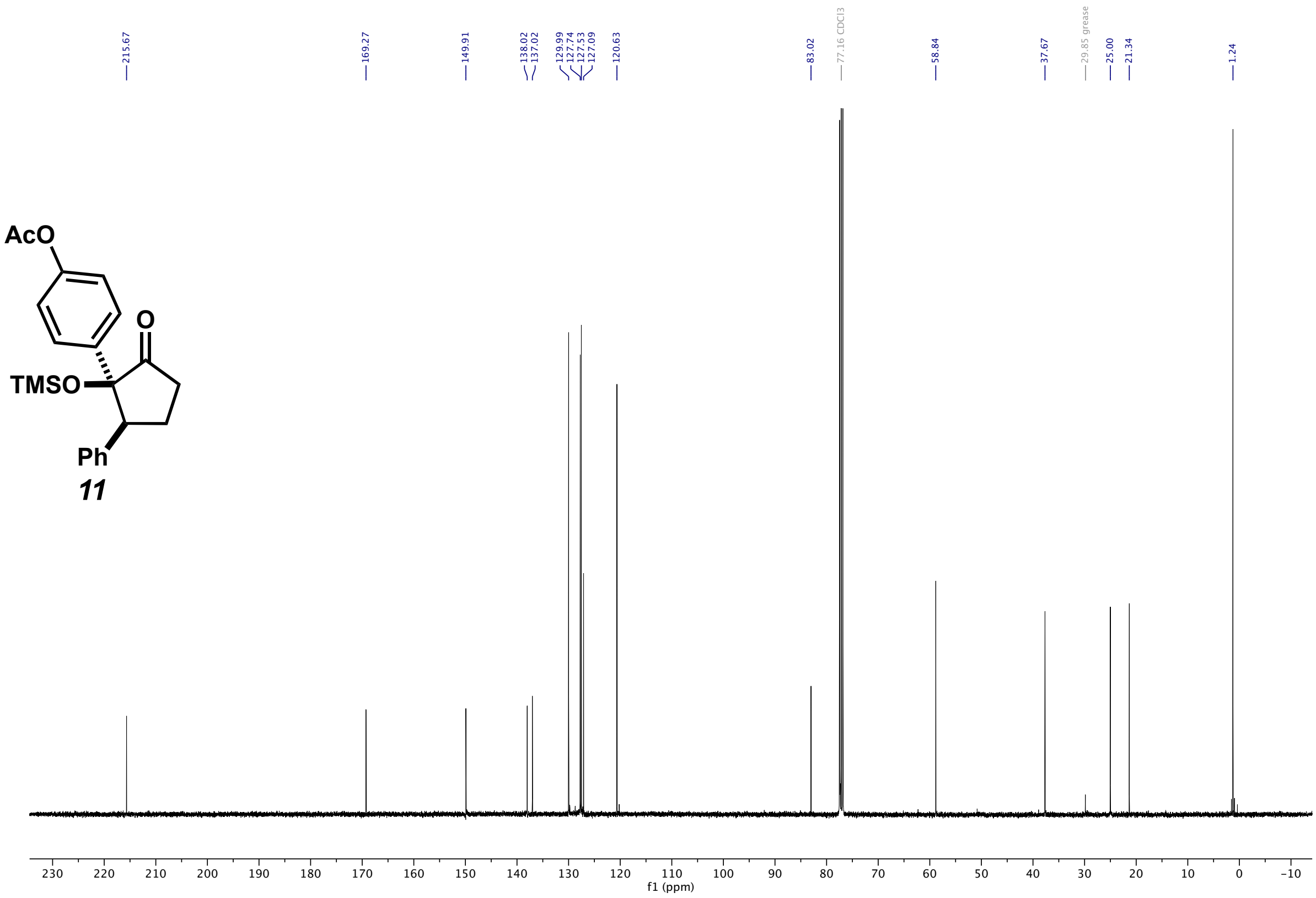

^{13}C NMR (101 MHz, $CDCl_3$) spectrum of compound **11**.

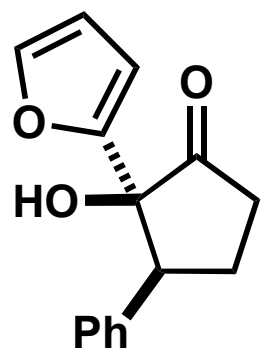

12

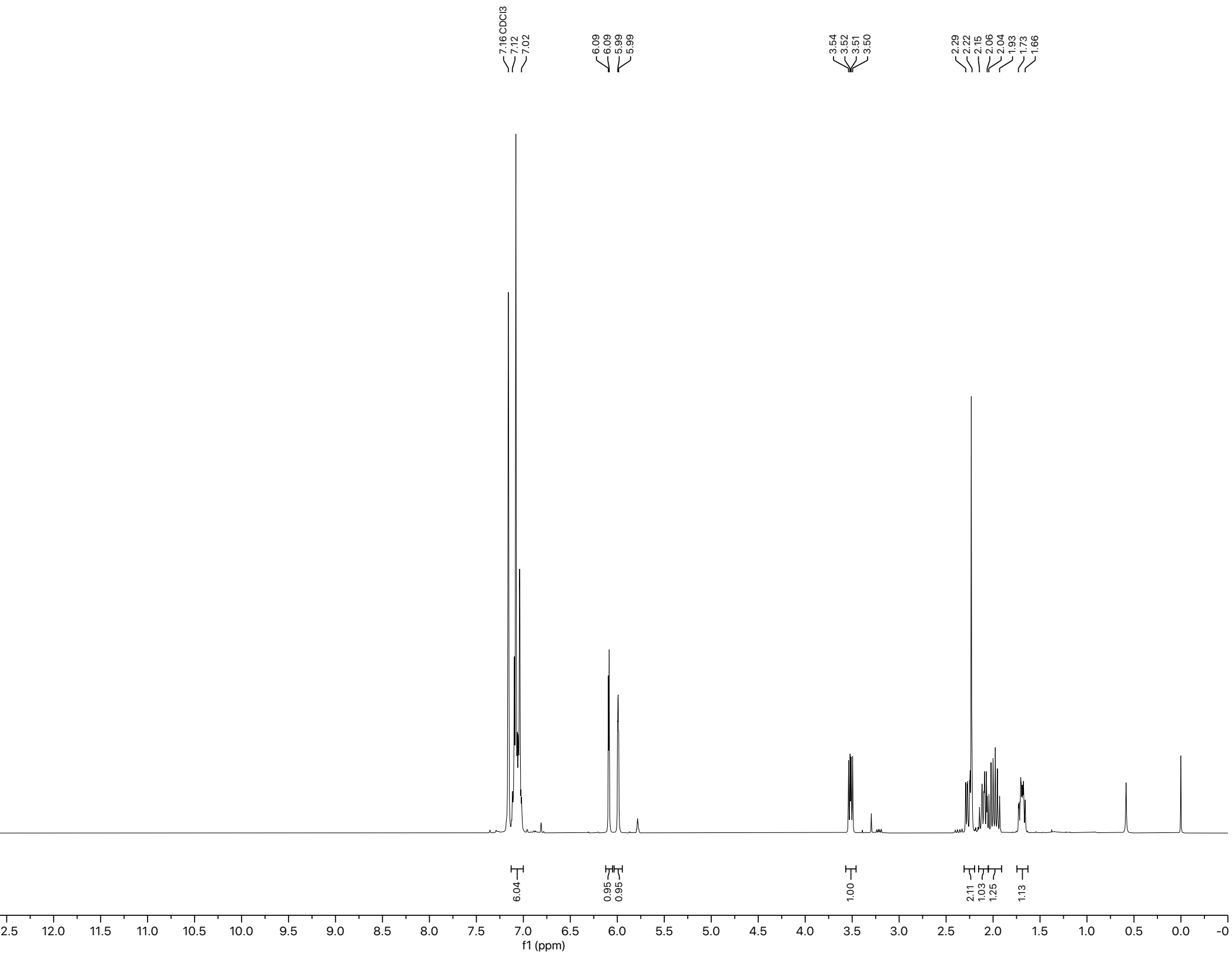

^{1}H NMR (400 MHz, C_6D_6) spectrum of compound **12**.

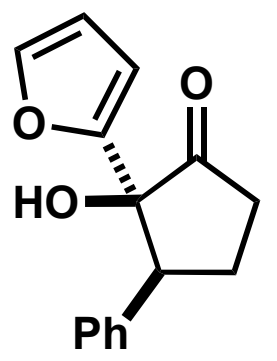

12

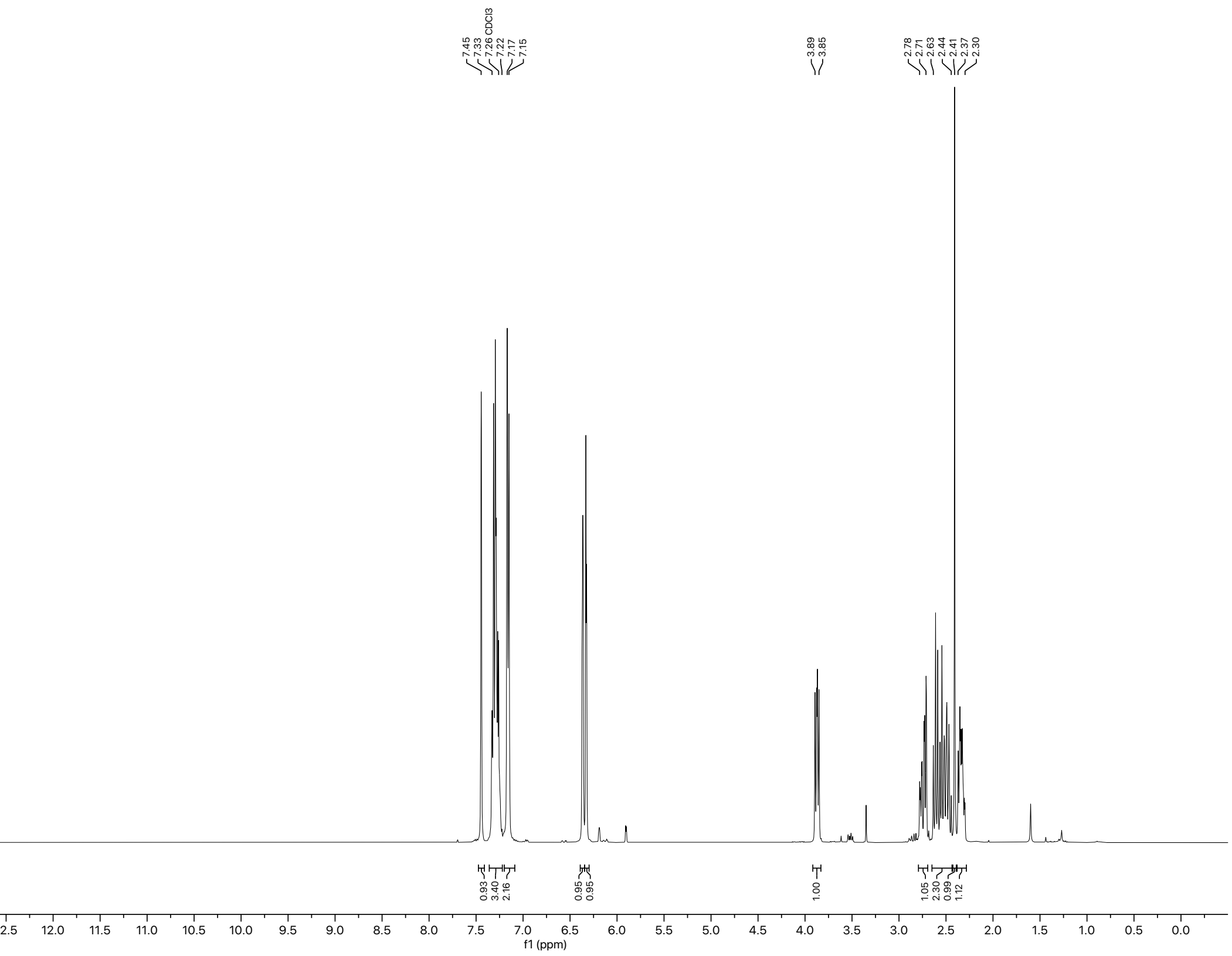

^{1}H NMR (400 MHz, $CDCl_3$) spectrum of compound **12**.

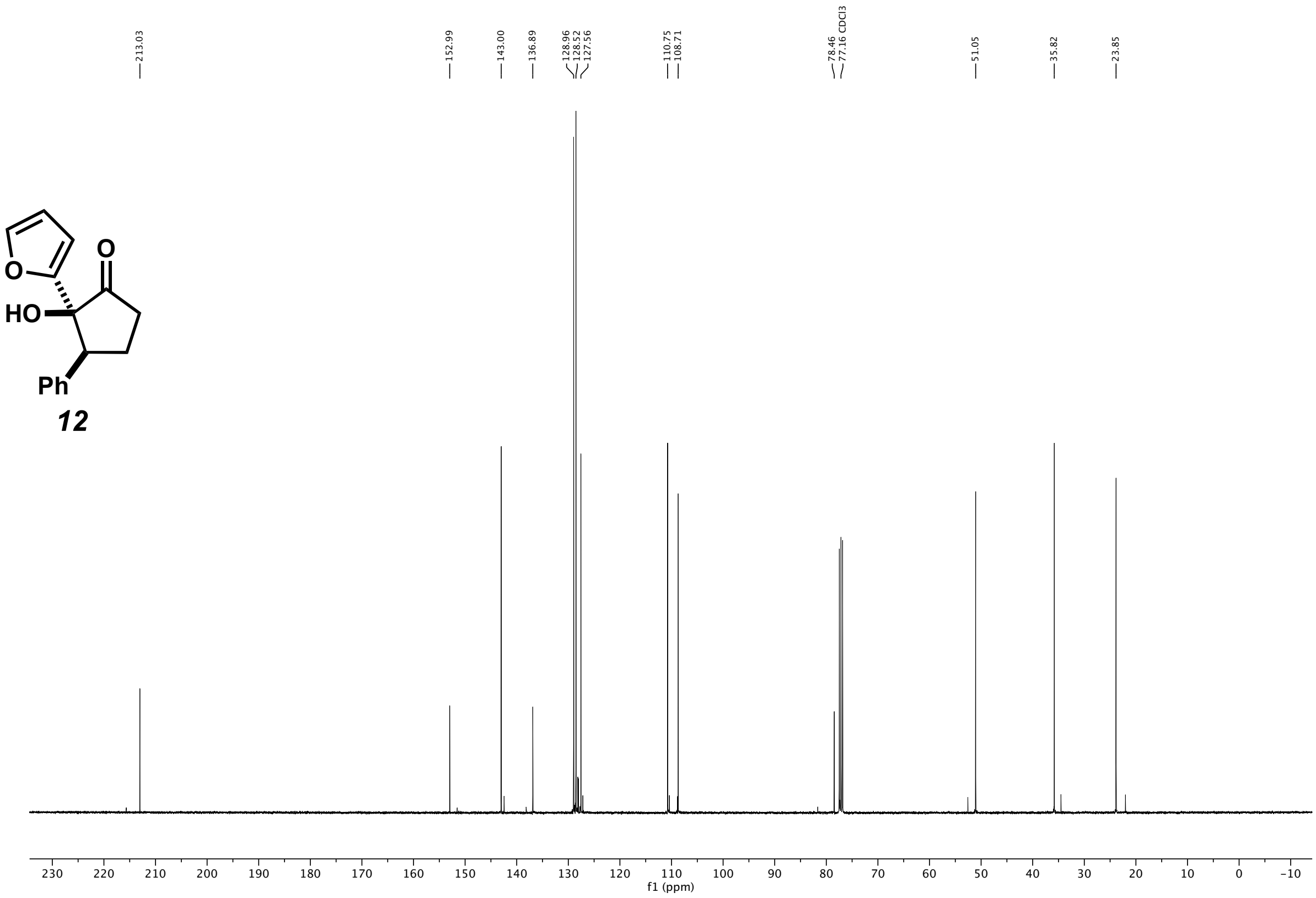

^{13}C NMR (101 MHz, $CDCl_3$) spectrum of compound **12**.

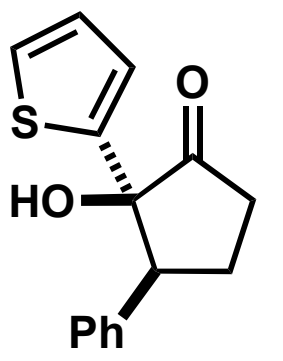

13

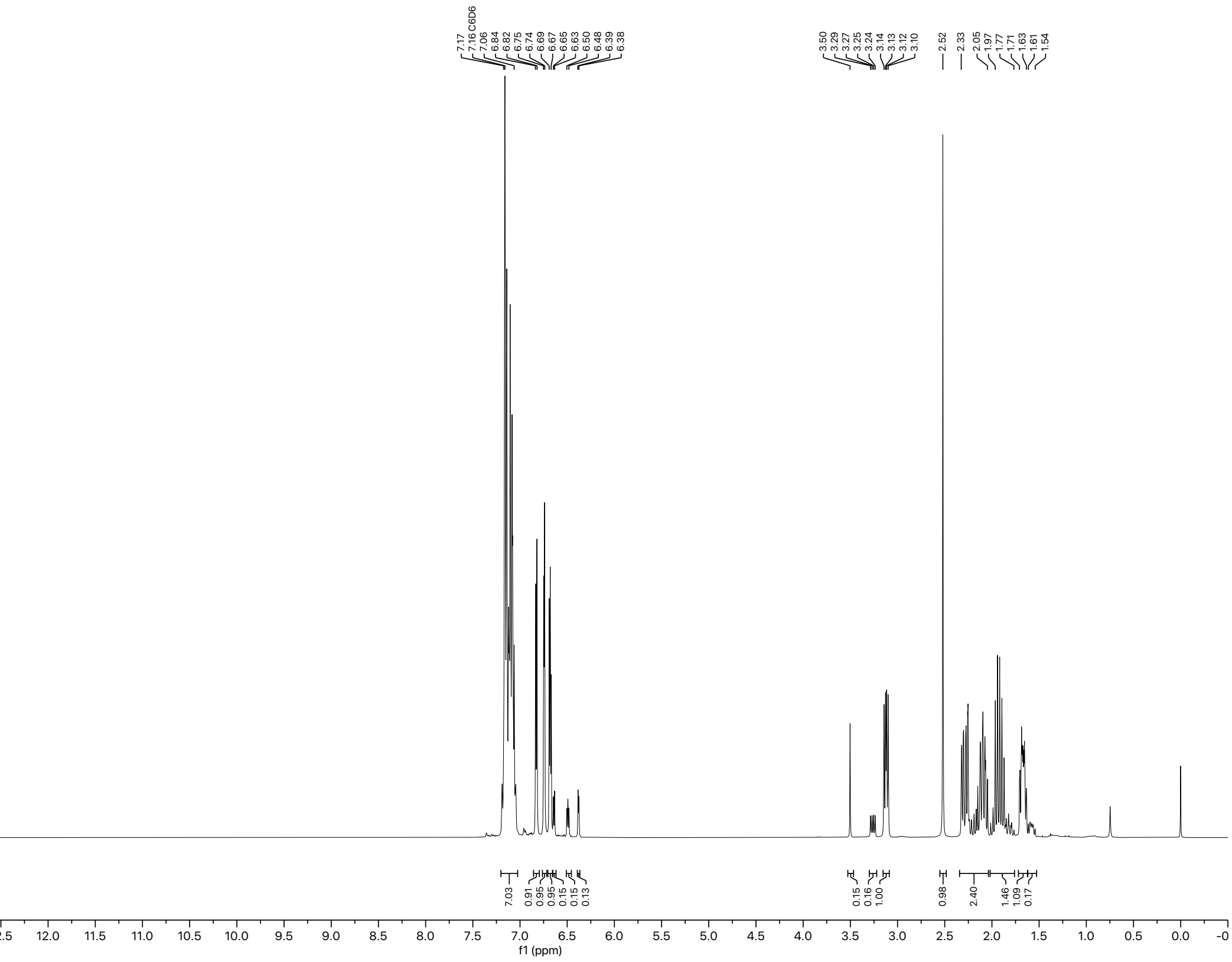

^{1}H NMR (400 MHz, C_6D_6) spectrum of compound **13**.

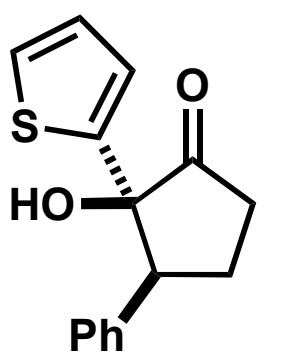

13

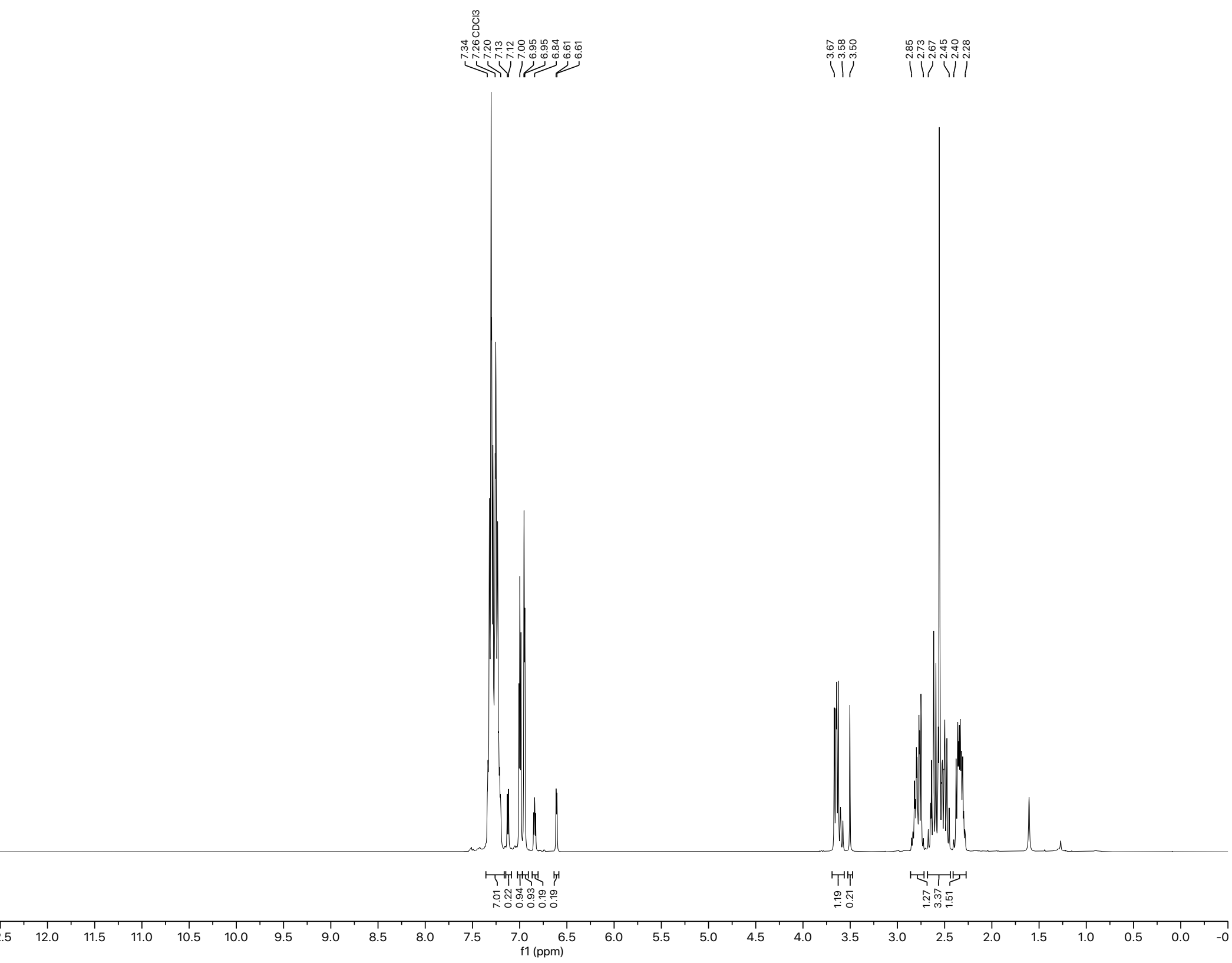

^{1}H NMR (400 MHz, $CDCl_3$) spectrum of compound **13**.

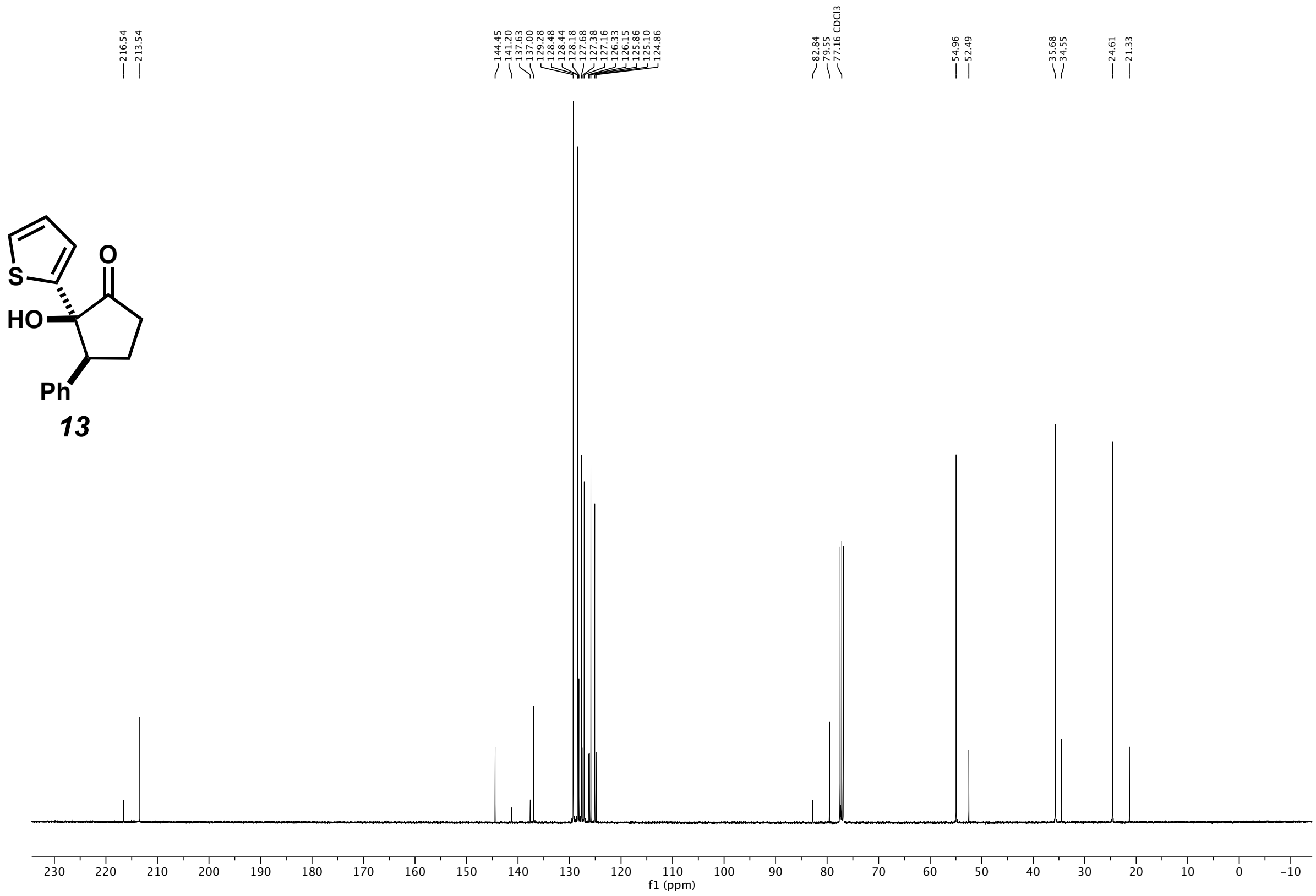

^{13}C NMR (101 MHz, $CDCl_3$) spectrum of compound **13**.

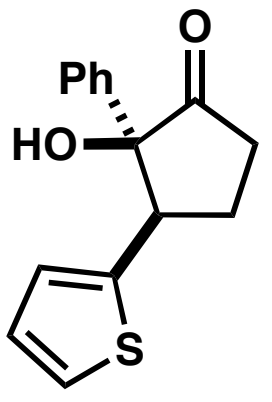

14

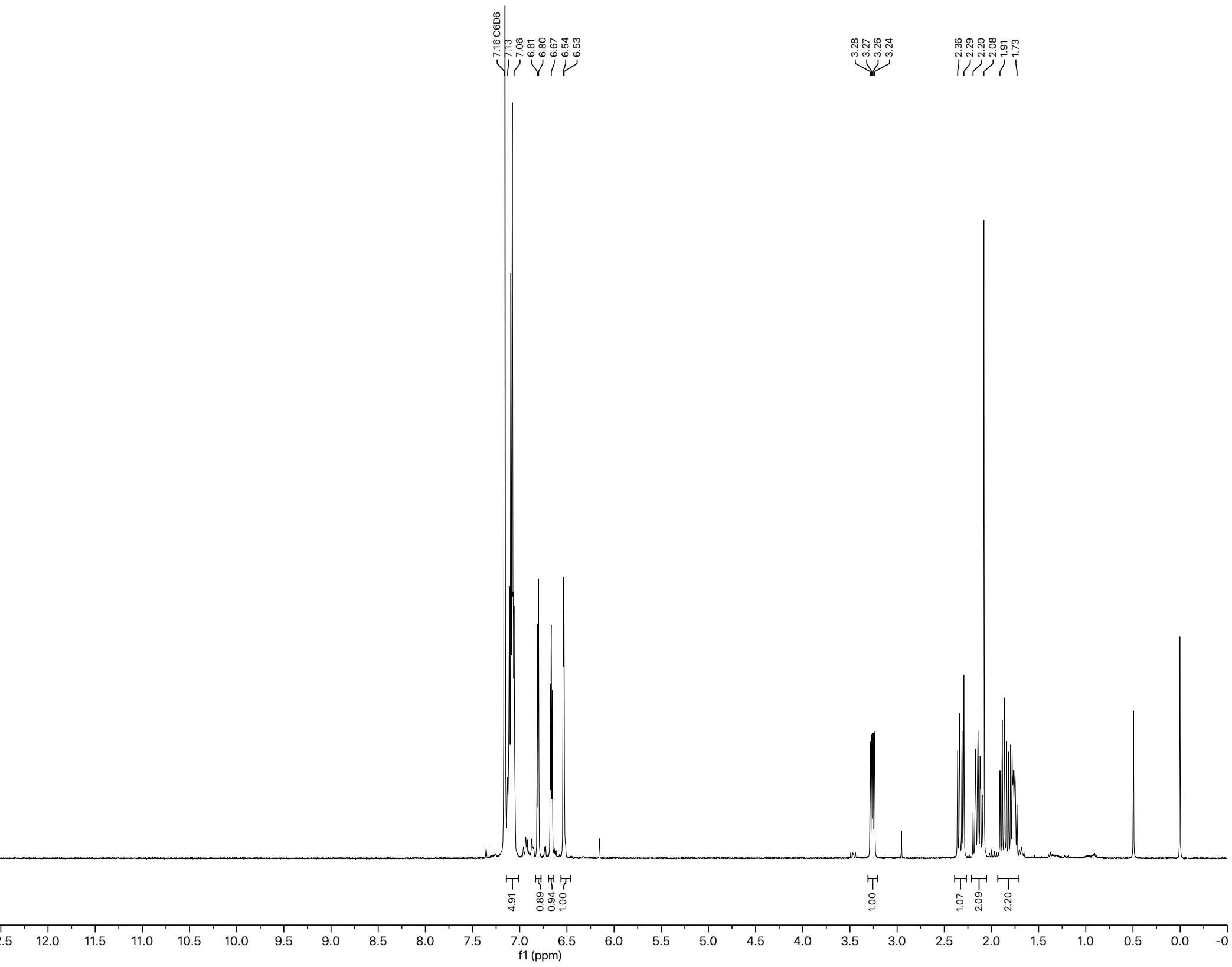

^{1}H NMR (400 MHz, C_6D_6) spectrum of compound **14**.

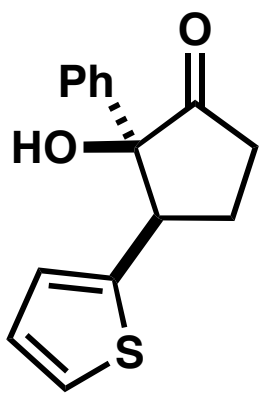

14

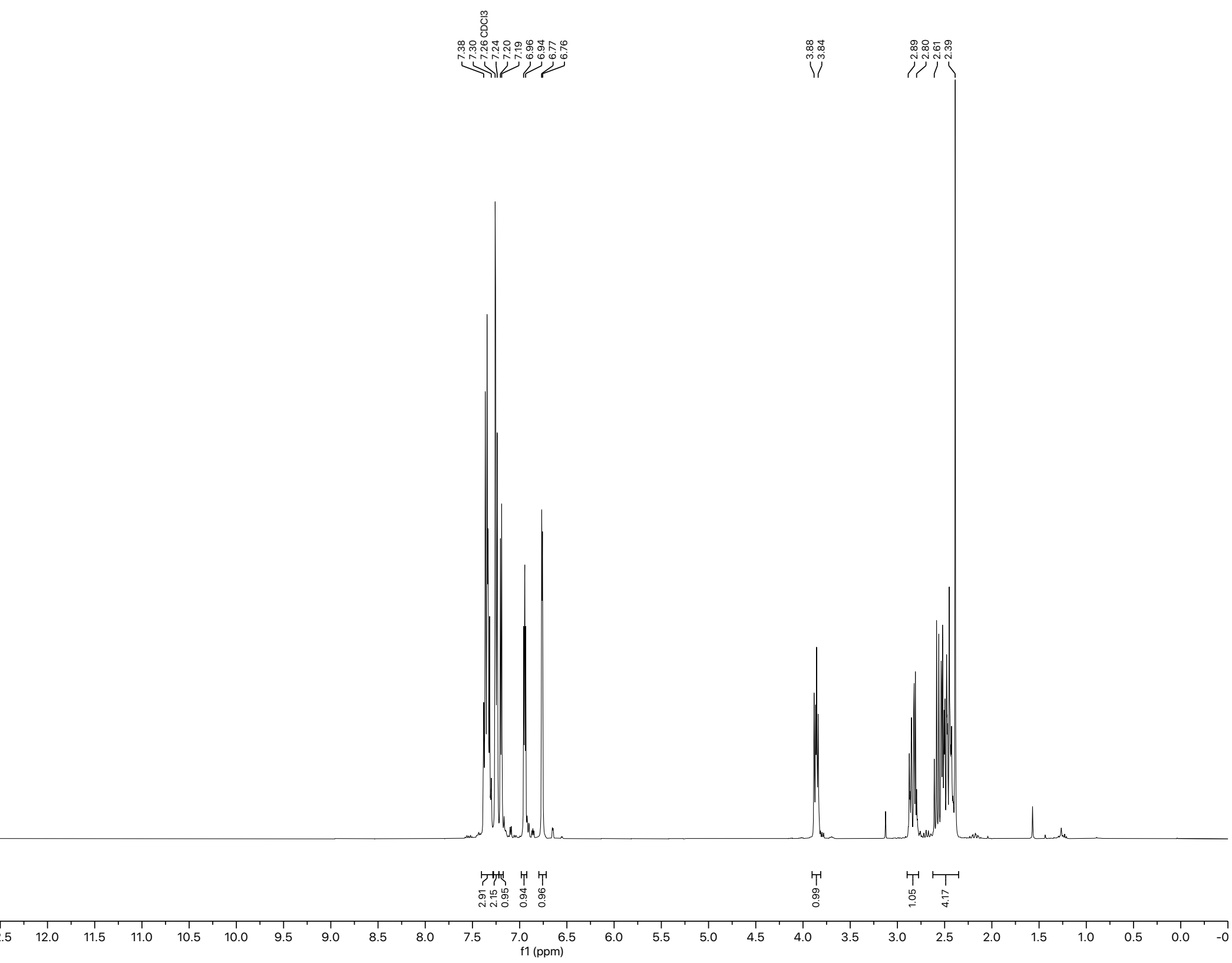

^{1}H NMR (400 MHz, $CDCl_3$) spectrum of compound **14**.

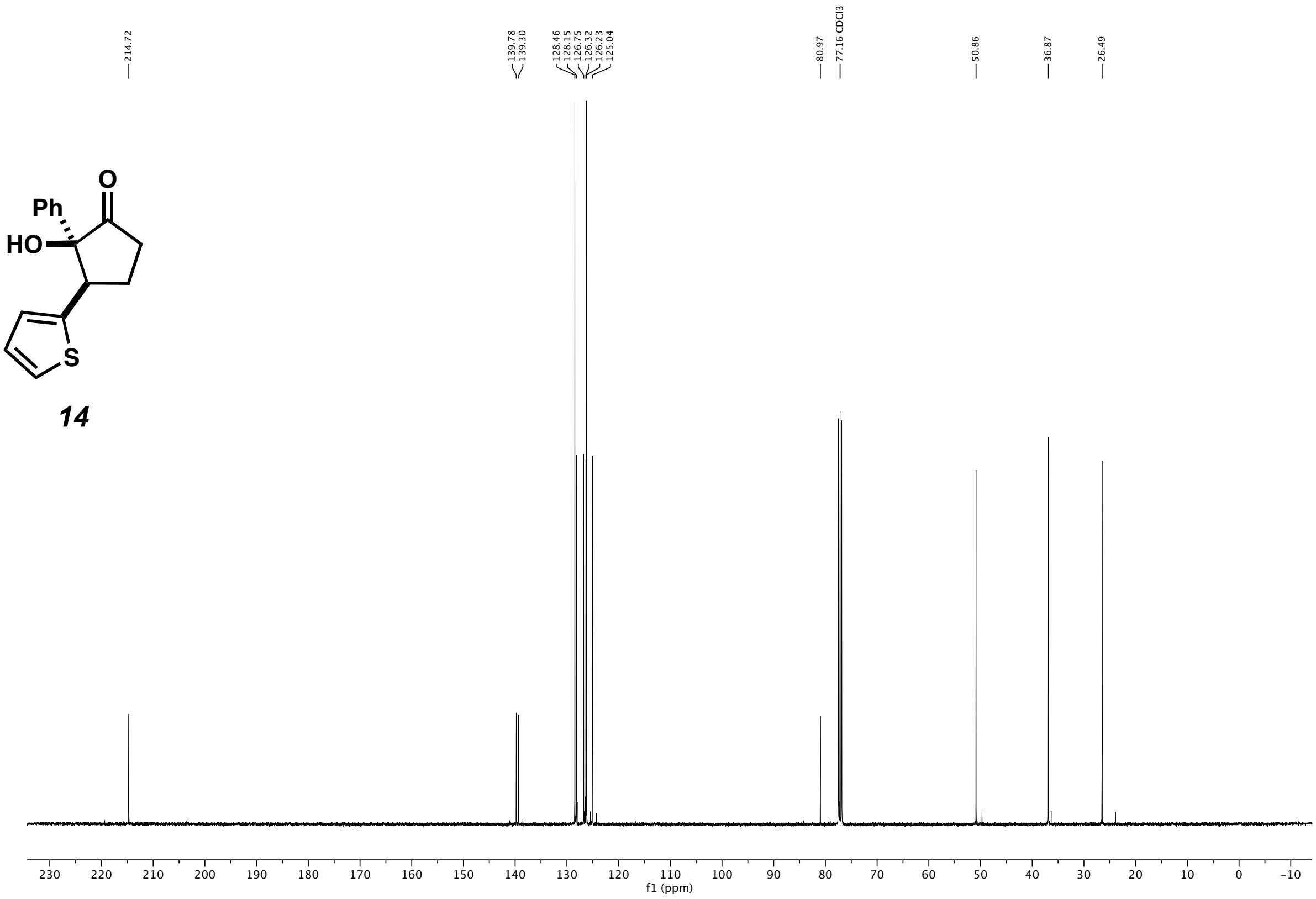

14

^{13}C NMR (101 MHz, $CDCl_3$) spectrum of compound **14**.

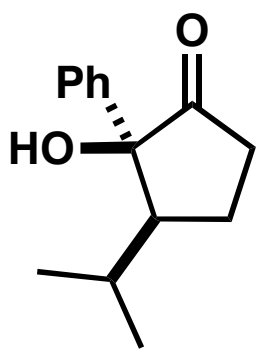

15

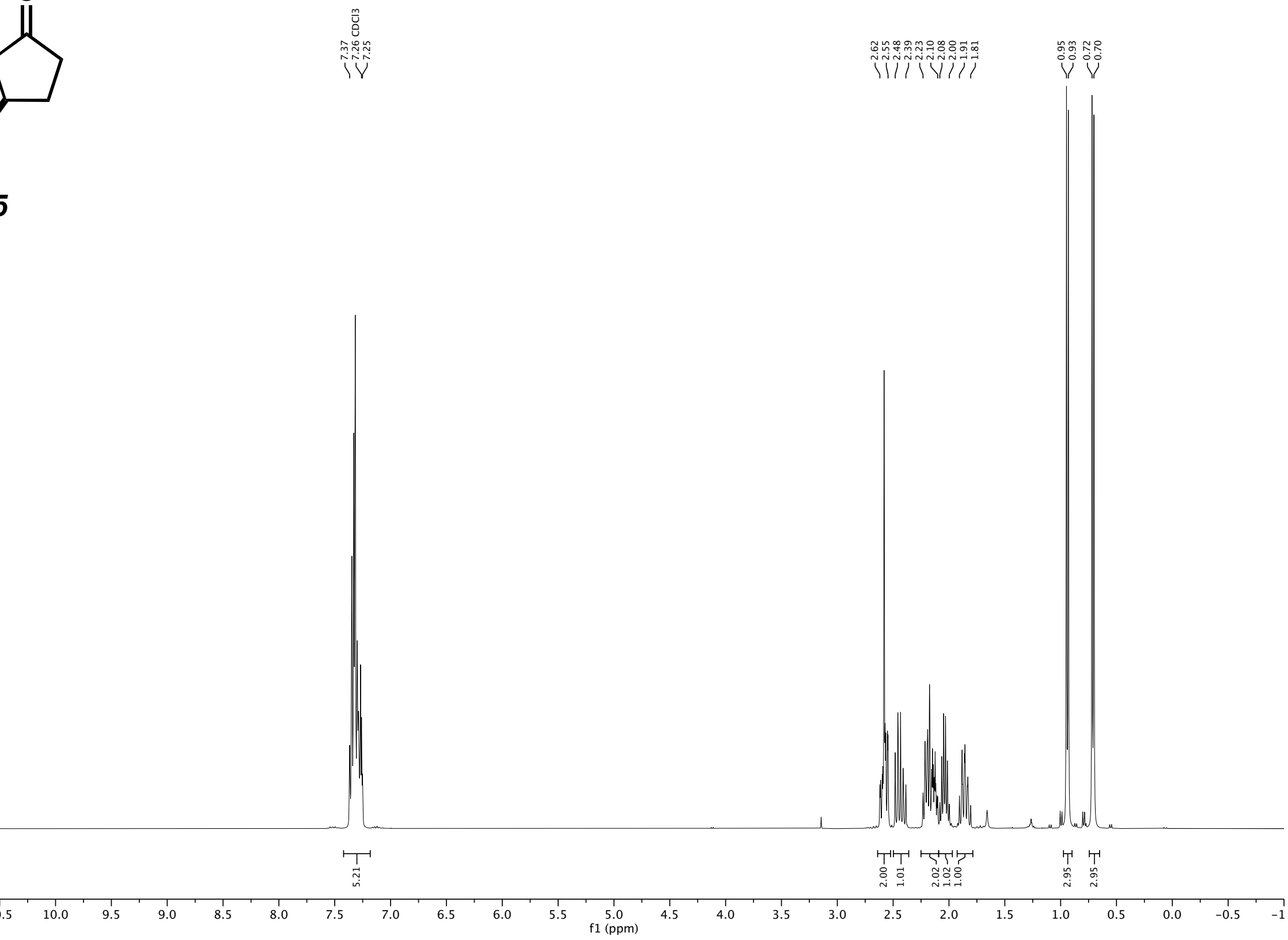

^{1}H NMR (400 MHz, $CDCl_3$) spectrum of compound **15**.

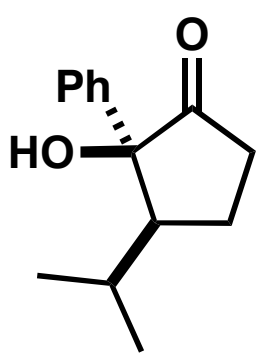

15

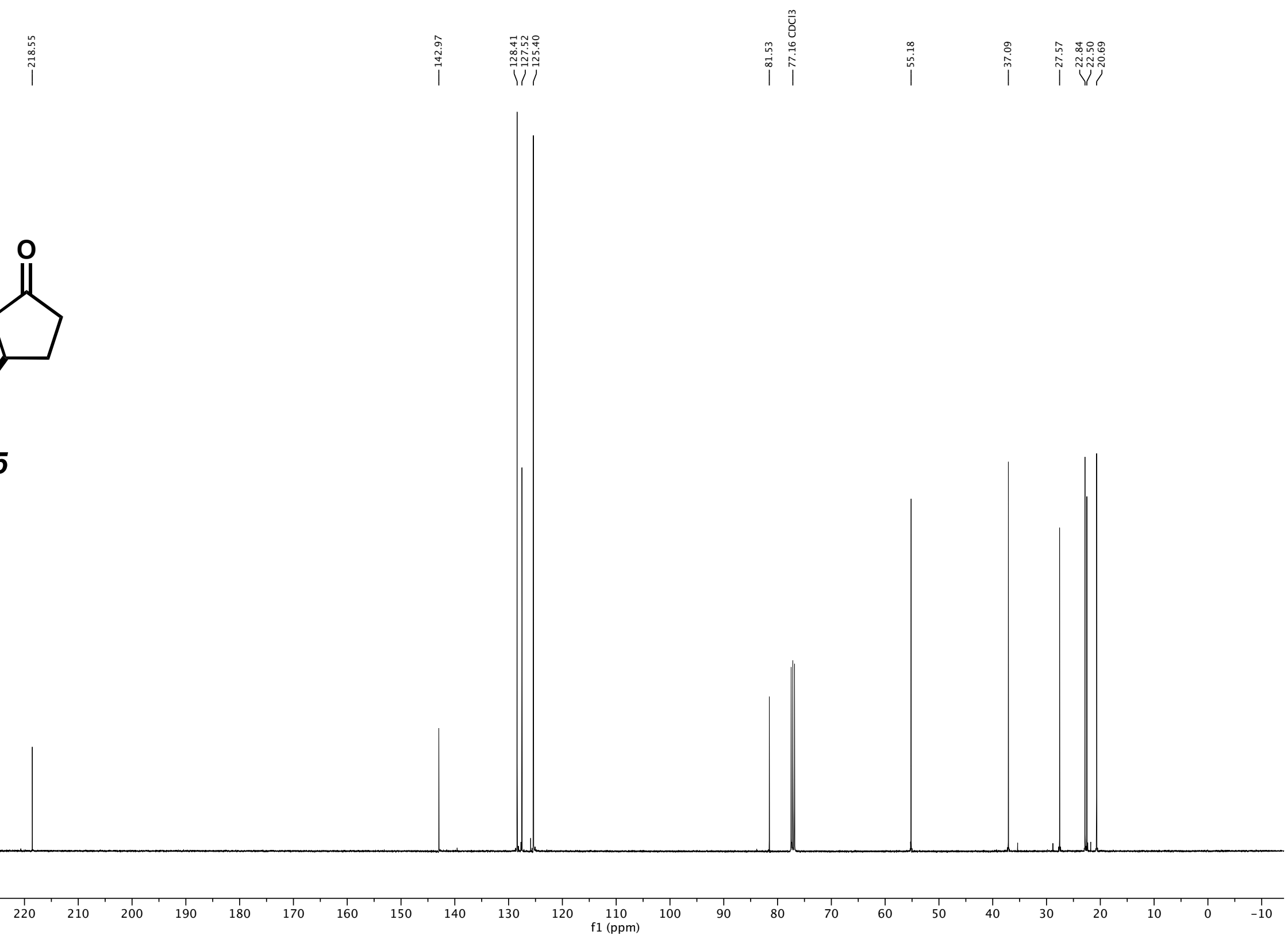

^{13}C NMR (101 MHz, $CDCl_3$) spectrum of compound **15**.

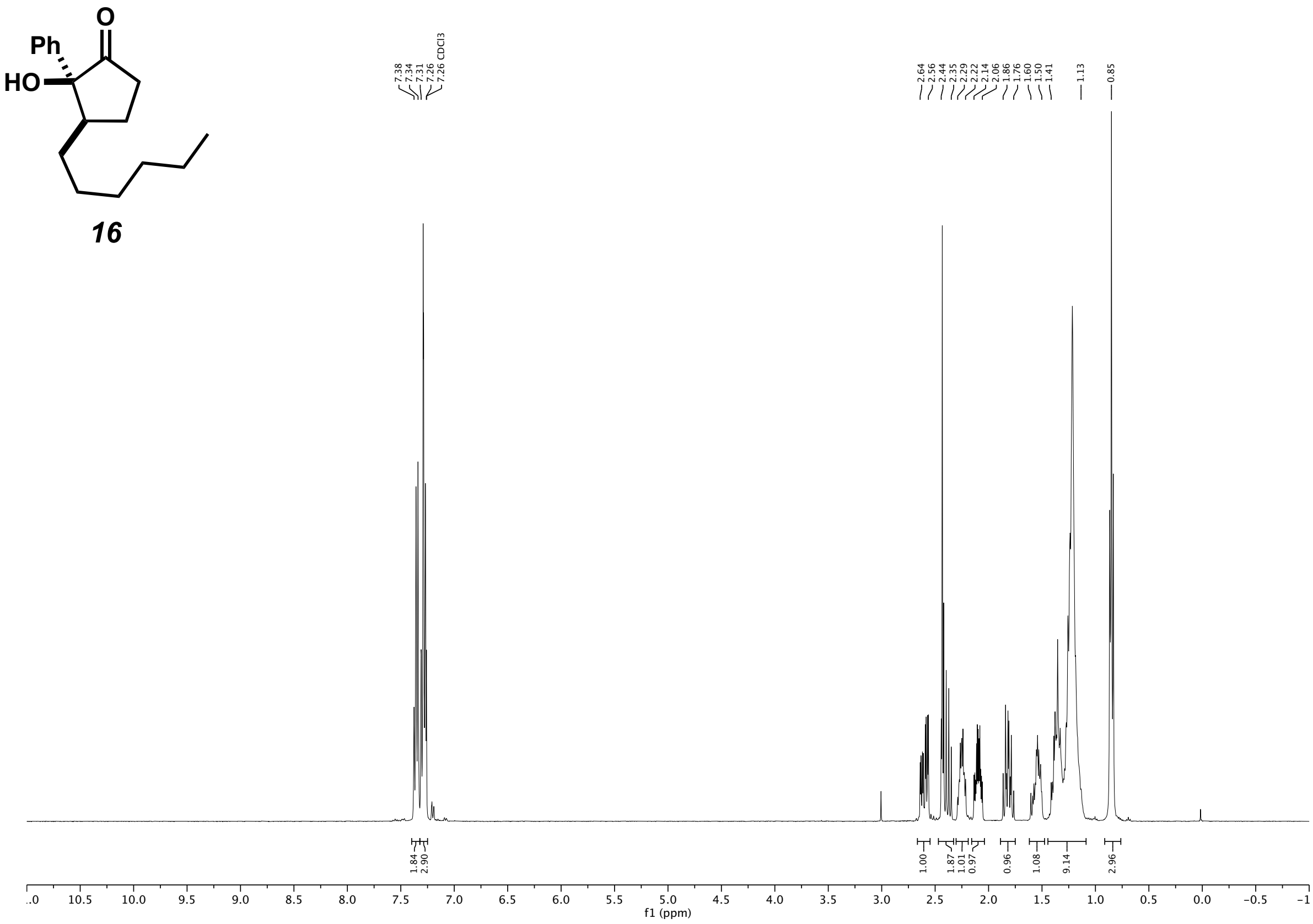

^{1}H NMR (400 MHz, $CDCl_3$) spectrum of compound **16**.

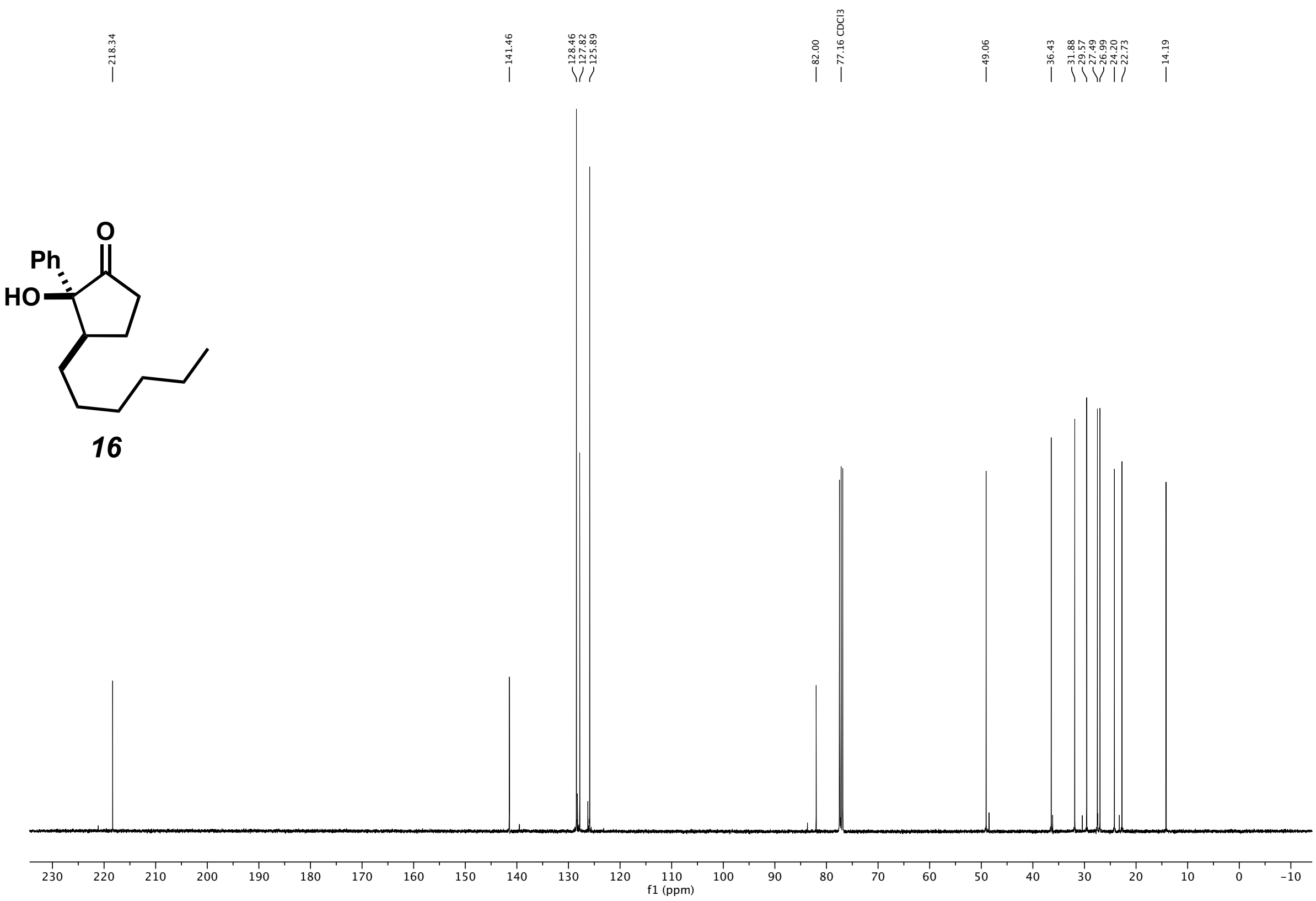

^{13}C NMR (101 MHz, $CDCl_3$) spectrum of compound **16**.

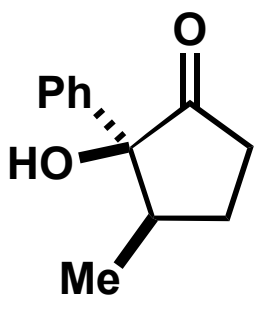

17

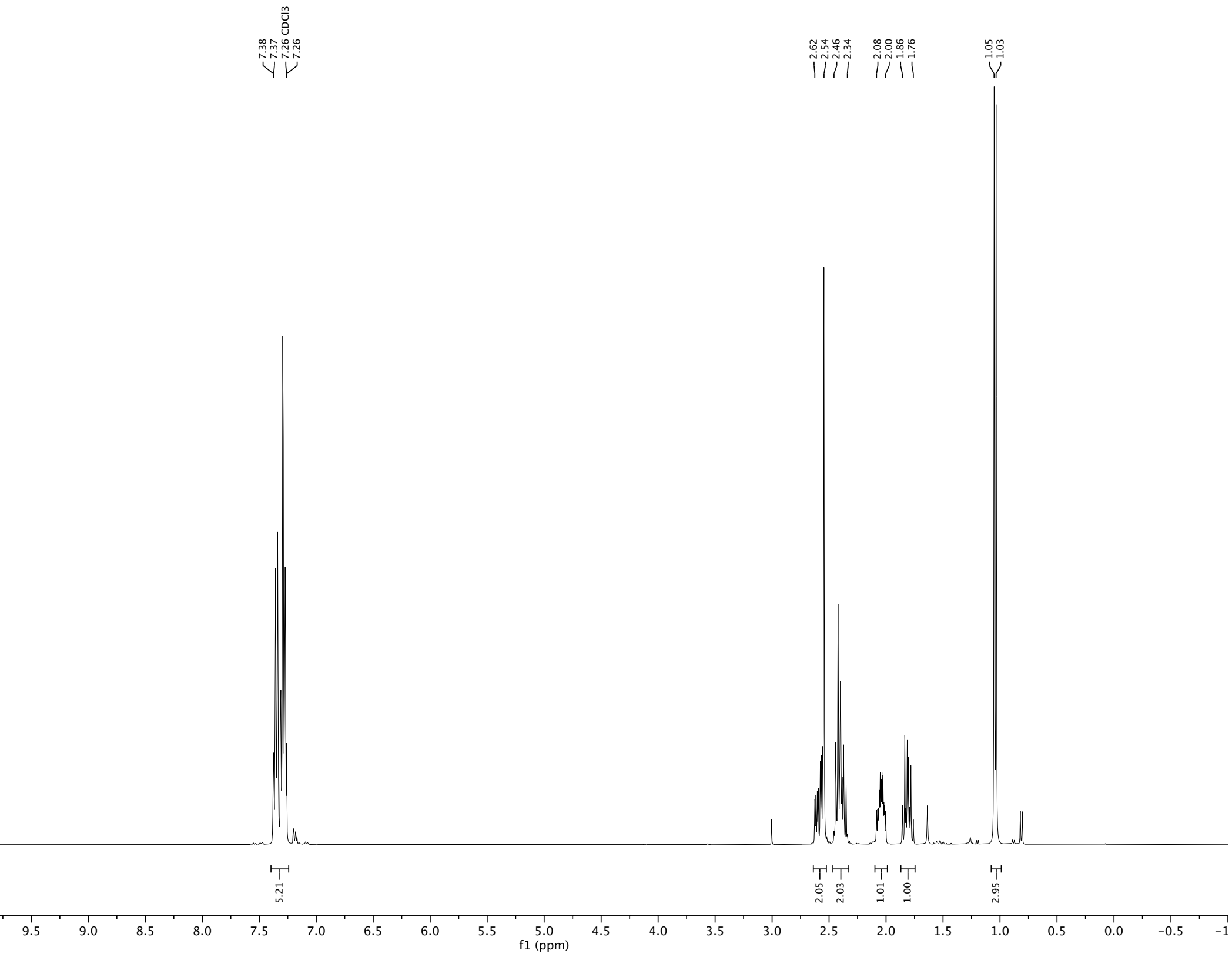

^{1}H NMR (400 MHz, $CDCl_3$) spectrum of compound **17**.

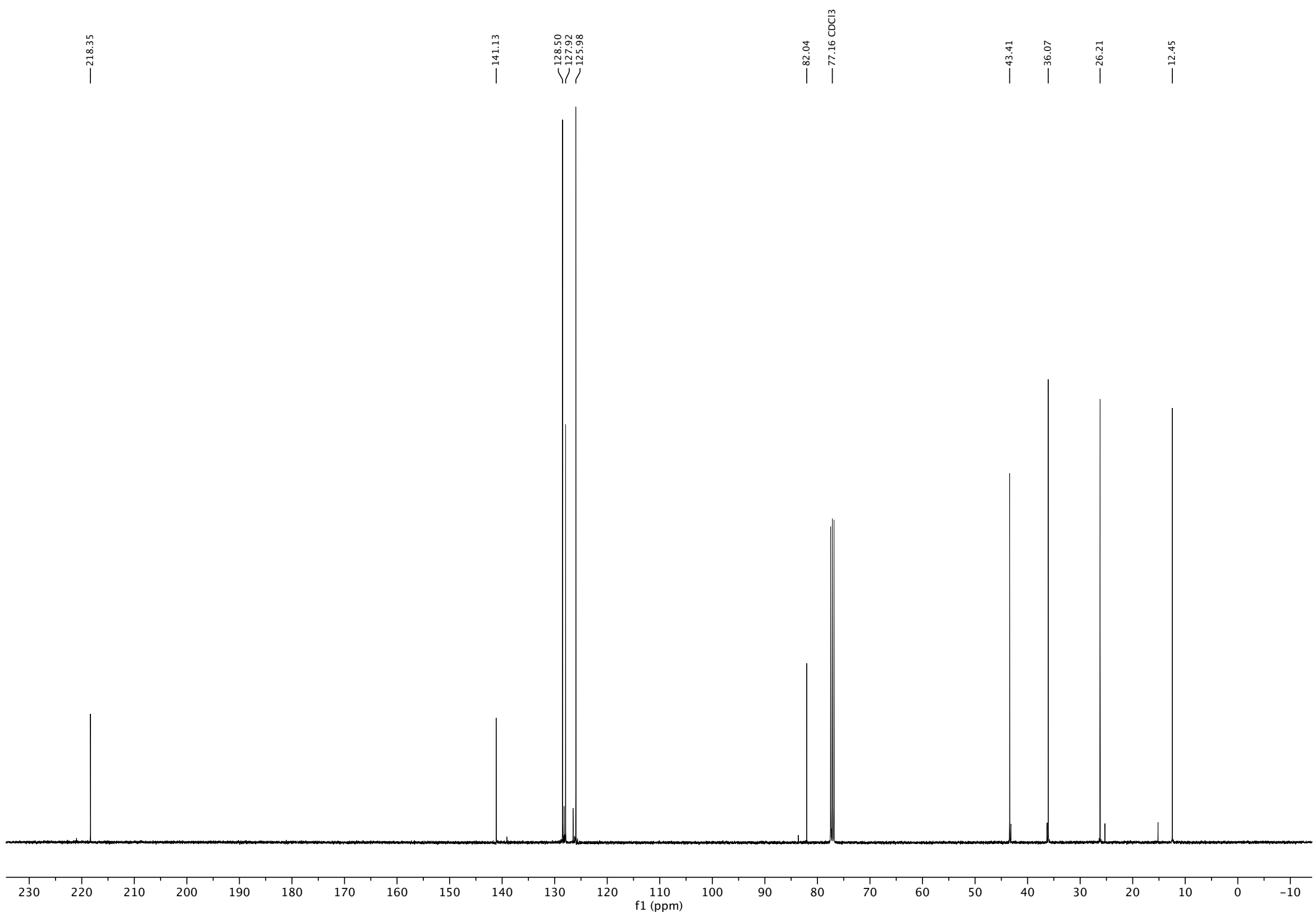

^{13}C NMR (101 MHz, $CDCl_3$) spectrum of compound **17**.

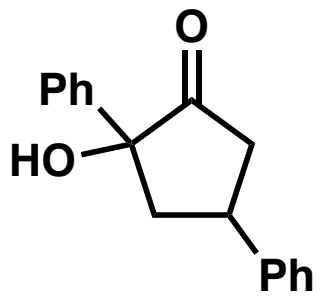

18
diastereomer 1

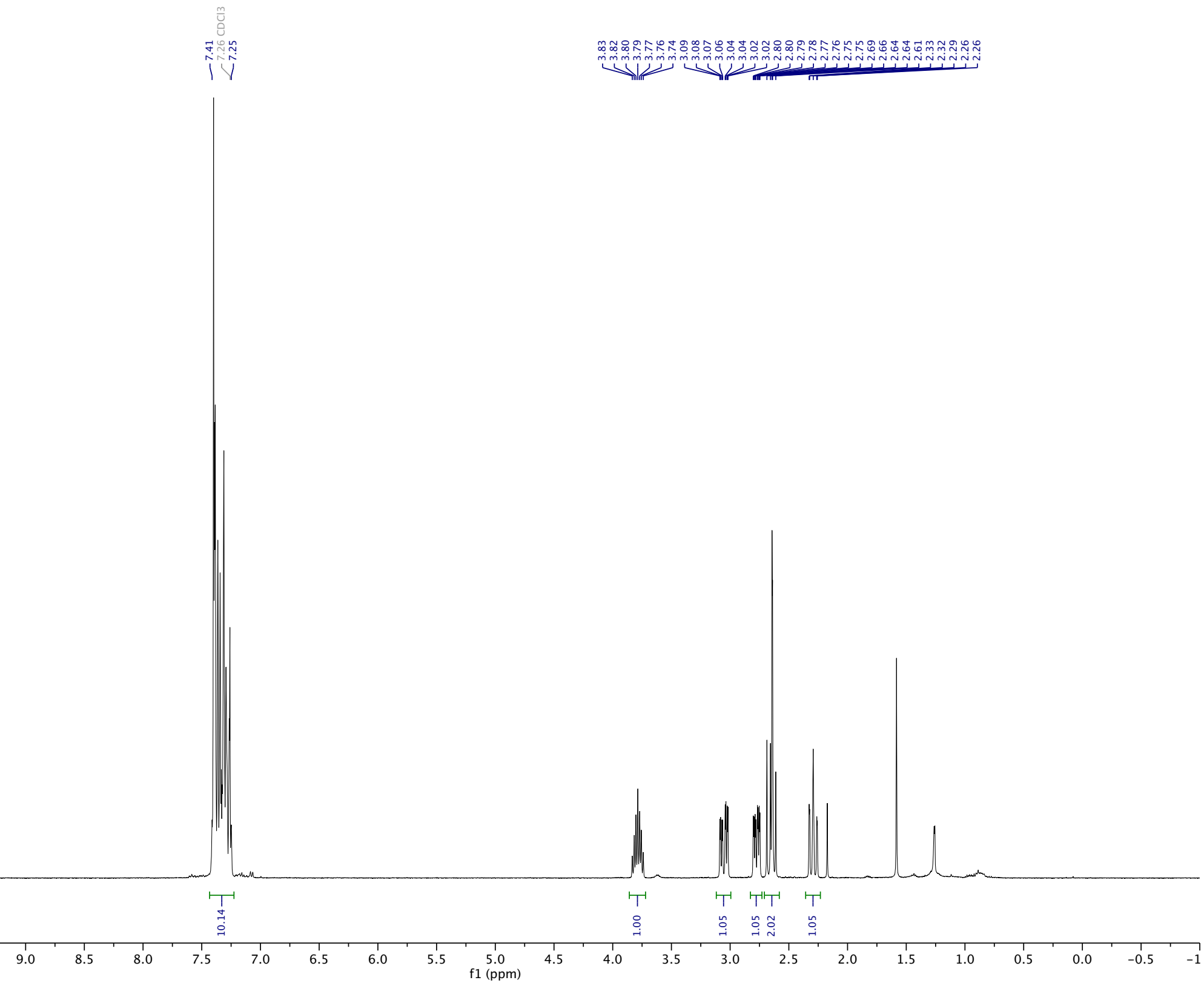

^{1}H NMR (400 MHz, $CDCl_3$) spectrum of compound **18**.

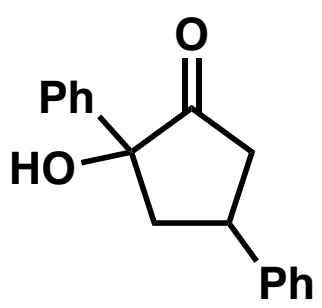

18
diastereomer 1

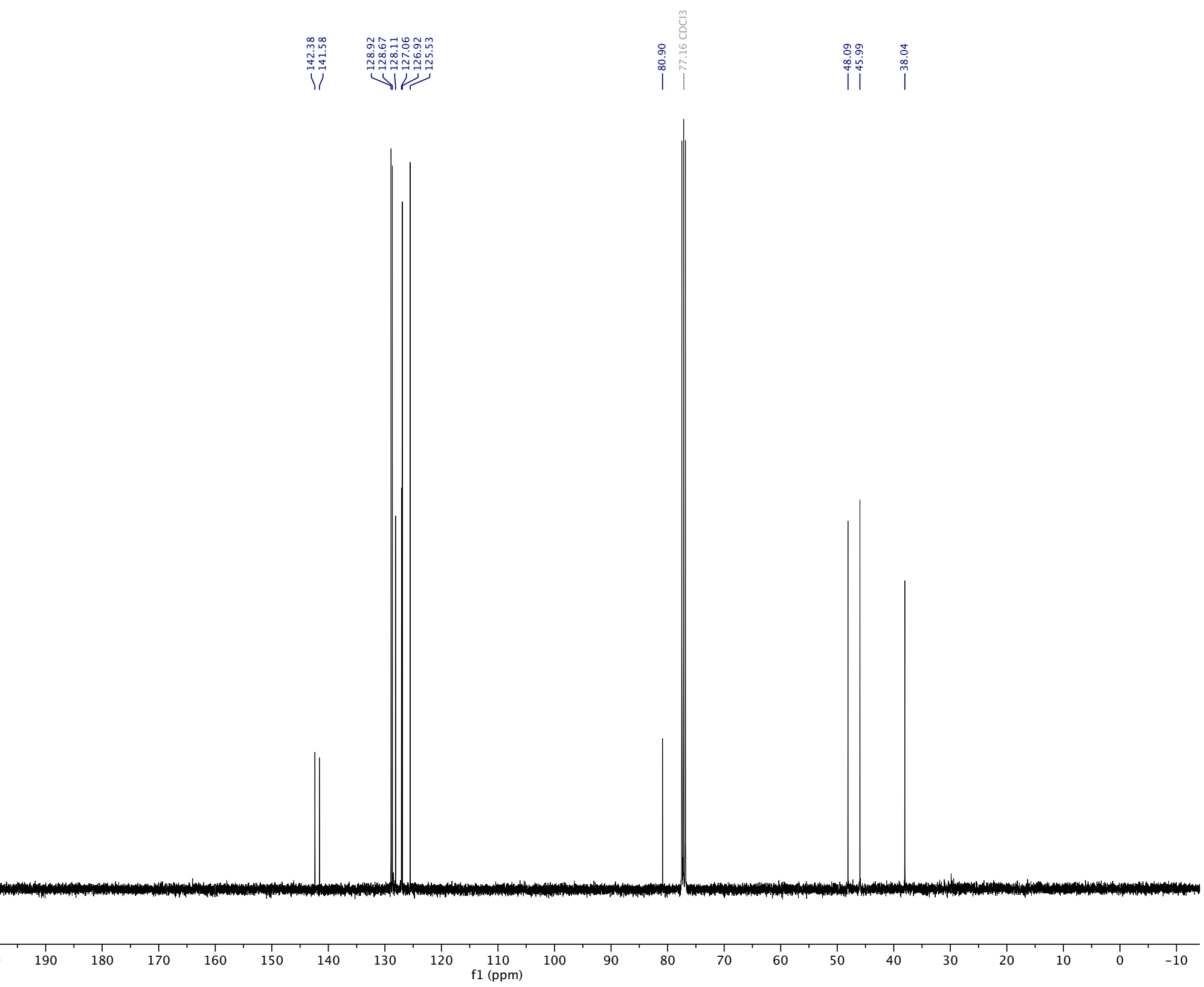

^{13}C NMR (101 MHz, $CDCl_3$) spectrum of compound **18**.

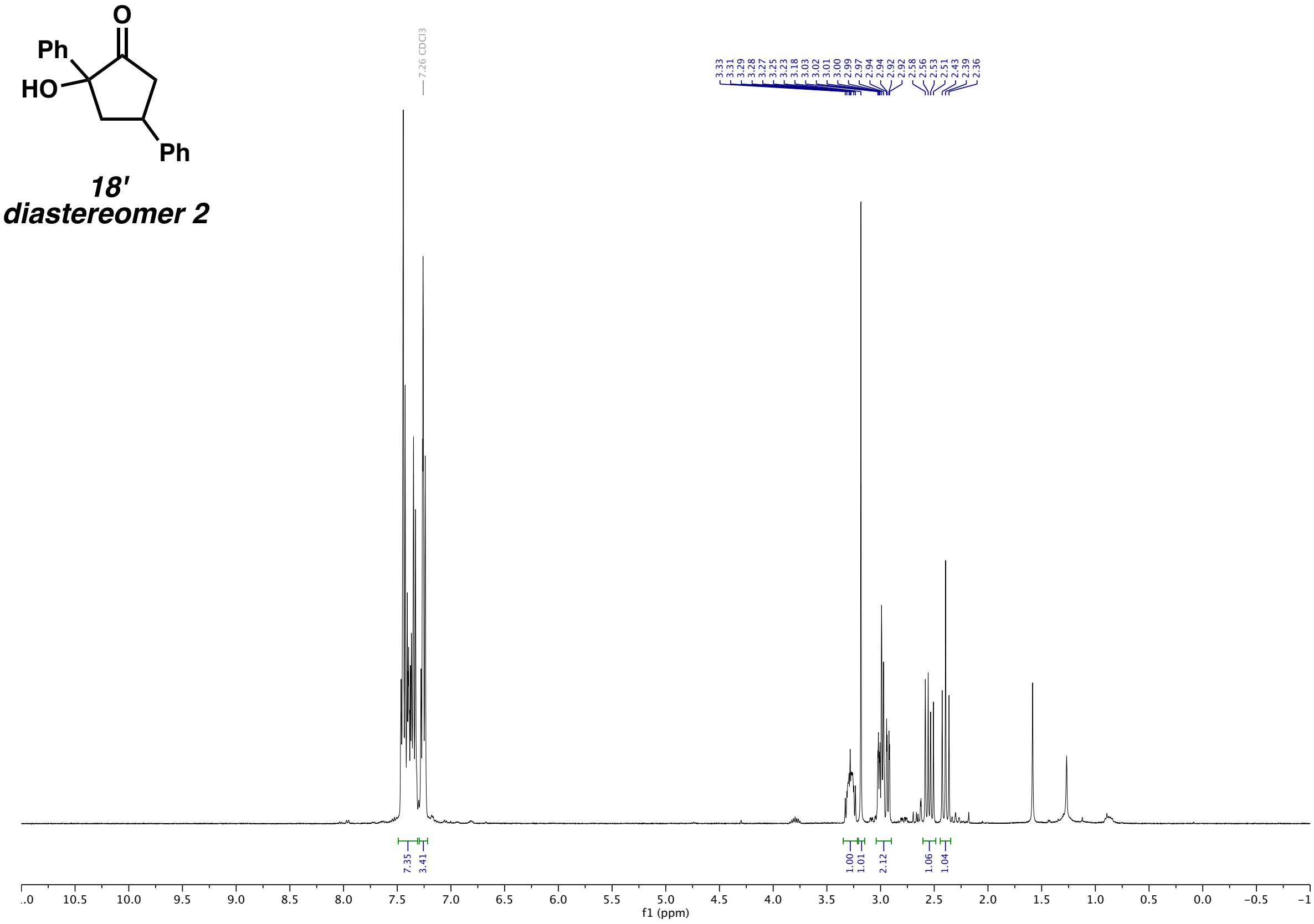

^{1}H NMR (400 MHz, $CDCl_3$) spectrum of compound **18'**.

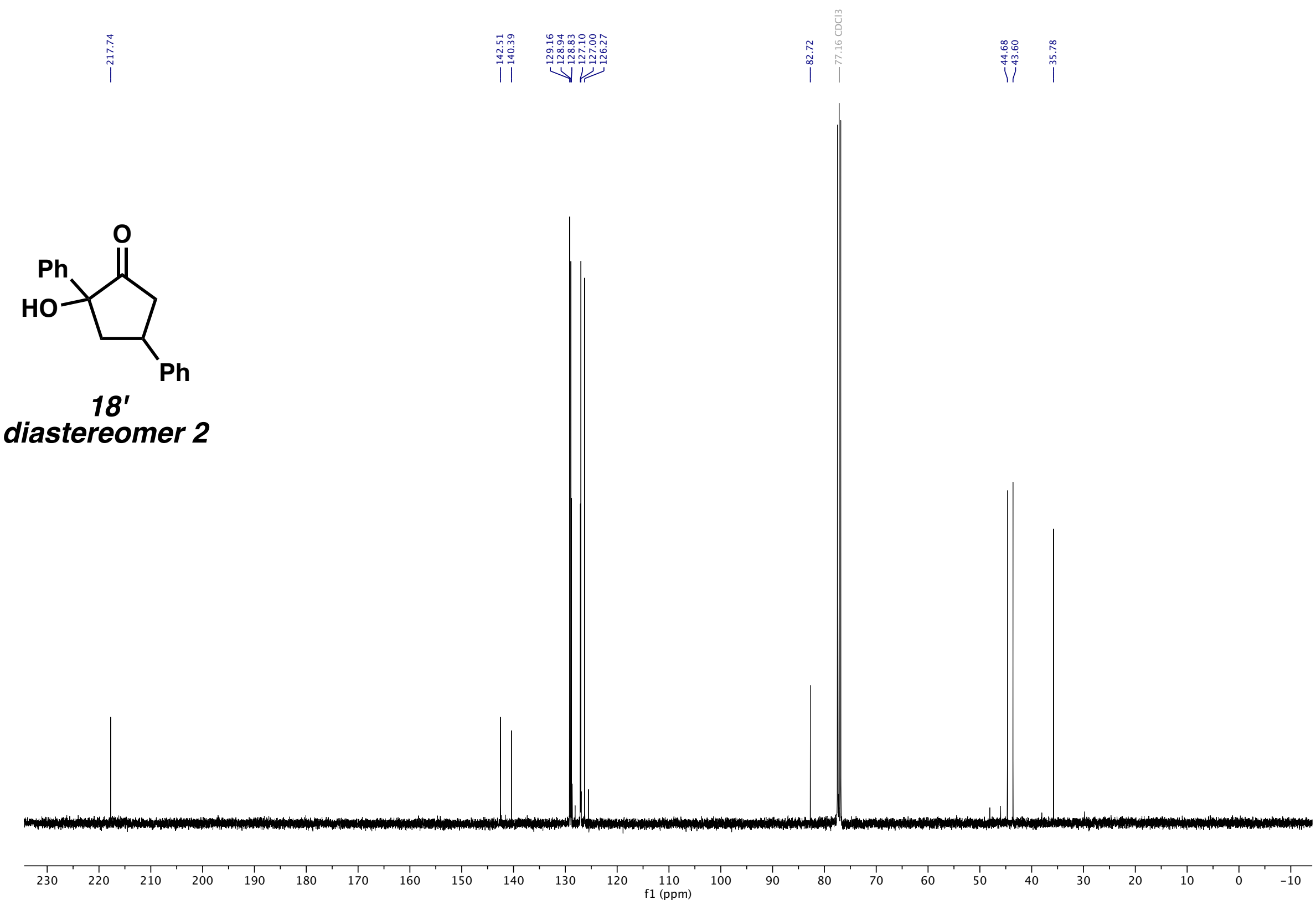

^{13}C NMR (101 MHz, $CDCl_3$) spectrum of compound **18'**.

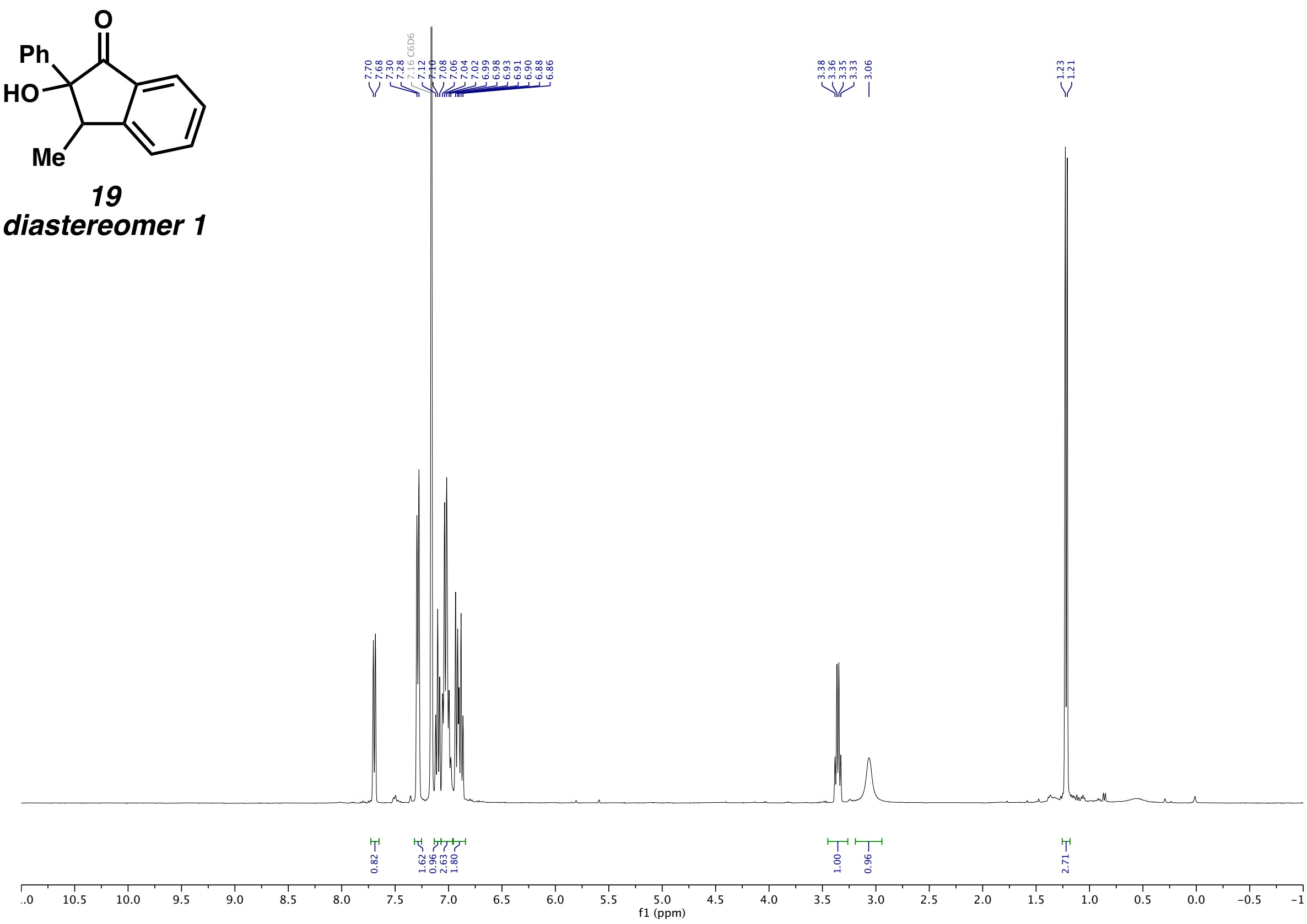

^{1}H NMR (400 MHz, $CDCl_3$) spectrum of compound **19**.

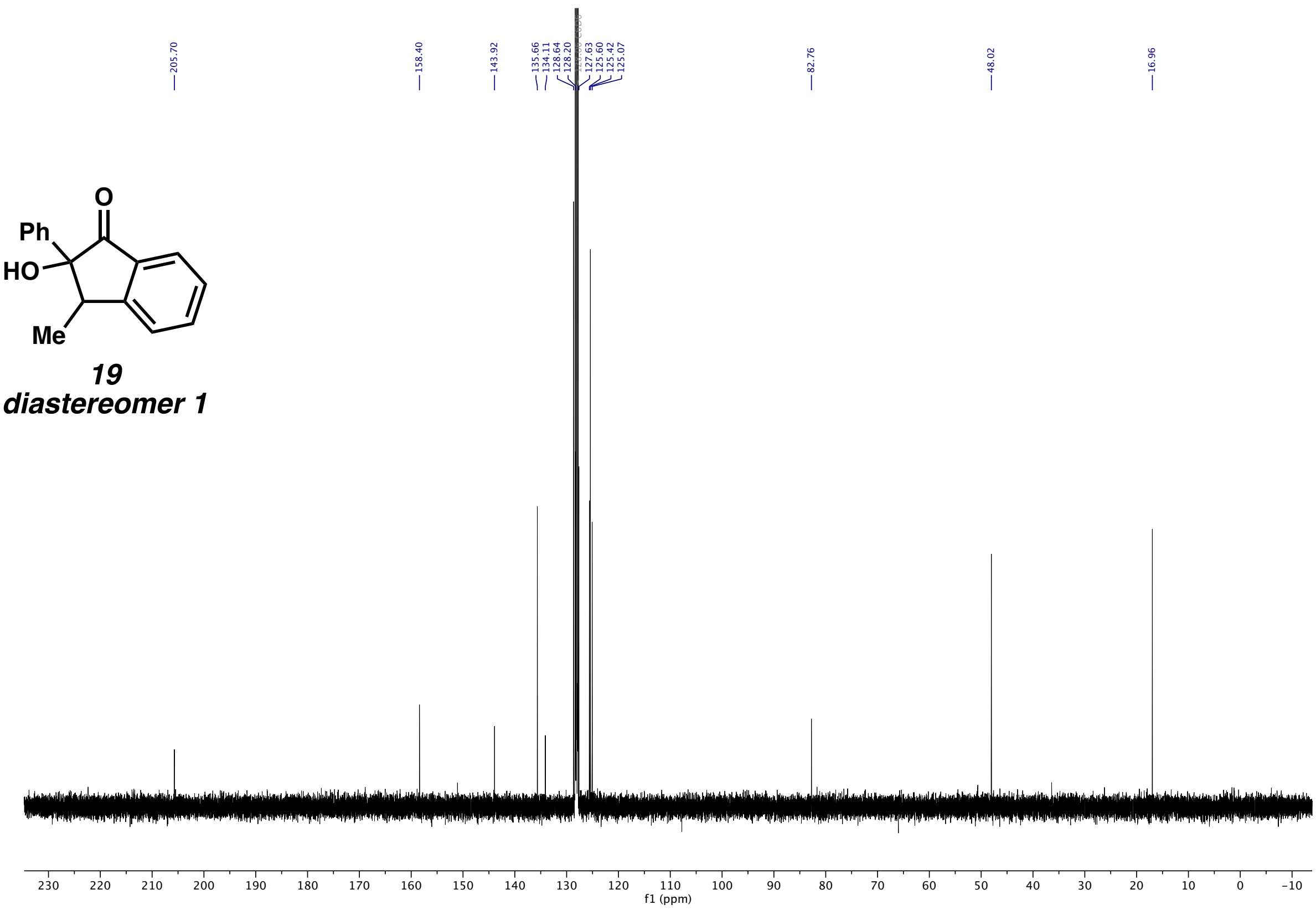

^{13}C NMR (101 MHz, $CDCl_3$) spectrum of compound **19**.

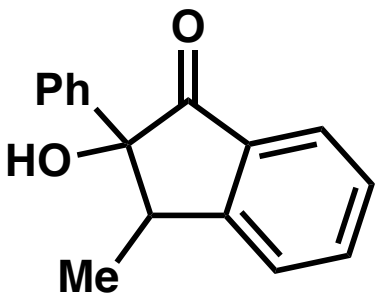

19'
diastereomer 2

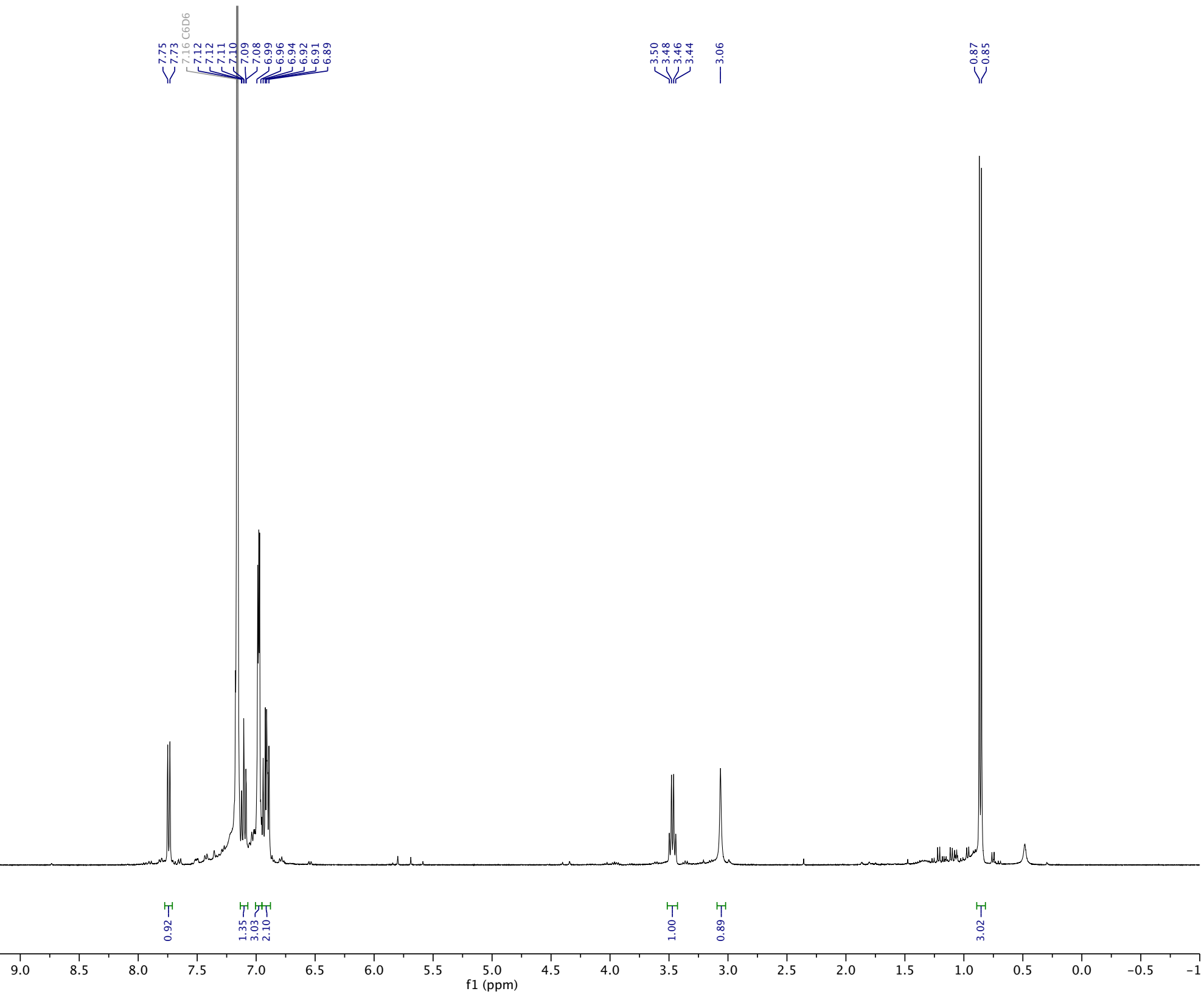

^{1}H NMR (400 MHz, CDCl$_3$) spectrum of compound **19'**.

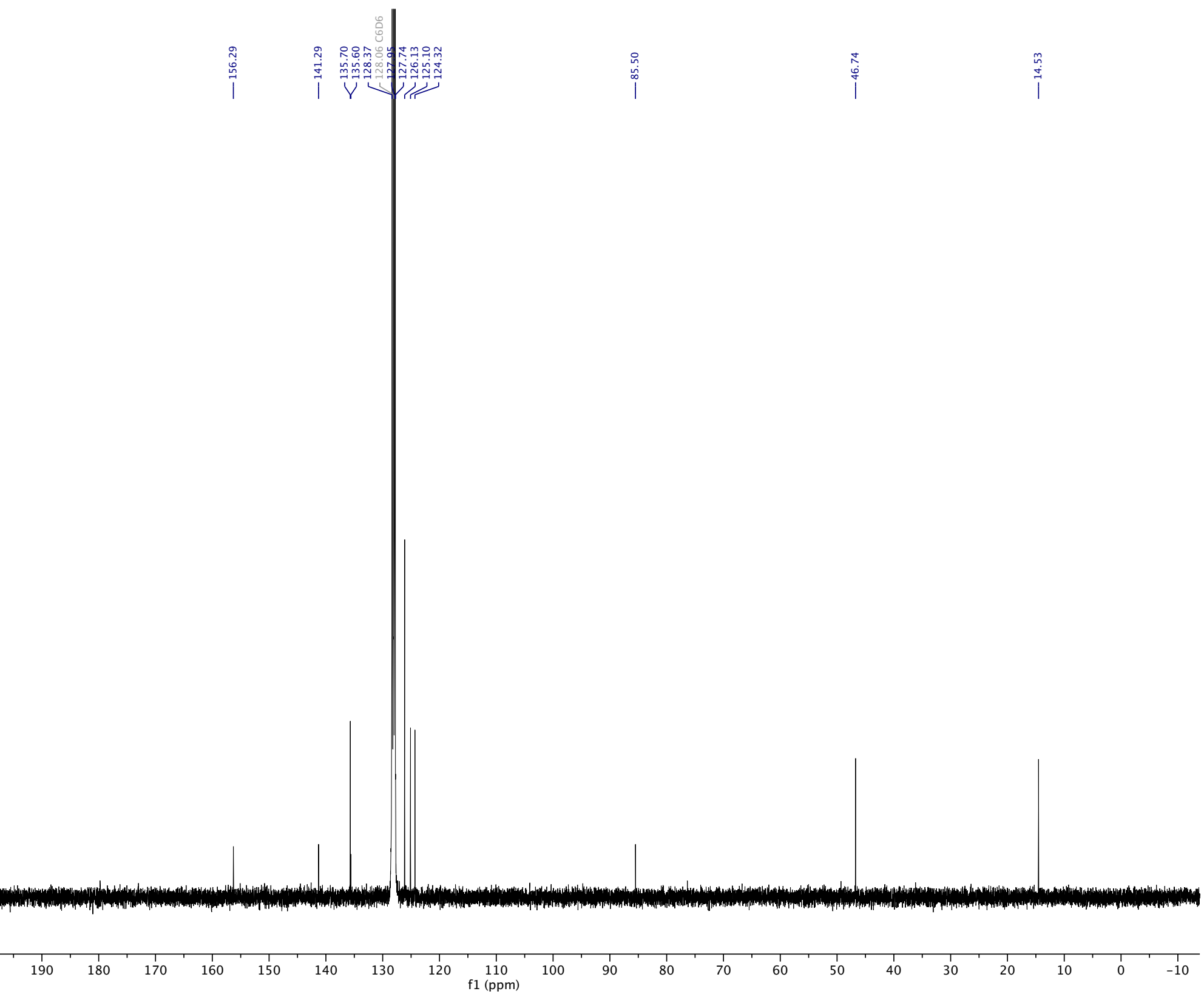

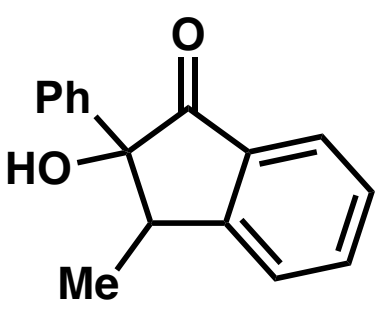

19'
diastereomer 2

^{13}C NMR (101 MHz, CDCl$_3$) spectrum of compound **19'**.

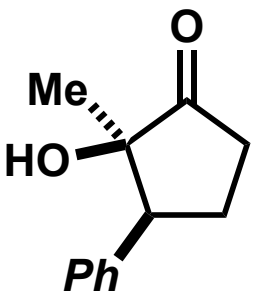

20

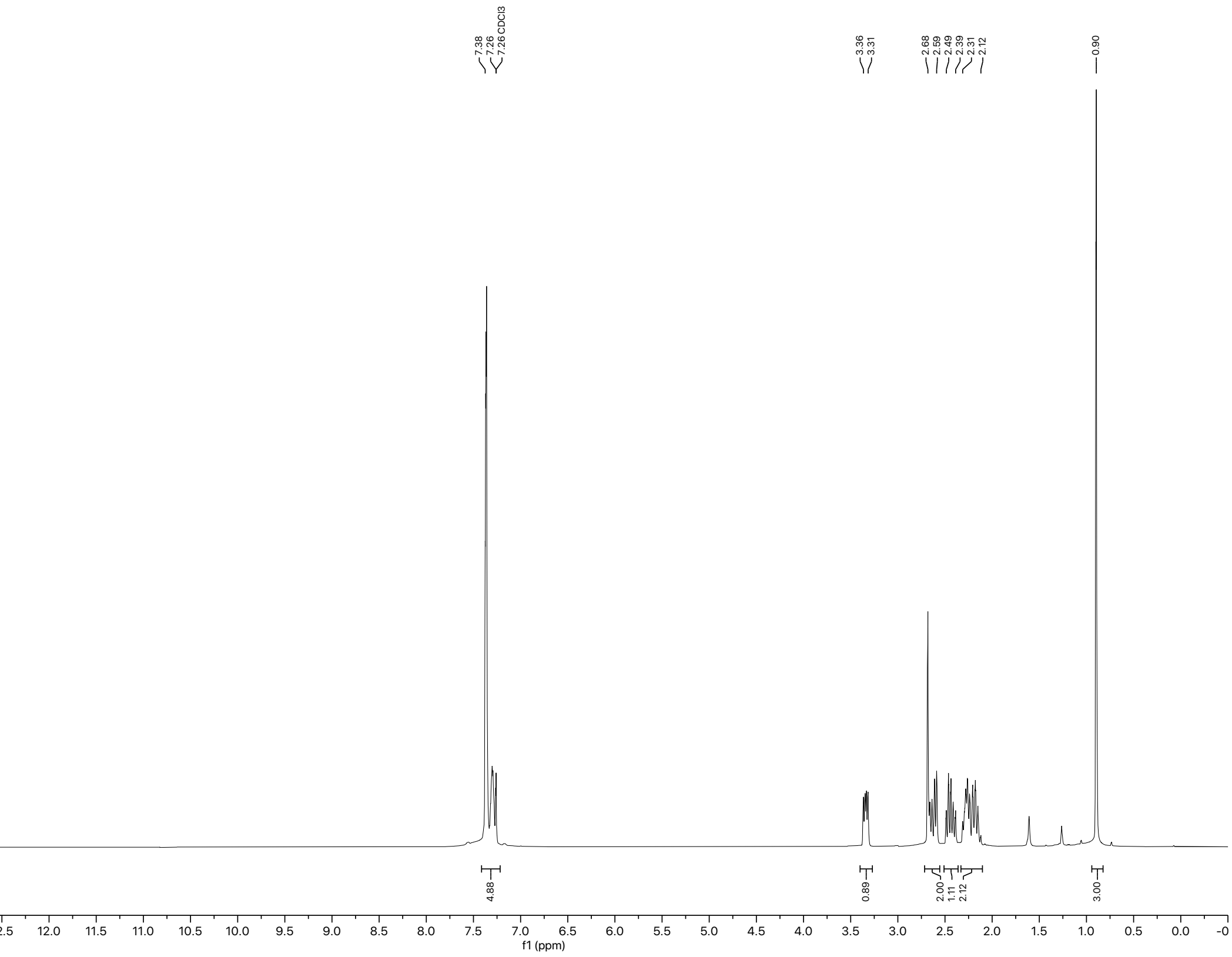

^{1}H NMR (400 MHz, $CDCl_3$) spectrum of compound **20**.

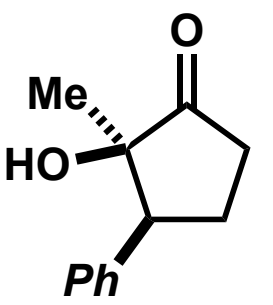

20

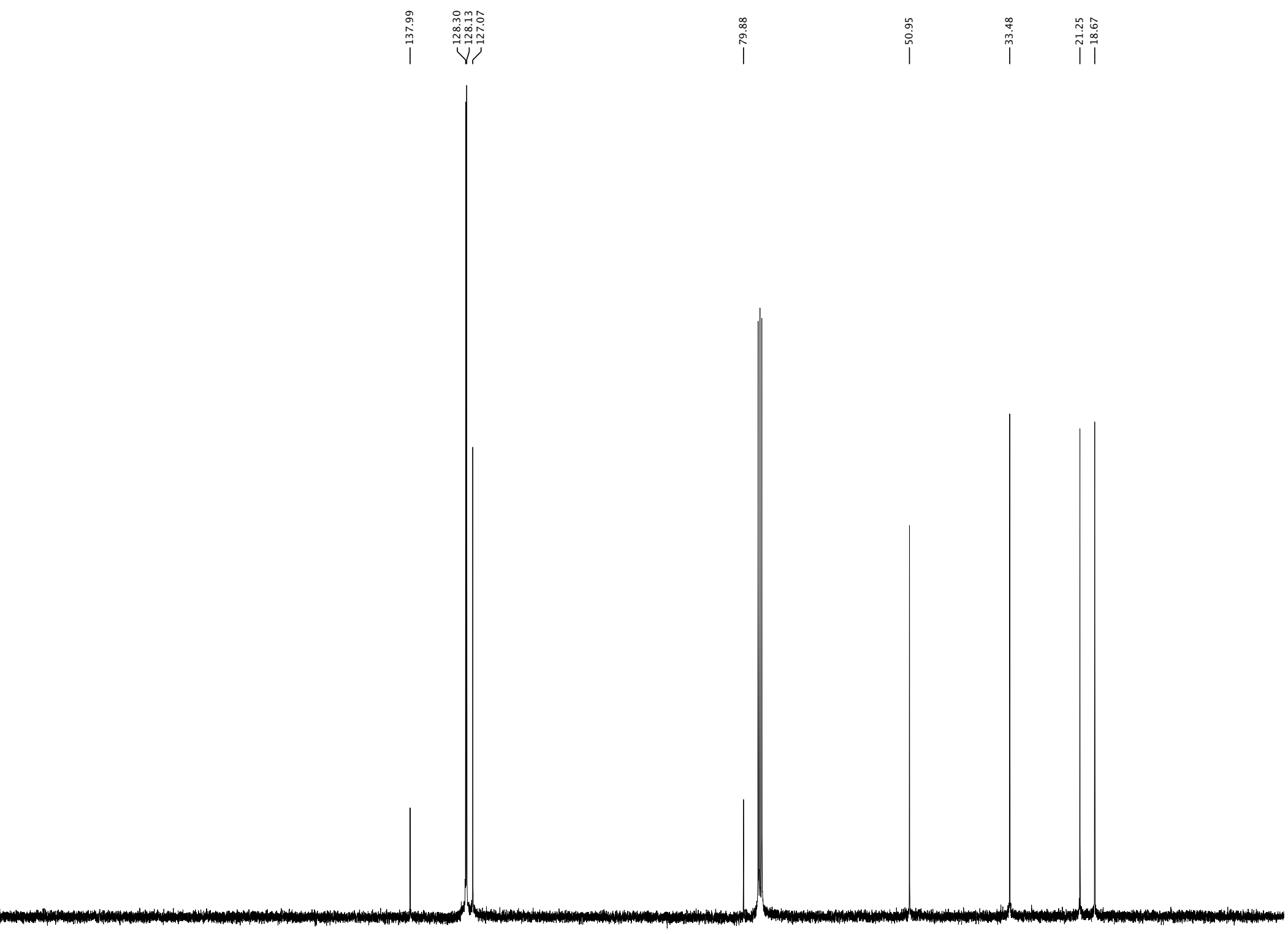

^{13}C NMR (101 MHz, $CDCl_3$) spectrum of compound **20**.

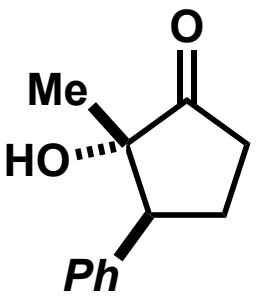

20'

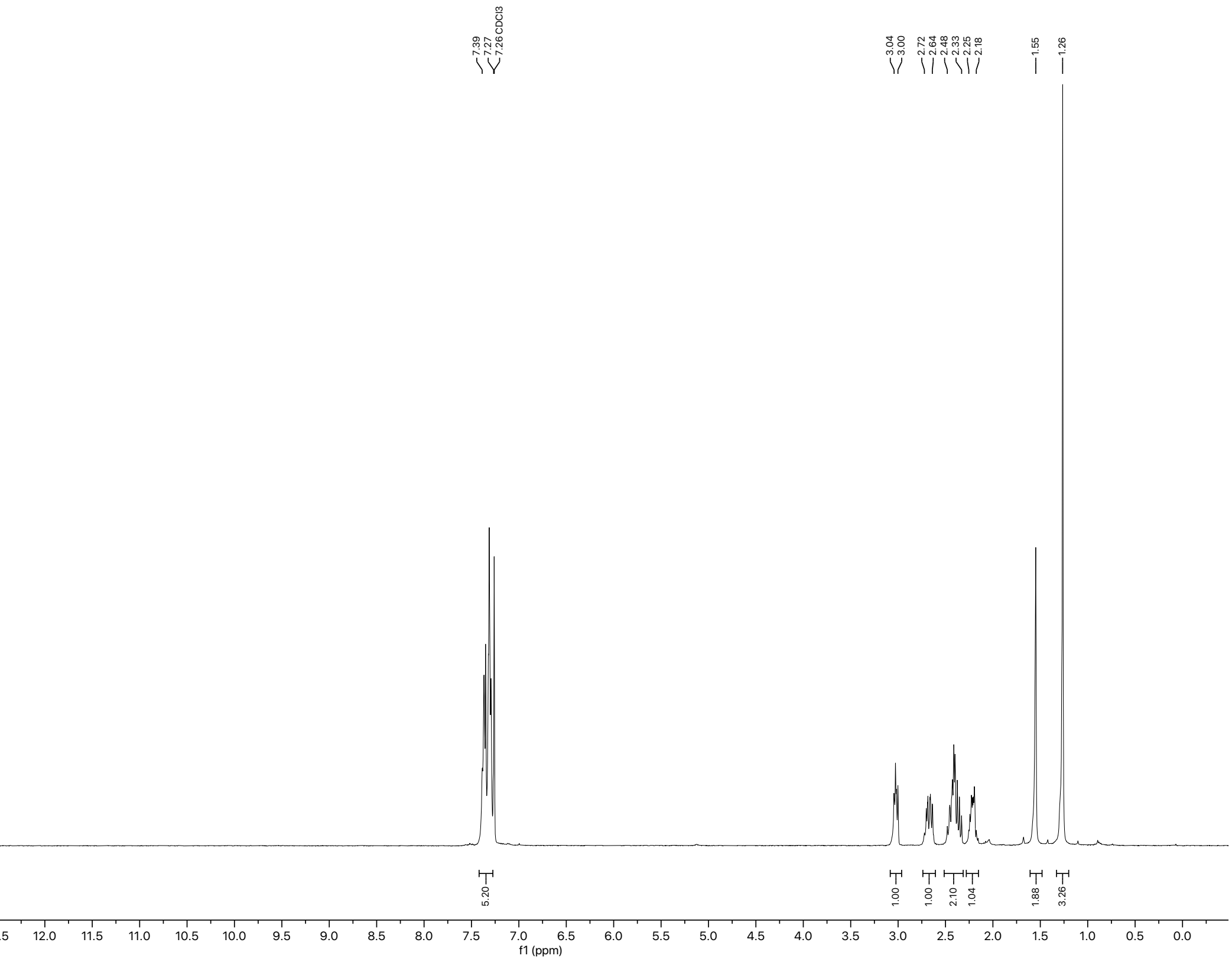

^{1}H NMR (400 MHz, $CDCl_3$) spectrum of compound **20'**.

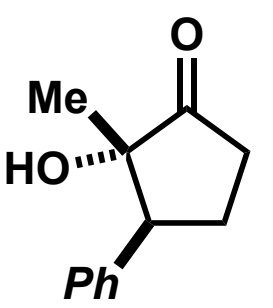

20'

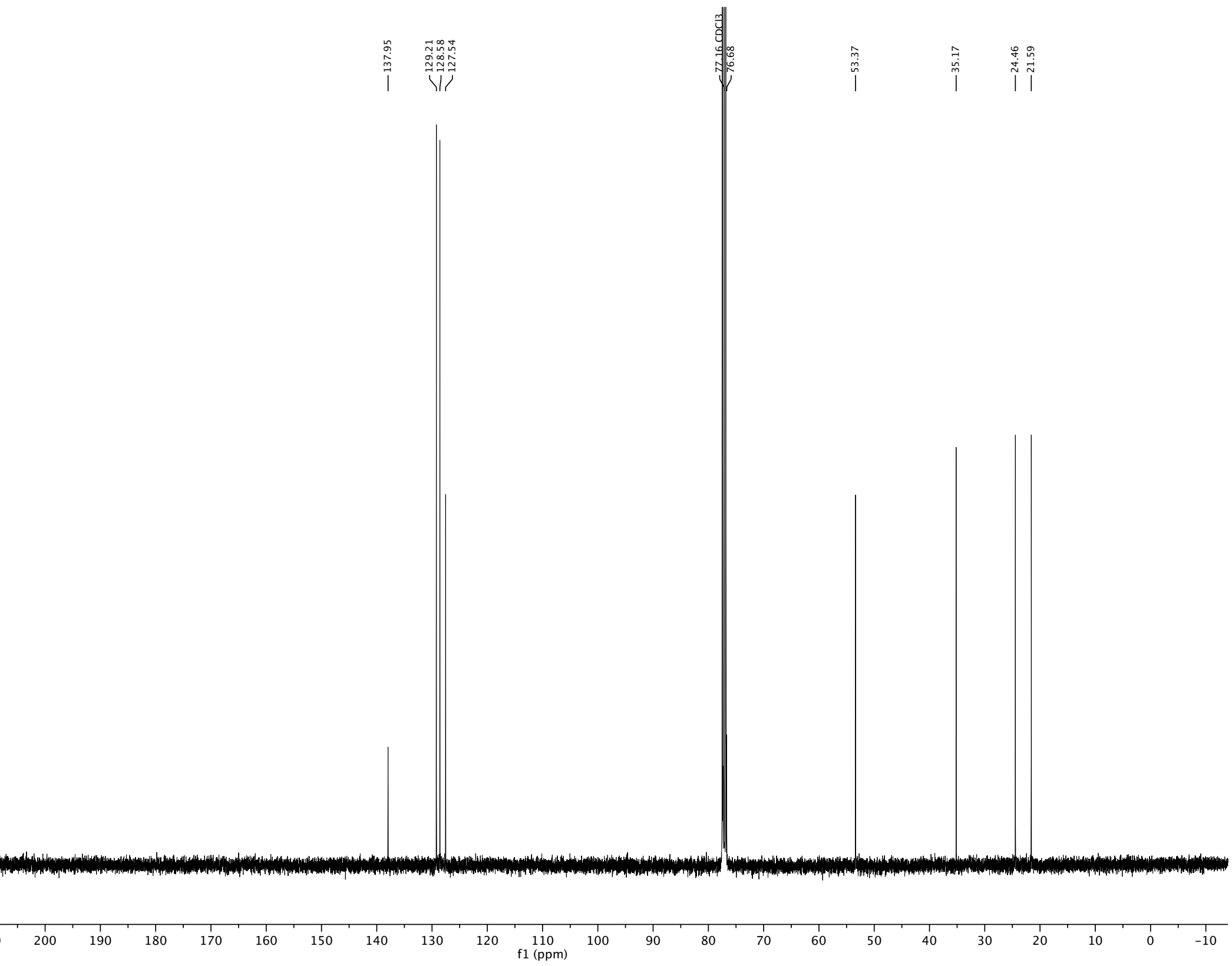

^{13}C NMR (101 MHz, $CDCl_3$) spectrum of compound **20'**.

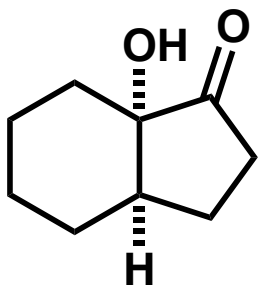

21

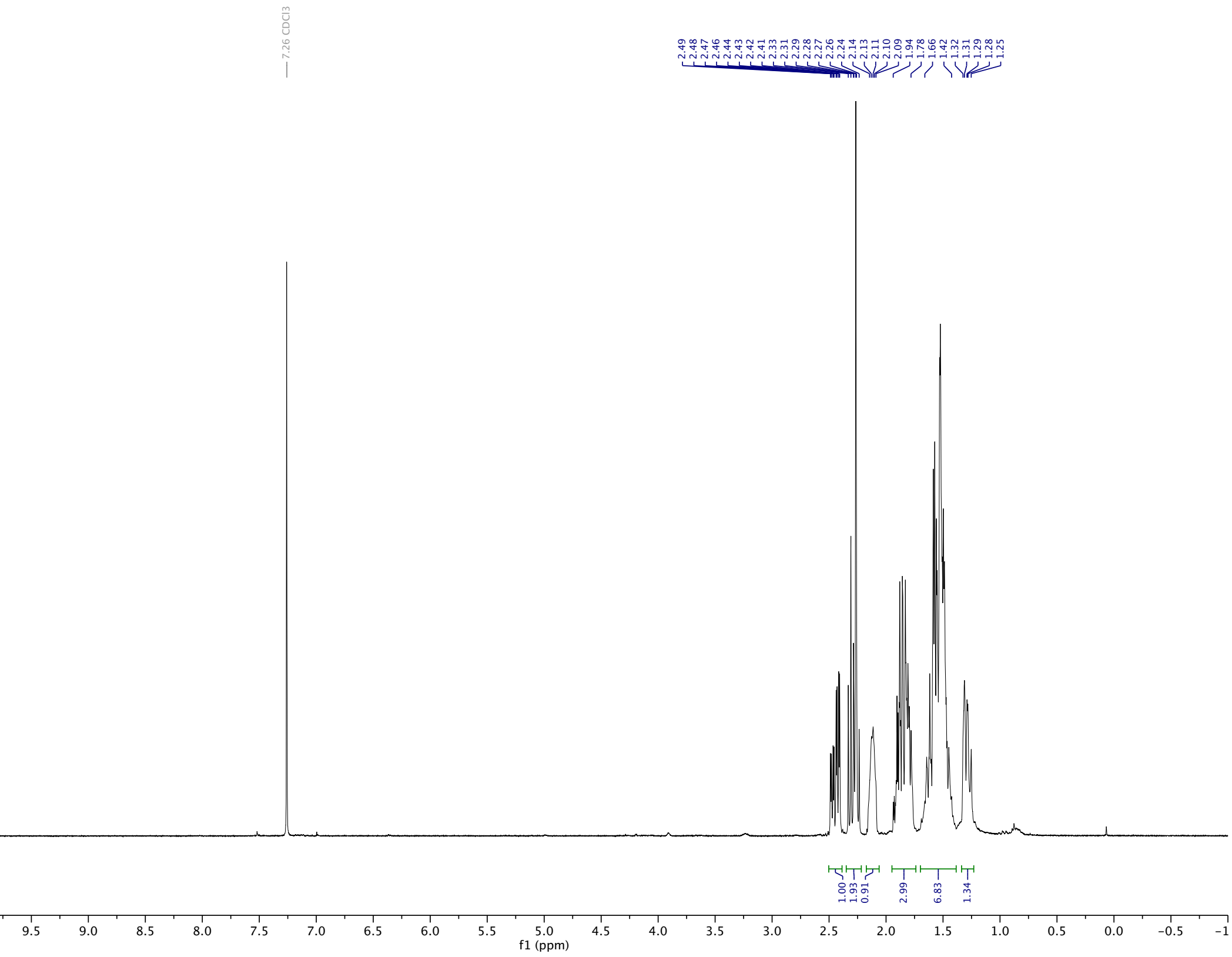

^{1}H NMR (400 MHz, $CDCl_3$) spectrum of compound **21**.

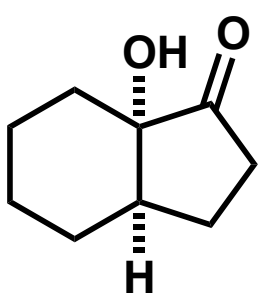

21

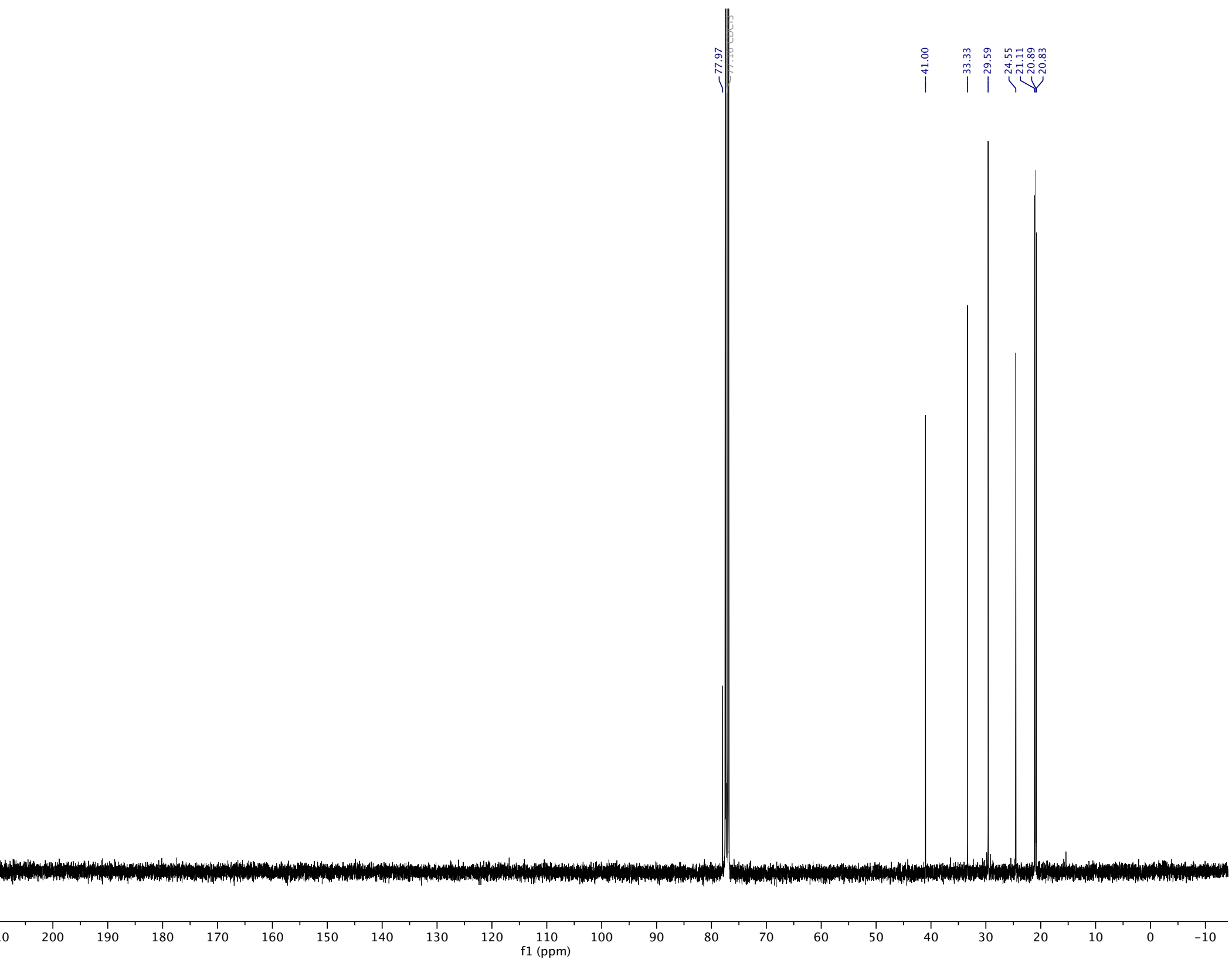

^{13}C NMR (101 MHz, $CDCl_3$) spectrum of compound **21**.

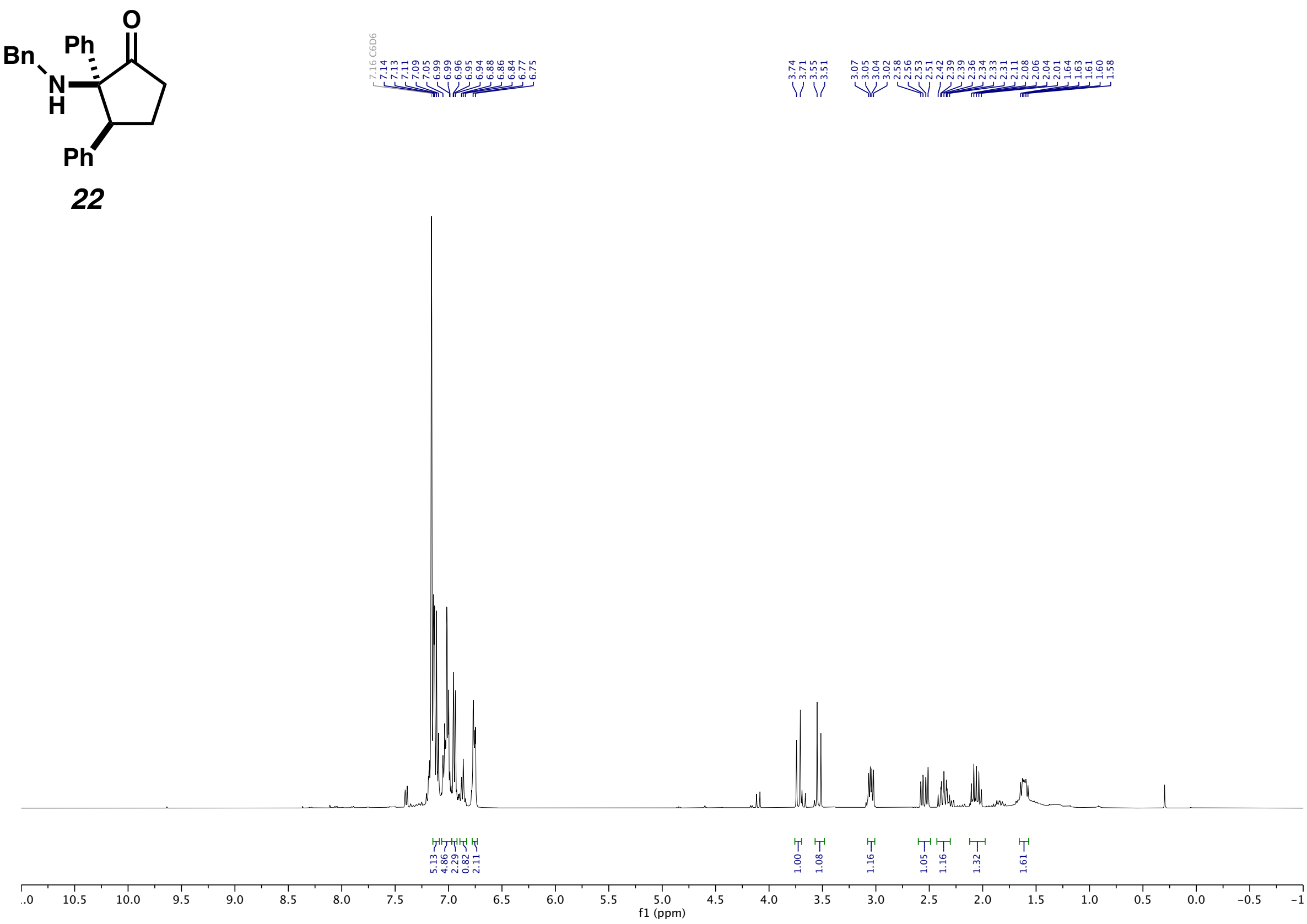

22

^{1}H NMR (400 MHz, C_6D_6) spectrum of compound **22**.

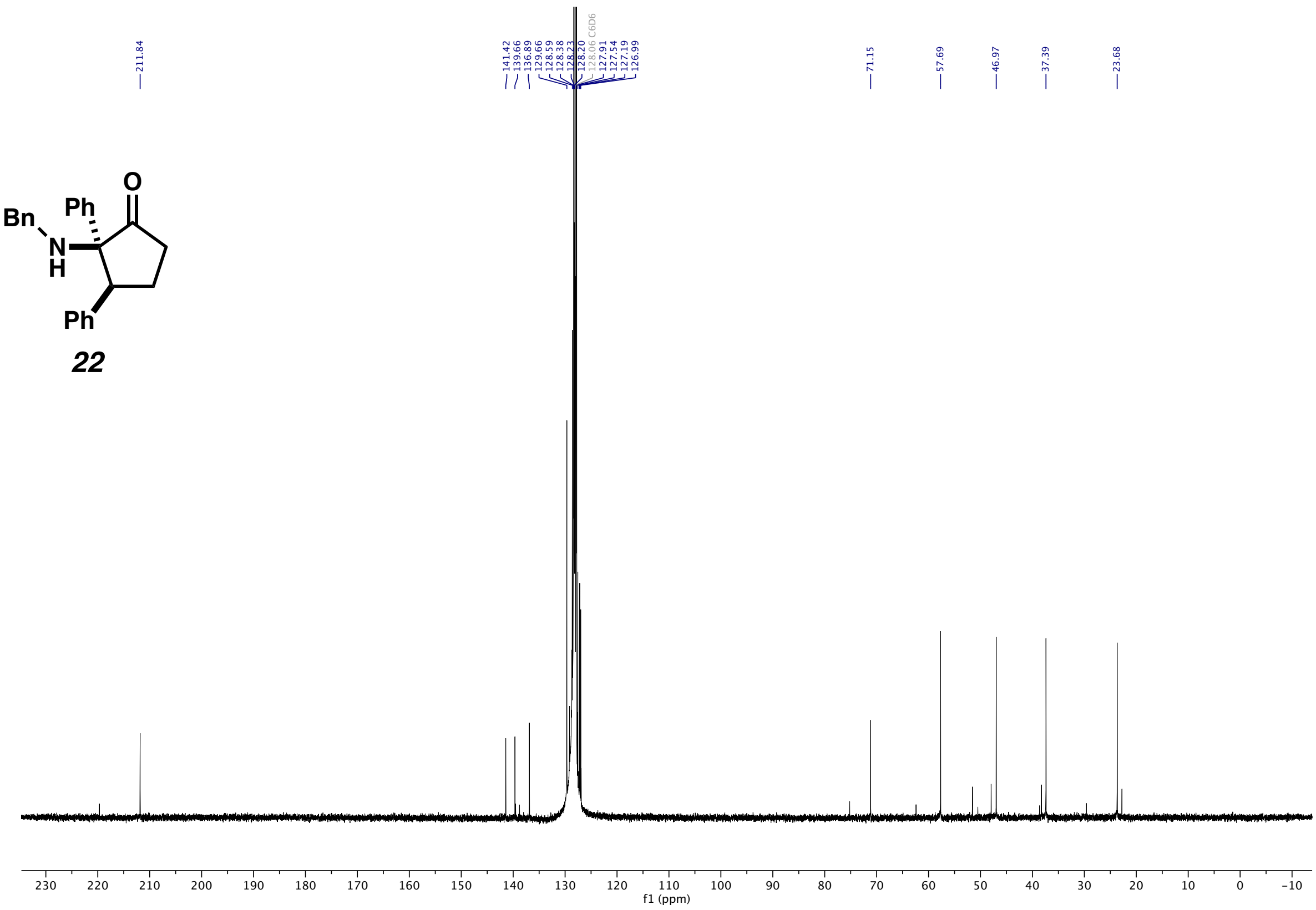

22

^{13}C NMR (101 MHz, C_6D_6) spectrum of compound **22**.

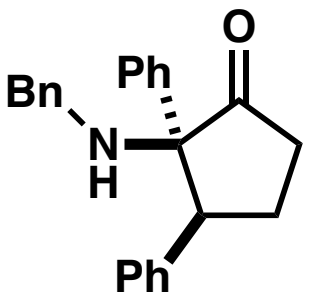

22

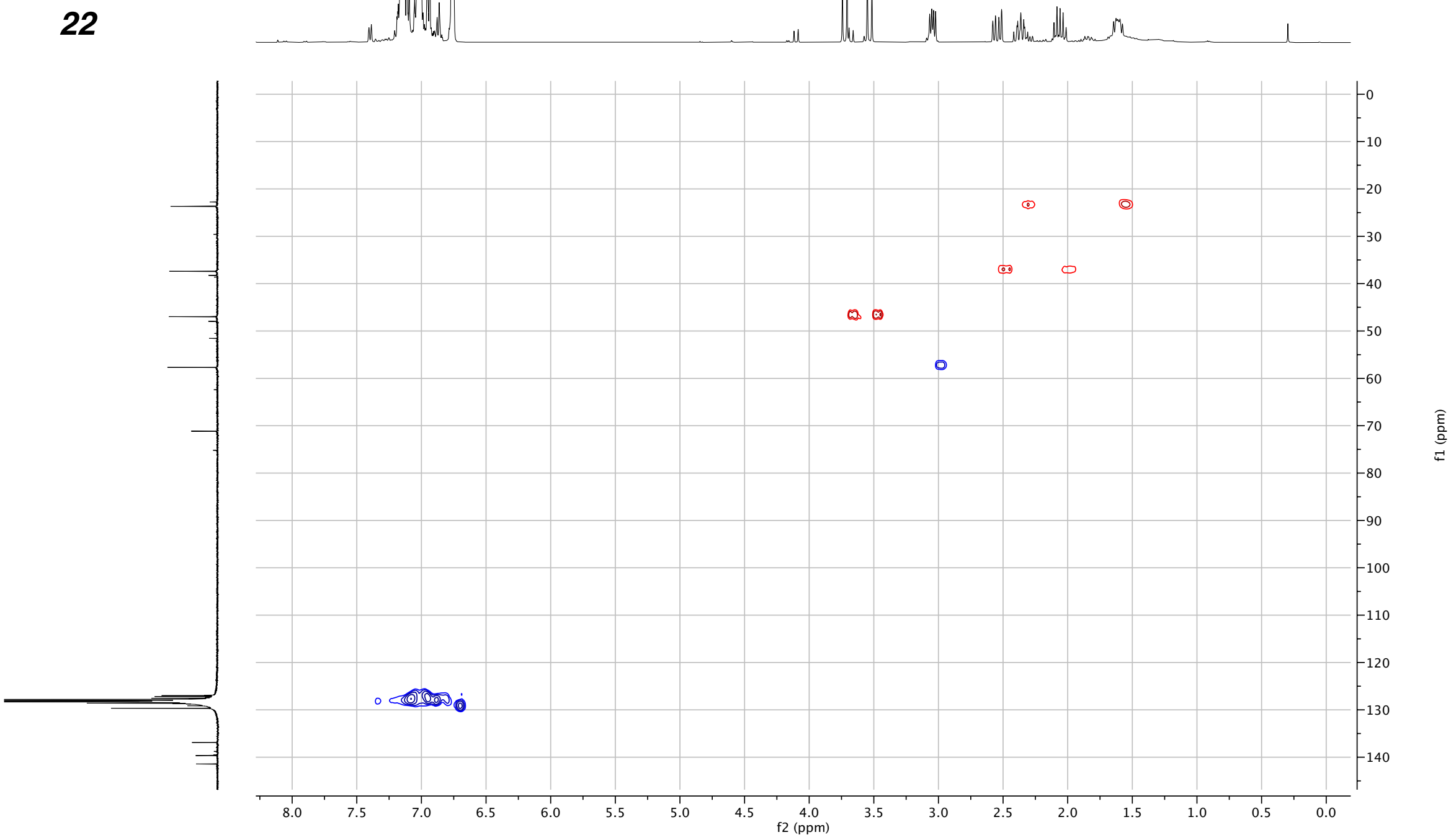

HSQC spectrum of compound **22**.

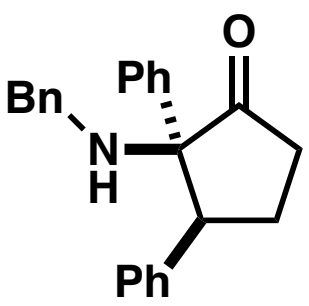

22

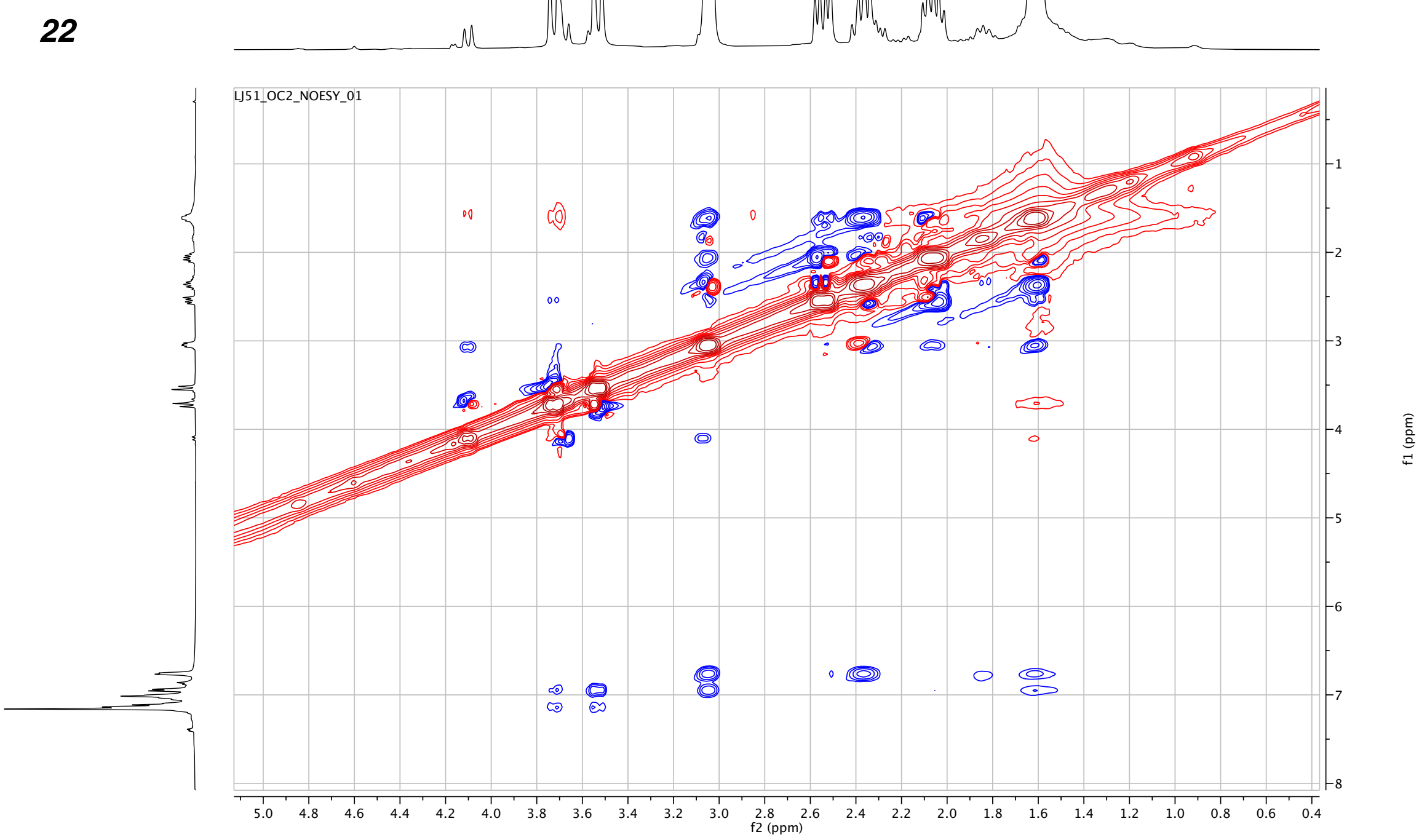

NOESY spectrum of compound **22**.

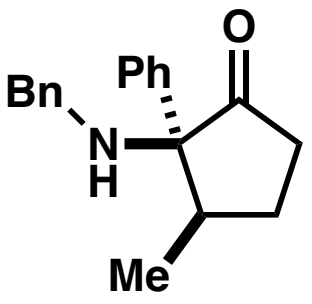

23

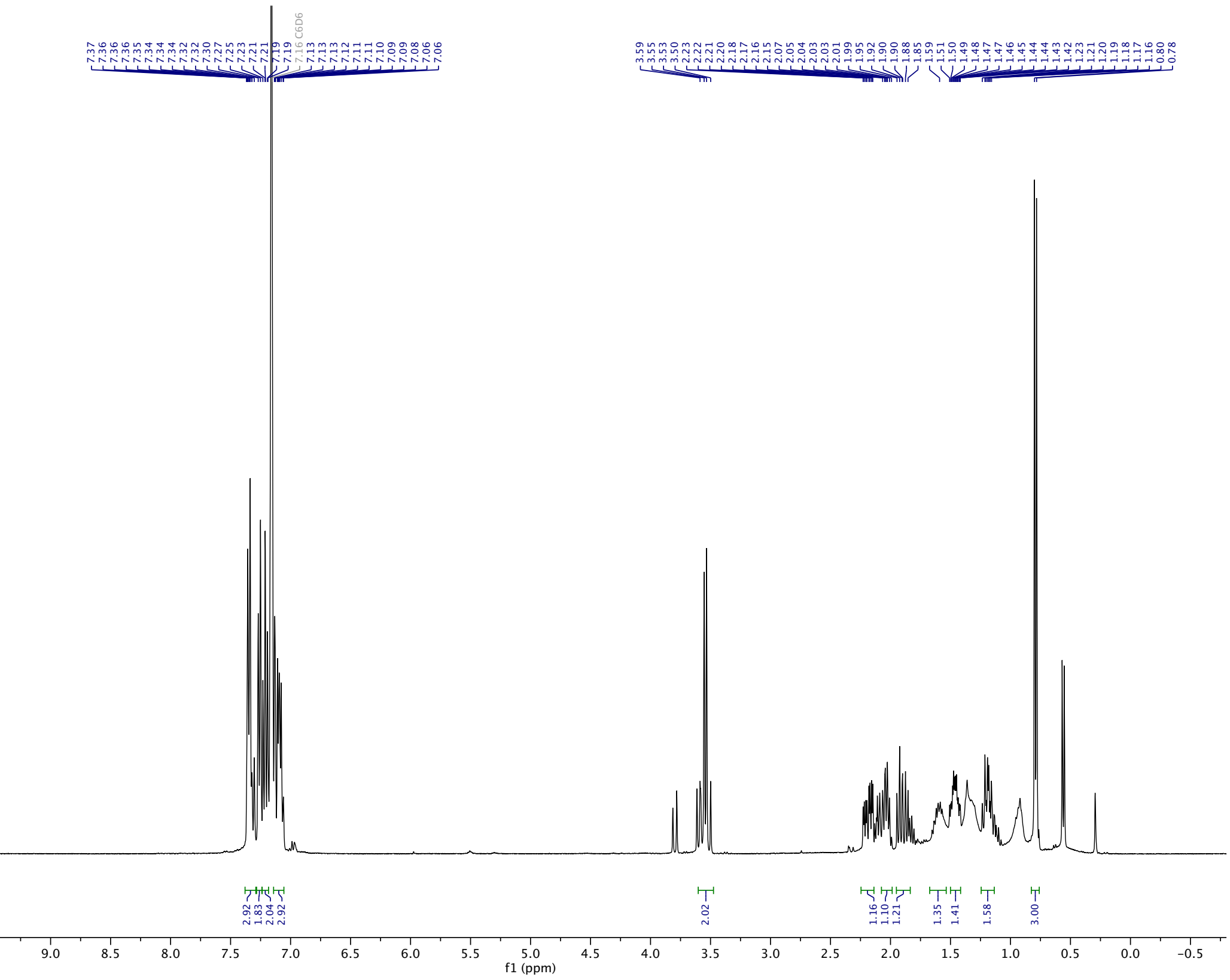

^{1}H NMR (400 MHz, C_6D_6) spectrum of compound **23**.

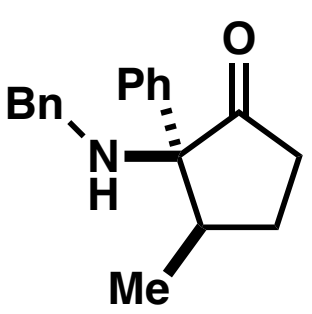

23

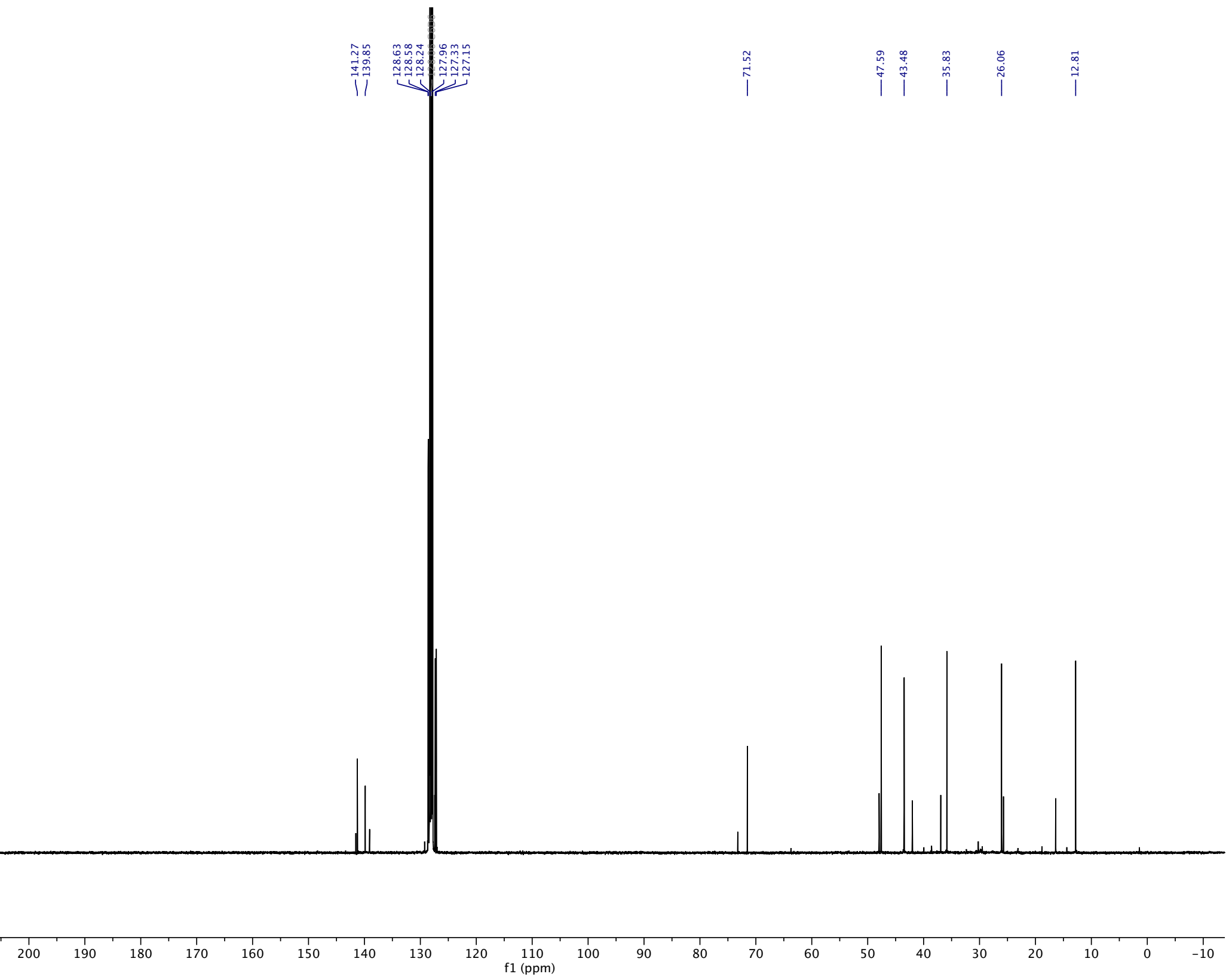

^{13}C NMR (101 MHz, C_6D_6) spectrum of compound **23**.

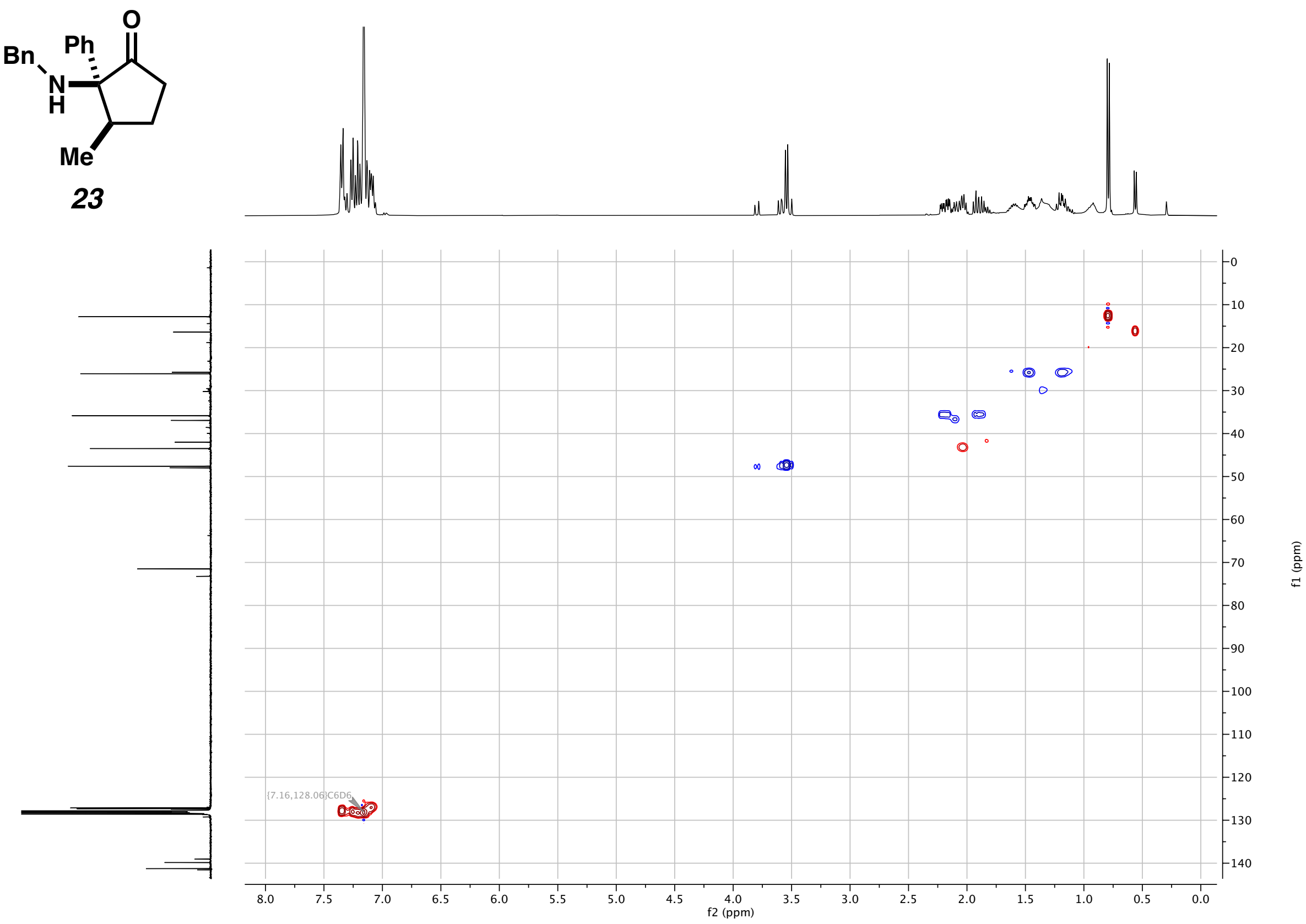

HSQC spectrum of compound **23**.

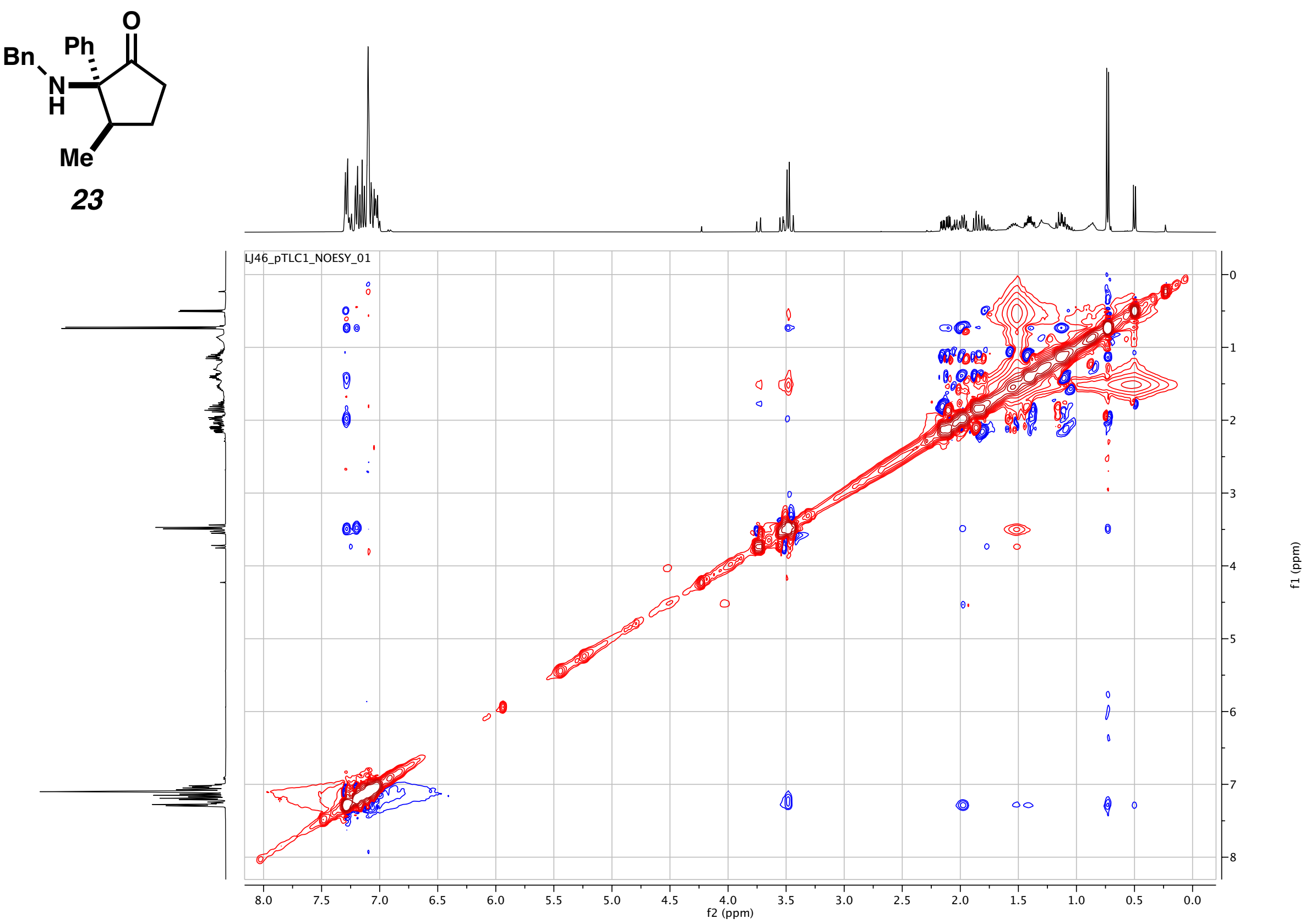

NOESY spectrum of compound **23**.

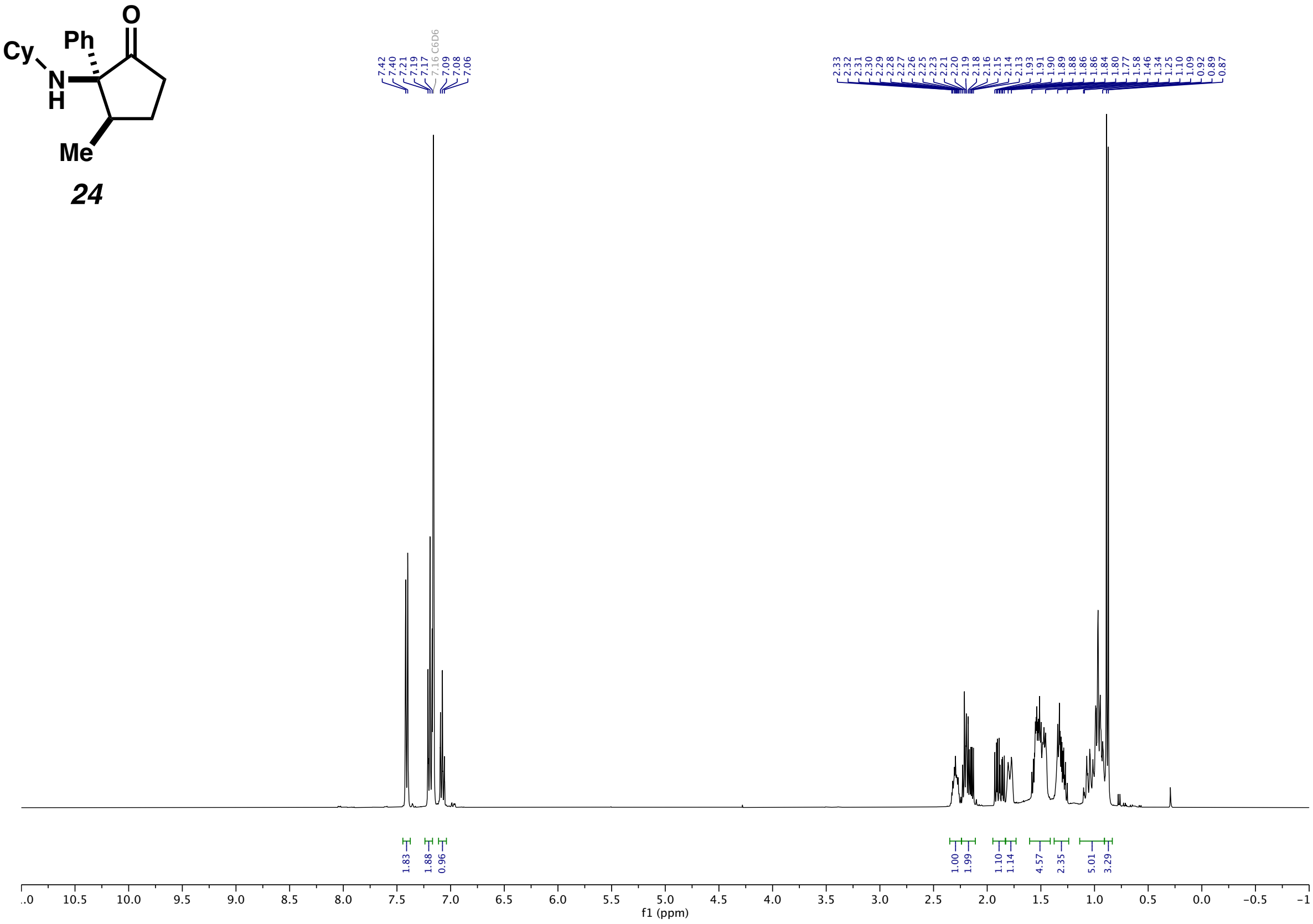

^{1}H NMR (400 MHz, C_6D_6) spectrum of compound **24**.

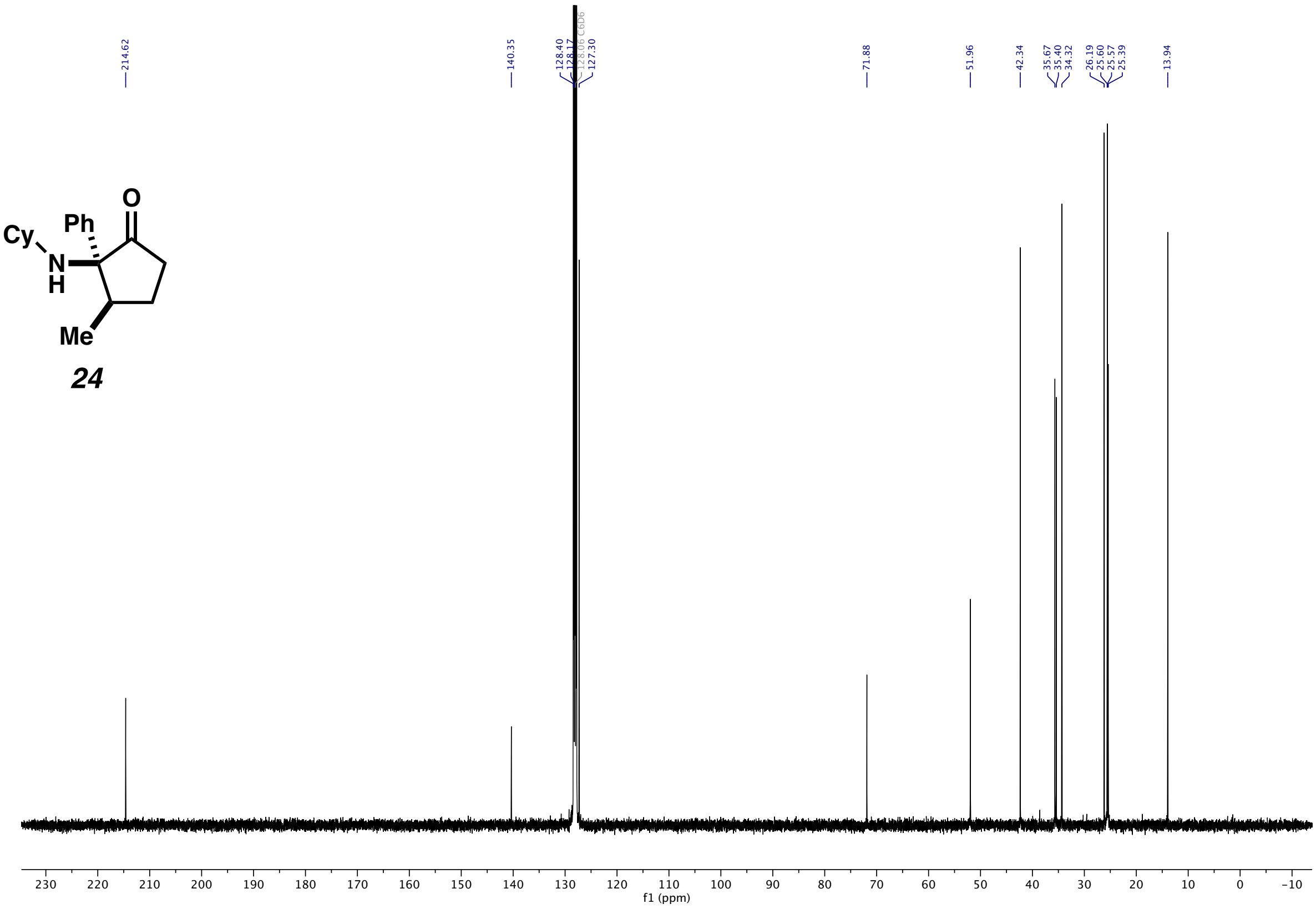

^{13}C NMR (101 MHz, C_6D_6) spectrum of compound **24**.

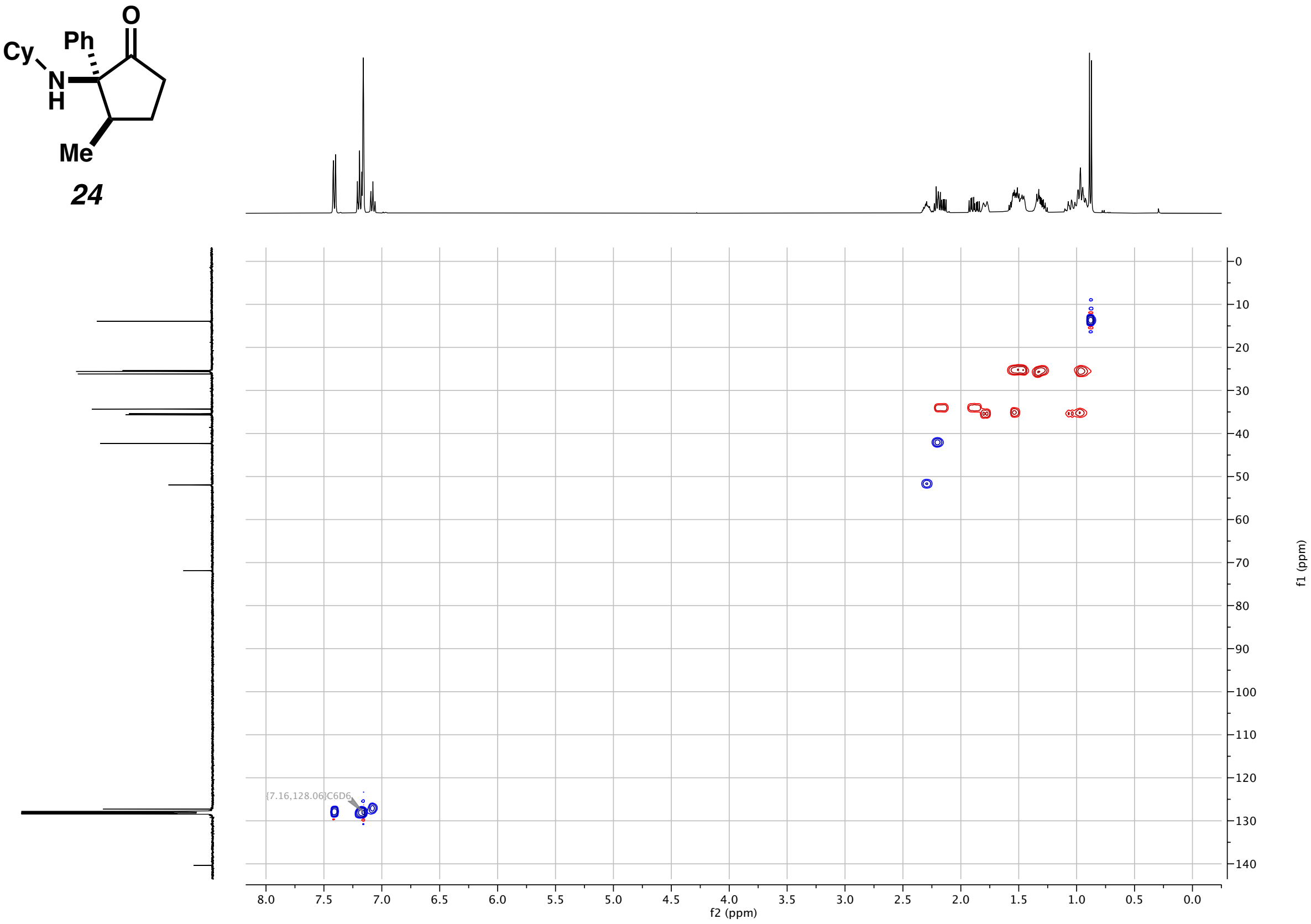

HSQC spectrum of compound **24**.

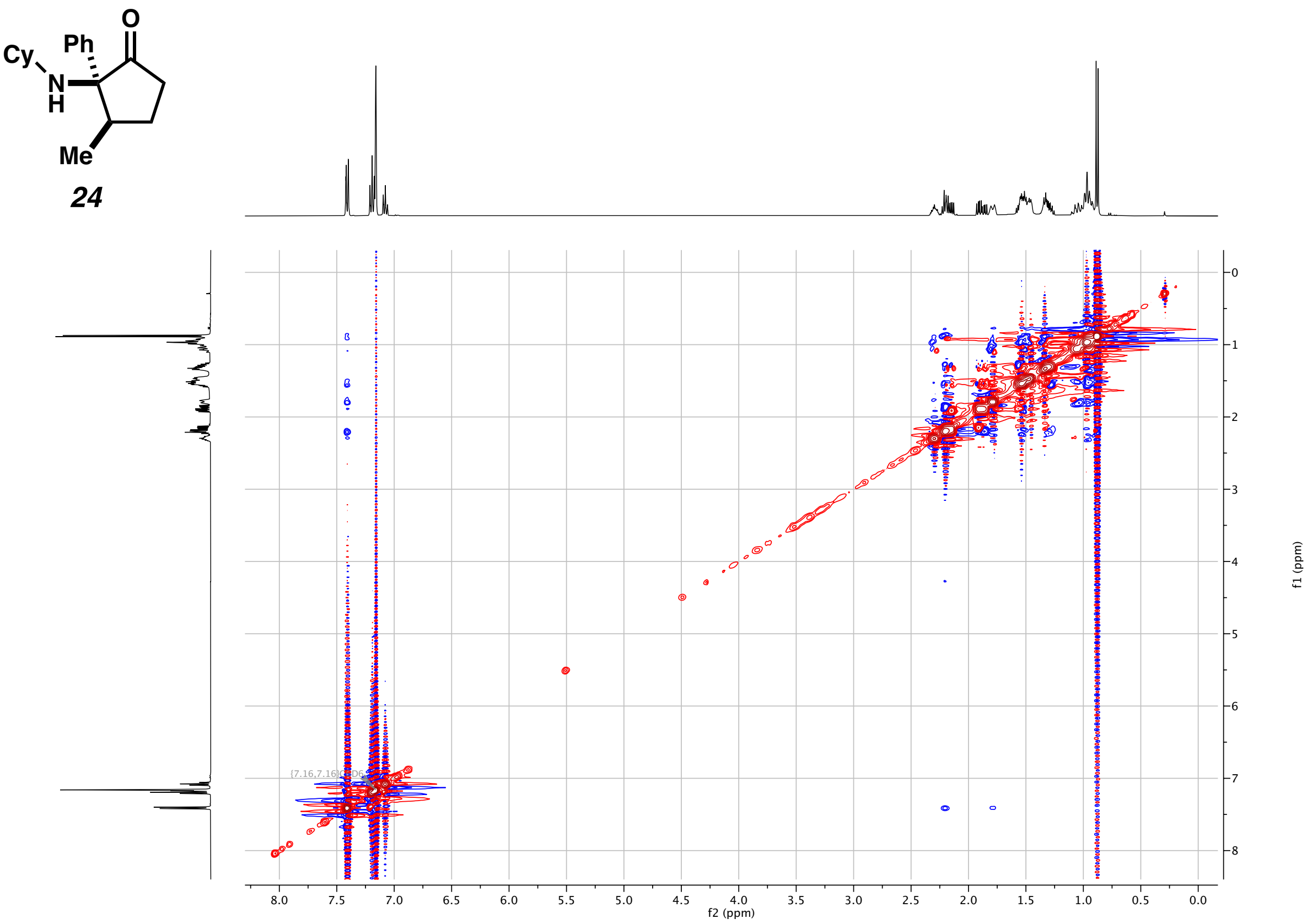

NOESY spectrum of compound **24**.

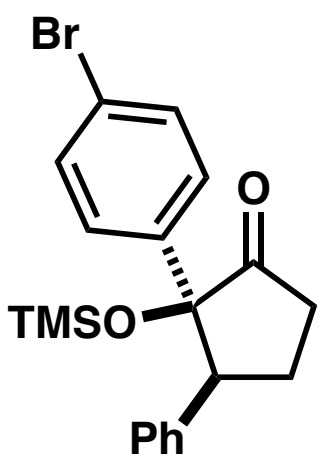

25

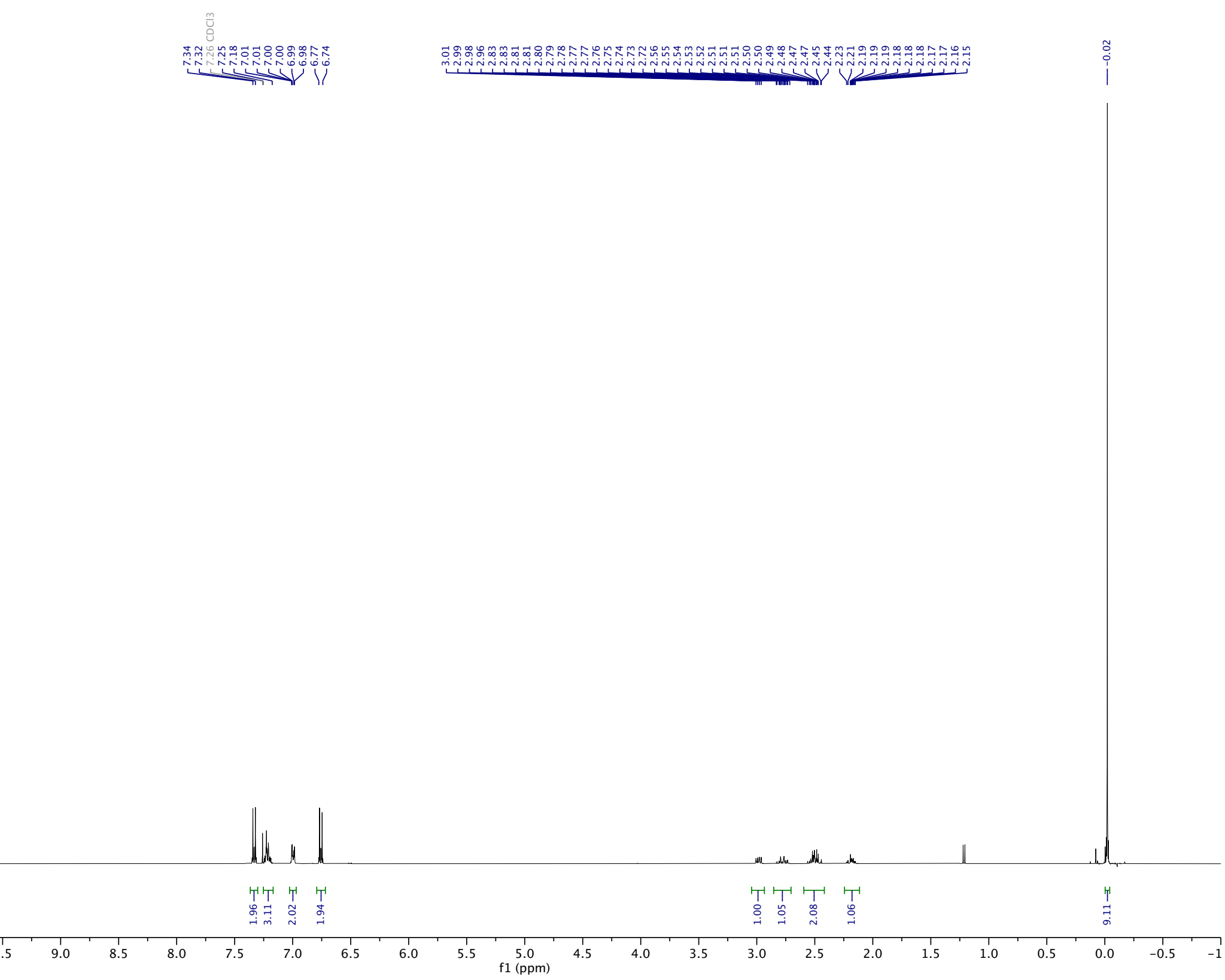

^{1}H NMR (400 MHz, $CDCl_3$) spectrum of compound **25**.

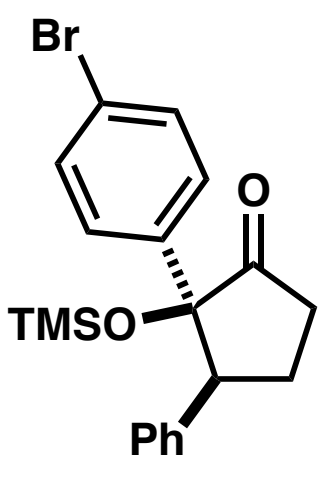

25

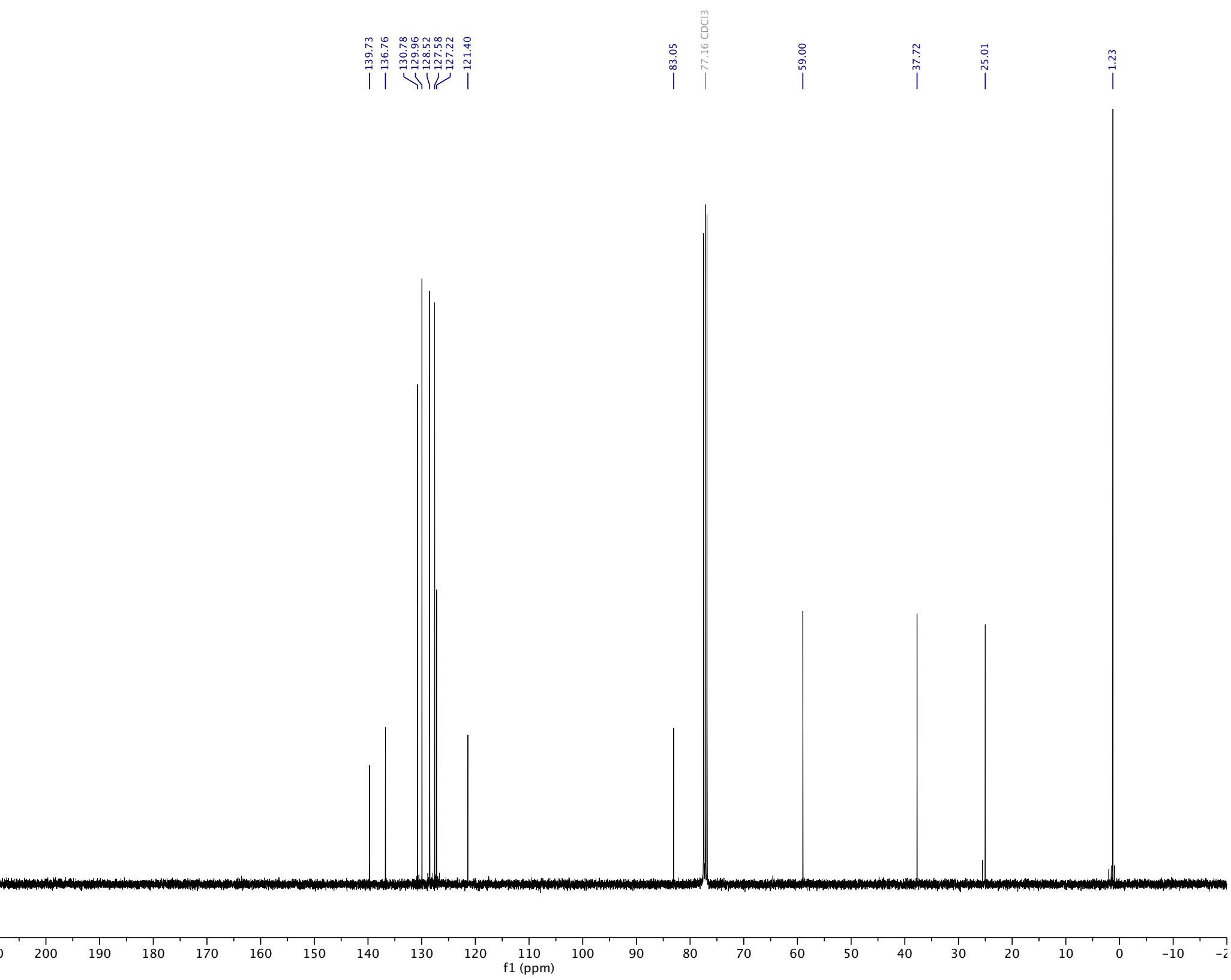

^{13}C NMR (101 MHz, $CDCl_3$) spectrum of compound **25**.

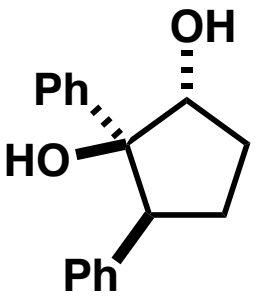

26

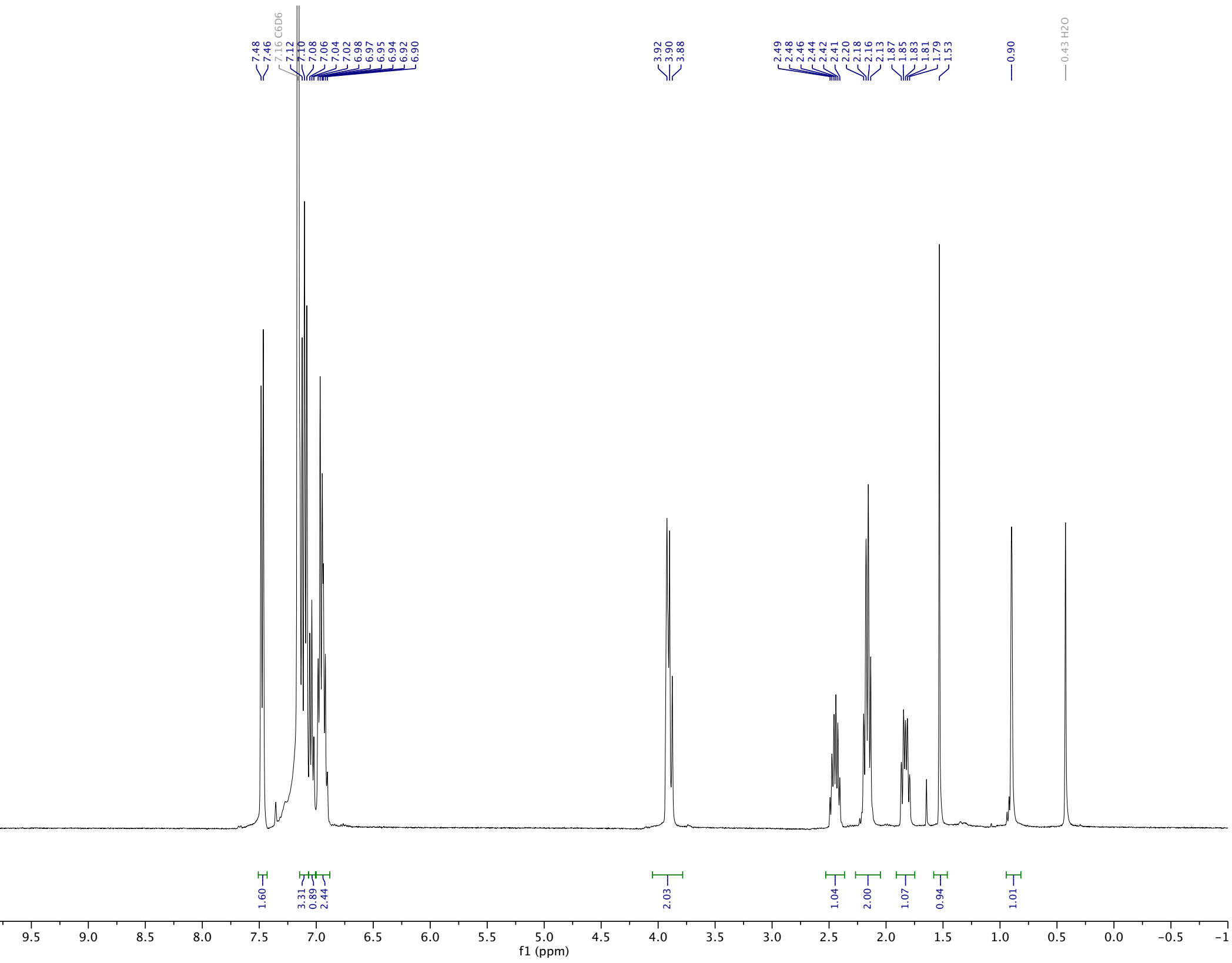

^1H NMR (400 MHz, C_6D_6) spectrum of compound **26**.

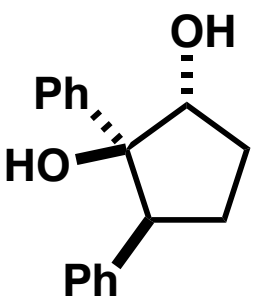

26

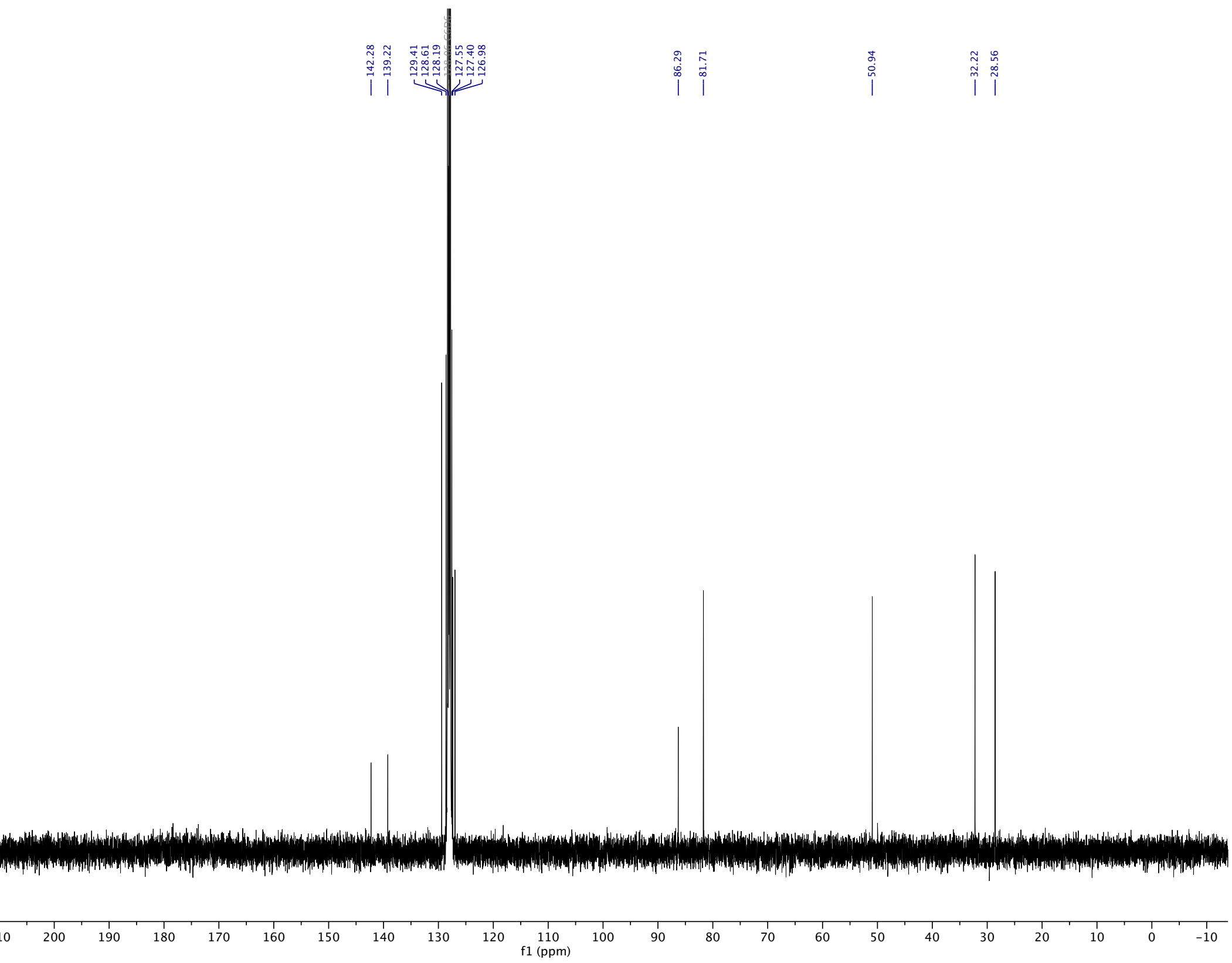

^{13}C NMR (101 MHz, C_6D_6) spectrum of compound **26**.

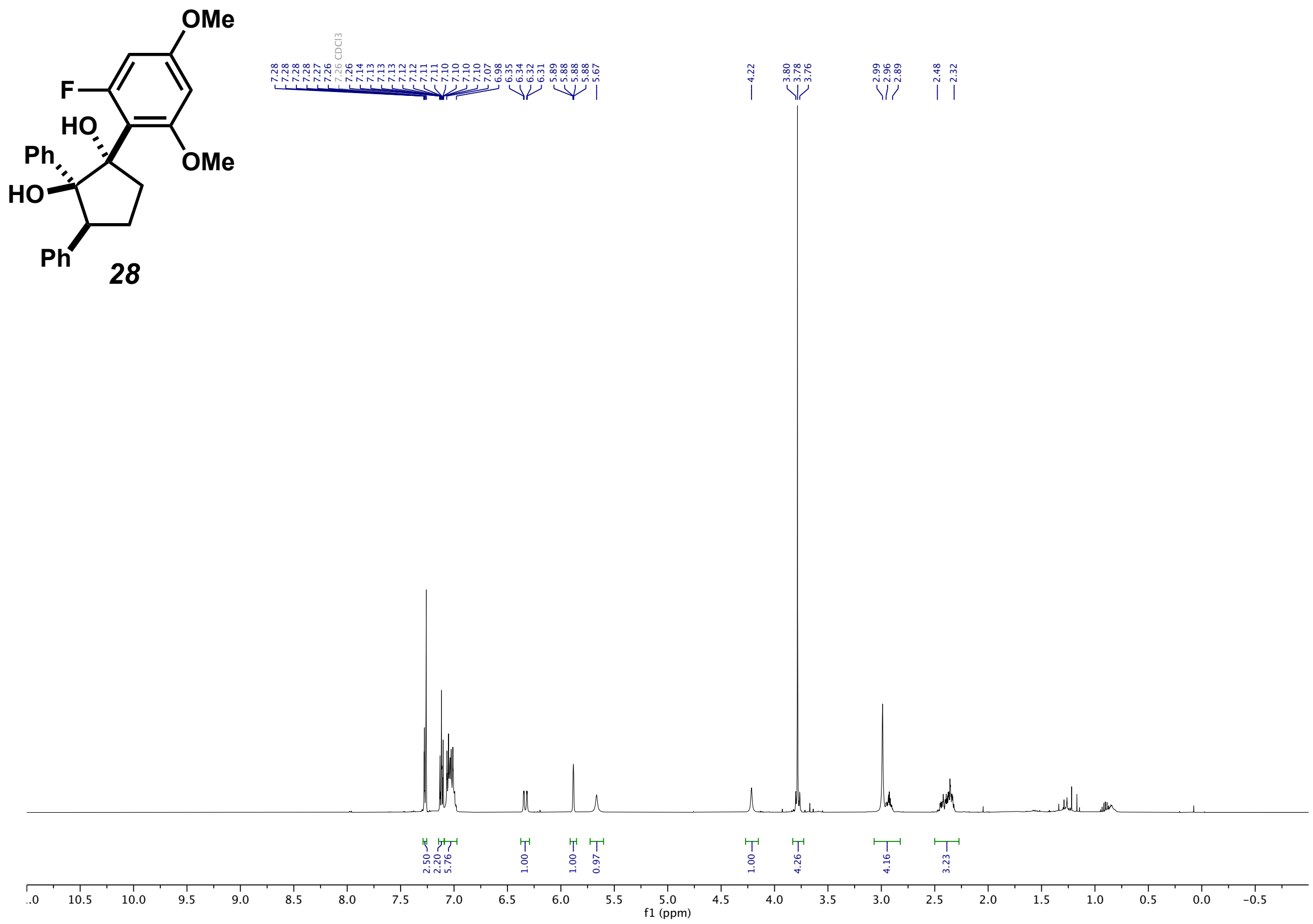

^{1}H NMR (400 MHz, $CDCl_3$) spectrum of compound **28**.

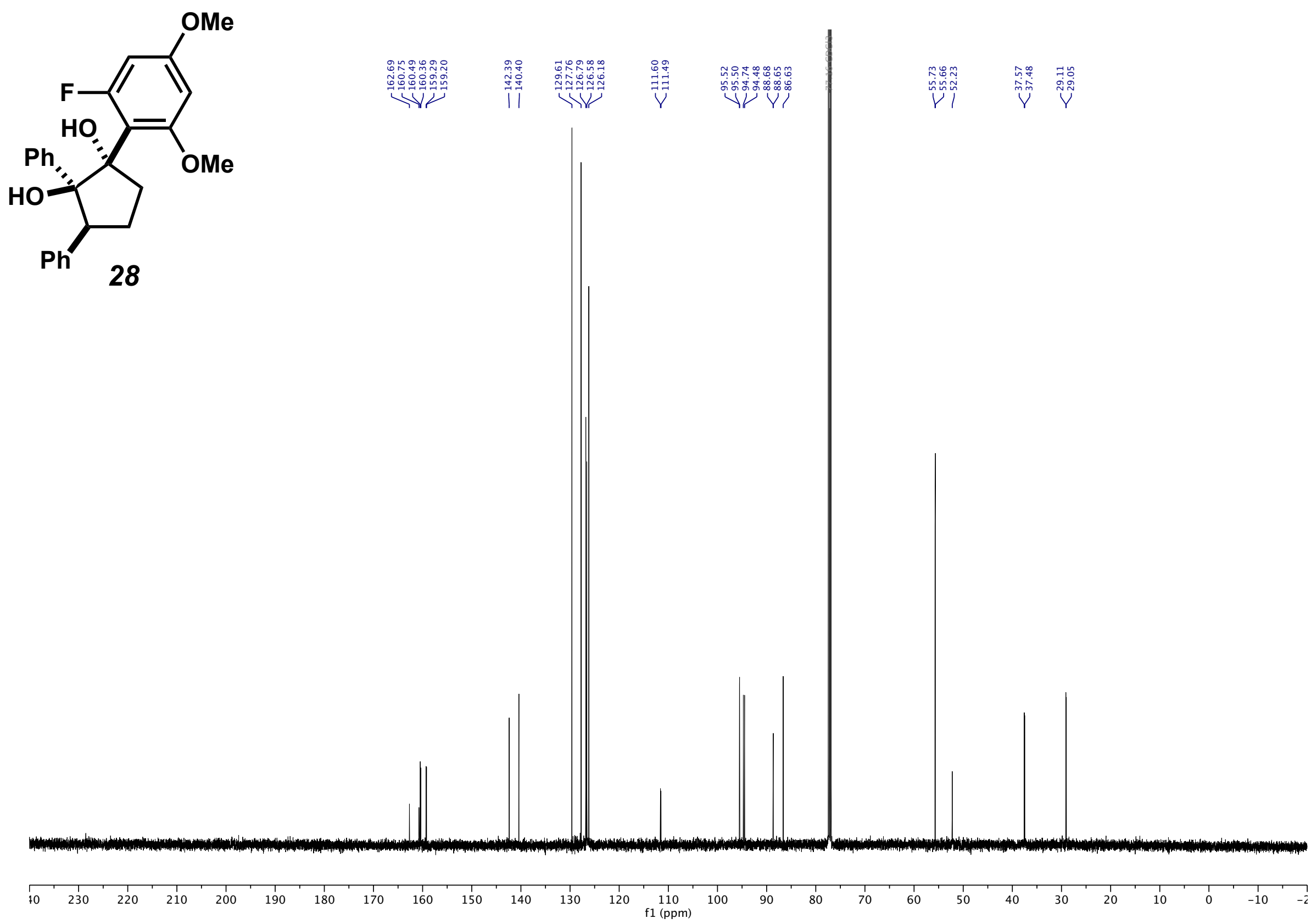

^{13}C NMR (101 MHz, $CDCl_3$) spectrum of compound **28**.

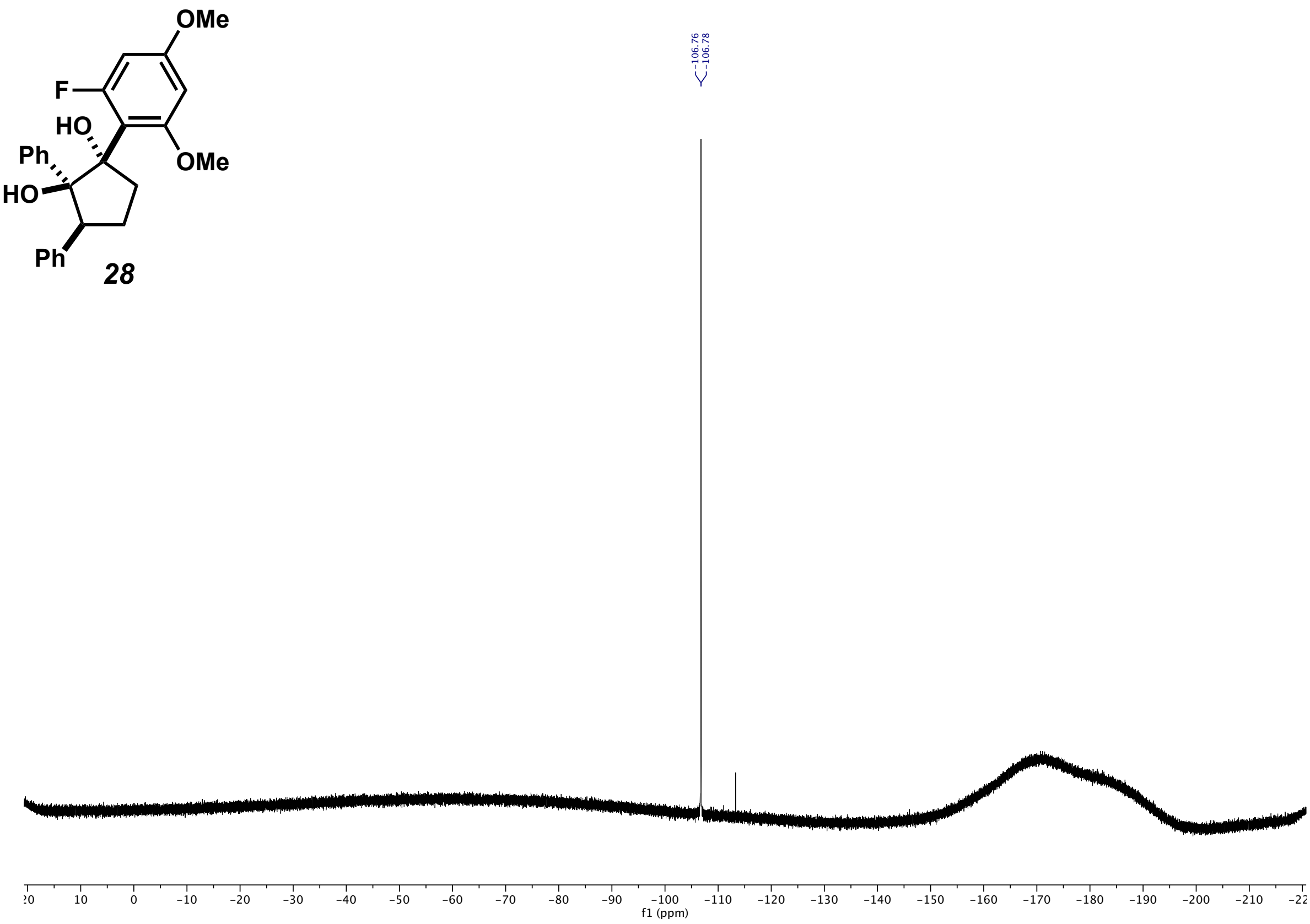

^{19}F NMR (470.7 MHz, $CDCl_3$) spectrum of compound **28**.

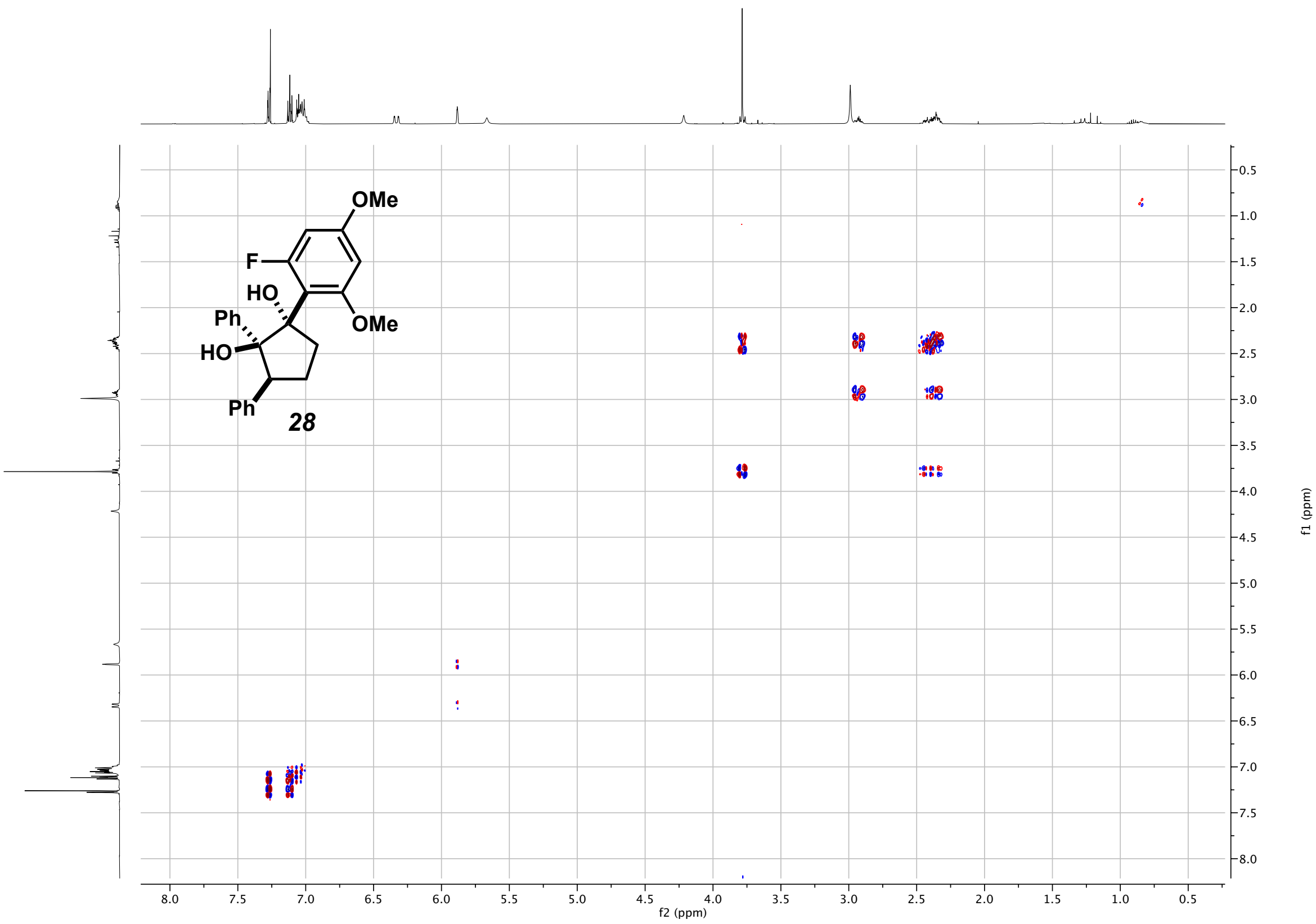

DQF-HH-COSY spectrum of compound **28**.

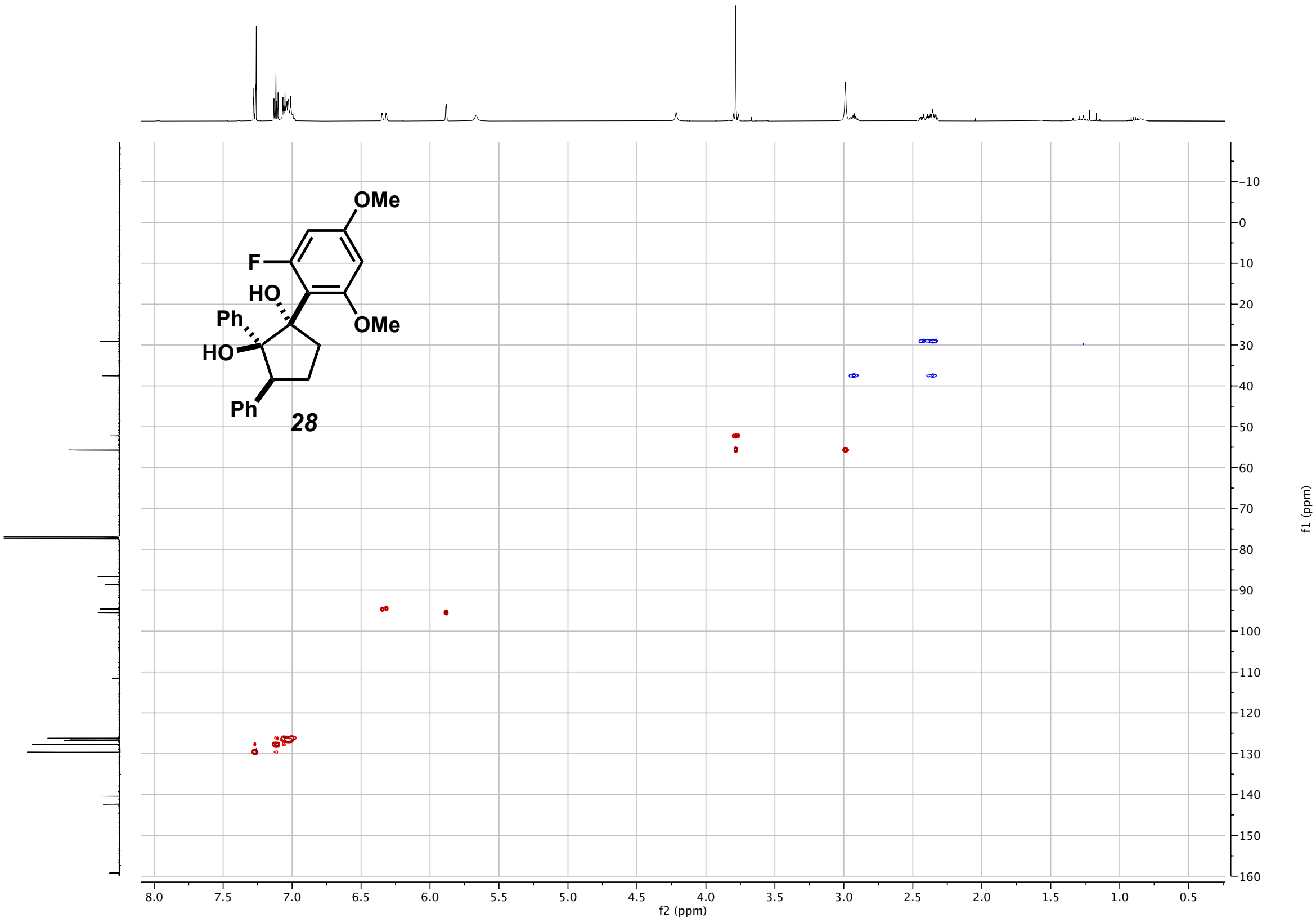

HSQC spectrum of compound **28**.

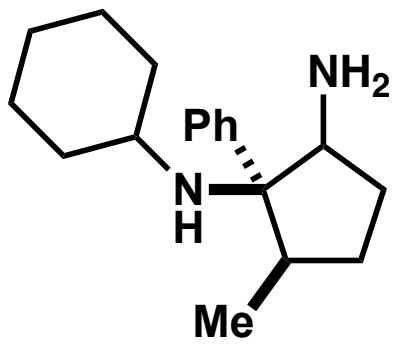

31
(6:1 d.r.)

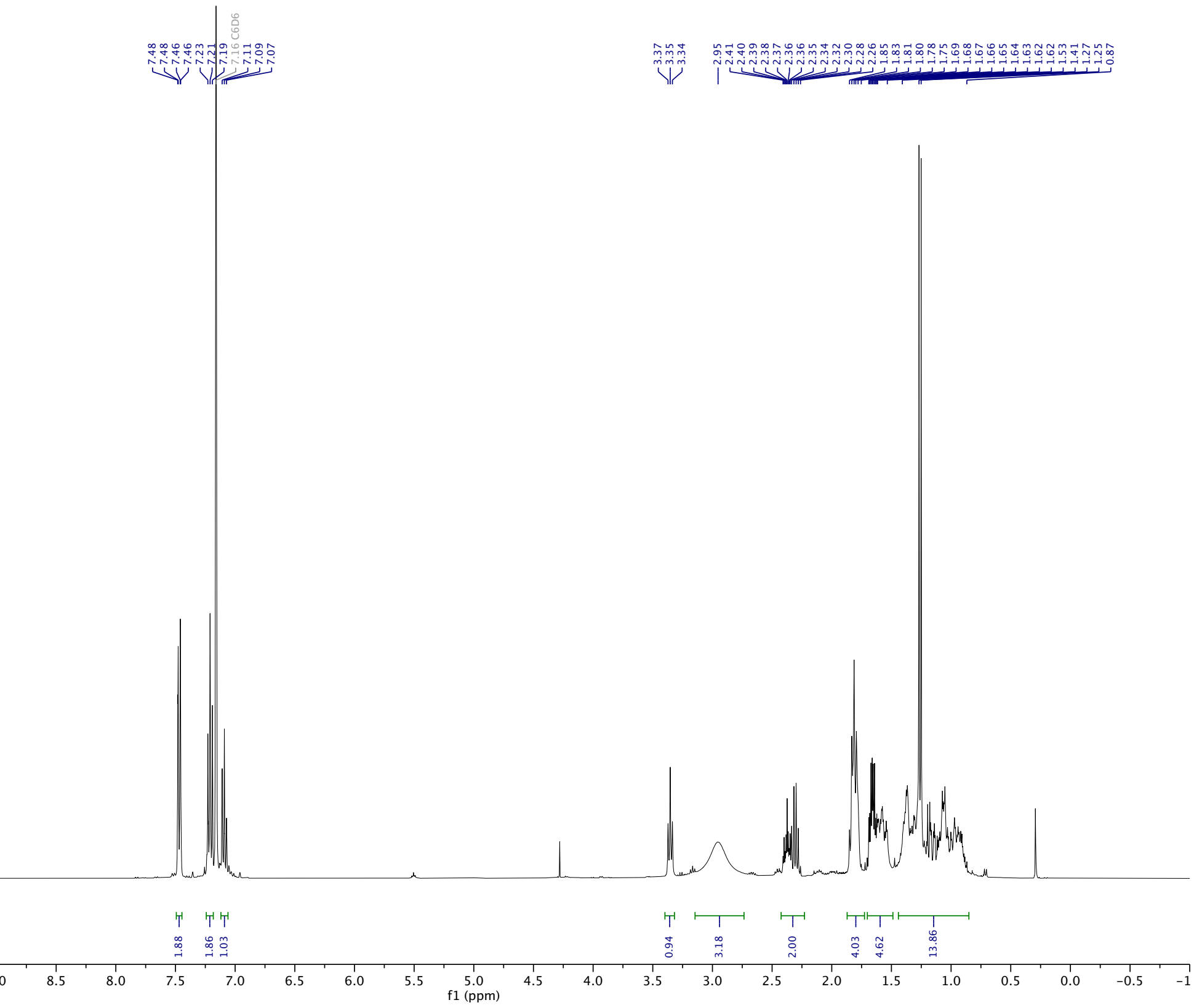

^{1}H NMR (400 MHz, C_6D_6) spectrum of compound **31**.

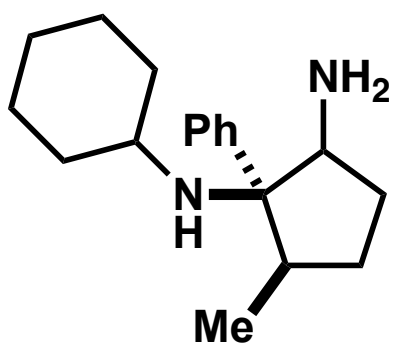

31
(6:1 d.r.)

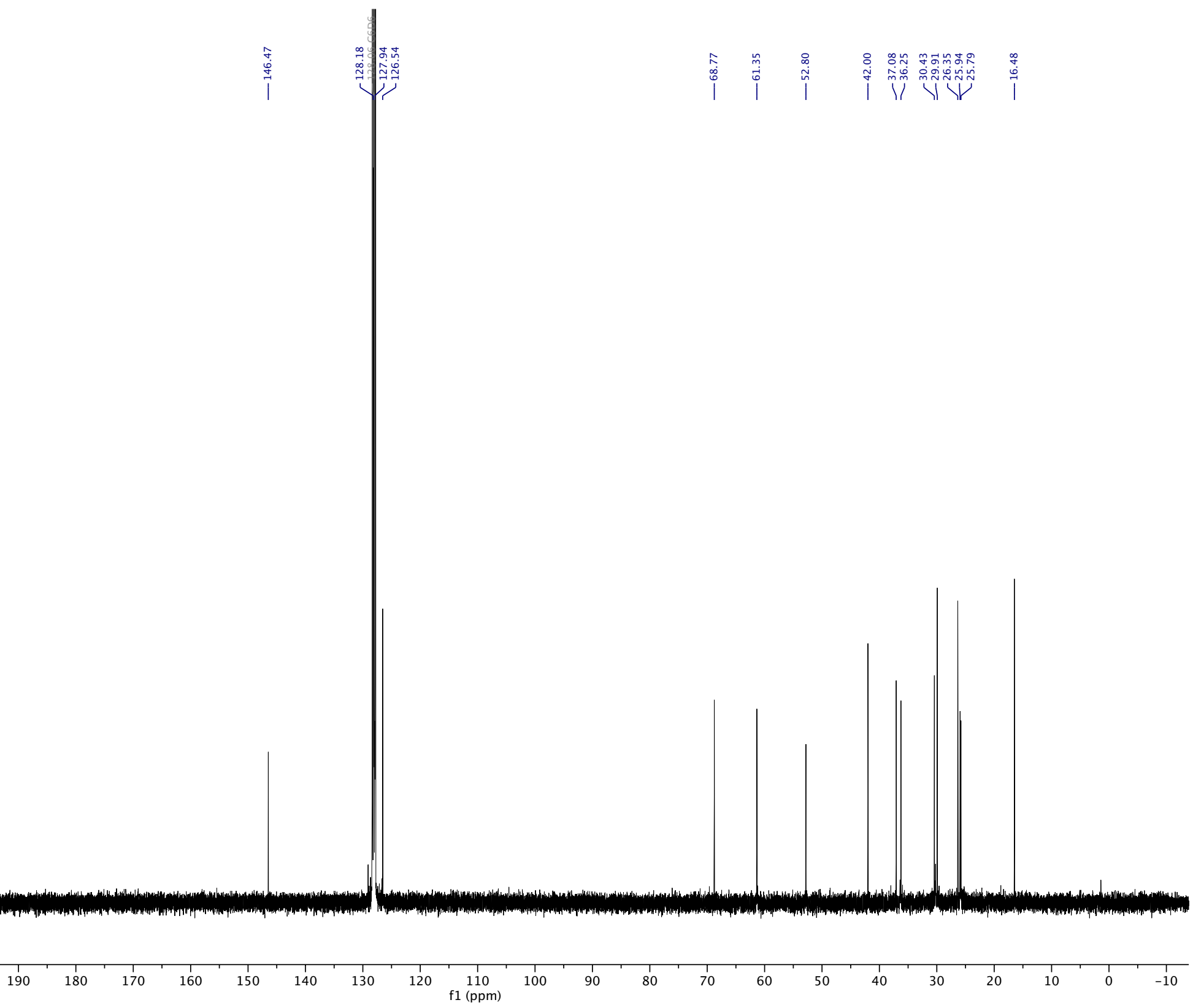

^{13}C NMR (101 MHz, C_6D_6) spectrum of compound **31**.

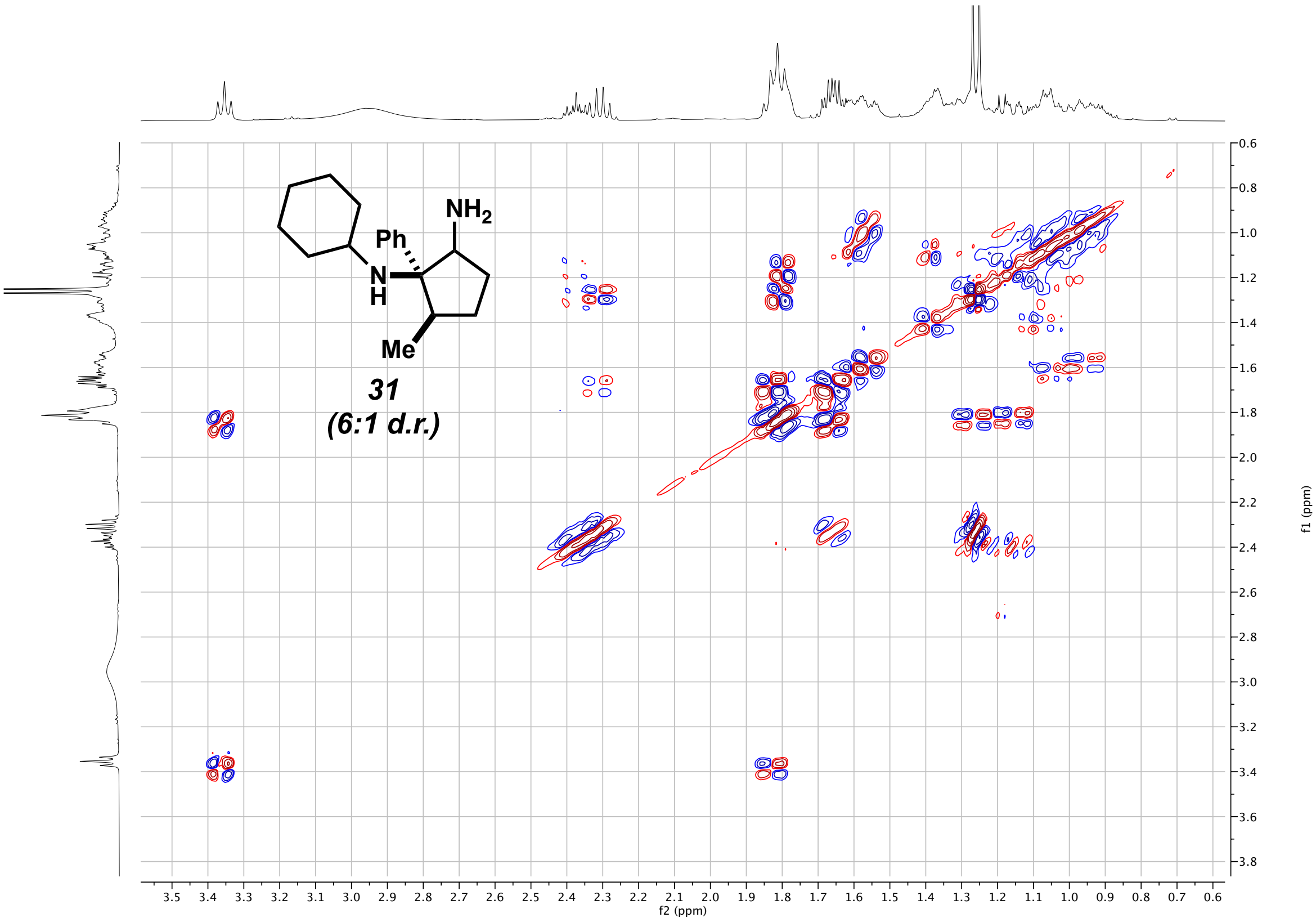

DQCOSY NMR spectrum of compound **31.**

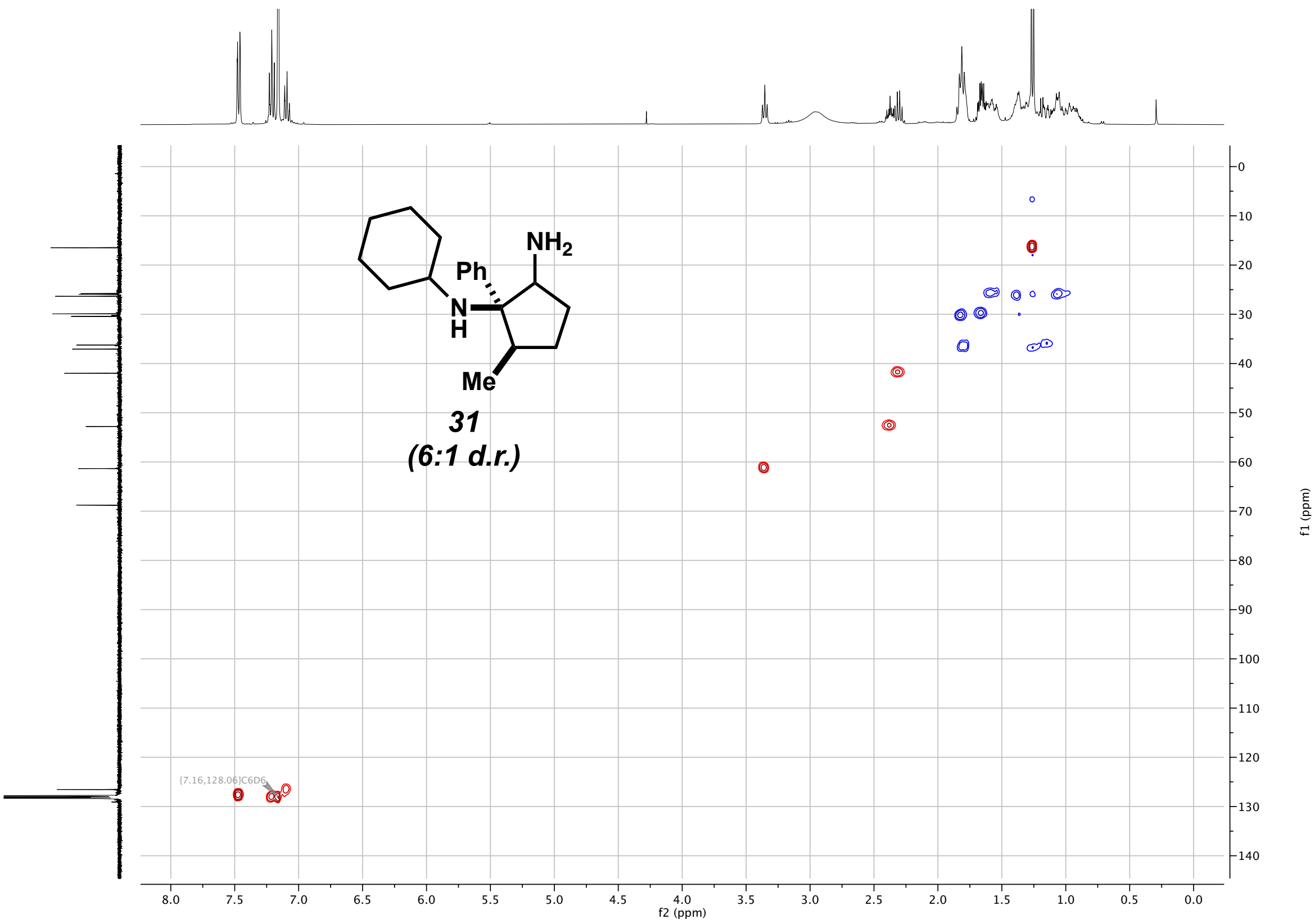

HSQC NMR spectrum of compound **31.**

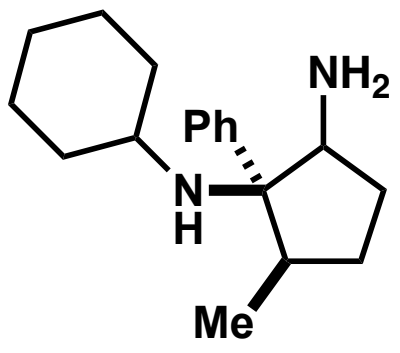

31
(6:1 d.r.)

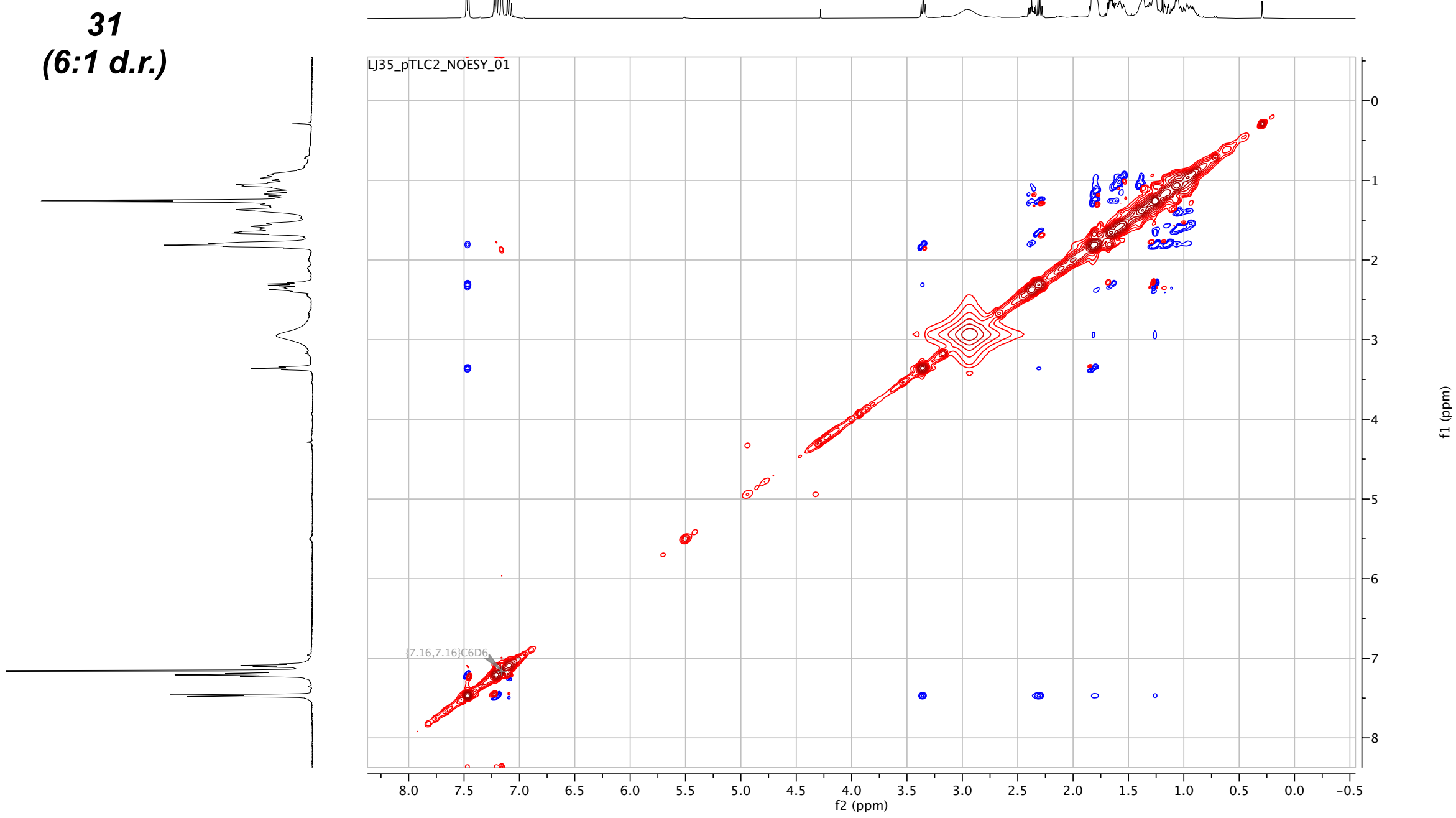

NOESY NMR spectrum of compound **31.**

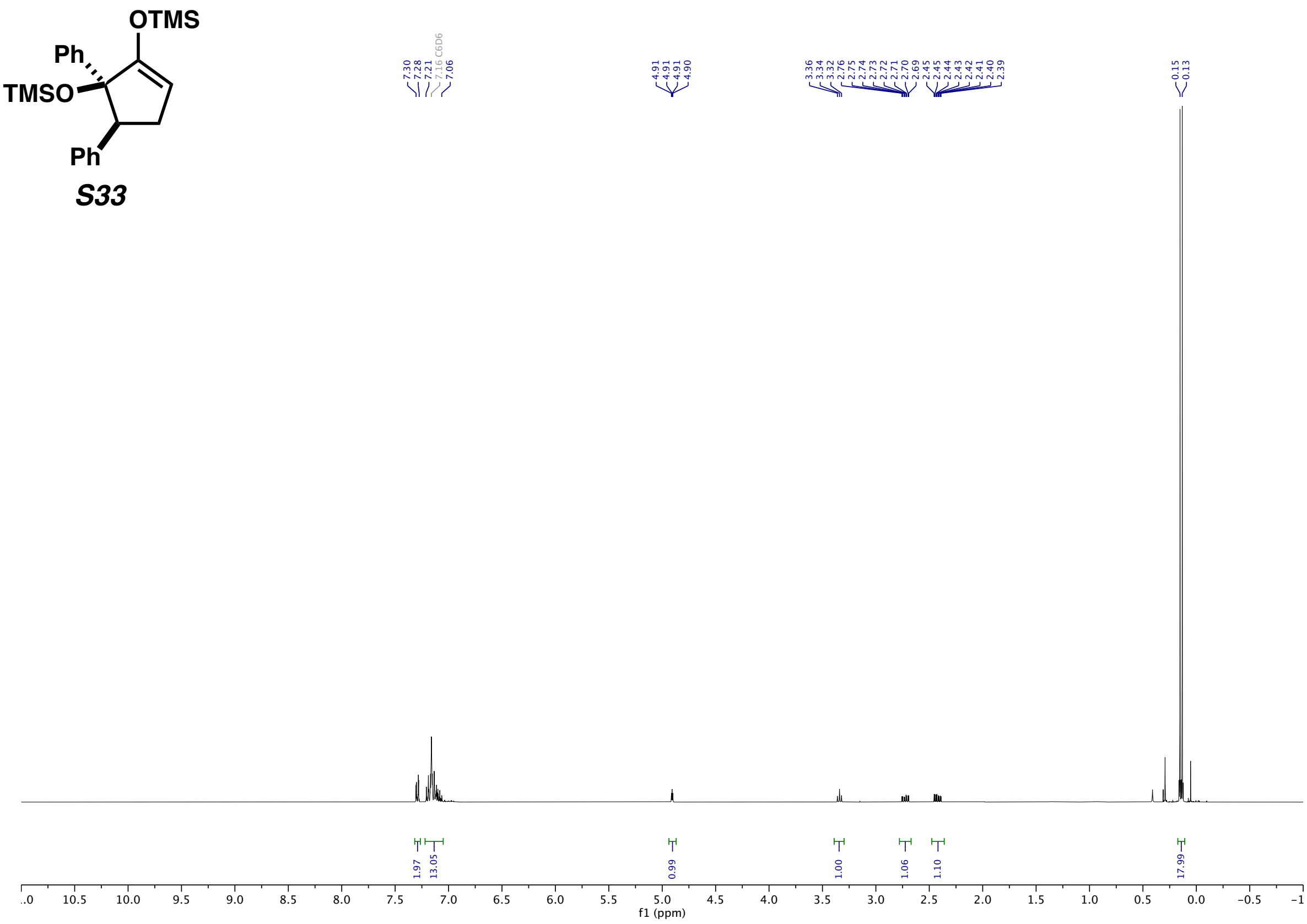

^{1}H NMR (400 MHz, C_6D_6) spectrum of compound **S33**.

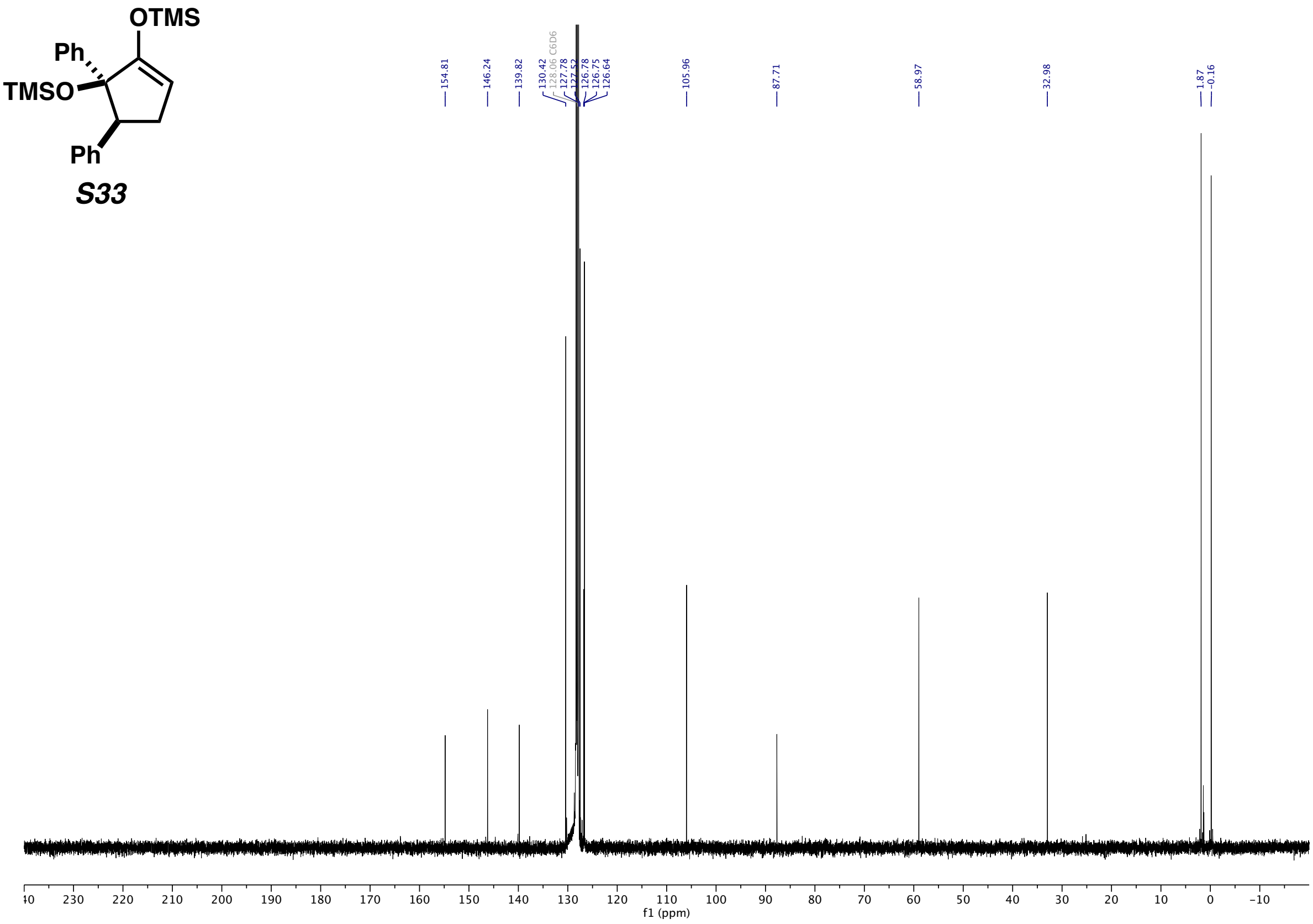

^{13}C NMR (101 MHz, C_6D_6) spectrum of compound **S33**.

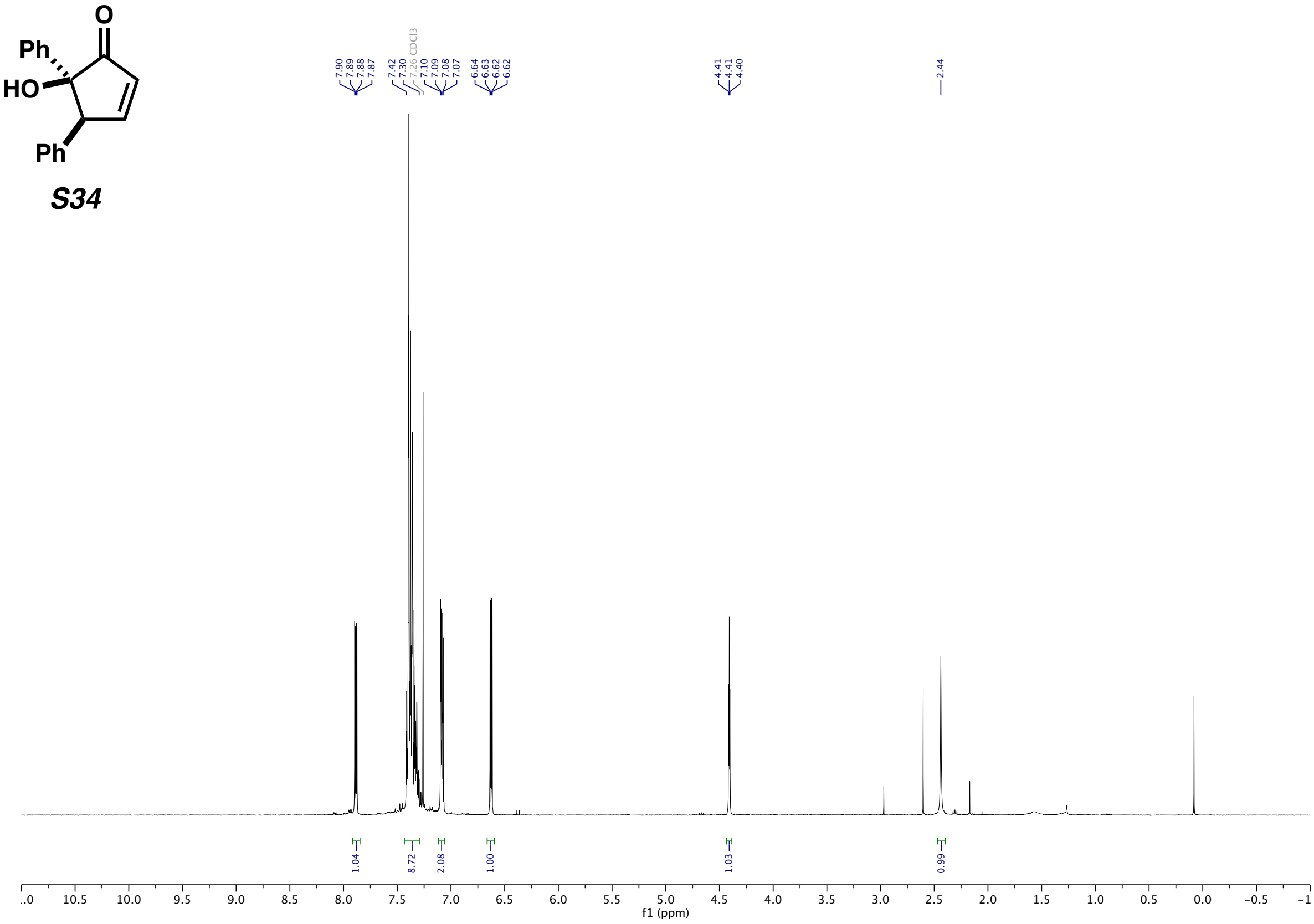

S34

^{1}H NMR (400 MHz, $CDCl_3$) spectrum of compound **S34**.

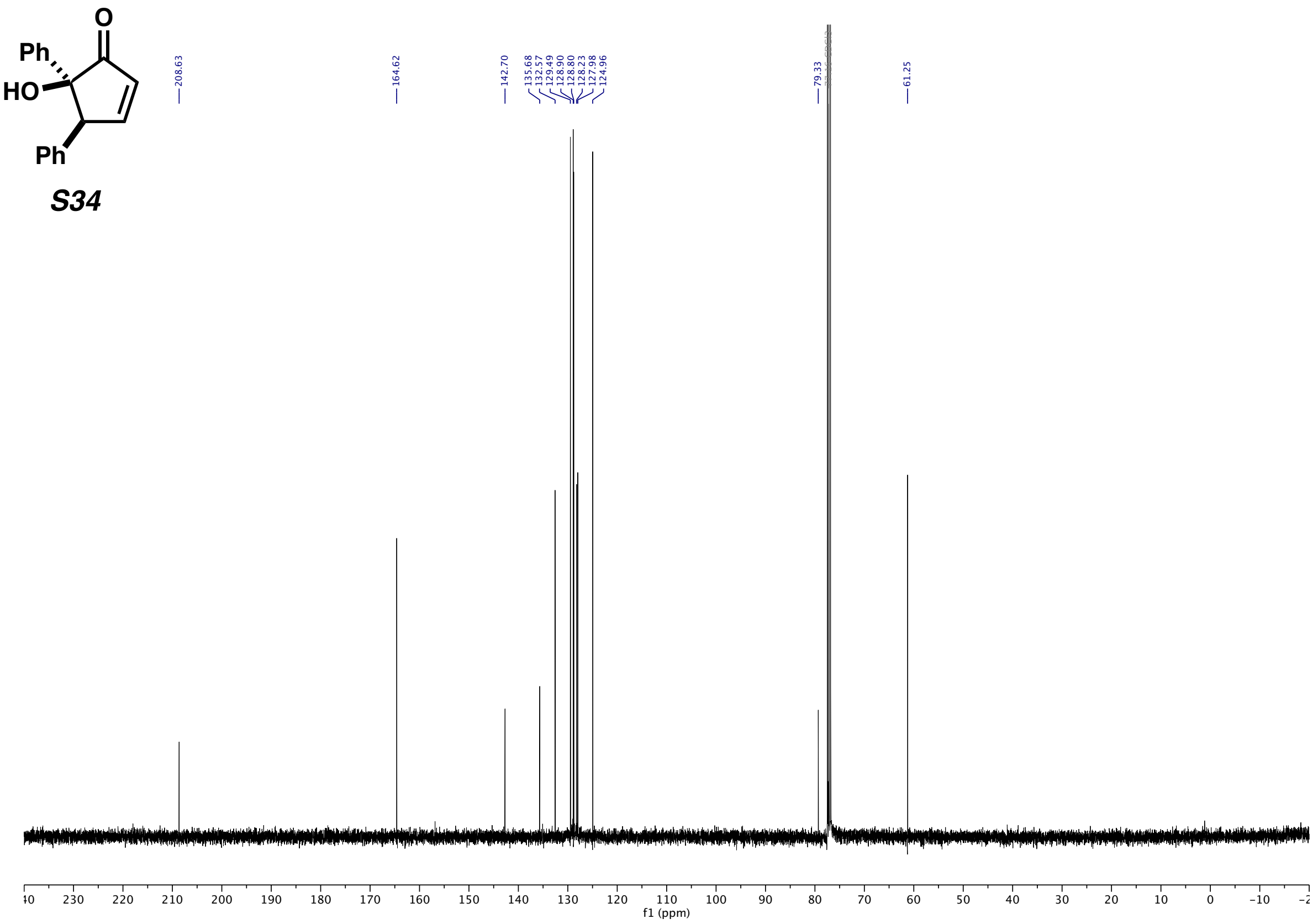

S34

^{13}C NMR (101 MHz, $CDCl_3$) spectrum of compound **S34**.

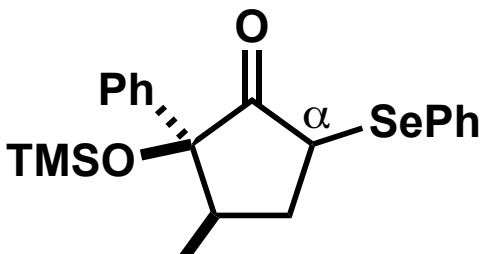

32

(Cα: 6.2:1 d.r.)

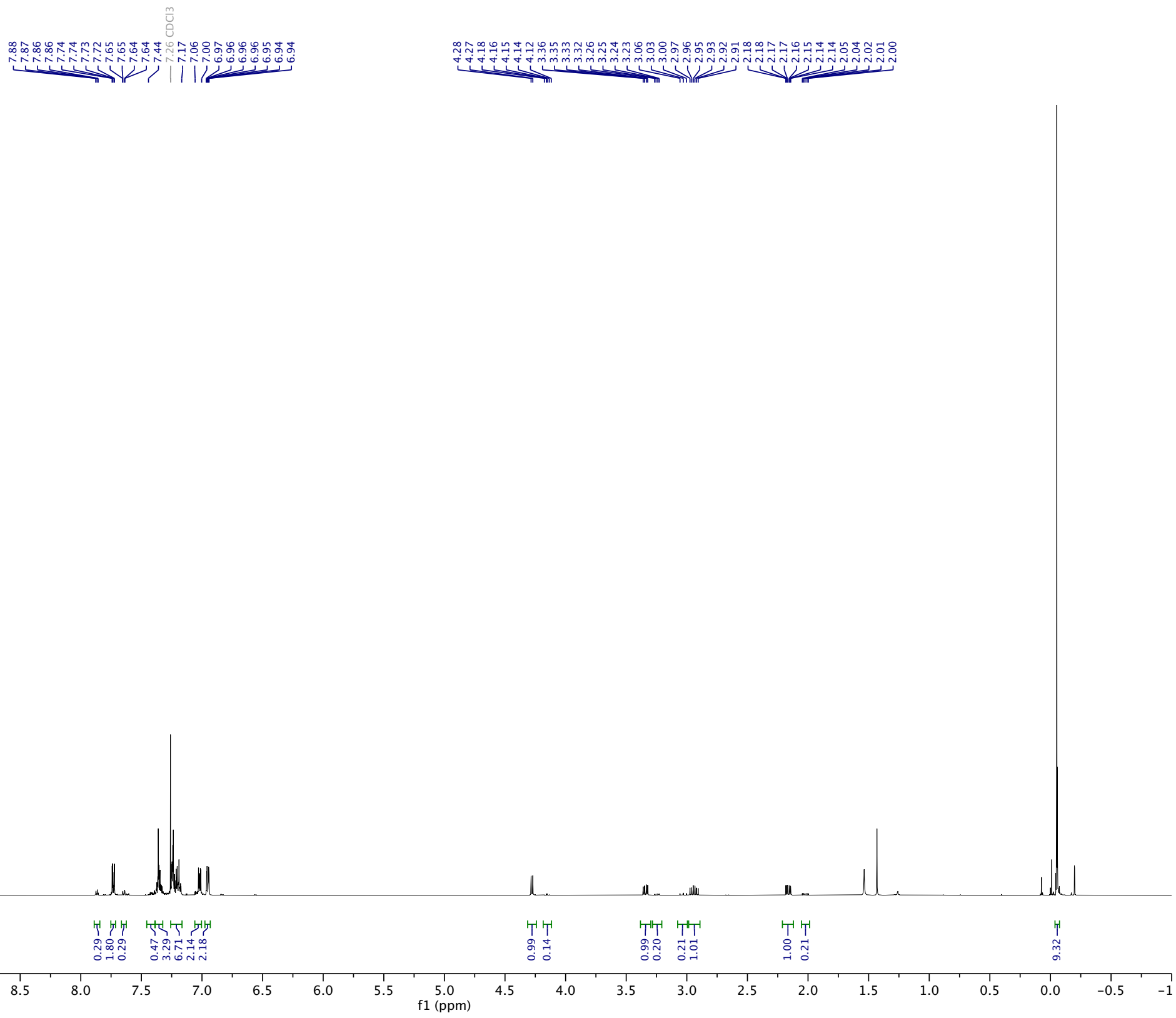

^{1}H NMR (500 MHz, $CDCl_3$) spectrum of compound **32**.

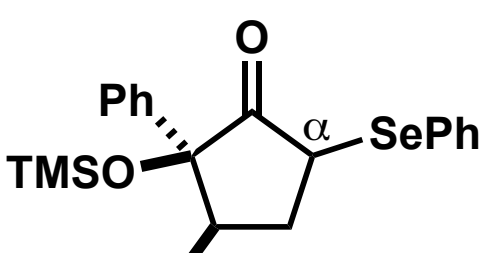

32

(Cα: 6.2:1 d.r.)

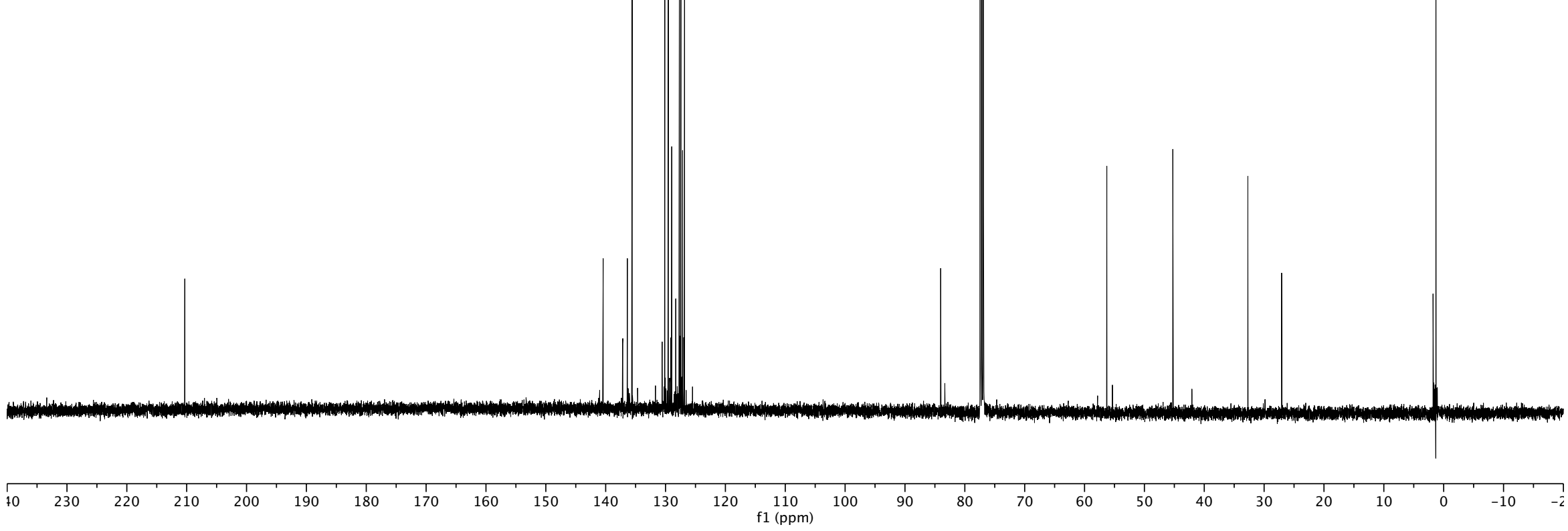

^{13}C NMR (125 MHz, $CDCl_3$) spectrum of compound **32**.

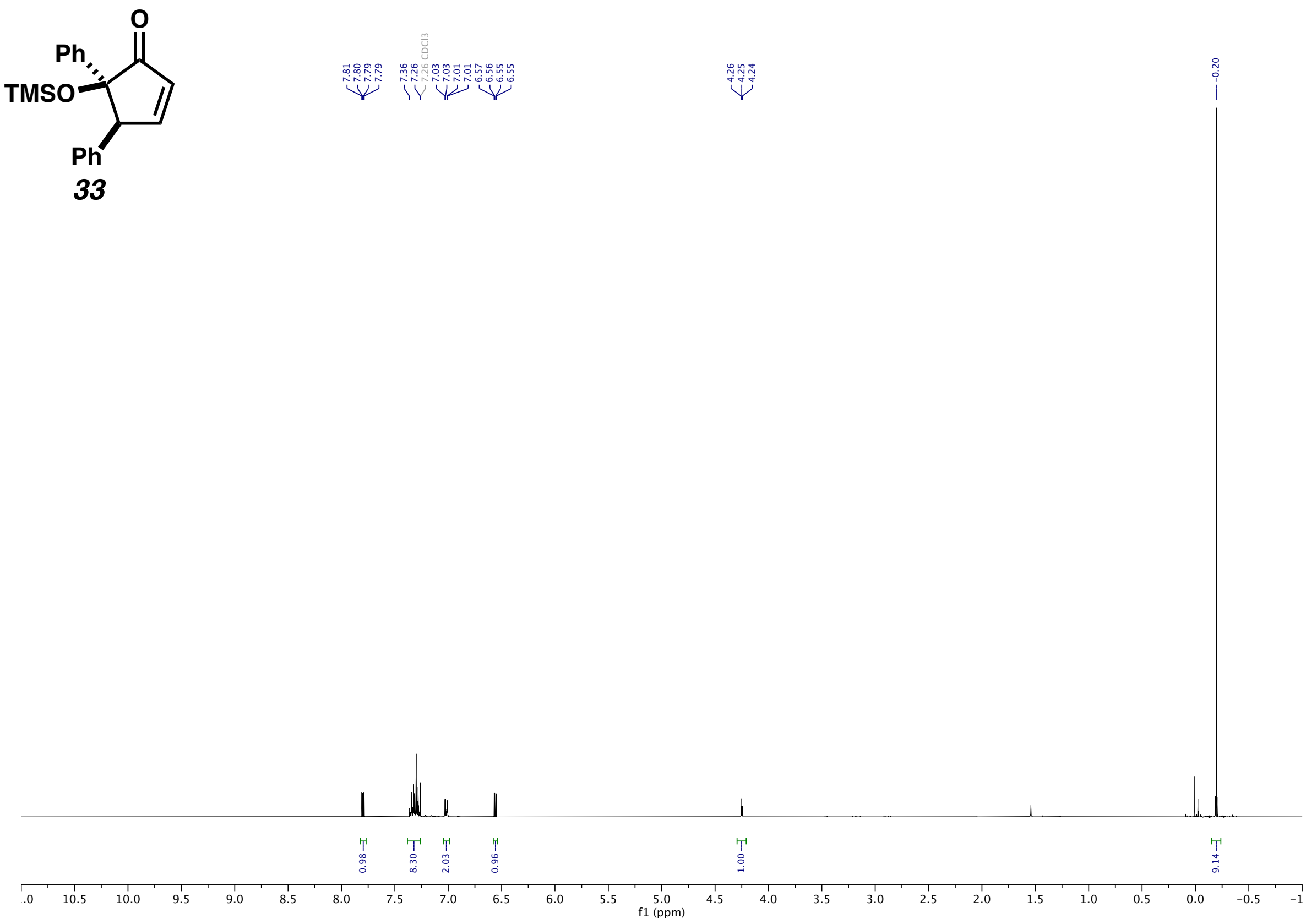

^{1}H NMR (400 MHz, $CDCl_3$) spectrum of compound **33**.

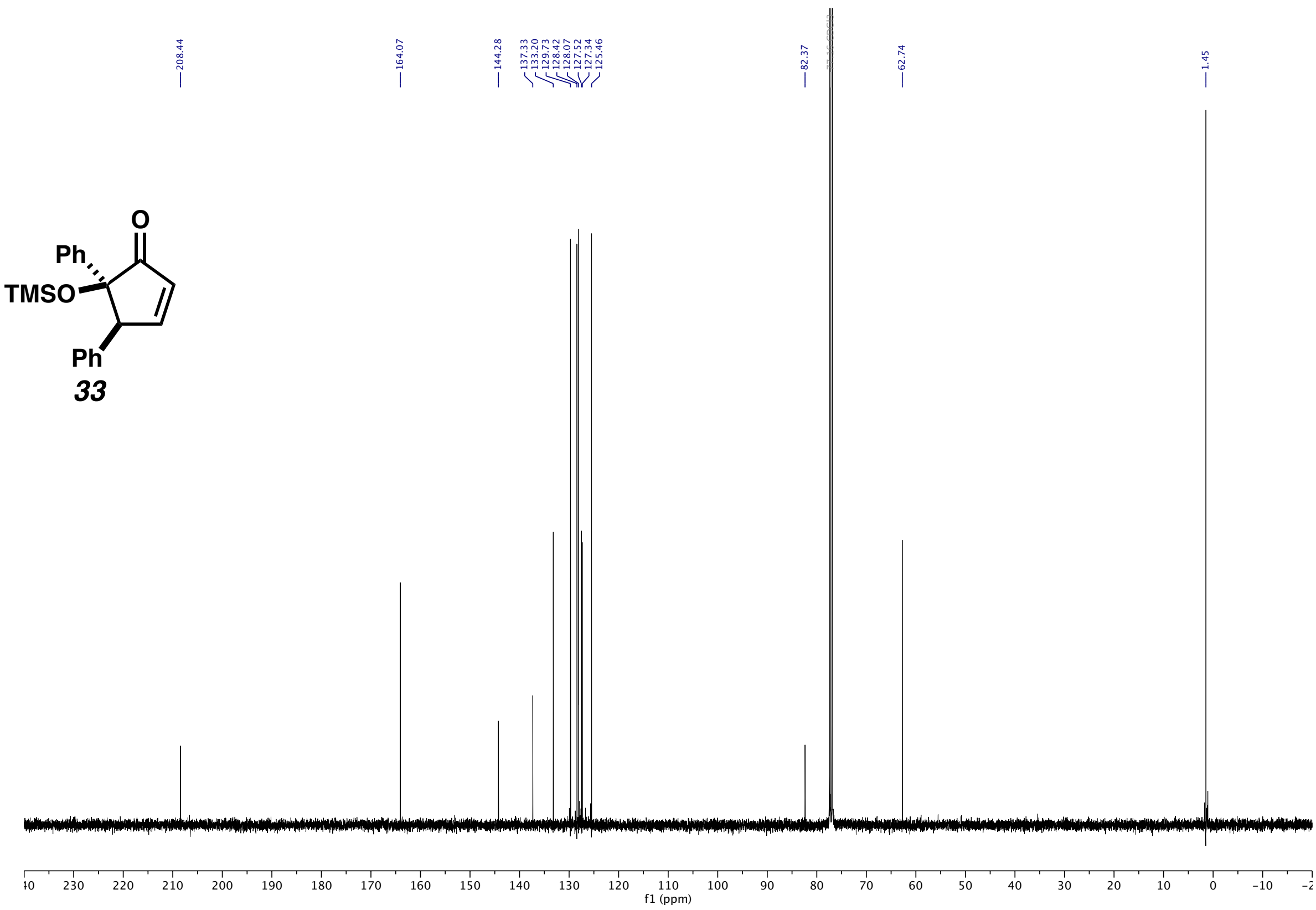

^{13}C NMR (101 MHz, $CDCl_3$) spectrum of compound **33**.

References

[1] J. Gordon, S. Hildebrandt, K. R. Dewese, S. Klare, A. Gansäuer, T. V. RajaBabu, W. A. Nugent, *Organometallics* **2018**, *37*, 4801–4809.

[2] M. J. Di Grandi, C. Bennett, K. Cagino, A. Muccini, C. Suraci, S. Saba, *Synth. Commun.* **2015**, *45*, 2601–2607.

[3] K. Zumbansen, A. Döhring, B. List, *Adv. Synth. Catal.* **2010**, *352*, 1135–1138.

[4] H. G. Steeds, J. P. Knowles, W. L. Yu, J. Richardson, K. G. Cooper, K.I. Booker-Milburn, *Chem. Eur. J.* **2020**, *26*,14330–14334.

[5] T. Drennhaus, L, Öhler, S. Djalali, S. Höfmann, C. Müller, J. Pietruszka D. Worgull, *Adv. Synth. Catal.* **2020**, *362*, 2385–2396.

[6] H. Lütjens, A. Zickgraf, H. Figler, J. Linden, R. A. Olsson, P. J. Scammells, *J. Med. Chem.* **2003**, *46*, 1870–1877.

[7] D. P. Sangi, M. R. Cominetti, A. B. Becceneri, F. A. Resende, E. A. Varanda, C. A. Montanari, M. W. Paixao, A. G. Correa, *Med. Chem.* **2015**, 11, 736–746.

[8] K. Pal, M. L. Behnke, L. Tong, *Tetrahedron Lett.* **1993**, *34*, 6205–6208.

[9] Y. Li, B. Liu, H.-B. Li, Q. Wang, J.-H. Li, *Chem. Commun.* **2015**, *51*, 1024–1026.

[10] J. Vaitla, A. Bayer, K. H. Hopmann, *Angew. Chem. Int. Ed.* **2017**, *56*, 4277–4281.

[11] A. I. Khalaf, J. K. Huggan, C. J. Suckling, C. L. Gibson, K. Stewart, F. Giordani, M. P. Barrett, P. E. Wong, K. L. Barrack, W. N. Hunter, *J. Med. Chem.* **2014**, *57*, 6479–6494.

[12] A. Takemiya, J. F. Hartwig, *J. Am. Chem. Soc.* **2006**, *128*, 14800–14801.

[13] R. Gust, V. Lubczyk, *J. Steroid Biochem. Mol. Biol.* **2003**, *86*, 57–70.

[14] R. B. Chhor, K. A. Singh, B. Nosse, V. K. Tandon, *Synth. Commun.* **2003**, *33*, 2519–2530.

[15] H.-P. Lin, N. Ibrahim, O. Provot, M. Alami, A. Hamze, *RSC Adv.* **2018**, *8*, 11536–11542.

[16] R. B. Watson, C. S. Schindler, *Org. Lett.* **2018**, *20*, 68–71.

[17] M. Luo, Y. Zhang, P. Fang, Y. Li, C. Qi, Y. Li, R. Shen, K. Cheng, H. Wang, *Org. Biomol. Chem.* **2022**, *20*, 630–635.

[18] J. Mazuela, T. Antonsson, L. Knerr, S. P. Marsden, R. H. Munday, M. J. Johansson, *Adv. Synth. Catal.* **2019**, *361*, 578–584.

[19] Y. Kang, R. Hou, X. Min, T. Wang, Y. Liang, Z. Zhang, *Synthesis* **2022**, *54*, 1621–1632.

[20] B. Shu, X.-T. Wang, Z.-X. Shen, T. Che, M. Zhong, J.-L. Song, H.J. Kang, H. Xie, L. Zhang, S.-S. Zhang**,** *Org. Chem. Front.* **2020**, *7*, 1802–1808.

[21] T. Miura, S. Fujioka, N. Takemura, H. Iwasaki, M. Ozeki, N. Kojima, M. Yamashita, *Synthesis* **2014**, *46*, 496–502.

[22] C.-C. Tseng, G. Baillie, G. Donvito, M. A. Mustafa, S. E. Juola, C. Zanato, C. Massarenti, S. Dall'Angelo, W. T. A. Harrison, A. H. Lichtman, R. A. Ross, M. Zanda, I. R. Greig, *J. Med. Chem.* **2019**, *62*, 5049–5062.

[23] H. Sharma, M. Kumar, A. Sethi, Poonam, B. Rathi, *Green Chem.* **2023**, *25*, 167–171.

[24] L. Tang, S. Jiang, X. Huang, Z. Song, J.-b. Wang, M. Ma, B. Chen, Y. Ma, *Org. Lett.* **2022**, *24*, 3232–3237.

[25] B. M. Trost, K. Lehr, D. J. Michaelis, J. Xu, A. K. Buckl, *J. Am. Chem. Soc.* **2010**, *132*, 8915–8917.

[26] W.-B. Wu, X. Yu, J.-S. Yu, X.Wang, W.-G. Wang, J. Zhou, *CCS Chem.* **2022**, *4*, 2140–2152.

[27] V. C. Purohit, S. P. Allwein, R. P. Bakale, *Org. Lett.* **2013**, *15*, 1650–1653.

[28] N. Takeda, M. Furuishi, Y. Nishijima, E. Futaki, M. Ueda, T. Shinada, O. Miyata *Org. Biomol. Chem.*, **2018**, *16*, 8940–8943.

[29] L. Cotarca, P. Delogu, A. Nardelli, P. Maggioni, R. Bianchini, S. Sguassero, S. Alini, R. Dario, G. Clauti, G. Pitta, G. Duse, F. Goffredi, *Org. Proc. Res. Dev.* **2001**, *5*, 69–76.

[30] M. J. Fink, R. Snajdrova, A. Winninger, M. D. Mihovilovic, *Tetrahedron* **2016**, *72*, 7241–7248.

[31] G. Frey, H.-T. Luu, P. Bichovsky, M. Feurer, J. Streuff, *Angew. Chem. Int. Ed.* **2013**, *52*, 7131–7134.

[32] a) R. C. Gadwood, I. M. Mallick, A. J. DeWinter, *J. Org. Chem.* **1987**, *52*, 774–782. b) A. Gao, X.-Y. Liu, C.-H. Ding, X.-L. Hou, *Synlett* **2017**, *28*, 2829–2832.

[33] N. K. Hamer, *J. Chem. Soc., Perkin Trans. 1* **1979**, 1285–1289.

[34] a) G. A. Molander, C. N. Wolfe, *J. Org. Chem.* **1998**, *63*, 9031–9036. b) N. Saito, Y. Sugimura, Y. Sato, *Org. Lett.* **2010**, *12*, 3494–3497.

[35] J. Streuff, M. Feurer, P. Bichovski, G. Frey, U. Gellrich, *Angew. Chem. Int. Ed.* **2012**, *51*, 8661–8664.

[36] Z. Zhou, M. A. Tius, *Angew. Chem. Int. Ed.* **2015**, *54*, 6037–6040.

[37] R. Liffert, J. Hoecker, C. K. Jana, T. M. Woods, P. Burch, H. J. Jessen, M. Neuburger, K. Gademann, *Chem. Sci.* **2013**, *4*, 2851–2857.

[38] M. A. Bau, *Master Thesis*, University of Freiburg, Freiburg, **2015**.
[39] a) F. Neese, *Wiley Interdiscip. Rev.: Comput. Mol. Sci.* **2017**, *8*, e1327. b) F. Neese, F. Wennmohs, U. Becker, C. Riplinger, *J. Chem. Phys.* **2020**, *152*, 224108. c) F. Neese, *Wiley Interdiscip. Rev.: Comput. Mol. Sci.* **2022**, *12*, e1606.
[40] a) S. Grimme, S. *J. Chem. Theory Comput.* **2019**, *15*, 2847–2862. b) P. Pracht, F. Bohle, S. Grimme, *Phys. Chem. Chem. Phys.* **2020**, *22*, 7169–7192. c) CREST version 2.11, University Bonn, Mulliken Center for Theoretical Chemistry, Bonn, Germany **2021**, https://github.com/grimme-lab/crest/releases. d) XTB version 6.4.0, University of Bonn, Mulliken Center for Theoretical Chemistry, Bonn, Germany **2021**, https://github.com/grimme-lab/xtb. e) S. Grimme, F. Bohle, A. Hansen, P. Pracht, S. Spicher, M. Stahn *J. Phys. Chem. A* **2021**, *125*, 4039–4054
[41] S. Grimme, A. Hansen, S. Ehlert, J.-M. Mewes, *J. Chem. Phys.* **2021**, *154*, 064103.
[42] a) J. W. Furness, A. D. Kaplan, J. Ning, J. P. Perdew, J. Sun, *J. Phys. Chem. Lett.* **2020**, *11*. 8208. b) J. W. Furness, A. D. Kaplan, J. Ning, J. P. Perdew, J. Sun, *J. Phys. Chem. Lett.* **2020**, *11*, 9248.
[43] H. Kruse, S. Grimme, *J. Chem. Phys.* **2012**, *136*, 154101.
[44] a) E. Caldeweyher, C. Bannwarth, S. Grimme, *J. Chem. Phys.* **2017**, *147*, 034112. b) E. Caldeweyher, S. Ehlert, A. Hansen, H. Neugebauer, S. Spicher, C. Bannwarth, S. Grimme, *J. Chem. Phys.* **2019**, *150*, 154122.
[45] M. Cossi, N. Rega, G. Scalmani, V. Barone, *J. Comput. Chem.* **2003**, *24*, 669-681.
[46] Y. Zhao, D. G. Truhlar, *J. Phys. Chem. A* **2005**, *109*, 5656–5667.
[47] F. Weigend, R. Ahlrichs, *Phys. Chem. Chem. Phys.* **2005**, *7*, 3297–3305.
[48] F. Weigend, *Phys. Chem. Chem. Phys.* **2006**, *8*, 1057–1065.
[49] a) L. Eichkorn, O. Treutler, H. Öhm, M. Häser, R. Ahlrichs, *Chem. Phys. Lett.* **1995**, *240*, 283–290. b) K. Eichkorn, F. Weigend, O. Treutler, R. Ahlrichs, *Theor. Chem. Acc.* **1997**, *97*, 119–124. c) R. Bauernschmitt, M. Häser, O. Treutler, R. Ahlrichs, *Chem. Phys. Lett.* **1997**, *264*, 573–578. d) P. Deglmann, K. May, F. Furche, R. Ahlrichs, *Chem. Phys. Lett.* **2004**, *384*, 103–107. e) C. K. Skylaris, L. Gagliardi, N. C. Handy, G. Ioannou, S. Spencer, A. Willets, *J. Mol. Struct. (THEOCHEM)* **2000**, *501*, 229–239.
[50] F. Neese, F. Wennmohs, A. Hansen, U. Becker, *Chem. Phys.* **2009**, *356*, 98–109.
[51] CYLview 2.0 (Build0001), C. Y. Legault, Université de Sherbrooke, **2020**. https://www.cylview.org (accessed 2026-05-21).
[52] a) J.-M. Mouesca, in *Metalloproteins, Methods and Protocols*, Eds.: J. C. Fontecilla-Camps, Y. Nicolet, Springer, New York, **2014**, 269–296. b) L. Noodleman, *J. Chem. Phys.* **1981**, *74*, 5737–5743. c) L. Noodleman, J. G. Norman, Jr., *J. Chem. Phys.* **1979**, *70*, 4903–4906. d) P. S. Bagus, B. I. Bennett, *Int. J. Quantum Chem.* **1975**, *9*, 143–148.
[53] J. Streuff, D. Himmel, S. L. Younas, *Dalton Trans.* **2018**, *47*, 5072–5082.
